# Design and structural basis of selective nonsteroidal inhibitors of aster cholesterol trafficking

**DOI:** 10.1073/pnas.2617638123

**Published:** 2026-09-01

**Authors:** Hyunsoo Kim, Beatriz Romartinez-Alonso, Xu Xiao, Yajing Gao, John Paul Kennelly, Alessandra Ferrari, Rui Li, Liujuan Cui, Emily Whang, John W. R. Schwabe, Michael E. Jung, Peter Tontonoz

**Affiliations:** ^a^ Department of Chemistry, University of California, Los Angeles, CA 90095; ^b^ Department of Molecular and Cell Biology, Institute of Structural and Chemical Biology, University of Leicester, Leicester LE1 7RH, United Kingdom; ^c^ Department of Pathology and Laboratory Medicine, University of California, Los Angeles, CA 90095; ^d^ Department of Biological Chemistry, University of California, Los Angeles, CA 90095; ^e^ Hematology and Oncology, Department of Medicine, University of California, Los Angeles, CA 90095; ^f^ Division of Pediatric Gastroenterology, Hepatology and Nutrition, University of California, Los Angeles, CA 90095

**Keywords:** cholesterol, lipid transport, small molecule inhibitors

## Abstract

Aster proteins (Aster-A, -B, and -C) are crucial for transporting cholesterol from the plasma membrane (PM) to the endoplasmic reticulum (ER). Asters are expressed in a cell type–specific manner across tissues. Their global disruption leads to varied physiological outcomes given the diverse roles of cholesterol throughout the body. We previously identified sterol analogs, such AI-3d, that inhibit all three Aster proteins. However, their utility is limited by toxicity and off-target effects. Here, we report the development of nonsteroidal Aster inhibitors that are active in cells and in vivo, using binding-guided design to generate compounds with isoform-selective affinities. We found that YKJ-124 is a low-toxicity, Aster-A–preferring inhibitor that elevates PM-accessible cholesterol in primary T cells and potentiates store-operated Ca^2+^ entry in Th17 cells, phenocopying Aster-A deficiency. YKJ-300 and YKJ-305 selectively target Aster-C; cocrystal structures and point mutation studies reveal a Ser477-dependent hydrogen bond (Gly in Aster-A/B) that underlies this specificity. We also explored the in vivo consequences of pharmacologic Aster-C inhibition. YKJ-305 treatment of mice blunted fasting-induced hepatic cholesterol transport and cholesterol ester formation, accompanied by compensatory activation of the SREBP2 pathway. Last, we also identify broader-spectrum inhibitors (YKJ-86) and dual Aster-A/C inhibitors (YKJ-262) that drive PM cholesterol accumulation in fibroblasts and human intestinal enteroids. Together, these chemical probes enable isoform-resolved manipulation of Aster-dependent cholesterol trafficking and provide a foundation for developing Aster-targeted therapies for cholesterol dysregulation.

## Significance

Cholesterol transport by Aster proteins from the plasma membrane (PM) to the endoplasmic reticulum is important for cholesterol homeostasis. We identify nonsteroidal Aster inhibitors with improved specificity and reduced toxicity compared to sterol-based inhibitors. YKJ-124, a low-toxicity Aster-A preferring compound elevates PM-accessible cholesterol and potentiates Ca^2+^ flux in primary T cells. We identify Aster-C-selective inhibitors YKJ-300 and YKJ-305, with YKJ-305 shown to modulate liver cholesterol metabolism in mice. Cocrystal structures and mechanistic studies reveal that ligand specificity for Aster-C is conferred by a Ser477-dependent hydrogen bond. These compounds enable precise dissection of Aster-dependent cholesterol transport in specialized cells and in vivo, and support further efforts to optimize Aster inhibitors as potential therapeutics for diseases associated with cholesterol dysregulation.

Cholesterol plays an important role in membrane structure, fluidity, and function. Maintaining proper cholesterol homeostasis is essential for numerous cellular processes, including signal transduction, membrane trafficking, and cell growth. Dysregulation of cholesterol homeostasis has been implicated in a wide range of human disorders, including cardiovascular disease, neurodegenerative disease, and cancer. Although most cellular cholesterol resides in the plasma membrane (PM), only a fraction is “accessible,” that is, active and available for binding by cholesterol-sensing proteins, toxins, or transport machinery, whereas the remainder is sequestered through interactions with phospholipids and other membrane components (1, 2). The transport of cholesterol between different organelles is tightly regulated by a complex network of proteins. Among these, the Asters (Aster-A, -B, and -C, also known as Gramd1a, -b, -c) have emerged as key mediators of accessible cholesterol transport from the PM to the ER in mammals (3). These proteins contain a conserved START domain that binds cholesterol, and an N-terminal GRAM domain that mediates the recruitment of Asters to cholesterol-enriched PM.

Asters are expressed in a tissue- and cell-type-specific manner. Previous studies in genetically modified mice have highlighted the critical functions of Aster proteins in different tissues and demonstrated the diverse physiological consequences of Aster inhibition (4–7). Loss of Aster-B function disrupts PM-to-ER cholesterol transport in the ovary, leading to hypoplasia and impaired estradiol synthesis and predisposing female mice to diet-induced obesity (7). Ablation of Aster-A and Aster-C in the liver impairs hepatic cholesterol transport, resulting in decreased cholesterol esterification and bile acid synthesis, elevated plasma cholesterol, and peripheral cholesterol accumulation (5). Aster-B and -C deficiency in the intestine impairs cholesterol absorption (6), while Aster-A loss in T cells reduces triglyceride uptake (8). Additionally, endothelial cell-specific loss of Aster-A causes inflammation in the lung and aorta (9).

Aster proteins have recently been recognized as potential targets for small-molecule inhibitors of cellular cholesterol trafficking and metabolism. One study identified the autophagy inhibitor Autogramin-2 as an inhibitor of Aster-A (10). Our previous work identified AI-3d, a sterol-based analog, as a pan inhibitor of all three Aster proteins (11). Another study explored a sterol-inspired compound library and identified Astercin as an inhibitor of Aster-C in liposomes (12). However, sterol-derived or sterol-inspired scaffolds often provide limited flexibility for structural modification, making further optimization difficult. This limited chemical modifiability, along with the potential for off-target toxicity, highlights the need for alternative inhibitory strategies.

Here, we report the design, synthesis, and characterization of a library of nonsteroidal Aster inhibitors. Employing a structure-based approach guided by binding affinity data, we identified compounds exhibiting both selective targeting of individual Aster proteins and potent pan-Aster inhibitory activity. We demonstrate the efficacy of these compounds in modulating cholesterol transport in cells and organoids and evaluate their toxicity profiles. YKJ-124 preferentially inhibits Aster-A, is markedly less toxic than the sterol analog AI-3d, increases PM–accessible cholesterol in primary T cells, and potentiates TCR-triggered Ca^2+^ influx in Th17 cells, phenocopying Aster-A deficiency. YKJ-300 and YKJ-305 preferentially inhibit Aster-C, with cocrystal structures revealing a Ser477-dependent hydrogen bond that underlies selectivity. YKJ-305 reduces fasting-induced hepatic cholesterol transport and cholesterol ester formation, elevating hepatic SREBP2 pathway gene expression in liver-specific Aster-A knockout mice. In addition, YKJ-86 broadly inhibits all three Asters to drive PM cholesterol accumulation in fibroblast cells and human intestinal enteroids, whereas YKJ-262 preferentially inhibits Aster-A and -C and similarly increases accessible cholesterol at the PM in fibroblast cells.

## Results

### Design and Synthesis of Nonsteroidal Aster Inhibitors.

**Fig. 1.**
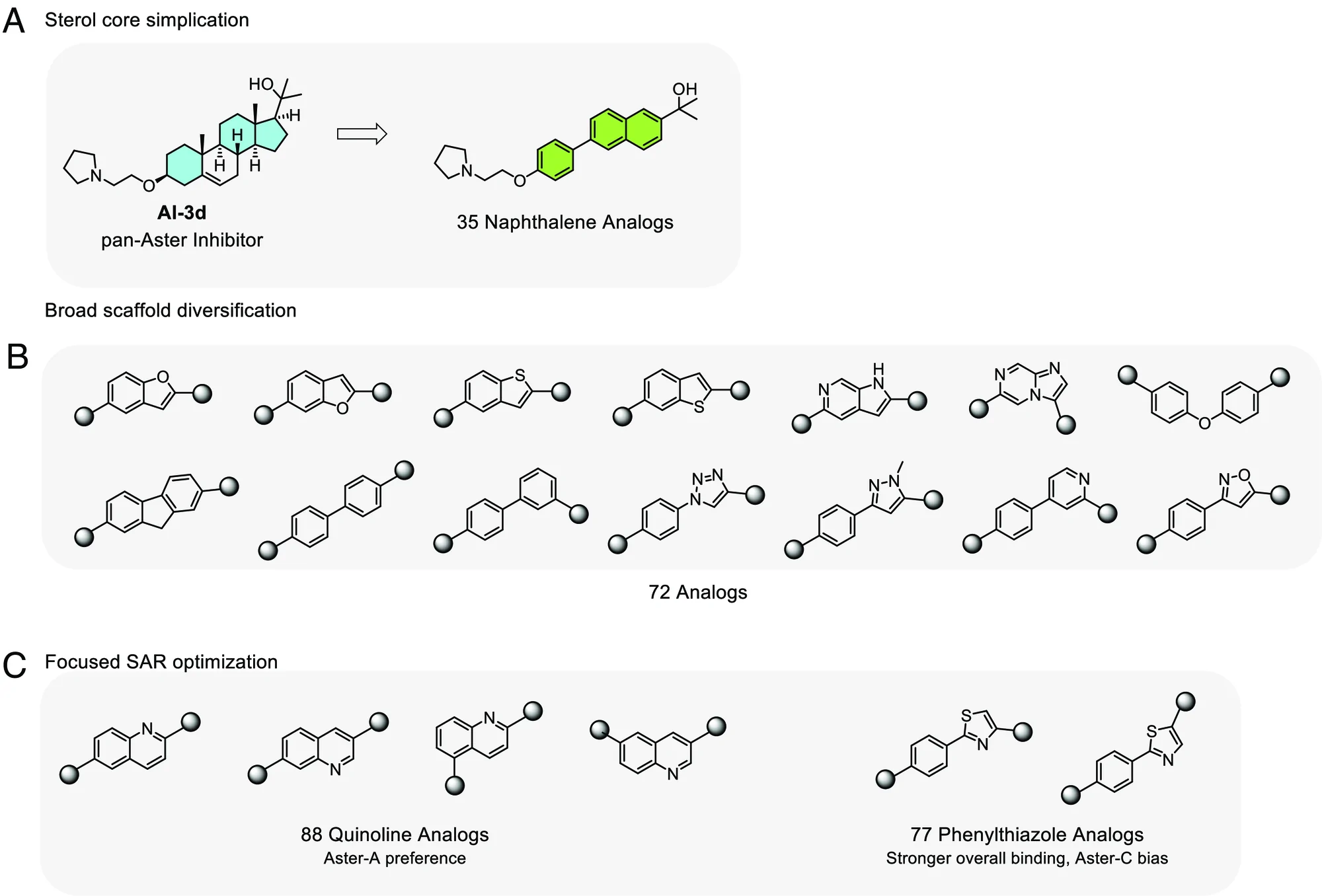
Design and synthesis of nonsteroidal Aster inhibitors. (*A*) Simplification of the Aster inhibitor sterol core. The sterol core of AI-3d was replaced with a phenyl–naphthalene framework as a nonsteroidal surrogate, generating an initial set of 35 naphthalene analogs. (*B*) Broad scaffold diversification. The naphthalene core was replaced with diverse aromatic frameworks to explore structure–activity relationships across Aster-A, Aster-B, and Aster-C (72 analogs). (*C*) Focused SAR optimization. Quinoline- and phenylthiazole-based libraries were constructed based on emerging activity trends. Quinoline analogs showed relative preference for Aster-A, whereas phenylthiazole analogs displayed stronger overall binding with a bias toward Aster-C. In total, 272 nonsteroidal small-molecule analogs were synthesized for systematic evaluation.

AI-3d is a useful inhibitor of Aster activity in cells but has important limitations. It exhibits toxicity at higher concentrations and inhibits Aster-A, Aster-B, and Aster-C without selectivity. Moreover, its rigid steroidal scaffold restricts structural modification, limiting systematic structure–activity exploration. To address these limitations, we sought to develop nonsteroidal Aster inhibitors that would allow improved selectivity and greater structural flexibility for scaffold optimization. Because the sterol core primarily contributes hydrophobic interactions within the binding pocket, we began with a simplified hydrophobic framework. Replacement of the C/D rings of cholesterol with a naphthalene unit and the A ring with a phenyl group generated a phenyl–naphthalene scaffold as a nonsteroidal surrogate of the steroid core (Fig. 1*A*). This scaffold exhibited measurable, albeit modest, binding activity, supporting further optimization.

Broader diversification of the aromatic core was then undertaken to interrogate structure–activity relationships (Fig. 1*B*). Iterative analysis revealed distinct activity patterns among scaffold classes. Quinoline-based analogs showed relative preference for Aster-A, whereas phenyl–thiazole analogs displayed stronger overall binding across the family, with certain structures showing a bias toward Aster-C. Based on these trends, focused libraries were constructed around the quinoline and phenyl–thiazole scaffolds (Fig. 1*C*). In total, 272 nonsteroidal small-molecule analogs were synthesized to enable systematic evaluation of activity across the Aster family (full structures are provided in *SI Appendix*).

### Binding Landscape of Nonsteroidal Aster Inhibitors.

**Fig. 2.**
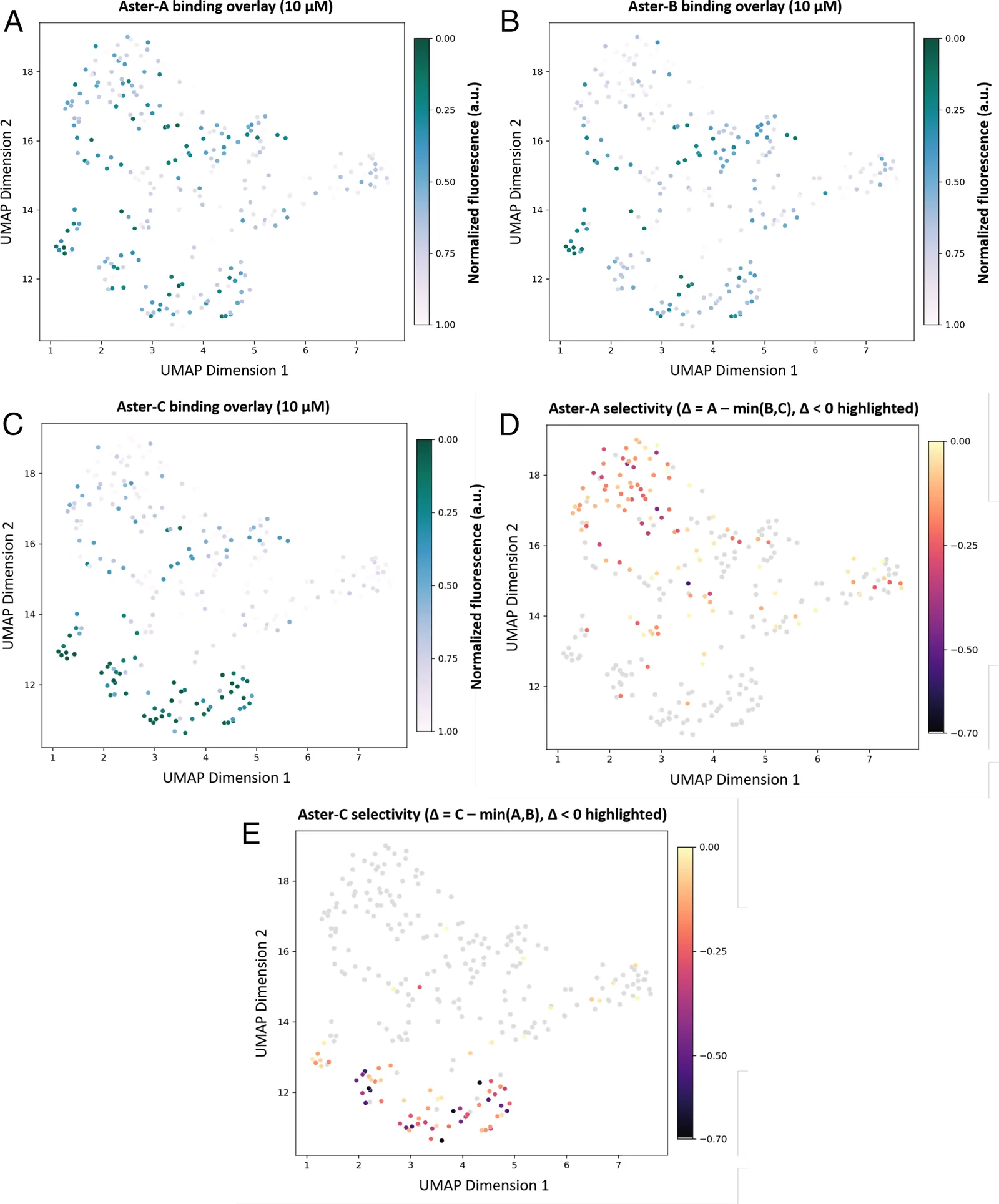
Binding landscape of nonsteroidal Aster inhibitors. (*A*–*C*) UMAP projection of the 272-compound library generated from Morgan fingerprints. Normalized fluorescence values from the NBD-cholesterol competition assay at 10 μM are overlaid for Aster-A (*A*), Aster-B (*B*), and Aster-C (*C*). Lower normalized fluorescence indicates stronger inhibition. Each point represents one compound. (*D* and *E*) Relative preference mapped onto the same UMAP projection using selectivity indices calculated from normalized fluorescence values [ΔA = A−min(B,C) in *D*; ΔC = C−min(A,B) in *E*]. More negative values indicate greater relative preference for the indicated Aster protein.

To evaluate activity across the series, all 272 compounds were tested at 10 μM using an NBD-cholesterol competition assay previously established in our laboratory (11). In this fluorescence-based assay, ligand binding is measured by displacement of the fluorescent probe, resulting in reduced fluorescence. Compounds that decreased normalized fluorescence to ≤0.6 (≥40% inhibition) were classified as active. To visualize activity distribution, the library was projected into two-dimensional chemical space using UMAP calculated from Morgan fingerprints. The UMAP coordinates reflect structural similarity and are used here solely to visualize activity patterns across the series. Inhibition values were overlaid for Aster-A, Aster-B, and Aster-C (Fig. 2 *A*–*C*), where lower normalized fluorescence corresponds to stronger inhibition. Each point represents a single compound.

Of the 272 analogs, 154 (56%) were active against at least one Aster protein. By target, 117 compounds inhibited Aster-A, 92 inhibited Aster-B, and 99 inhibited Aster-C. Aster-B showed fewer inhibitors than Aster-A and Aster-C. The most potent Aster-B inhibitors largely overlapped with compounds active against the other Aster proteins, and no strongly Aster-B–selective inhibitors were identified under these conditions. To assess relative preference across the three proteins, selectivity indices were calculated from normalized fluorescence values as ΔA = A−min(B,C) and ΔC = C−min(A,B), where more negative values indicate relative preference. Mapping these values onto the same UMAP projection (Fig. 2 *D* and *E*) showed that preferential activity was not randomly distributed. Relative Aster-A preference was more frequently associated with quinoline-based analogs, whereas relative Aster-C preference was enriched within a defined region corresponding to the phenyl–thiazole scaffold cluster (*SI Appendix*, Fig. S1). These analyses guided the selection of compounds for subsequent selectivity and structural studies.

### Off-Target Testing of Nonsteroidal Analogs.

To assess the potential off-target effects of the newly synthesized compounds, we focused on two key cholesterol-binding pathways: NPC1/2 and the Insig-SCAP pathway (*SI Appendix*, Fig. S2). NPC1/2 are lysosomal cholesterol transporters, and mutations in these proteins lead to cholesterol accumulation in lysosomes, detectable by filipin staining (13). To evaluate whether the candidate nonsteroidal analogs inhibited NPC1/2 function, CHO-K1 cells were treated with the compounds at a concentration of 10 μM for 16 h in cholesterol-containing medium. Filipin staining was performed using the potent NPC1 inhibitor U18666A as a positive control. The results showed that 26 compounds caused minimal lysosomal cholesterol accumulation as revealed by filipin staining, indicating minimal off-target effects on NPC1/2 (*SI Appendix*, Fig. S3).

We also investigated off-target effects on the Insig-SCAP pathway using SREBP2 cleavage inhibition as a functional readout. CHO cells were depleted of cholesterol for 16 h to induce SREBP2 cleavage. The cells were then treated with the nonsteroidal YKJ compounds for 2 h, with 25-hydroxycholesterol (25-HC) serving as a positive control (14). The analysis revealed that 42 YKJ compounds, including YKJ-86, 262, 300, and 305, did not inhibit SREBP2 cleavage, demonstrating that, unlike many oxysterols, these compounds do not interfere with the Insig-SCAP pathway (*SI Appendix*, Fig. S4). After these two rounds of off-target testing, we were left with 10 YKJ compounds that inhibited neither NPC1/2 nor the Insig-SCAP pathway.

### Cellular Activity of Nonsteroidal Aster Inhibitors.

We further examined using a cell-based cholesterol transport assay to evaluate the candidate compound’s potential as an Aster inhibitor. Aster proteins play a critical role in transporting cholesterol from PM to ER, a process that suppresses the cleavage and activation of SREBP2. To assess the impact of the YKJ compounds on Aster function, CHO-K1 cells were pretreated with the YKJ compounds for 4 h before being exposed to cyclodextrin-cholesterol for another 2 h. Cyclodextrin-cholesterol normally suppresses SREBP2 cleavage through Aster-dependent cholesterol transport to ER (11). These assays revealed that 6 out of 20 YKJ compounds, including YKJ-86, 124, and 262, effectively blocked the ability of exogenous cholesterol to suppress SREBP2 cleavage (*SI Appendix*, Fig. S5). Importantly, the cellular activities of these Aster inhibitors align with the in vivo consequences of Aster genetic knockouts in mice (3–7).

### YKJ-124 Specifically Inhibits Aster-A.

**Fig. 3.**
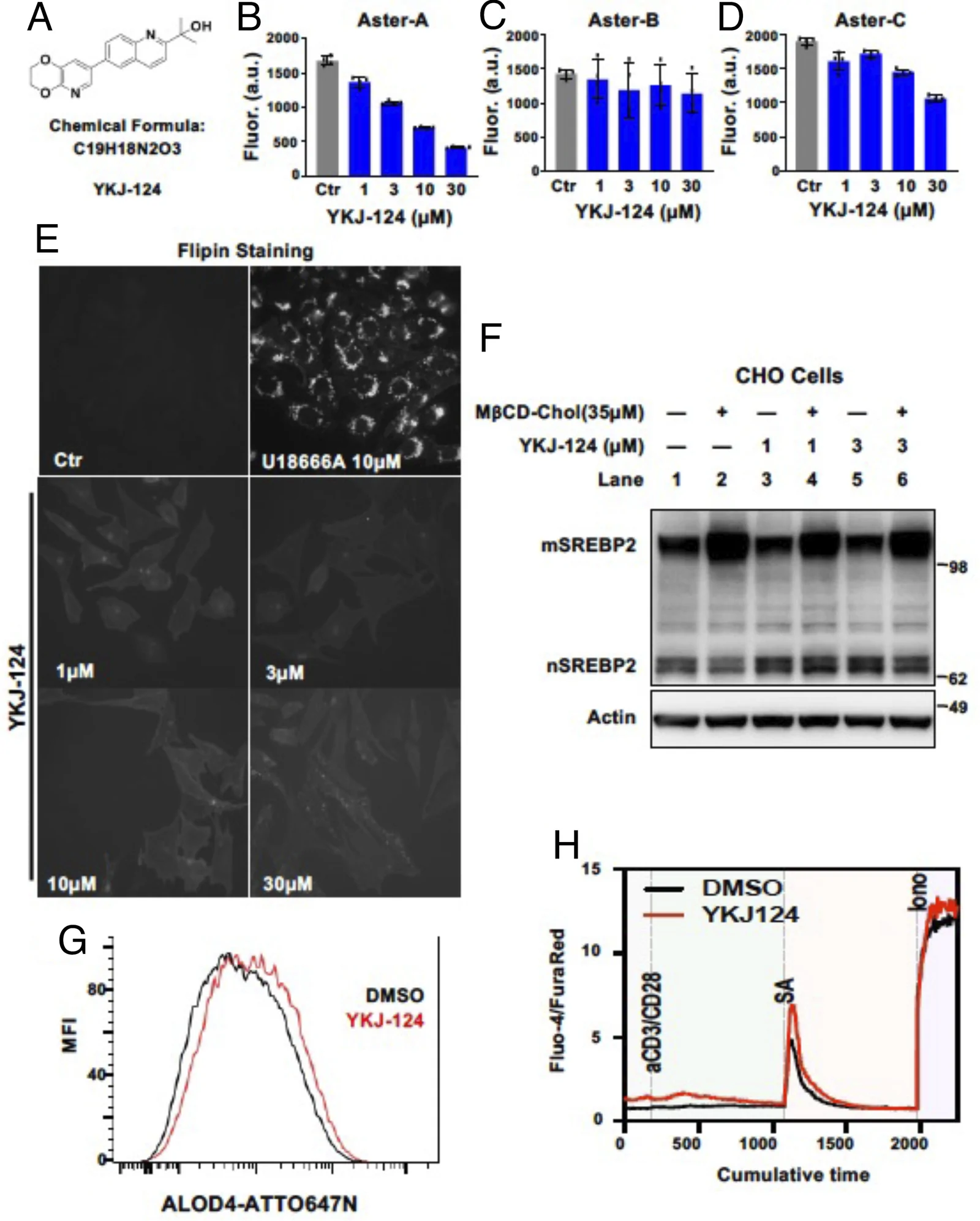
YKJ-124 specifically inhibits Aster-A. (*A*) Structure of YKJ-124. (*B*–*D*) Competition assay in which purified Aster domains of Aster-A (*B*), Aster-B (*C*), and Aster-C (*D*) were incubated with 22-NBD-cholesterol in the presence of vehicle or YKJ-124 at indicated concentrations. Data bars represent means ± SD. (*E*) CHO-K1 cells were incubated in medium containing 5% FBS, U18666A, and YKJ-124 at the indicated concentration or vehicle for 16 h at 37 °C. Cholesterol was visualized by filipin staining. (*F*) CHO-K1WT cells were depleted of sterols by incubating in medium containing 1% LPDS, simvastatin (5 μM), and mevalonate (10 μM) for 16 h at 37 °C. Cells were switched to the same medium with vehicle or YKJ-124 (1 and 3 μM) for 6 h. Cells were then treated with same medium with or without MβCD-cholesterol for another 2 h. Immunoblots were performed with anti-SREBP2. mSREBP2: membrane/precursor form SREBP2; nSREBP2: nuclear/mature forms SREBP2. (*G*) Flow cytometry plots of ALOD4 staining in primary mouse Th17 cells treated with DMSO or YKJ-124 compound (3 μM) for 6 h. MFI, mean fluorescence intensity. (*H*) Ca^2+^ flux trace in primary mouse Th17 cells treated with DMSO or YKJ-124 (3 μM) for 6 h.

The binding data from Fig. 2 identified YKJ-124 as a selective inhibitor of Aster-A (Fig. 3*A*). To further investigate its specificity, we performed dose–response curves assessing the binding affinity of YKJ-124 for Aster-A, -B, and -C. YKJ-124 bound preferentially to Aster-A over Aster-B and Aster-C (Fig. 3 *B*–*D*). Filipin staining showed that YKJ-124 did not induce cholesterol accumulation in lysosomes, even at a concentration of 30 μM, in contrast to the pronounced accumulation caused by the NPC1 inhibitor U18666A (Fig. 3*D*). This suggests that YKJ-124 selectively targets Aster-A without affecting other cholesterol transport pathways. Treatment of CHO cells with YKJ-124 led to the inhibition of Aster-mediated cholesterol transport, which in turn attenuated the suppression of nuclear SREBP2 by cyclodextrin-cholesterol (Fig. 3*F* and *SI Appendix*, Fig. S6*A*). We further found that YKJ-124 exhibited lower toxicity compared to the sterol-based inhibitor AI-3d (*SI Appendix*, Fig. S6*B*).

Aster-A is highly expressed in T lymphocytes where it regulates PM accessible cholesterol levels and thereby modulates T cell receptor activation-dependent and -independent Ca^2+^ flux (8). AI-3d exhibited notable toxicity toward primary T cells, precluding our prior use of small molecule Aster inhibitors in probing immune functions. To test if YKJ-124 could modulate Ca^2+^ flux in T cells, we incubated primary mouse T helper-17 cells with 10 μM YKJ-124 for 6 h. Consistent with the previously established function of Aster proteins in T cells (8), inhibition of Aster-A by YKJ-124 increased PM accessible cholesterol, as shown by ALOD4 staining (Fig. 3*G*). ALOD4 is a recombinant cholesterol-binding probe that selectively binds the accessible pool of cholesterol at the PM, allowing visualization of changes in PM cholesterol availability rather than total cellular cholesterol (15, 16). We also examined Ca^2+^ flux during T cell activation following YKJ-124 incubation. Similar to Aster-A KO T cells, YKJ-124-treated T cells showed elevated store-operated Ca^2+^ entry upon T cell receptor crosslinking and increased maximum Ca^2+^ influx induced by Ionomycin, compared to DMSO treated controls (Fig. 3*H*). Thus, we demonstrate the application of a low-toxicity, nonsteroidal small molecule to manipulate Aster-A function in specialized primary cells.

### Structure of the Aster-C Sterol-Binding Domain in Complex with YKJ-300 and YKJ-305.

**Fig. 4.**
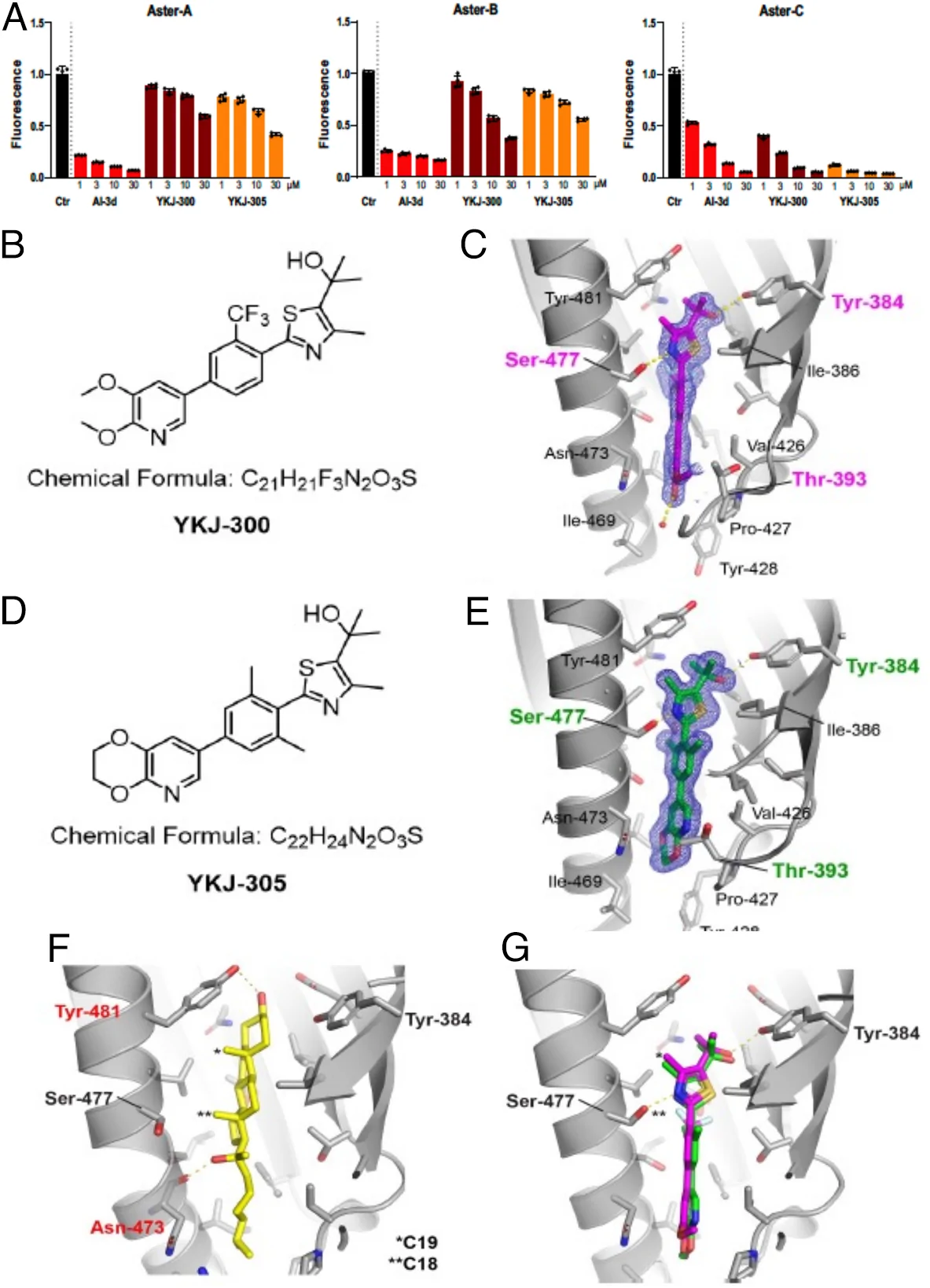
Structural basis of Aster-C recognition by YKJ-300 and YKJ-305. (*A*) Competition assay in which purified Aster domains of Aster-A (*A*), Aster-B (*B*), and Aster-C (*C*) were incubated with 22-NBD-cholesterol in the presence of vehicle, AI-3d, YKJ-300, or YKJ-305 at indicated concentrations. Data bars represent means ± SD. (*B*) Chemical formula of YKJ-300. (*C*) Close-up view of YKJ-300 in the Aster-C binding pocket. The 2F_0_-F_c_ electron density for the ligand is contoured at 1.0 sigma (blue mesh). Pocket residues are shown as sticks. Highlighted amino acids include Thr393, which forms part of the loop that closes over the ligand, and Ser477 and Tyr384, which form hydrogen bonds with YKJ-300. (*D*) Chemical formula of YKJ-305. (*E*) Close-up view of YKJ-305 bound to Aster-C domain. The 2F_0_-F_c_ electron density for the ligand is contoured at 1.0 sigma (blue mesh). Pocket residues are shown as sticks. Key interactions include Thr393, which forms part of the loop that closes over the ligand, and Ser477 and Tyr384, which form hydrogen bonds with YKJ-305. (*F*) Close-up view of the Aster Inhibitor 1l (AI-1l) in the Aster-C binding pocket (PDB ID: 7AZN). Electron density is contoured at 1.0 sigma (blue mesh). Residues labeled in red are essential for AI-1l interaction (Tyr481 and Asn473). Residues in black are conserved for YKJ-300/305 interaction. Axial methyl groups of AI-1l are indicated by asterisks. (*G*) Structural overlay of the Aster-C domain in complex with YKJ-300 (magenta) and YKJ-305 (green). The thiazole methyl (one asterisk) and nitrogen (two asterisks) moieties mimic sterol-derived interactions. Specifically, the thiazole nitrogen forms a hydrogen bond with Ser477, inducing a slight conformational shift in the helix.

A separate group of compounds, including YKJ-300 and 305, showed higher specificity for Aster-C than Aster-A or Aster-B (Fig. 4*A*). To understand the structural basis of this selectivity, we determined the crystal structures of the Aster domain of Aster-C in complex with YKJ-300 at 2.00 Å resolution, and YKJ-305, at 1.75 Å resolution. The overall structure of the Aster-C domain exhibits the canonical curved seven stranded-beta-sheet that forms a cavity, enclosed by a long carboxyl-terminal helix and two shorter helices. As observed in other Aster domain structures, additional electron density adjacent to the isopropyl hydroxyl group of the ligands reveals extra space within the cavity. This extra space accommodates an ethanol and a glycerol molecule, likely from the copurification and cryoprotectant, respectively (*SI Appendix*, Fig. S7 *A* and *B*). Clear electron density within the binding pocket unambiguously defines the position and orientation of both ligands which is dictated primarily by inward-facing residues located on the C-terminal helix and the beta-sheets of the Aster domain (Fig. 4 *C* and *E*).

In contrast to sterol-based molecules previously investigated, YKJ-300 and YKJ-305 possess a nonsteroidal scaffold that confers a largely planar molecular framework (Fig. 4 *B* and *D*). The pyridine, phenyl, and thiazole rings are individually planar but are connected by single C–C bonds that allow rotational flexibility between them. This flexibility permits multiple conformations of the ligand and makes the orientation of the compounds within the Aster-C binding pocket difficult to predict. Consequently, structural determination was required to establish their precise binding mode, despite their overall size and length being comparable to cholesterol.

The binding position and orientation of both ligands are similar but not identical (compare Fig. 4 *C* and *E*), as YKJ-300 and YKJ-305 share a common thiazole ring substituted with an isopropyl group. These groups are critical for anchoring the ligands via interaction with two key residues within the Aster-C cavity: Tyr384 and Ser477. Specifically, Tyr-384 forms a hydrogen bond with the hydroxyl group of the isopropyl moiety, while Ser477 forms a hydrogen bond with the thiazole nitrogen. Together, these interactions constrain the thiazole ring into an almost perpendicular orientation relative to the rest of the molecule, which remains planar. The thiazole conformation is further restricted by steric effects. In YKJ-300, the -CF_3_ group introduces a steric constraint through interaction with Thr434 (*SI Appendix*, Fig. S7*C*). In YKJ-305 the two methyl substituents of the thiazole ring create a “steric-clamp” effect mediated by van der Waals interactions with hydrophobic amino acids, Phe432/Leu456 and Ile386/Leu388, on opposite sides of the pocket (*SI Appendix*, Fig. S7 *D* and *E*). Additionally, the phenyl and pyridine rings of both compounds are stabilized through hydrophobic interactions within the cavity (Fig. 4 *C* and *E*).

The affinity of sterol-based molecules for Aster proteins is remarkably high (3). The molecular bases for this strong interaction were revealed in the structures of Aster-A domain in complex with 25-hydroxy-cholesterol and Aster-C domain in complex with a sterol-derived compound, AI-1l, which is chemically similar to cholesterol (11). These structures demonstrated that ligand positioning and orientation within the cavities are primarily driven by hydrophobic forces and a key hydrogen bond between the C3-hydroxyl group and Tyr481 in Aster C (Fig. 4*F*) (or Tyr524 in Aster A, *SI Appendix*, Fig. S7*F*). Specifically, the hydrophobic amino acids forming the binding cavity interact with the sterol rings and the two bulky axial methyl groups (C18 and C19) which largely contribute to the precise seating of the compounds within the hydrophobic pocket (Fig. 4*F* and *SI Appendix*, Fig. S7*F*). These amino acids are conserved in Aster-A, -B, and -C (1). Notably, in the case of ligand AI-1l, there is an additional hydrogen bond with Asn473 (Fig. 4*F*).

These specific interactions are absent in the nonsteroidal compounds. Instead, the thiazole group in YKJ-300/305 adopts a nearly 90-degree twist relative to the B ring of AI-1l and 25-hydroxy-cholesterol. This conformation shift places the thiazole methyl group in a position analogous to the axial C19 methyl of the sterol molecules, while orienting the thiazole nitrogen to form a hydrogen bond with the hydroxyl group of Ser477 (Fig. 4*G*). Thus, the thiazole methyl contributes to the hydrophobic interaction of the nonsteroidal compounds in a manner similar to the C19 methyl in the sterol molecules. Furthermore, the interaction typically involving the C18 axial methyl in the sterol-derived compounds is functionally mirrored by the hydrogen bond between the thiazole nitrogen and Ser477. Interestingly, this hydrogen bond attracts Ser477 to directly engage with the nonsteroidal compounds causing a slight conformational change in the helix (*SI Appendix*, Fig. S7*G*).

Interestingly, binding assays indicated that YKJ-305 binds more strongly to the Aster-C domain than YKJ-300 (Fig. 4*A*). Structural comparison suggested that Thr434, located within the binding cavity, lies in close proximity to the bulky trifluoromethyl group (-CF_3_) of YKJ-300 (*SI Appendix*, Fig. S7*C*). This steric constraint likely reduces binding affinity relative to YKJ-305, which contains a smaller methyl group (-CH_3_) that fits more favorably within this confined space (*SI Appendix*, Fig. S7*D*). The additional thiazole methyl in YKJ-305 potentially contributes to the enhanced affinity by making additional hydrophobic interactions with surrounding residues such as Ile386 and Leu388 (yellow arrow, *SI Appendix*, Fig. S7*E*). Moreover, the closed ring structure in YKJ-305, which is linked to the pyridine moiety, may restrict conformational flexibility and entropically favor the bound state.

Notably, both structures reveal differences in the position of the loop that closes over the pyridine ring of the ligands (Fig. 4 *C* and *E* and *SI Appendix*, Fig. S7*E*). Consistent with previous structures of the Aster domains, this loop exhibits elevated B-factors and adopts multiple conformations, indicating inherent flexibility. The degree to which this loop accommodates distinct ligands is likely to influence binding kinetics and, consequently, overall affinity and specificity.

### Aster-C S477 (Aster-A G520) Determines Binding Specificity for YKJ-300 and YKJ-305.

**Fig. 5.**
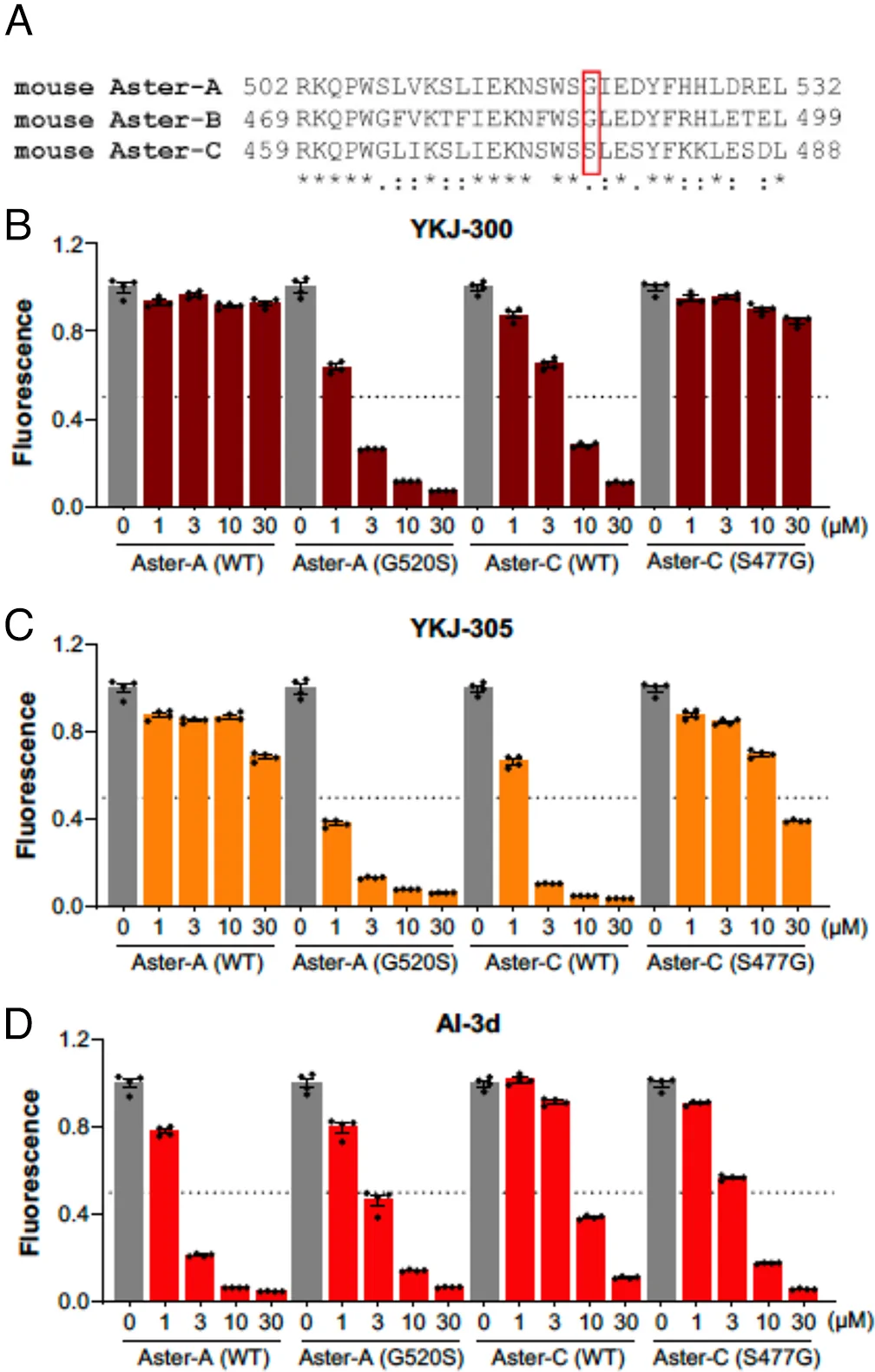
Aster-C S477 determines binding specificity for YKJ-300 and YKJ-305. (*A*) Multiple sequence alignment of mouse Aster-A, -B, and -C reveals a glycine in Aster-A and -B at the position corresponding to Ser477 in Aster-C. (*B*–*D*) Competition assay in which purified Aster domains of Aster-A (WT), Aster-A (G520S), Aster-C (WT), and Aster-C (S477G) were incubated with 22-NBD-cholesterol in the presence of vehicle, YKJ-300, YKJ-305, or AI-3d at indicated concentrations. Data bars represent means ± SD.

To investigate the molecular basis for selective binding of YKJ-300 and YKJ-305 to Aster-C, we compared these structures with that of Aster-A bound to 25-hydroxy-cholesterol. It is clear that Ser477 in Aster-C plays a key role in specificity. The equivalent residue is a glycine in Aster-A and -B which could not make an equivalent hydrogen bond with the thiazole ring (Fig. 5*A* and *SI Appendix*, Fig. S7*F*). Importantly, Ser477 is also present in human Aster-C but is a glycine in human Aster-A and B. The role of Ser477 on YKJ-300 and YKJ-305 selectivity for Aster-C was interrogated by site-directed mutagenesis and binding assays. Ser477 in Aster-C was mutated to a glycine (S477G). Conversely, Gly520 in Aster-A was mutated to serine (G520S). Binding assays clearly demonstrated that this single residue was a critical determinant of selective ligand interaction. Specifically, the S477G mutation in Aster-C markedly reduced affinity, whereas the reciprocal G520S in Aster-A enhanced ligand binding (Fig. 5 *B* and *C*). Importantly, these mutations did not affect binding to the pan-Aster sterol analog AI-3d, indicating that Ser477 specifically enables YKJ-300/305 binding through the hydrogen-bond interaction observed in the cocrystal structures (Fig. 5*D*). Together, these results confirm that the presence of serine at this position is essential for the specific recognition of YKJ-300 and YKJ-305 by the Aster-C domain.

### YKJ-305 Blocks Cholesterol Transfer by Aster-C In Vivo.

**Fig. 6.**
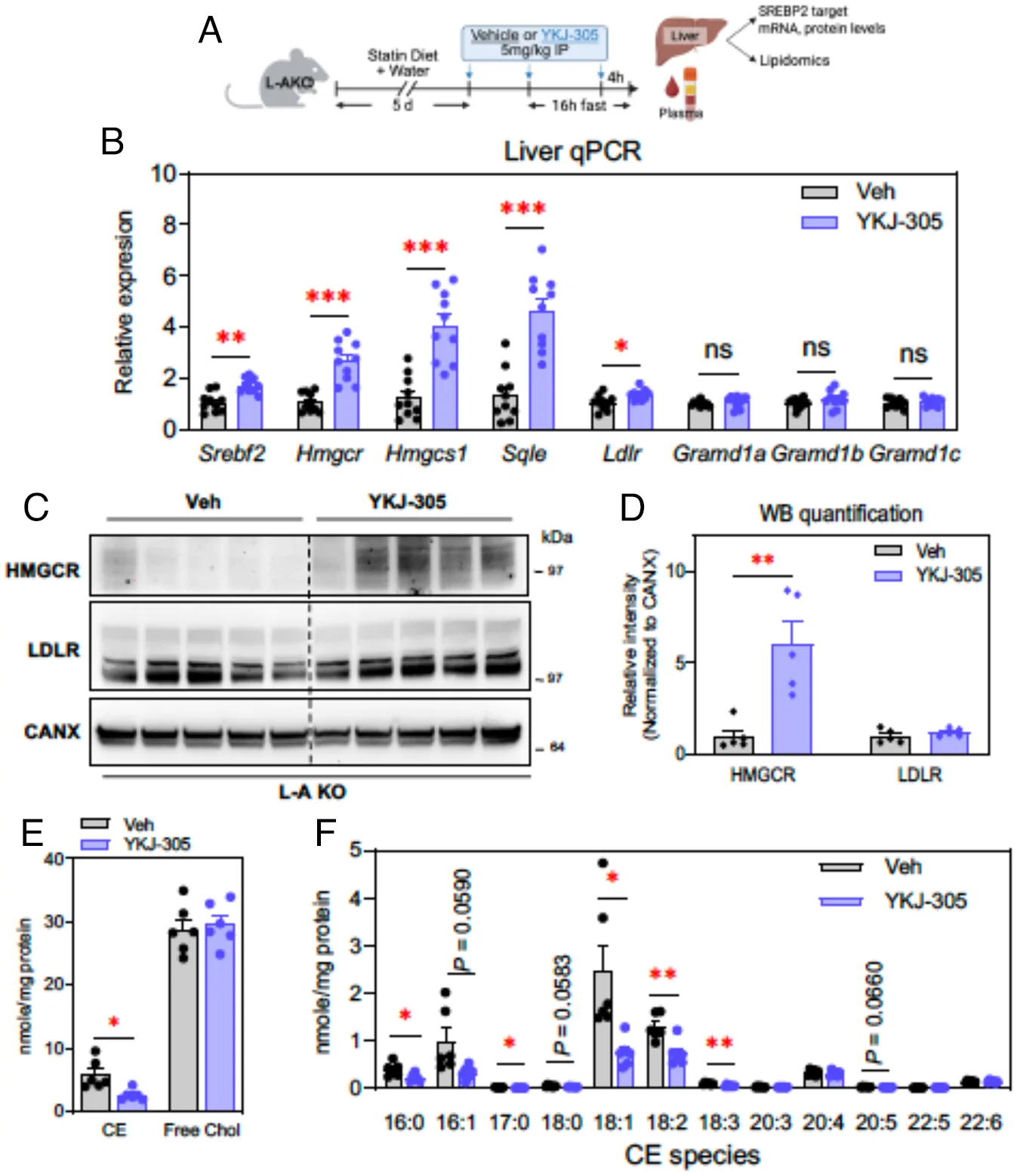
YKJ-305 blocks cholesterol transfer by Aster-C in vivo. (*A*) Experiment schematic of in vivo YKJ-305 treatment regimen and sample collection. (*B*) Hepatic mRNA expression of indicated genes from vehicle or YKJ-305-administered mice (n = 10/9). (*C*) Immunoblot analysis of SREBP2 pathway proteins in the livers of mice administered with vehicle or YKJ-305. Calnexin (CANX) was used as a loading control. (*D*) Immunoblot quantification from *C*. Normalized to Calnexin. (*E* and *F*) Total liver CEs (*E*) and individual hepatic CE species (*F*) from A (n = 6/6).

Since most immortalized cell lines lack Aster-C expression, we tested whether YKJ-305 inhibits Aster-C function in vivo using liver-specific Aster-A knockout mice, in which hepatic PM-to-ER cholesterol transport is expected to rely predominantly on Aster-C (Fig. 6*A*). Aster-A liver–KO mice were intraperitoneally injected with YKJ-305 or vehicle daily. This regimen was well tolerated, with no change in body weight and hepatic Aster-C expression was comparable between YKJ-305-treated mice and vehicle treated controls (*SI Appendix*, Fig. S8 *A* and *B*). We observed no appreciable changes in plasma lipid levels following YKJ-305 treatment (*SI Appendix*, Fig. S8 *C*–*E*). Notably, however, YKJ-305 blunted fasting-induced PM-to-ER cholesterol transfer, as indicated by increased expression of SREBP2 pathway genes by qPCR (Fig. 6*B*). Consistent with SREBP2 activation, expression of the rate-limiting cholesterol biosynthetic enzyme HMGCR was also elevated after YKJ-305 treatment (Fig. 6 *C* and *D*). Lipidomic analysis of liver tissue revealed that YKJ-305 treatment reduced hepatic cholesteryl ester (CE), whereas free cholesterol was largely unchanged (Fig. 6*E*). Lipid profiling further showed that YKJ-305 decreased multiple CE species, including 16:0, 17:0, 18:1, 18:2, and 18:3 species (Fig. 6*F*). In contrast, diacylglycerol (DG) levels increased, whereas most other lipid classes were largely unchanged (*SI Appendix*, Fig. S8*F*). These findings are consistent with our prior observations in liver-specific Aster-A/C knockout mice and strongly support the in vivo efficacy of YKJ-305 as an Aster-C inhibitor. The results establish YKJ-305 as a valuable chemical probe for interrogating Aster-C biology in vivo.

### Pan-Aster Inhibition by YKJ-86 and Selective Aster-A/C Targeting by YKJ-262.

**Fig. 7.**
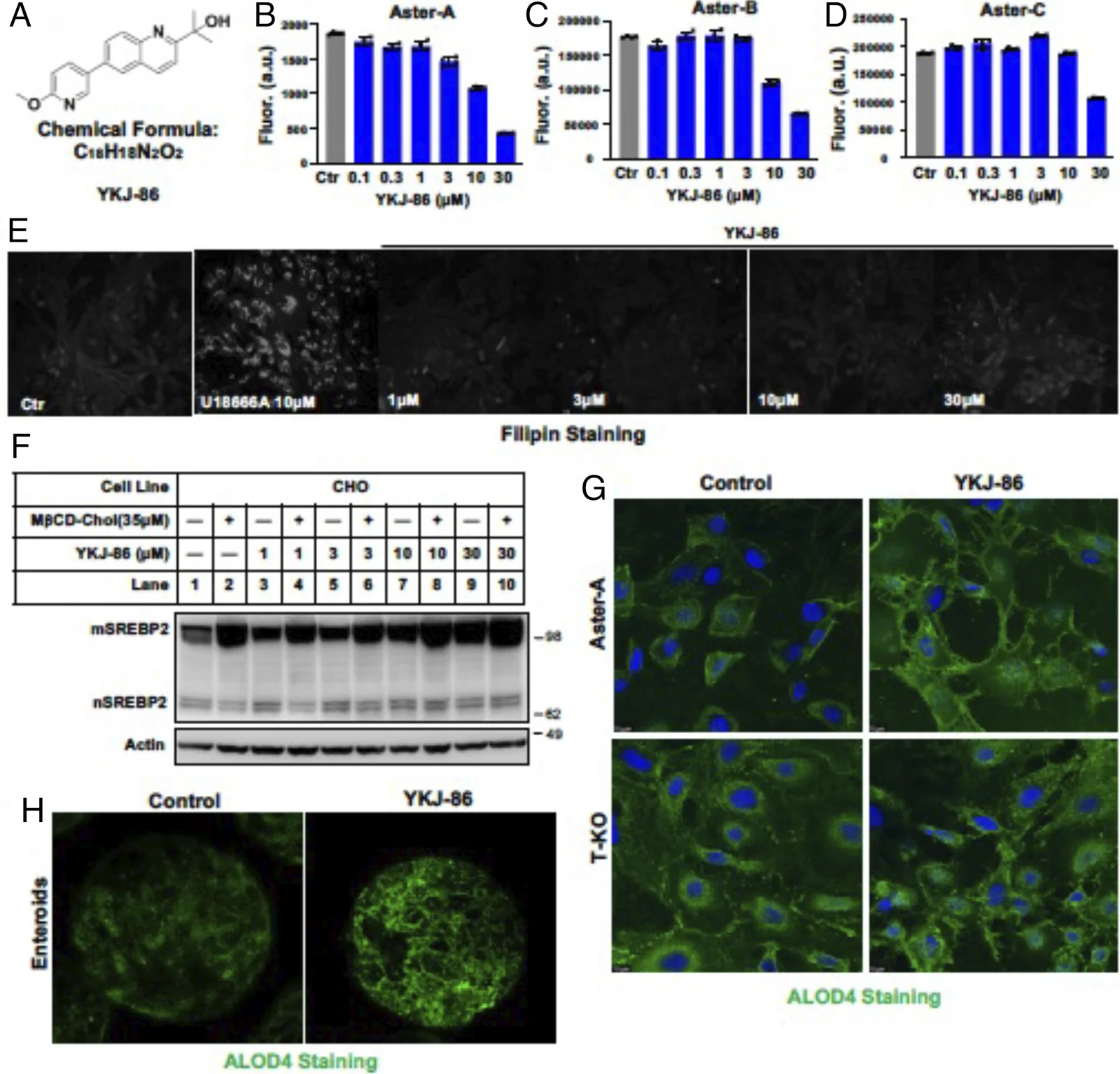
YKJ-86 targets all 3 Asters. (*A*) Structures of YKJ-86. (*B*–*D*) Competition assay in which purified Aster domains of Aster-A (*B*), Aster-B (*C*), and Aster-C (*D*) were incubated with 22-NBD-cholesterol in the presence of vehicle or YKJ-86 at indicated concentrations. Data bars represent means ± SD. (*E*) CHO-K1 cells were incubated in medium containing 5% FBS, U1866A, and YKJ-86 at the indicated concentration or vehicle for 16 h at 37 °C. Cholesterol was visualized by filipin staining. (*F*) CHO-K1WT cells were depleted of sterols by incubating in medium containing 1% LPDS, simvastatin (5 μM), and mevalonate (10 μM) for 16 h at 37 °C. Cells were switched to the same medium with vehicle or YKJ-86 at the indicated concentration for 6 h. Cells were then treated with same medium with or without MβCD-cholesterol for another 2 h. Immunoblots were performed with anti-SREBP2. mSREBP2: membrane/precursor form SREBP2; nSREBP2: nuclear/mature forms SREBP2. (*G*) ALOD4 staining of Aster-null fibroblasts ± Aster-A overexpression treated with vehicle or YKJ-86, to assess PM accessible cholesterol. Images were acquired 1 h after MβCD–cholesterol loading. (*H*) ALOD4 imaging of human intestinal enteroids treated with vehicle or YKJ-86, with images acquired 1 h after MβCD–cholesterol loading.

Our first three rounds of screening identified YKJ-86 as a potent inhibitor of Aster-A, -B, and -C (Fig. 7*A*). To further characterize its efficacy, we conducted a detailed analysis of its activity across a range of concentrations (Fig. 7 *B*–*D*). Treatment of CHO-K1 cells with YKJ-86 for 16 h did not cause cholesterol accumulation in lysosomes, as confirmed by filipin staining (Fig. 7*E*). Additionally, YKJ-86 effectively blocked Aster-mediated cholesterol transport in a concentration-dependent manner and completely inhibited cholesterol transport at 10 μM (Fig. 7*E*, lanes 7 and 8 and *SI Appendix*, Fig. S9*A*).

Loss of Aster expression results in a marked accumulation of free cholesterol in the PM of fibroblast cells, as demonstrated by ALOD4 staining (Fig. 7 *G*, *Left* and *SI Appendix*, Fig. S9*B*). Overexpression of Aster-A effectively alleviates this accumulation, restoring PM cholesterol to normal levels. Strikingly, treatment of Aster-A-overexpressing cells with YKJ-86 completely reversed this cholesterol-lowering effect, driving PM cholesterol levels back to those observed in Aster-knockout cells (Fig. 7 *G*, *Upper* *Right* and *SI Appendix*, Fig. S9*B*). Similarly, the loss of Aster function in the intestine is known to impair cholesterol absorption, resulting in cholesterol accumulation in the PM of intestinal enteroids (6). Treatment of human enteroids with YKJ-86 led to an increase in accessible PM cholesterol (Fig. 7*H* and *SI Appendix*, Fig. S9*C*), further confirming the ability of YKJ-86 to modify Aster pathway function ex vivo.

Sterol analogs such as AI-3d show substantial cellular toxicity, similar to that of 25-HC (*SI Appendix*, Fig. S6*B*). YKJ-86 exhibited less toxicity compared to AI-3d. Beyond YKJ-86, we also identified YKJ-262 as a more potent inhibitor of Aster-A and -C (*SI Appendix*, Fig. S10 *A*–*D*). Treatment with YKJ-262 at 10 μM for 16 h did not lead to cholesterol accumulation in lysosomes. Functional assays in cells overexpressing Aster-A demonstrated that YKJ-262 efficiently reversed Aster-induced cholesterol depletion at the PM (Fig. 8*F* and *SI Appendix*, Fig. S9).

## Discussion

Aster proteins function in a tissue-specific and context-dependent manner, contributing to cell-type-specific cholesterol sensing and lipid homeostasis. However, tools to interrogate specific roles for individual Aster proteins have been limited. In this study, we designed and characterized nonsteroidal analogs with the ability to target all three Aster proteins, or to selectively inhibit Aster-A and/or Aster-C. These inhibitors offer distinct advantages over previously reported sterol-based Aster inhibitors, such as AI-3d, including reduced cytotoxicity and improved specificity for Aster cholesterol transport.

Our results identify YKJ-124 as an Aster-A selective inhibitor and YKJ-305 as an Aster-C–selective inhibitor, providing chemical tools to interrogate isoform-specific Aster biology. YKJ-124 enabled selective perturbation of Aster-A-dependent pathways in immune cells. In primary Th17 cells, YKJ-124 increased PM accessible cholesterol and reshaped Ca^2+^ signaling during T cell activation, closely phenocopying genetic Aster-A loss (8). YKJ-300 and YKJ-305 emerged as a distinct class of nonsteroidal inhibitors with strong selectivity for Aster-C over Aster-A and Aster-B. A major advance of this study is the cocrystal structures of YKJ-300 and YKJ-305 bound to the Aster-C sterol-binding domain, which provide a clear mechanistic basis for isoform selectivity. In both complexes, Ser477 of Aster-C (substituted by glycine in Aster-A and Aster-B) forms a key hydrogen bond with the ligands. This interaction is likely essential for high-affinity binding and offers a straightforward structural explanation for why YKJ-300 and 305 preferentially engage Aster-C.

Aster-C has the most restricted expression pattern of the family, being most prominently expressed in liver and intestine. Aster-C has been challenging to interrogate in vitro because it is poorly expressed in most commonly used cell lines. To establish that Aster-C could be targeted pharmacologically in a physiological setting, we tested YKJ-305 in vivo using liver-specific Aster-A knockout mice. YKJ-305 was well tolerated in mice and its administration blunted fasting-induced PM-to-ER cholesterol transfer, as evidenced by increased SREBP2 pathway gene expression and elevated protein expression of the cholesterol biosynthetic enzyme HMGCR. These findings provide functional evidence that acute pharmacological inhibition of Aster-C can reprogram hepatic cholesterol sensing in vivo.

We also showed that the nonsteroidal inhibitor YKJ-86 effectively targets the function of all three Aster proteins in cells in vitro. Treatment of fibroblasts or human enteroids with YKJ-86 led to increased cholesterol accumulation in the PM, mimicking the phenotype of Aster genetic knockout models. We also found that YKJ-262 is a dual inhibitor of Aster-A and -C. The ability of YKJ-262 to spare Aster-B opens additional possibilities for tissue-specific transport modulation with reduced off-target effects. For example, while Aster-A and -C are enriched in liver, Aster-B is predominantly expressed in steroidogenic tissues like the adrenal gland and ovary (3, 7).

A major barrier to developing useful Aster inhibitors is the potential for off-target effects, given the many biological pathways impacted by sterols. Sterol-based inhibitors face limitations due to their ability to interact with various steroid-binding proteins in cells. Conducting comprehensive off-target analysis is therefore critical. We incorporated experiments to rule out compounds that directly target other cholesterol transport pathways, including NPC1/2 and the Insig-SCAP system. Our lead nonsteroidal compounds are also less toxic to cells compared to the sterol inhibitor AI-3d. As widespread Aster binding in many tissues would likely have undesirable effects, the development of selective inhibitors provides a strategy to minimize toxicity.

The thiazole methyl group of YKJ-300/305 occupies a position analogous to the C19 methyl group of cholesterol (Fig. 4 *F* and *G*). A recent structural study of cholesterol–sphingomyelin interactions suggested that the C19 methyl group packs against sphingomyelin acyl chains in a manner that facilitates hydrogen bonding with sphingomyelin (17). This structural parallel raises the possibility that Aster proteins may compete with sphingomyelin for cholesterol and promote extraction of cholesterol from cholesterol–sphingomyelin complexes. PM cholesterol accessibility and Aster-mediated transport may be reciprocally linked, with lipid packing influencing Aster recognition and Aster activity, in turn, reshaping the accessible cholesterol pool. The similarity also suggests that YKJ-300/305 may mimic specific sterol-recognition features that regulate cholesterol accessibility and transfer. Further structural and biophysical studies will be required to test these possibilities.

Looking forward, our nonsteroidal compound library offers a highly adaptable platform that is easier to modify and optimize compared to traditional sterol-based inhibitors. This enhanced flexibility provides a foundation for future innovations, allowing for the incorporation of advanced technologies such as proteolysis-targeting chimeric (PROTAC) technology (18). Furthermore, the modular nature of these nonsteroidal compounds allows for rapid adjustments to improve potency, selectivity, and pharmacokinetic properties, enhancing their potential for clinical use. Asters have been implicated in sterol homeostasis in key metabolic tissues, including liver and intestine. By selectively modulating Aster function, nonsteroidal inhibitors have the potential to offer approaches for managing metabolic disorders, such as hypercholesterolemia and metabolic dysfunction-associated steatotic liver disease (MASLD). Further preclinical studies are needed to explore the potential of Aster inhibitors for therapeutic applications.

## Methods

### Cell Culture.

CHO-K1 cells were obtained from the American Type Culture Collection and maintained in a 1:1 mixture of F-12K medium and Dulbecco’s Modified Eagle’s media. The media also contained 100 units/mL penicillin and 100 mg/mL streptomycin and 5% fetal bovine serum. Immortalized mouse fibroblast cells were plated in Dulbecco’s Modified Eagle’s media containing 10% FBS. The same media containing 1% lipoprotein-deficient serum, simvastatin (5 μM), and mevalonate (10 μM) was used for depletion of cholesterol.

### Human Enteroid Culture and Treatment.

Deidentified human jejunal enteroids (a gift of Martin Martin, UCLA, Los Angeles) were maintained in human enteroid culture medium as described (6). Human enteroids were differentiated for 5 d in differentiation media (Advanced DMEM/F12, 2 mM GlutaMAX, 10 mM HEPES, 100 U/mL penicillin, 100 μg/mL streptomycin, 1× N2 supplement, 1× B27 supplement, 50 ng/mL EGF, 50 ng/mL noggin, 50 ng/mL r-Spondin, 500 nM A83-01, 10 μM Y-27632, 2.5 μg/mL fungizone, and 200 μg/mL normocin). For the experiment, human enteroids were depleted of cholesterol in differentiation medium containing 1 µM Ro 48-8071 and 50 µM mevalonate for 16 h. Enteroids were then pretreated with vehicle or 10 µM YKJs in cholesterol-deprived differentiation medium for 5 h before the administration of vehicle or 150 µM mβCD-cholesterol for 1 h in the presence or absence of 10 µM YKJs. Upon completion of the experiment, enteroids were released from Matrigel for ALOD4 staining (6).

### Binding Assays.

The protocol for Aster binding assays was previously described (3). In brief, 3 µM of purified N-terminal GST-tagged Aster-A334–562, Aster-B303–533, and Aster-C296–517 were incubated with vehicle or compounds in binding buffer (0.003% Triton X-100 in 1× PBS) for 30 min. Subsequently, NBD-Cholesterol was added to achieve a final concentration of 500 nM, and the mixture was incubated for an additional 30 min. Measurements were taken using a CLARIOstar microplate reader, with excitation at λ(ex) = 470 nm and emission at λ(em) = 525 nm.

### Staining.

Aster triple-KO fibroblasts were depleted of sterols by incubating in DMEM containing 1% LPDS, simvastatin (5 μM), and mevalonate (10 μM) for 16 h at 37 °C. Cells were then switched to the same medium containing vehicle or YKJs for 6 h. For the final 1 h, 35 μM MβCD–cholesterol was added. Cells were subsequently washed three times with DPBS (containing Ca^2+^/Mg^2+^ and 0.2% fatty acid free bovine serum albumin), then incubated with ALOD4–488 (20 μg mL^−1^ in DPBS + 0.2% BSA) for 2 h with shaking at 4 °C as described previously (5). After three washes with cold DPBS, cells were fixed with 4% paraformaldehyde (PFA) at room temperature for 15 min. Coverslips were mounted with ProLong Diamond Antifade with DAPI (Invitrogen, P36962). Images were acquired with a Leica TCS-SP8-SMD Confocal Microscope. CHO-K1 cells were incubated in a 1:1 mixture of F-12K medium and Dulbecco’s Modified Eagle’s Medium, supplemented with 5% FBS and either the indicated compounds or vehicle control, for 16 h at 37 °C. After incubation, cells were fixed with 4% PFA at room temperature for 30 min. Cholesterol was visualized by incubating cells with filipin (5 μg/mL) in 1× PBS containing 1% FBS for 30 min at room temperature. Images were captured using 405 nm UV excitation.

### Western Blotting.

For the SREBP2 cleavage assay, CHO-K1 cells were depleted of cholesterol for 16 h at 37 °C. Cells were then switched to the same medium with vehicle or 25-HC or YKJs for 2 h. For the cholesterol transport analysis, CHO-K1 cells deplete cholesterol, as shown above. The cells were then treated with vehicle or YKJs for 4 h, followed with or without 35 μM mβCD-cholesterol for another 2 h. Cells were harvested in RIPA (Boston BioProducts, BP-115) supplemented with protease inhibitor (Thermo Scientific Pierce, A32965) and phosphatase inhibitor (Thermo Scientific Pierce, A32957) cocktail tablets. Protein concentrations were measured using the Bicinchoninic Acid Assay Protein Assay kit (Thermo Scientific Pierce, PI23223/4). Equal amounts of protein were loaded onto NuPAGE 4 to 12% gels (Invitrogen, NP0321). Immunoblots were performed with anti-SREBP2 antibody (7D4) or actin.

### Toxicity Assessment.

CHO-K1 cells were seeded in a 6-well plate and treated with either the vehicle or YKJ compounds for 72 h. After treatment, dead cells were removed by washing the cells three times with PBS. The remaining cells were then fixed with 4% PFA at room temperature for 30 min. Following fixation, the cells were stained with a 0.1% crystal violet solution (Sigma) at room temperature for 30 min, and then washed 3 to 5 times with water to remove excess stain.

### Ca^2+^ Flux Assay.

Th17 cells were differentiated as previously described (8). Day 4 Th17 cells were removed from differentiation cocktail and rested overnight in fresh X-VIVO15 media without any cytokine. For flow cytometry-based assay, T cells were labeled with 1 μM Fluo-4-AM and 1 μM FuraRed-AM mixed 1:1 with 20% Pluronic F-127 in DMSO for 30 min and resuspended in Ringer’s solution. Live single cells were gated based on forward and side scatter parameters. Fluo-4 (Ca^2+^ bound) and FuraRed (Ca^2+^ unbound) fluorescence were recorded on the following sequence: 1) 3 min of baseline acquisition; 2) 15 min following the addition of 5 μg/mL of biotin-anti-CD3ε and biotin-anti-CD28; 3) 15 min following the addition of 10 μg/mL streptavidin (crosslinking agent), 4) 5 min following addition of 1 μM Ionomycin. Kinetics platform in FlowJo v10 was used to plot time vs. median Fluo-4/FuraRed ratio.

### Protein Expression and Purification.

Protein expression and purification of mouse Aster-C_296-517_ was undertaken as described previously (11). Excess of YKJ-300 or YKJ-305 was added to partially purified Aster-C domain (molar ratio 1:5, protein:ligand) from a 50 mM stock solution dissolved in ethanol before loading the complex on a Superdex S-75 column for size exclusion chromatography. The peak fractions were concentrated to 7.5 mg/mL and used for crystallization. Site-directed mutagenesis was performed to generate the following mouse Aster-A_334-562_ mutant: Aster-A_G520S and the following mouse Aster-C_296-517_ mutant: Aster-C_S477G. The resulting inserts were cloned and purified as described previously (11), without adding any compound to the protein samples. Instead, 10% of glycerol was added to the size exclusion chromatography, and the resulting samples were used for binding assays.

### Crystallization and X-ray Structure Determination.

Crystals of the Aster-C_296-517_ in complex with YKJ-300 and YKJ-305 ligands were obtained using sitting drop vapor diffusion at room temperature. Crystals of Aster-C_296-517_ in complex with YKJ-300 were grown using 0.15 M Potassium bromide, 30% PEG 2000 MME (condition G10, JCSG+, Molecular Dimensions) and crystals of Aster-C_296-517_ in complex with YKJ-305 were grown using 0.2 Ammonium nitrate, 20% PEG 3350 (condition C3, JCSG+, Molecular Dimensions). Micro seeding procedure was used using a micro seed stock from Aster-C_296-517_ apo grown in 0.2 M Sodium iodide 0.1 M Bis-tris propane pH 7.5 and 20% PEG 3350 (condition G3, PACT, Molecular Dimensions). The crystals were cryoprotected with 30% glycerol. Data were collected to 2.0 and 1.75 Å respectively on the I04 beamline at Diamond Light Source, UK. Data were processed using XDS (within Xia2) and Pointless/Aimless (within CCP4) (19–21). The structures were solved using molecular replacement using Phaser (within CCP4) (22) and the Aster-C structure (PDB ID code 8AXW) as a template (6). Model fitting and refinement were performed using Coot, Refmac (within CCP4), and Phenix (23–26).

### Mouse Studies.

Aster-A liver-specific KO mice (Albumin-Cre; Aster-A flox/flox) were fed a chow diet containing 0.008% Atorvastatin (Research Diets, D18062105i) and provided with drinking water [5 mg/L simvastatin sodium salt (Millipore Sigma, 5670215MG)] for 5 d. At 24 h, −16 h, and 4 h prior to harvest, mice were IV injected with Vehicle (5% DMSO, 45% PEG400, 50% Saline) or 5 mg/kg body weight YKJ-305. Mice were fasted for 16 h overnight. Liver and blood plasma were harvested.

### Lipidomic Analysis.

Liver (50 to 100 mg) was collected in a 2-mL homogenizer tube preloaded with 2.8-mm ceramic beads (Omni 19-628). PBS (0.75 mL) was added to the tube and the sample was homogenized in an Omni Bead Ruptor Elite (three cycles of 10 s at 5 m s^−1^ with a 10-s dwell time). Homogenate containing 2 to 6 mg of tissue was transferred to a glass tube for extraction. A modified Bligh and Dyer extraction was carried out on all samples (27). Before biphasic extraction, an internal standard mixture consisting of 70 lipid standards across 17 subclasses was added to each sample (AB Sciex, 5040156; Avanti, 330827, 330830, 330828, and 791642). Following two successive extractions, pooled organic layers were dried in a Thermo SpeedVac SPD300DDA using ramp setting 4 at 35 °C for 45 min with a run time of 90 min. Lipid samples were resuspended in 1:1 methanol/dichloromethane with 10 mM ammonium acetate and transferred to robovials (Thermo, 10800107) for analysis. Samples were analyzed on the Sciex 5500 with DMS device (Lipidyzer Platform) with an expanded targeted acquisition list consisting of 1,450 lipid species across 17 subclasses (or the original acquisition list of 1,100 lipids across 13 subclasses). A Differential Mobility Device on Lipidyzer was tuned with EquiSPLASH LIPIDOMIX (Avanti, 330731). Data analysis was performed on an in-house data analysis platform comparable to the Lipidyzer Workflow Manager (28). Instrument method including settings, tuning protocol, and MRM list is available from Su et al. (28) Quantitative values were normalized to mg of protein.

### Compound Synthesis.

See *SI Appendix*.

## Supplementary Material

Appendix 01 (PDF)

## Data Availability

X-ray structure data have been deposited in PDB (30JR (29); 30JI (30)).
