# Supplementary material for: Design and structural basis of selective nonsteroidal inhibitors of aster cholesterol trafficking
Source: Proc Natl Acad Sci U S A. 2026 Sep 1;123(36):e2617638123. doi: 10.1073/pnas.2617638123 (PMC13552655; doi:10.1073/pnas.2617638123)
Supplement: Supplementary file 1 [file pnas.2617638123.sapp.pdf]

PNAS

Supporting Information for

Design and Structural Basis of Selective Non-Steroidal Inhibitors of Aster Cholesterol Trafficking

Hyunsoo Kim, Beatriz Romartinez-Alonso, Xu Xiao, Yajing Gao, John Paul Kennelly, Alessandra Ferrari, Rui Li, Liujuan Cui, Emily Whang, John W. R. Schwabe, Michael E. Jung, and Peter Tontonoz

Corresponding author: Peter Tontonoz

Email: ptontonoz@mednet.ucla.edu

This PDF file includes:

Figures S1 to S11

Other supporting materials for this manuscript include the following:

Compound synthesis data.

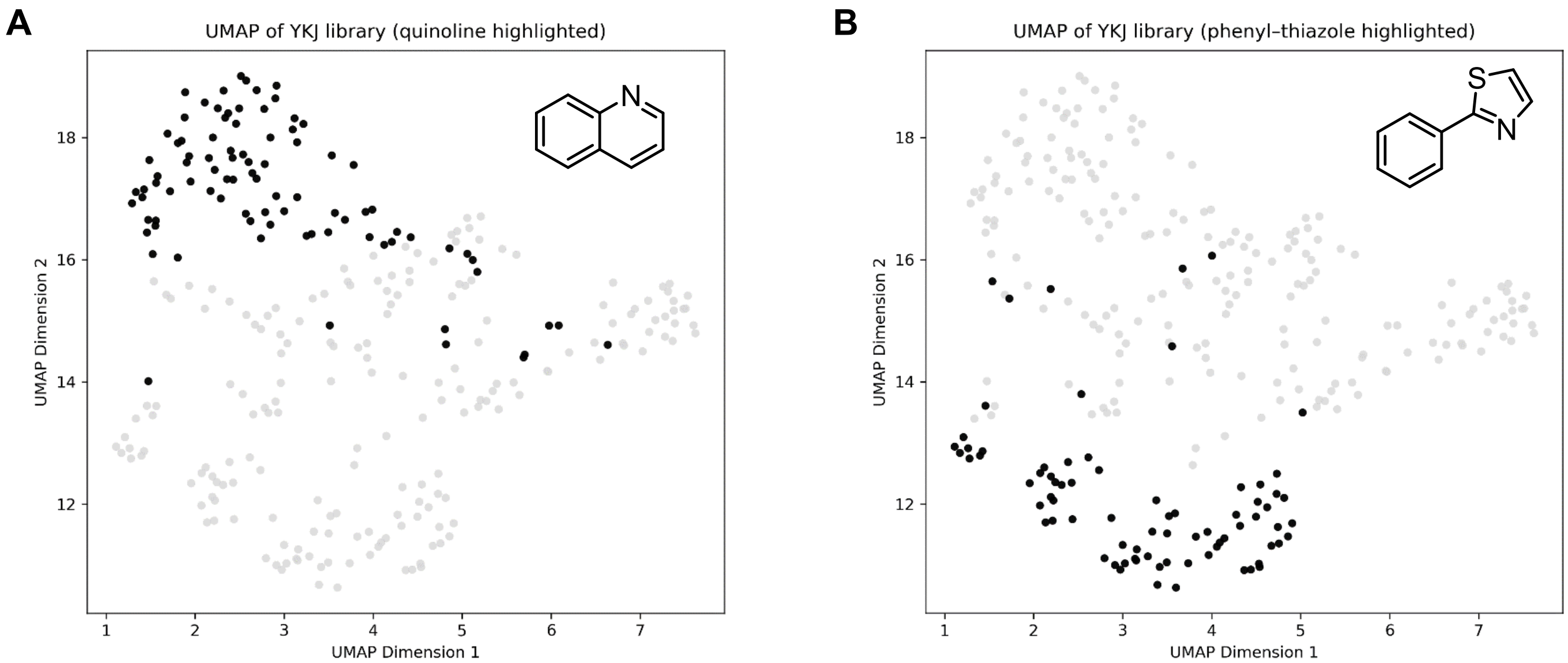

Figure S1. Scaffold-dependent distribution of quinoline and phenyl thiazole analogs in the YKJ chemical space.

(**A**) UMAP projection of the YKJ library with quinoline-containing compounds highlighted in black against the full library shown in gray. Quinoline analogs cluster in a region associated with relative Aster-A preference.

(**B**) UMAP projection of the YKJ library with phenyl thiazole-containing compounds highlighted in black against the full library shown in gray. Phenyl thiazole analogs are enriched in a distinct region associated with relative Aster-C preference.

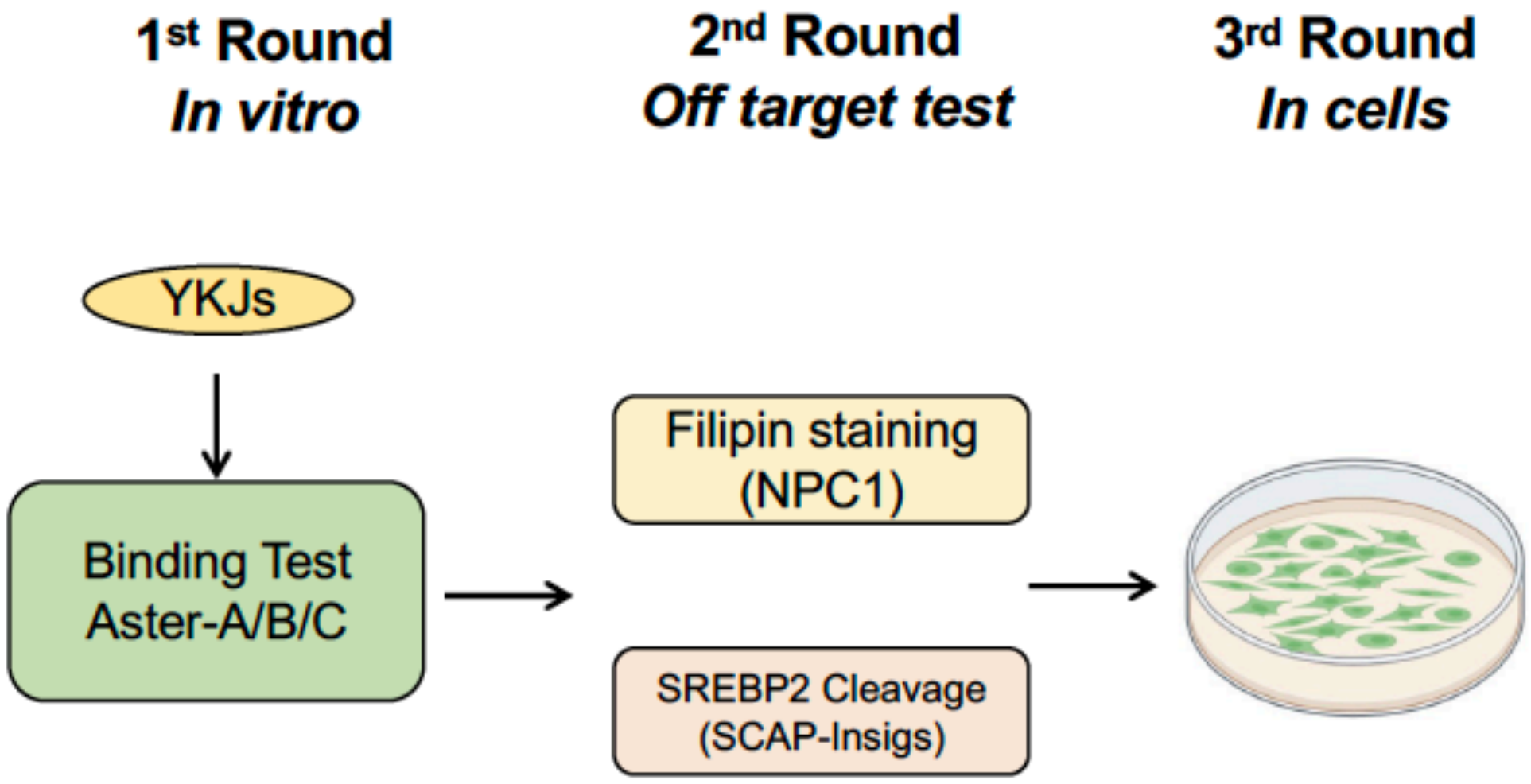

Figure S2. The process of non-steroidal analogues screening.
Candidate YKJ compounds were first evaluated for direct binding to Aster-A, Aster-B, and Aster-C. Compounds showing Aster binding were then subjected to off-target counter-screens, including filipin staining to assess NPC1-related lysosomal cholesterol accumulation and measurement of SREBP2 cleavage to monitor perturbation of the SCAP–Insig pathway. Compounds that passed these filters were advanced to a third round of cell-based assays to test effects on Aster-dependent cholesterol transport.

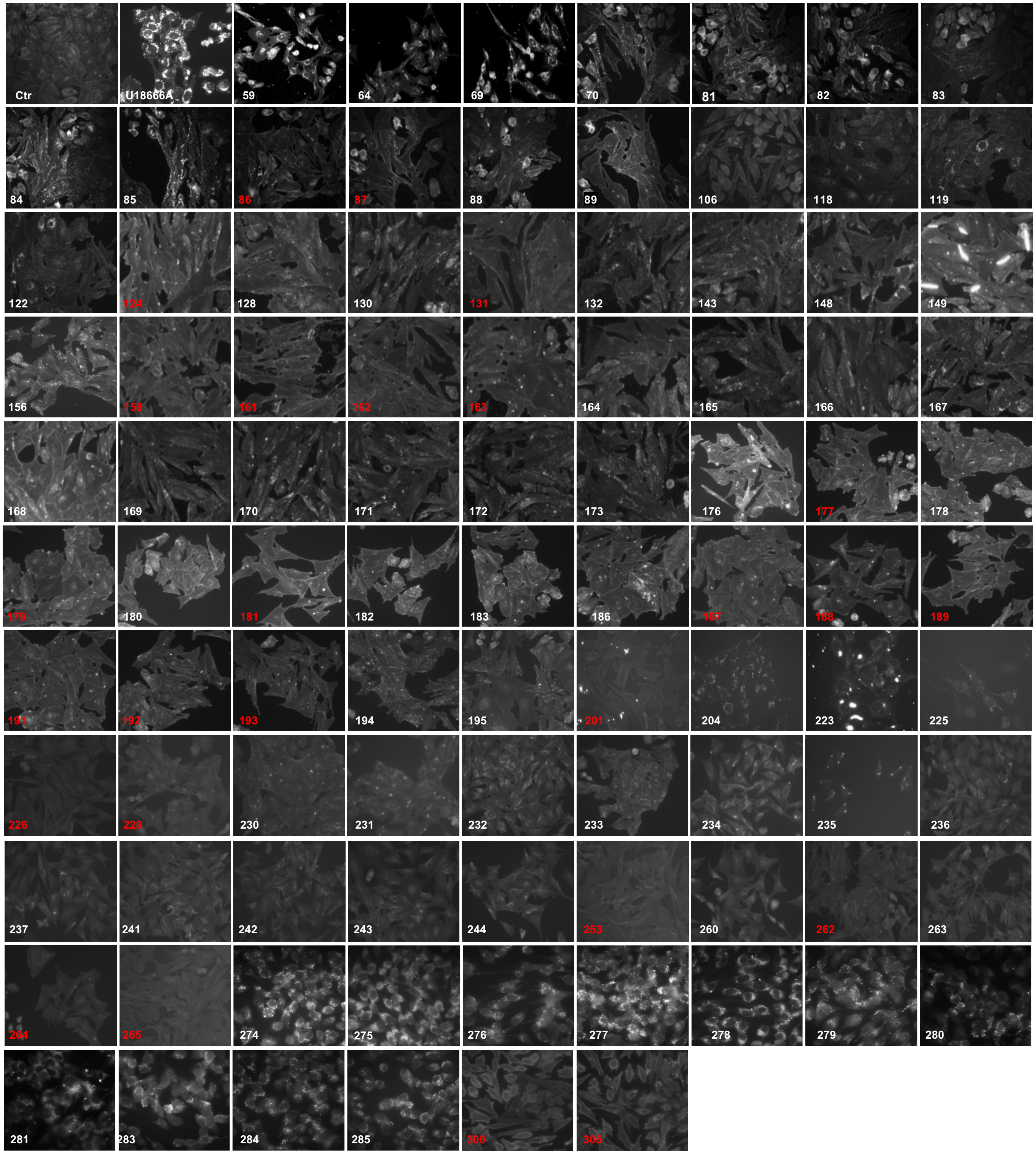

Figure S3. Filipin staining test of YKJs.
CHO-K1 cells were treated with YKJ compounds (10 μM, 16 h) and stained with filipin. U18666A served as a positive control for NPC1 inhibition. Compounds labeled in red did not produce the U18666A-like lysosomal cholesterol accumulation pattern, indicating no detectable NPC1 inhibition under these conditions.

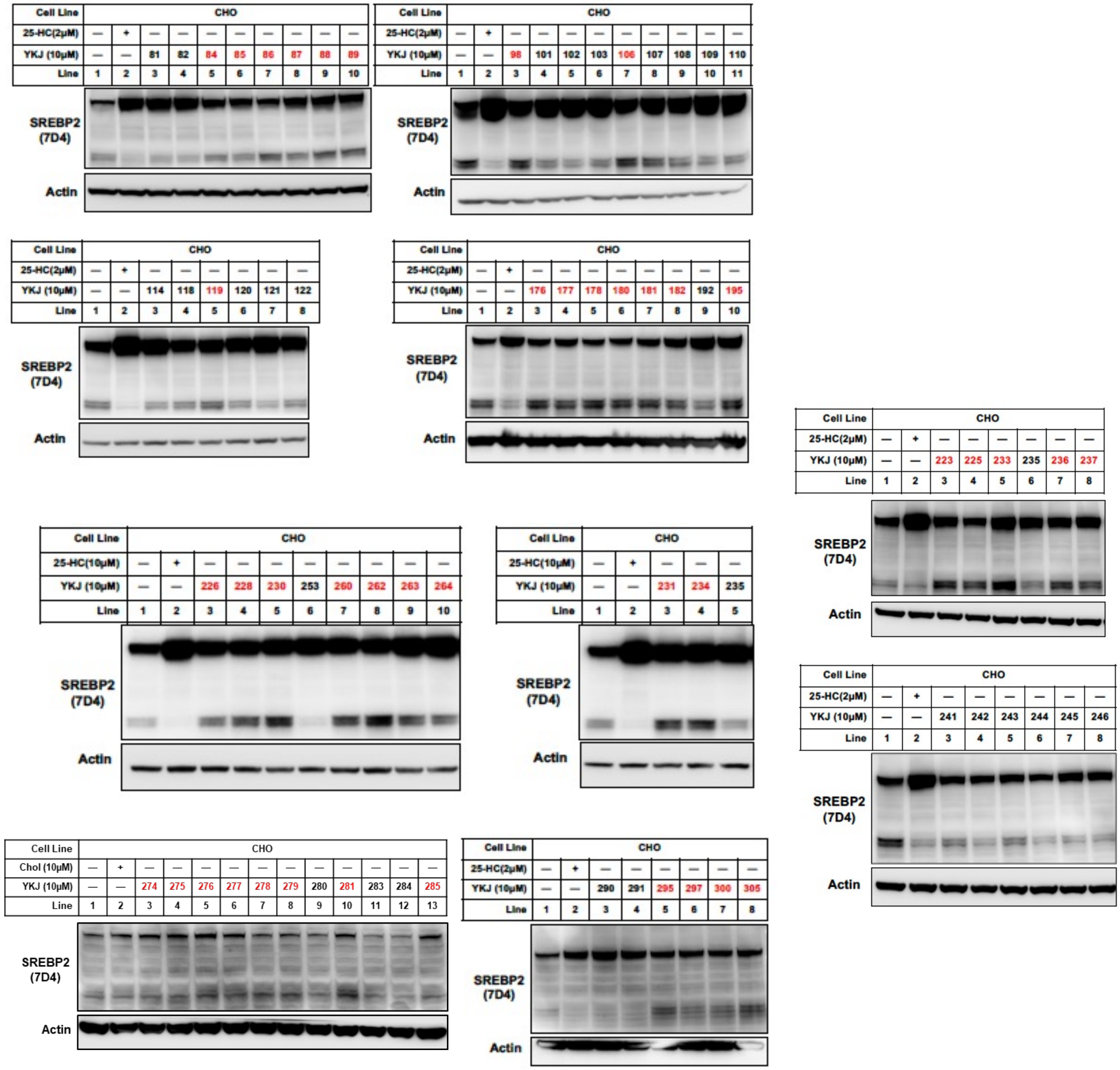

Figure S4. SREBP2 cleavage screen for SCAP–Insig off-target activity.
CHO-K1 cells were treated with the indicated YKJ compounds (10 μM) and SREBP2 processing was assessed by immunoblotting as a readout of SCAP–Insig pathway perturbation. Compounds labeled in red did not alter SREBP2 cleavage compared with vehicle, indicating no detectable inhibition of the SCAP–Insig pathway under these conditions.

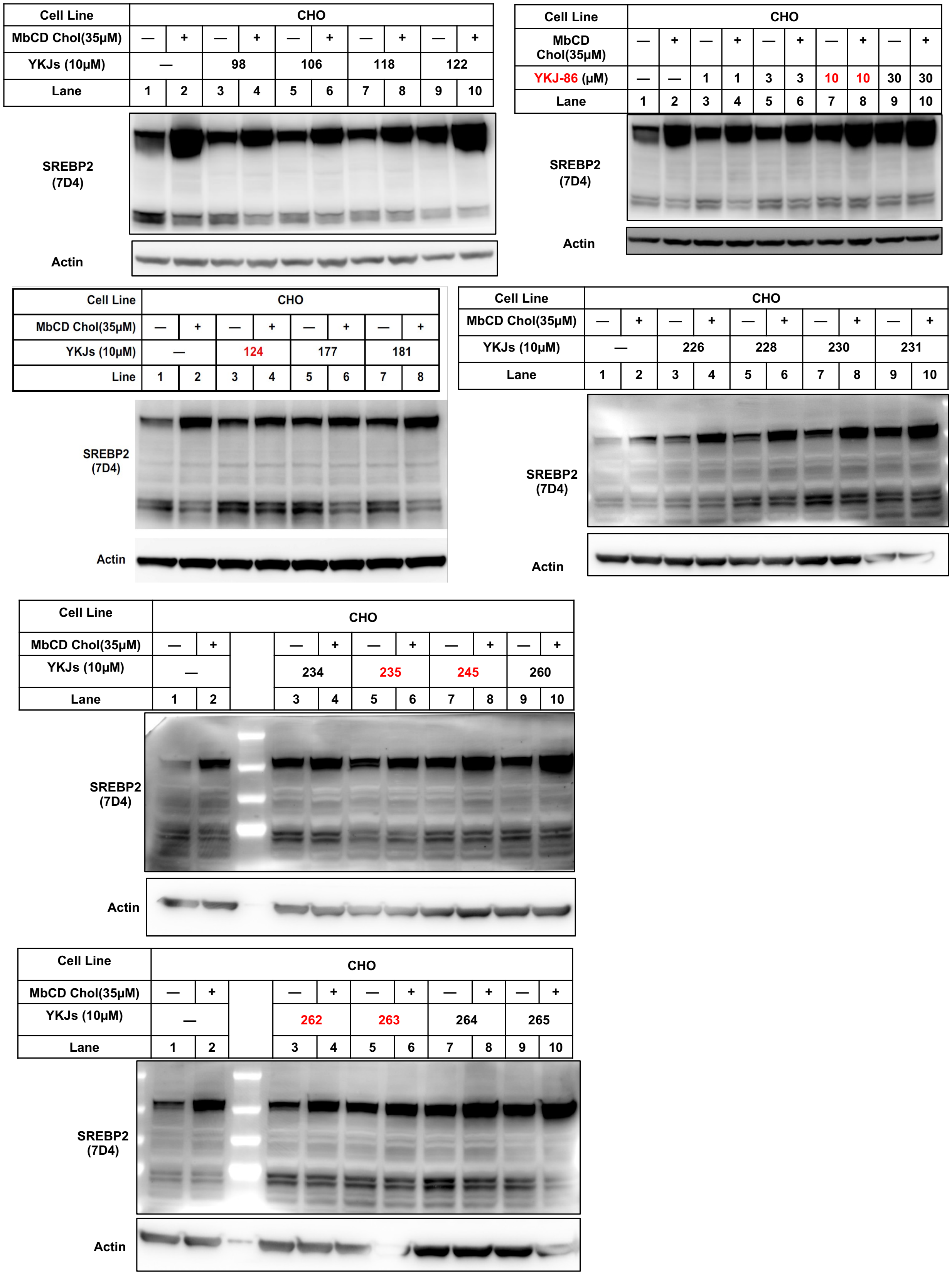

Figure S5. Cell-based cholesterol transport test.

CHO-K1 cells were first cholesterol-depleted to activate SREBP2 processing and then loaded with MβCD–cholesterol to restore ER cholesterol and suppress SREBP2 cleavage. Cells were treated with the indicated YKJ compounds (10 μM), and SREBP2 cleavage was assessed by immunoblotting (Actin, loading control). Compounds that block PM-to-ER cholesterol transport attenuate the ability of MβCD–cholesterol to suppress SREBP2 cleavage, indicating impaired delivery of cholesterol to the ER.

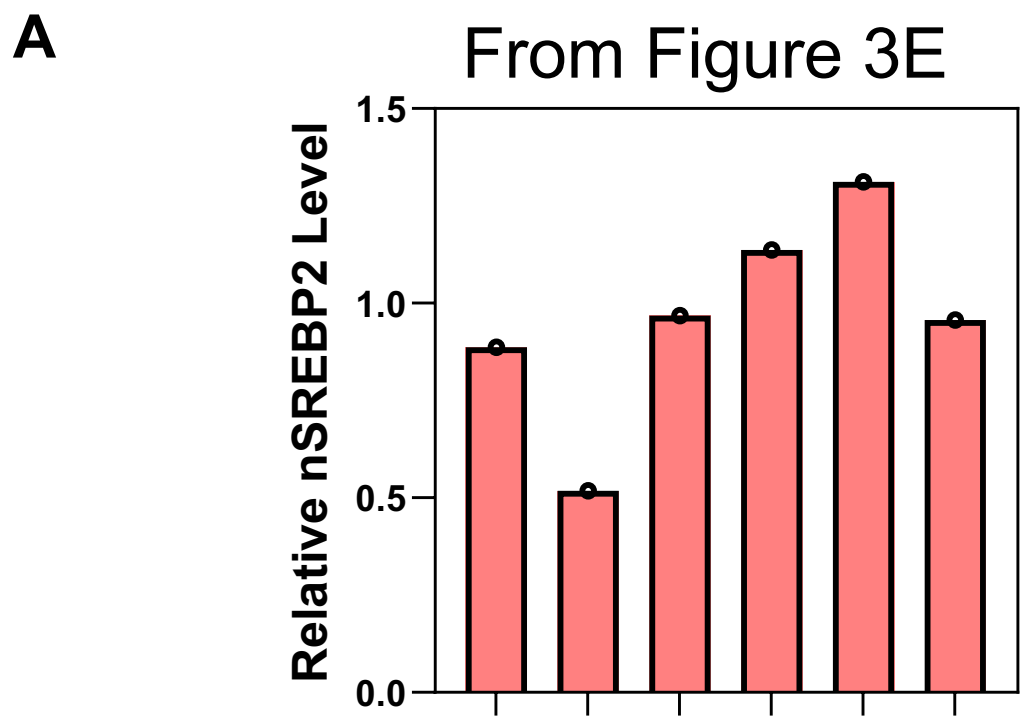

Lane	1	2	3	4	5	6
MβCD-Chol(35μM)	—	+	—	+	—	+
YKJ-124 (μM)	—	—	1	1	3	3

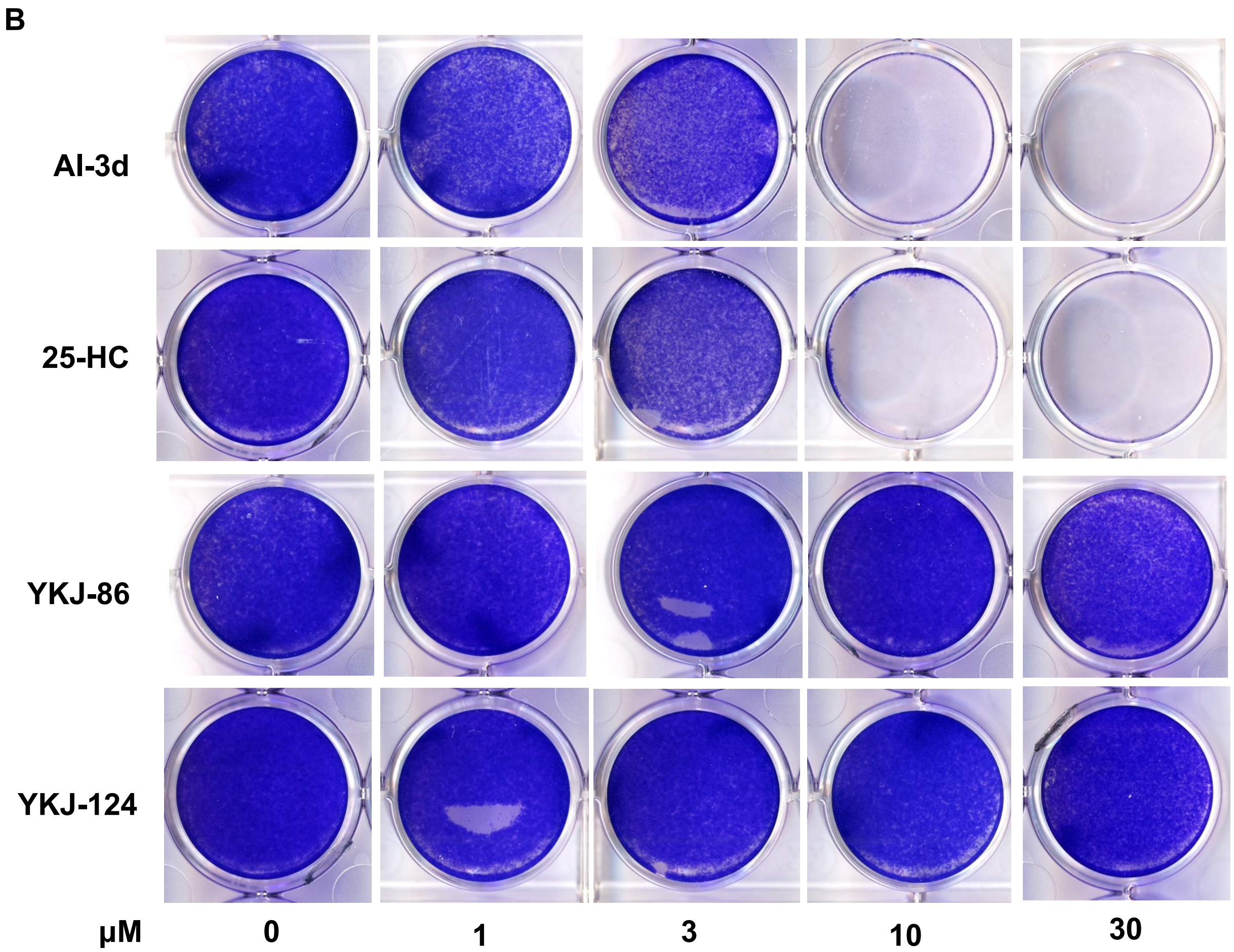

Figure S6. Cell viability test for YKJ-86 and YKJ-124 by crystal violet staining.
(**A**) Quantification of nSREBP2 western blots shown in Fig. 3E.
(**B**) Representative crystal violet stained wells following treatment with the indicated compounds (as labeled in the figure). Crystal violet intensity reflects adherent cell mass/viability, with reduced staining indicating decreased cell survival or proliferation under the corresponding treatment conditions.

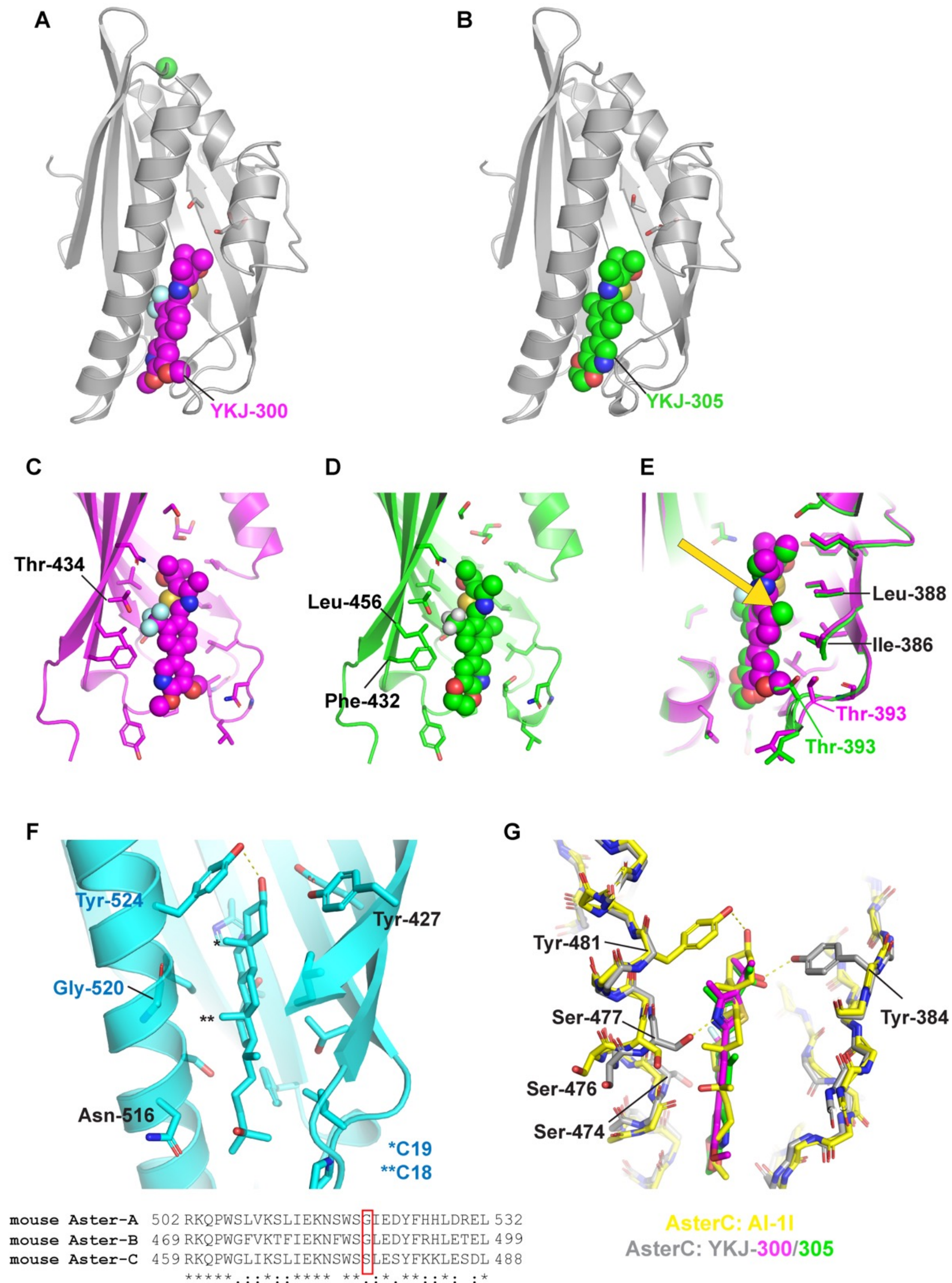

Figure S7. Aster-C in Complex with YKJ-300 and YKJ-305. (A) Crystal structure of the Aster-C domain in complex with YKJ-300. **(B)** Structure of the Aster-C domain in complex with YKJ-305. **(C)** Detailed view of YKJ-300 within the Aster-C binding pocket highlighting the hydrophobic region accommodating the -CF_3 group. The ligand is shown in sphere representation, while surrounding residues are depicted as sticks. Thr434 is identified as the residue in closest proximity to the −CH_3 substituent. **(D)** View of YKJ-305 within the Aster-C binding pocket, illustrating the environment surrounding one of the -CH_3 groups. The ligand is shown in spheres; residues within van der Waals distance, including Phe432 and Leu456, are shown as sticks. **(E)** Overlay of the Aster-C domain bound to YKJ-300 (magenta) and YKJ-305 (green), highlighting the position of Thr393. The different positioning of Thr393 demonstrates the flexibility of the loop that closes over the ligand and its ability to adapt to diverse ligand scaffolds. The yellow arrow indicates the methyl group of YKJ-305 that engages in hydrophobic interactions with Ile386 and Leu388. **(F)** Close-up view of the Aster-A binding pocket bound to 25-hydroxy-cholesterol (PDB ID: 6GQF). Amino acids forming the binding pocket are shown as sticks, with key interacting residues, Tyr524 and Gly520, labelled. Axial methyl groups of 25-hydroxycholesterol are indicated by asterisks. Sequence alignment of mouse Aster-A, -B and -C reveals a glycine in Aster-A and -B at the position corresponding to Ser477 in Aster-C. **(G)** Overlay of the Aster-C bound to YKJ-300/305 (grey) and AI-1I (yellow) (PDB ID: 7AZN). A slight conformational shift in the helix is observed upon binding of YKJ-300/305, driven by the orientation of Ser477 toward the thiazole nitrogen to facilitate hydrogen bond formation.

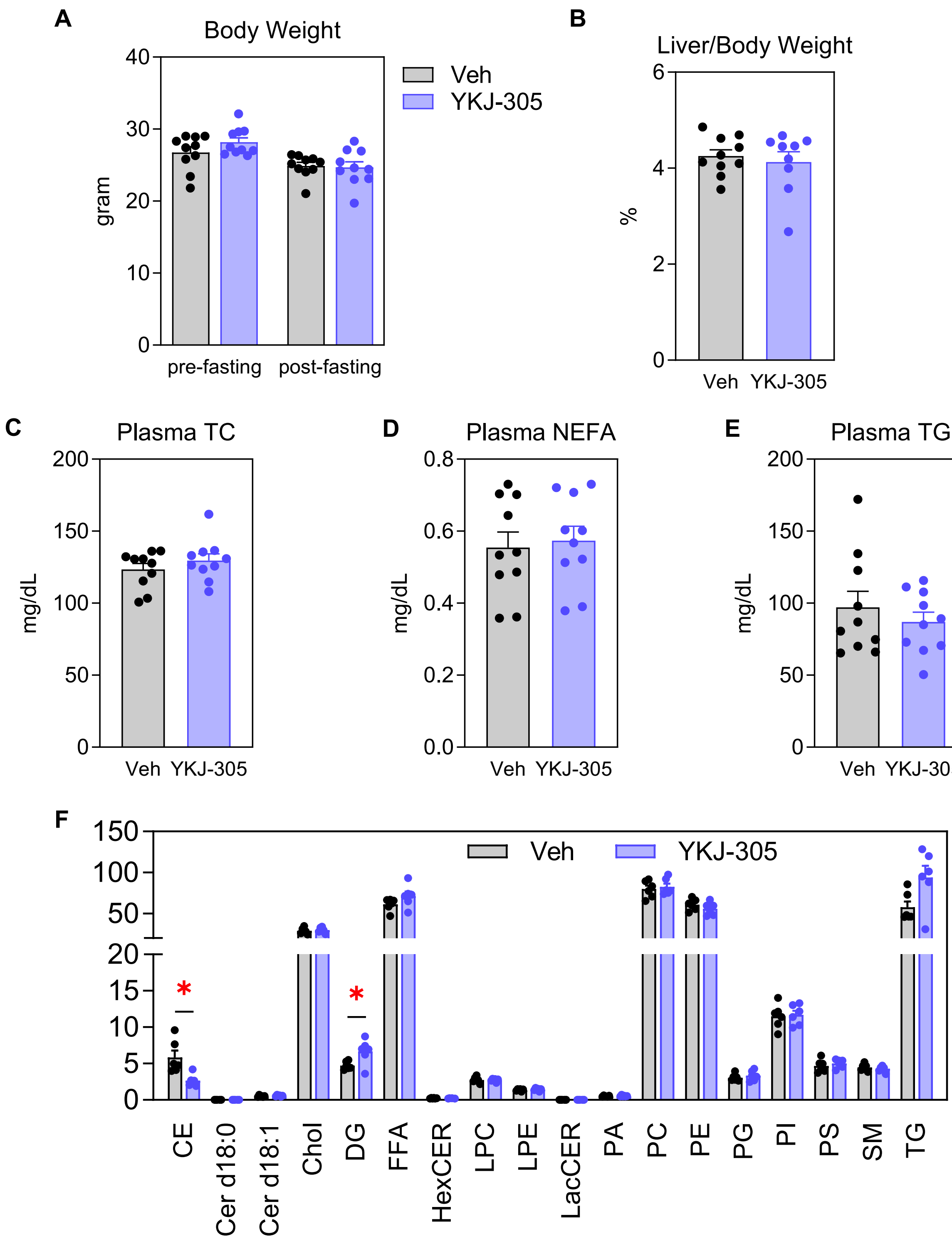

Figure S8. Mouse weight and lipids data after YKJ-305 injection.
(**A**) Bodyweight of mice pre- and post-fasting and after the vehicle or YKJ-305 administration (n = 10/10).
(**B**) Liver/bodyweight ratio of mice pre- and post-fasting and after the vehicle or YKJ-305 administration (n = 10/9).
(**C–E**) Plasma total cholesterol (TC) (A), non-esterified fatty acids (NEFA) (B), and triglycerides (TG) (C) measured in mice treated with vehicle (Veh) or YKJ-305; ad libitum–fed mice are shown as an additional reference group. Bars represent mean ± SEM, n=10/10/1.
(**F**) Lipidomics analysis of lipid species from mouse liver after vehicle or YKJ-305 administration (n = 6/6).

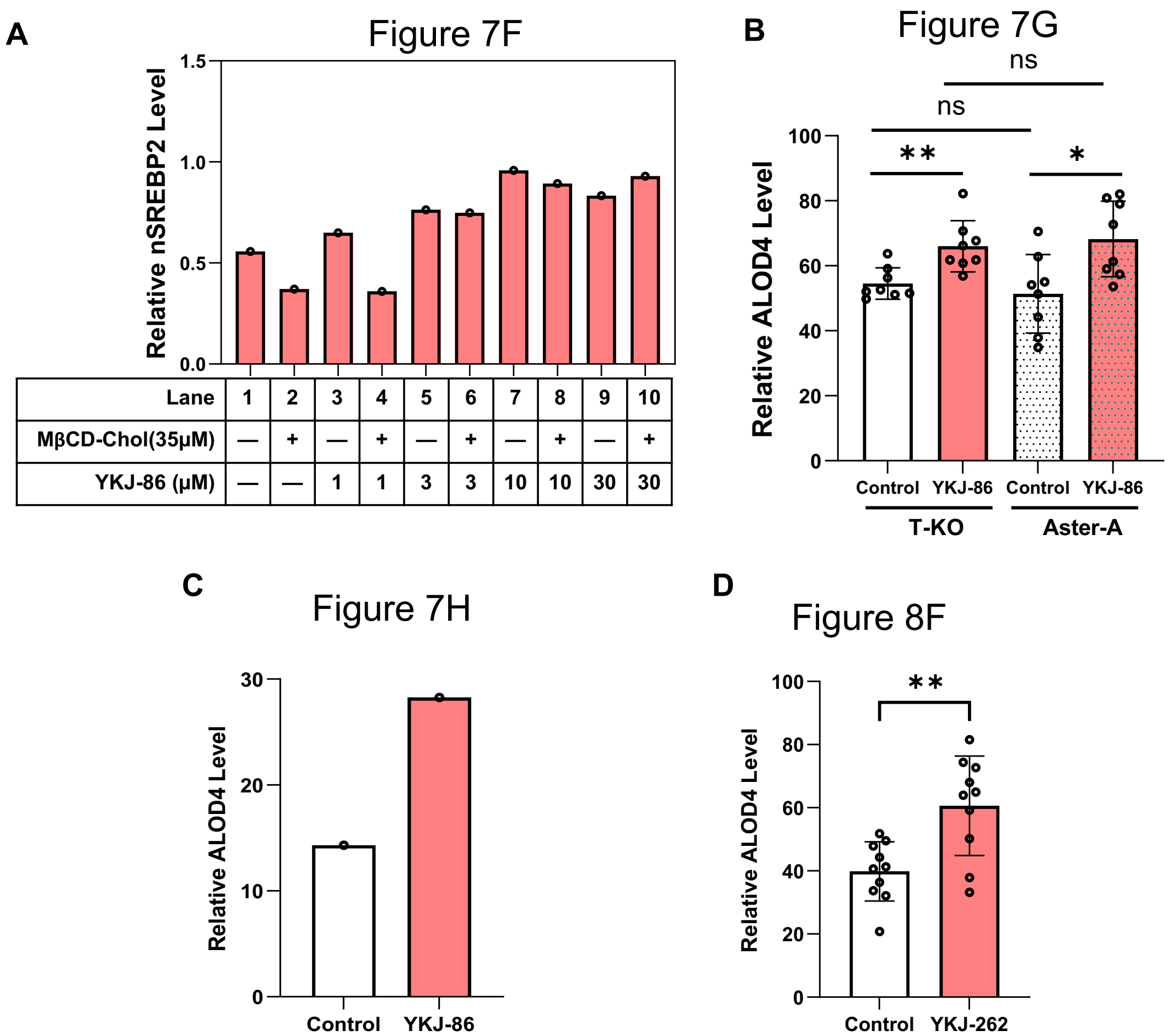

Figure S9. Western blots and ALOD4 image quantification.
(A) Quantification of nSREBP2 western blots shown in Fig. 7F.
(B-D) Quantification of ALOD4 staining shown in Fig. 7G (B), Fig. 7H (C) and Fig. 8F (D).

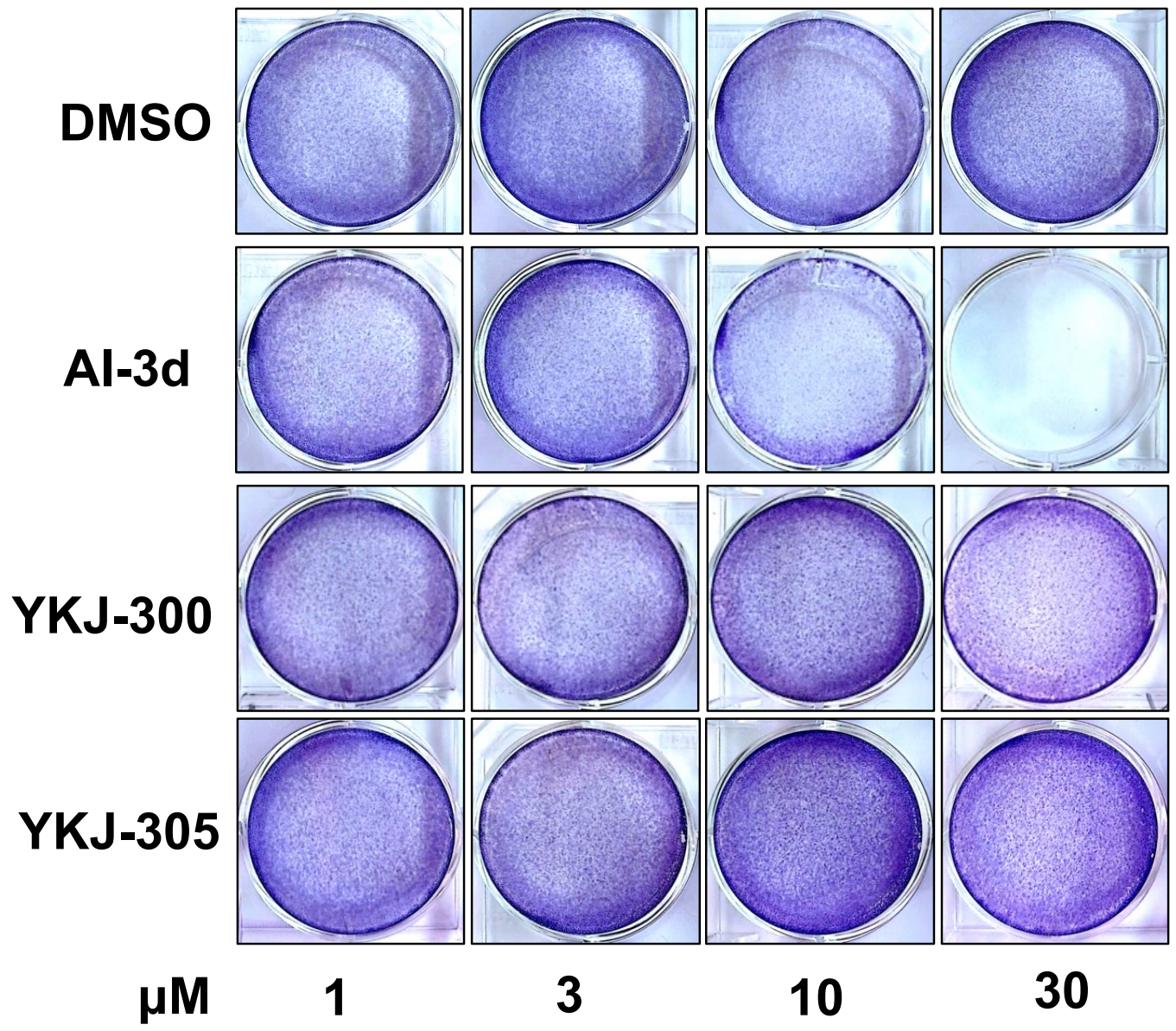

Figure S10. Cell viability test for YKJ-300 and YKJ-305 by crystal violet staining.
Representative crystal violet stained wells following treatment with the indicated compounds (as labeled in the figure). Crystal violet intensity reflects adherent cell mass/viability, with reduced staining indicating decreased cell survival or proliferation under the corresponding treatment conditions.

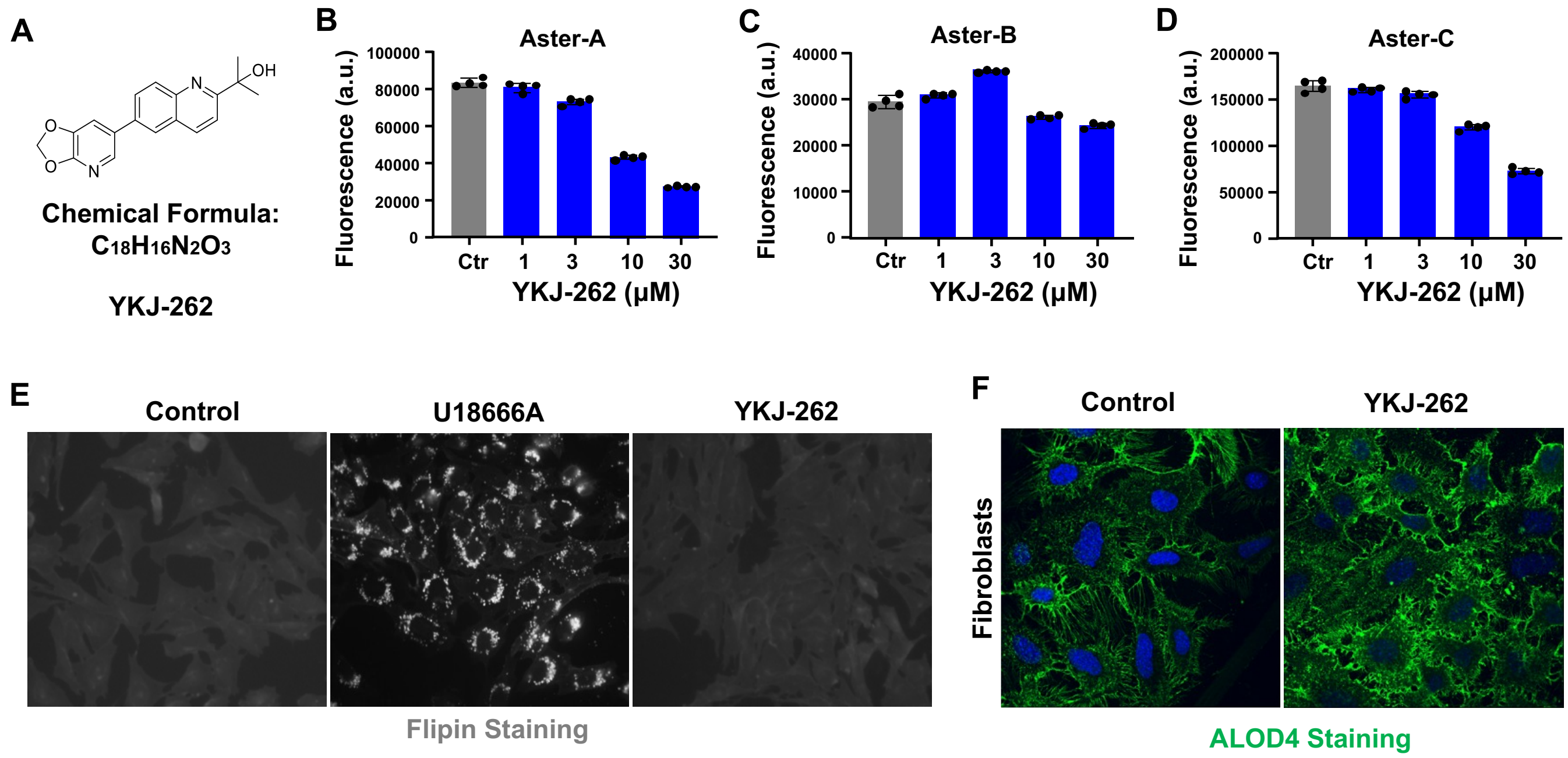

Figure S11. YKJ-262 selectively targets Aster-A and C.
(**A**) Structure of YKJ-262.
(**B-D**) Competition assay in which purified Aster domains of Aster-A (B), Aster-B (C) and Aster-C (D) were incubated with 22-NBD-cholesterol in the presence of vehicle or YKJ-262 at indicated concentrations. Data bars represent means ± SD.
(**E**) CHO-K1 cells were incubated in medium containing 5% FBS, U18666A and YKJ-262 (10 µM) or vehicle for 16 h at 37 °C. Cholesterol was visualized by filipin staining.
(**F**) ALOD4 staining of Aster-null fibroblasts with Aster-A overexpression, treated with vehicle or YKJ-262, to assess plasma membrane–accessible cholesterol. Images were acquired 1 h after MβCD–cholesterol loading.

Supplementary Information for

YKJ Compounds: Structures, Synthetic Procedures, and Spectral Data

Full List of YKJ Compounds

Preparation of YKJ Compounds

Spectral Data for YKJ Compounds

NMR Spectra of Selected YKJ Compounds

Full List of YKJ Compounds

35 Naphthalene Analogs

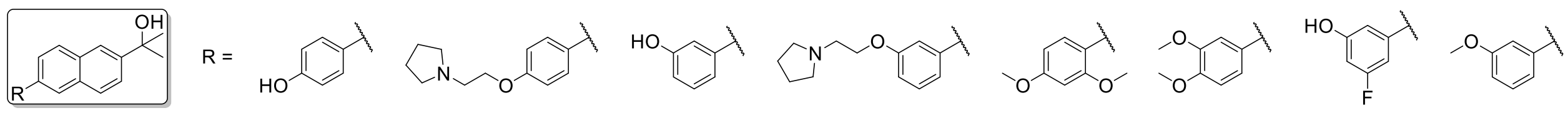

YKJ-51 YKJ-52 YKJ-53 YKJ-54 YKJ-55 YKJ-56 YKJ-57 YKJ-58

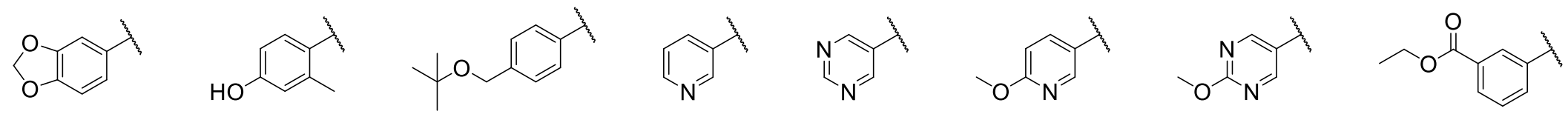

YKJ-59 YKJ-60 YKJ-61 YKJ-62 YKJ-63 YKJ-64 YKJ-65 YKJ-66

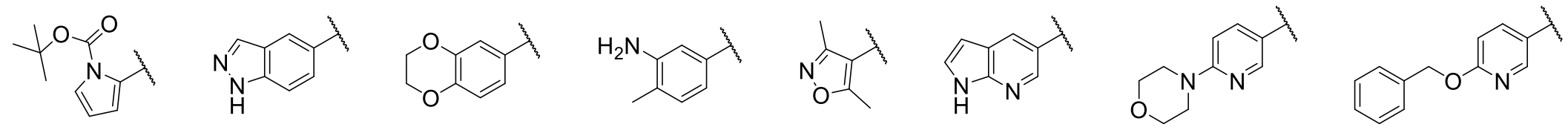

YKJ-67 YKJ-68 YKJ-69 YKJ-70 YKJ-71 YKJ-72 YKJ-73 YKJ-75

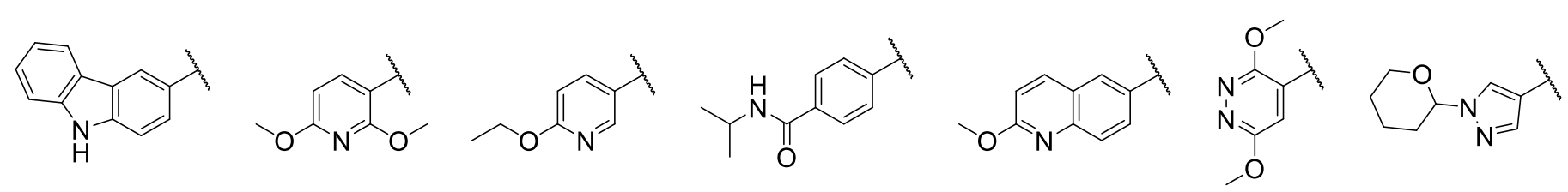

YKJ-76 YKJ-77 YKJ-78 YKJ-79 YKJ-80 YKJ-90 YKJ-91

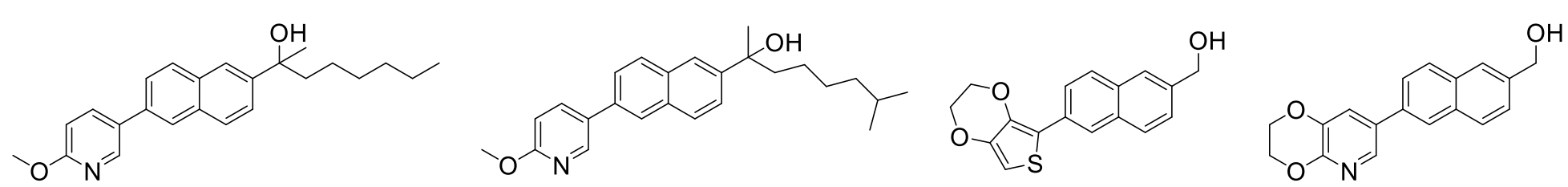

YKJ-123 YKJ-126 YKJ-265 YKJ-266

Broad scaffold diversification: 72 Analogs

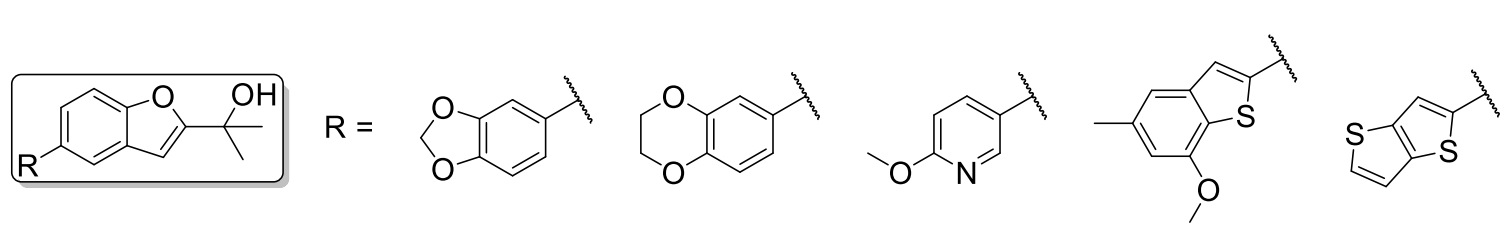

YKJ-160 YKJ-161 YKJ-162 YKJ-224 YKJ-225

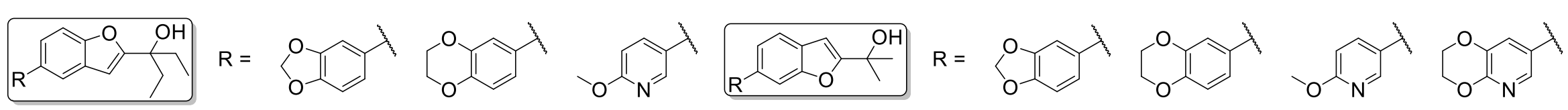

YKJ-164 YKJ-165 YKJ-163 YKJ-178 YKJ-177 YKJ-179 YKJ-207

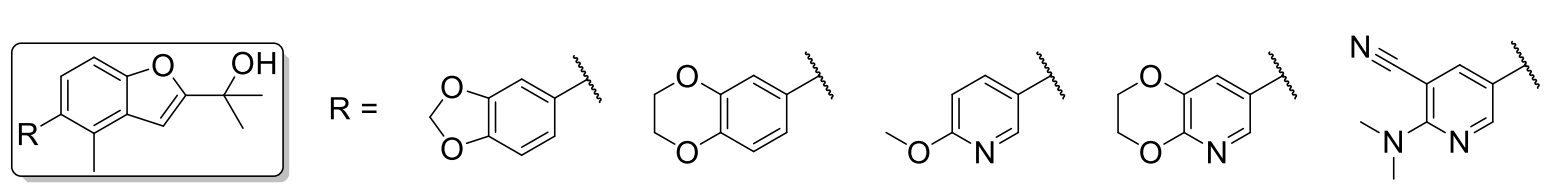

YKJ-203 YKJ-204 YKJ-205 YKJ-218 YKJ-219

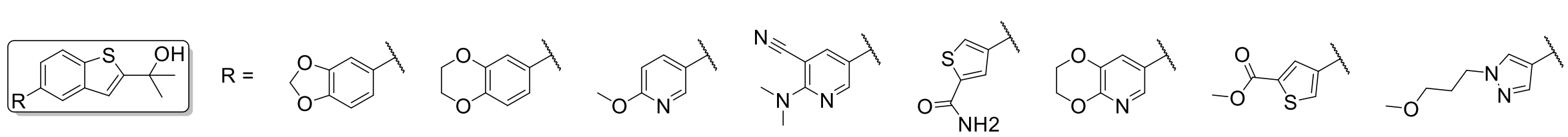

YKJ-166 YKJ-167 YKJ-168 YKJ-199 YKJ-202 YKJ-206 YKJ-211 YKJ-212

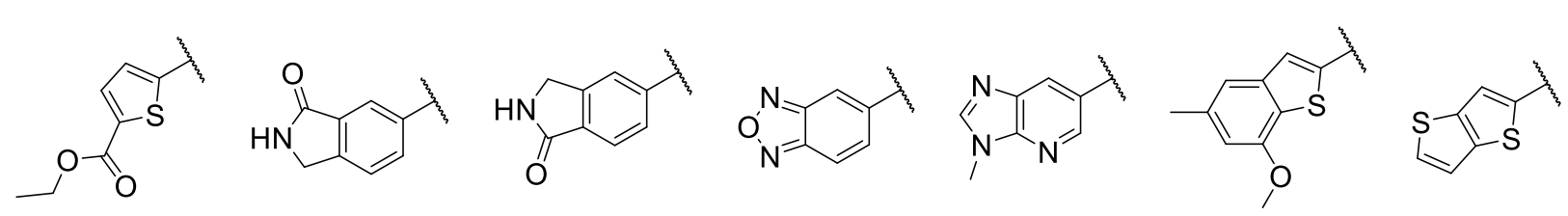

YKJ-213 YKJ-214 YKJ-215 YKJ-220 YKJ-221 YKJ-222 YKJ-223

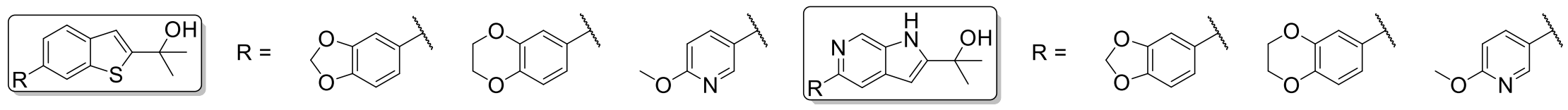

YKJ-172 YKJ-173 YKJ-174 YKJ-183 YKJ-184 YKJ-185

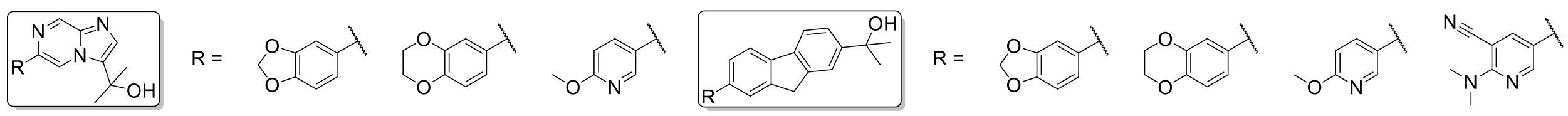

YKJ-196 YKJ-198 YKJ-197 YKJ-186 YKJ-188 YKJ-187 YKJ-201

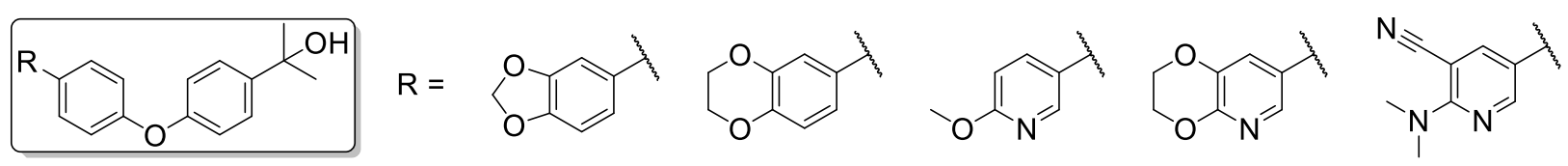

YKJ-208 YKJ-209 YKJ-210 YKJ-216 YKJ-217

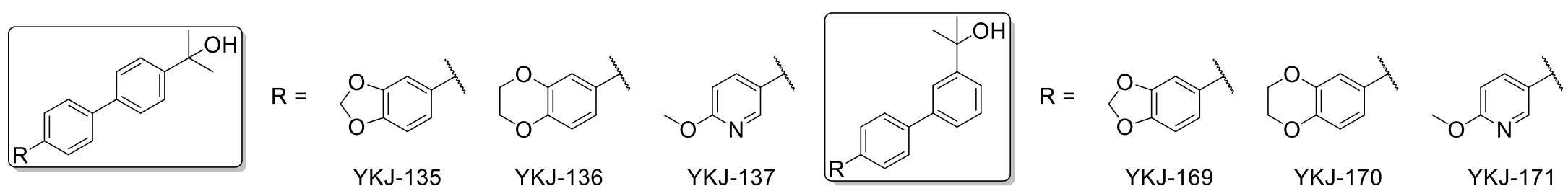

YKJ-135 YKJ-136 YKJ-137 YKJ-169 YKJ-170 YKJ-171

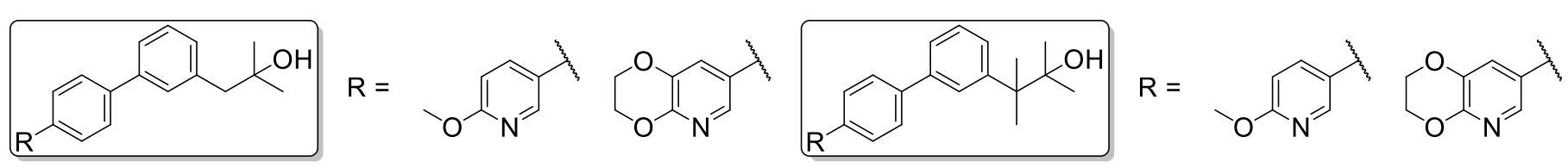

YKJ-255 YKJ-256 YKJ-257 YKJ-258

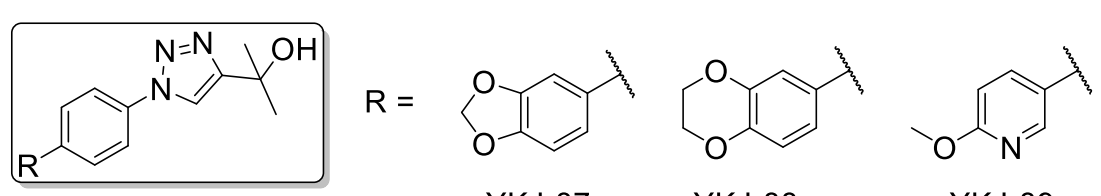

YKJ-87 YKJ-88 YKJ-89

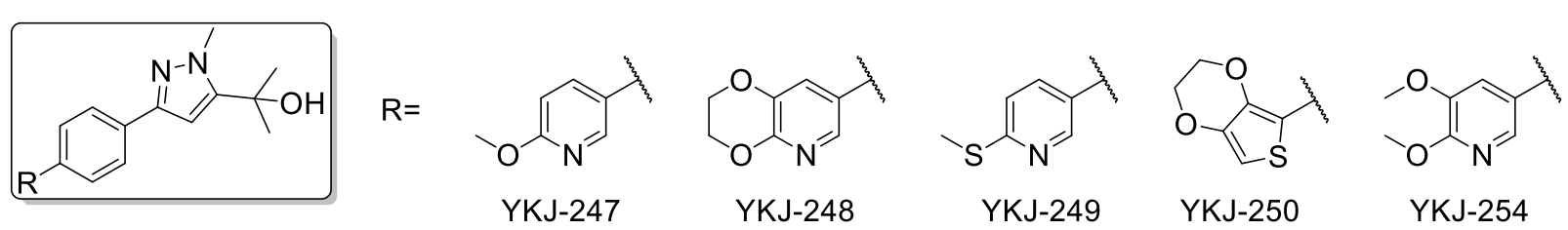

YKJ-247 YKJ-248 YKJ-249 YKJ-250 YKJ-254

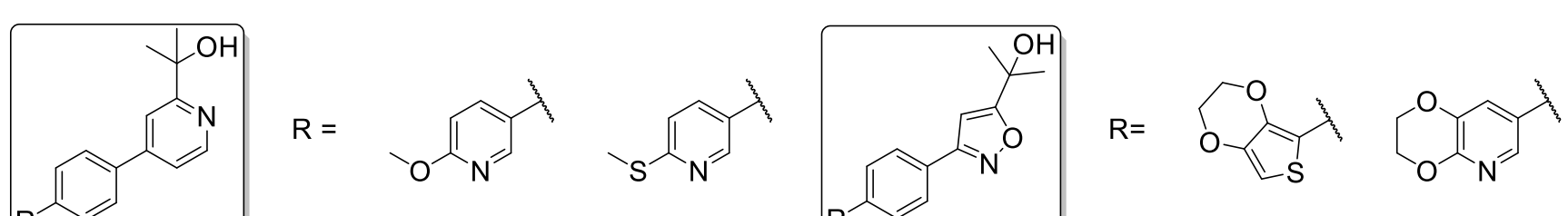

YKJ-251 YKJ-252 YKJ-263 YKJ-264

88 Quinoline Analogs

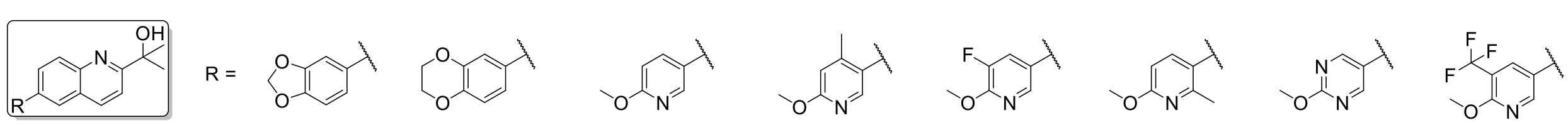

YKJ-84 YKJ-85 YKJ-86 YKJ-97 YKJ-98 YKJ-99 YKJ-100 YKJ-101

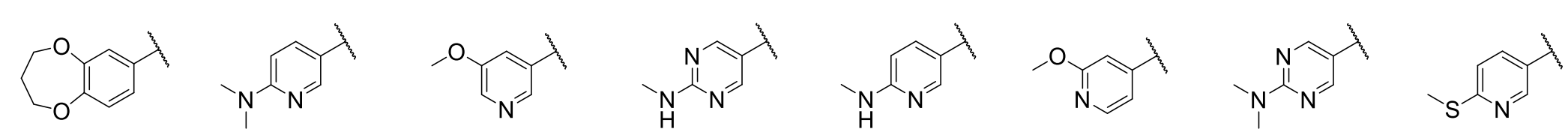

YKJ-102 YKJ-103 YKJ-104 YKJ-105 YKJ-106 YKJ-107 YKJ-108 YKJ-109

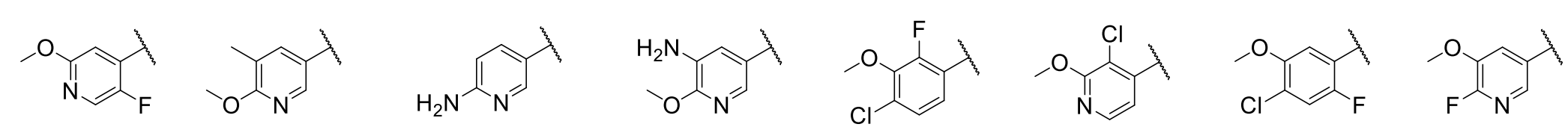

YKJ-110 YKJ-111 YKJ-112 YKJ-113 YKJ-114 YKJ-115 YKJ-116 YKJ-117

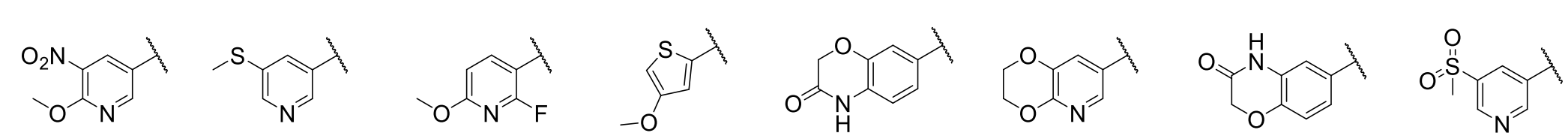

YKJ-118 YKJ-119 YKJ-120 YKJ-121 YKJ-122 YKJ-124 YKJ-125 YKJ-127

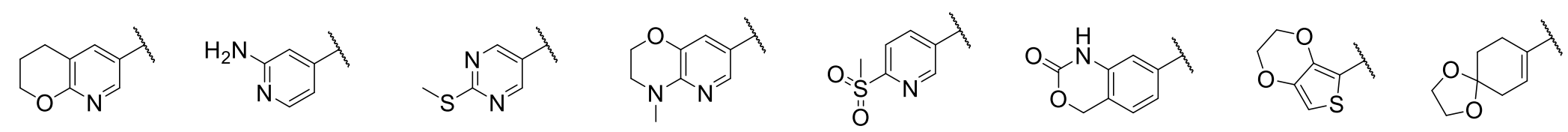

YKJ-128 YKJ-129 YKJ-131 YKJ-132 YKJ-133 YKJ-134 YKJ-143 YKJ-144

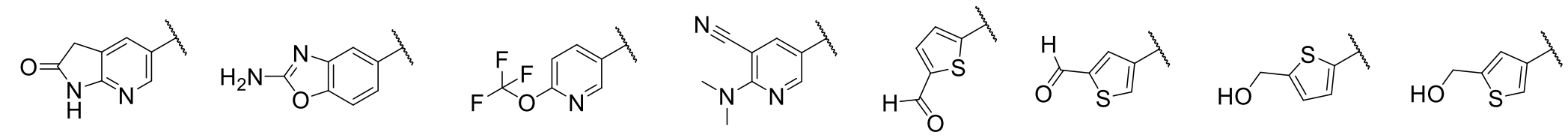

YKJ-146 YKJ-147 YKJ-148 YKJ-149 YKJ-150 YKJ-151 YKJ 152 YKJ 153

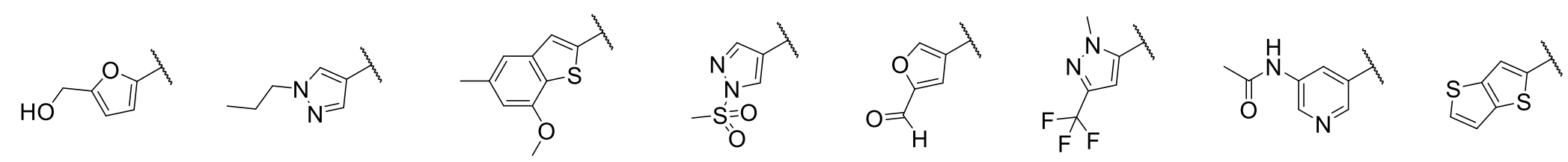

YKJ 154 YKJ 155 YKJ 156 YKJ 157 YKJ 158 YKJ 159 YKJ 175 YKJ-176

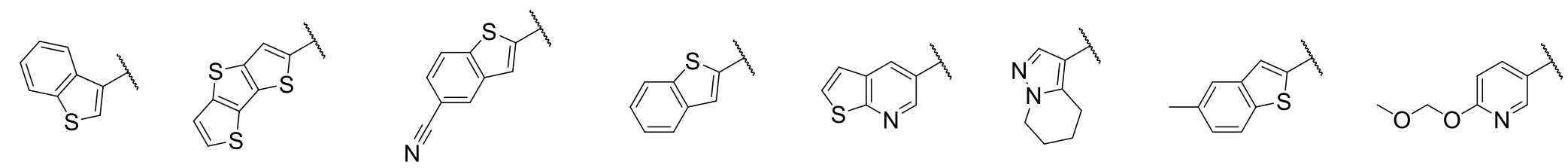

YKJ-227 YKJ-228 YKJ-229 YKJ-230 YKJ-232 YKJ-238 YKJ-239 YKJ-259

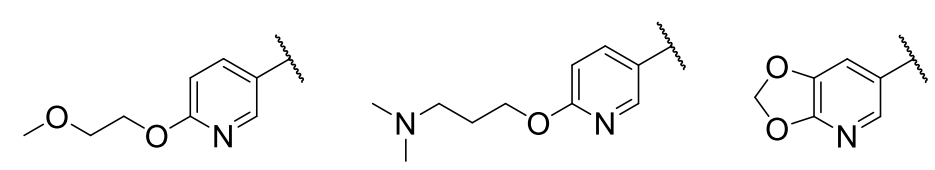

YKJ-260 YKJ-261 YKJ-262

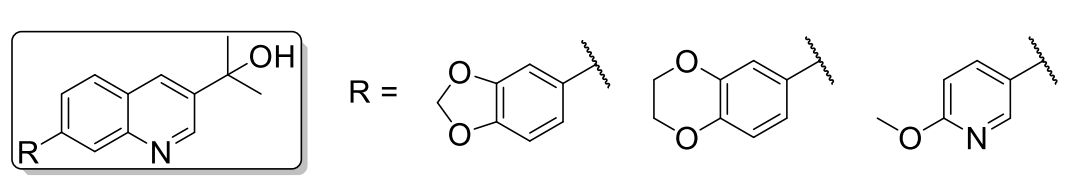

YKJ-81 YKJ-82 YKJ-83

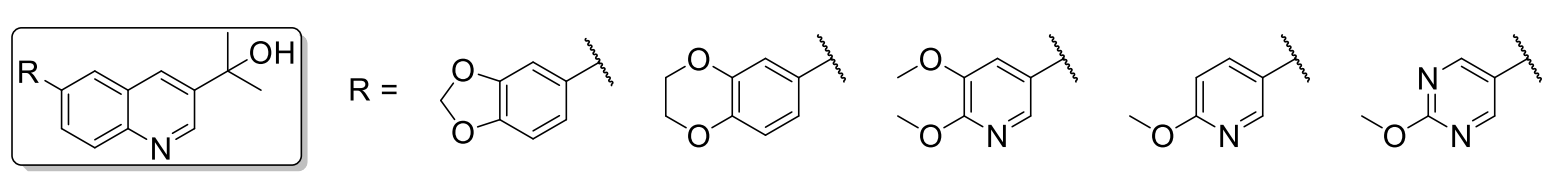

YKJ-92 YKJ-93 YKJ-94 YKJ-95 YKJ-96

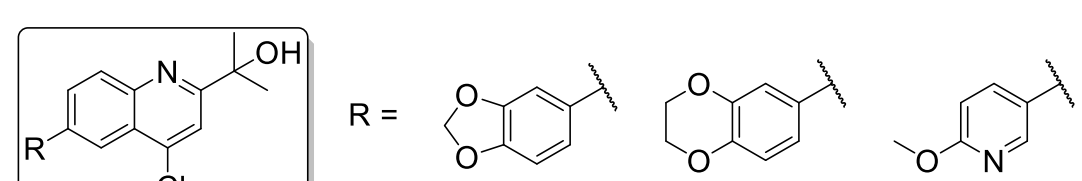

YKJ-192 YKJ-194 YKJ-195

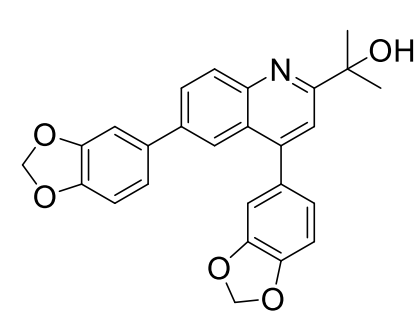

YKJ-193

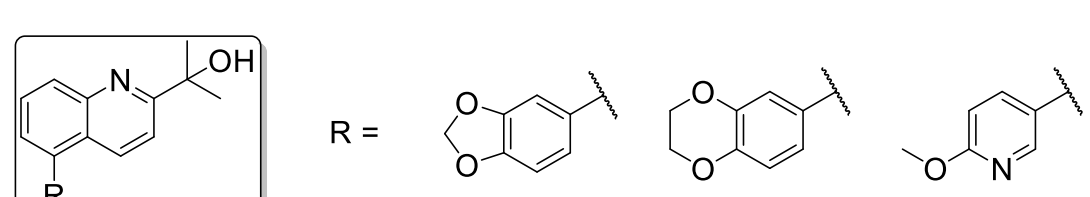

YKJ-189 YKJ-190 YKJ-191

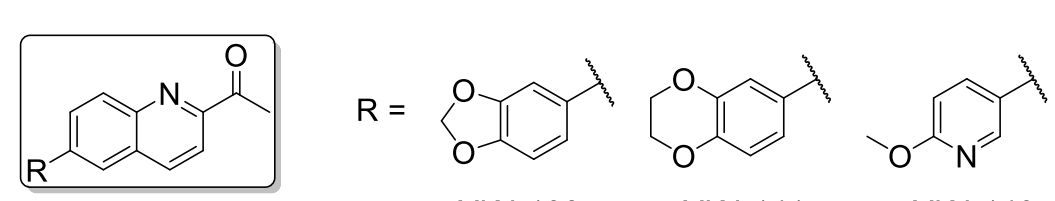

YKJ-138 YKJ-141 YKJ-140

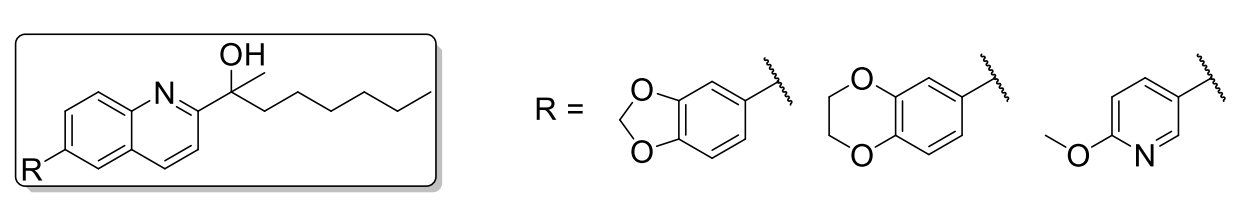

YKJ-139 YKJ-145 YKJ-142

77 Phenylthiazole Analogs

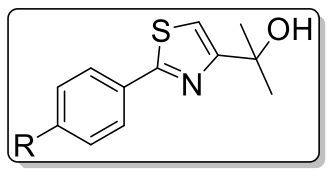

R =

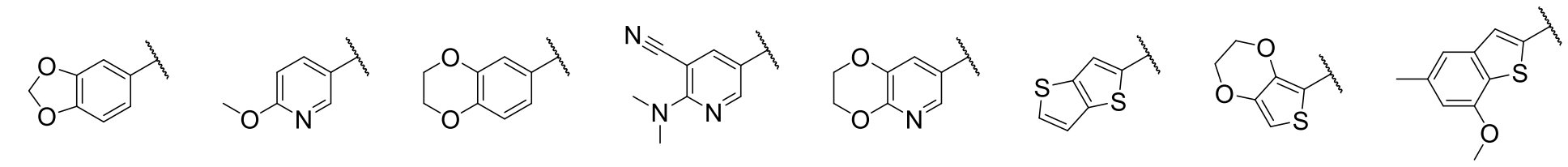

YKJ-180 YKJ-181 YKJ-182 YKJ-200 YKJ-226 YKJ-231 YKJ-233 YKJ-234

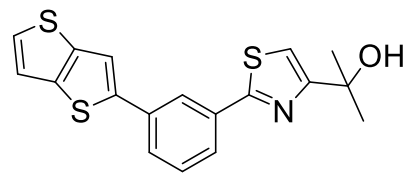

YKJ-240

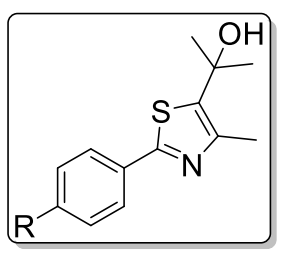

R =

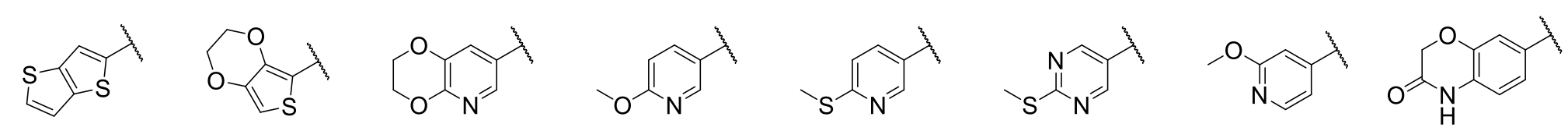

YKJ-235 YKJ-236 YKJ-237 YKJ-241 YKJ-242 YKJ-243 YKJ-244 YKJ-245

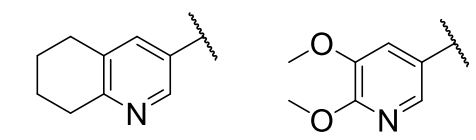

YKJ-246 YKJ-253

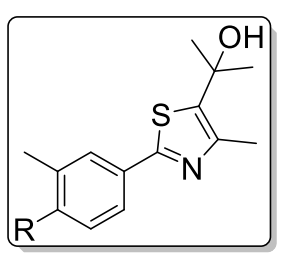

R =

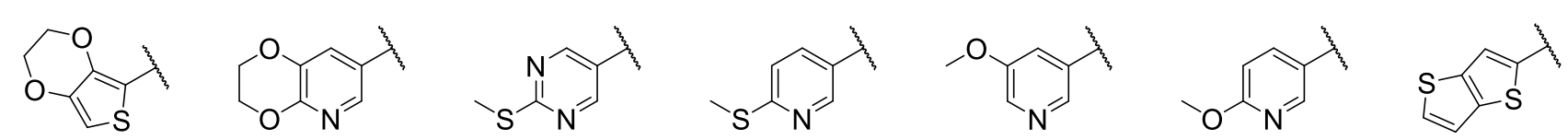

YKJ-267 YKJ-268 YKJ-269 YKJ-270 YKJ-271 YKJ-272 YKJ-273

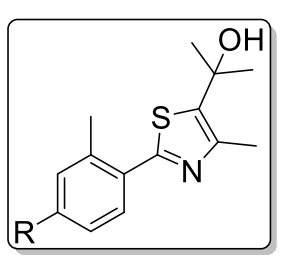

R =

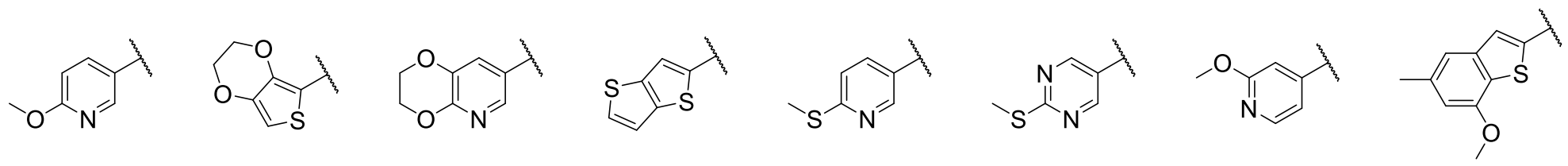

YKJ-274 YKJ-275 YKJ-276 YKJ-277 YKJ-278 YKJ-279 YKJ-280 YKJ-281

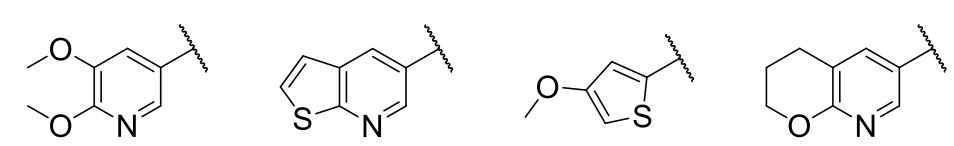

YKJ-283 YKJ-284 YKJ-286 YKJ-287

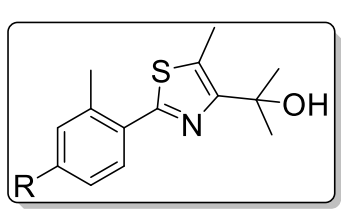

R =

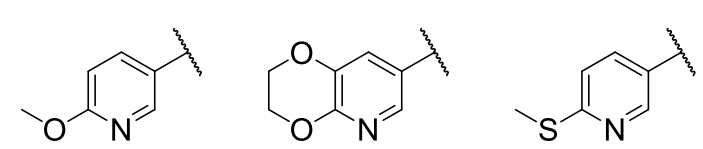

YKJ-301 YKJ-302 YKJ-303

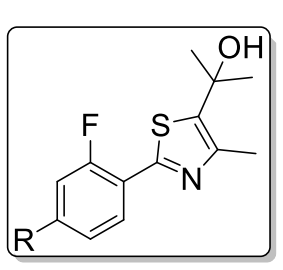

R =

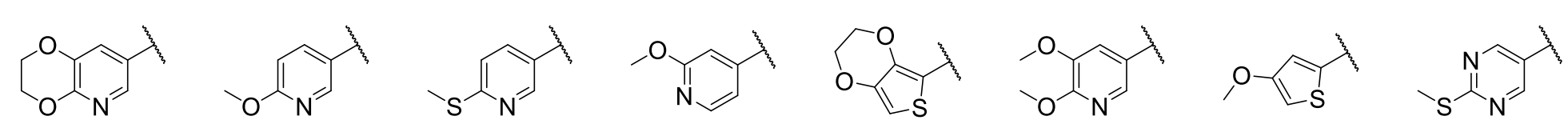

YKJ-282 YKJ-285 YKJ-288 YKJ-289 YKJ-297 YKJ-299 YKJ-316 YKJ-318

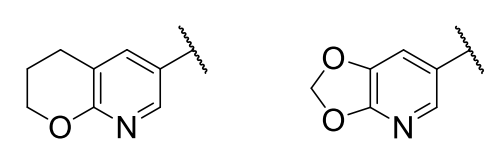

YKJ-319 YKJ-321

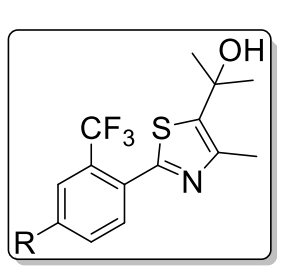

R =

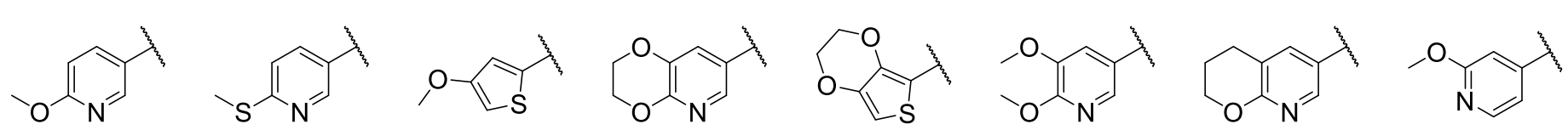

YKJ-290 YKJ-291 YKJ-292 YKJ-295 YKJ-298 YKJ-300 YKJ-310 YKJ-315

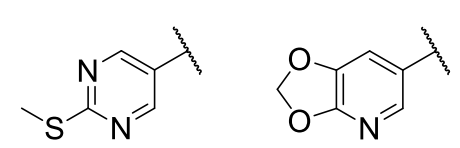

YKJ-317 YKJ-320

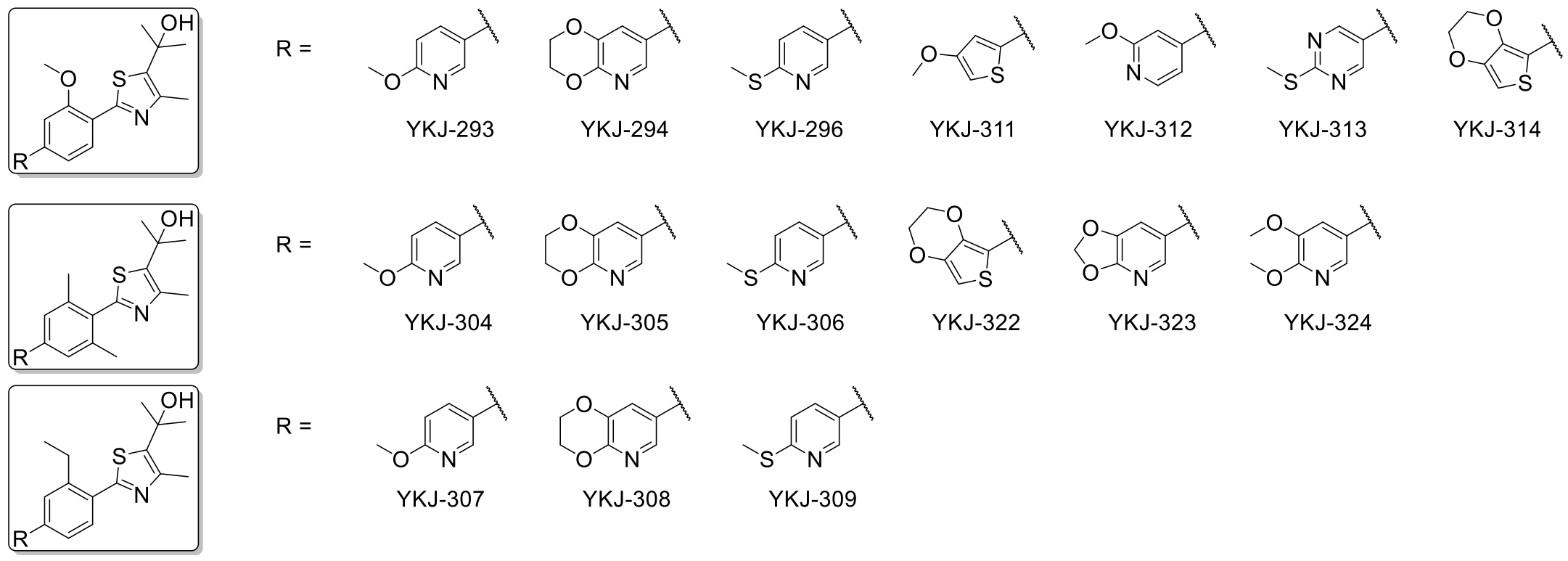

Preparation of YKJ Compounds

35 Naphthalene Analogs

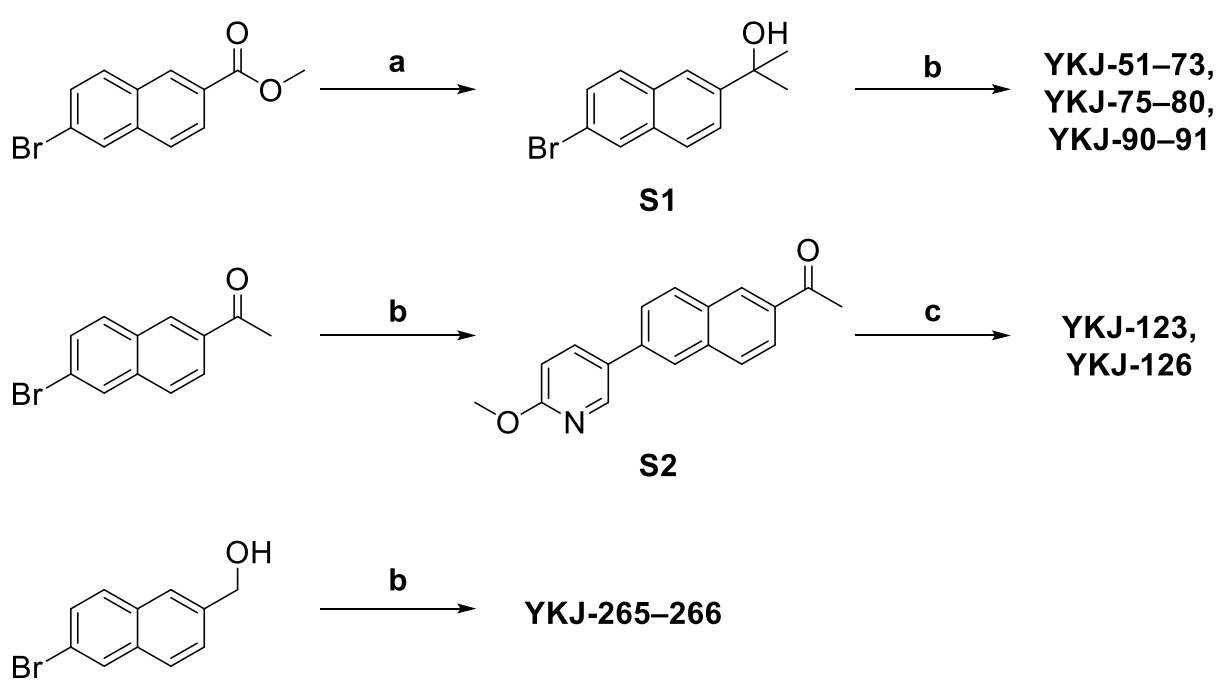

Reagents and conditions: a) MeMgBr, THF, 0 ℃ to rt, 2–4 h b) K_2CO_3, $Pd(PPh_3)_4$, dioxane/H_2O (5:1), 100 ℃, 3–5 h c) c) Mg, alkyl bromide, THF, reflux, 2 h; then ketone, 0 ℃ to rt, 12 h

General procedure for step a

A solution of the corresponding bromo-substituted ester (1.0 equiv) in anhydrous THF was added dropwise to a stirred solution of methylmagnesium bromide (3.0 equiv) in diethyl ether at 0 ℃ under an inert atmosphere. The reaction mixture was allowed to warm to room temperature and stirred for 2–4 h, while monitoring by TLC. The reaction was quenched with saturated aqueous NH_4Cl at 0 °C and diluted with ethyl acetate. The layers were separated, and the aqueous phase was extracted with ethyl acetate. The combined organic layers were dried over anhydrous $MgSO_4$, filtered, and concentrated under reduced pressure. The crude product was purified by flash column chromatography on silica gel (ethyl acetate/hexanes) to afford the corresponding tertiary alcohol.

General procedure for step b

The corresponding intermediate (1.0 equiv), boronic acid or boronic acid ester (1.1 equiv), K_2CO_3 (3.0 equiv), and $Pd(PPh_3)_4$ (0.1 equiv) were combined in 1,4-dioxane/H_2O (5:1) under an inert atmosphere. The reaction mixture was heated at 100 ℃ for 3–5 h with monitoring by TLC. After completion, the mixture was cooled to room temperature, diluted with ethyl acetate and brine, and the layers were separated. The aqueous phase was extracted with ethyl acetate. The combined organic layers were dried over anhydrous $MgSO_4$, filtered, and concentrated under reduced pressure. The crude product was purified by flash column chromatography on silica gel to afford the corresponding product.

Procedure for step c

Magnesium turnings (9 equiv) were suspended in anhydrous THF under an inert atmosphere, and the corresponding alkyl bromide (6 equiv) was added at room temperature. The reaction mixture was heated to reflux and stirred for 2 h to generate the Grignard reagent. The resulting Grignard reagent was cooled to 0 ℃ and added dropwise to a solution of the corresponding ketone (1.0 equiv) in anhydrous THF. The reaction mixture was allowed to warm to room temperature and stirred for 12 h. The reaction was quenched with saturated aqueous NH_4Cl, diluted with ethyl acetate, and the layers were separated. The aqueous phase was extracted with ethyl acetate. The combined organic layers were dried over anhydrous $MgSO_4$, filtered, and concentrated under reduced pressure. The crude product was purified by flash column chromatography on silica gel to afford the corresponding tertiary alcohol.

S1. 2-(6-bromonaphthalen-2-yl)propan-2-ol

Yield: 93.8%. ^{1}H NMR (500 MHz, DMSO) δ 8.14 (d, *J* = 2.0 Hz, 1H), 7.99 – 7.97 (m, 1H), 7.87 (d, *J* = 8.8 Hz, 1H), 7.84 (d, *J* = 8.7 Hz, 1H), 7.70 (dd, *J* = 8.7, 1.8 Hz, 1H), 7.59 (dd, *J* = 8.7, 2.0 Hz, 1H), 5.19 (s, 1H), 1.50 (s, 6H). ^{13}C NMR (126 MHz, DMSO) δ 148.85, 132.83, 131.26, 130.21, 129.16, 128.81, 126.54, 125.37, 122.29, 118.43, 70.80, 31.72. ESI–MS calcd for $C_{13}H_{12}Br$ $[M - OH]^+$ 247.0117; found 247.0116.

S2. 1-(6-(6-methoxypyridin-3-yl)naphthalen-2-yl)ethan-1-one

Yield: 78.3%. ^{1}H NMR (400 MHz, DMSO-d_6) δ 8.72 – 8.67 (m, 2H), 8.33 – 8.19 (m, 3H), 8.09 – 7.95 (m, 3H), 6.98 (d, J = 8.6 Hz, 1H), 3.93 (s, 3H), 2.71 (s, 3H). ^{13}C NMR (101 MHz, DMSO-d_6) δ 197.77, 163.44, 145.31, 137.88, 136.92, 135.43, 134.14, 131.32, 130.41, 130.14, 128.63, 128.48, 125.58, 124.51, 124.11, 110.80, 53.38, 26.74.

Broad scaffold diversification: 72 Analogs

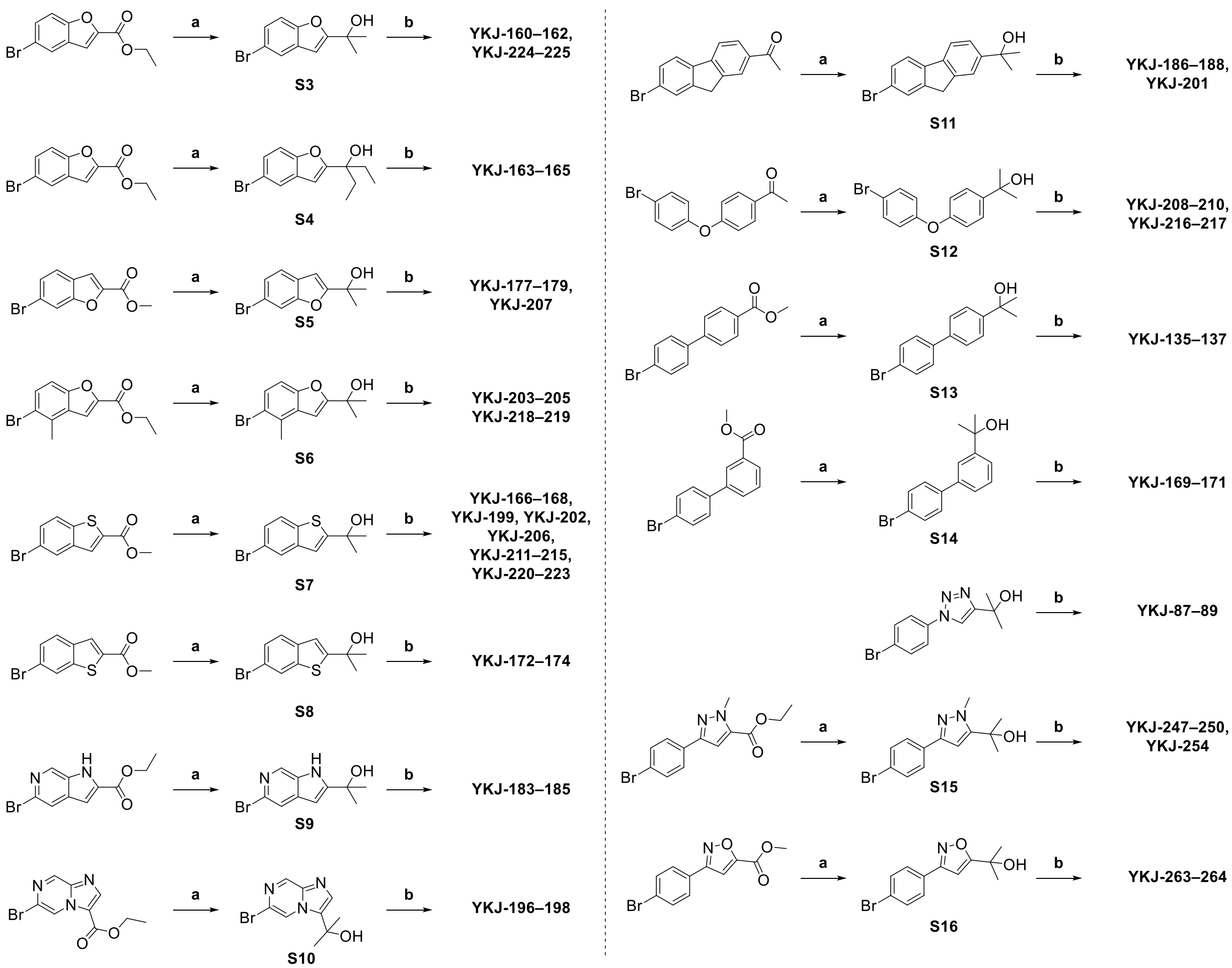

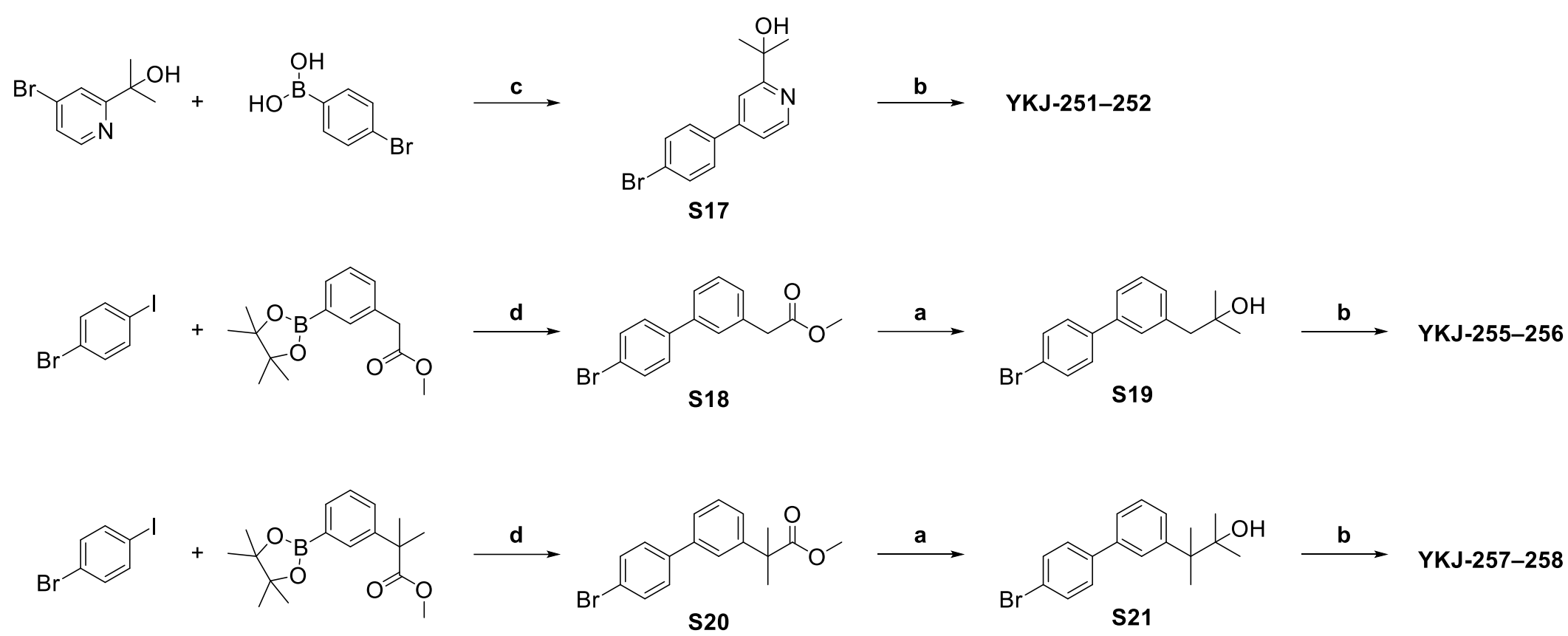

Reagents and conditions: a) MeMgBr, THF, 0 ℃ to rt, 2–4 h b) K_2CO_3, $Pd(PPh_3)_4$, dioxane/H_2O (5:1), 100 ℃, 3–5 h c) K_2CO_3, $Pd(PPh_3)_4$, dioxane/H_2O (5:1), 100 °C, 3 h d) K_2CO_3, $Pd(PPh_3)_4$, toluene/EtOH/H_2O (4:1:1), 100 °C, 3 h

General procedure for step a

A solution of the corresponding bromo-substituted ester (1.0 equiv) in anhydrous THF was added dropwise to a stirred solution of methylmagnesium bromide (3.0 equiv) in diethyl ether at 0 ℃ under an inert atmosphere. The reaction mixture was allowed to warm to room temperature and stirred for 2–4 h, while monitoring by TLC. The reaction was quenched with saturated aqueous NH_4Cl at 0 °C and diluted with ethyl acetate. The layers were separated, and the aqueous phase was extracted with ethyl acetate. The combined organic layers were dried over anhydrous $MgSO_4$, filtered, and concentrated under reduced pressure. The crude product was purified by flash column chromatography on silica gel (ethyl acetate/hexanes) to afford the corresponding tertiary alcohol.

General procedure for step b

The corresponding intermediate (1.0 equiv), boronic acid or boronic acid ester (1.1 equiv), K_2CO_3 (3.0 equiv), and $Pd(PPh_3)_4$ (0.1 equiv) were combined in 1,4-dioxane/H_2O (5:1) under an inert atmosphere. The reaction mixture was heated at 100 ℃ for 3–5 h with monitoring by TLC. After completion, the mixture was cooled to room temperature, diluted with ethyl acetate and brine, and the layers were separated. The aqueous phase was extracted with ethyl acetate. The combined organic layers were dried over anhydrous $MgSO_4$, filtered, and concentrated under reduced

pressure. The crude product was purified by flash column chromatography on silica gel to afford the corresponding product.

Procedure for step c

The corresponding aryl halide (2.5 equiv), boronic acid (1.0 equiv), K_2CO_3 (3.0 equiv), and $Pd(PPh_3)_4$ (0.2 equiv) were combined in 1,4-dioxane/H_2O (5:1) under an inert atmosphere. The reaction mixture was heated at 100 ℃ for 3 h with monitoring by TLC. After completion, the mixture was cooled to room temperature, diluted with ethyl acetate and brine, and the layers were separated. The aqueous phase was extracted with ethyl acetate. The combined organic layers were dried over anhydrous $MgSO_4$, filtered, and concentrated under reduced pressure. The crude product was purified by flash column chromatography on silica gel to afford the corresponding product.

Procedure for step d

The corresponding aryl halide (1.1 equiv), boronic acid ester (1.0 equiv), K_2CO_3 (3.0 equiv), and $Pd(PPh_3)_4$ (0.05 equiv) were combined in toluene/ethanol/water (4:1:1) under an inert atmosphere. The reaction mixture was heated at 100 ℃ for 3 h with monitoring by TLC. After completion, the mixture was cooled to room temperature, diluted with ethyl acetate and brine, and the layers were separated. The aqueous phase was extracted with ethyl acetate. The combined organic layers were dried over anhydrous $MgSO_4$, filtered, and concentrated under reduced pressure. The crude product was purified by flash column chromatography on silica gel to afford the corresponding product.

S3. 2-(5-Bromobenzofuran-2-yl)propan-2-ol

Yield: 96.9% ^{1}H NMR (400 MHz, DMSO) δ 7.79 (d, *J* = 2.1 Hz, 1H), 7.52 (d, *J* = 8.7 Hz, 1H), 7.38 (dd, *J* = 8.7, 2.1 Hz, 1H), 6.67 (d, *J* = 0.9 Hz, 1H), 5.46 (s, 1H), 1.51 (s, 6H). ^{13}C NMR (101 MHz, DMSO) δ 166.40, 152.81, 130.55, 126.21, 123.32, 114.88, 112.96, 99.79, 67.53, 28.88. ESI–MS calcd for $C_{11}H_{10}BrO$ [M - OH]$^+$ 236.9910; found 236.9916.

S4. 3-(5-Bromobenzofuran-2-yl)pentan-3-ol

Yield: 61.2% ^{1}H NMR (400 MHz, DMSO) δ 7.78 (d, *J* = 2.1 Hz, 1H), 7.50 (d, *J* = 8.6 Hz, 1H), 7.35 (dd, *J* = 8.7, 2.1 Hz, 1H), 6.69 (d, *J* = 1.0 Hz, 1H), 5.11 (s, 1H), 1.86 – 1.69 (m, 4H), 0.74 (t, *J* = 7.4 Hz, 6H). ^{13}C NMR (101 MHz, DMSO) δ 164.53, 152.96, 130.50, 125.89, 123.11, 114.89, 112.86, 102.35, 73.75, 31.88, 7.87. ESI–MS calcd for $C_{13}H_{14}BrO$ [M - OH]$^+$ 265.0223; found 265.0220.

S5. 2-(6-Bromobenzofuran-2-yl)propan-2-ol
Yield: 90.6% ^{1}H NMR (400 MHz, DMSO) δ 7.83 (s, 1H), 7.54 (d, *J* = 8.2 Hz, 1H), 7.37 (dd, *J* = 8.3, 1.7 Hz, 1H), 6.70 (d, *J* = 1.0 Hz, 1H), 5.46 (s, 1H), 1.50 (s, 6H). ^{13}C NMR (101 MHz, DMSO) δ 165.69, 154.38, 127.60, 125.80, 122.41, 115.94, 114.16, 100.15, 67.51, 28.89. ESI–MS calcd for $C_{11}H_{10}BrO$ [M - OH]$^+$ 236.9910; found 236.9908.

S6. 2-(5-Bromo-4-methylbenzofuran-2-yl)propan-2-ol
Yield: 93.6% ^{1}H NMR (400 MHz, DMSO) δ 7.41 (d, *J* = 8.7 Hz, 1H), 7.34 (d, *J* = 8.7 Hz, 1H), 6.82 (d, *J* = 0.9 Hz, 1H), 5.44 (s, 1H), 2.49 (s, 3H), 1.52 (s, 6H). ^{13}C NMR (101 MHz, DMSO) δ 165.55, 152.52, 129.82, 129.65, 127.00, 117.06, 110.44, 99.54, 67.52, 28.92, 18.89. ESI–MS calcd for $C_{12}H_{12}BrO$ [M - OH]$^+$ 251.0066; found 251.0067.

S7. 2-(5-Bromobenzo[I]thiophen-2-yl)propan-2-ol
Yield: 95.1% ^{1}H NMR (400 MHz, DMSO) δ 7.96 (d, *J* = 2.0 Hz, 1H), 7.85 (d, *J* = 8.5 Hz, 1H), 7.40 (dd, *J* = 8.5, 2.0 Hz, 1H), 7.20 (s, 1H), 5.70 (s, 1H), 1.56 (s, 6H). ^{13}C NMR (101 MHz, DMSO) δ 159.71, 141.70, 137.34, 126.17, 125.45, 124.27, 117.25, 117.04, 69.94, 31.98. ESI–MS calcd for $C_{11}H_{10}BrS$ [M - OH]$^+$ 252.9681; found 252.9680.

S8. 2-(6-Bromobenzo[*b*]thiophen-2-yl)propan-2-ol
Yield: 81.4% ^{1}H NMR (400 MHz, DMSO) δ 8.17 (d, *J* = 1.8 Hz, 1H), 7.68 (d, *J* = 8.5 Hz, 1H), 7.46 (dd, *J* = 8.5, 1.9 Hz, 1H), 7.21 (d, *J* = 0.8 Hz, 1H), 5.70 (s, 1H), 1.55 (s, 6H). ^{13}C NMR (101 MHz, DMSO) δ 158.50, 140.25, 138.89, 127.17, 124.80, 124.66, 117.36, 116.38, 69.92, 31.97. ESI–MS calcd for $C_{11}H_{10}BrS$ [M - OH]$^+$ 252.9681; found 252.9682.

S9. 2-(5-Bromo-1*H*-pyrrolo[2,3-*c*]pyridin-2-yl)propan-2-ol

Yield: 64.4% ^{1}H NMR (400 MHz, DMSO) δ 11.60 (s, 1H), 8.40 (s, 1H), 7.63 (s, 1H), 6.29 (d, *J* = 1.9 Hz, 1H), 5.49 (s, 1H), 1.52 (s, 6H). ^{13}C NMR (101 MHz, DMSO) δ 154.70, 135.94, 133.51, 132.84, 128.74, 116.97, 95.20, 67.88, 30.53. ESI–MS calcd for $C_{10}H_{12}BrN_2O$ [M + H]$^+$ 255.0128; found 255.0132.

S10. 2-(6-Bromoimidazo[1,2-*a*]pyrazin-3-yl)propan-2-ol

Yield: 56.8% ^{1}H NMR (400 MHz, DMSO) δ 8.95 (d, *J* = 1.3 Hz, 1H), 8.86 (d, *J* = 1.4 Hz, 1H), 7.76 (s, 1H), 5.67 (s, 1H), 1.62 (s, 6H). ^{13}C NMR (101 MHz, DMSO) δ 142.78, 139.89, 133.44, 133.14, 121.75, 119.85, 67.24, 28.89. ESI–MS calcd for $C_9H_{11}BrN_3O$ [M + H]$^+$ 256.0080; found 256.0076.

S11. 2-(7-Bromo-9*H*-fluoren-2-yl)propan-2-ol

Yield: 79.6% ^{1}H NMR (400 MHz, DMSO) δ 7.82 (d, *J* = 3.2 Hz, 1H), 7.80 (d, *J* = 3.3 Hz, 1H), 7.76 (d, *J* = 1.9 Hz, 1H), 7.69 (d, *J* = 1.6 Hz, 1H), 7.54 (dd, *J* = 8.2, 1.9 Hz, 1H), 7.49 (dd, *J* = 8.0, 1.7 Hz, 1H), 5.06 (s, 1H), 3.92 (s, 2H), 1.47 (s, 6H). ^{13}C NMR (101 MHz, DMSO) δ 150.11, 145.70, 142.52, 140.43, 137.85, 129.55, 128.09, 123.47, 121.52, 121.36, 119.53, 119.29, 70.88, 36.42, 32.10. ESI–MS calcd for $C_{16}H_{14}Br$ [M - OH]$^+$ 285.0273; found 285.0273.

S12. 2-(4-(4-Bromophenoxy)phenyl)propan-2-ol

Yield: 82.4% ^{1}H NMR (400 MHz, DMSO) δ 7.58 – 7.44 (m, 4H), 7.01 – 6.89 (m, 4H), 5.02 (s, 1H), 1.42 (s, 6H). ^{13}C NMR (101 MHz, DMSO) δ 156.62, 153.99, 146.38, 132.71, 126.34, 120.18, 118.36, 114.61, 70.40, 31.97. ESI–MS calcd for $C_{15}H_{14}BrO$ [M - OH]$^+$ 289.0223; found 289.0222.

S13. 2-(4'-bromo-[1,1'-biphenyl]-4-yl)propan-2-ol

Yield: 87.8% ^{1}H NMR (400 MHz, DMSO) δ 7.66 – 7.52 (m, 8H), 5.05 (s, 1H), 1.45 (s, 6H). ^{13}C NMR (101 MHz, DMSO) δ 150.24, 139.31, 136.46, 131.72, 128.62, 125.98, 125.24, 120.56, 70.53, 31.86. ESI–MS calcd for $C_{15}H_{14}Br$ [M - OH]$^+$ 273.0273; found 273.0273

S14. 2-(4'-Bromo-[1,1'-biphenyl]-3-yl)propan-2-ol

Yield: 96.6% ^{1}H NMR (400 MHz, DMSO) δ 7.74 (t, *J* = 1.9 Hz, 1H), 7.67 – 7.60 (m, 4H), 7.50 – 7.45 (m, 2H), 7.41 – 7.36 (m, 1H), 5.08 (s, 1H), 1.47 (s, 6H). ^{13}C NMR (101 MHz, DMSO) δ 151.39, 139.86, 138.35, 131.74, 128.82, 128.48, 124.22, 124.16, 122.83, 120.69, 70.73, 31.92. ESI–MS calcd for $C_{15}H_{14}Br$ [M - OH]$^{+}$ 273.0273 found 273.0273.

S15. 2-(3-(4-bromophenyl)-1-methyl-1H-pyrazol-5-yl)propan-2-ol
Yield: quantitative ^{1}H NMR (500 MHz, DMSO) δ 7.73 – 7.68 (m, 2H), 7.58 – 7.53 (m, 2H), 6.58 (s, 1H), 5.34 (s, 1H), 3.99 (s, 3H), 1.53 (s, 6H). ^{13}C NMR (126 MHz, DMSO) δ 150.67, 146.44, 132.79, 131.50, 126.82, 120.11, 100.57, 66.91, 38.80, 30.20. ESI–MS calcd for $C_{13}H_{16}BrN_2O$ [M + H]$^{+}$ 295.0441; found 295.0443.

S16. 2-(3-(4-bromophenyl)isoxazol-5-yl)propan-2-ol
Yield: 91.7% ^{1}H NMR (500 MHz, DMSO-d_6) δ 7.86 – 7.79 (m, 2H), 7.74 – 7.68 (m, 2H), 6.90 (s, 1H), 5.69 (s, 1H), 1.51 (s, 6H). ^{13}C NMR (126 MHz, DMSO-d_6) δ 180.10, 160.68, 132.13, 128.58, 128.05, 123.50, 97.75, 67.40, 28.99.

S17. 2-(4-(4-bromophenyl)pyridin-2-yl)propan-2-ol
Yield: 39.0% ^{1}H NMR (500 MHz, DMSO-d_6) δ 8.55 (d, *J* = 5.1 Hz, 1H), 7.94 – 7.92 (m, 1H), 7.73 (s, 4H), 7.54 (dd, *J* = 5.1, 1.5 Hz, 1H), 5.30 (s, 1H), 1.48 (s, 6H). ^{13}C NMR (126 MHz, DMSO-d_6) δ 169.06, 148.85, 146.24, 137.08, 132.15, 128.93, 122.67, 118.83, 115.74, 72.56, 30.69.

S18. methyl 2-(4'-bromo-[1,1'-biphenyl]-3-yl)acetate
Yield: 46.6 % ^{1}H NMR (500 MHz, Chloroform-*d*) δ 7.58 – 7.54 (m, 2H), 7.48 – 7.43 (m, 4H), 7.42 – 7.38 (m, 1H), 7.31 – 7.27 (m, 1H), 3.71 (s, 3H), 3.69 (s, 2H). ^{13}C NMR (126 MHz, Chloroform-*d*) δ 172.05, 140.53, 139.96, 134.78, 132.01, 129.31, 128.95, 128.71, 128.09, 125.95, 121.82, 52.29, 41.32.

S19. 1-(4'-bromo-[1,1'-biphenyl]-3-yl)-2-methylpropan-2-ol
Yield: 84.2 % ^{1}H NMR (500 MHz, DMSO-d_6) δ 7.67 – 7.57 (m, 4H), 7.50 – 7.45 (m, 2H), 7.35 (t, *J* = 7.7 Hz, 1H), 7.25 – 7.20 (m, 1H), 4.35 (s, 1H), 2.72 (s, 2H), 1.08 (s, 6H). ^{13}C NMR (126

MHz, DMSO-d_6) δ 139.71, 139.70, 138.15, 131.78, 129.99, 128.79, 128.76, 128.26, 124.12, 120.70, 69.30, 49.31, 29.26.

S20. methyl 2-(4'-bromo-[1,1'-biphenyl]-3-yl)-2-methylpropanoate
Yield: 37.6 % ^{1}H NMR (500 MHz, DMSO-d_6) δ 7.68 – 7.58 (m, 4H), 7.56 – 7.50 (m, 2H), 7.44 (t, J = 7.7 Hz, 1H), 7.34 – 7.29 (m, 1H), 3.60 (s, 3H), 1.56 (s, 6H). ^{13}C NMR (126 MHz, DMSO-d_6) δ 176.33, 145.42, 139.42, 139.10, 131.84, 129.17, 129.00, 125.15, 125.13, 123.76, 121.02, 52.13, 46.26, 26.38.

S21. 3-(4'-bromo-[1,1'-biphenyl]-3-yl)-2,3-dimethylbutan-2-ol
Yield: 75.1 % ^{1}H NMR (500 MHz, DMSO-d_6) δ 7.67 – 7.62 (m, 3H), 7.60 – 7.55 (m, 2H), 7.46 – 7.40 (m, 2H), 7.38 – 7.33 (m, 1H), 4.18 (s, 1H), 1.36 (s, 6H), 1.01 (s, 6H). ^{13}C NMR (126 MHz, DMSO-d_6) δ 148.19, 140.26, 137.53, 131.76, 128.92, 127.74, 127.59, 126.73, 123.72, 120.57, 72.81, 44.74, 26.19, 24.44.

88 Quinoline Analogs

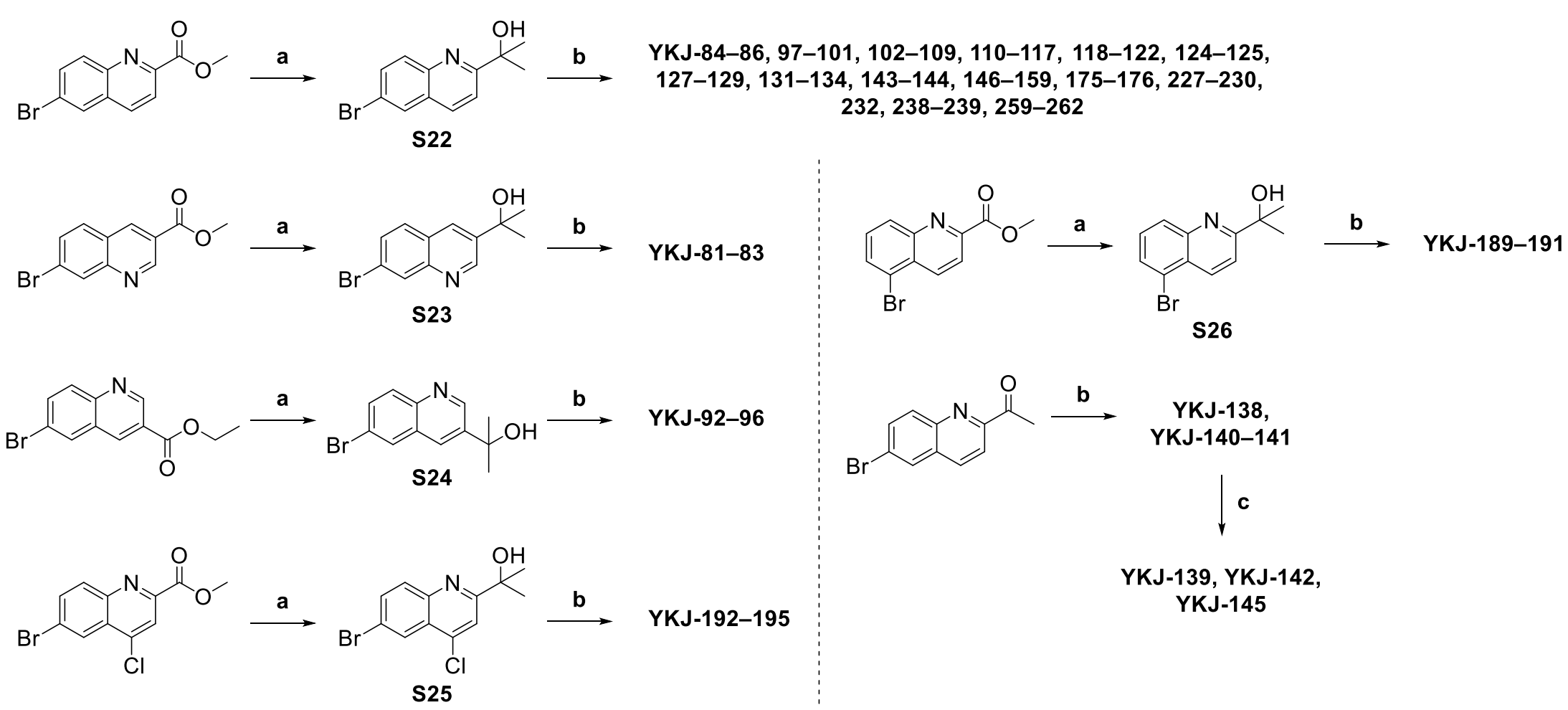

Reagents and conditions: a) MeMgBr, THF, 0 ℃ to rt, 2–4 h b) K_2CO_3, $Pd(PPh_3)_4$, dioxane/H_2O (5:1), 100 ℃, 3–5 h c) hexylmagnesium bromide (2.0 M in Et_2O), THF, 0 ℃ to rt, 12 h

General procedure for step a

A solution of the corresponding bromo-substituted ester (1.0 equiv) in anhydrous THF was added dropwise to a stirred solution of methylmagnesium bromide (3.0 equiv) in diethyl ether at 0 ℃ under an inert atmosphere. The reaction mixture was allowed to warm to room temperature and stirred for 2–4 h, while monitoring by TLC. The reaction was quenched with saturated aqueous NH_4Cl at 0 °C and diluted with ethyl acetate. The layers were separated, and the aqueous phase was extracted with ethyl acetate. The combined organic layers were dried over anhydrous $MgSO_4$, filtered, and concentrated under reduced pressure. The crude product was purified by flash column chromatography on silica gel (ethyl acetate/hexanes) to afford the corresponding tertiary alcohol.

General procedure for step b

The corresponding intermediate (1.0 equiv), boronic acid or boronic acid ester (1.1 equiv), K_2CO_3 (3.0 equiv), and $Pd(PPh_3)_4$ (0.1 equiv) were combined in 1,4-dioxane/H_2O (5:1) under an inert atmosphere. The reaction mixture was heated at 100 ℃ for 3–5 h with monitoring by TLC. After completion, the mixture was cooled to room temperature, diluted with ethyl acetate and brine, and the layers were separated. The aqueous phase was extracted with ethyl acetate. The combined organic layers were dried over anhydrous $MgSO_4$, filtered, and concentrated under reduced pressure. The crude product was purified by flash column chromatography on silica gel to afford the corresponding product.

Procedure for step c

A solution of the corresponding ketone (1.0 equiv) in anhydrous THF was cooled to 0 ℃ under an inert atmosphere. Hexylmagnesium bromide (2.0 M in diethyl ether, 4 equiv) was added dropwise, and the reaction mixture was stirred at room temperature for 12 h. The reaction was quenched with saturated aqueous NH_4Cl, diluted with ethyl acetate, and the layers were separated. The aqueous phase was extracted with ethyl acetate. The combined organic layers were dried over anhydrous $MgSO_4$, filtered, and concentrated under reduced pressure. The crude product was purified by flash column chromatography on silica gel to afford the corresponding tertiary alcohol.

S22. 2-(6-Bromoquinolin-2-yl)propan-2-ol

Yield: 75.6% ^{1}H NMR (400 MHz, DMSO) δ 8.32 (dd, *J* = 8.8, 0.8 Hz, 1H), 8.23 (d, *J* = 2.3 Hz, 1H), 7.93 – 7.88 (m, 2H), 7.82 (dd, *J* = 9.0, 2.3 Hz, 1H), 5.45 (s, 1H), 1.53 (s, 6H). ^{13}C NMR

(101 MHz, DMSO) δ 169.00, 144.85, 135.68, 132.35, 130.84, 129.63, 127.91, 118.80, 118.72, 72.69, 30.33. ESI–MS calcd for $C_{12}H_{13}BrNO$ [M + H]$^+$ 266.0175; 266.0174.

S23. 2-(7-Bromoquinolin-3-yl)propan-2-ol

Yield: 67.2% ^{1}H NMR (400 MHz, DMSO) δ 9.09 (d, *J* = 2.3 Hz, 1H), 8.38 (dd, *J* = 2.3, 0.9 Hz, 1H), 8.20 (d, *J* = 2.0 Hz, 1H), 7.97 (d, *J* = 8.7 Hz, 1H), 7.73 (dd, *J* = 8.7, 2.0 Hz, 1H), 5.41 (s, 1H), 1.55 (s, 6H). ^{13}C NMR (101 MHz, DMSO) δ 150.43, 146.85, 143.56, 130.43, 130.40, 130.16, 129.66, 126.10, 121.88, 69.89, 31.43. ESI–MS calcd for $C_{12}H_{13}BrNO$ [M + H]$^+$ 266.0175; found 266.0176.

S24. 2-(6-Bromoquinolin-3-yl)propan-2-ol

Yield: 77.4% ^{1}H NMR (400 MHz, DMSO) δ 9.08 (d, *J* = 2.3 Hz, 1H), 8.33 (dd, *J* = 2.3, 0.8 Hz, 1H), 8.27 (d, *J* = 2.2 Hz, 1H), 7.93 (d, *J* = 8.9 Hz, 1H), 7.82 (dd, *J* = 8.9, 2.3 Hz, 1H), 5.42 (s, 1H), 1.55 (s, 6H). ^{13}C NMR (101 MHz, DMSO) δ 149.90, 144.83, 143.92, 131.87, 130.70, 130.03, 129.53, 128.69, 119.46, 69.87, 31.45. ESI–MS calcd for $C_{12}H_{13}BrNO$ [M + H]$^+$ 266.0175; 266.0177

S25. 2-(6-Bromo-4-chloroquinolin-2-yl)propan-2-ol

Yield: 43.2% ^{1}H NMR (400 MHz, DMSO) δ 8.31 – 8.28 (m, 1H), 8.04 (s, 1H), 7.98 – 7.95 (m, 2H), 5.58 (s, 1H), 1.53 (s, 6H). ^{13}C NMR (101 MHz, DMSO) δ 169.67, 146.04, 140.45, 133.82, 131.56, 125.47, 125.39, 120.68, 118.87, 72.74, 30.13. ESI–MS calcd for $C_{12}H_{12}BrClNO$ [M + H]$^+$ 299.9785; found 299.9785.

S26. 2-(5-Bromoquinolin-2-yl)propan-2-ol

Yield: 79.9% ^{1}H NMR (400 MHz, DMSO) δ 8.41 (d, *J* = 8.6 Hz, 1H), 8.11 (dd, *J* = 7.5, 1.3 Hz, 1H), 7.99 (dd, *J* = 8.2, 1.3 Hz, 1H), 7.94 (d, *J* = 8.6 Hz, 1H), 7.48 (t, *J* = 7.8 Hz, 1H), 5.52 (s, 1H), 1.57 (s, 6H). ^{13}C NMR (101 MHz, DMSO) δ 169.24, 142.73, 137.29, 132.93, 127.98, 127.88, 126.82, 123.89, 118.76, 72.84, 30.45. ESI–MS calcd for $C_{12}H_{13}BrNO$ [M + H]$^+$ 266.0175; found 266.0177.

77 Phenylthiazole Analogs

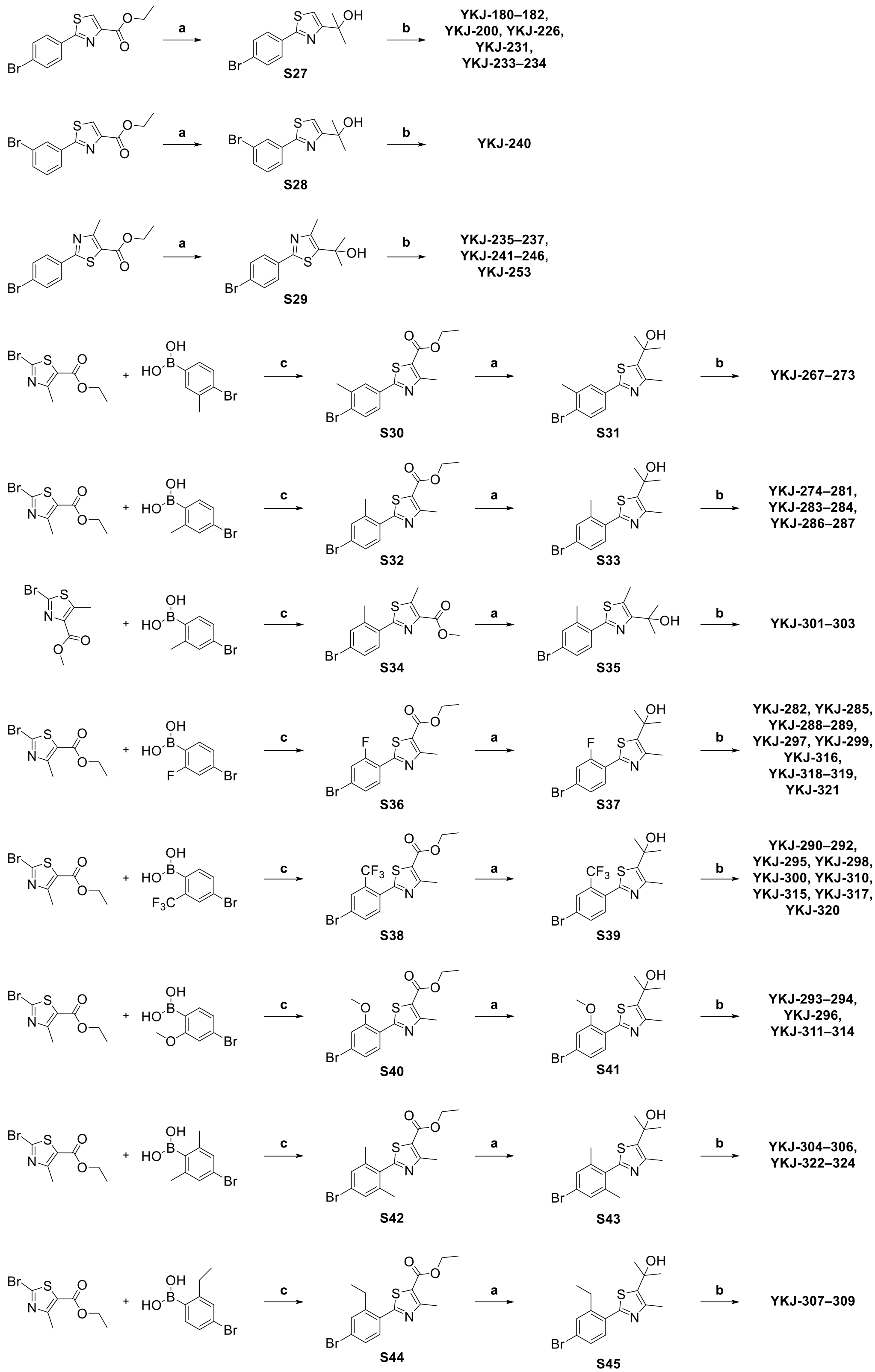

Reagents and conditions: a) MeMgBr, THF, 0 °C to rt, 2–4 h b) K_2CO_3, $Pd(PPh_3)_4$, dioxane/H_2O (5:1), 100 °C, 3–5 h c) K_2CO_3, $Pd(PPh_3)_4$, dioxane/H_2O (10:1), 100 °C, 16 h

General procedure for step a

A solution of the corresponding bromo-substituted ester (1.0 equiv) in anhydrous THF was added dropwise to a stirred solution of methylmagnesium bromide (3.0 equiv) in diethyl ether at 0 °C under an inert atmosphere. The reaction mixture was allowed to warm to room temperature and stirred for 2–4 h, while monitoring by TLC. The reaction was quenched with saturated aqueous NH_4Cl at 0 °C and diluted with ethyl acetate. The layers were separated, and the aqueous phase was extracted with ethyl acetate. The combined organic layers were dried over anhydrous $MgSO_4$, filtered, and concentrated under reduced pressure. The crude product was purified by flash column chromatography on silica gel (ethyl acetate/hexanes) to afford the corresponding tertiary alcohol.

General procedure for step b

The corresponding intermediate (1.0 equiv), boronic acid or boronic acid ester (1.1 equiv), K_2CO_3 (3.0 equiv), and $Pd(PPh_3)_4$ (0.1 equiv) were combined in 1,4-dioxane/H_2O (5:1) under an inert atmosphere. The reaction mixture was heated at 100 °C for 3–5 h with monitoring by TLC. After completion, the mixture was cooled to room temperature, diluted with ethyl acetate and brine, and the layers were separated. The aqueous phase was extracted with ethyl acetate. The combined organic layers were dried over anhydrous $MgSO_4$, filtered, and concentrated under reduced pressure. The crude product was purified by flash column chromatography on silica gel to afford the corresponding product.

Procedure for step c

The corresponding aryl bromide (1.0 equiv), boronic acid (0.8–1.4 equiv), K_2CO_3 (2.5 equiv), and $Pd(PPh_3)_4$ (0.08 equiv) were combined in 1,4-dioxane/H_2O (10:1) under an inert atmosphere. The reaction mixture was heated at 100 °C for 16 h with monitoring by TLC. After completion, the mixture was cooled to room temperature, diluted with ethyl acetate and brine, and the layers were separated. The aqueous phase was extracted with ethyl acetate. The combined organic layers were dried over anhydrous $MgSO_4$, filtered, and concentrated under reduced pressure. The crude

product was purified by flash column chromatography on silica gel to afford the corresponding bromo-substituted ester.

S27. 2-(2-(4-Bromophenyl)thiazol-4-yl)propan-2-ol

Yield: 84.4% ^{1}H NMR (400 MHz, DMSO) δ 7.89 – 7.84 (m, 2H), 7.71 – 7.67 (m, 2H), 7.45 (s, 1H), 5.23 (s, 1H), 1.50 (s, 6H). ^{13}C NMR (101 MHz, DMSO) δ 166.50, 164.99, 132.49, 132.15, 127.90, 123.22, 113.34, 70.43, 30.56. ESI–MS calcd for $C_{12}H_{11}BrNS$ [M - OH]$^{+}$ 279.9790; found 279.9792.

S28. 2-(2-(3-Bromophenyl)thiazol-4-yl)propan-2-ol

Yield: 86.8% ^{1}H NMR (500 MHz, DMSO) δ 8.08 (t, J = 1.8 Hz, 1H), 7.90 (ddd, J = 7.8, 1.7, 1.0 Hz, 1H), 7.67 (ddd, J = 8.0, 2.0, 1.0 Hz, 1H), 7.48 (s, 1H), 7.45 (t, J = 7.9 Hz, 1H), 5.24 (s, 1H), 1.50 (s, 6H). ^{13}C NMR (126 MHz, DMSO) δ 166.50, 164.33, 135.38, 132.61, 131.42, 128.06, 125.18, 122.39, 113.76, 70.42, 30.55. ESI–MS calcd for $C_{12}H_{13}BrNOS$ [M + H]$^{+}$ 297.9896; found 297.9897.

S29. 2-(2-(4-Bromophenyl)-4-methylthiazol-5-yl)propan-2-ol

Yield: 89.8% ^{1}H NMR (500 MHz, DMSO) δ 7.82 – 7.75 (m, 2H), 7.67 – 7.61 (m, 2H), 5.73 (s, 1H), 2.44 (s, 3H), 1.55 (s, 6H). ^{13}C NMR (126 MHz, DMSO) δ 159.79, 146.15, 143.87, 132.59, 132.08, 127.41, 122.73, 69.28, 30.93, 16.53. ESI–MS calcd for $C_{13}H_{15}BrNOS$ [M + H]$^{+}$ 312.0052; found 312.0052.

S30. ethyl 2-(4-bromo-3-methylphenyl)-4-methylthiazole-5-carboxylate

Yield: 39.8% ^{1}H NMR (500 MHz, DMSO-d_6) δ 7.99 – 7.95 (m, 1H), 7.76 – 7.68 (m, 2H), 4.30 (q, J = 7.0 Hz, 2H), 2.68 (s, 3H), 2.42 (s, 3H), 1.30 (t, J = 7.1 Hz, 3H). ^{13}C NMR (126 MHz, DMSO-d_6) δ 167.89, 161.35, 160.33, 138.69, 133.11, 131.48, 128.76, 127.48, 125.66, 121.59, 61.28, 22.30, 17.19, 14.13. ESI–MS calcd for $C_{14}H_{15}BrNO_2S$ [M + H]$^{+}$ 340.0001 found 340.0009

S31. 2-(2-(4-bromo-3-methylphenyl)-4-methylthiazol-5-yl)propan-2-ol

Yield: 83.3% ^{1}H NMR (500 MHz, DMSO-d_6) δ 7.83 (d, J = 2.3 Hz, 1H), 7.64 (d, J = 8.3 Hz, 1H), 7.58 (dd, J = 8.3, 2.3 Hz, 1H), 5.71 (s, 1H), 2.44 (s, 3H), 2.40 (s, 3H), 1.55 (s, 6H). ^{13}C NMR (126 MHz, DMSO-d_6) δ 159.92, 146.14, 143.62, 138.20, 132.85, 132.82, 127.73, 125.27, 124.75, 69.24, 30.98, 22.36, 16.52. ESI–MS calcd for $C_{14}H_{17}BrNOS$ $[M + H]^+$ 326.0209 found 326.0209

S32. ethyl 2-(4-bromo-2-methylphenyl)-4-methylthiazole-5-carboxylate
Yield: 48.8% ^{1}H NMR (500 MHz, DMSO-d_6) δ 7.80 (d, J = 8.4 Hz, 1H), 7.66 (d, J = 1.6 Hz, 1H), 7.55 (dd, J = 8.4, 1.7 Hz, 1H), 4.31 (q, J = 7.1 Hz, 2H), 2.70 (s, 3H), 2.56 (s, 3H), 1.31 (t, J = 7.1 Hz, 3H). ^{13}C NMR (126 MHz, DMSO-d_6) δ 167.44, 161.40, 159.49, 138.93, 134.25, 131.44, 130.72, 129.48, 123.97, 122.15, 61.27, 21.01, 17.21, 14.16. ESI–MS calcd for $C_{14}H_{15}BrNO_2S$ $[M + H]^+$ 340.0001 found 340.0007

S33. 2-(2-(4-bromo-2-methylphenyl)-4-methylthiazol-5-yl)propan-2-ol
Yield: 89.8% ^{1}H NMR (500 MHz, DMSO-d_6) δ 7.66 (d, J = 8.4 Hz, 1H), 7.58 (d, J = 1.7 Hz, 1H), 7.48 (dd, J = 8.4, 1.8 Hz, 1H), 5.70 (s, 1H), 2.53 (s, 3H), 2.46 (s, 3H), 1.56 (s, 6H). ^{13}C NMR (126 MHz, DMSO-d_6) δ 159.60, 145.46, 143.80, 137.95, 133.90, 131.91, 130.83, 129.16, 122.11, 69.28, 31.04, 21.01, 16.55. ESI–MS calcd for $C_{14}H_{17}BrNOS$ $[M + H]^+$ 326.0209 found 326.0212

S34. methyl 2-(4-bromo-2-methylphenyl)-5-methylthiazole-4-carboxylate
Yield: 50.0% ^{1}H NMR (500 MHz, DMSO-d_6) δ 7.69 – 7.62 (m, 2H), 7.57 – 7.51 (m, 1H), 3.84 (s, 3H), 2.76 (s, 3H), 2.53 (s, 3H). ^{13}C NMR (126 MHz, DMSO-d_6) δ 162.35, 161.19, 145.41, 141.09, 138.50, 134.05, 131.26, 130.96, 129.38, 123.17, 51.88, 20.73, 12.68. ESI–MS calcd for $C_{13}H_{13}BrNO_2S$ $[M + H]^+$ 325.9845 found 325.9860

S35. 2-(2-(4-Bromo-2-methylphenyl)-5-methylthiazol-4-yl)propan-2-ol
Yield: 85.0% ^{1}H NMR (500 MHz, DMSO-d_6) δ 7.64 – 7.56 (m, 2H), 7.49 (dd, J = 8.4, 2.1 Hz, 1H), 5.07 (s, 1H), 2.61 (s, 3H), 2.53 (s, 3H), 1.53 (s, 6H). ^{13}C NMR (126 MHz, DMSO-d_6) δ 159.04, 158.12, 138.07, 134.03, 131.73, 130.79, 129.20, 128.48, 122.09, 72.09, 31.21, 21.00, 12.03. ESI–MS calcd for $C_{14}H_{17}BrNOS$ $[M + H]^+$ 326.0209 found 326.0209

S36. ethyl 2-(4-bromo-2-fluorophenyl)-4-methylthiazole-5-carboxylate

Yield: 43.5% ^{1}H NMR (500 MHz, DMSO-d_6) δ 8.18 (t, *J* = 8.3 Hz, 1H), 7.86 (dd, *J* = 11.0, 1.9 Hz, 1H), 7.62 (dd, *J* = 8.5, 1.9 Hz, 1H), 4.31 (q, *J* = 7.1 Hz, 2H), 2.71 (s, 3H), 1.31 (t, *J* = 7.1 Hz, 3H). ^{13}C NMR (126 MHz, DMSO-d_6) δ 161.37, 160.13 (d, *J* = 5.7 Hz), 159.37 (d, *J* = 257.0 Hz), 159.23, 129.87 (d, *J* = 2.9 Hz), 128.77 (d, *J* = 3.2 Hz), 124.95 (d, *J* = 10.2 Hz), 122.66 (d, *J* = 8.8 Hz), 120.06 (d, *J* = 25.0 Hz), 119.28 (d, *J* = 11.4 Hz), 61.38, 17.10, 14.14. ESI–MS calcd for $C_{13}H_{12}BrFNO_2S$ $[M + H]^+$ 343.9751 found 343.9760

S37. 2-(2-(4-bromo-2-fluorophenyl)-4-methylthiazol-5-yl)propan-2-ol

Yield: 86.4% ^{1}H NMR (500 MHz, DMSO-d_6) δ 8.08 (t, *J* = 8.4 Hz, 1H), 7.75 (dd, *J* = 11.0, 2.0 Hz, 1H), 7.54 (dd, *J* = 8.5, 2.0 Hz, 1H), 5.77 (s, 1H), 2.47 (s, 3H), 1.56 (s, 6H). ^{13}C NMR (126 MHz, DMSO-d_6) δ 158.62 (d, *J* = 254.0 Hz), 152.50 (d, *J* = 5.3 Hz), 145.30, 145.07 (d, *J* = 7.3 Hz), 129.08 (d, *J* = 3.4 Hz), 128.36 (d, *J* = 3.3 Hz), 122.46 (d, *J* = 9.8 Hz), 120.37 (d, *J* = 11.9 Hz), 119.71 (d, *J* = 25.2 Hz), 69.35, 30.80, 16.42. ESI–MS calcd for $C_{13}H_{14}BrFNOS$ $[M + H]^+$ 329.9958 found 329.9966

S38. ethyl 2-(4-bromo-2-(trifluoromethyl)phenyl)-4-methylthiazole-5-carboxylate

Yield: 32.9% ^{1}H NMR (500 MHz, DMSO-d_6) δ 8.15 (d, *J* = 2.0 Hz, 1H), 8.06 (dd, *J* = 8.2, 2.0 Hz, 1H), 7.75 (d, *J* = 8.3 Hz, 1H), 4.32 (q, *J* = 7.1 Hz, 2H), 2.69 (s, 3H), 1.30 (t, *J* = 7.1 Hz, 3H). ^{13}C NMR (126 MHz, DMSO-d_6) δ 164.77, 161.14, 159.43, 135.87, 134.30, 130.23 (d, *J* = 1.9 Hz), 129.93 (q, *J* = 5.5 Hz), 128.82 (q, *J* = 31.7 Hz), 124.36, 123.90, 122.57 (q, *J* = 274.7 Hz), 61.49, 17.08, 14.10. ESI–MS calcd for $C_{14}H_{12}BrF_3NO_2S$ $[M + H]^+$ 393.9719 found 393.9730

S39. 2-(2-(4-bromo-2-(trifluoromethyl)phenyl)-4-methylthiazol-5-yl)propan-2-ol

Yield: 84.3% ^{1}H NMR (500 MHz, DMSO-d_6) δ 8.06 (d, *J* = 2.0 Hz, 1H), 7.98 (dd, *J* = 8.3, 2.1 Hz, 1H), 7.65 (d, *J* = 8.3 Hz, 1H), 5.79 (s, 1H), 2.44 (s, 3H), 1.56 (s, 6H). ^{13}C NMR (126 MHz, DMSO-d_6) δ 156.77, 145.75, 145.62, 135.62, 134.14, 131.75 (d, *J* = 1.7 Hz), 129.63 (q, *J* = 5.7 Hz), 128.42 (q, *J* = 31.4 Hz), 122.89, 122.75 (q, *J* = 274.7 Hz), 69.32, 30.90, 16.40. ESI–MS calcd for $C_{14}H_{14}BrF_3NOS$ $[M + H]^+$ 379.9926 found 379.9936

S40. ethyl 2-(4-bromo-2-methoxyphenyl)-4-methylthiazole-5-carboxylate

Yield: 46.3% ^{1}H NMR (500 MHz, DMSO-d_6) δ 8.22 (d, *J* = 8.4 Hz, 1H), 7.51 (d, *J* = 1.8 Hz, 1H), 7.33 (dd, *J* = 8.4, 1.8 Hz, 1H), 4.30 (q, *J* = 7.1 Hz, 2H), 4.08 (s, 3H), 2.68 (s, 3H), 1.31 (t, *J* = 7.1 Hz, 3H). ^{13}C NMR (126 MHz, DMSO-d_6) δ 161.99, 161.92, 158.51, 157.16, 129.27, 125.37, 124.16, 121.77, 119.84, 115.69, 60.97, 56.74, 17.22, 14.23. ESI–MS calcd for $C_{14}H_{15}BrNO_3S$ [M + H]$^+$ 355.9951 found 355.9958

S41. 2-(2-(4-bromo-2-methoxyphenyl)-4-methylthiazol-5-yl)propan-2-ol

Yield: 84.2% ^{1}H NMR (500 MHz, DMSO-d_6) δ 8.11 (d, *J* = 8.4 Hz, 1H), 7.41 (d, *J* = 1.9 Hz, 1H), 7.25 (dd, *J* = 8.4, 1.9 Hz, 1H), 5.60 (s, 1H), 4.00 (s, 3H), 2.45 (s, 3H), 1.54 (s, 6H). ^{13}C NMR (126 MHz, DMSO-d_6) δ 156.20, 154.38, 144.35, 143.60, 128.32, 123.84, 122.83, 121.17, 115.37, 69.31, 56.39, 31.07, 16.58. ESI–MS calcd for $C_{14}H_{17}BrNO_2S$ [M + H]$^+$ 342.0158 found 342.0158

S42. ethyl 2-(4-bromo-2,6-dimethylphenyl)-4-methylthiazole-5-carboxylate

Yield: 31.4% ^{1}H NMR (500 MHz, DMSO-d_6) δ 7.45 (s, 2H), 4.31 (q, *J* = 7.1 Hz, 2H), 2.71 (s, 3H), 2.09 (s, 6H), 1.30 (t, *J* = 7.1 Hz, 3H). ^{13}C NMR (126 MHz, DMSO-d_6) δ 166.99, 161.33, 159.64, 139.52, 131.77, 130.26, 123.19, 122.98, 61.34, 19.49, 17.15, 14.15. ESI–MS calcd for $C_{15}H_{17}BrNO_2S$ [M + H]$^+$ 354.0158 found 354.0146

S43. 2-(2-(4-Bromo-2,6-dimethylphenyl)-4-methylthiazol-5-yl)propan-2-ol

Yield: 95.9% ^{1}H NMR (500 MHz, DMSO-d_6) δ 7.39 (s, 2H), 5.68 (s, 1H), 2.45 (s, 3H), 2.08 (s, 6H), 1.56 (s, 6H). ^{13}C NMR (126 MHz, DMSO-d_6) δ 158.70, 145.03, 143.88, 139.70, 133.08, 129.99, 122.06, 69.27, 31.14, 19.67, 16.42. ESI–MS calcd for $C_{15}H_{19}BrNOS$ [M + H]$^+$ 340.0365 found 340.0370

S44. Ethyl 2-(4-bromo-2-ethylphenyl)-4-methylthiazole-5-carboxylate

Yield: 62.5% ^{1}H NMR (500 MHz, DMSO-d_6) δ 7.66 – 7.63 (m, 2H), 7.54 (dd, *J* = 8.4, 2.1 Hz, 1H), 4.31 (q, *J* = 7.0 Hz, 2H), 2.92 (q, *J* = 7.5 Hz, 2H), 2.70 (s, 3H), 1.30 (t, *J* = 7.1 Hz, 3H), 1.14 (t, *J* = 7.5 Hz, 3H). ^{13}C NMR (126 MHz, DMSO-d_6) δ 167.71, 161.35, 159.61, 145.10,

132.76, 132.10, 130.29, 129.45, 124.24, 122.30, 61.29, 26.03, 17.25, 15.27, 14.14. ESI–MS calcd for $C_{15}H_{17}BrNO_2S$ $[M + H]^+$ 354.0158 found 354.0149

S45. 2-(2-(4-Bromo-2-ethylphenyl)-4-methylthiazol-5-yl)propan-2-ol

Yield: 68.4% ^{1}H NMR (500 MHz, DMSO-d_6) δ 7.57 (d, *J* = 2.1 Hz, 1H), 7.54 – 7.45 (m, 2H), 5.70 (s, 1H), 2.91 (q, *J* = 7.5 Hz, 2H), 2.45 (s, 3H), 1.56 (s, 6H), 1.14 (t, *J* = 7.5 Hz, 3H). ^{13}C NMR (126 MHz, DMSO-d_6) δ 159.65, 145.49, 144.40, 143.83, 132.42, 131.58, 131.52, 129.12, 122.54, 69.27, 31.05, 26.02, 16.58, 15.39. ESI–MS calcd for $C_{15}H_{19}BrNOS$ $[M + H]^+$ 340.0365 found 340.0365

Spectral Data for YKJ compounds

(Total: 272 Compounds; YKJ-74 and YKJ-130 Not Included)

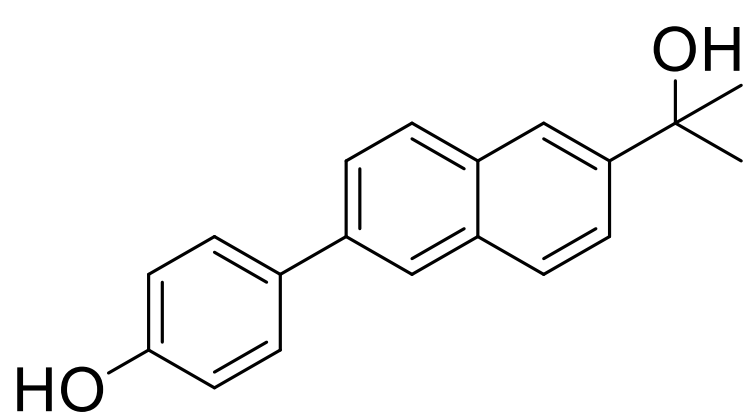

YKJ-51

4-(6-(2-Hydroxypropan-2-yl)naphthalen-2-yl)phenol

Yield: 78.7% ^{1}H NMR (400 MHz, DMSO) δ 9.60 (s, 1H), 8.05 (d, *J* = 1.9 Hz, 1H), 7.96 – 7.84 (m, 3H), 7.74 (dd, *J* = 8.5, 1.9 Hz, 1H), 7.63 (d, *J* = 8.7 Hz, 3H), 7.63 (d, *J* = 8.7 Hz, 3H), 6.89 (d, *J* = 8.6 Hz, 2H), 5.15 (s, 1H), 1.52 (s, 6H). ^{13}C NMR (101 MHz, DMSO) δ 157.17, 147.76, 136.96, 132.06, 131.45, 130.76, 128.43, 127.93, 127.43, 124.73, 124.47, 123.46, 121.89, 115.82, 70.80, 31.82. ESI–MS calcd for $C_{19}H_{17}O$ $[M - OH]^+$ 261.1274; found 261.1275.

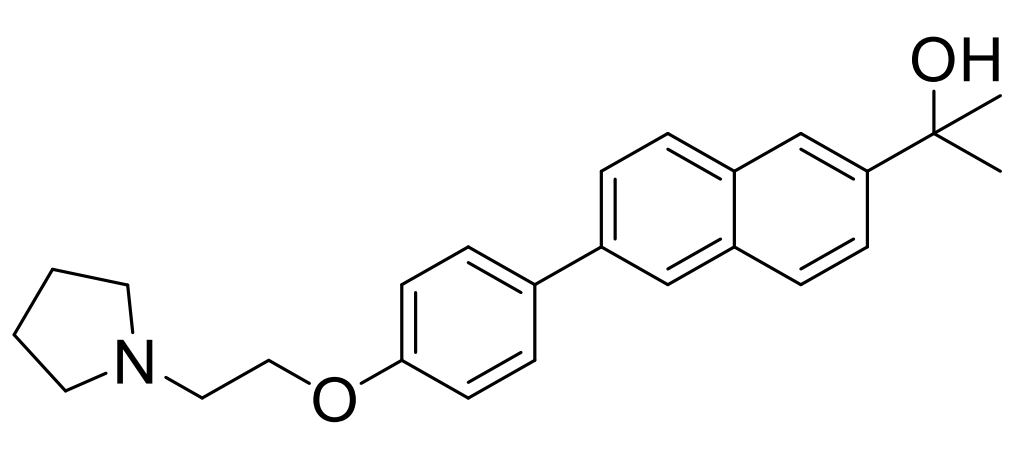

YKJ-52

2-(6-(4-(2-(Pyrrolidin-1-yl)ethoxy)phenyl)naphthalen-2-yl)propan-2-ol

Yield: 69.9% ^{1}H NMR (400 MHz, DMSO) δ 8.11 (d, *J* = 1.9 Hz, 1H), 7.94 (d, *J* = 8.5 Hz, 2H), 7.89 (d, *J* = 8.7 Hz, 1H), 7.82 – 7.73 (m, 3H), 7.65 (dd, *J* = 8.6, 1.8 Hz, 1H), 7.14 – 7.07 (m, 2H), 5.16 (s, 1H), 4.26 (t, *J* = 5.5 Hz, 2H), 3.23 – 3.10 (m, 2H), 2.92 (m, 4H), 1.82 (m, 4H), 1.52 (s, 6H). ^{13}C NMR (101 MHz, DMSO) δ 157.73, 148.00, 136.46, 132.75, 132.01, 131.64, 128.53, 127.98, 127.50, 124.75, 124.55, 123.90, 121.91, 115.11, 70.79, 53.88, 53.57, 31.81, 22.89 (one high field carbon not observed). ESI–MS calcd for $C_{25}H_{30}NO_2$ $[M + H]^+$ 376.2271; found 376.2274.

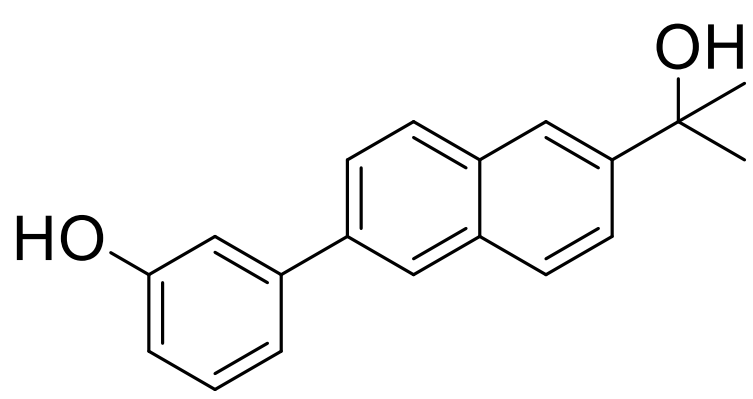

YKJ-53

3-(6-(2-Hydroxypropan-2-yl)naphthalen-2-yl)phenol

Yield: quantitative ^{1}H NMR (400 MHz, DMSO) δ 9.55 (s, 1H), 8.09 (s, 1H), 7.99 – 7.89 (m, 3H), 7.74 (dd, *J* = 8.4, 1.9 Hz, 1H), 7.70 – 7.63 (m, 1H), 7.29 (t, *J* = 7.8 Hz, 1H), 7.24 – 7.13 (m, 2H), 6.83 – 6.76 (m, 1H), 5.17 (s, 1H), 1.52 (s, 6H). ^{13}C NMR (101 MHz, DMSO) δ 157.88, 148.27, 141.53, 137.06, 131.99, 131.90, 129.99, 128.53, 127.68, 124.95, 124.64, 124.58, 121.90, 117.67, 114.44, 113.68, 70.82, 31.81. ESI–MS calcd for $C_{19}H_{17}O$ $[M - OH]^+$ 261.1274; found 261.1278.

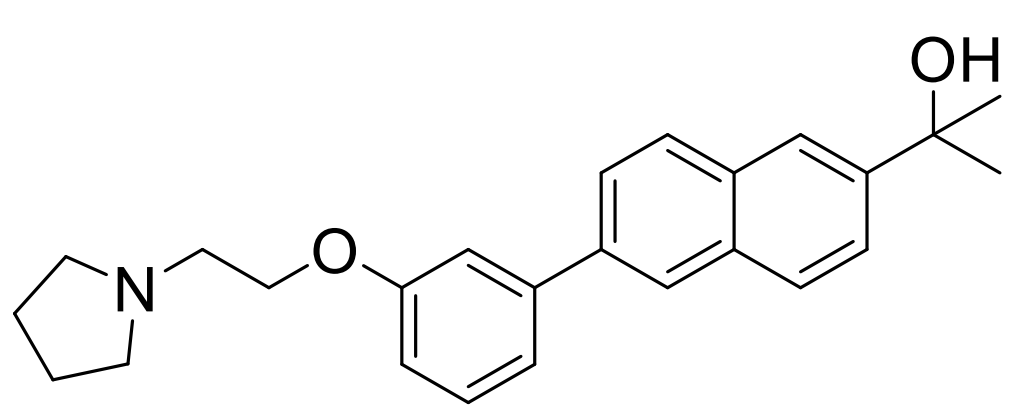

YKJ-54

2-(6-(3-(2-(Pyrrolidin-1-yl)ethoxy)phenyl)naphthalen-2-yl)propan-2-ol

Yield: 79.7% ^{1}H NMR (400 MHz, DMSO) δ 8.19 (d, *J* = 2.0 Hz, 1H), 8.00 – 7.90 (m, 3H), 7.83 (dd, *J* = 8.5, 1.9 Hz, 1H), 7.67 (dd, *J* = 8.6, 1.8 Hz, 1H), 7.47 – 7.35 (m, 3H), 6.99 (dt, *J* = 7.3, 2.2 Hz, 1H), 5.19 (s, 1H), 4.30 (t, *J* = 5.6 Hz, 2H), 3.17 (m, 2H), 2.91 (m, 4H), 1.81 (m, 4H), 1.53 (s,

6H). ^{13}C NMR (101 MHz, DMSO) δ 158.71, 148.40, 141.62, 136.63, 132.10, 131.89, 130.12, 128.56, 127.70, 125.02, 124.91, 124.63, 121.93, 119.54, 113.70, 112.95, 70.82, 53.92, 53.70, 31.82, 22.91 (one high field carbon not observed) ESI–MS calcd for $C_{25}H_{30}NO_2$ $[M + H]^+$ 376.2271; 376.2273.

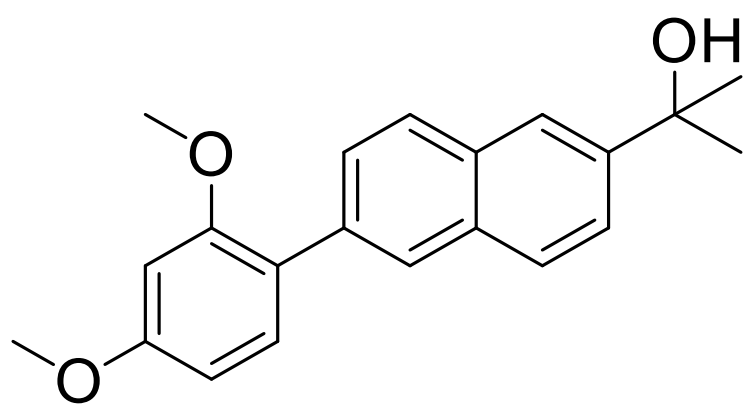

YKJ-55

2-(6-(2,4-Dimethoxyphenyl)naphthalen-2-yl)propan-2-ol

Yield: 86.5% ^{1}H NMR (500 MHz, DMSO) δ 7.95 – 7.92 (m, 1H), 7.88 – 7.82 (m, 3H), 7.62 (dd, *J* = 8.5, 1.8 Hz, 1H), 7.58 (dd, *J* = 8.4, 1.8 Hz, 1H), 7.32 (d, *J* = 8.3 Hz, 1H), 6.70 (d, *J* = 2.4 Hz, 1H), 6.65 (dd, *J* = 8.4, 2.4 Hz, 1H), 5.16 (s, 1H), 3.82 (s, 3H), 3.78 (s, 3H), 1.52 (s, 6H). ^{13}C NMR (126 MHz, DMSO) δ 160.16, 157.32, 147.87, 135.22, 131.63, 131.38, 131.19, 127.93, 127.36, 127.16, 126.86, 124.18, 122.48, 121.80, 105.42, 99.03, 70.83, 55.62, 55.32, 31.87. ESI–MS calcd for $C_{21}H_{21}O_2$ $[M - OH]^+$ 305.1536; found 305.1530.

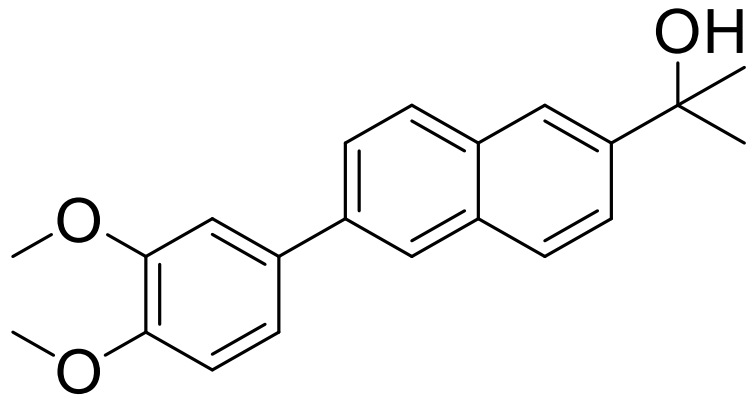

YKJ-56

2-(6-(3,4-Dimethoxyphenyl)naphthalen-2-yl)propan-2-ol

Yield: 88.2% ^{1}H NMR (500 MHz, DMSO) δ 8.14 (d, *J* = 1.8 Hz, 1H), 7.97 – 7.87 (m, 3H), 7.82 (dd, *J* = 8.5, 1.9 Hz, 1H), 7.65 (dd, *J* = 8.6, 1.8 Hz, 1H), 7.39 – 7.31 (m, 2H), 7.07 (d, *J* = 8.4 Hz, 1H), 5.16 (s, 1H), 3.89 (s, 3H), 3.81 (s, 3H), 1.53 (s, 6H). ^{13}C NMR (126 MHz, DMSO) δ 149.16, 148.57, 147.95, 136.83, 132.76, 132.01, 131.69, 128.44, 127.51, 124.98, 124.54, 124.02, 121.93, 119.07, 112.24, 110.51, 70.82, 55.64, 55.60, 31.83. ESI–MS calcd for $C_{21}H_{21}O_2$ $[M - OH]^+$ 305.1536; found 305.1535.

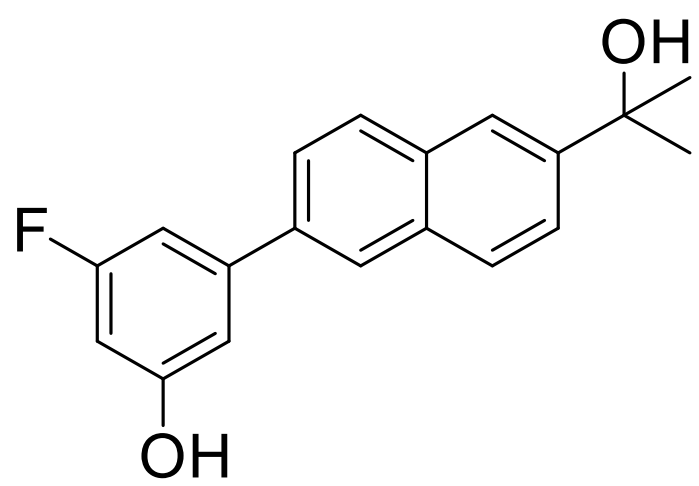

YKJ-57

3-Fluoro-5-(6-(2-hydroxypropan-2-yl)naphthalen-2-yl)phenol

Yield: 82.3% ^{1}H NMR (400 MHz, DMSO) δ 10.06 (s, 1H), 8.15 (d, *J* = 1.9 Hz, 1H), 8.00 – 7.90 (m, 3H), 7.75 (dd, *J* = 8.5, 1.9 Hz, 1H), 7.67 (dd, *J* = 8.6, 1.8 Hz, 1H), 7.10 – 7.01 (m, 2H), 6.59 (dt, *J* = 10.8, 2.3 Hz, 1H), 5.18 (s, 1H), 1.52 (s, 6H). ^{13}C NMR (126 MHz, DMSO) δ 163.51 (d, *J* = 241.5 Hz), 159.37 (d, *J* = 12.4 Hz), 148.64, 142.96 (d, *J* = 10.3 Hz), 135.81 (d, *J* = 2.7 Hz), 132.30, 131.81, 128.67, 127.83, 125.03, 124.73 (two peaks overlap), 121.95, 110.00 (d, *J* = 2.3 Hz), 104.27 (d, *J* = 22.5 Hz), 101.56 (d, *J* = 23.8 Hz), 70.86, 31.82. ESI–MS calcd for $C_{19}H_{16}FO$ $[M - OH]^+$ 279.1180; found 279.1181.

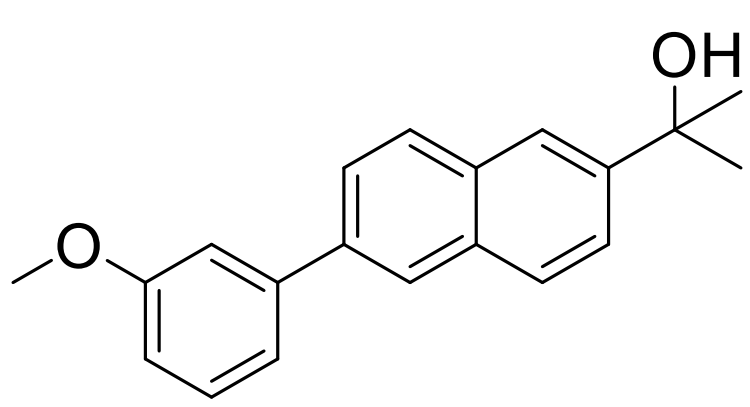

YKJ-58

2-(6-(3-Methoxyphenyl)naphthalen-2-yl)propan-2-ol

Yield: 83.8% ^{1}H NMR (500 MHz, DMSO) δ 8.18 (d, *J* = 1.8 Hz, 1H), 7.99 – 7.90 (m, 3H), 7.82 (dd, *J* = 8.5, 2.0 Hz, 1H), 7.67 (dd, *J* = 8.6, 1.8 Hz, 1H), 7.45 – 7.31 (m, 3H), 6.99 – 6.93 (m, 1H), 5.18 (s, 1H), 3.86 (s, 3H), 1.53 (s, 6H). ^{13}C NMR (126 MHz, DMSO) δ 159.84, 148.37, 141.59, 136.76, 132.09, 131.91, 130.07, 128.56, 127.72, 125.06, 124.91, 124.62, 121.94, 119.24, 113.10, 112.30, 70.84, 55.20, 31.82. ESI–MS calcd for $C_{20}H_{19}O$ $[M - OH]^+$ 275.1430; found 275.1429.

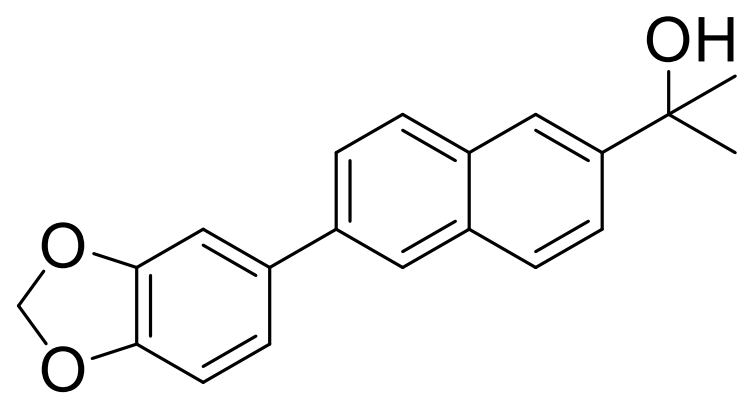

YKJ-59

2-(6-(Benzo[*d*][1,3]dioxol-5-yl)naphthalen-2-yl)propan-2-ol

Yield: 93.9 % ^{1}H NMR (500 MHz, DMSO) δ 8.10 (d, *J* = 1.9 Hz, 1H), 7.97 – 7.86 (m, 3H), 7.76 (dd, *J* = 8.5, 1.9 Hz, 1H), 7.65 (dd, *J* = 8.6, 1.8 Hz, 1H), 7.39 (d, *J* = 1.8 Hz, 1H), 7.29 (dd, *J* = 8.1, 1.9 Hz, 1H), 7.04 (d, *J* = 8.1 Hz, 1H), 6.08 (s, 2H), 5.16 (s, 1H), 1.52 (s, 6H). ^{13}C NMR (126 MHz, DMSO) δ 148.10, 148.09, 146.87, 136.62, 134.36, 131.95, 131.75, 128.48, 127.57, 124.94, 124.57, 124.28, 121.90, 120.48, 108.75, 107.25, 101.18, 70.82, 31.82. ESI–MS calcd for $C_{20}H_{17}O_2$ [M - OH]$^+$ 289.1223; found 289.1220.

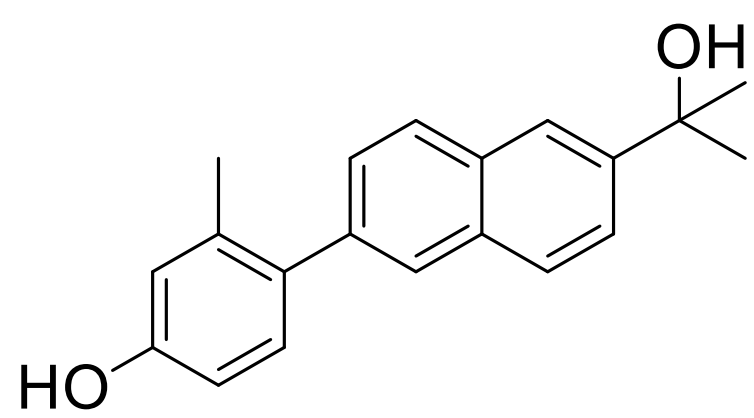

YKJ-60

4-(6-(2-Hydroxypropan-2-yl)naphthalen-2-yl)-3-methylphenol

Yield: 84.7% ^{1}H NMR (400 MHz, DMSO) δ 9.38 (s, 1H), 7.99 – 7.94 (m, 1H), 7.88 (d, *J* = 8.5 Hz, 1H), 7.85 (d, *J* = 8.6 Hz, 1H), 7.75 – 7.70 (m, 1H), 7.64 (dd, *J* = 8.6, 1.8 Hz, 1H), 7.42 (dd, *J* = 8.4, 1.8 Hz, 1H), 7.10 (d, *J* = 8.2 Hz, 1H), 6.75 – 6.66 (m, 2H), 5.16 (s, 1H), 2.20 (s, 3H), 1.52 (s, 6H). ^{13}C NMR (101 MHz, DMSO) δ 156.57, 147.90, 138.39, 136.03, 132.19, 131.58, 131.28, 130.84, 127.86, 127.45, 127.27, 126.93, 124.31, 121.89, 116.97, 112.95, 70.81, 31.86, 20.44. ESI–MS calcd for $C_{20}H_{19}O$ [M - OH]$^+$ 275.1430; found 275.1430.

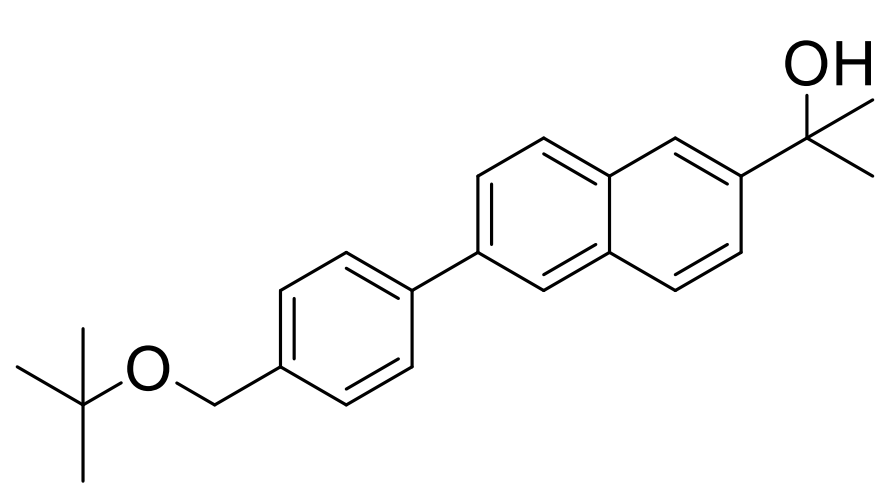

YKJ-61

2-(6-(4-(tert-Butyloxymethyl)phenyl)naphthalen-2-yl)propan-2-ol

Yield: 82.2% ^{1}H NMR (500 MHz, DMSO) δ 8.15 (d, *J* = 1.8 Hz, 1H), 8.00 – 7.89 (m, 3H), 7.81 (dd, *J* = 8.6, 1.9 Hz, 1H), 7.76 (d, *J* = 8.3 Hz, 2H), 7.67 (dd, *J* = 8.6, 1.8 Hz, 1H), 7.43 (d, *J* = 8.4 Hz, 2H), 5.17 (s, 1H), 4.46 (s, 2H), 1.53 (s, 6H), 1.26 (s, 9H). ^{13}C NMR (126 MHz, DMSO) δ 148.26, 139.28, 138.69, 136.77, 131.96, 131.94, 128.61, 127.92, 127.65, 126.59, 124.95, 124.62, 124.55, 121.95, 72.98, 70.83, 63.07, 31.82, 27.53. ESI–MS calcd for $C_{24}H_{27}O$ $[M - OH]^+$ 331.2056; found 331.2055.

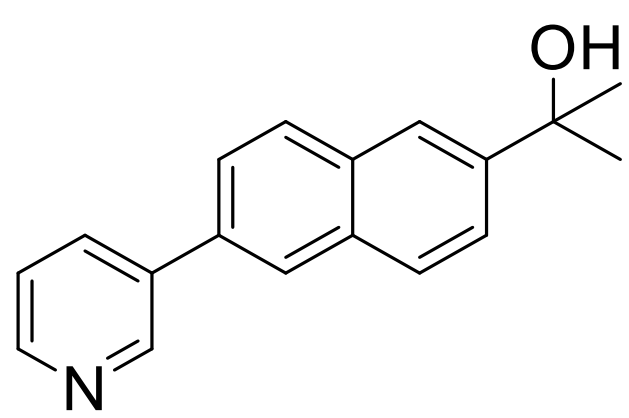

YKJ-62

2-(6-(Pyridin-3-yl)naphthalen-2-yl)propan-2-ol

Yield: 69.3% ^{1}H NMR (400 MHz, DMSO) δ 9.09 (s, 1H), 8.65 (s, 1H), 8.27 (d, *J* = 1.9 Hz, 1H), 8.22 (d, *J* = 8.0 Hz, 1H), 8.05 – 7.99 (m, 2H), 7.95 (d, *J* = 8.7 Hz, 1H), 7.87 (dd, *J* = 8.5, 1.8 Hz, 1H), 7.70 (dd, *J* = 8.6, 1.8 Hz, 1H), 7.58 – 7.50 (m, 1H), 5.19 (s, 1H), 1.53 (s, 6H). ^{13}C NMR (101 MHz, DMSO) δ 148.72, 148.39, 147.80, 134.20, 133.76, 132.23, 131.84, 128.87, 127.72, 125.28, 124.76, 124.66, 121.96, 70.79, 31.76 (two low field carbons not observed). ESI–MS calcd for $C_{18}H_{18}NO$ $[M + H]^+$ 264.1383; found 264.1383.

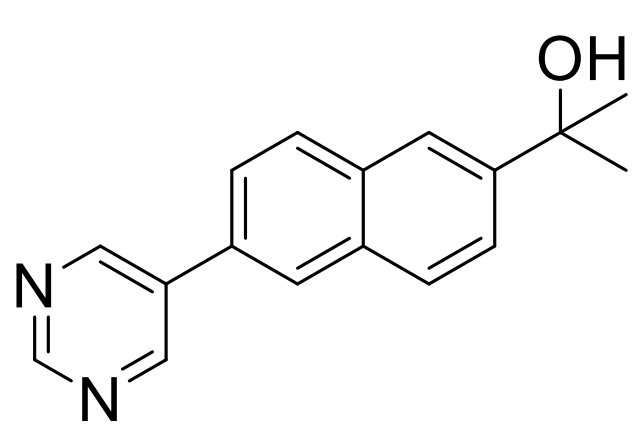

YKJ-63

2-(6-(Pyrimidin-5-yl)naphthalen-2-yl)propan-2-ol

Yield: 85.9% ^{1}H NMR (400 MHz, DMSO) δ 9.29 (s, 2H), 9.21 (s, 1H), 8.37 (d, *J* = 1.9 Hz, 1H), 8.09 – 8.01 (m, 2H), 7.98 – 7.90 (m, 2H), 7.72 (dd, *J* = 8.6, 1.8 Hz, 1H), 5.21 (s, 1H), 1.53 (s, 6H).

^{13}C NMR (101 MHz, DMSO) δ 157.17, 154.82, 149.17, 133.16, 132.57, 131.78, 130.45, 129.12, 127.81, 125.69, 124.98, 124.27, 122.08, 70.83, 31.77. ESI–MS calcd for $C_{17}H_{17}N_2O$ $[M + H]^+$ 265.1335; found 265.1335.

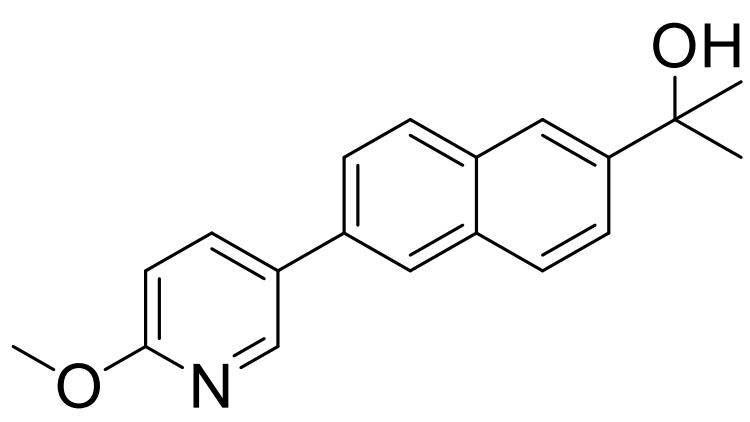

YKJ-64

2-(6-(6-Methoxypyridin-3-yl)naphthalen-2-yl)propan-2-ol

Yield: 93.7% ^1H NMR (400 MHz, DMSO) δ 8.63 (dd, *J* = 2.6, 0.8 Hz, 1H), 8.19 – 8.13 (m, 2H), 8.00 – 7.95 (m, 2H), 7.91 (d, *J* = 8.7 Hz, 1H), 7.81 (dd, *J* = 8.6, 1.9 Hz, 1H), 7.67 (dd, *J* = 8.6, 1.8 Hz, 1H), 6.95 (dd, *J* = 8.7, 0.8 Hz, 1H), 5.17 (s, 1H), 3.92 (s, 3H), 1.53 (s, 6H). ^{13}C NMR (101 MHz, DMSO) δ 163.06, 148.31, 144.85, 137.68, 133.72, 131.94, 131.87, 129.21, 128.75, 127.54, 124.69, 124.53, 124.27, 121.97, 110.67, 70.80, 53.30, 31.80. ESI–MS calcd for $C_{19}H_{20}NO_2$ $[M + H]^+$ 294.1489; found 294.1486.

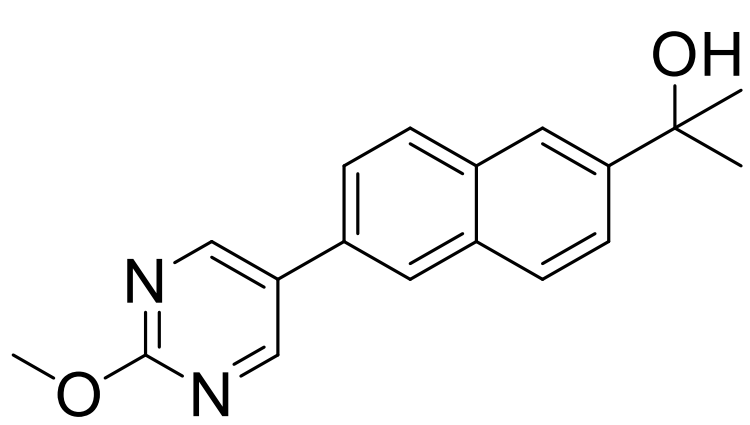

YKJ-65

2-(6-(2-Methoxypyrimidin-5-yl)naphthalen-2-yl)propan-2-ol

Yield: 93.05% ^1H NMR (400 MHz, DMSO) δ 9.07 (s, 2H), 8.26 (d, *J* = 1.9 Hz, 1H), 8.04 – 7.98 (m, 2H), 7.91 (d, *J* = 8.6 Hz, 1H), 7.86 (dd, *J* = 8.5, 1.9 Hz, 1H), 7.70 (dd, *J* = 8.6, 1.8 Hz, 1H), 5.19 (s, 1H), 3.99 (s, 3H), 1.53 (s, 6H). ^{13}C NMR (101 MHz, DMSO) δ 164.53, 157.40, 148.68, 132.15, 131.83, 130.58, 128.95, 127.58, 127.33, 124.85, 124.53, 124.16, 122.04, 70.80, 54.74, 31.77. ESI–MS calcd for $C_{18}H_{19}N_2O_2$ $[M + H]^+$ 295.1441; found 295.1440.

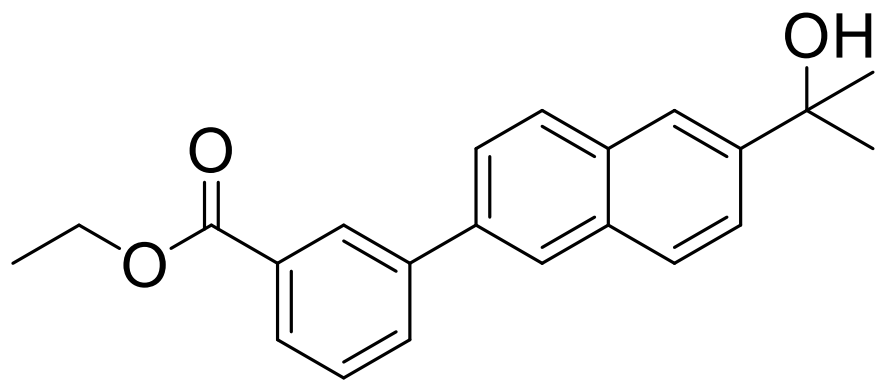

YKJ-66

Ethyl 3-(6-(2-hydroxypropan-2-yl)naphthalen-2-yl)benzoate

Yield: 83.8 % ^{1}H NMR (400 MHz, DMSO) δ 8.32 (t, *J* = 1.8 Hz, 1H), 8.23 (d, *J* = 1.9 Hz, 1H), 8.12 – 8.05 (m, 1H), 8.05 – 7.94 (m, 4H), 7.84 (dd, *J* = 8.5, 1.9 Hz, 1H), 7.73 – 7.62 (m, 2H), 5.19 (s, 1H), 4.37 (q, *J* = 7.1 Hz, 2H), 1.53 (s, 6H), 1.36 (t, *J* = 7.1 Hz, 3H). ^{13}C NMR (101 MHz, DMSO) δ 165.71, 148.66, 140.61, 135.80, 132.20, 131.90, 131.66, 130.70, 129.55, 128.87, 128.02, 127.81, 127.23, 125.16, 124.79, 124.76, 121.96, 70.83, 60.92, 31.79, 14.20. ESI–MS calcd for $C_{22}H_{23}O_3$ $[M + H]^+$ 335.1642; found 335.1648.

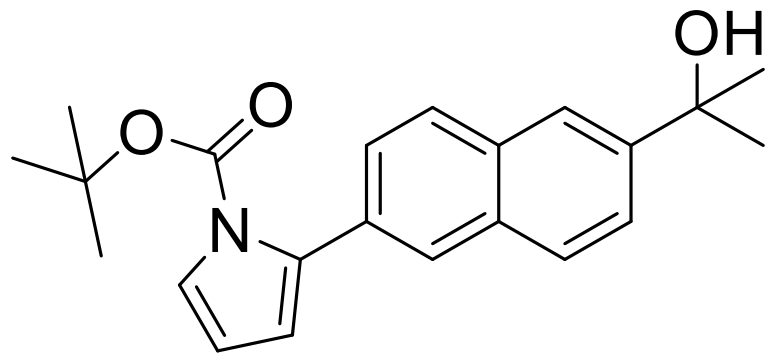

YKJ-67

tert-Butyl 2-(6-(2-hydroxypropan-2-yl)naphthalen-2-yl)-1*H*-pyrrole-1-carboxylate

Yield: 75.5% ^{1}H NMR (400 MHz, DMSO) δ 7.97 – 7.94 (m, 1H), 7.86 (d, *J* = 3.0 Hz, 1H), 7.84 (d, *J* = 3.1 Hz, 1H), 7.82 – 7.79 (m, 1H), 7.65 (dd, *J* = 8.6, 1.8 Hz, 1H), 7.44 (dd, *J* = 8.4, 1.8 Hz, 1H), 7.39 (dd, *J* = 3.3, 1.8 Hz, 1H), 5.16 (s, 1H), 1.52 (s, 6H), 1.27 (s, 9H). ^{13}C NMR (101 MHz, DMSO) δ 148.85, 148.17, 134.55, 131.68, 131.15, 130.67, 127.46, 127.27, 126.89, 126.37, 124.42, 122.78, 121.91, 114.68, 110.96, 83.62, 70.80, 31.81, 27.10. ESI–MS calcd for $C_{22}H_{24}NO_2$ $[M - OH]^+$ 334.1802; found 334.1802.

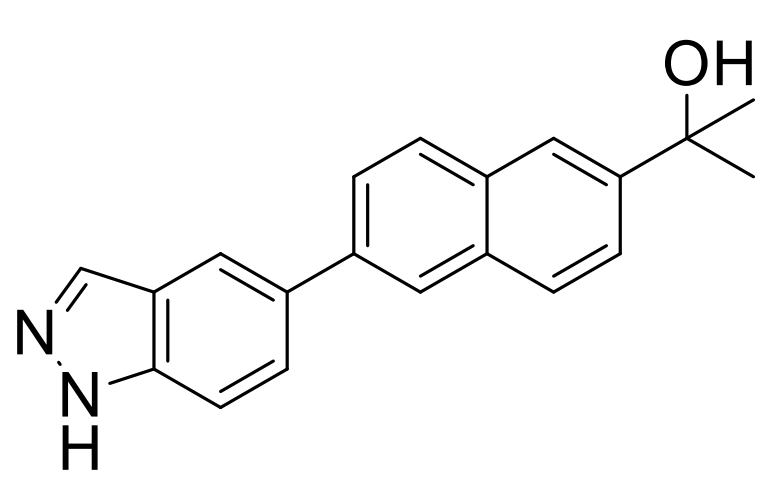

YKJ-68

2-(6-(1*H*-Indazol-5-yl)naphthalen-2-yl)propan-2-ol

Yield: 57.9 % ^{1}H NMR (400 MHz, DMSO) δ 13.14 (s, 1H), 8.20 – 8.13 (m, 3H), 8.00 – 7.95 (m, 2H), 7.92 (d, *J* = 8.6 Hz, 1H), 7.86 (dd, *J* = 8.6, 1.9 Hz, 1H), 7.82 (dd, *J* = 8.7, 1.7 Hz, 1H), 7.69 – 7.64 (m, 2H), 5.17 (s, 1H), 1.53 (s, 6H). ^{13}C NMR (101 MHz, DMSO) δ 148.01, 139.37, 137.56, 134.03, 132.63, 132.06, 131.64, 128.55, 127.54, 125.80, 125.35, 124.61, 124.56, 123.65, 121.91, 118.49, 110.61, 70.81, 31.82. ESI–MS calcd for $C_{20}H_{19}N_2O$ $[M + H]^+$ 303.1492 found 303.1496.

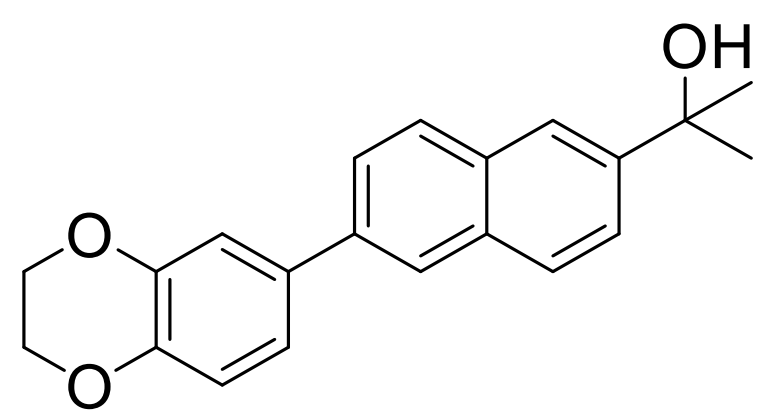

YKJ-69

2-(6-(2,3-Dihydrobenzo[*b*][1,4]dioxin-6-yl)naphthalen-2-yl)propan-2-ol

Yield: 87.1 % ^{1}H NMR (400 MHz, DMSO) δ 8.08 (d, *J* = 2.1 Hz, 1H), 7.96 – 7.86 (m, 3H), 7.75 (dd, *J* = 8.6, 1.9 Hz, 1H), 7.64 (dd, *J* = 8.6, 1.9 Hz, 1H), 7.32 – 7.25 (m, 2H), 7.00 – 6.95 (m, 1H), 5.16 (s, 1H), 4.29 (s, 4H), 1.52 (s, 6H). ^{13}C NMR (101 MHz, DMSO) δ 148.04, 143.75, 143.14, 136.31, 133.35, 131.96, 131.71, 128.47, 127.55, 124.73, 124.52, 124.04, 121.88, 119.69, 117.55, 115.26, 70.80, 64.19, 64.14, 31.81. ESI–MS calcd for $C_{21}H_{19}O_2$ $[M - OH]^+$ 303.1380; found 303.1380.

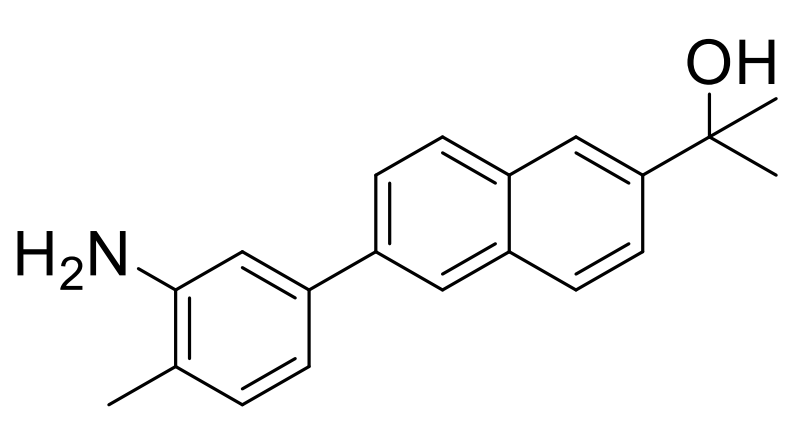

YKJ-70

2-(6-(3-Amino-4-methylphenyl)naphthalen-2-yl)propan-2-ol

Yield: 96.6% ^{1}H NMR (400 MHz, DMSO) δ 8.01 (s, 1H), 7.97 – 7.85 (m, 3H), 7.73 – 7.62 (m, 2H), 7.09 – 7.00 (m, 2H), 6.93 – 6.87 (m, 1H), 5.15 (s, 1H), 4.94 (s, 2H), 2.11 (s, 3H), 1.52 (s, 6H). ^{13}C NMR (101 MHz, DMSO) δ 147.90, 147.01, 138.35, 137.77, 131.94, 131.73, 130.50, 128.32,

127.47, 124.94, 124.47, 123.91, 121.89, 120.59, 114.73, 112.28, 70.79, 31.81, 17.14. ESI–MS calcd for $C_{20}H_{22}NO$ $[M + H]^+$ 292.1696; found 292.1696.

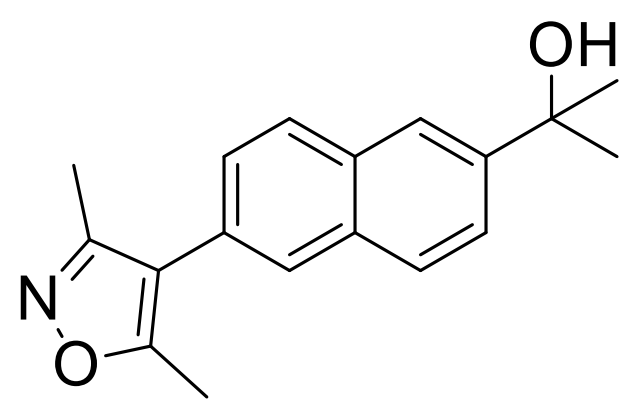

YKJ-71

2-(6-(3,5-Dimethylisoxazol-4-yl)naphthalen-2-yl)propan-2-ol

Yield: 87.5% ^{1}H NMR (400 MHz, DMSO) δ 8.02 – 7.95 (m, 2H), 7.93 – 7.85 (m, 2H), 7.68 (dd, *J* = 8.6, 1.8 Hz, 1H), 7.49 (dd, *J* = 8.4, 1.8 Hz, 1H), 5.18 (s, 1H), 2.45 (s, 3H), 2.28 (s, 3H), 1.52 (s, 6H). ^{13}C NMR (101 MHz, DMSO) δ 165.19, 158.28, 148.58, 131.76, 131.67, 128.45, 127.31 (two peaks overlap), 126.71(two peaks overlap), 124.66, 122.00, 116.03, 70.81, 31.82, 11.39, 10.54. ESI–MS calcd for $C_{18}H_{20}NO_2$ $[M + H]^+$ 282.1489; found 282.1488.

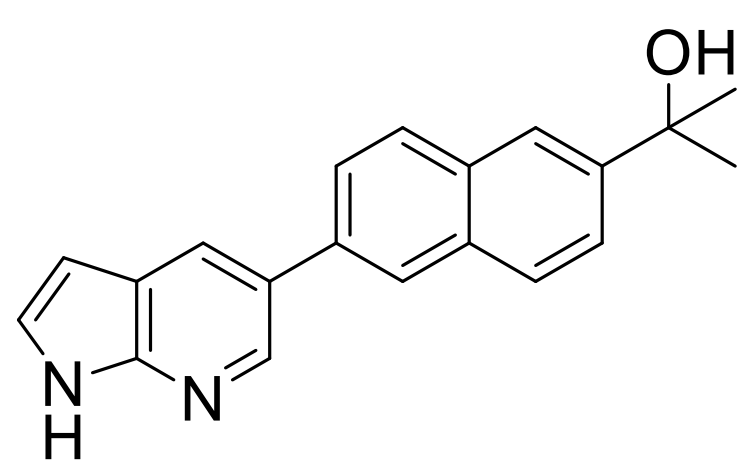

YKJ-72

2-(6-(1*H*-Pyrrolo[2,3-*b*]pyridin-5-yl)naphthalen-2-yl)propan-2-ol

Yield: 78.7% ^{1}H NMR (400 MHz, DMSO) δ 11.73 (s, 1H), 8.66 (d, *J* = 2.2 Hz, 1H), 8.34 (d, *J* = 2.2 Hz, 1H), 8.23 – 8.18 (m, 1H), 8.02 – 7.84 (m, 4H), 7.67 (dd, *J* = 8.6, 1.8 Hz, 1H), 7.53 (dd, *J* = 3.4, 2.4 Hz, 1H), 6.53 (dd, *J* = 3.4, 1.8 Hz, 1H), 5.17 (s, 1H), 1.53 (s, 6H). ^{13}C NMR (101 MHz, DMSO) δ 148.04, 141.72, 135.86, 132.08, 131.63, 128.63, 128.03, 127.50, 126.98, 126.23, 125.34, 124.62, 124.57, 121.93, 119.76, 100.20, 70.81, 31.82 (one low field carbon not observed). ESI–MS calcd for $C_{20}H_{19}N_2O$ $[M + H]^+$ 303.1492; found 303.1496.

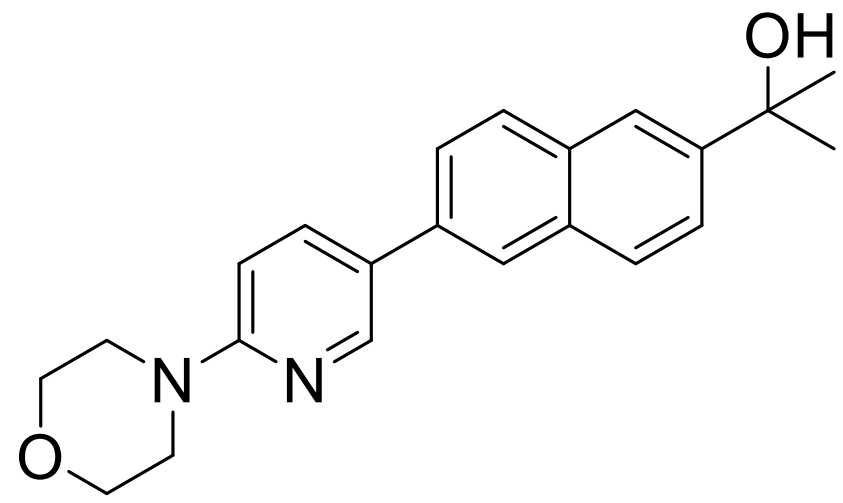

YKJ-73

2-(6-(6-Morpholinopyridin-3-yl)naphthalen-2-yl)propan-2-ol

Yield: 45.9% ^{1}H NMR (400 MHz, DMSO) δ 8.62 (dd, *J* = 2.6, 0.7 Hz, 1H), 8.12 (d, *J* = 1.8 Hz, 1H), 8.03 (dd, *J* = 8.9, 2.6 Hz, 1H), 7.97 – 7.91 (m, 2H), 7.88 (d, *J* = 8.7 Hz, 1H), 7.79 (dd, *J* = 8.6, 1.9 Hz, 1H), 7.65 (dd, *J* = 8.6, 1.8 Hz, 1H), 6.97 (d, *J* = 8.9 Hz, 1H), 5.15 (s, 1H), 3.76 – 3.69 (m, 4H), 3.55 – 3.48 (m, 4H), 1.52 (s, 6H). ^{13}C NMR (101 MHz, DMSO) δ 158.42, 147.92, 145.65, 135.87, 134.26, 132.05, 131.59, 128.62, 127.39, 125.16, 124.56, 124.23, 123.24, 121.94, 107.05, 70.78, 65.95, 45.16, 31.80. ESI–MS calcd for $C_{22}H_{25}N_2O_2$ $[M + H]^+$ 349.1911; found 349.1911.

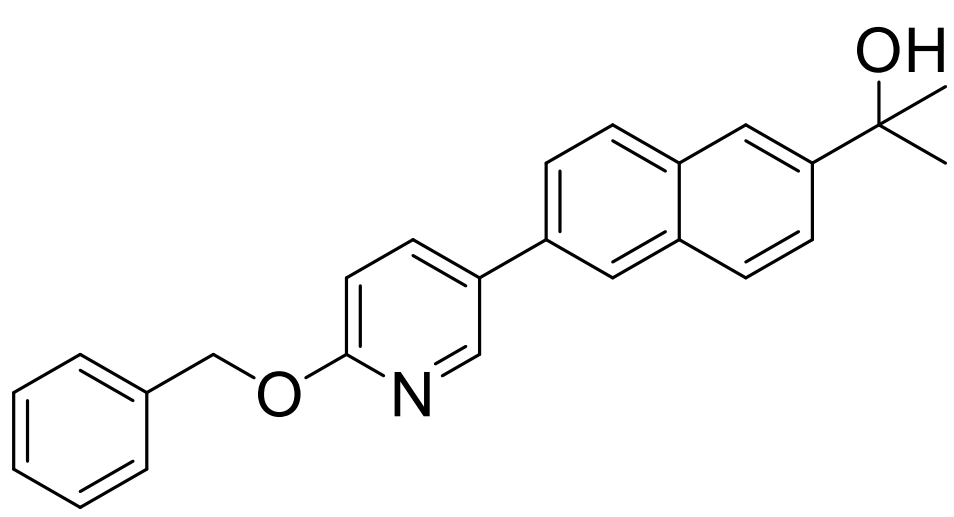

YKJ-75

2-(6-(6-(Benzyloxy)pyridin-3-yl)naphthalen-2-yl)propan-2-ol

Yield: quantitative ^{1}H NMR (400 MHz, DMSO) δ 8.63 (dd, *J* = 2.6, 0.8 Hz, 1H), 8.21 – 8.15 (m, 2H), 8.01 – 7.96 (m, 2H), 7.90 (d, *J* = 8.7 Hz, 1H), 7.82 (dd, *J* = 8.6, 1.9 Hz, 1H), 7.67 (dd, *J* = 8.6, 1.8 Hz, 1H), 7.51 – 7.46 (m, 2H), 7.43 – 7.31 (m, 3H), 7.02 (dd, *J* = 8.6, 0.8 Hz, 1H), 5.43 (s, 2H), 5.18 (s, 1H), 1.53 (s, 6H). ^{13}C NMR (101 MHz, DMSO) δ 162.49, 148.33, 144.77, 137.85, 137.29, 133.65, 131.93, 131.89, 129.45, 128.75, 128.37, 127.89, 127.74, 127.54, 124.68, 124.51, 124.31, 121.96, 110.96, 70.80, 67.05, 31.79. ESI–MS calcd for $C_{25}H_{24}NO_2$ $[M + H]^+$ 370.1802; found 370.1808.

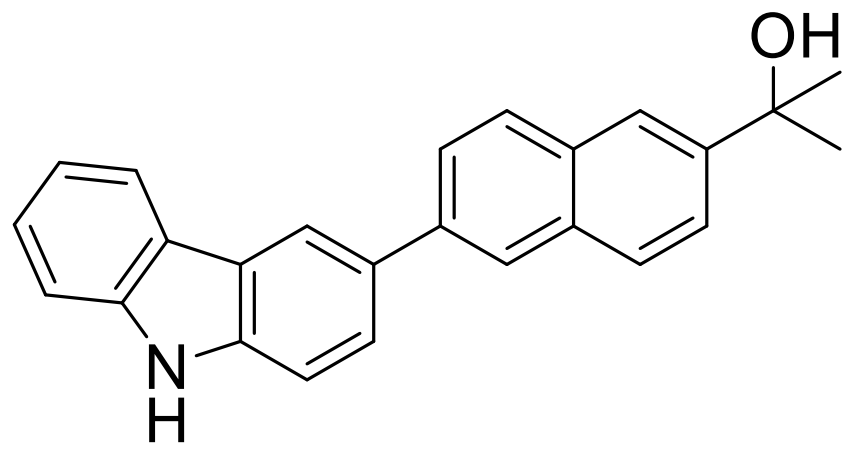

YKJ-76

2-(6-(9*H*-carbazol-3-yl)naphthalen-2-yl)propan-2-ol

Yield: 81.2% ^{1}H NMR (400 MHz, DMSO) δ 11.34 (s, 1H), 8.59 (d, *J* = 1.9 Hz, 1H), 8.32 – 8.20 (m, 2H), 8.04 – 7.92 (m, 4H), 7.86 (dd, *J* = 8.5, 1.9 Hz, 1H), 7.67 (dd, *J* = 8.7, 1.8 Hz, 1H), 7.60 (dd, *J* = 8.4, 0.6 Hz, 1H), 7.51 (dt, *J* = 8.1, 0.9 Hz, 1H), 7.41 (ddd, *J* = 8.2, 7.1, 1.2 Hz, 1H), 7.19 (ddd, *J* = 8.0, 7.1, 1.0 Hz, 1H), 5.17 (s, 1H), 1.54 (s, 6H). ^{13}C NMR (101 MHz, DMSO) δ 147.75, 140.25, 139.29, 138.07, 132.18, 131.48, 130.78, 128.46, 127.47, 125.75, 125.43, 124.83, 124.49, 124.14, 123.18, 122.64, 121.94, 120.48, 118.68, 118.48, 111.38, 111.09, 70.81, 31.83. ESI–MS calcd for $C_{25}H_{22}NO$ $[M + H]^+$ 351.1623; found 351.1627.

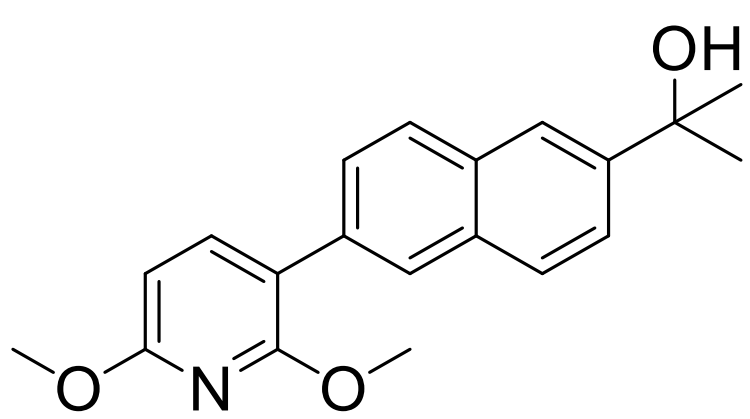

YKJ-77

2-(6-(2,6-Dimethoxypyridin-3-yl)naphthalen-2-yl)propan-2-ol

Yield: 94.2% ^{1}H NMR (400 MHz, DMSO) δ 7.98 – 7.93 (m, 2H), 7.91 – 7.83 (m, 2H), 7.82 (d, *J* = 8.1 Hz, 1H), 7.66 (dd, *J* = 4.9, 1.8 Hz, 1H), 7.63 (dd, *J* = 5.0, 1.8 Hz, 1H), 6.52 (d, *J* = 8.1 Hz, 1H), 5.16 (s, 1H), 3.93 (s, 3H), 3.93 (s, 3H), 1.52 (s, 6H). ^{13}C NMR (101 MHz, DMSO) δ 161.72, 158.89, 148.09, 142.04, 133.29, 131.58, 131.52, 127.49, 127.38, 127.17, 126.70, 124.32, 121.82, 115.02, 101.46, 70.79, 53.38, 53.30, 31.82. ESI–MS calcd for $C_{20}H_{22}NO_3$ $[M + H]^+$ 324.1594; found 324.1596.

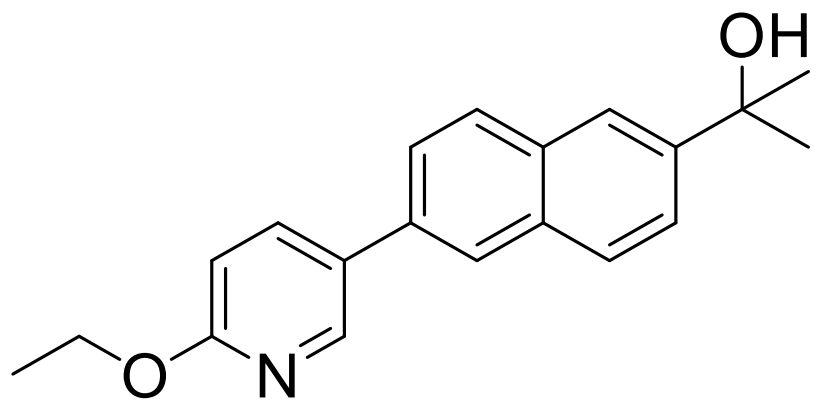

YKJ-78

2-(6-(6-Ethoxypyridin-3-yl)naphthalen-2-yl)propan-2-ol

Yield: quantitative ^{1}H NMR (400 MHz, DMSO) δ 8.61 (dd, *J* = 2.6, 0.8 Hz, 1H), 8.18 – 8.12 (m, 2H), 7.99 – 7.95 (m, 2H), 7.90 (d, *J* = 8.7 Hz, 1H), 7.81 (dd, *J* = 8.6, 1.9 Hz, 1H), 7.67 (dd, *J* = 8.7, 1.9 Hz, 1H), 6.92 (dd, *J* = 8.6, 0.7 Hz, 1H), 5.17 (s, 1H), 4.37 (q, *J* = 7.0 Hz, 2H), 1.52 (s, 6H), 1.35 (t, *J* = 7.0 Hz, 3H). ^{13}C NMR (101 MHz, DMSO) δ 162.74, 148.29, 144.87, 137.63, 133.75, 131.94, 131.85, 129.01, 128.73, 127.53, 124.67, 124.50, 124.21, 121.96, 110.76, 70.80, 61.33, 31.79, 14.56. ESI–MS calcd for $C_{20}H_{22}NO_2$ $[M + H]^+$ 308.1645; 308.1646.

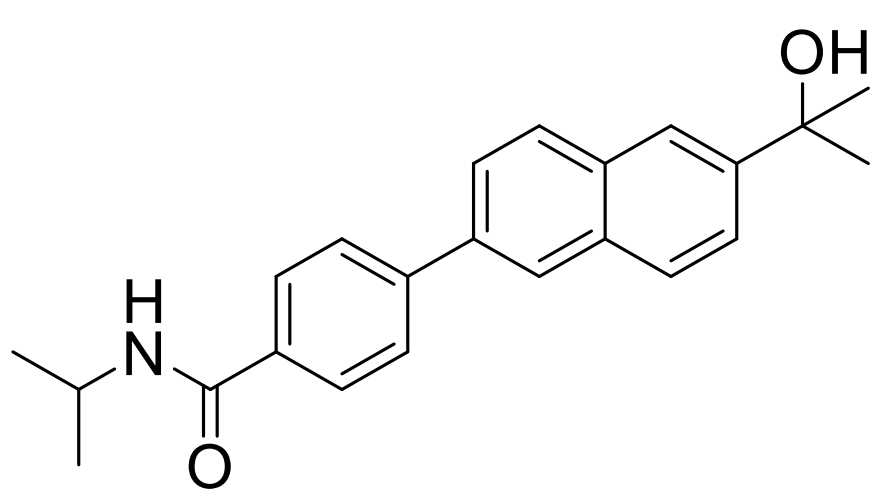

YKJ-79

4-(6-(2-Hydroxypropan-2-yl)naphthalen-2-yl)-*N*-isopropylbenzamide

Yield: 67.5% ^{1}H NMR (400 MHz, DMSO) δ 8.29 (d, *J* = 7.8 Hz, 1H), 8.26 (d, *J* = 1.9 Hz, 1H), 8.02 – 7.94 (m, 5H), 7.92 – 7.86 (m, 3H), 7.69 (dd, *J* = 8.6, 1.8 Hz, 1H), 5.18 (s, 1H), 4.14 (heptet, *J* = 6.7 Hz, 1H), 1.53 (s, 6H), 1.19 (d, *J* = 6.6 Hz, 6H). ^{13}C NMR (101 MHz, DMSO) δ 164.95, 148.63, 142.34, 135.88, 133.54, 132.24, 131.88, 128.71, 127.97, 127.79, 126.48, 125.15, 124.83, 124.73, 121.96, 70.82, 40.99, 31.79, 22.37. ESI–MS calcd for $C_{23}H_{26}NO_2$ $[M + H]^+$ 348.1958; found 348.1959

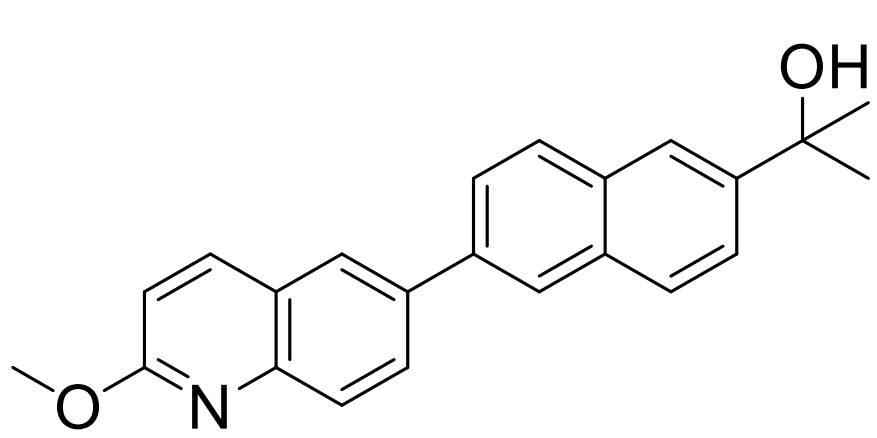

YKJ-80

2-(6-(2-Methoxyquinolin-6-yl)naphthalen-2-yl)propan-2-ol

Yield: 90.8% ^{1}H NMR (400 MHz, DMSO) δ 8.42 – 8.29 (m, 3H), 8.15 (dd, *J* = 8.7, 2.2 Hz, 1H), 8.07 – 7.86 (m, 5H), 7.69 (dd, *J* = 8.7, 1.8 Hz, 1H), 7.07 (d, *J* = 8.8 Hz, 1H), 5.19 (s, 1H), 4.02 (s, 3H), 1.54 (s, 6H). ^{13}C NMR (101 MHz, DMSO) δ 162.11, 148.41, 145.37, 139.62, 136.22, 135.65, 132.01, 131.99, 128.79, 128.74, 127.69, 127.34, 125.49, 125.21, 124.98, 124.92, 124.71, 121.98, 113.40, 70.83, 53.22, 31.81. ESI–MS calcd for $C_{23}H_{22}NO_2$ $[M + H]^+$ 334.1645; found 344.1640

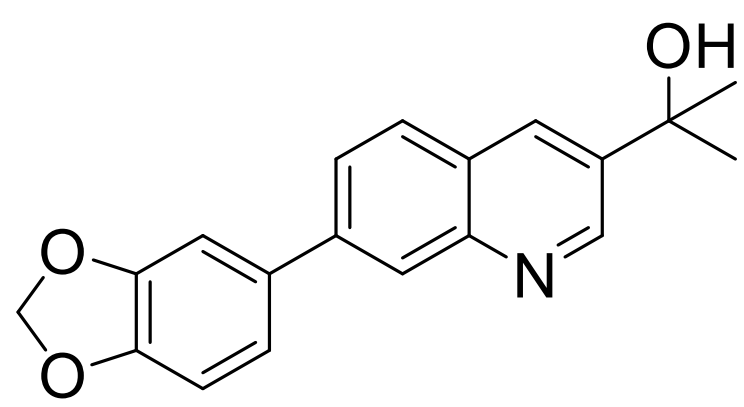

YKJ-81

2-(7-(Benzo[*d*][1,3]dioxol-5-yl)quinolin-3-yl)propan-2-ol

Yield: quantitative ^{1}H NMR (400 MHz, DMSO) δ 9.14 – 8.98 (m, 1H), 8.33 (d, *J* = 2.3 Hz, 1H), 8.17 (s, 1H), 8.01 (d, *J* = 8.5 Hz, 1H), 7.87 (dd, *J* = 8.5, 1.9 Hz, 1H), 7.45 (d, *J* = 1.9 Hz, 1H), 7.35 (dd, *J* = 8.1, 1.9 Hz, 1H), 7.06 (d, *J* = 8.1 Hz, 1H), 6.10 (s, 2H), 5.38 (s, 1H), 1.57 (s, 6H). ^{13}C NMR (101 MHz, DMSO) δ 149.70, 148.17, 147.28, 142.79, 140.14, 133.66, 129.91, 128.60, 126.20, 125.64, 125.09, 120.87, 108.81, 107.42, 101.27, 69.90, 31.55 (one low field carbon not observed). ESI–MS calcd for $C_{19}H_{18}NO_3$ $[M + H]^+$ 308.1281; found 308.1285

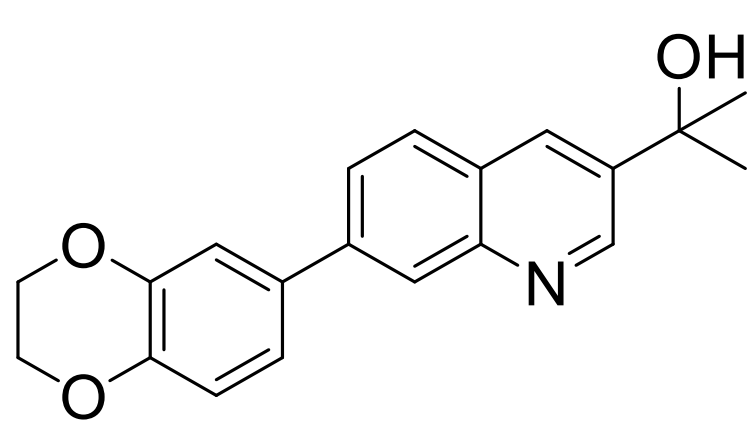

YKJ-82

2-(7-(2,3-Dihydrobenzo[*b*][1,4]dioxin-6-yl)quinolin-3-yl)propan-2-ol

Yield: quantitative ^{1}H NMR (400 MHz, DMSO) δ 9.06 (d, *J* = 2.3 Hz, 1H), 8.32 (d, *J* = 2.3 Hz, 1H), 8.15 (d, *J* = 1.8 Hz, 1H), 8.01 (d, *J* = 8.5 Hz, 1H), 7.86 (dd, *J* = 8.5, 1.9 Hz, 1H), 7.33 (m, 2H), 7.02 – 6.96 (m, 1H), 5.37 (s, 1H), 4.30 (s, 4H), 1.57 (s, 6H). ^{13}C NMR (101 MHz, DMSO) δ

149.68, 146.65, 143.82, 143.56, 142.76, 139.86, 132.68, 129.91, 128.61, 126.17, 125.47, 124.87, 119.97, 117.68, 115.51, 69.89, 64.21, 64.12, 31.56. ESI–MS calcd for $C_{20}H_{20}NO_3$ $[M + H]^+$ 322.1438; found 322.1435

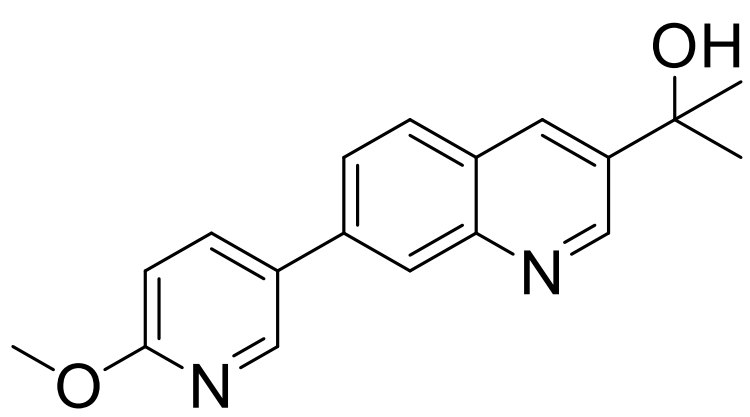

YKJ-83

2-(7-(6-Methoxypyridin-3-yl)quinolin-3-yl)propan-2-ol

Yield: quantitative ^{1}H NMR (400 MHz, DMSO) δ 9.08 (d, *J* = 2.3 Hz, 1H), 8.68 (d, *J* = 2.6 Hz, 1H), 8.35 (d, *J* = 2.3 Hz, 1H), 8.26 (s, 1H), 8.22 (dd, *J* = 8.6, 2.6 Hz, 1H), 8.07 (d, *J* = 8.5 Hz, 1H), 7.93 (dd, *J* = 8.5, 1.9 Hz, 1H), 6.97 (d, *J* = 8.7 Hz, 1H), 5.40 (s, 1H), 3.93 (s, 3H), 1.57 (s, 6H). ^{13}C NMR (101 MHz, DMSO) δ 163.37, 149.82, 145.21, 137.90, 132.05, 132.02, 131.53, 131.43, 130.00, 128.82, 128.70, 125.25, 125.11, 110.79, 69.92, 53.37, 31.55. ESI–MS calcd for $C_{18}H_{19}N_2O_2$ $[M + H]^+$ 295.1441; found 295.1441

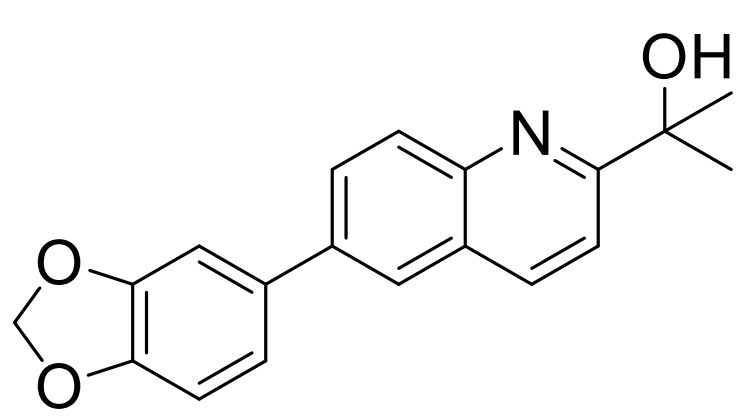

YKJ-84

2-(6-(Benzo[*d*][1,3]dioxol-5-yl)quinolin-2-yl)propan-2-ol

Yield: 67.7% ^{1}H NMR (400 MHz, DMSO) δ 8.35 (d, *J* = 8.7 Hz, 1H), 8.18 (t, *J* = 1.3 Hz, 1H), 8.03 – 7.96 (m, 2H), 7.87 (d, *J* = 8.7 Hz, 1H), 7.42 (d, *J* = 1.8 Hz, 1H), 7.32 (dd, *J* = 8.1, 1.9 Hz, 1H), 7.05 (d, *J* = 8.1 Hz, 1H), 6.09 (s, 2H), 5.44 (s, 1H), 1.55 (s, 6H). ^{13}C NMR (101 MHz, DMSO) δ 168.11, 148.14, 147.12, 145.41, 137.15, 136.63, 133.62, 129.04, 128.38, 126.84, 124.49, 120.68, 118.17, 108.78, 107.31, 101.25, 72.65, 30.48. ESI–MS calcd for $C_{19}H_{18}NO_3$ $[M + H]^+$ 308.1281; found 308.1286

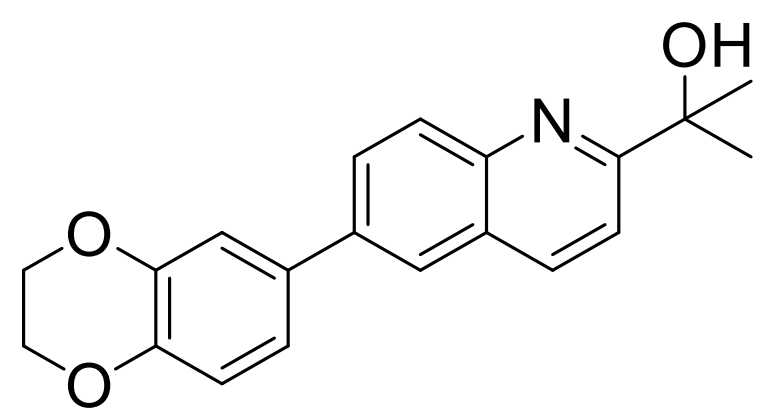

YKJ-85

2-(6-(2,3-Dihydrobenzo[*b*][1,4]dioxin-6-yl)quinolin-2-yl)propan-2-ol

Yield: 93.0% ^{1}H NMR (400 MHz, DMSO) δ 8.36 (d, *J* = 8.6 Hz, 1H), 8.21 – 8.16 (m, 1H), 8.03 – 7.97 (m, 2H), 7.86 (d, *J* = 8.6 Hz, 1H), 7.36 – 7.28 (m, 2H), 6.99 (d, *J* = 8.3 Hz, 1H), 5.44 (s, 1H), 4.30 (s, 4H), 1.54 (s, 6H). ^{13}C NMR (101 MHz, DMSO) δ 168.06, 145.40, 143.82, 143.41, 136.85, 136.64, 132.62, 129.06, 128.19, 126.88, 124.27, 119.83, 118.13, 117.64, 115.42, 72.65, 64.21, 64.15, 30.49. ESI–MS calcd for $C_{20}H_{20}NO_3$ $[M + H]^+$ 322.1438; found 322.1442

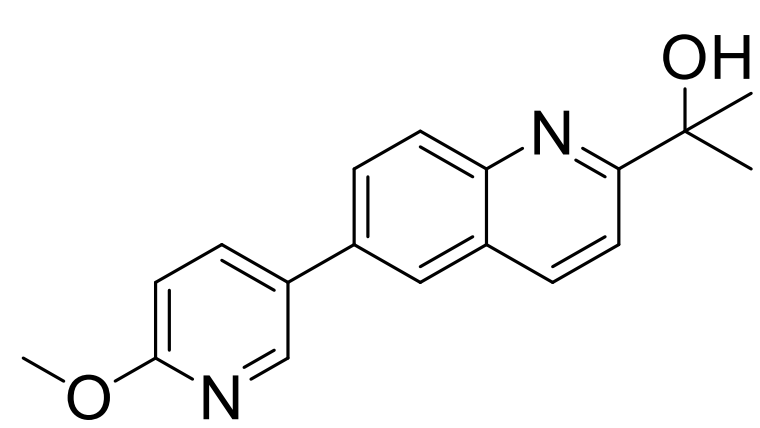

YKJ-86

2-(6-(6-Methoxypyridin-3-yl)quinolin-2-yl)propan-2-ol

Yield: 94.6% ^{1}H NMR (400 MHz, DMSO) δ 8.65 (dd, *J* = 2.6, 0.8 Hz, 1H), 8.41 – 8.35 (m, 1H), 8.25 (d, *J* = 1.9 Hz, 1H), 8.18 (dd, *J* = 8.6, 2.6 Hz, 1H), 8.08 – 8.00 (m, 2H), 7.89 (d, *J* = 8.6 Hz, 1H), 6.97 (dd, *J* = 8.6, 0.7 Hz, 1H), 5.45 (s, 1H), 3.92 (s, 3H), 1.55 (s, 6H). ^{13}C NMR (101 MHz, DMSO) δ 168.36, 163.24, 145.54, 145.05, 137.77, 136.62, 134.33, 129.33, 128.57, 128.03, 126.88, 124.54, 118.30, 110.75, 72.68, 53.35, 30.47. ESI–MS calcd for $C_{18}H_{19}N_2O_2$ $[M + H]^+$ 295.1441; found 295.1443

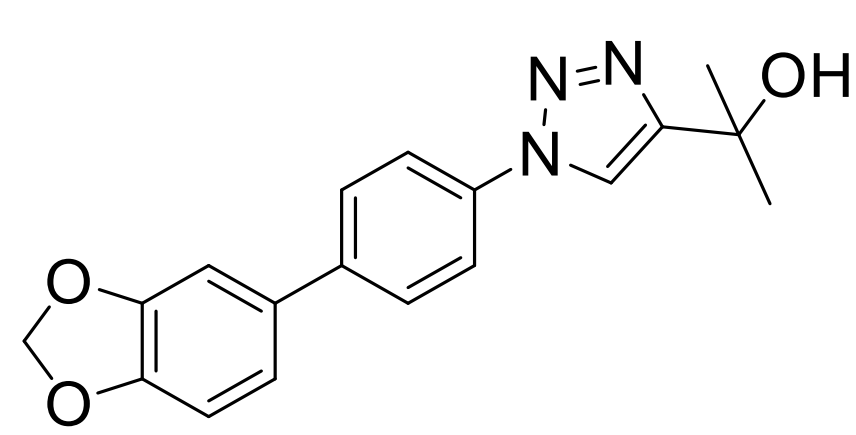

YKJ-87

2-(1-(4-(Benzo[*d*][1,3]dioxol-5-yl)phenyl)-1*H*-1,2,3-triazol-4-yl)propan-2-ol

Yield: 90.7 % ^{1}H NMR (400 MHz, DMSO) δ 8.64 (s, 1H), 8.01 – 7.90 (m, 2H), 7.89 – 7.76 (m, 2H), 7.35 (d, *J* = 1.9 Hz, 1H), 7.24 (dd, *J* = 8.1, 1.9 Hz, 1H), 7.03 (d, *J* = 8.1 Hz, 1H), 6.08 (s, 2H), 5.26 (s, 1H), 1.54 (s, 6H). ^{13}C NMR (101 MHz, DMSO) δ 156.93, 148.11, 147.24, 139.68, 135.63, 133.04, 127.65, 120.45, 120.13, 118.74, 108.77, 107.12, 101.28, 67.00, 30.58. ESI–MS calcd for $C_{18}H_{18}N_3O_3$ $[M + H]^+$ 324.1343; found 324.1345

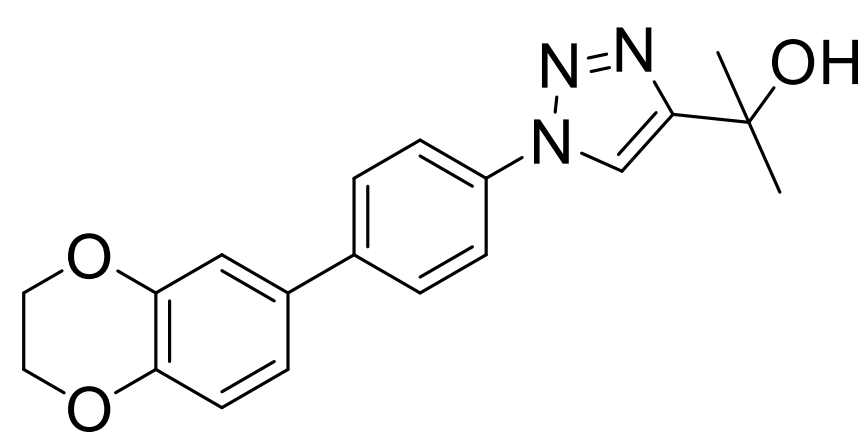

YKJ-88

2-(1-(4-(2,3-Dihydrobenzo[*b*][1,4]dioxin-6-yl)phenyl)-1*H*-1,2,3-triazol-4-yl)propan-2-ol

Yield: 67.1 % ^{1}H NMR (400 MHz, DMSO) δ 8.63 (s, 1H), 7.98 – 7.92 (m, 2H), 7.83 – 7.77 (m, 2H), 7.27 – 7.19 (m, 2H), 6.97 (d, *J* = 8.3 Hz, 1H), 5.25 (s, 1H), 4.29 (s, 4H), 1.54 (s, 6H). ^{13}C NMR (101 MHz, DMSO) δ 156.91, 143.78, 143.52, 139.40, 135.58, 132.06, 127.45, 120.17, 119.59, 118.73, 117.62, 115.18, 66.99, 64.20, 64.13, 30.58. ESI–MS calcd for $C_{19}H_{20}N_3O_3$ $[M + H]^+$ 338.1499; found 338.1501

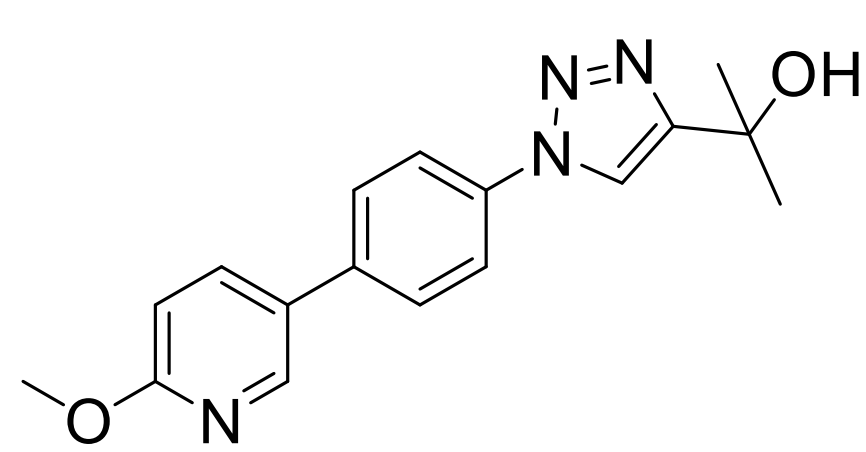

YKJ-89

2-(1-(4-(6-Methoxypyridin-3-yl)phenyl)-1*H*-1,2,3-triazol-4-yl)propan-2-ol

Yield: quantitative ^{1}H NMR (400 MHz, DMSO) δ 8.66 (s, 1H), 8.60 – 8.57 (m, 1H), 8.11 (dd, *J* = 8.7, 2.6 Hz, 1H), 8.05 – 7.98 (m, 2H), 7.92 – 7.87 (m, 2H), 6.95 (dd, *J* = 8.7, 0.7 Hz, 1H), 5.27 (s, 1H), 3.91 (s, 3H), 1.55 (s, 6H). ^{13}C NMR (101 MHz, DMSO) δ 163.33, 156.98, 144.86, 137.58, 136.85, 135.97, 128.04, 127.58, 120.30, 118.77, 110.71, 67.02, 53.36, 30.59. ESI–MS calcd for $C_{17}H_{19}N_4O_2$ $[M + H]^+$ 311.1503; found 311.1507

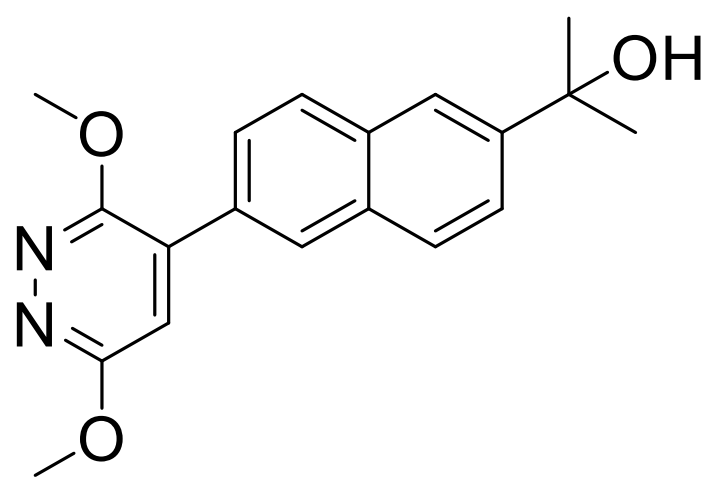

YKJ-90

2-(6-(3,6-Dimethoxypyridazin-4-yl)naphthalen-2-yl)propan-2-ol

Yield: 59.5% ^{1}H NMR (400 MHz, DMSO) δ 8.21 (d, *J* = 1.8 Hz, 1H), 8.01 (d, *J* = 1.9 Hz, 1H), 7.98 (d, *J* = 8.6 Hz, 1H), 7.92 (d, *J* = 8.7 Hz, 1H), 7.74 (dd, *J* = 8.5, 1.8 Hz, 1H), 7.70 (dd, *J* = 8.6, 1.8 Hz, 1H), 7.35 (s, 1H), 5.22 (s, 1H), 4.02 (s, 3H), 4.01 (s, 3H), 1.52 (s, 6H). ^{13}C NMR (101 MHz, DMSO) δ 162.41, 159.13, 149.45, 133.28, 132.73, 131.16, 129.88, 128.41, 127.94 (two peaks overlap), 126.26, 124.80, 121.97, 119.09, 70.84, 54.58, 54.23, 31.78. ESI–MS calcd for $C_{19}H_{21}N_2O_3$ $[M + H]^+$ 325.1547; found 325.1547

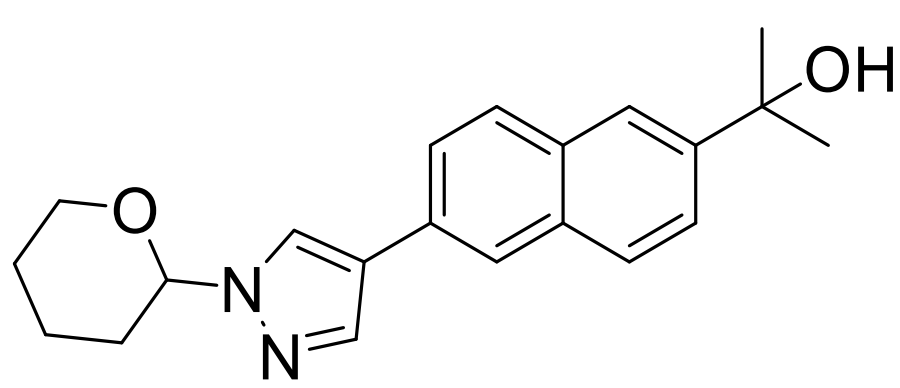

YKJ-91

2-(6-(1-(Tetrahydro-2*H*-pyran-2-yl)-1*H*-pyrazol-4-yl)naphthalen-2-yl)propan-2-ol

Yield: 81.0% ^{1}H NMR (400 MHz, DMSO) δ 8.50 – 8.45 (m, 1H), 8.13 – 8.09 (m, 1H), 8.07 (d, *J* = 0.8 Hz, 1H), 7.91 (d, *J* = 1.8 Hz, 1H), 7.88 (d, *J* = 8.6 Hz, 1H), 7.82 – 7.74 (m, 2H), 7.62 (dd, *J* = 8.6, 1.8 Hz, 1H), 5.43 (dd, *J* = 10.0, 2.1 Hz, 1H), 5.13 (s, 1H), 4.01 – 3.90 (m, 1H), 3.72 – 3.59 (m, 1H), 2.22 – 2.09 (m, 1H), 2.02 – 1.92 (m, 2H), 1.77 – 1.63 (m, 1H), 1.61 – 1.53 (m, 2H), 1.51 (s, 6H). ^{13}C NMR (101 MHz, DMSO) δ 147.53, 136.52, 132.05, 131.43, 129.24, 128.46, 126.97, 126.00, 124.55, 124.23, 122.37, 122.22, 122.09, 86.94, 70.75, 66.83, 31.80, 29.83, 24.66, 22.00. ESI–MS calcd for $C_{21}H_{25}N_2O_2$ $[M + H]^+$ 337.1911; found 337.1913

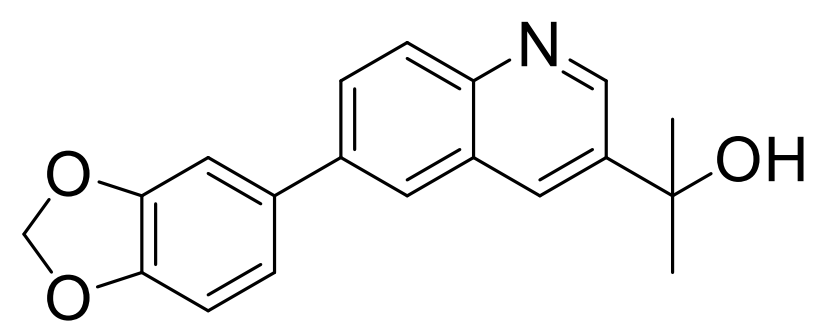

YKJ-92

2-(6-(Benzo[*d*][1,3]dioxol-5-yl)quinolin-3-yl)propan-2-ol

Yield: 96.6% ^{1}H NMR (400 MHz, DMSO) δ 9.02 (d, *J* = 2.3 Hz, 1H), 8.36 (d, *J* = 2.3 Hz, 1H), 8.20 (d, *J* = 1.9 Hz, 1H), 8.05 – 7.95 (m, 2H), 7.43 (d, *J* = 1.8 Hz, 1H), 7.32 (dd, *J* = 8.1, 1.9 Hz, 1H), 7.06 (d, *J* = 8.1 Hz, 1H), 6.10 (s, 2H), 5.39 (s, 1H), 1.57 (s, 6H). ^{13}C NMR (101 MHz, DMSO) δ 149.10, 148.13, 147.17, 145.49, 143.27, 137.72, 133.69, 130.39, 128.90, 127.89, 127.52, 124.91, 120.76, 108.79, 107.39, 101.25, 69.88, 31.55. ESI–MS calcd for $C_{19}H_{18}NO_3$ $[M + H]^+$ 308.1281 found 308.1283

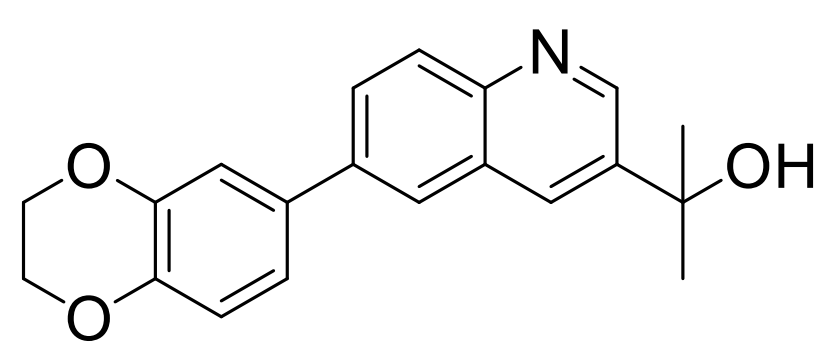

YKJ-93

2-(6-(2,3-Dihydrobenzo[*b*][1,4]dioxin-6-yl)quinolin-3-yl)propan-2-ol

Yield: quantitative ^{1}H NMR (400 MHz, DMSO) δ 9.02 (d, *J* = 2.3 Hz, 1H), 8.36 (d, *J* = 2.3 Hz, 1H), 8.19 (d, *J* = 2.0 Hz, 1H), 8.04 – 7.94 (m, 2H), 7.37 – 7.28 (m, 2H), 6.99 (d, *J* = 8.3 Hz, 1H), 5.38 (s, 1H), 4.30 (s, 4H), 1.57 (s, 6H). ^{13}C NMR (101 MHz, DMSO) δ 149.06, 145.48, 143.80, 143.45, 143.24, 137.44, 132.70, 130.40, 128.91, 127.71, 127.54, 124.68, 119.90, 117.63, 115.50, 69.89, 64.21, 64.14, 31.55. ESI–MS calcd for $C_{20}H_{20}NO_3$ $[M + H]^+$ 322.1438; found 322.1442

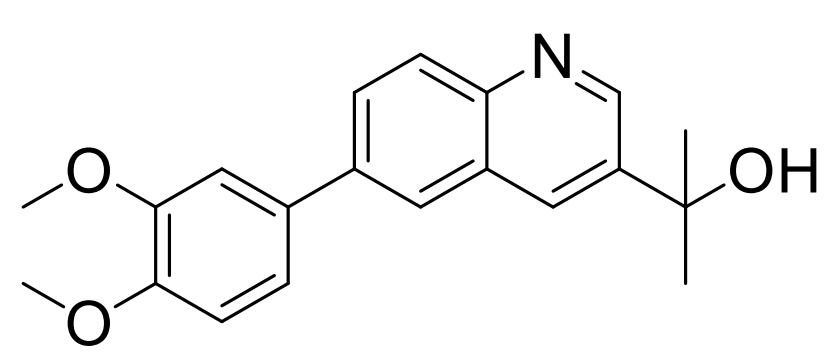

YKJ-94

2-(6-(3,4-Dimethoxyphenyl)quinolin-3-yl)propan-2-ol

Yield: quantitative ^{1}H NMR (400 MHz, DMSO) δ 9.02 (d, *J* = 2.3 Hz, 1H), 8.38 (d, *J* = 2.3 Hz, 1H), 8.28 – 8.24 (m, 1H), 8.12 – 7.97 (m, 2H), 7.46 – 7.33 (m, 2H), 7.09 (d, *J* = 8.4 Hz, 1H), 5.38 (s, 1H), 3.89 (s, 3H), 3.82 (s, 3H), 1.57 (s, 6H). ^{13}C NMR (101 MHz, DMSO) δ 149.19, 148.97, 148.83, 145.46, 143.21, 137.90, 131.53, 131.43, 128.82, 128.70, 127.88, 124.68, 119.25, 112.25, 110.63, 69.90, 55.61 (two peaks overlap), 31.57. ESI–MS calcd for $C_{20}H_{22}NO_3$ $[M + H]^+$ 324.1594; found 324.1599

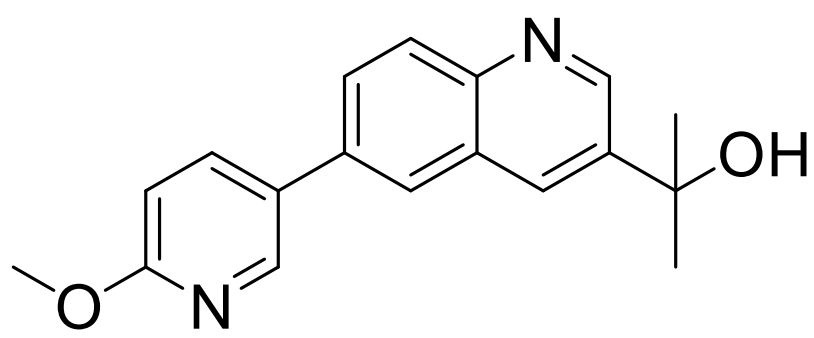

YKJ-95

2-(6-(6-Methoxypyridin-3-yl)quinolin-3-yl)propan-2-ol

Yield: 91.8% ^{1}H NMR (400 MHz, DMSO) δ 9.05 (d, *J* = 2.3 Hz, 1H), 8.66 (dd, *J* = 2.6, 0.7 Hz, 1H), 8.38 (d, *J* = 2.3 Hz, 1H), 8.27 (d, *J* = 1.9 Hz, 1H), 8.18 (dd, *J* = 8.6, 2.6 Hz, 1H), 8.12 – 8.00 (m, 2H), 6.98 (dd, *J* = 8.6, 0.7 Hz, 1H), 5.41 (s, 1H), 3.92 (s, 3H), 1.57 (s, 6H). ^{13}C NMR (101 MHz, DMSO) δ 163.27, 149.33, 145.58, 145.13, 143.40, 137.82, 134.91, 131.52, 131.43, 128.81, 128.69, 127.56, 124.97, 110.76, 69.90, 53.36, 31.54. ESI–MS calcd for $C_{18}H_{19}N_2O_2$ $[M + H]^+$ 295.1441; found 295.1442

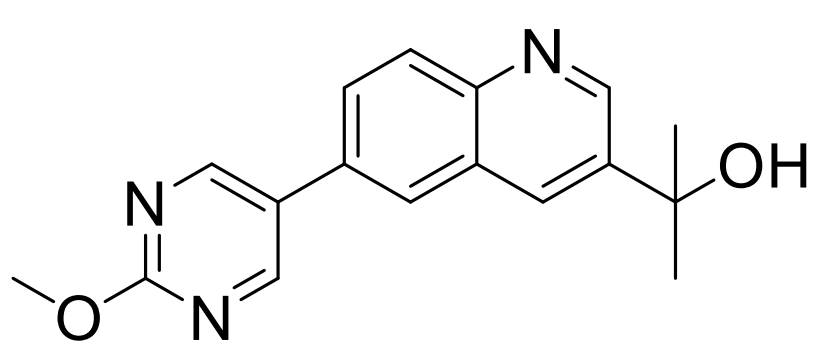

YKJ-96

2-(6-(2-Methoxypyrimidin-5-yl)quinolin-3-yl)propan-2-ol

Yield: 83.6% ^{1}H NMR (400 MHz, DMSO) δ 9.10 (s, 2H), 9.07 (d, *J* = 2.3 Hz, 1H), 8.39 – 8.35 (m, 2H), 8.09 (d, *J* = 1.4 Hz, 2H), 5.43 (s, 1H), 4.00 (s, 3H), 1.57 (s, 6H). ^{13}C NMR (101 MHz, DMSO) δ 164.72, 157.68, 149.71, 145.79, 143.59, 131.87, 130.46, 129.39, 127.54, 127.27, 126.86, 125.36, 69.91, 54.83, 31.54. ESI–MS calcd for $C_{17}H_{18}N_3O_2$ $[M + H]^+$ 296.1394 found 296.1394

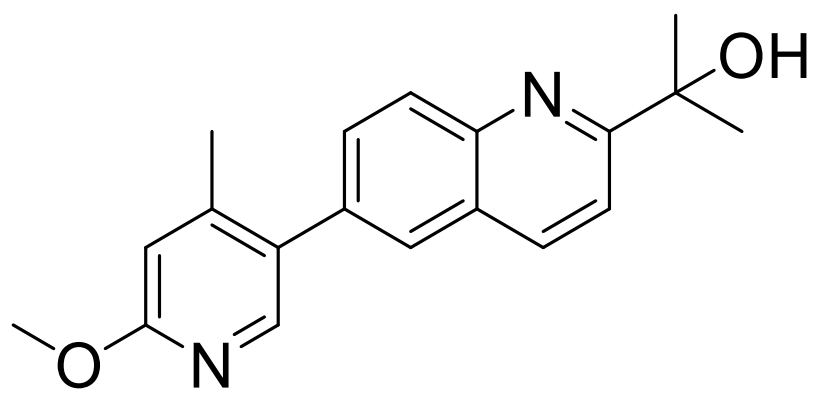

YKJ-97

2-(6-(6-Methoxy-4-methylpyridin-3-yl)quinolin-2-yl)propan-2-ol

Yield: 99.5% ^{1}H NMR (400 MHz, DMSO) δ 8.37 (d, *J* = 8.6 Hz, 1H), 8.09 (s, 1H), 8.01 (d, *J* = 8.6 Hz, 1H), 7.93 – 7.88 (m, 2H), 7.72 (dd, *J* = 8.6, 2.1 Hz, 1H), 6.83 (s, 1H), 5.46 (s, 1H), 3.89 (s, 3H), 2.27 (s, 3H), 1.56 (s, 6H). ^{13}C NMR (101 MHz, DMSO) δ 168.52, 163.16, 147.72, 146.58, 145.34, 136.55, 135.01, 131.05, 130.74, 128.49, 127.82, 126.49, 118.14, 110.95, 72.69, 53.13, 30.50, 19.79. ESI–MS calcd for $C_{19}H_{21}N_2O_2$ $[M + H]^+$ 309.1598 found 309.1598

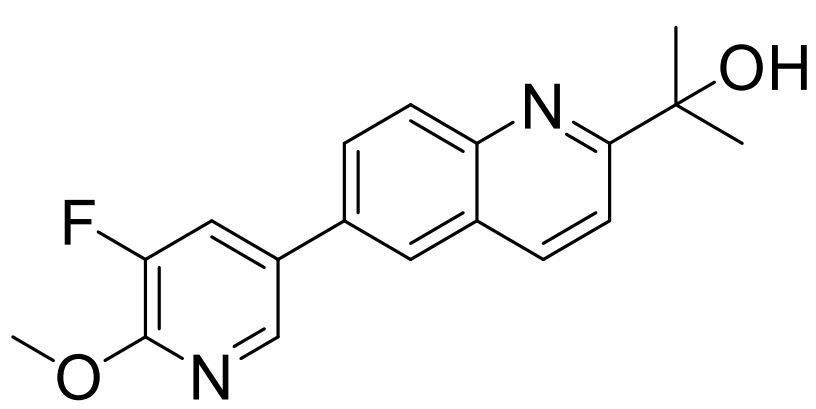

YKJ-98

2-(6-(5-Fluoro-6-methoxypyridin-3-yl)quinolin-2-yl)propan-2-ol

Yield: 79.7% ^{1}H NMR (400 MHz, DMSO) δ 8.50 (d, *J* = 2.1 Hz, 1H), 8.39 – 8.34 (m, 1H), 8.31 (d, *J* = 2.2 Hz, 1H), 8.23 (dd, *J* = 11.9, 2.1 Hz, 1H), 8.10 (dd, *J* = 8.8, 2.2 Hz, 1H), 8.03 (d, *J* = 8.8 Hz, 1H), 7.90 (d, *J* = 8.6 Hz, 1H), 5.46 (s, 1H), 4.02 (s, 3H), 1.55 (s, 6H). ^{13}C NMR (126 MHz, DMSO) δ 168.69, 152.04 (d, *J* = 11.4 Hz), 146.92 (d, *J* = 257.0 Hz), 145.76, 139.48 (d, *J* = 5.6 Hz), 136.72, 133.12, 129.73 (d, *J* = 1.9 Hz), 129.37, 128.06, 126.83, 125.15, 122.33 (d, *J* = 16.1 Hz), 118.44, 72.73, 53.80, 30.48. ESI–MS calcd for $C_{18}H_{18}FN_2O_2$ $[M + H]^+$ 313.1347; found 313.1347

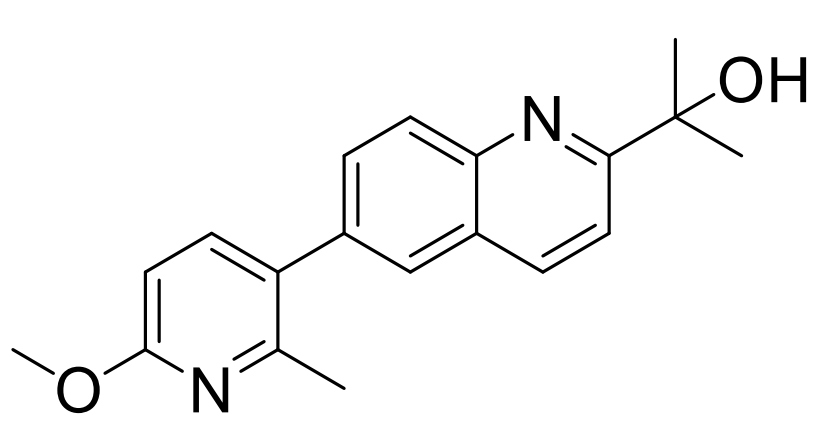

YKJ-99

2-(6-(6-Methoxy-2-methylpyridin-3-yl)quinolin-2-yl)propan-2-ol

Yield: quantitative ^{1}H NMR (400 MHz, DMSO) δ 8.36 (d, *J* = 8.6 Hz, 1H), 8.01 (d, *J* = 8.5 Hz, 1H), 7.92 – 7.86 (m, 2H), 7.75 – 7.69 (m, 1H), 7.65 (d, *J* = 8.3 Hz, 1H), 6.76 (d, *J* = 8.3 Hz, 1H), 5.47 (s, 1H), 3.90 (s, 3H), 2.41 (s, 3H), 1.56 (s, 6H). ^{13}C NMR (101 MHz, DMSO) δ 168.93, 162.62, 153.19, 145.68, 141.21, 137.37, 136.99, 131.39, 129.17, 128.98, 128.07, 126.95, 118.63, 108.23, 73.15, 53.56, 30.97, 23.48. ESI–MS calcd for $C_{19}H_{21}N_2O_2$ $[M + H]^+$ 309.1598 found 309.1596

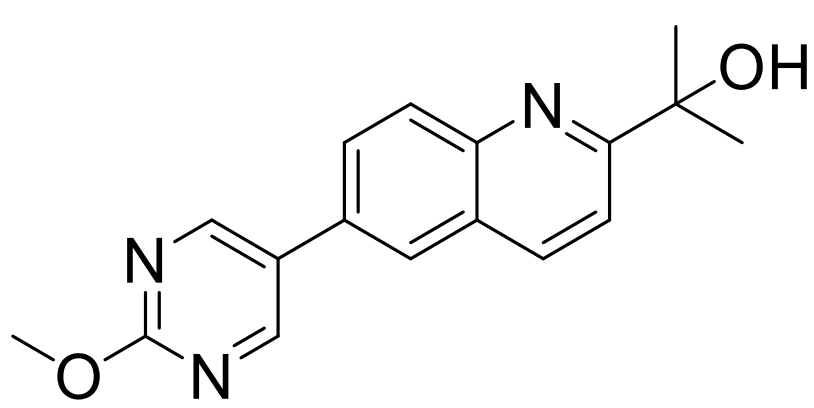

YKJ-100

2-(6-(2-Methoxypyrimidin-5-yl)quinolin-2-yl)propan-2-ol

Yield: 98.1% ^{1}H NMR (400 MHz, DMSO) δ 9.06 (s, 2H), 8.34 (d, *J* = 8.7 Hz, 1H), 8.30 (d, *J* = 2.1 Hz, 1H), 8.08 (dd, *J* = 8.8, 2.1 Hz, 1H), 8.02 (d, *J* = 8.8 Hz, 1H), 7.88 (d, *J* = 8.7 Hz, 1H), 5.43 (s, 1H), 3.96 (s, 3H), 1.52 (s, 6H). ^{13}C NMR (101 MHz, DMSO) δ 168.78, 164.67, 157.60, 145.78, 136.68, 131.27, 129.53, 127.75, 126.84, 126.78, 124.91, 118.49, 72.72, 54.81, 30.46. ESI–MS calcd for $C_{17}H_{18}N_3O_2$ $[M + H]^+$ 296.1394; found 296.1394

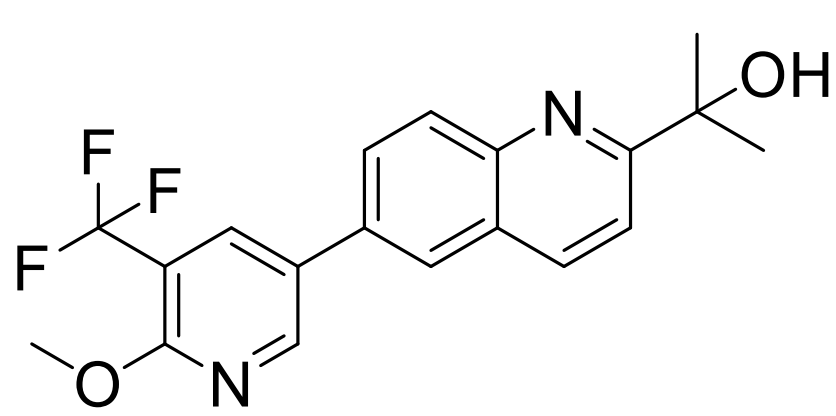

YKJ-101

2-(6-(6-Methoxy-5-(trifluoromethyl)pyridin-3-yl)quinolin-2-yl)propan-2-ol

Yield: 93.7% ^{1}H NMR (400 MHz, DMSO) δ 8.93 (d, *J* = 2.4 Hz, 1H), 8.50 (d, *J* = 2.7 Hz, 1H), 8.41 – 8.35 (m, 2H), 8.15 (dd, *J* = 8.8, 2.2 Hz, 1H), 8.05 (d, *J* = 8.8 Hz, 1H), 7.91 (d, *J* = 8.6 Hz, 1H), 5.46 (s, 1H), 4.06 (s, 3H), 1.56 (s, 6H). ^{13}C NMR (126 MHz, DMSO) δ 168.80, 159.54 (d, *J* = 1.3 Hz), 149.20, 145.80, 136.78, 135.45 (q, *J* = 5.0 Hz), 132.84, 129.44, 128.47, 128.10, 126.85,

125.42, 123.11 (q, J = 275.9 Hz), 118.46, 111.69 (q, J = 32.8 Hz), 72.75, 54.40, 30.48. ESI–MS calcd for $C_{19}H_{18}F_3N_2O_2$ $[M + H]^+$ 363.1315; found 363.1315

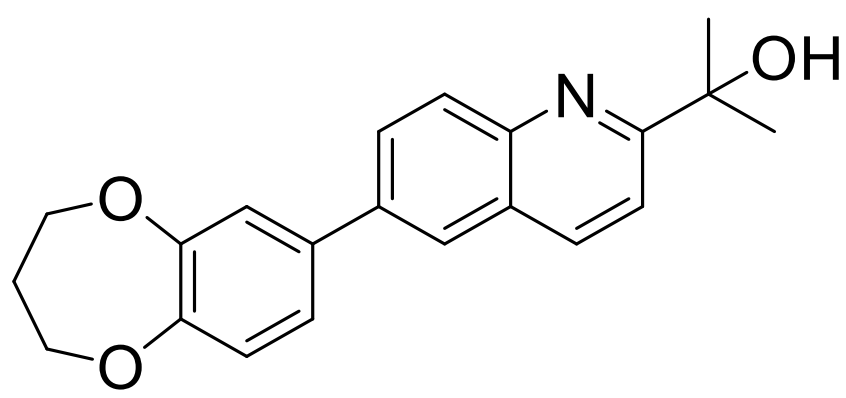

YKJ-102

2-(6-(3,4-Dihydro-2H-benzo[*b*][1,4]dioxepin-7-yl)quinolin-2-yl)propan-2-ol

Yield: 72.4% ^{1}H NMR (400 MHz, DMSO) δ 8.36 (d, J = 8.7 Hz, 1H), 8.20 (d, J = 2.0 Hz, 1H), 8.04 – 7.96 (m, 2H), 7.87 (d, J = 8.7 Hz, 1H), 7.46 – 7.39 (m, 2H), 7.09 (d, J = 8.2 Hz, 1H), 5.44 (s, 1H), 4.19 (q, J = 5.7 Hz, 4H), 2.15 (pentet, J = 5.4 Hz, 2H), 1.55 (s, 6H). ^{13}C NMR (101 MHz, DMSO) δ 168.20, 151.38, 150.87, 145.47, 136.69, 136.43, 134.66, 129.10, 128.17, 126.86, 124.55, 122.14, 121.80, 119.93, 118.16, 72.65, 70.52, 31.49, 30.47. ESI–MS calcd for $C_{21}H_{22}NO_3$ $[M + H]^+$ 336.1594; found 336.1598

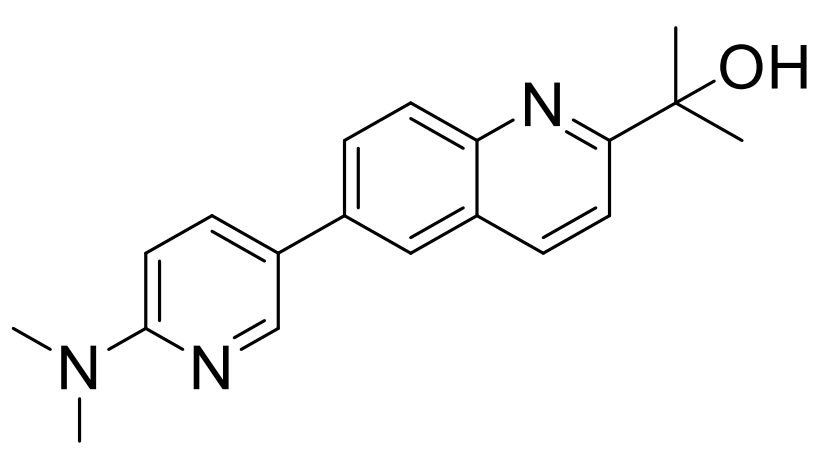

YKJ-103

2-(6-(6-(Dimethylamino)pyridin-3-yl)quinolin-2-yl)propan-2-ol

Yield: 57.9% ^{1}H NMR (400 MHz, DMSO) δ 8.60 (d, J = 2.5 Hz, 1H), 8.33 (d, J = 8.7 Hz, 1H), 8.15 (d, J = 2.0 Hz, 1H), 8.05 – 7.94 (m, 3H), 7.85 (d, J = 8.6 Hz, 1H), 6.77 (d, J = 8.9 Hz, 1H), 5.44 (s, 1H), 3.09 (s, 6H), 1.55 (s, 6H). ^{13}C NMR (101 MHz, DMSO) δ 167.70, 158.45, 145.88, 145.15, 136.39, 135.57, 135.25, 129.15, 127.60, 127.04, 122.90, 122.41, 118.12, 105.87, 72.62, 37.70, 30.48. ESI–MS calcd for $C_{19}H_{22}N_3O$ $[M + H]^+$ 308.1757; found 308.1762

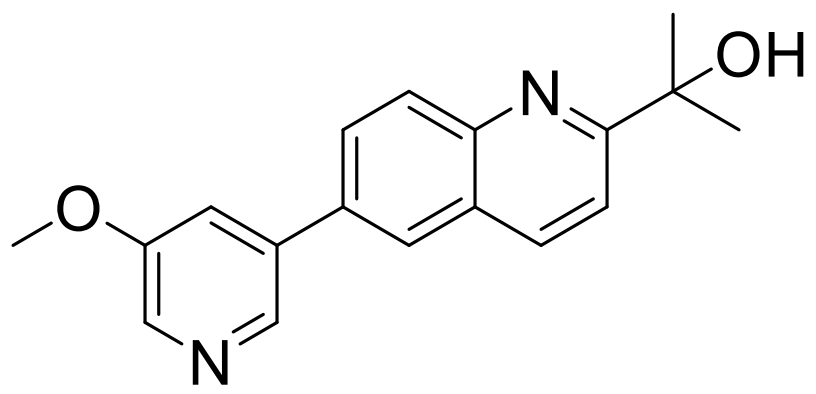

YKJ-104

2-(6-(5-Methoxypyridin-3-yl)quinolin-2-yl)propan-2-ol

Yield: quantitative ^{1}H NMR (400 MHz, DMSO) δ 8.66 (s, 1H), 8.44 – 8.31 (m, 3H), 8.18 – 8.04 (m, 2H), 7.91 (d, *J* = 8.6 Hz, 1H), 7.80 (s, 1H), 5.47 (s, 1H), 3.95 (s, 3H), 1.56 (s, 6H). ^{13}C NMR (101 MHz, DMSO) δ 168.84, 155.72, 145.95, 140.04, 136.84 (two peaks overlap), 135.68, 134.17, 129.38, 128.45, 126.79, 125.88, 118.42 (two peaks overlap), 72.72, 55.72, 30.46. ESI–MS calcd for $C_{18}H_{19}N_2O_2$ $[M + H]^+$ 295.1441 found 295.1441

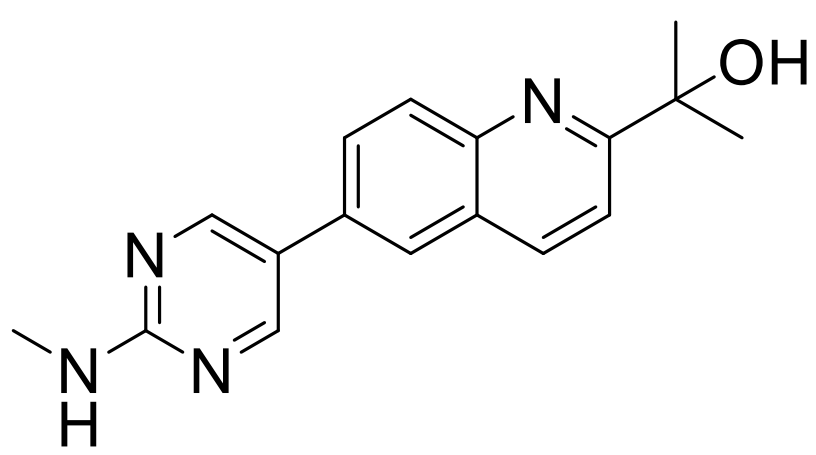

YKJ-105

2-(6-(2-(Methylamino)pyrimidin-5-yl)quinolin-2-yl)propan-2-ol

Yield: quantitative ^{1}H NMR (400 MHz, DMSO) δ 8.79 (s, 2H), 8.33 (d, *J* = 8.7 Hz, 1H), 8.20 (d, *J* = 2.0 Hz, 1H), 8.07 – 7.97 (m, 2H), 7.87 (d, *J* = 8.6 Hz, 1H), 7.34 (q, *J* = 4.8 Hz, 1H), 5.45 (s, 1H), 2.87 (d, *J* = 4.8 Hz, 3H), 1.54 (s, 6H). ^{13}C NMR (101 MHz, DMSO) δ 168.02, 162.17, 145.32, 136.43, 131.54, 131.44, 129.33, 128.83, 128.72, 127.25, 122.95, 118.27, 72.67, 30.49, 27.98. ESI–MS calcd for $C_{17}H_{19}N_4O$ $[M + H]^+$ 295.1553 found 295.1553

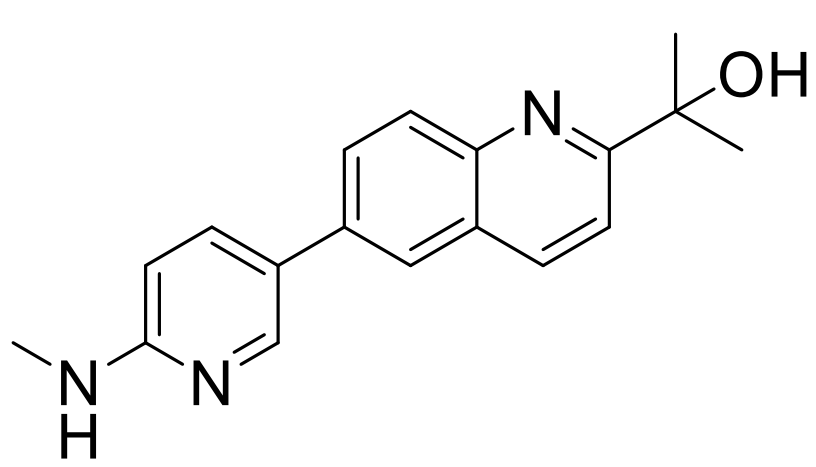

YKJ-106

2-(6-(6-(Methylamino)pyridin-3-yl)quinolin-2-yl)propan-2-ol

Yield: quantitative ^{1}H NMR (400 MHz, DMSO) δ 8.50 (d, *J* = 2.5 Hz, 1H), 8.33 (d, *J* = 8.7 Hz, 1H), 8.12 (s, 1H), 8.03 – 7.95 (m, 2H), 7.91 – 7.81 (m, 2H), 6.72 (q, *J* = 5.1 Hz, 1H), 6.58 (d, *J* = 8.7 Hz, 1H), 5.44 (s, 1H), 2.83 (d, *J* = 4.8 Hz, 3H), 1.54 (s, 6H). ^{13}C NMR (101 MHz, DMSO) δ 167.63, 159.02, 146.05, 145.11, 136.39, 135.56, 135.11, 131.55, 131.45, 129.13, 128.84, 128.73, 122.73, 118.11, 72.64, 30.50, 28.02. ESI–MS calcd for $C_{18}H_{20}N_3O$ $[M + H]^+$ 294.1601 found 294.1598

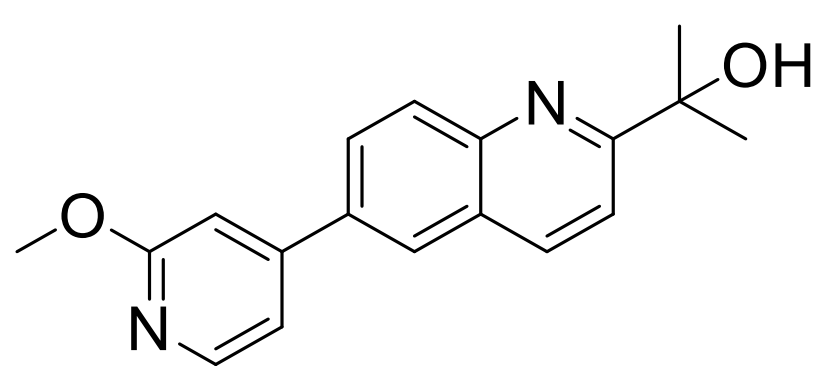

YKJ-107

2-(6-(2-Methoxypyridin-4-yl)quinolin-2-yl)propan-2-ol

Yield: 59.9% ^{1}H NMR (400 MHz, DMSO) δ 8.46 – 8.40 (m, 2H), 8.29 (d, *J* = 5.4 Hz, 1H), 8.14 (dd, *J* = 8.8, 2.1 Hz, 1H), 8.06 (d, *J* = 8.8 Hz, 1H), 7.92 (d, *J* = 8.7 Hz, 1H), 7.48 (dd, *J* = 5.4, 1.6 Hz, 1H), 7.28 (d, *J* = 1.5 Hz, 1H), 5.47 (s, 1H), 3.92 (s, 3H), 1.55 (s, 6H). ^{13}C NMR (101 MHz, DMSO) δ 169.27, 164.57, 149.52, 147.63, 146.44, 137.08, 134.30, 129.44, 127.90, 126.68, 126.18, 118.48, 115.23, 107.73, 72.74, 53.33, 30.44. ESI–MS calcd for $C_{18}H_{19}N_2O_2$ $[M + H]^+$ 295.1441; found 295.1441

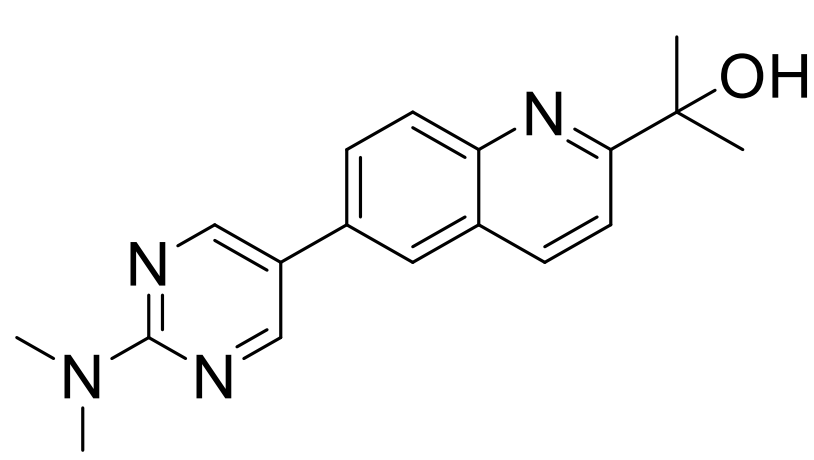

YKJ-108

2-(6-(2-(Dimethylamino)pyrimidin-5-yl)quinolin-2-yl)propan-2-ol

Yield: 84.1% ^{1}H NMR (400 MHz, DMSO) δ 8.85 (s, 2H), 8.33 (d, *J* = 8.7 Hz, 1H), 8.20 (d, *J* = 2.0 Hz, 1H), 8.08 – 7.98 (m, 2H), 7.87 (d, *J* = 8.6 Hz, 1H), 5.44 (s, 1H), 3.19 (s, 6H), 1.55 (s, 6H). ^{13}C NMR (101 MHz, DMSO) δ 168.04, 161.20, 155.85, 145.34, 136.40, 132.59, 129.34, 127.24, 126.97, 123.03, 120.28, 118.26, 72.64, 36.75, 30.46. ESI–MS calcd for $C_{18}H_{21}N_4O$ $[M + H]^+$ 309.1710 found 309.1715

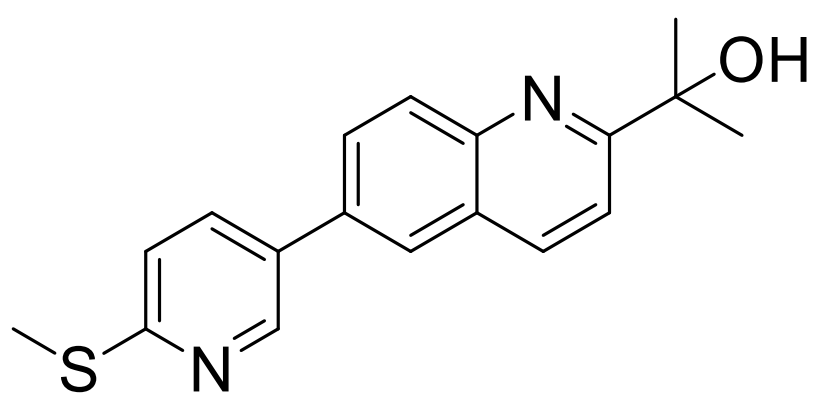

YKJ-109

2-(6-(6-(Methylthio)pyridin-3-yl)quinolin-2-yl)propan-2-ol

Yield: 98.0% ^{1}H NMR (400 MHz, DMSO) δ 8.93 (d, *J* = 2.4 Hz, 1H), 8.38 (d, *J* = 8.6 Hz, 1H), 8.30 (d, *J* = 2.1 Hz, 1H), 8.15 – 7.99 (m, 3H), 7.90 (d, *J* = 8.6 Hz, 1H), 7.44 (d, *J* = 8.5 Hz, 1H), 5.46 (s, 1H), 2.58 (s, 3H), 1.56 (s, 6H). ^{13}C NMR (101 MHz, DMSO) δ 168.59, 158.68, 147.41, 145.74, 136.70, 134.71, 134.02, 130.63, 129.42, 127.95, 126.86, 124.94, 121.07, 118.36, 72.68, 30.45, 12.83. ESI–MS calcd for $C_{18}H_{19}N_2OS$ $[M + H]^+$ 311.1213 found 311.1216

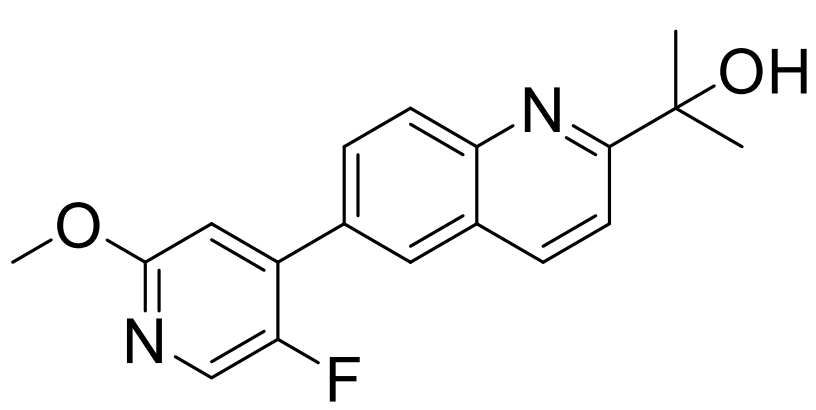

YKJ-110

2-(6-(5-Fluoro-2-methoxypyridin-4-yl)quinolin-2-yl)propan-2-ol

Yield: 38.6% ^{1}H NMR (400 MHz, DMSO) δ 8.44 (d, *J* = 8.7 Hz, 1H), 8.33 – 8.25 (m, 2H), 8.07 (d, *J* = 8.8 Hz, 1H), 7.99 – 7.91 (m, 2H), 7.15 (d, *J* = 5.4 Hz, 1H), 5.48 (s, 1H), 3.90 (s, 3H), 1.56 (s, 6H). ^{13}C NMR (126 MHz, DMSO) δ 169.94, 160.87 (d, *J* = 1.3 Hz), 152.90 (d, *J* = 245.4 Hz), 146.56, 139.05 (d, *J* = 13.4 Hz), 137.73, 134.68 (d, *J* = 28.3 Hz), 130.48 (d, *J* = 1.4 Hz), 129.97 (d, *J* = 3.2 Hz), 129.51, 128.78 (d, *J* = 3.3 Hz), 126.86, 118.95, 110.96, 73.31, 54.25, 30.73. ESI–MS calcd for $C_{18}H_{18}FN_2O_2$ $[M + H]^+$ 313.1347; found 313.1348

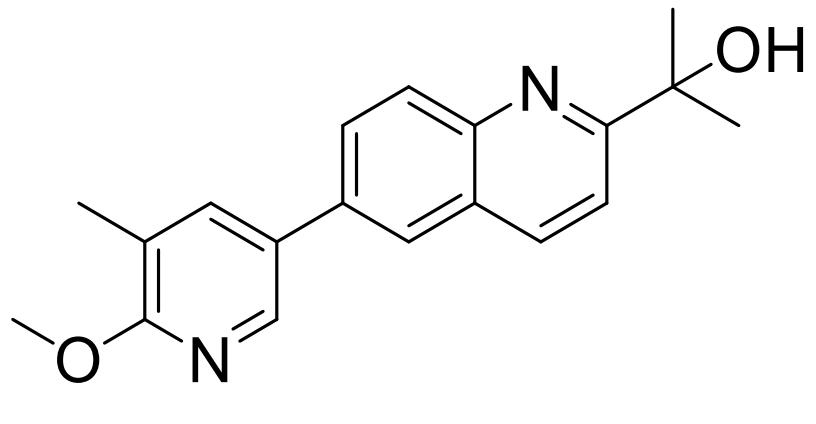

YKJ-111

2-(6-(6-Methoxy-5-methylpyridin-3-yl)quinolin-2-yl)propan-2-ol

Yield: 33.4% ^{1}H NMR (400 MHz, DMSO) δ 8.47 (d, *J* = 2.5 Hz, 1H), 8.36 (d, *J* = 8.7 Hz, 1H), 8.23 (d, *J* = 2.0 Hz, 1H), 8.08 – 7.99 (m, 3H), 7.88 (d, *J* = 8.6 Hz, 1H), 5.45 (s, 1H), 3.95 (s, 3H), 2.25 (s, 3H), 1.55 (s, 6H). ^{13}C NMR (101 MHz, DMSO) δ 168.26, 161.52, 145.50, 142.06, 137.42, 136.58, 134.51, 129.26, 128.46, 128.04, 126.90, 124.40, 120.19, 118.27, 72.67, 53.38, 30.47, 15.53. ESI–MS calcd for $C_{19}H_{21}N_2O_2$ $[M + H]^+$ 309.1598; found 309.1598

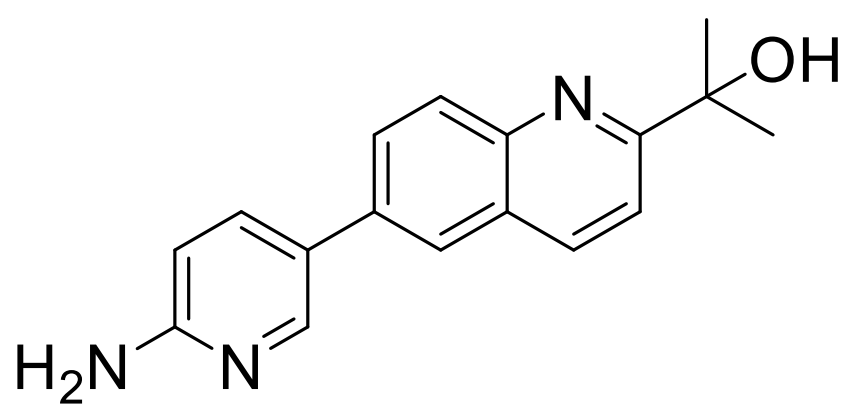

YKJ-112

2-(6-(6-Aminopyridin-3-yl)quinolin-2-yl)propan-2-ol

Yield: 90.4% ^{1}H NMR (400 MHz, DMSO) δ 8.42 (d, *J* = 2.6 Hz, 1H), 8.33 (d, *J* = 8.7 Hz, 1H), 8.12 (s, 1H), 7.98 (s, 2H), 7.91 – 7.79 (m, 2H), 6.58 (d, *J* = 8.6 Hz, 1H), 6.16 (s, 2H), 5.43 (s, 1H), 1.54 (s, 6H). ^{13}C NMR (101 MHz, DMSO) δ 167.62, 159.37, 146.17, 145.09, 136.37, 135.50, 129.10, 127.56, 127.02, 123.00, 122.74, 118.08, 108.05, 72.61, 30.48 (one low field carbon not observed). ESI–MS calcd for $C_{17}H_{18}N_3O$ $[M + H]^+$ 280.1444; found 280.1444

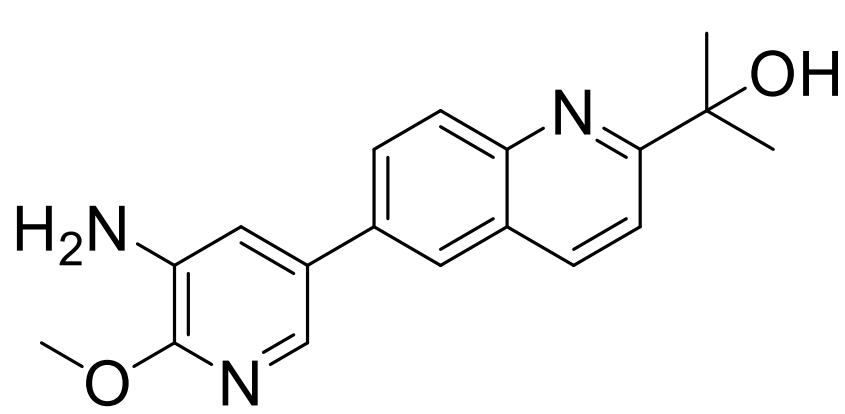

YKJ-113

2-(6-(5-Amino-6-methoxypyridin-3-yl)quinolin-2-yl)propan-2-ol

Yield: 99.6% ^{1}H NMR (400 MHz, DMSO) δ 8.37 (d, *J* = 8.7 Hz, 1H), 8.10 (d, *J* = 2.2 Hz, 1H), 8.03 – 7.92 (m, 2H), 7.87 (d, *J* = 8.6 Hz, 1H), 7.81 (d, *J* = 2.2 Hz, 1H), 7.31 (d, *J* = 2.2 Hz, 1H), 5.44 (s, 1H), 5.12 (s, 2H), 3.93 (s, 3H), 1.55 (s, 6H). ^{13}C NMR (101 MHz, DMSO) δ 168.08, 151.78, 145.43, 136.55, 135.51, 132.60, 130.57, 129.30, 129.18, 128.15, 126.87, 124.16, 118.18, 117.06, 72.64, 53.03, 30.47. ESI–MS calcd for $C_{18}H_{20}N_3O_2$ $[M + H]^+$ 310.1550; found 310.1555

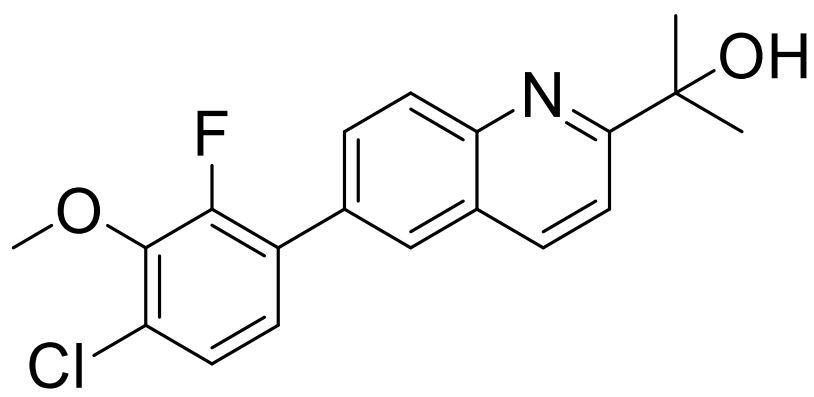

YKJ-114

2-(6-(4-Chloro-2-fluoro-3-methoxyphenyl)quinolin-2-yl)propan-2-ol

Yield: quantitative ^{1}H NMR (400 MHz, DMSO) δ 8.42 (d, *J* = 8.6 Hz, 1H), 8.15 (t, *J* = 1.7 Hz, 1H), 8.06 (d, *J* = 8.7 Hz, 1H), 7.96 – 7.88 (m, 2H), 7.49 – 7.39 (m, 2H), 5.47 (s, 1H), 3.96 (s, 3H), 1.56 (s, 6H). ^{13}C NMR (126 MHz, DMSO) δ 169.13, 153.17 (d, *J* = 250.2 Hz), 145.76, 144.34 (d, *J* = 14.3 Hz), 136.88, 131.50, 130.04 (d, *J* = 2.4 Hz), 128.97, 128.50 (d, *J* = 12.0 Hz), 127.88 (d, *J* = 3.0 Hz),126.82 (d, *J* = 3.8 Hz), 126.55, 125.60 (two peaks overlap), 118.43, 72.78, 61.62 (d, *J* = 4.1 Hz), 30.49. ESI–MS calcd for $C_{19}H_{18}ClFNO_2$ $[M + H]^+$ 346.1005; found 346.1007

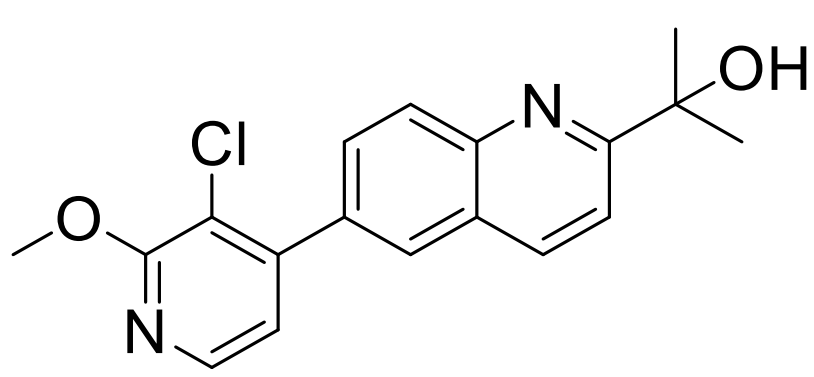

YKJ-115

2-(6-(3-Chloro-2-methoxypyridin-4-yl)quinolin-2-yl)propan-2-ol

Yield: 89.6% ^{1}H NMR (400 MHz, DMSO) δ 8.42 (d, *J* = 8.6 Hz, 1H), 8.22 (d, *J* = 5.1 Hz, 1H), 8.09 – 8.04 (m, 2H), 7.93 (d, *J* = 8.6 Hz, 1H), 7.81 (dd, *J* = 8.7, 2.1 Hz, 1H), 7.17 (d, *J* = 5.1 Hz, 1H), 5.48 (s, 1H), 4.01 (s, 3H), 1.56 (s, 6H). ^{13}C NMR (101 MHz, DMSO) δ 169.40, 159.37, 149.02, 145.90, 144.52, 136.86, 133.99, 129.88, 128.59, 128.09, 126.21, 119.48, 118.41, 115.19, 72.73, 54.41, 30.44. ESI–MS calcd for $C_{18}H_{18}ClN_2O_2$ $[M + H]^+$ 329.1051; found 329.1053

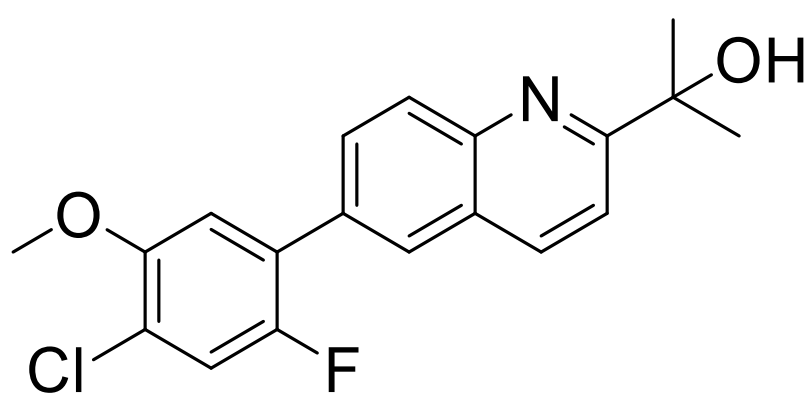

YKJ-116

2-(6-(4-Chloro-2-fluoro-5-methoxyphenyl)quinolin-2-yl)propan-2-ol

Yield: 88.8% ^{1}H NMR (400 MHz, DMSO) δ 8.41 (d, *J* = 8.6 Hz, 1H), 8.20 (s, 1H), 8.06 (d, *J* = 8.7 Hz, 1H), 7.99 – 7.90 (m, 2H), 7.61 (d, *J* = 10.1 Hz, 1H), 7.37 (d, *J* = 6.8 Hz, 1H), 5.47 (s, 1H), 3.96 (s, 3H), 1.56 (s, 6H). ^{13}C NMR (126 MHz, DMSO) δ 169.06, 152.63 (d, *J* = 243.2 Hz), 151.55 (d, *J* = 2.5 Hz), 145.76, 136.88, 131.90 (d, *J* = 1.6 Hz), 130.18 (d, *J* = 3.2 Hz), 128.89, 127.95 (d, *J* = 3.2 Hz), 127.05 (d, *J* = 14.2 Hz), 126.53, 120.94 (d, *J* = 10.7 Hz), 118.41, 117.92 (d, *J* = 27.9 Hz), 114.06 (d, *J* = 3.6 Hz), 72.80, 56.80, 30.50. ESI–MS calcd for $C_{19}H_{18}ClFNO_2$ $[M + H]^+$ 346.1005; found 346.1007

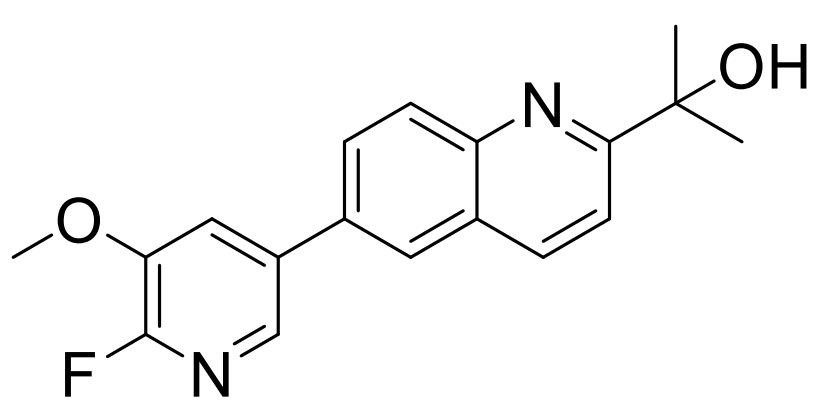

YKJ-117

2-(6-(6-Fluoro-5-methoxypyridin-3-yl)quinolin-2-yl)propan-2-ol

Yield: 96.8% ^{1}H NMR (400 MHz, DMSO) δ 8.40 (d, *J* = 8.1 Hz, 1H), 8.37 (d, *J* = 2.1 Hz, 1H), 8.19 (t, *J* = 2.0 Hz, 1H), 8.15 (dd, *J* = 8.8, 2.2 Hz, 1H), 8.08 – 8.03 (m, 2H), 7.92 (d, *J* = 8.6 Hz, 1H), 5.46 (s, 1H), 4.03 (s, 3H), 1.56 (s, 6H). ^{13}C NMR (126 MHz, DMSO) δ 168.88, 152.33 (d, *J* = 236.2 Hz), 145.91, 142.40 (d, *J* = 26.4 Hz), 136.80, 134.68 (d, *J* = 4.3 Hz), 134.47 (d, *J* = 13.9 Hz), 133.45, 129.36, 128.49, 126.77, 125.82, 121.25 (d, *J* = 4.4 Hz), 118.49, 72.75, 56.40, 30.47. ESI–MS calcd for $C_{18}H_{18}FN_2O_2$ $[M + H]^+$ 313.1347 found 313.1349

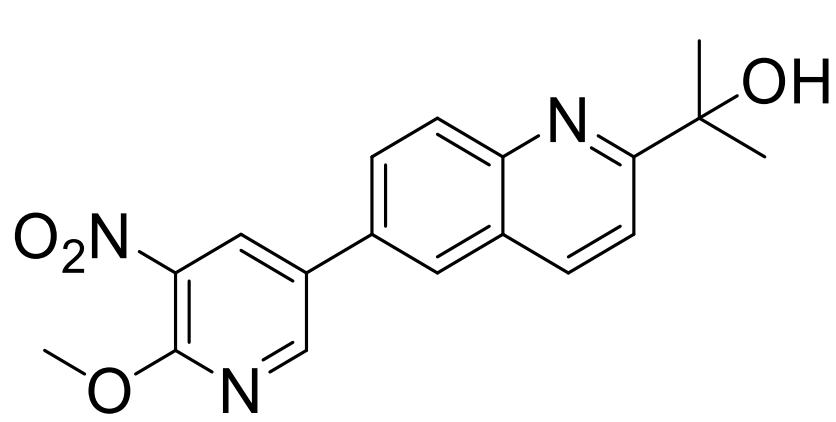

YKJ-118

2-(6-(6-Methoxy-5-nitropyridin-3-yl)quinolin-2-yl)propan-2-ol

Yield: 88.9% ^{1}H NMR (400 MHz, DMSO) δ 9.02 (d, *J* = 2.4 Hz, 1H), 8.87 (d, *J* = 2.4 Hz, 1H), 8.42 – 8.35 (m, 2H), 8.16 (dd, *J* = 8.8, 2.2 Hz, 1H), 8.06 (d, *J* = 8.8 Hz, 1H), 7.92 (d, *J* = 8.7 Hz, 1H), 5.46 (s, 1H), 4.10 (s, 3H), 1.56 (s, 6H). ^{13}C NMR (101 MHz, DMSO) δ 168.96, 154.81,

149.56, 145.87, 136.77, 133.98, 133.19, 132.00, 129.52, 128.99, 127.91, 126.79, 125.57, 118.52, 72.71, 54.88, 30.43. ESI–MS calcd for $C_{18}H_{18}N_3O_4$ $[M + H]^+$ 340.1292; found 340.1292

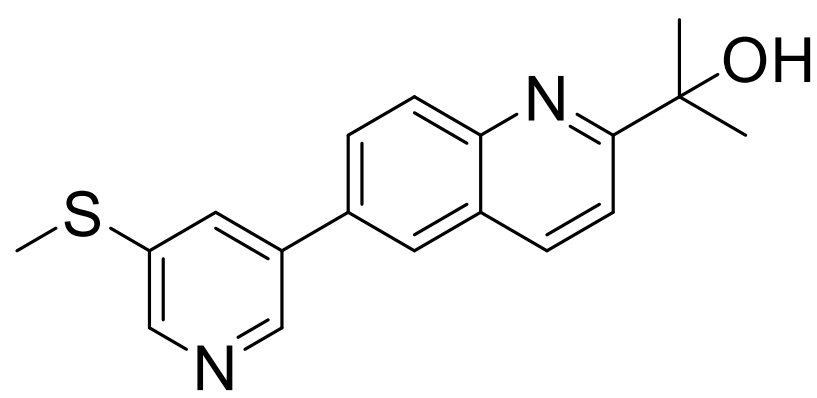

YKJ-119

2-(6-(5-(Methylthio)pyridin-3-yl)quinolin-2-yl)propan-2-ol

Yield: 86.2% ^{1}H NMR (400 MHz, DMSO) δ 8.82 (d, *J* = 2.0 Hz, 1H), 8.51 (d, *J* = 2.2 Hz, 1H), 8.44 – 8.37 (m, 2H), 8.18 – 8.03 (m, 3H), 7.92 (d, *J* = 8.6 Hz, 1H), 5.47 (s, 1H), 2.64 (s, 3H), 1.56 (s, 6H). ^{13}C NMR (101 MHz, DMSO) δ 168.90, 145.96, 145.87, 144.24, 136.84, 135.68, 134.98, 133.86, 131.40, 129.41, 128.33, 126.79, 125.95, 118.41, 72.71, 30.45, 14.43. ESI–MS calcd for $C_{18}H_{19}N_2OS$ $[M + H]^+$ 311.1213; found 311.1214

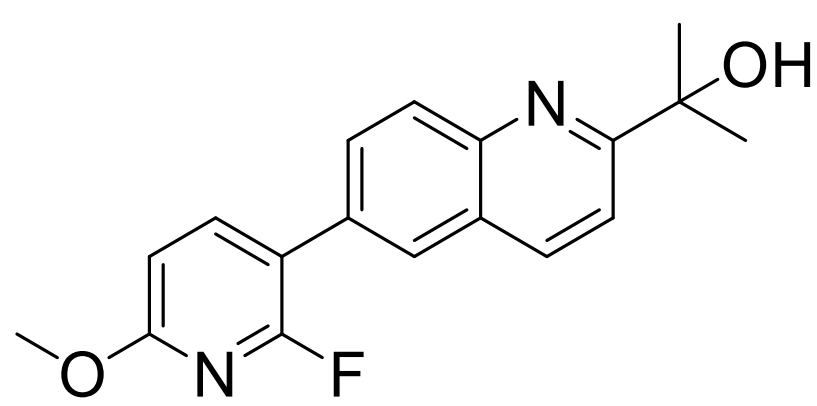

YKJ-120

2-(6-(2-Fluoro-6-methoxypyridin-3-yl)quinolin-2-yl)propan-2-ol

Yield: quantitative ^{1}H NMR (400 MHz, DMSO) δ 8.39 (d, *J* = 8.6 Hz, 1H), 8.19 (dd, *J* = 10.4, 8.2 Hz, 1H), 8.14 (s, 1H), 8.04 (d, *J* = 8.7 Hz, 1H), 7.94 – 7.88 (m, 2H), 6.95 (dd, *J* = 8.3, 1.2 Hz, 1H), 5.45 (s, 1H), 3.91 (s, 3H), 1.55 (s, 6H). ^{13}C NMR (126 MHz, DMSO) δ 168.83, 162.09 (d, *J* = 13.8 Hz), 158.02 (d, *J* = 240.6 Hz), 145.49, 144.08 (d, *J* = 4.3 Hz), 136.72, 130.99 (d, *J* = 5.2 Hz), 129.81 (d, *J* = 3.1 Hz), 128.96, 127.26 (d, *J* = 3.1 Hz), 126.60, 118.36, 113.35 (d, *J* = 26.3 Hz), 108.09 (d, *J* = 5.0 Hz), 72.74, 54.22, 30.48. ESI–MS calcd for $C_{18}H_{18}FN_2O_2$ $[M + H]^+$ 313.1347; found 313.1345

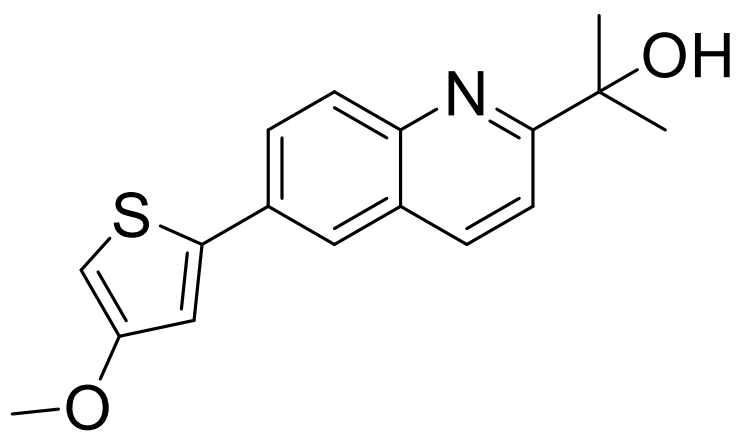

YKJ-121

2-(6-(4-Methoxythiophen-2-yl)quinolin-2-yl)propan-2-ol

Yield: 96.4% ^{1}H NMR (400 MHz, DMSO) δ 8.36 (d, *J* = 8.6 Hz, 1H), 8.18 (d, *J* = 2.1 Hz, 1H), 8.04 – 7.92 (m, 2H), 7.87 (d, *J* = 8.6 Hz, 1H), 7.40 (d, *J* = 1.7 Hz, 1H), 6.67 (d, *J* = 1.7 Hz, 1H), 5.43 (s, 1H), 3.80 (s, 3H), 1.54 (s, 6H). ^{13}C NMR (101 MHz, DMSO) δ 168.39, 158.56, 145.73, 141.03, 136.51, 131.09, 129.36, 126.82, 126.77, 122.66, 118.51, 116.47, 98.00, 72.65, 57.19, 30.41. ESI–MS calcd for $C_{17}H_{18}NO_2S$ [M + H]$^+$ 300.1053; found 300.1057

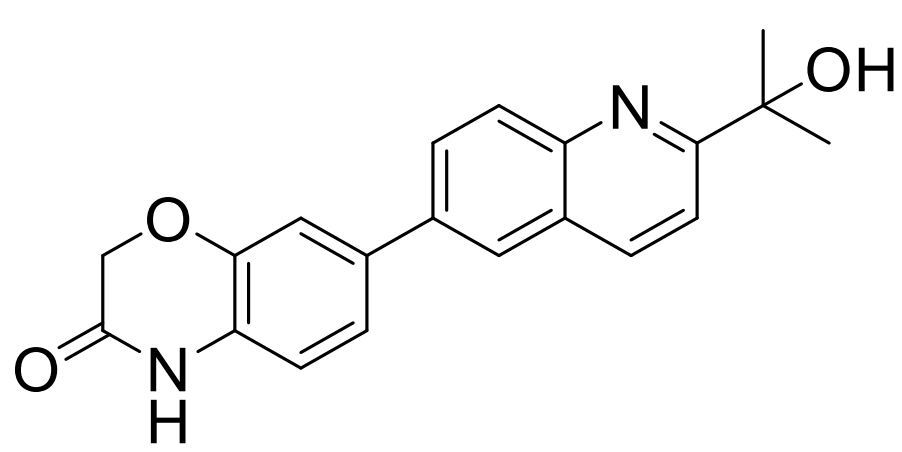

YKJ-122

7-(2-(2-Hydroxypropan-2-yl)quinolin-6-yl)-2*H*-benzo[*b*][1,4]oxazin-3(4*H*)-one

Yield: 54.3% ^{1}H NMR (400 MHz, DMSO) δ 10.84 (s, 1H), 8.36 (d, *J* = 8.7 Hz, 1H), 8.21 (d, *J* = 2.0 Hz, 1H), 8.08 – 7.94 (m, 2H), 7.87 (d, *J* = 8.6 Hz, 1H), 7.52 – 7.37 (m, 2H), 7.02 (d, *J* = 7.9 Hz, 1H), 5.44 (s, 1H), 4.64 (s, 2H), 1.55 (s, 6H). ^{13}C NMR (101 MHz, DMSO) δ 168.19, 164.77, 145.51, 143.77, 136.66, 136.56, 134.38, 129.11, 128.11, 126.95, 126.85, 124.43, 120.98, 118.18, 116.32, 114.48, 72.65, 66.81, 30.46. ESI–MS calcd for $C_{20}H_{19}N_2O_3$ [M + H]$^+$ 335.1390; found 335.1390

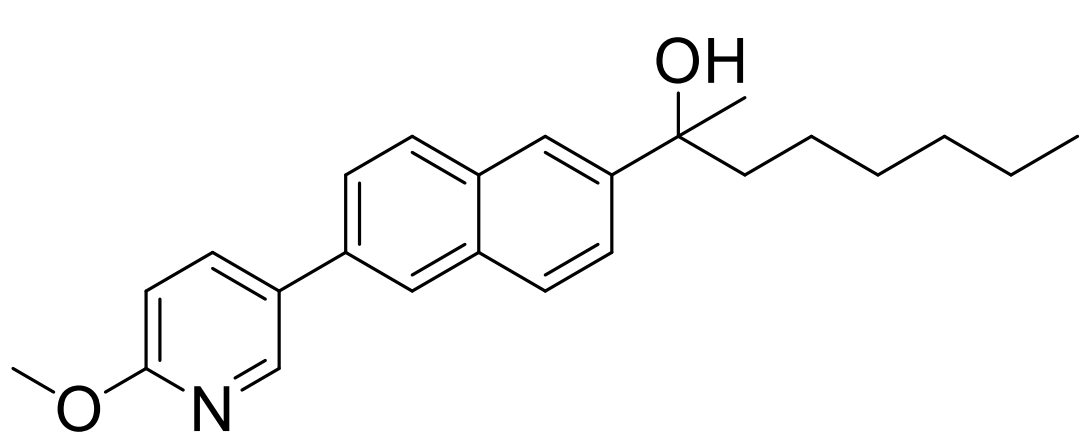

YKJ-123

2-(6-(6-Methoxypyridin-3-yl)naphthalen-2-yl)octan-2-ol

Yield: 41.1% ^{1}H NMR (400 MHz, DMSO) δ 8.62 (d, *J* = 2.4 Hz, 1H), 8.19 – 8.12 (m, 2H), 8.00 – 7.92 (m, 2H), 7.89 (d, *J* = 8.7 Hz, 1H), 7.80 (dd, *J* = 8.5, 1.9 Hz, 1H), 7.60 (dd, *J* = 8.6, 1.8 Hz, 1H), 6.95 (d, *J* = 8.4 Hz, 1H), 4.98 (s, 1H), 3.92 (s, 3H), 1.84 – 1.68 (m, 2H), 1.51 (s, 3H), 1.31 – 1.10 (m, 8H), 0.82 – 0.75 (m, 3H). ^{13}C NMR (101 MHz, DMSO) δ 163.04, 147.11, 144.83, 137.66, 133.68, 131.84, 131.82, 129.26, 128.70, 127.39, 124.80, 124.49, 124.29, 122.71, 110.65, 72.99, 53.28, 43.89, 31.24, 30.16, 29.10, 23.55, 21.99, 13.86. ESI–MS calcd for $C_{24}H_{30}NO_2$ $[M + H]^+$ 364.2271; found 364.2272

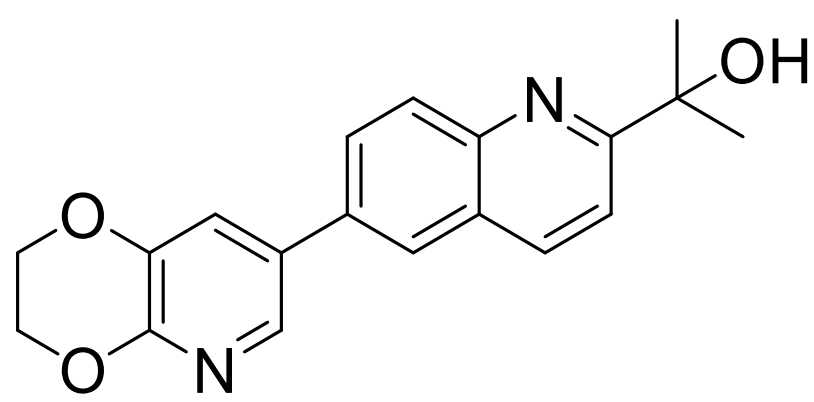

YKJ-124

2-(6-(2,3-Dihydro-[1,4]dioxino[2,3-*b*]pyridin-7-yl)quinolin-2-yl)propan-2-ol

Yield: 85.4% ^{1}H NMR (400 MHz, DMSO) δ 8.36 (d, *J* = 8.7 Hz, 1H), 8.26 (d, *J* = 2.0 Hz, 1H), 8.24 (d, *J* = 2.2 Hz, 1H), 8.09 – 7.98 (m, 2H), 7.89 (d, *J* = 8.6 Hz, 1H), 7.77 (d, *J* = 2.2 Hz, 1H), 5.45 (s, 1H), 4.50 – 4.42 (m, 2H), 4.36 – 4.29 (m, 2H), 1.55 (s, 6H). ^{13}C NMR (101 MHz, DMSO) δ 168.44, 150.62, 145.61, 139.28, 137.22, 136.66, 133.90, 130.36, 129.26, 128.08, 126.84, 124.82, 122.68, 118.28, 72.67, 64.83, 63.81, 30.45. ESI–MS calcd for $C_{19}H_{19}N_2O_3$ $[M + H]^+$ 323.1390; found 323.1387

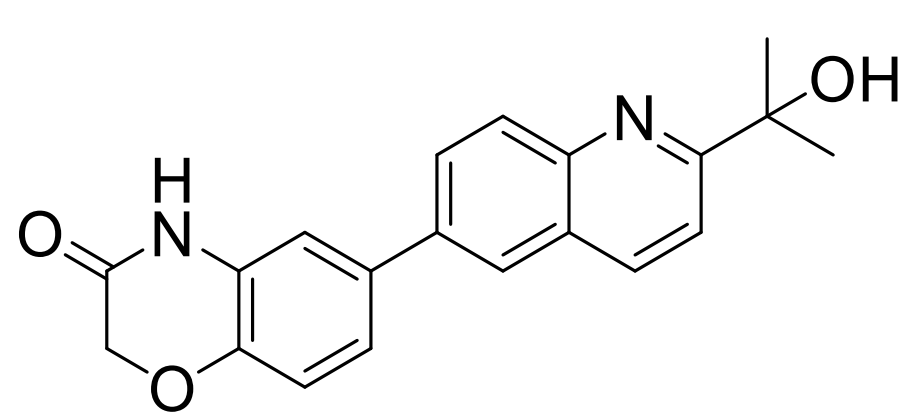

YKJ-125

6-(2-(2-Hydroxypropan-2-yl)quinolin-6-yl)-2*H*-benzo[*b*][1,4]oxazin-3(4*H*)-one

Yield: 59.3% ^{1}H NMR (400 MHz, DMSO) δ 10.82 (s, 1H), 8.39 (d, *J* = 8.6 Hz, 1H), 8.11 (d, *J* = 2.1 Hz, 1H), 8.03 (d, *J* = 8.8 Hz, 1H), 7.93 (dd, *J* = 8.8, 2.1 Hz, 1H), 7.88 (d, *J* = 8.6 Hz, 1H), 7.38 (dd, *J* = 8.4, 2.2 Hz, 1H), 7.29 (d, *J* = 2.2 Hz, 1H), 7.09 (d, *J* = 8.4 Hz, 1H), 5.44 (s, 1H), 4.64 (s,

2H), 1.55 (s, 6H). ^{13}C NMR (101 MHz, DMSO) δ 168.27, 164.77, 145.47, 143.17, 136.89, 136.67, 133.87, 129.30, 128.15, 127.82, 126.84, 124.40, 121.78, 118.26, 116.74, 114.22, 72.66, 66.83, 30.46. ESI–MS calcd for $C_{20}H_{19}N_2O_3$ $[M + H]^+$ 335.1390; found 335.1390

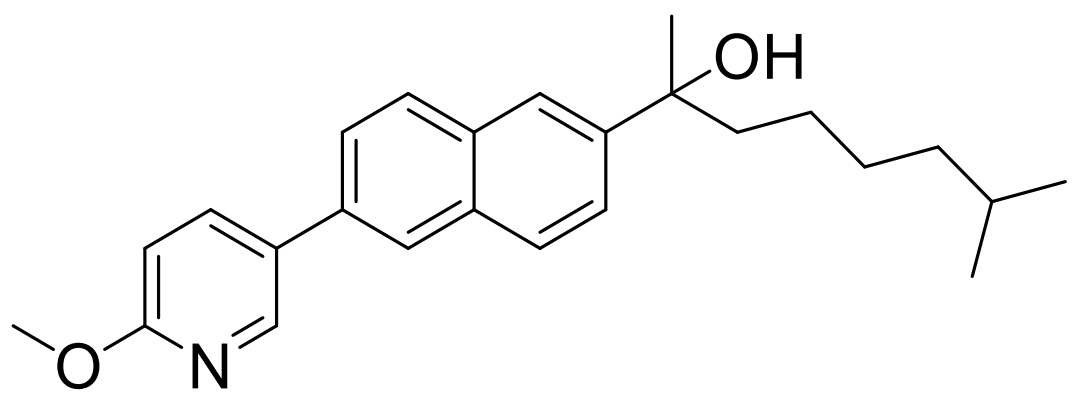

YKJ-126

2-(6-(6-Methoxypyridin-3-yl)naphthalen-2-yl)-7-methyloctan-2-ol

Yield: 25.1% ^{1}H NMR (400 MHz, DMSO) δ 8.62 (d, *J* = 2.6 Hz, 1H), 8.22 – 8.11 (m, 2H), 8.02 – 7.93 (m, 2H), 7.89 (d, *J* = 8.6 Hz, 1H), 7.80 (dd, *J* = 8.5, 1.9 Hz, 1H), 7.60 (dd, *J* = 8.6, 1.8 Hz, 1H), 6.96 (d, *J* = 8.6 Hz, 1H), 4.98 (s, 1H), 3.92 (s, 3H), 1.84 – 1.68 (m, 2H), 1.51 (s, 3H), 1.44 – 1.35 (m, 1H), 1.30 – 1.11 (m, 3H), 1.08 – 0.91 (m, 3H), 0.78 (d, *J* = 6.5 Hz, 6H). ^{13}C NMR (101 MHz, DMSO) δ 163.05, 147.11, 144.84, 137.68, 133.68, 131.84, 129.26, 128.71, 127.40, 124.81, 124.50, 124.30, 122.72, 110.66, 73.00, 53.29, 43.94, 38.45, 30.17, 27.28, 27.19, 23.86, 22.46, 22.43. (one low field carbon not observed). ESI–MS calcd for $C_{25}H_{32}NO_2$ $[M + H]^+$ 378.2428; found 378.2433

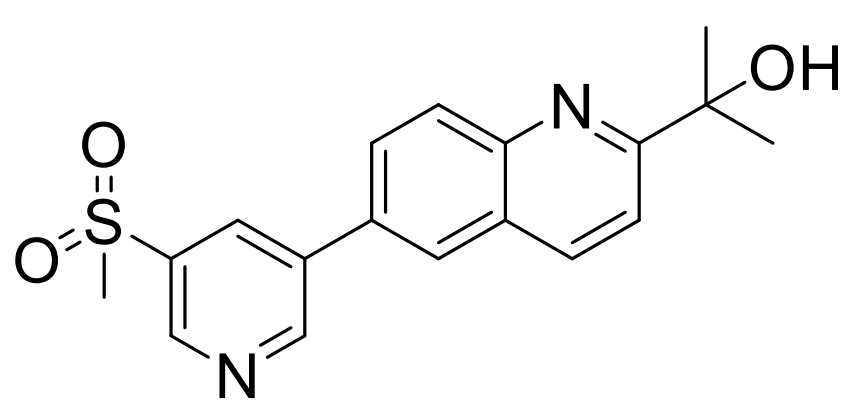

YKJ-127

2-(6-(5-(Methylsulfonyl)pyridin-3-yl)quinolin-2-yl)propan-2-ol

Yield: quantitative ^{1}H NMR (400 MHz, DMSO) δ 9.41 (d, *J* = 2.2 Hz, 1H), 9.10 (d, *J* = 2.2 Hz, 1H), 8.72 (t, *J* = 2.2 Hz, 1H), 8.52 (d, *J* = 2.2 Hz, 1H), 8.45 (d, *J* = 8.6 Hz, 1H), 8.25 (dd, *J* = 8.8, 2.2 Hz, 1H), 8.12 (d, *J* = 8.8 Hz, 1H), 7.95 (d, *J* = 8.7 Hz, 1H), 5.48 (s, 1H), 3.44 (s, 3H), 1.57 (s, 6H). ^{13}C NMR (101 MHz, DMSO) δ 169.35, 152.27, 146.40, 137.36, 136.98, 133.07, 132.59, 131.52, 131.43, 129.69, 128.82, 128.70, 128.25, 118.63, 72.76, 43.62, 30.43. ESI–MS calcd for $C_{18}H_{19}N_2O_3S$ $[M + H]^+$ 343.1111; found 343.1111

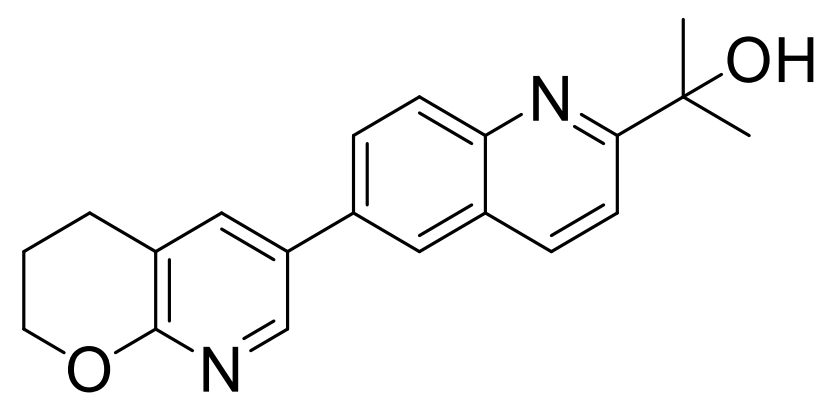

YKJ-128

2-(6-(3,4-Dihydro-2*H*-pyrano[2,3-*b*]pyridin-6-yl)quinolin-2-yl)propan-2-ol

Yield: 88.2% ^{1}H NMR (400 MHz, DMSO) δ 8.47 (d, *J* = 2.5 Hz, 1H), 8.36 (d, *J* = 8.7 Hz, 1H), 8.24 (d, *J* = 2.0 Hz, 1H), 8.07 – 7.98 (m, 3H), 7.88 (d, *J* = 8.6 Hz, 1H), 5.45 (s, 1H), 4.33 (t, *J* = 5.2 Hz, 2H), 2.89 (t, *J* = 6.4 Hz, 2H), 2.03 – 1.91 (m, 2H), 1.55 (s, 6H). ^{13}C NMR (101 MHz, DMSO) δ 168.27, 160.76, 145.48, 143.90, 137.15, 136.58, 134.40, 129.27, 128.53, 127.95, 126.90, 124.30, 118.26, 117.73, 72.66, 66.93, 30.46, 24.27, 21.30. ESI–MS calcd for $C_{20}H_{21}N_2O_2$ [M + H]$^+$ 321.1598; found 321.1599

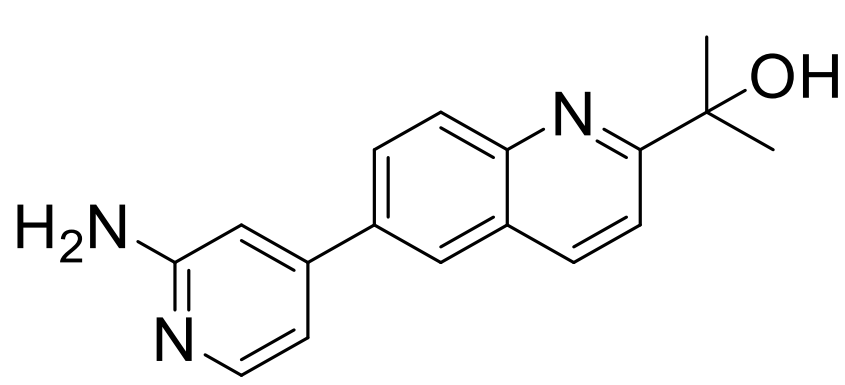

YKJ-129

2-(6-(2-Aminopyridin-4-yl)quinolin-2-yl)propan-2-ol

Yield: 84.4 % ^{1}H NMR (400 MHz, DMSO) δ 8.43 (d, *J* = 8.6 Hz, 1H), 8.25 (d, *J* = 2.1 Hz, 1H), 8.08 – 7.94 (m, 3H), 7.90 (d, *J* = 8.7 Hz, 1H), 6.93 (dd, *J* = 5.4, 1.6 Hz, 1H), 6.85 (s, 1H), 6.03 (s, 2H), 5.46 (s, 1H), 1.55 (s, 6H). ^{13}C NMR (101 MHz, DMSO) δ 168.90, 160.51, 148.63, 147.44, 146.21, 136.96, 135.71, 129.37, 127.78, 126.70, 125.36, 118.37, 110.18, 105.31, 72.71, 30.45. ESI–MS calcd for $C_{17}H_{18}N_3O$ [M + H]$^+$ 280.1444; found 280.1444

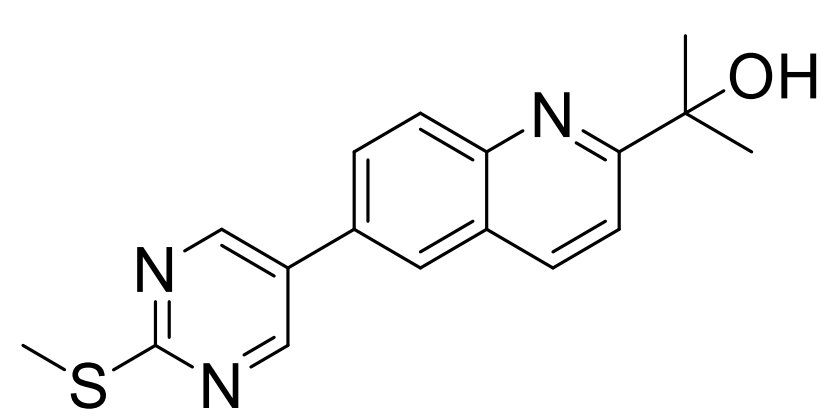

YKJ-131

2-(6-(2-(Methylthio)pyrimidin-5-yl)quinolin-2-yl)propan-2-ol

Yield: 98.5% ^{1}H NMR (400 MHz, DMSO) δ 9.14 (s, 2H), 8.42 – 8.34 (m, 2H), 8.16 – 8.05 (m, 2H), 7.92 (d, *J* = 8.7 Hz, 1H), 5.47 (s, 1H), 2.59 (s, 3H), 1.56 (s, 6H). ^{13}C NMR (101 MHz, DMSO) δ 170.45, 168.98, 155.49, 145.97, 136.73, 131.07, 129.60, 127.94, 127.67, 126.82, 125.29, 118.54, 72.72, 30.44, 13.68. ESI–MS calcd for $C_{17}H_{18}N_3OS$ [M + H]$^+$ 312.1165; found 312.1169

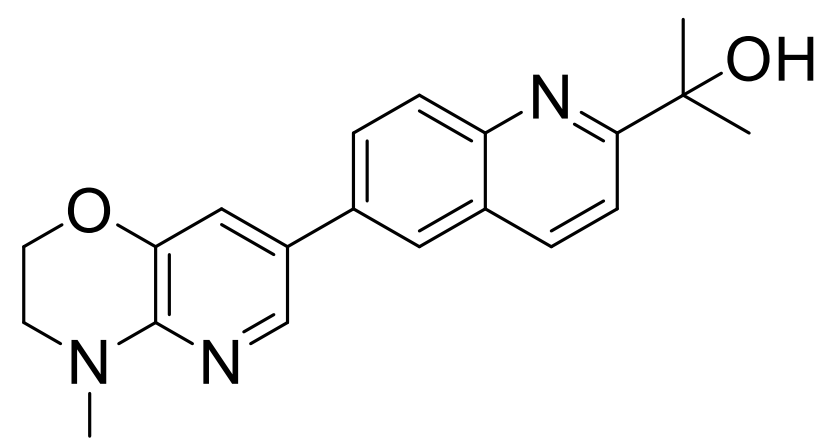

YKJ-132

2-(6-(4-Methyl-3,4-dihydro-2*H*-pyrido[3,2-*b*][1,4]oxazin-7-yl)quinolin-2-yl)propan-2-ol

Yield: 56.3% ^{1}H NMR (400 MHz, DMSO) δ 8.32 (d, *J* = 8.6 Hz, 1H), 8.19 (d, *J* = 2.1 Hz, 1H), 8.15 (d, *J* = 2.0 Hz, 1H), 8.04 – 7.94 (m, 2H), 7.85 (d, *J* = 8.6 Hz, 1H), 7.42 (d, *J* = 2.2 Hz, 1H), 5.43 (s, 1H), 4.32 – 4.21 (m, 2H), 3.54 – 3.44 (m, 2H), 3.09 (s, 3H), 1.54 (s, 6H). ^{13}C NMR (101 MHz, DMSO) δ 167.80, 147.41, 145.25, 139.66, 137.53, 136.43, 134.93, 129.08, 127.71, 126.98, 124.37, 123.34, 118.44, 118.11, 72.62, 63.88, 47.34, 35.41, 30.48. ESI–MS calcd for $C_{20}H_{22}N_3O_2$ [M + H]$^+$ 336.1707; found 336.1708

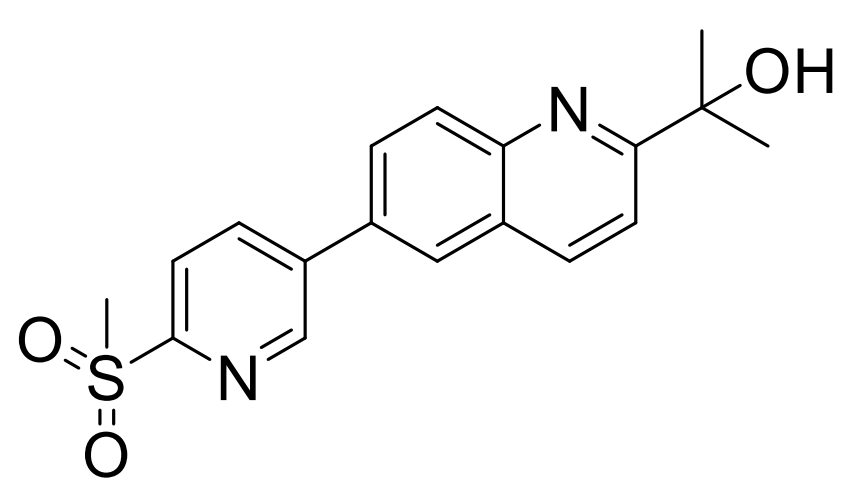

YKJ-133

2-(6-(6-(Methylsulfonyl)pyridin-3-yl)quinolin-2-yl)propan-2-ol

Yield: 84.7% ^{1}H NMR (400 MHz, DMSO) δ 9.27 (d, *J* = 2.3 Hz, 1H), 8.59 (dd, *J* = 8.2, 2.3 Hz, 1H), 8.52 – 8.41 (m, 2H), 8.23 – 8.09 (m, 3H), 7.95 (d, *J* = 8.6 Hz, 1H), 5.48 (s, 1H), 3.35 (s, 3H), 1.56 (s, 6H). ^{13}C NMR (101 MHz, DMSO) δ 169.49, 156.49, 148.44, 146.29, 138.65, 137.03, 136.83, 132.68, 129.72, 128.28, 126.88, 126.77, 121.07, 118.65, 72.76, 40.03, 30.43. ESI–MS calcd for $C_{18}H_{19}N_2O_3S$ [M + H]$^+$ 343.1111; found 343.1107

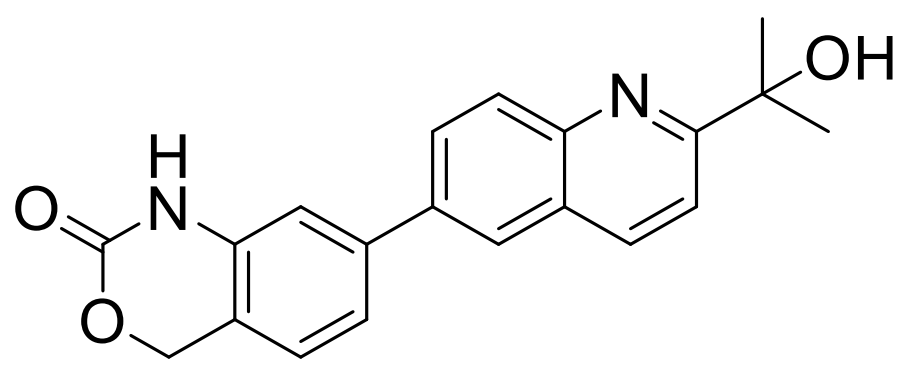

YKJ-134

7-(2-(2-Hydroxypropan-2-yl)quinolin-6-yl)-1,4-dihydro-2*H*-benzo[*d*][1,3]oxazin-2-one

Yield: 82.3% ^{1}H NMR (400 MHz, DMSO) δ 10.28 (s, 1H), 8.42 (d, *J* = 8.7 Hz, 1H), 8.16 (d, *J* = 2.1 Hz, 1H), 8.05 (d, *J* = 8.8 Hz, 1H), 7.95 (dd, *J* = 8.8, 2.1 Hz, 1H), 7.90 (d, *J* = 8.6 Hz, 1H), 7.46 (dd, *J* = 7.8, 1.7 Hz, 1H), 7.35 (d, *J* = 7.8 Hz, 1H), 7.26 (d, *J* = 1.7 Hz, 1H), 5.45 (s, 1H), 5.36 (s, 2H), 1.55 (s, 6H). ^{13}C NMR (101 MHz, DMSO) δ 168.56, 151.73, 145.74, 140.18, 137.19, 136.96, 136.79, 129.38, 128.25, 126.78, 125.19, 125.05, 121.16, 118.33, 118.07, 111.98, 72.68, 67.37, 30.45. ESI–MS calcd for $C_{20}H_{19}N_2O_3$ $[M + H]^+$ 335.1390; found 335.1390

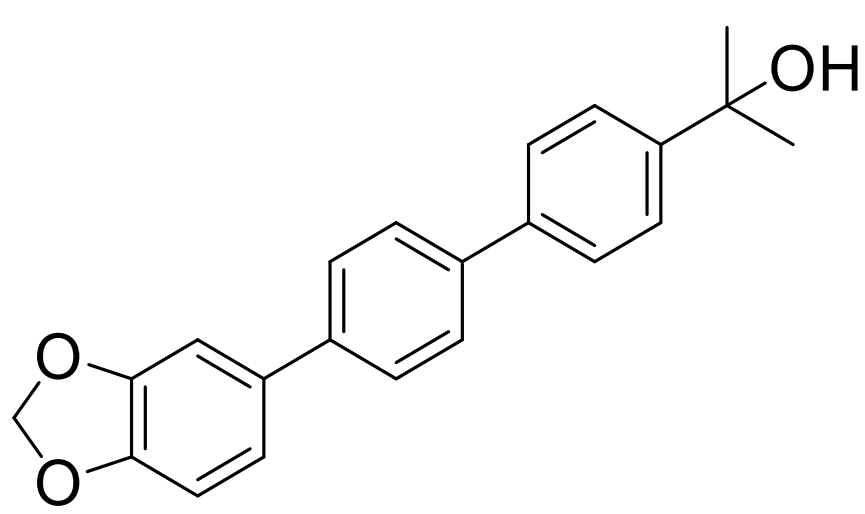

YKJ-135

2-(4'-(Benzo[*d*][1,3]dioxol-5-yl)-[1,1'-biphenyl]-4-yl)propan-2-ol

Yield: 96.9% ^{1}H NMR (400 MHz, DMSO) δ 7.73 – 7.66 (m, 4H), 7.63 (d, *J* = 8.4 Hz, 2H), 7.55 (d, *J* = 8.4 Hz, 2H), 7.30 (d, *J* = 1.8 Hz, 1H), 7.20 (dd, *J* = 8.1, 1.9 Hz, 1H), 7.01 (d, *J* = 8.1 Hz, 1H), 6.07 (s, 2H), 5.04 (s, 1H), 1.46 (s, 6H). ^{13}C NMR (101 MHz, DMSO) δ 149.81, 148.00, 146.88, 138.63, 137.22, 133.94, 126.87 (four peaks overlap – two sets of 2 identical carbons), 125.87, 125.18, 120.11, 108.69, 106.96, 101.13, 70.53, 31.88 (one low field carbon not observed). ESI–MS calcd for $C_{22}H_{19}O_2$ $[M - OH]^+$ 315.1380; found 315.1380

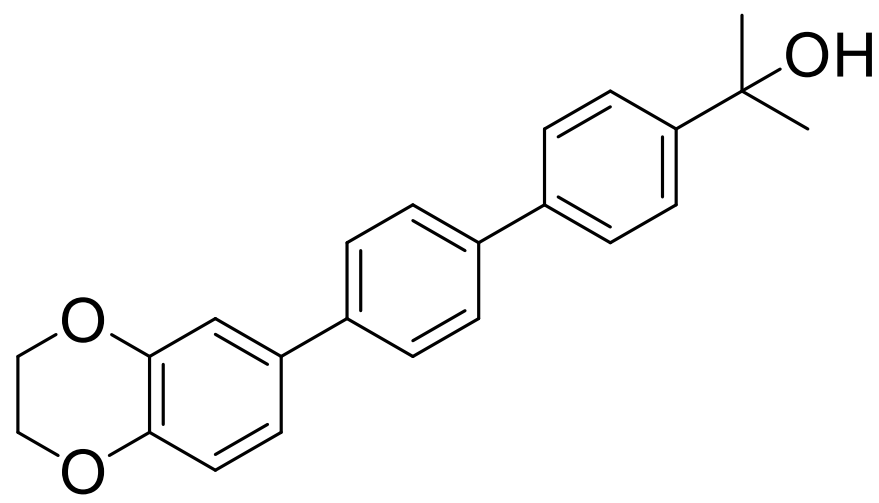

YKJ-136

2-(4'-(2,3-Dihydrobenzo[*b*][1,4]dioxin-6-yl)-[1,1'-biphenyl]-4-yl)propan-2-ol

Yield: 93.4% ^{1}H NMR (400 MHz, DMSO) δ 7.72 – 7.65 (m, 4H), 7.65 – 7.60 (m, 2H), 7.57 – 7.52 (m, 2H), 7.21 – 7.16 (m, 2H), 6.95 (dd, *J* = 8.1, 0.7 Hz, 1H), 5.04 (s, 1H), 4.28 (s, 4H), 1.46 (s, 6H). ^{13}C NMR (101 MHz, DMSO) δ 149.78, 143.69, 143.17, 138.56, 137.26, 132.95, 126.90, 126.66, 125.87, 125.18, 119.38, 117.51, 114.94, 70.53, 64.11, 31.89 (one low field carbon not observed). ESI–MS calcd for $C_{23}H_{21}O_2$ $[M + H]^+$ 329.1536; found 329.1539

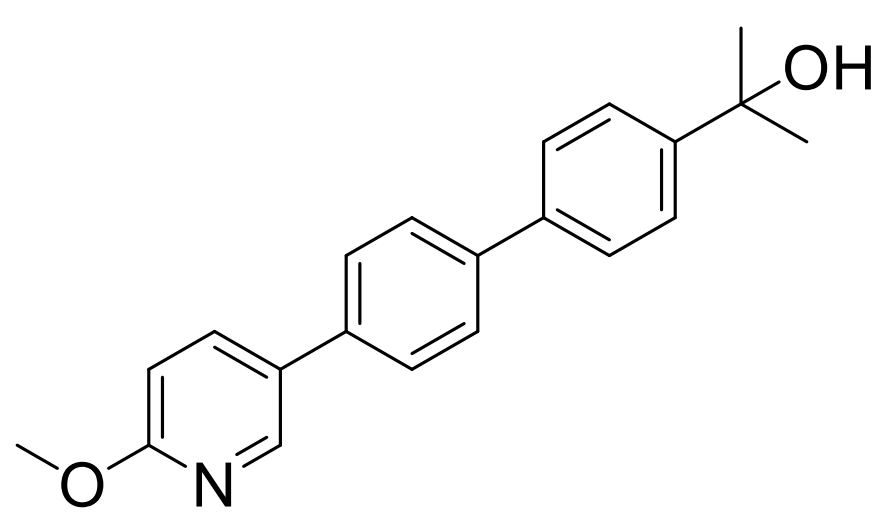

YKJ-137

2-(4'-(6-Methoxypyridin-3-yl)-[1,1'-biphenyl]-4-yl)propan-2-ol

Yield: 91.7% ^{1}H NMR (400 MHz, $CDCl_3$) δ 8.44 (d, *J* = 2.5 Hz, 1H), 7.84 (dd, *J* = 8.5, 2.5 Hz, 1H), 7.68 (d, *J* = 8.2 Hz, 2H), 7.64 – 7.57 (m, 6H), 6.84 (d, *J* = 8.6 Hz, 1H), 4.00 (s, 3H), 1.64 (s, 6H). ^{13}C NMR (101 MHz, $CDCl_3$) δ 163.69, 148.38, 144.92, 138.97, 137.37, 135.07, 129.65, 127.63, 127.02, 126.86, 125.00, 110.91, 72.50, 53.61, 31.80 (one low field carbon not observed). ESI–MS calcd for $C_{21}H_{22}NO_2$ $[M + H]^+$ 320.1645; found 320.1647

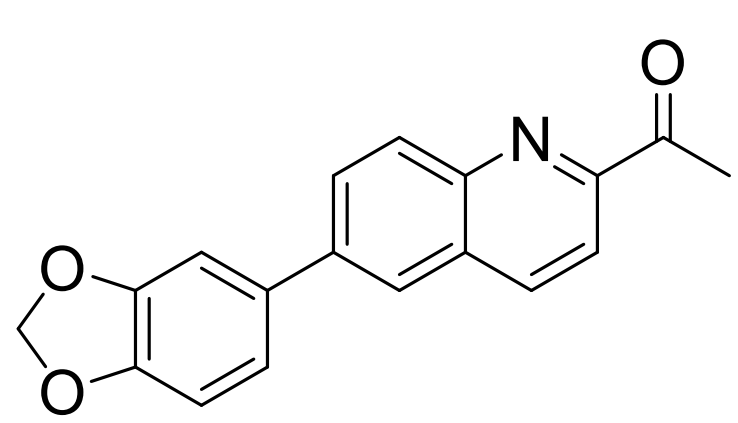

YKJ-138

1-(6-(Benzo[*d*][1,3]dioxol-5-yl)quinolin-2-yl)ethan-1-one

Yield: quantitative ^{1}H NMR (400 MHz, DMSO) δ 8.53 (d, *J* = 8.5 Hz, 1H), 8.32 (d, *J* = 1.9 Hz, 1H), 8.22 – 8.14 (m, 2H), 8.06 (d, *J* = 8.5 Hz, 1H), 7.48 (d, *J* = 1.9 Hz, 1H), 7.39 (dd, *J* = 8.1, 1.9 Hz, 1H), 7.08 (d, *J* = 8.1 Hz, 1H), 6.11 (s, 2H), 2.78 (s, 3H). ^{13}C NMR (101 MHz, DMSO) δ 199.60, 152.61, 148.27, 147.64, 145.68, 139.81, 137.62, 133.00, 130.32, 129.61, 129.56, 124.63, 121.17, 118.02, 108.90, 107.45, 101.42, 25.39. ESI–MS calcd for $C_{18}H_{14}NO_3$ $[M + H]^+$ 292.0968; found 292.0968

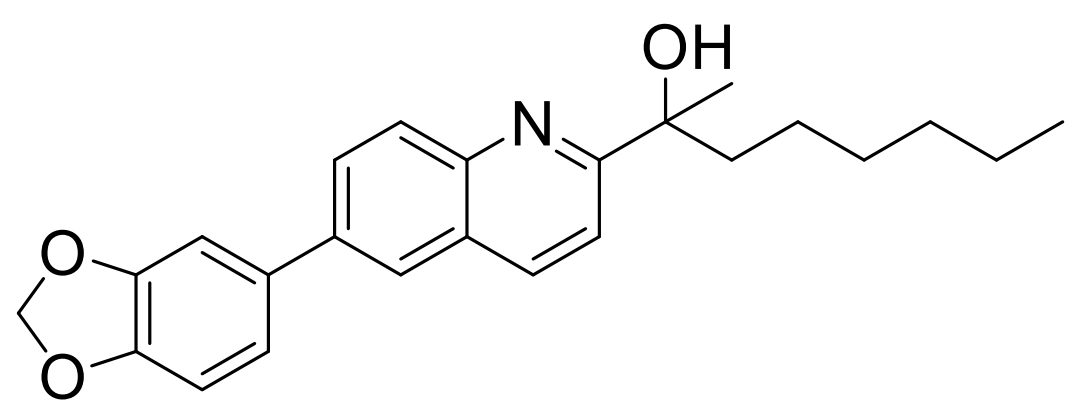

YKJ-139

2-(6-(Benzo[*d*][1,3]dioxol-5-yl)quinolin-2-yl)octan-2-ol

Yield: 33.6% ^{1}H NMR (400 MHz, $CDCl_3$) δ 8.19 (dd, *J* = 8.7, 0.8 Hz, 1H), 8.14 – 8.06 (m, 1H), 7.95 – 7.88 (m, 2H), 7.42 (d, *J* = 8.6 Hz, 1H), 7.23 – 7.15 (m, 2H), 6.94 (d, *J* = 8.6 Hz, 1H), 6.04 (s, 2H), 5.87 (s, 1H), 1.98 – 1.81 (m, 2H), 1.58 (s, 3H), 1.28 – 1.13 (m, 8H), 0.84 – 0.78 (m, 3H). ^{13}C NMR (101 MHz, $CDCl_3$) δ 165.03, 148.59, 147.72, 145.17, 139.17, 137.49, 134.89, 129.57, 129.41, 127.42, 124.87, 121.30, 117.95, 108.99, 108.07, 101.53, 74.14, 43.18, 31.95, 29.83, 29.66, 23.88, 22.78, 14.24. ESI–MS calcd for $C_{24}H_{28}NO_3$ $[M + H]^+$ 378.2064; found 378.2064

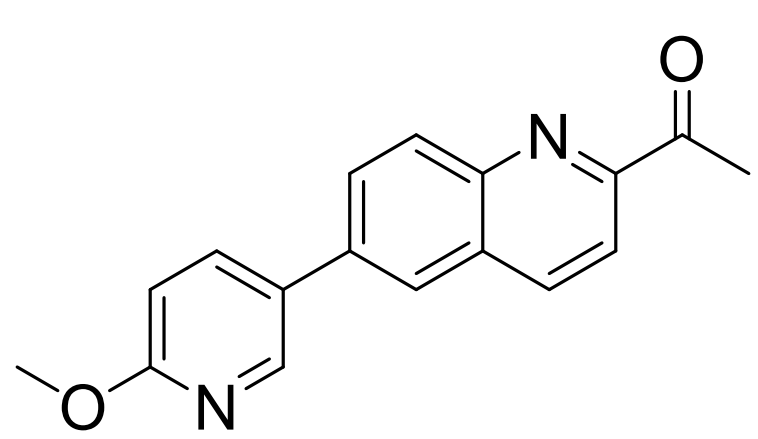

YKJ-140

1-(6-(6-Methoxypyridin-3-yl)quinolin-2-yl)ethan-1-one

Yield: quantitative ^{1}H NMR (400 MHz, DMSO) δ 8.70 (dd, *J* = 2.7, 0.8 Hz, 1H), 8.54 (d, *J* = 8.6 Hz, 1H), 8.40 – 8.35 (m, 1H), 8.25 – 8.20 (m, 3H), 8.07 (d, *J* = 8.6 Hz, 1H), 6.98 (dd, *J* = 8.6, 0.8 Hz, 1H), 3.93 (s, 3H), 2.78 (s, 3H). ^{13}C NMR (101 MHz, DMSO) δ 199.53, 163.56, 152.74, 145.74,

145.43, 137.89, 137.61, 137.03, 130.53, 129.57, 129.14, 128.01, 124.66, 118.08, 110.85, 53.41, 25.36. ESI–MS calcd for $C_{17}H_{15}N_2O_2$ $[M + H]^+$ 279.1128; found 279.1128

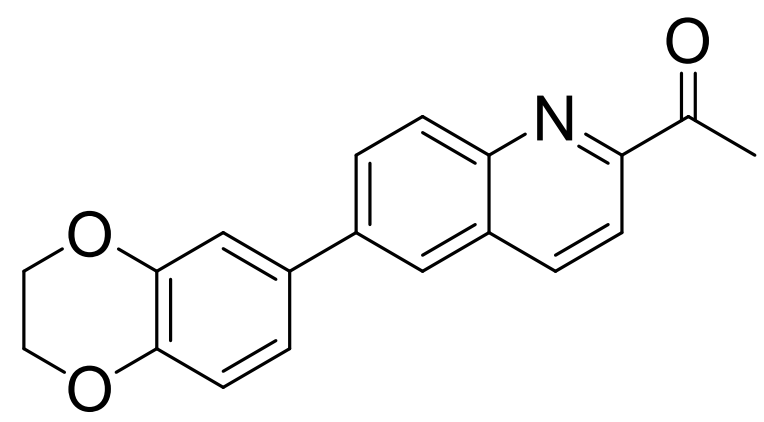

YKJ-141

1-(6-(2,3-Dihydrobenzo[*b*][1,4]dioxin-6-yl)quinolin-2-yl)ethan-1-one

Yield: quantitative ^{1}H NMR (400 MHz, DMSO) δ 8.54 (d, *J* = 8.6 Hz, 1H), 8.32 (d, *J* = 1.9 Hz, 1H), 8.22 – 8.14 (m, 2H), 8.06 (d, *J* = 8.5 Hz, 1H), 7.42 – 7.34 (m, 2H), 7.02 (d, *J* = 8.3 Hz, 1H), 4.31 (s, 4H), 2.78 (s, 3H). ^{13}C NMR (101 MHz, DMSO) δ 199.56, 152.55, 145.64, 143.91, 143.89, 139.50, 137.58, 131.97, 130.30, 129.61, 129.35, 124.39, 120.12, 117.96, 117.74, 115.66, 64.25, 64.13, 25.36. ESI–MS calcd for $C_{19}H_{16}NO_3$ $[M + H]^+$ 306.1125; found 306.1125

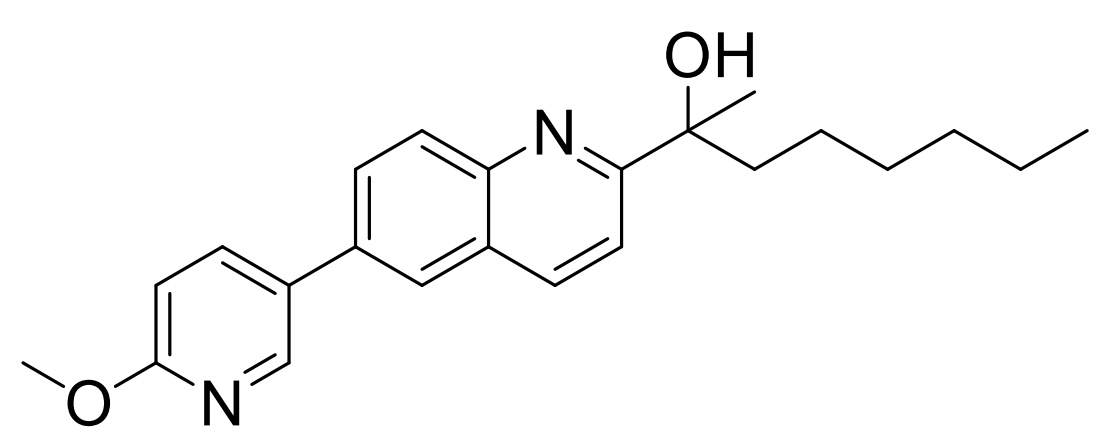

YKJ-142

2-(6-(6-Methoxypyridin-3-yl)quinolin-2-yl)octan-2-ol

Yield: 44.6% ^{1}H NMR (400 MHz, $CDCl_3$) δ 8.51 (d, *J* = 2.6 Hz, 1H), 8.21 (d, *J* = 8.6 Hz, 1H), 8.15 (d, *J* = 8.6 Hz, 1H), 7.97 – 7.86 (m, 3H), 7.44 (d, *J* = 8.6 Hz, 1H), 6.88 (d, *J* = 8.6 Hz, 1H), 5.84 (s, 1H), 4.01 (s, 3H), 1.98 – 1.81 (m, 2H), 1.59 (s, 3H), 1.27 – 1.18 (m, 8H), 0.81 (t, *J* = 6.7 Hz, 3H). ^{13}C NMR (101 MHz, $CDCl_3$) δ 165.33, 164.12, 145.59, 145.28, 137.82, 137.48, 136.21, 129.78, 129.51, 129.11, 127.48, 124.83, 118.12, 111.29, 74.18, 53.86, 43.17, 31.94, 29.81, 29.64, 23.88, 22.77, 14.24. ESI–MS calcd for $C_{23}H_{29}N_2O_2$ $[M + H]^+$ 365.2224; found 365.2226

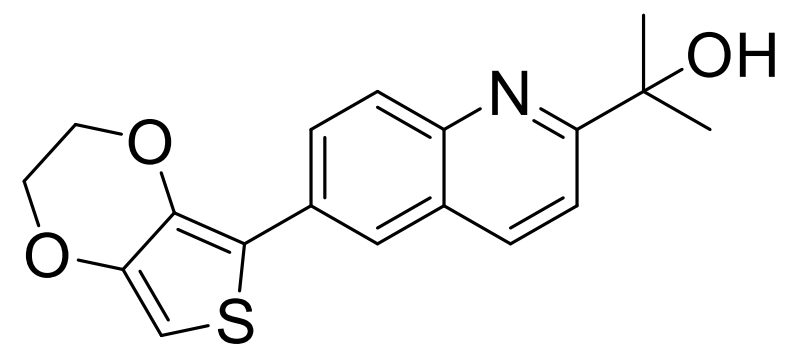

YKJ-143

2-(6-(2,3-Dihydrothieno[3,4-*b*][1,4]dioxin-5-yl)quinolin-2-yl)propan-2-ol

Yield: 98.1% ^{1}H NMR (400 MHz, DMSO) δ 8.34 (d, *J* = 8.7 Hz, 1H), 8.17 (d, *J* = 2.1 Hz, 1H), 8.04 (dd, *J* = 8.8, 2.1 Hz, 1H), 7.96 (d, *J* = 8.8 Hz, 1H), 7.84 (d, *J* = 8.6 Hz, 1H), 6.71 (s, 1H), 5.42 (s, 1H), 4.44 – 4.37 (m, 2H), 4.33 – 4.24 (m, 2H), 1.54 (s, 6H). ^{13}C NMR (101 MHz, DMSO) δ 167.99, 144.93, 142.34, 139.12, 136.37, 130.41, 129.11, 127.24, 126.82, 122.82, 118.38, 115.22, 98.83, 72.62, 64.88, 64.11, 30.43. ESI–MS calcd for $C_{18}H_{18}NO_3S$ $[M + H]^+$ 328.1002; found 328.1001

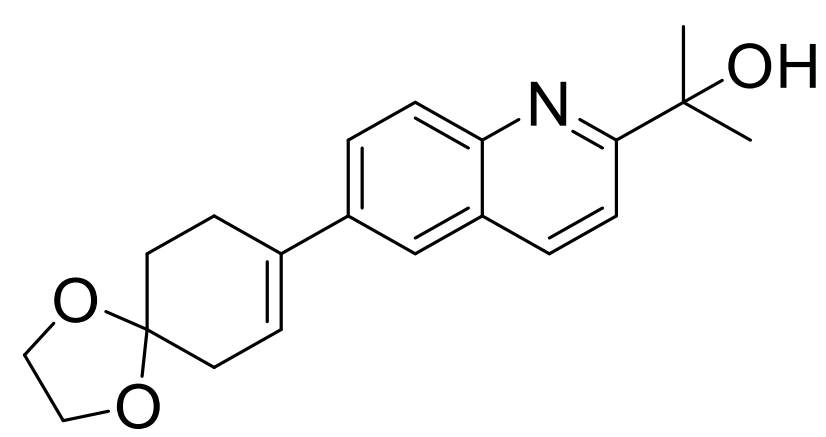

YKJ-144

2-(6-(1,4-Dioxaspiro[4.5]dec-7-en-8-yl)quinolin-2-yl)propan-2-ol

Yield: 60.9% ^{1}H NMR (400 MHz, DMSO) δ 8.30 (d, *J* = 8.6 Hz, 1H), 7.93 – 7.86 (m, 3H), 7.83 (d, *J* = 8.6 Hz, 1H), 6.25 (t, *J* = 4.1 Hz, 1H), 5.41 (s, 1H), 3.94 (s, 4H), 2.73 – 2.64 (m, 2H), 2.46 – 2.40 (m, 2H), 1.87 (t, *J* = 6.5 Hz, 2H), 1.53 (s, 6H). ^{13}C NMR (101 MHz, DMSO) δ 167.80, 145.46, 137.88, 136.59, 134.45, 128.40, 126.86, 126.54, 123.14, 122.78, 117.95, 106.83, 72.59, 63.76, 35.83, 30.82, 30.47, 26.12. ESI–MS calcd for $C_{20}H_{24}NO_3$ $[M + H]^+$ 326.1751; found 326.1748

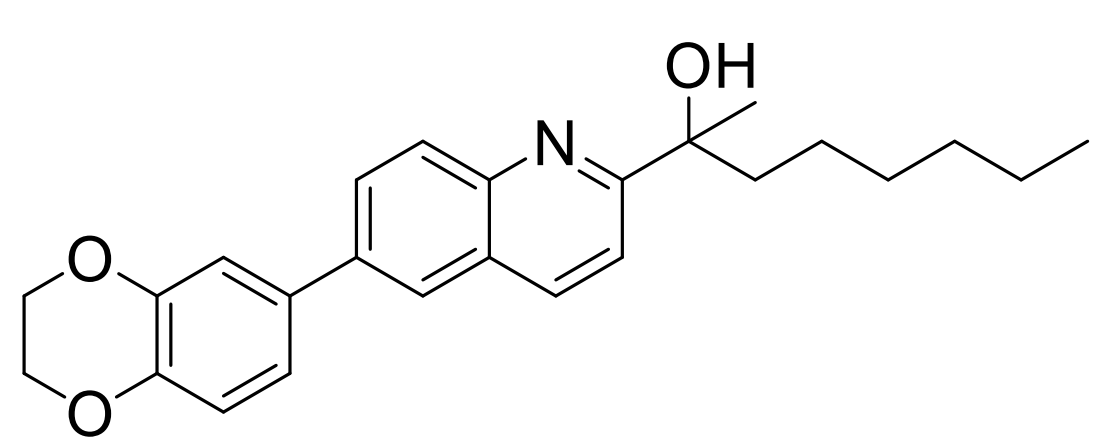

YKJ-145

2-(6-(2,3-Dihydrobenzo[*b*][1,4]dioxin-6-yl)quinolin-2-yl)octan-2-ol

Yield: 28.3% ^{1}H NMR (400 MHz, $CDCl_3$) δ 8.18 (d, *J* = 8.6 Hz, 1H), 8.12 – 8.08 (m, 1H), 7.97 – 7.91 (m, 2H), 7.41 (d, *J* = 8.6 Hz, 1H), 7.25 – 7.18 (m, 2H), 6.99 (d, *J* = 8.3 Hz, 1H), 5.88 (s, 1H), 4.32 (s, 4H), 1.98 – 1.81 (m, 2H), 1.58 (s, 3H), 1.27 – 1.14 (m, 8H), 0.81 (t, *J* = 6.7 Hz, 3H). ^{13}C NMR (101 MHz, $CDCl_3$) δ 164.93, 145.14, 144.11, 143.80, 138.83, 137.51, 134.05, 129.44, 129.34, 127.42, 124.70, 120.65, 118.00, 117.87, 116.37, 74.11, 64.72, 64.67, 43.17, 31.93, 29.82, 29.66, 23.87, 22.77, 14.23. ESI–MS calcd for $C_{25}H_{30}NO_3$ $[M + H]^+$ 392.2220; found 392.2221

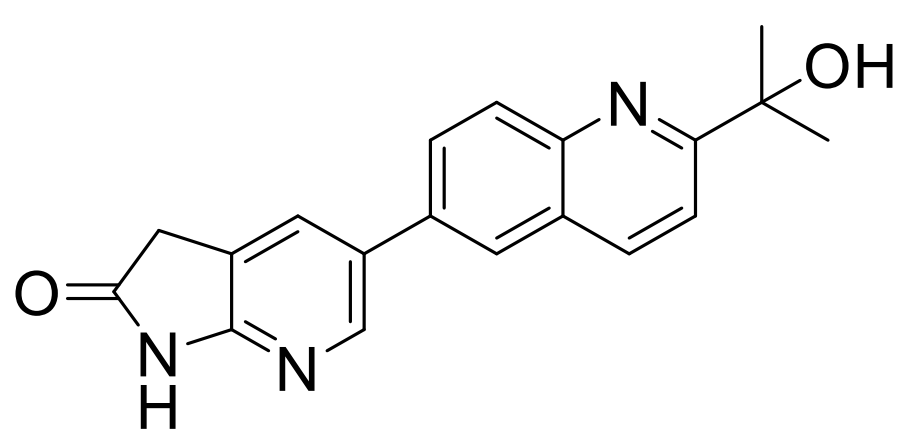

YKJ-146

5-(2-(2-Hydroxypropan-2-yl)quinolin-6-yl)-1,3-dihydro-2*H*-pyrrolo[2,3-*b*]pyridin-2-one

Yield: 69.4% ^{1}H NMR (400 MHz, DMSO) δ 11.13 (s, 1H), 8.53 (d, *J* = 2.2 Hz, 1H), 8.37 (d, *J* = 8.7 Hz, 1H), 8.24 (d, *J* = 1.9 Hz, 1H), 8.08 – 7.99 (m, 3H), 7.89 (d, *J* = 8.7 Hz, 1H), 5.45 (s, 1H), 3.66 (s, 2H), 1.55 (s, 6H). ^{13}C NMR (101 MHz, DMSO) δ 175.87, 168.32, 157.97, 145.51, 144.59, 136.62, 134.95, 130.44, 129.31, 129.08, 128.04, 126.91, 124.50, 120.90, 118.28, 72.67, 35.36, 30.46. ESI–MS calcd for $C_{19}H_{18}N_3O_2$ $[M + H]^+$ 320.1394; found 320.1394

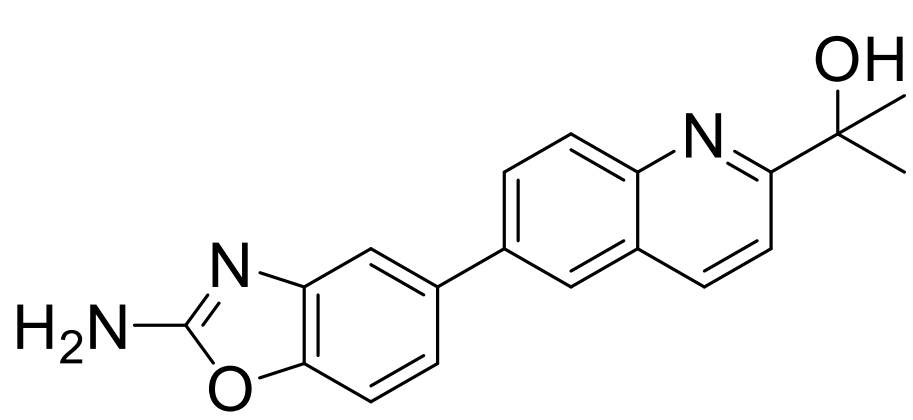

YKJ-147

2-(6-(2-Aminobenzo[*d*]oxazol-5-yl)quinolin-2-yl)propan-2-ol

Yield: 93.5% ^{1}H NMR (400 MHz, DMSO) δ 8.39 (d, *J* = 8.6 Hz, 1H), 8.22 (s, 1H), 8.09 – 7.97 (m, 2H), 7.87 (d, *J* = 8.7 Hz, 1H), 7.62 (s, 1H), 7.50 (s, 2H), 7.43 (q, *J* = 8.2 Hz, 2H), 5.45 (s, 1H), 1.55 (s, 6H). ^{13}C NMR (101 MHz, DMSO) δ 168.04, 163.33, 147.88, 145.39, 144.59, 138.10,

136.69, 135.45, 129.03, 128.81, 126.88, 124.89, 119.26, 118.10, 113.78, 108.69, 72.65, 30.48. ESI–MS calcd for $C_{19}H_{18}N_3O_2$ $[M + H]^+$ 320.1394; found 320.1395

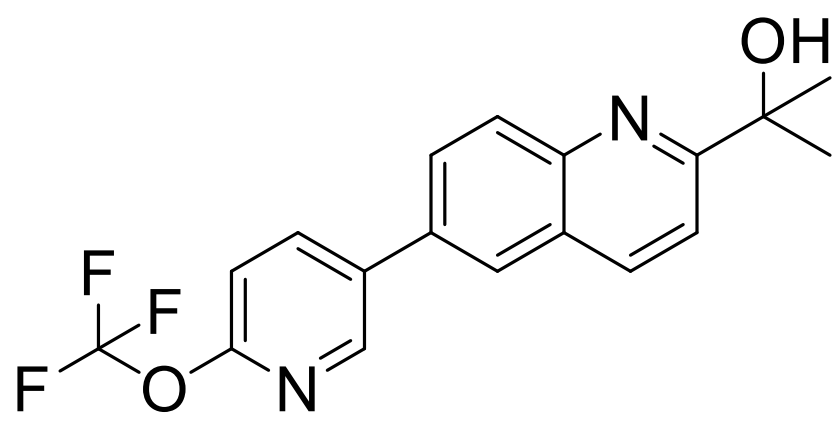

YKJ-148

2-(6-(6-(Trifluoromethoxy)pyridin-3-yl)quinolin-2-yl)propan-2-ol

Yield: 95.0% ^{1}H NMR (400 MHz, DMSO) δ 8.85 (d, *J* = 2.6 Hz, 1H), 8.47 (dd, *J* = 8.6, 2.6 Hz, 1H), 8.40 (d, *J* = 8.6 Hz, 1H), 8.36 (s, 1H), 8.14 – 8.04 (m, 2H), 7.92 (d, *J* = 8.6 Hz, 1H), 7.44 (d, *J* = 8.5 Hz, 1H), 5.47 (s, 1H), 1.56 (s, 6H). ^{13}C NMR (126 MHz, DMSO) δ 169.05, 155.40, 146.03, 145.97, 139.84, 136.88, 134.29, 133.01, 129.55, 128.23, 126.80, 125.94, 119.83 (q, *J* = 260.8 Hz), 118.53, 113.36, 72.76, 30.47. ESI–MS calcd for $C_{18}H_{16}F_3N_2O_2$ $[M + H]^+$ 349.1158; found 349.1159

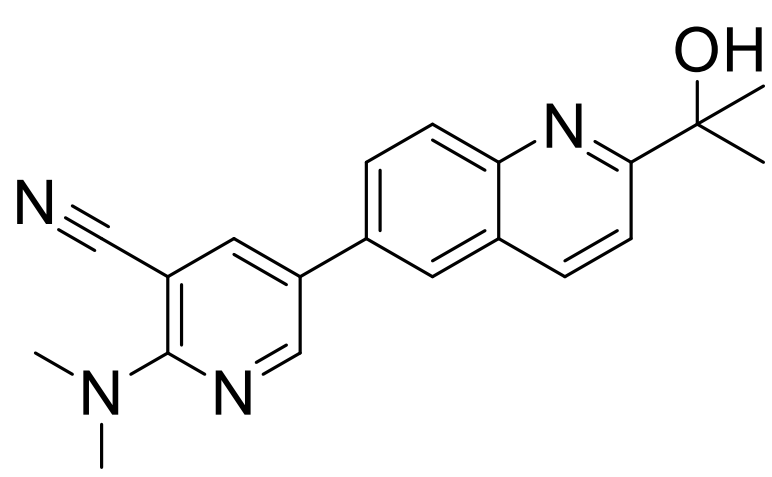

YKJ-149

2-(Dimethylamino)-5-(2-(2-hydroxypropan-2-yl)quinolin-6-yl)nicotinonitrile

Yield: quantitative ^{1}H NMR (400 MHz, DMSO) δ 8.86 (d, *J* = 2.5 Hz, 1H), 8.47 (d, *J* = 2.6 Hz, 1H), 8.33 (d, *J* = 8.6 Hz, 1H), 8.28 (d, *J* = 2.1 Hz, 1H), 8.09 (dd, *J* = 8.8, 2.1 Hz, 1H), 8.00 (d, *J* = 8.8 Hz, 1H), 7.88 (d, *J* = 8.6 Hz, 1H), 5.45 (s, 1H), 3.28 (s, 6H), 1.55 (s, 6H). ^{13}C NMR (101 MHz, DMSO) δ 168.32, 157.96, 150.21, 145.50, 142.38, 136.54, 132.86, 129.28, 127.45, 126.88, 123.97, 123.49, 118.82, 118.32, 90.28, 72.66, 40.01, 30.46. ESI–MS calcd for $C_{20}H_{21}N_4O$ $[M + H]^+$ 333.1710; found 333.1710

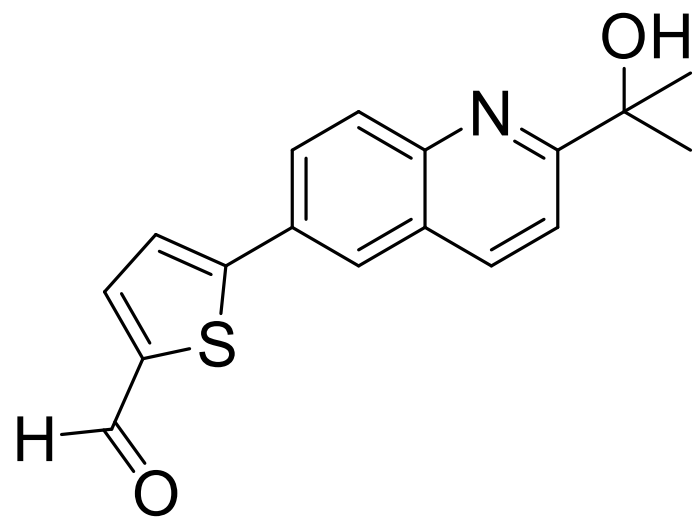

YKJ-150

5-(2-(2-Hydroxypropan-2-yl)quinolin-6-yl)thiophene-2-carboxaldehyde

Yield: 77.1% ^{1}H NMR (400 MHz, DMSO) δ 9.95 (s, 1H), 8.46 – 8.39 (m, 2H), 8.16 (dd, *J* = 8.7, 2.2 Hz, 1H), 8.10 (d, *J* = 4.0 Hz, 1H), 8.03 (d, *J* = 8.7 Hz, 1H), 7.95 – 7.89 (m, 2H), 5.47 (s, 1H), 1.55 (s, 6H). ^{13}C NMR (101 MHz, DMSO) δ 184.13, 169.39, 151.89, 146.43, 142.40, 139.31, 136.90, 129.87, 129.74, 127.32, 126.79, 126.02, 125.11, 118.82, 72.74, 30.39. ESI–MS calcd for $C_{17}H_{16}NO_2S$ $[M + H]^+$ 298.0896; found 298.0898

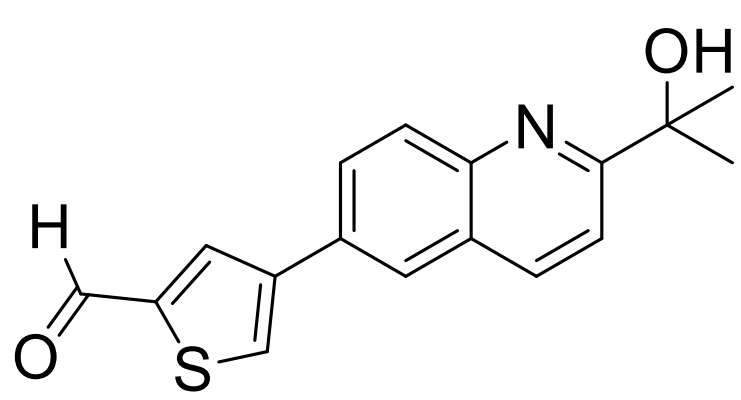

YKJ-151

4-(2-(2-Hydroxypropan-2-yl)quinolin-6-yl)thiophene-2-carboxaldehyde

Yield: 90.9% ^{1}H NMR (400 MHz, DMSO) δ 9.98 (d, *J* = 1.2 Hz, 1H), 8.63 – 8.56 (m, 2H), 8.35 – 8.28 (m, 2H), 8.12 (dd, *J* = 8.8, 2.1 Hz, 1H), 7.99 (d, *J* = 8.8 Hz, 1H), 7.86 (d, *J* = 8.6 Hz, 1H), 5.41 (s, 1H), 1.51 (s, 6H). ^{13}C NMR (101 MHz, DMSO) δ 184.31, 168.58, 145.76, 144.18, 141.97, 136.64, 136.25, 131.24, 131.15, 129.41, 127.80, 126.85, 124.47, 118.48, 72.69, 30.45. ESI–MS calcd for $C_{17}H_{16}NO_2S$ $[M + H]^+$ 298.0896; found 298.0897

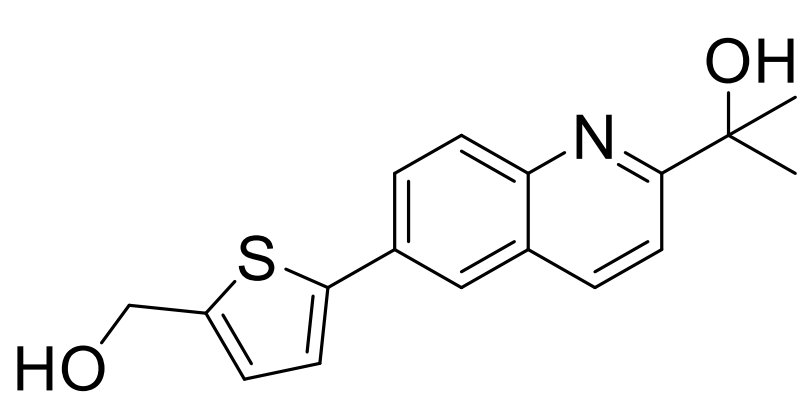

YKJ-152

2-(6-(5-(Hydroxymethyl)thiophen-2-yl)quinolin-2-yl)propan-2-ol

Yield: quantitative ^{1}H NMR (400 MHz, DMSO) δ 8.36 (d, *J* = 8.7 Hz, 1H), 8.15 (d, *J* = 2.1 Hz, 1H), 8.03 (dd, *J* = 8.8, 2.1 Hz, 1H), 7.97 (d, *J* = 8.8 Hz, 1H), 7.86 (d, *J* = 8.7 Hz, 1H), 7.52 (d, *J* = 3.6 Hz, 1H), 7.01 (d, *J* = 3.6 Hz, 1H), 5.56 (s, 1H), 5.43 (s, 1H), 4.67 (s, 2H), 1.54 (s, 6H). ^{13}C NMR (101 MHz, DMSO) δ 168.16, 146.91, 145.50, 141.38, 136.47, 131.48, 129.35, 127.07, 126.95, 125.40, 124.01, 122.71, 118.46, 72.64, 58.44, 30.43. ESI–MS calcd for $C_{17}H_{18}NO_2S$ [M + H]$^+$ 300.1053; 300.1056

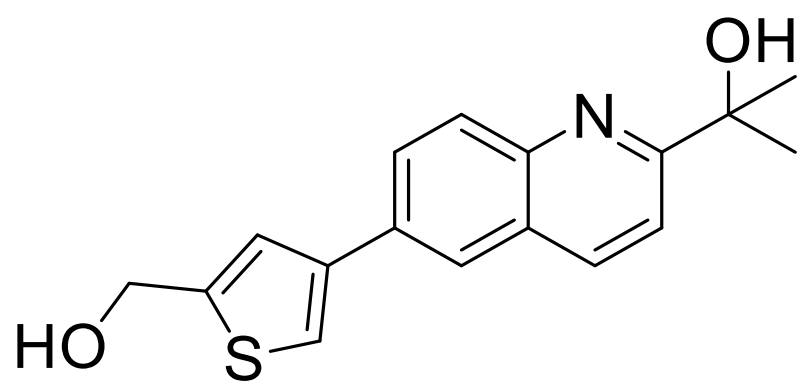

YKJ-153

2-(6-(5-(Hydroxymethyl)thiophen-3-yl)quinolin-2-yl)propan-2-ol

Yield: 89.9% ^{1}H NMR (400 MHz, DMSO) δ 8.33 (d, *J* = 8.7 Hz, 1H), 8.23 (d, *J* = 2.1 Hz, 1H), 8.09 (dd, *J* = 8.8, 2.1 Hz, 1H), 7.99 – 7.90 (m, 2H), 7.86 (d, *J* = 8.6 Hz, 1H), 7.56 (s, 1H), 5.55 (t, *J* = 5.7 Hz, 1H), 5.43 (s, 1H), 4.70 (d, *J* = 5.4 Hz, 2H), 1.54 (s, 6H). ^{13}C NMR (101 MHz, DMSO) δ 167.99, 147.71, 145.43, 139.91, 136.50, 132.67, 129.10, 127.92, 126.93, 123.65, 122.92, 120.66, 118.22, 72.63, 58.45, 30.46. ESI–MS calcd for $C_{17}H_{18}NO_2S$ [M + H]$^+$ 300.1053; found 300.1054

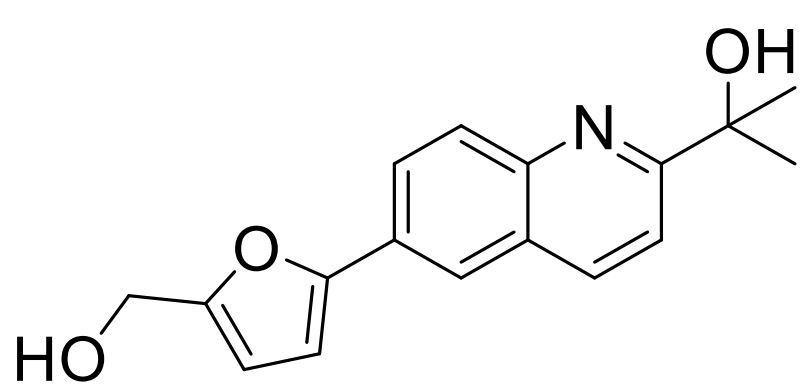

YKJ-154

2-(6-(5-(Hydroxymethyl)furan-2-yl)quinolin-2-yl)propan-2-ol

Yield: 47.3% ^{1}H NMR (400 MHz, DMSO) δ 8.38 (d, *J* = 8.7 Hz, 1H), 8.19 (d, *J* = 2.0 Hz, 1H), 8.06 (dd, *J* = 8.8, 2.0 Hz, 1H), 7.97 (d, *J* = 8.8 Hz, 1H), 7.86 (d, *J* = 8.7 Hz, 1H), 7.05 (d, *J* = 3.3 Hz, 1H), 6.47 (d, *J* = 3.3 Hz, 1H), 5.42 (s, 1H), 5.32 (t, *J* = 5.7 Hz, 1H), 4.50 (d, *J* = 5.7 Hz, 2H), 1.54 (s, 6H). ^{13}C NMR (101 MHz, DMSO) δ 168.12, 156.00, 151.77, 145.49, 136.52, 129.29, 127.79, 126.84, 125.51, 120.74, 118.44, 109.45, 107.66, 72.64, 55.78, 30.44. ESI–MS calcd for $C_{17}H_{18}NO_3$ [M + H]$^+$ 284.1281; found 284.1282

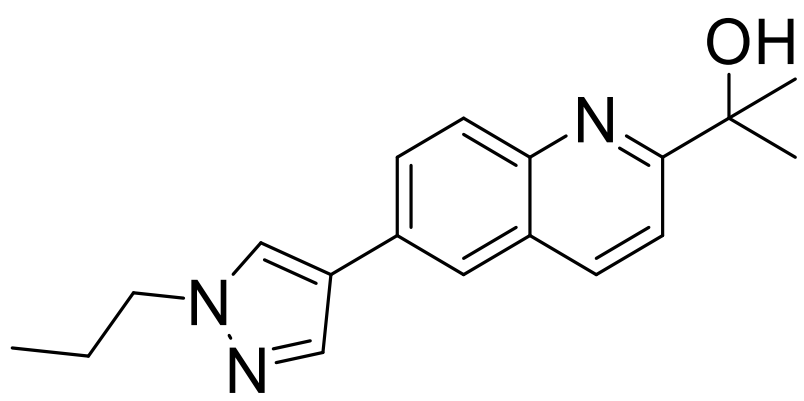

YKJ-155

2-(6-(1-Propyl-1*H*-pyrazol-4-yl)quinolin-2-yl)propan-2-ol

Yield: 83.8% ^{1}H NMR (400 MHz, DMSO) δ 8.33 (s, 1H), 8.26 (d, *J* = 8.6 Hz, 1H), 8.10 (d, *J* = 1.9 Hz, 1H), 8.02 (s, 1H), 8.00 – 7.90 (m, 2H), 7.83 (d, *J* = 8.6 Hz, 1H), 5.41 (s, 1H), 4.11 (t, *J* = 7.0 Hz, 2H), 1.84 (sextet, *J* = 7.2 Hz, 2H), 1.54 (s, 6H), 0.87 (t, *J* = 7.4 Hz, 3H). ^{13}C NMR (101 MHz, DMSO) δ 167.30, 144.94, 136.25, 135.98, 130.30, 129.06, 127.60, 127.50, 127.05, 121.90, 121.01, 118.13, 72.56, 53.09, 30.46, 23.13, 10.95. ESI–MS calcd for $C_{18}H_{22}N_3O$ $[M + H]^+$ 296.1757; found 296.1750

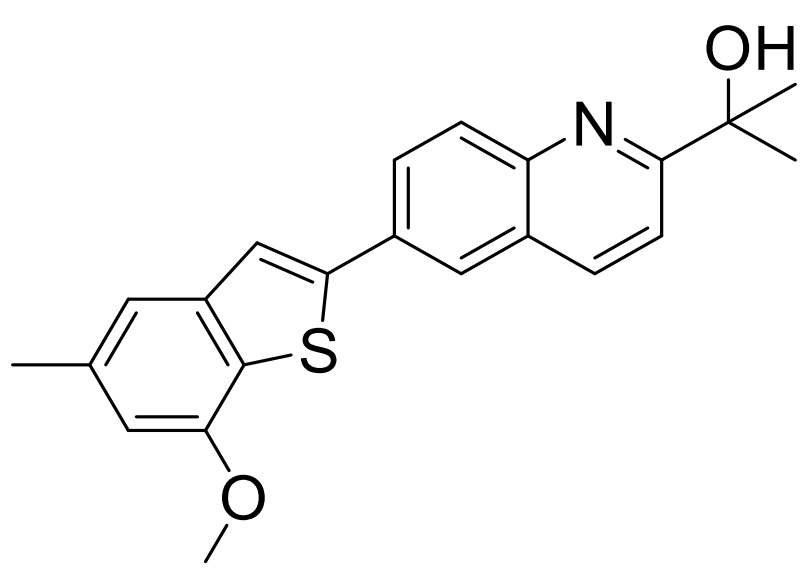

YKJ-156

2-(6-(7-Methoxy-5-methylbenzo[*b*]thiophen-2-yl)quinolin-2-yl)propan-2-ol

Yield: 99.3% ^{1}H NMR (400 MHz, DMSO) δ 8.41 (d, *J* = 8.7 Hz, 1H), 8.31 (d, *J* = 2.2 Hz, 1H), 8.17 (dd, *J* = 8.8, 2.1 Hz, 1H), 8.02 (d, *J* = 8.8 Hz, 1H), 7.93 (s, 1H), 7.90 (d, *J* = 8.6 Hz, 1H), 7.29 (s, 1H), 6.83 (s, 1H), 5.46 (s, 1H), 3.97 (s, 3H), 2.44 (s, 3H), 1.55 (s, 6H). ^{13}C NMR (101 MHz, DMSO) δ 168.77, 153.49, 146.02, 142.79, 142.07, 136.69, 136.09, 131.08, 129.49, 127.37, 126.86, 124.35, 121.12, 118.62, 116.31, 107.07, 72.70, 55.70, 30.43, 21.50 (one low field carbon not observed). ESI–MS calcd for $C_{22}H_{22}NO_2S$ $[M + H]^+$ 364.1366; found 364.1372

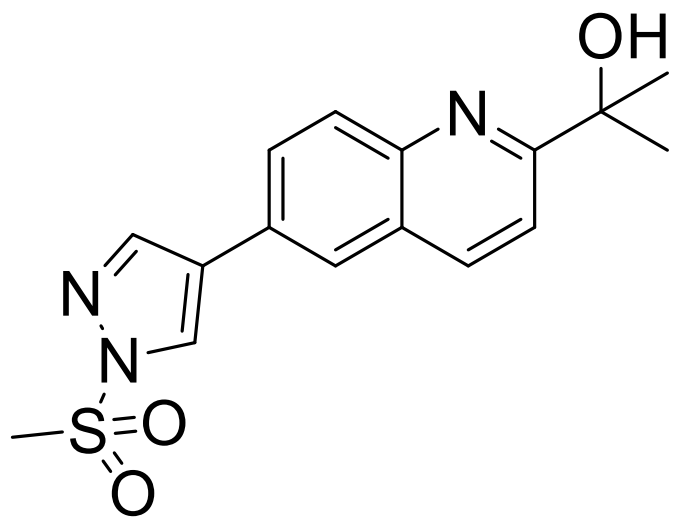

YKJ-157

2-(6-(1-(Methylsulfonyl)-1*H*-pyrazol-4-yl)quinolin-2-yl)propan-2-ol

Yield: 51.5% ^{1}H NMR (400 MHz, DMSO) δ 8.94 (s, 1H), 8.63 (s, 1H), 8.37 (d, *J* = 2.0 Hz, 1H), 8.29 (d, *J* = 8.7 Hz, 1H), 8.16 (dd, *J* = 8.7, 2.0 Hz, 1H), 7.98 (d, *J* = 8.8 Hz, 1H), 7.88 (d, *J* = 8.6 Hz, 1H), 5.44 (s, 1H), 3.61 (s, 3H), 1.54 (s, 6H). ^{13}C NMR (101 MHz, DMSO) δ 168.30, 145.60, 142.89, 136.30, 129.29, 128.29, 127.86, 124.12, 123.98, 118.42, 112.51, 72.65, 41.12, 30.45 (one low field carbon not observed). ESI–MS calcd for $C_{16}H_{18}N_3O_3S$ $[M + H]^+$ 332.1063; found 332.1063

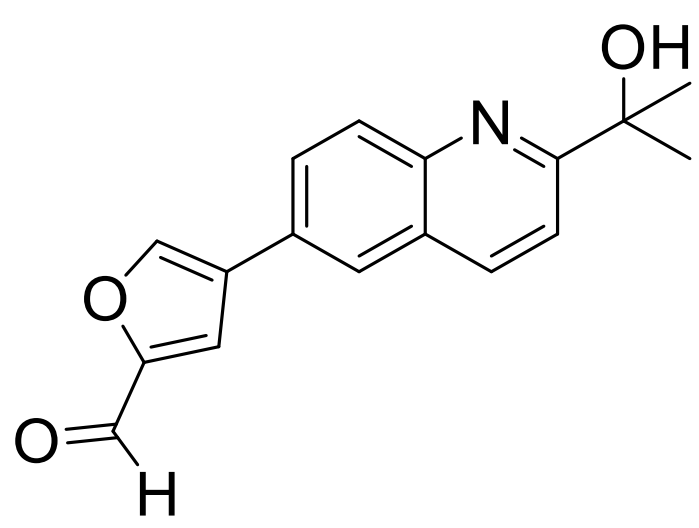

YKJ-158

4-(2-(2-Hydroxypropan-2-yl)quinolin-6-yl)furan-2-carboxaldehyde

Yield: 92.9% ^{1}H NMR (500 MHz, DMSO) δ 9.70 (s, 1H), 8.78 (s, 1H), 8.35 – 8.29 (m, 2H), 8.17 (s, 1H), 8.09 (dd, *J* = 8.7, 2.1 Hz, 1H), 8.01 (d, *J* = 8.7 Hz, 1H), 7.89 (d, *J* = 8.6 Hz, 1H), 5.45 (s, 1H), 1.55 (s, 6H). ^{13}C NMR (126 MHz, DMSO) δ 178.81, 168.50, 153.36, 145.81, 145.79, 136.45, 129.39, 127.92, 127.83, 127.54, 126.84, 124.10, 120.30, 118.52, 72.70, 30.47. ESI–MS calcd for $C_{17}H_{16}NO_3$ $[M + H]^+$ 282.1125; found 282.1124

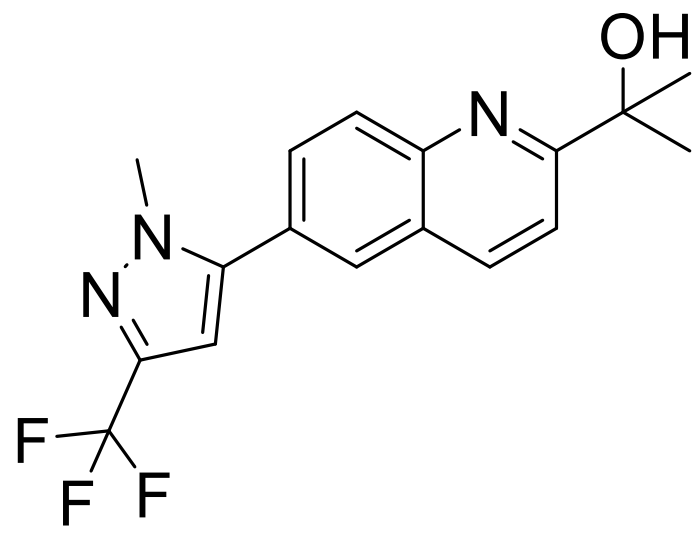

YKJ-159

2-(6-(1-Methyl-3-(trifluoromethyl)-1*H*-pyrazol-5-yl)quinolin-2-yl)propan-2-ol

Yield: 91.7% ^{1}H NMR (400 MHz, DMSO) δ 8.42 (d, *J* = 8.7 Hz, 1H), 8.23 (d, *J* = 2.0 Hz, 1H), 8.08 (d, *J* = 8.7 Hz, 1H), 7.98 – 7.90 (m, 2H), 7.06 (s, 1H), 5.49 (s, 1H), 4.02 (s, 3H), 1.56 (s, 6H). ^{13}C NMR (126 MHz, DMSO) δ 169.66, 146.03, 144.33, 139.66 (q, *J* = 37.8 Hz), 136.95, 129.61, 129.29, 128.23, 126.40, 126.04, 121.57 (q, *J* = 268.4 Hz), 118.69, 104.85 (d, *J* = 2.5 Hz), 72.80, 38.56, 30.47. ESI–MS calcd for $C_{17}H_{17}F_3N_3O$ $[M + H]^+$ 336.1318; found 336.1319

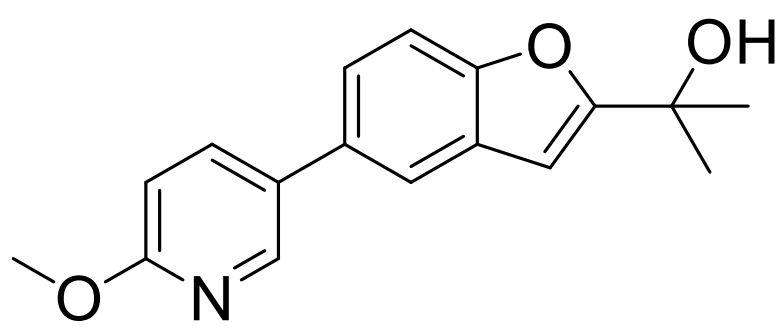

YKJ-160

2-(5-(6-Methoxypyridin-3-yl)benzofuran-2-yl)propan-2-ol

Yield: 72.9% ^{1}H NMR (400 MHz, DMSO) δ 8.47 (s, 1H), 8.01 (d, *J* = 8.6 Hz, 1H), 7.82 (s, 1H), 7.61 (d, *J* = 8.6 Hz, 1H), 7.51 (d, *J* = 8.6 Hz, 1H), 6.91 (d, *J* = 8.6 Hz, 1H), 6.70 (s, 1H), 5.44 (s, 1H), 3.90 (s, 3H), 1.53 (s, 6H). ^{13}C NMR (101 MHz, DMSO) δ 165.65, 162.74, 153.56, 144.65, 137.79, 131.98, 129.87, 128.99, 122.48, 118.67, 111.35, 110.48, 100.22, 67.58, 53.22, 28.97. ESI–MS calcd for $C_{17}H_{18}NO_3$ $[M + H]^+$ 284.1281 found 284.1286

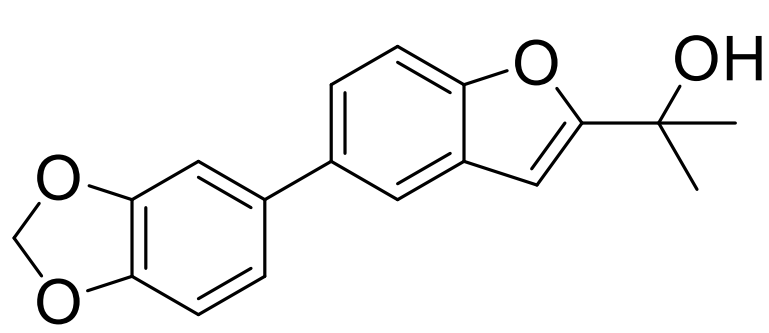

YKJ-161

2-(5-(Benzo[*d*][1,3]dioxol-5-yl)benzofuran-2-yl)propan-2-ol

Yield: quantitative ^{1}H NMR (400 MHz, DMSO) δ 7.76 (s, 1H), 7.55 (d, *J* = 8.6 Hz, 1H), 7.45 (d, *J* = 8.6 Hz, 1H), 7.23 (s, 1H), 7.13 (d, *J* = 8.6 Hz, 1H), 6.99 (d, *J* = 8.1 Hz, 1H), 6.68 (s, 1H), 6.06 (s, 2H), 5.42 (s, 1H), 1.53 (s, 6H). ^{13}C NMR (101 MHz, DMSO) δ 165.50, 153.37, 147.88, 146.42, 135.07, 128.81, 122.67, 120.31, 118.71, 111.05, 108.59, 107.42, 101.04, 100.25, 67.57, 28.98. ESI–MS calcd for $C_{18}H_{15}O_3$ $[M - OH]^+$ 279.1016 found 279.1016

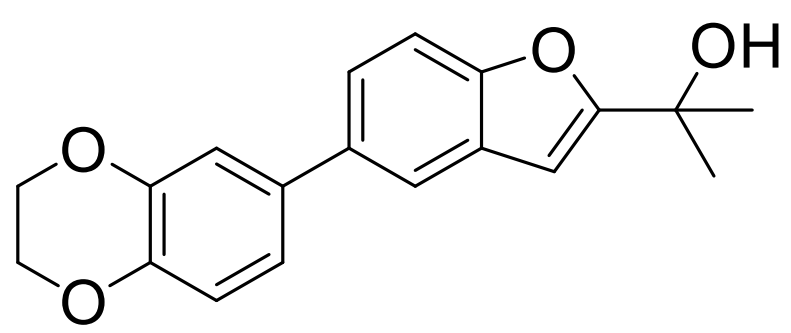

YKJ-162

2-(5-(2,3-Dihydrobenzo[*b*][1,4]dioxin-6-yl)benzofuran-2-yl)propan-2-ol

Yield: 89.1% ^{1}H NMR (400 MHz, DMSO) δ 7.74 (d, *J* = 1.8 Hz, 1H), 7.54 (d, *J* = 8.6 Hz, 1H), 7.44 (dd, *J* = 8.6, 2.0 Hz, 1H), 7.15 – 7.09 (m, 2H), 6.93 (d, *J* = 8.1 Hz, 1H), 6.68 (d, *J* = 1.0 Hz, 1H), 5.42 (s, 1H), 4.27 (s, 4H), 1.53 (s, 6H). ^{13}C NMR (101 MHz, DMSO) δ 165.46, 153.37, 143.60, 142.71, 134.78, 134.12, 128.85, 122.48, 119.70, 118.51, 117.41, 115.32, 111.07, 100.29, 67.59, 64.14, 28.99. ESI–MS calcd for $C_{19}H_{17}O_3$ $[M - OH]^+$ 293.1172; found 293.1173

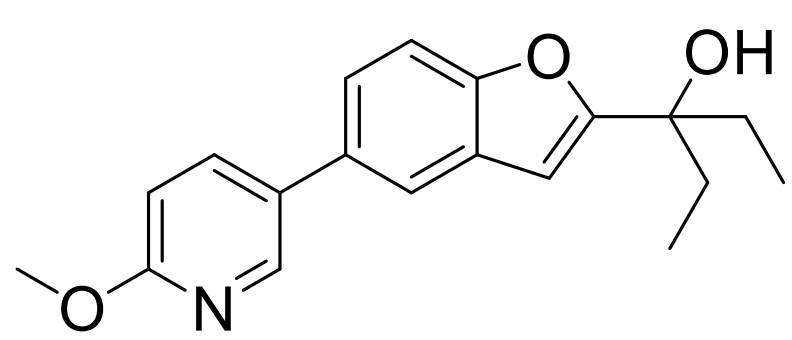

YKJ-163

3-(5-(6-Methoxypyridin-3-yl)benzofuran-2-yl)pentan-3-ol

Yield: quantitative ^{1}H NMR (500 MHz, DMSO) δ 8.47 (d, *J* = 2.6 Hz, 1H), 8.01 (dd, *J* = 8.6, 2.6 Hz, 1H), 7.81 (d, *J* = 1.9 Hz, 1H), 7.59 (d, *J* = 8.5 Hz, 1H), 7.47 (dd, *J* = 8.5, 1.9 Hz, 1H), 6.91 (d, *J* = 8.5 Hz, 1H), 6.73 (d, *J* = 0.9 Hz, 1H), 5.09 (s, 1H), 3.89 (s, 3H), 1.91 – 1.69 (m, 4H), 0.76 (t, *J* = 7.4 Hz, 6H). ^{13}C NMR (126 MHz, DMSO) δ 163.67, 162.75, 153.74, 144.69, 137.86, 132.03, 130.00, 128.93, 122.27, 118.57, 111.29, 110.50, 102.86, 73.83, 53.24, 32.00, 7.96. ESI–MS calcd for $C_{19}H_{22}NO_3$ $[M + H]^+$ 312.1594; found 312.1600

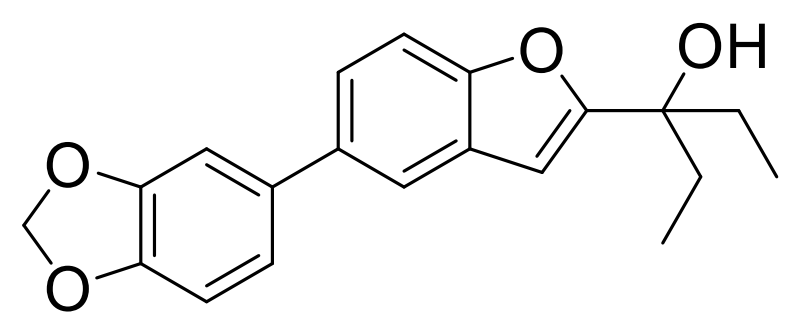

YKJ-164

3-(5-(Benzo[*d*][1,3]dioxol-5-yl)benzofuran-2-yl)pentan-3-ol

Yield: 97.3% ^{1}H NMR (400 MHz, DMSO) δ 7.75 (d, *J* = 1.9 Hz, 1H), 7.53 (d, *J* = 8.6 Hz, 1H), 7.42 (dd, *J* = 8.5, 2.0 Hz, 1H), 7.23 (d, *J* = 1.8 Hz, 1H), 7.12 (dd, *J* = 8.1, 1.9 Hz, 1H), 6.99 (d, *J* = 8.1 Hz, 1H), 6.71 (s, 1H), 6.05 (s, 2H), 5.06 (s, 1H), 1.80 (m, 4H), 0.76 (t, *J* = 7.4 Hz, 6H). ^{13}C NMR (101 MHz, DMSO) δ 163.52, 153.53, 147.86, 146.41, 135.18, 135.10, 128.74, 122.41, 120.33, 118.55, 110.96, 108.58, 107.45, 102.84, 101.03, 73.80, 31.96, 7.93. ESI–MS calcd for $C_{20}H_{19}O_3$ [M - OH]$^+$ 307.1329; found 307.1331

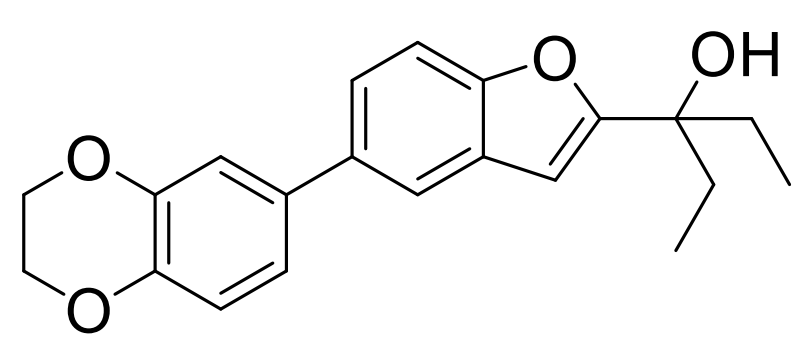

YKJ-165

3-(5-(2,3-Dihydrobenzo[*b*][1,4]dioxin-6-yl)benzofuran-2-yl)pentan-3-ol

Yield: 91.3% ^{1}H NMR (400 MHz, DMSO) δ 7.74 (d, *J* = 1.9 Hz, 1H), 7.52 (d, *J* = 8.5 Hz, 1H), 7.41 (dd, *J* = 8.6, 2.0 Hz, 1H), 7.15 – 7.09 (m, 2H), 6.92 (d, *J* = 8.1 Hz, 1H), 6.71 (d, *J* = 0.9 Hz, 1H), 5.06 (s, 1H), 4.27 (s, 4H), 1.80 (m, 4H), 0.76 (t, *J* = 7.4 Hz, 6H). ^{13}C NMR (101 MHz, DMSO) δ 163.47, 153.50, 143.58, 142.68, 134.79, 134.20, 128.76, 122.20, 119.70, 118.33, 117.37, 115.33, 110.96, 102.85, 73.78, 64.11, 31.95, 7.92. ESI–MS calcd for $C_{21}H_{21}O_3$ [M - OH]$^+$ 321.1485; found 321.1488

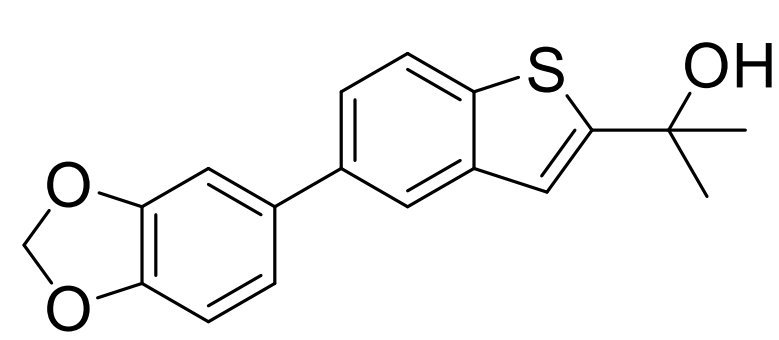

YKJ-166

2-(5-(Benzo[*d*][1,3]dioxol-5-yl)benzo[*b*]thiophen-2-yl)propan-2-ol

Yield: 94.5% ^{1}H NMR (500 MHz, DMSO) δ 7.94 (d, *J* = 1.9 Hz, 1H), 7.90 (d, *J* = 8.4 Hz, 1H), 7.51 (dd, *J* = 8.4, 1.9 Hz, 1H), 7.28 (d, *J* = 1.9 Hz, 1H), 7.22 (s, 1H), 7.18 (dd, *J* = 8.1, 1.9 Hz, 1H), 7.01 (d, *J* = 8.1 Hz, 1H), 6.06 (s, 2H), 5.65 (s, 1H), 1.58 (s, 6H). ^{13}C NMR (126 MHz, DMSO) δ 158.06, 147.98, 146.66, 140.51, 137.23, 136.25, 134.80, 122.61, 122.59, 120.83, 120.33, 117.91, 108.70, 107.31, 101.12, 69.96, 32.08. ESI–MS calcd for $C_{18}H_{15}O_2S$ [M - OH]$^+$ 295.0787; found 295.0791

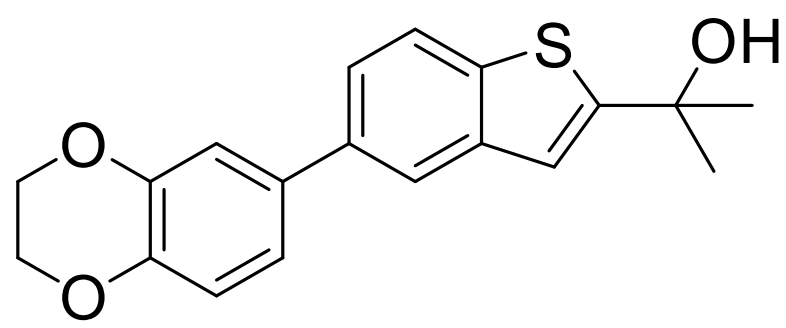

YKJ-167

2-(5-(2,3-Dihydrobenzo[*b*][1,4]dioxin-6-yl)benzo[*b*]thiophen-2-yl)propan-2-ol

Yield: 85.2% ^{1}H NMR (400 MHz, DMSO) δ 7.95 – 7.86 (m, 2H), 7.50 (dd, *J* = 8.4, 1.9 Hz, 1H), 7.23 (s, 1H), 7.20 – 7.13 (m, 2H), 6.94 (d, *J* = 7.9 Hz, 1H), 5.63 (s, 1H), 4.28 (s, 4H), 1.57 (s, 6H). ^{13}C NMR (101 MHz, DMSO) δ 157.96, 143.65, 142.91, 140.50, 137.14, 135.92, 133.79, 122.55, 122.38, 120.59, 119.60, 117.91, 117.46, 115.22, 69.92, 64.13, 32.05. ESI–MS calcd for $C_{19}H_{17}O_2S$ [M - OH]$^+$ 309.0944; found 309.0944

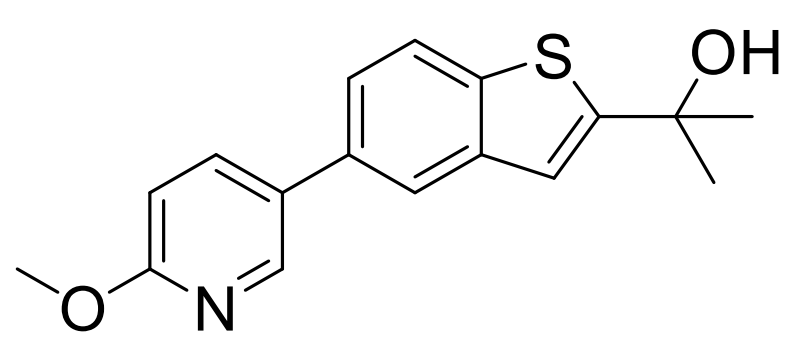

YKJ-168

2-(5-(6-Methoxypyridin-3-yl)benzo[*b*]thiophen-2-yl)propan-2-ol

Yield: quantitative ^{1}H NMR (400 MHz, DMSO) δ 8.52 (d, *J* = 2.6 Hz, 1H), 8.05 (dd, *J* = 8.6, 2.6 Hz, 1H), 8.00 (d, *J* = 1.8 Hz, 1H), 7.96 (d, *J* = 8.3 Hz, 1H), 7.56 (dd, *J* = 8.3, 1.8 Hz, 1H), 7.25 (s, 1H), 6.93 (d, *J* = 8.6 Hz, 1H), 5.66 (s, 1H), 3.90 (s, 3H), 1.58 (s, 6H). ^{13}C NMR (101 MHz, DMSO) δ 162.90, 158.27, 144.68, 140.57, 137.69, 137.58, 133.18, 129.61, 122.84, 122.27, 120.72, 117.83, 110.57, 69.95, 53.24, 32.05. ESI–MS calcd for $C_{17}H_{18}NO_2S$ [M + H]$^+$ 300.1053; found 300.1058

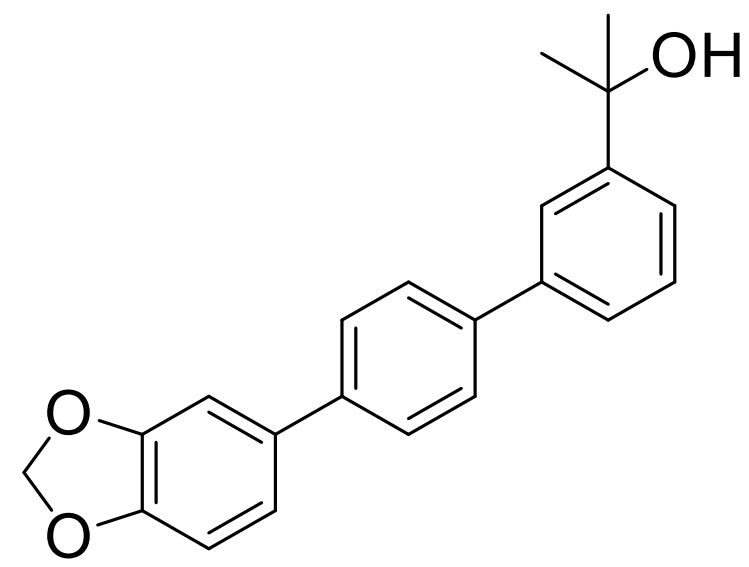

YKJ-169

2-(4'-(Benzo[*d*][1,3]dioxol-5-yl)-[1,1'-biphenyl]-3-yl)propan-2-ol

Yield: 71.0% ^{1}H NMR (500 MHz, DMSO) δ 7.78 (t, *J* = 1.8 Hz, 1H), 7.70 (bs, 4H), 7.51 (dt, *J* = 7.5, 1.6 Hz, 1H), 7.46 (dt, *J* = 7.8, 1.5 Hz, 1H), 7.40 (t, *J* = 7.6 Hz, 1H), 7.30 (d, *J* = 1.9 Hz, 1H), 7.21 (dd, *J* = 8.1, 1.9 Hz, 1H), 7.02 (d, *J* = 8.1 Hz, 1H), 6.07 (s, 2H), 5.10 (s, 1H), 1.48 (s, 6H). ^{13}C NMR (126 MHz, DMSO) δ 151.31, 148.06, 146.94, 139.24, 139.15, 138.77, 133.95, 128.46, 127.16, 126.91, 124.19, 123.83, 122.83, 120.18, 108.75, 107.01, 101.19, 70.82, 32.00. ESI–MS calcd for $C_{22}H_{19}O_2$ [M - OH]$^+$ 315.1380; found 315.1380

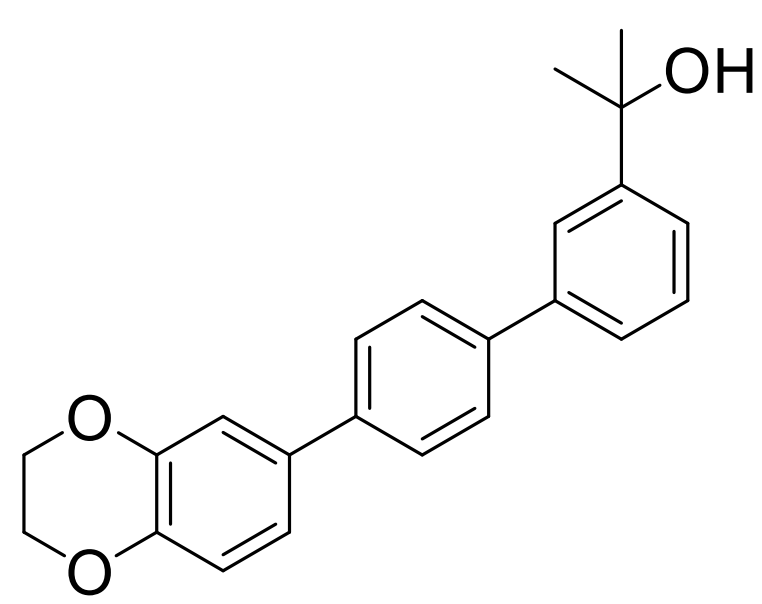

YKJ-170

2-(4'-(2,3-Dihydrobenzo[*b*][1,4]dioxin-6-yl)-[1,1'-biphenyl]-3-yl)propan-2-ol

Yield: 80.8% ^{1}H NMR (400 MHz, DMSO) δ 7.78 (s, 1H), 7.70 (bs, 4H), 7.54 – 7.35 (m, 3H), 7.24 – 7.16 (m, 2H), 6.95 (d, *J* = 8.2 Hz, 1H), 5.08 (s, 1H), 4.29 (s, 4H), 1.48 (s, 6H). ^{13}C NMR (101 MHz, DMSO) δ 151.27, 143.71, 143.19, 139.15, 138.44, 132.92, 128.40, 127.13, 126.67, 124.13, 123.77, 122.80, 119.40, 117.52, 114.96, 70.76, 64.17, 64.12, 31.96 (one low field carbon not observed). ESI–MS calcd for $C_{23}H_{21}O_2$ [M - OH]$^+$ 329.1536; found 329.1538

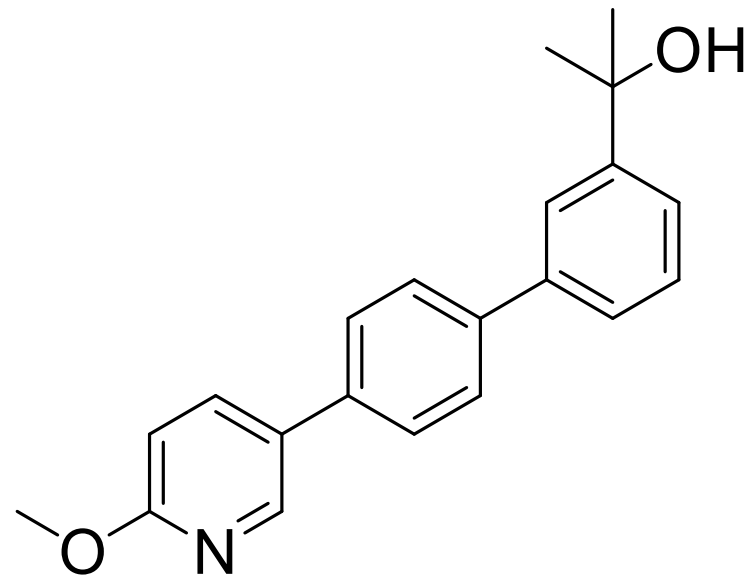

YKJ-171

2-(4'-(6-Methoxypyridin-3-yl)-[1,1'-biphenyl]-3-yl)propan-2-ol

Yield: quantitative ^{1}H NMR (400 MHz, DMSO) δ 8.55 (d, *J* = 2.6 Hz, 1H), 8.07 (dd, *J* = 8.6, 2.6 Hz, 1H), 7.79 (t, *J* = 1.9 Hz, 1H), 7.76 (s, 4H), 7.54 – 7.45 (m, 2H), 7.43 – 7.38 (m, 1H), 6.93 (d, *J* = 8.6 Hz, 1H), 5.09 (s, 1H), 3.91 (s, 3H), 1.49 (s, 6H). ^{13}C NMR (101 MHz, DMSO) δ 163.10, 151.31, 144.60, 139.63, 139.05, 137.42, 135.84, 128.81, 128.43, 127.32, 126.75, 124.21, 123.89, 122.85, 110.63, 70.77, 53.28, 31.96. ESI–MS calcd for $C_{21}H_{22}NO_2$ $[M + H]^+$ 320.1645; found 320.1648

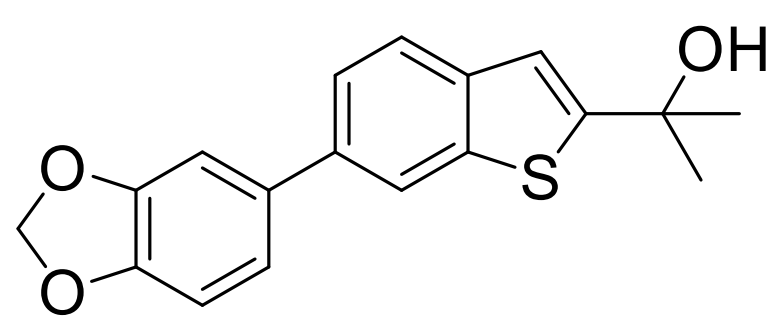

YKJ-172

2-(6-(Benzo[*d*][1,3]dioxol-5-yl)benzo[*b*]thiophen-2-yl)propan-2-ol

Yield: 89.9% ^{1}H NMR (400 MHz, DMSO) δ 8.14 – 8.09 (m, 1H), 7.76 (d, *J* = 8.3 Hz, 1H), 7.57 (dd, *J* = 8.3, 1.7 Hz, 1H), 7.32 (d, *J* = 1.8 Hz, 1H), 7.23 – 7.19 (m, 2H), 7.00 (d, *J* = 8.1 Hz, 1H), 6.06 (s, 2H), 5.63 (s, 1H), 1.57 (s, 6H). ^{13}C NMR (101 MHz, DMSO) δ 157.60, 147.99, 146.67, 139.32, 138.76, 135.52, 134.49, 123.35, 123.06, 120.33, 119.83, 117.34, 108.67, 107.24, 101.11, 69.96, 32.05. ESI–MS calcd for $C_{18}H_{15}O_2S$ $[M - OH]^+$ 295.0787 found 295.0788

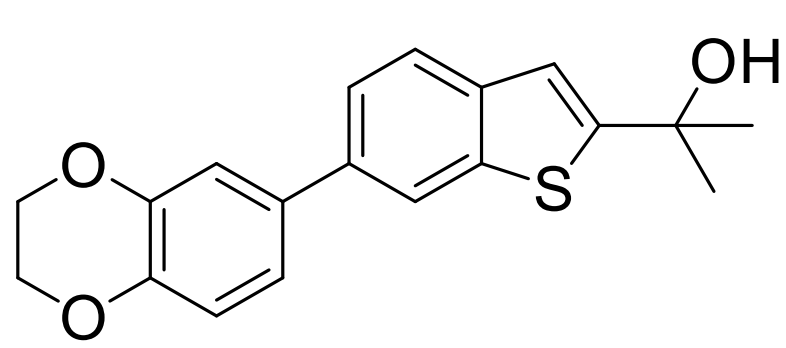

YKJ-173

2-(6-(2,3-Dihydrobenzo[*b*][1,4]dioxin-6-yl)benzo[*b*]thiophen-2-yl)propan-2-ol

Yield: 87.2% ^{1}H NMR (400 MHz, DMSO) δ 8.13 – 8.08 (m, 1H), 7.75 (d, *J* = 8.3 Hz, 1H), 7.55 (dd, *J* = 8.3, 1.8 Hz, 1H), 7.23 – 7.17 (m, 3H), 6.94 (d, *J* = 8.2 Hz, 1H), 5.63 (s, 1H), 4.28 (s, 4H), 1.57 (s, 6H). ^{13}C NMR (101 MHz, DMSO) δ 157.53, 143.68, 142.96, 139.35, 138.72, 135.23, 133.50, 123.36, 122.87, 119.63, 117.48, 117.34, 115.21, 69.96, 64.16, 64.12, 32.06 (one low field carbon not observed). ESI–MS calcd for $C_{19}H_{17}O_2S$ [M - OH]$^+$ 309.0944; found 309.0944

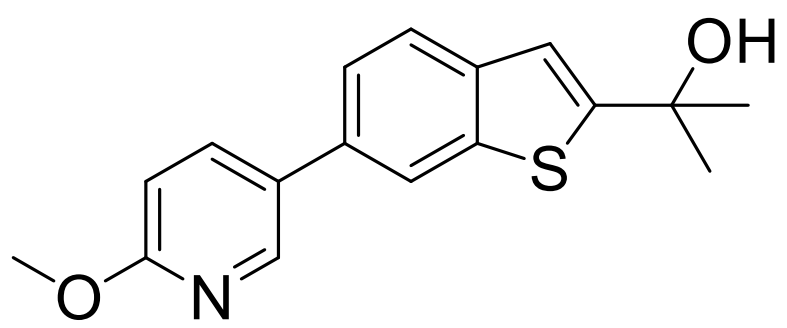

YKJ-174

2-(6-(6-Methoxypyridin-3-yl)benzo[*b*]thiophen-2-yl)propan-2-ol

Yield: 89.2% ^{1}H NMR (400 MHz, DMSO) δ 8.55 (d, *J* = 2.6 Hz, 1H), 8.19 (d, *J* = 1.7 Hz, 1H), 8.08 (dd, *J* = 8.6, 2.6 Hz, 1H), 7.81 (d, *J* = 8.3 Hz, 1H), 7.62 (dd, *J* = 8.3, 1.7 Hz, 1H), 7.23 (s, 1H), 6.92 (d, *J* = 8.6 Hz, 1H), 5.65 (s, 1H), 3.90 (s, 3H), 1.58 (s, 6H). ^{13}C NMR (101 MHz, DMSO) δ 162.91, 157.89, 144.72, 139.43, 139.05, 137.65, 132.51, 129.36, 123.61, 122.79, 119.78, 117.39, 110.58, 69.98, 53.27, 32.05. ESI–MS calcd for $C_{17}H_{18}NO_2S$ [M + H]$^+$ 300.1053; found 300.1050

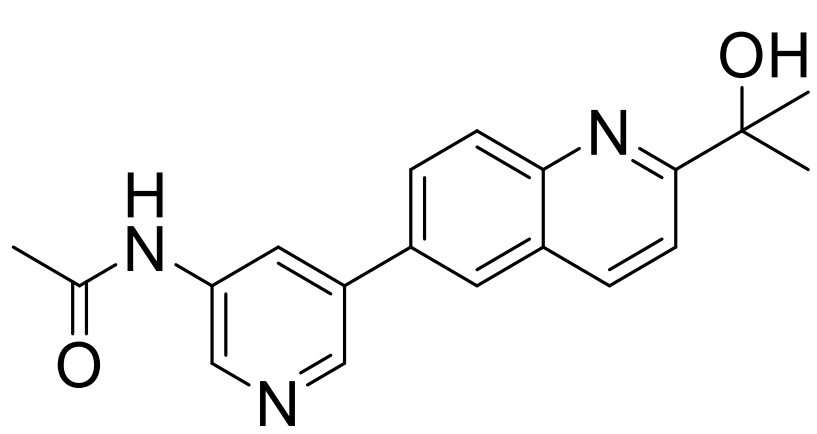

YKJ-175

N-(5-(2-(2-Hydroxypropan-2-yl)quinolin-6-yl)pyridin-3-yl)acetamide

Yield: 86.1% ^{1}H NMR (400 MHz, DMSO) δ 10.33 (s, 1H), 8.80 – 8.64 (m, 2H), 8.50 – 8.40 (m, 2H), 8.27 (d, *J* = 2.2 Hz, 1H), 8.09 (d, *J* = 8.7 Hz, 1H), 8.02 (dd, *J* = 8.8, 2.1 Hz, 1H), 7.92 (d, *J* = 8.6 Hz, 1H), 5.48 (s, 1H), 2.13 (s, 3H), 1.56 (s, 6H). ^{13}C NMR (101 MHz, DMSO) δ 169.16, 168.90, 145.92, 142.37, 139.74, 136.88, 136.13, 134.94, 134.38, 129.63, 128.23, 126.85, 125.67, 124.01, 118.48, 72.74, 30.47, 23.96. ESI–MS calcd for $C_{19}H_{20}N_3O_2$ [M + H]$^+$ 322.1550; found 322.1550

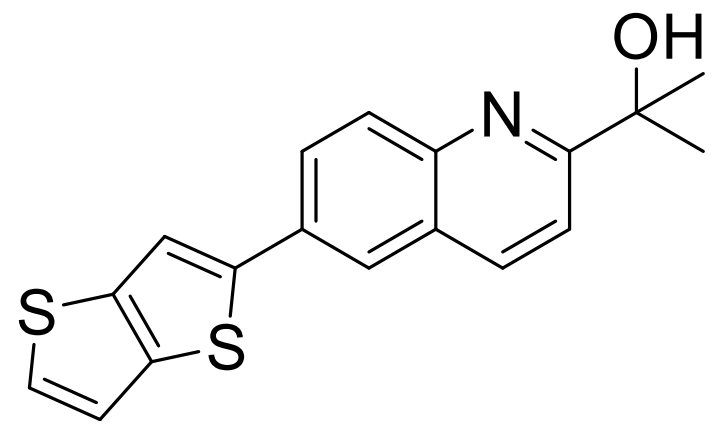

YKJ-176

2-(6-(Thieno[3,2-*b*]thiophen-2-yl)quinolin-2-yl)propan-2-ol

Yield: 91.8% ^{1}H NMR (400 MHz, DMSO) δ 8.40 (d, *J* = 8.6 Hz, 1H), 8.22 (d, *J* = 2.2 Hz, 1H), 8.10 (dd, *J* = 8.8, 2.2 Hz, 1H), 8.07 (s, 1H), 8.01 (d, *J* = 8.8 Hz, 1H), 7.88 (d, *J* = 8.6 Hz, 1H), 7.72 (d, *J* = 5.3 Hz, 1H), 7.50 (d, *J* = 5.2 Hz, 1H), 5.45 (s, 1H), 1.55 (s, 6H). ^{13}C NMR (101 MHz, DMSO) δ 168.50, 145.80, 144.48, 139.92, 138.39, 136.56, 131.67, 129.54, 128.81, 127.06, 126.97, 123.15, 120.22, 118.61, 117.18, 72.69, 30.44. ESI–MS calcd for $C_{18}H_{16}NOS_2$ $[M + H]^+$ 326.0668; found 326.0668

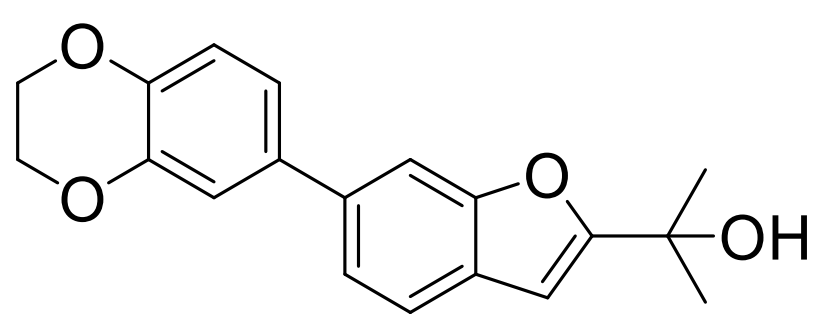

YKJ-177

2-(6-(2,3-Dihydrobenzo[*b*][1,4]dioxin-6-yl)benzofuran-2-yl)propan-2-ol

Yield: 62.2% ^{1}H NMR (400 MHz, DMSO) δ 7.73 (s, 1H), 7.58 (d, *J* = 8.1 Hz, 1H), 7.44 (dd, *J* = 8.1, 1.6 Hz, 1H), 7.24 – 7.15 (m, 2H), 6.93 (d, *J* = 8.2 Hz, 1H), 6.67 (s, 1H), 5.41 (s, 1H), 4.27 (s, 4H), 1.53 (s, 6H). ^{13}C NMR (101 MHz, DMSO) δ 165.27, 154.73, 143.64, 142.92, 135.79, 133.74, 127.13, 121.41, 120.97, 119.69, 117.45, 115.30, 108.55, 99.94, 67.57, 64.15, 64.11, 29.01.

ESI–MS calcd for $C_{19}H_{17}O_3$ $[M - OH]^+$ 293.1172; found 293.1175

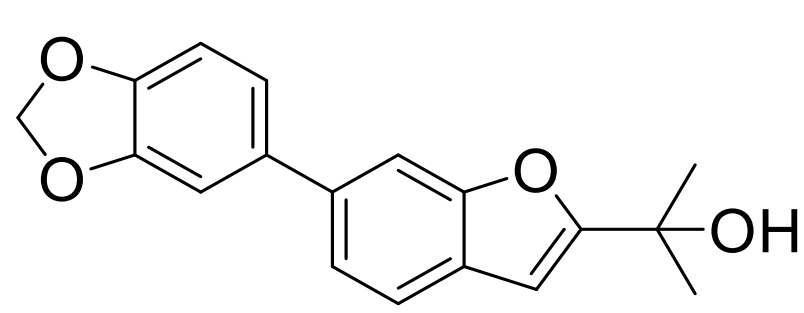

YKJ-178

2-(6-(Benzo[*d*][1,3]dioxol-5-yl)benzofuran-2-yl)propan-2-ol

Yield: 85.7% ^{1}H NMR (400 MHz, DMSO) δ 7.75 (s, 1H), 7.59 (d, *J* = 8.1 Hz, 1H), 7.46 (dd, *J* = 8.0, 1.6 Hz, 1H), 7.30 (d, *J* = 1.8 Hz, 1H), 7.19 (dd, *J* = 8.0, 1.9 Hz, 1H), 6.99 (d, *J* = 8.1 Hz, 1H), 6.67 (s, 1H), 6.06 (s, 2H), 5.42 (s, 1H), 1.53 (s, 6H). ^{13}C NMR (101 MHz, DMSO) δ 165.32, 154.71, 147.96, 146.64, 136.07, 134.72, 127.19, 121.61, 120.97, 120.37, 108.77, 108.65, 107.37, 101.09, 99.94, 67.58, 29.01. ESI–MS calcd for $C_{18}H_{15}O_3$ $[M - OH]^+$ 279.1016; found 279.1016

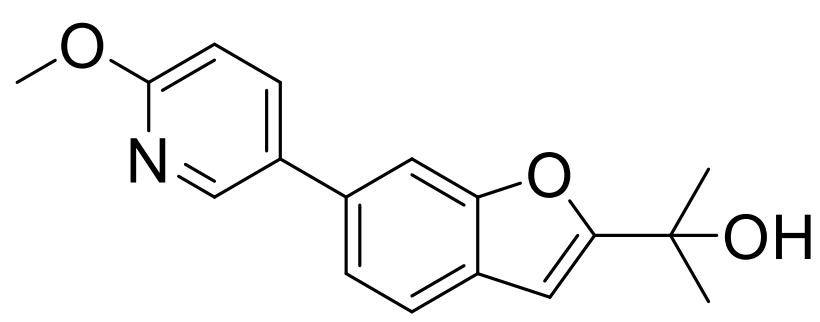

YKJ-179

2-(6-(6-Methoxypyridin-3-yl)benzofuran-2-yl)propan-2-ol

Yield: 60.1% ^{1}H NMR (400 MHz, DMSO) δ 8.54 (d, *J* = 2.6 Hz, 1H), 8.07 (dd, *J* = 8.6, 2.6 Hz, 1H), 7.84 (s, 1H), 7.64 (d, *J* = 8.1 Hz, 1H), 7.51 (dd, *J* = 8.1, 1.6 Hz, 1H), 6.91 (d, *J* = 8.6 Hz, 1H), 6.69 (d, *J* = 0.9 Hz, 1H), 5.44 (s, 1H), 3.90 (s, 3H), 1.53 (s, 6H). ^{13}C NMR (101 MHz, DMSO) δ 165.49, 162.87, 154.73, 144.75, 137.71, 132.94, 129.56, 127.53, 121.36, 121.26, 110.55, 108.69, 99.98, 67.60, 53.26, 29.00. ESI–MS calcd for $C_{17}H_{18}NO_3$ $[M + H]^+$ 284.1281; found 284.1285

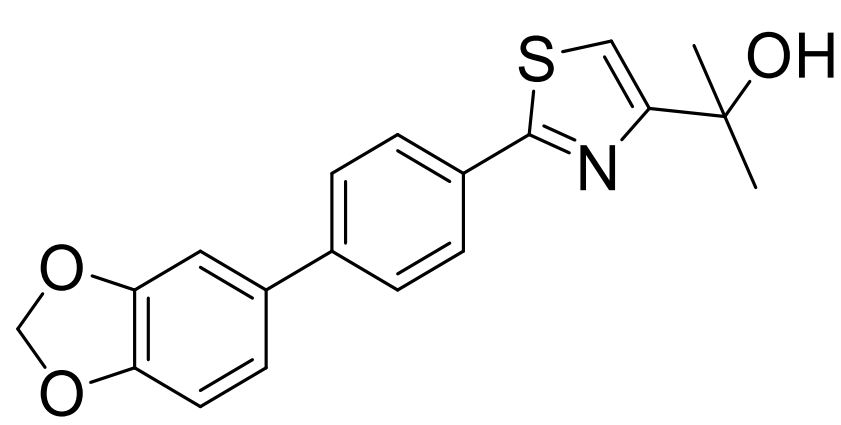

YKJ-180

2-(2-(4-(Benzo[*d*][1,3]dioxol-5-yl)phenyl)thiazol-4-yl)propan-2-ol

Yield: 98.8% ^{1}H NMR (400 MHz, DMSO) δ 8.00 – 7.90 (m, 2H), 7.80 – 7.68 (m, 2H), 7.42 (s, 1H), 7.33 (d, *J* = 1.8 Hz, 1H), 7.23 (dd, *J* = 8.1, 1.9 Hz, 1H), 7.03 (d, *J* = 8.2 Hz, 1H), 6.08 (s, 2H), 5.22 (s, 1H), 1.52 (s, 6H). ^{13}C NMR (101 MHz, DMSO) δ 166.37, 165.89, 148.11, 147.27, 141.18, 133.38, 131.90, 127.01, 126.47, 120.41, 112.63, 108.77, 106.99, 101.28, 70.45, 30.58. ESI–MS calcd for $C_{19}H_{18}NO_3S$ $[M + H]^+$ 340.1002; found 340.1002

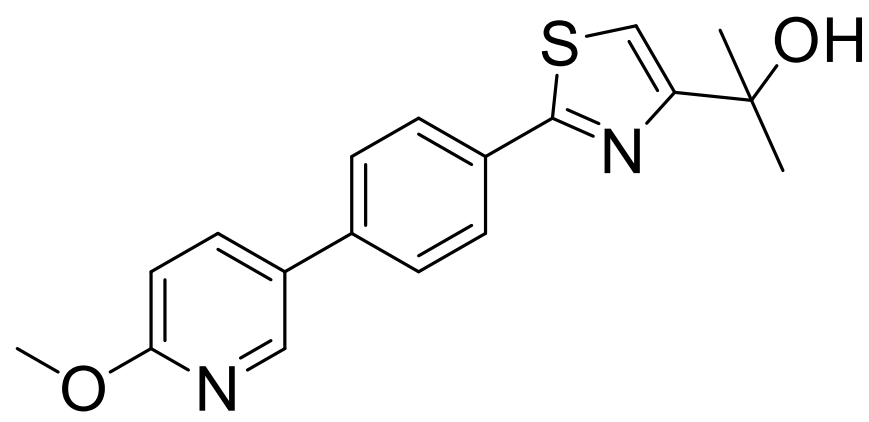

YKJ-181

2-(2-(4-(6-Methoxypyridin-3-yl)phenyl)thiazol-4-yl)propan-2-ol

Yield: 97.3% ^{1}H NMR (400 MHz, DMSO) δ 8.57 (s, 1H), 8.09 (dd, *J* = 8.6, 2.7 Hz, 1H), 7.99 (d, *J* = 8.5 Hz, 2H), 7.80 (d, *J* = 8.1 Hz, 2H), 7.43 (s, 1H), 6.95 (d, *J* = 8.5 Hz, 1H), 5.23 (s, 1H), 3.91 (s, 3H), 1.52 (s, 6H). ^{13}C NMR (101 MHz, DMSO) δ 166.42, 165.78, 163.35, 144.83, 138.35, 137.49, 132.28, 128.36, 126.90, 126.64, 112.81, 110.73, 70.46, 53.36, 30.59. ESI–MS calcd for $C_{18}H_{19}N_2O_2S$ $[M + H]^+$ 327.1162; found 327.1162

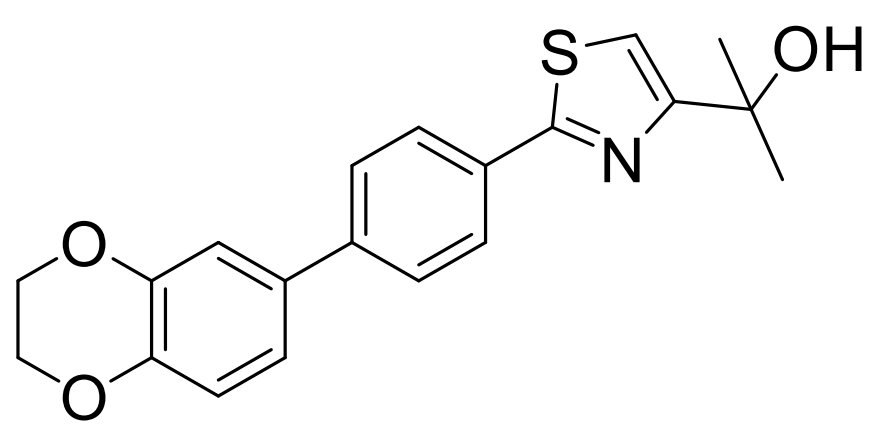

YKJ-182

2-(2-(4-(2,3-Dihydrobenzo[*b*][1,4]dioxin-6-yl)phenyl)thiazol-4-yl)propan-2-ol

Yield: 95.6% ^{1}H NMR (400 MHz, DMSO) δ 7.98 – 7.91 (m, 2H), 7.76 – 7.70 (m, 2H), 7.41 (s, 1H), 7.26 – 7.18 (m, 2H), 6.96 (d, *J* = 8.3 Hz, 1H), 5.22 (s, 1H), 4.29 (s, 4H), 1.51 (s, 6H). ^{13}C NMR (101 MHz, DMSO) δ 166.37, 165.94, 143.77, 143.57, 140.89, 132.38, 131.84, 126.80, 126.50, 119.54, 117.62, 115.09, 112.60, 70.47, 64.22, 64.12, 30.59. ESI–MS calcd for $C_{20}H_{20}NO_3S$ $[M + H]^+$ 354.1158; found 354.1159

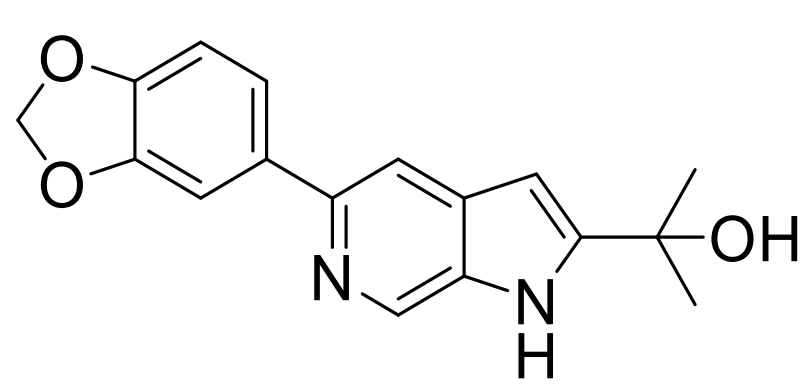

YKJ-183

2-(5-(Benzo[*d*][1,3]dioxol-5-yl)-1*H*-pyrrolo[2,3-*c*]pyridin-2-yl)propan-2-ol

Yield: 98.2% ^{1}H NMR (400 MHz, DMSO) δ 11.48 (s, 1H), 8.66 (s, 1H), 7.92 (s, 1H), 7.60 (d, *J* = 1.7 Hz, 1H), 7.56 (dd, *J* = 8.2, 1.8 Hz, 1H), 6.97 (d, *J* = 8.1 Hz, 1H), 6.34 (d, *J* = 2.2 Hz, 1H), 6.05 (s, 2H), 5.45 (s, 1H), 1.55 (s, 6H). ^{13}C NMR (101 MHz, DMSO) δ 153.61, 147.72, 146.74, 144.44, 133.91, 132.60, 132.27, 119.75, 110.21, 108.29, 106.60, 100.99, 96.04, 67.94, 30.61 (one low field carbon not observed). ESI–MS calcd for $C_{17}H_{17}N_2O_3$ [M + H]$^+$ 297.1234; found 297.1235

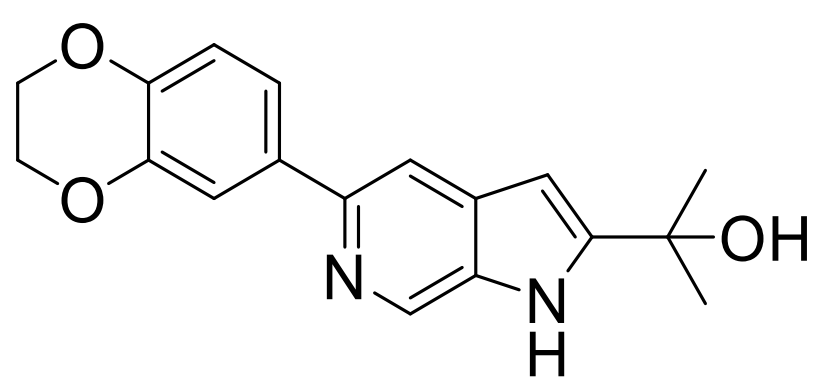

YKJ-184

2-(5-(2,3-Dihydrobenzo[*b*][1,4]dioxin-6-yl)-1*H*-pyrrolo[2,3-*c*]pyridin-2-yl)propan-2-ol

Yield: 96.2% ^{1}H NMR (400 MHz, DMSO) δ 11.54 (s, 1H), 8.66 (s, 1H), 7.92 (s, 1H), 7.59 – 7.48 (m, 2H), 6.92 (d, *J* = 8.3 Hz, 1H), 6.35 (d, *J* = 1.8 Hz, 1H), 5.47 (s, 1H), 4.28 (s, 4H), 1.55 (s, 6H). ^{13}C NMR (101 MHz, DMSO) δ 154.06, 143.98, 143.44, 143.16, 134.15, 132.25, 132.13, 119.14, 117.09, 114.84, 110.21, 96.18, 67.96, 64.21, 64.13, 30.60 (one low field carbon not observed). ESI–MS calcd for $C_{18}H_{19}N_2O_3$ [M + H]$^+$ 311.1390; found 311.1390

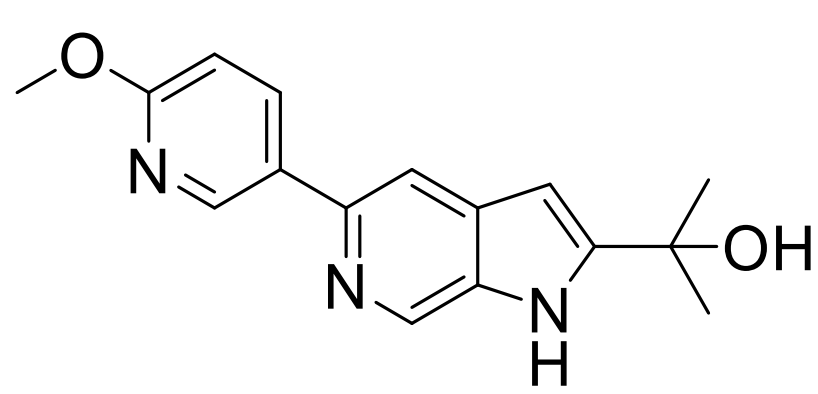

YKJ-185

2-(5-(6-Methoxypyridin-3-yl)-1*H*-pyrrolo[2,3-*c*]pyridin-2-yl)propan-2-ol

Yield: quantitative ^{1}H NMR (400 MHz, DMSO) δ 11.47 (s, 1H), 8.82 (d, *J* = 2.5 Hz, 1H), 8.71 (s, 1H), 8.34 (dd, *J* = 8.7, 2.5 Hz, 1H), 7.97 (s, 1H), 6.88 (d, *J* = 8.6 Hz, 1H), 6.34 (s, 1H), 5.43 (s, 1H), 3.90 (s, 3H), 1.55 (s, 6H). ^{13}C NMR (101 MHz, DMSO) δ 162.91, 153.23, 144.38, 142.68, 137.02, 133.56, 133.39, 132.48, 129.88, 110.11, 110.08, 95.87, 67.90, 53.21, 30.60. ESI–MS calcd for $C_{16}H_{18}N_3O_2$ [M + H]$^+$ 284.1394; found 284.1398

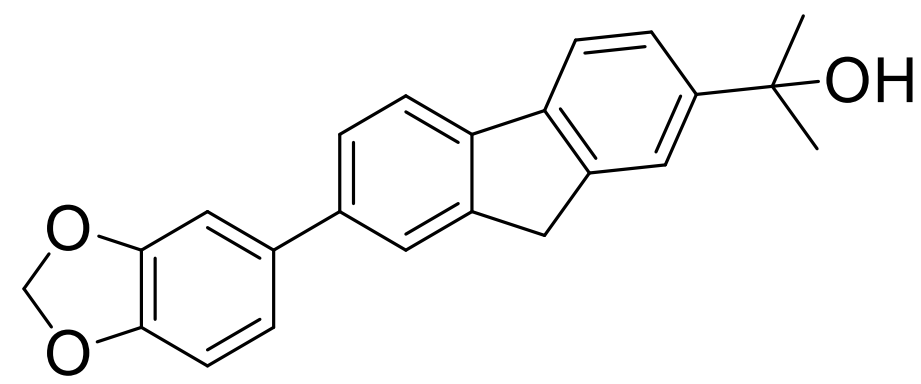

YKJ-186

2-(7-(Benzo[*d*][1,3]dioxol-5-yl)-9*H*-fluoren-2-yl)propan-2-ol

Yield: quantitative ^{1}H NMR (400 MHz, DMSO) δ 7.87 (d, *J* = 7.9 Hz, 1H), 7.83 – 7.78 (m, 2H), 7.70 (s, 1H), 7.60 (dd, *J* = 8.0, 1.7 Hz, 1H), 7.49 (dd, *J* = 7.9, 1.7 Hz, 1H), 7.30 (d, *J* = 1.8 Hz, 1H), 7.20 (dd, *J* = 8.1, 1.9 Hz, 1H), 7.01 (d, *J* = 8.1 Hz, 1H), 6.07 (s, 2H), 5.05 (s, 1H), 3.94 (s, 2H), 1.48 (s, 6H). ^{13}C NMR (101 MHz, DMSO) δ 149.52, 147.97, 146.66, 143.88, 142.86, 140.05, 138.61, 138.17, 134.86, 125.25, 123.37, 123.15, 121.36, 120.18, 120.04, 119.27, 108.66, 107.10, 101.10, 70.87, 36.57, 32.14. ESI–MS calcd for $C_{23}H_{19}O_2$ $[M - OH]^+$ 327.1380; found 327.1382

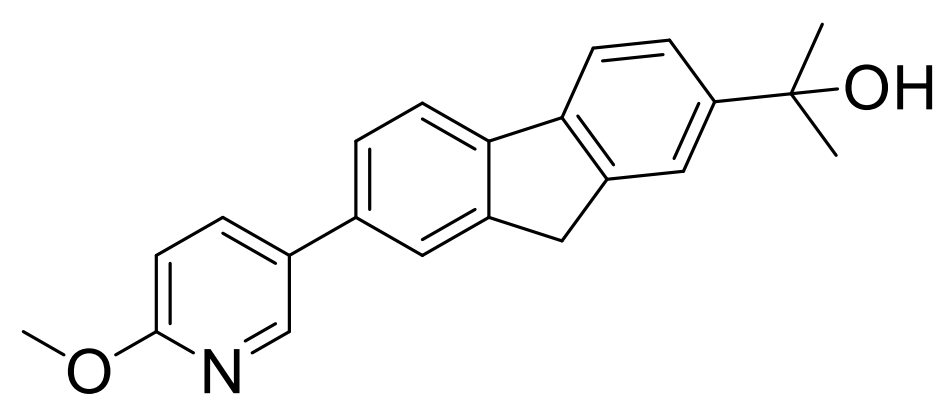

YKJ-187

2-(7-(6-Methoxypyridin-3-yl)-9*H*-fluoren-2-yl)propan-2-ol

Yield: 87.4% ^{1}H NMR (400 MHz, DMSO) δ 8.54 (d, *J* = 2.6 Hz, 1H), 8.07 (dd, *J* = 8.6, 2.6 Hz, 1H), 7.92 (d, *J* = 7.9 Hz, 1H), 7.88 – 7.80 (m, 2H), 7.71 (s, 1H), 7.66 (dd, *J* = 7.9, 1.7 Hz, 1H), 7.50 (dd, *J* = 8.0, 1.7 Hz, 1H), 6.92 (d, *J* = 8.6 Hz, 1H), 5.05 (s, 1H), 3.96 (s, 2H), 3.91 (s, 3H), 1.48 (s, 6H). ^{13}C NMR (101 MHz, DMSO) δ 162.90, 149.67, 144.56, 144.06, 142.86, 140.41, 138.50, 137.53, 135.13, 129.66, 125.09, 123.39, 122.99, 121.37, 120.26, 119.37, 110.55, 70.86, 53.26, 36.58, 32.13. ESI–MS calcd for $C_{22}H_{22}NO_2$ $[M + H]^+$ 332.1645; found 332.1649

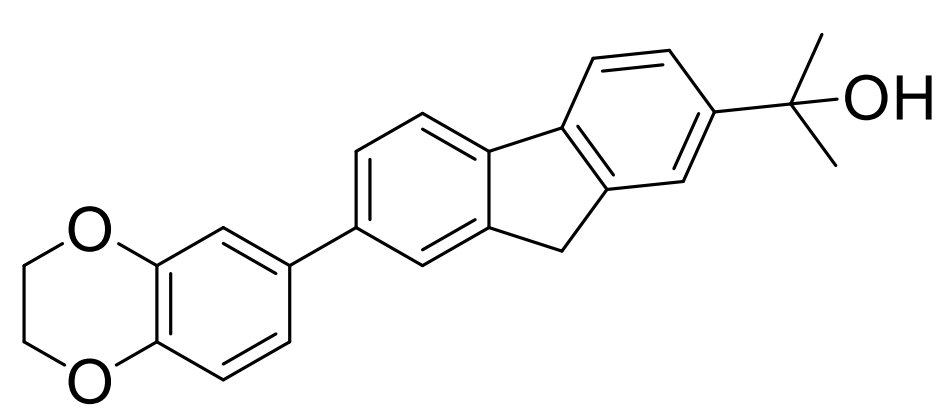

YKJ-188

2-(7-(2,3-Dihydrobenzo[*b*][1,4]dioxin-6-yl)-9*H*-fluoren-2-yl)propan-2-ol

Yield: 93.5% ^{1}H NMR (400 MHz, DMSO) δ 7.86 (d, *J* = 7.9 Hz, 1H), 7.83 – 7.76 (m, 2H), 7.70 (s, 1H), 7.59 (d, *J* = 8.0 Hz, 1H), 7.49 (d, *J* = 8.0 Hz, 1H), 7.23 – 7.18 (m, 2H), 6.94 (d, *J* = 8.1 Hz, 1H), 5.05 (s, 1H), 4.28 (s, 4H), 3.94 (s, 2H), 1.48 (s, 6H). ^{13}C NMR (101 MHz, DMSO) δ 149.48, 143.89, 143.66, 142.94, 142.85, 139.98, 138.63, 137.87, 133.85, 125.03, 123.35, 122.92, 121.34, 120.04, 119.49, 119.24, 117.46, 115.04, 70.86, 64.12, 36.56, 32.13. ESI–MS calcd for $C_{24}H_{21}O_2$ $[M - OH]^+$ 341.1536; found 341.1539

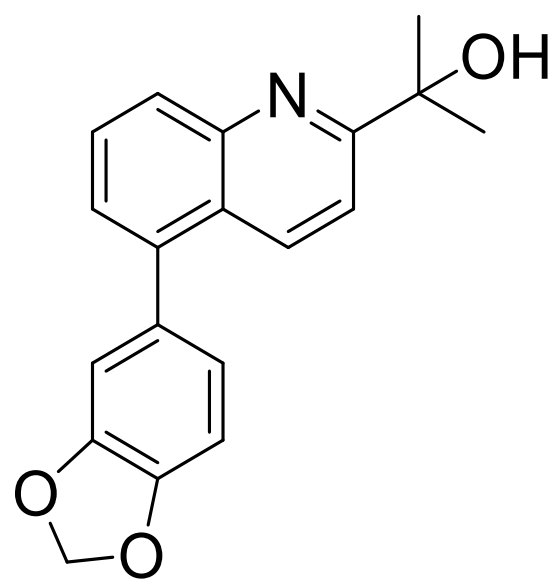

YKJ-189

2-(5-(Benzo[*d*][1,3]dioxol-5-yl)quinolin-2-yl)propan-2-ol

Yield: quantitative ^{1}H NMR (400 MHz, DMSO) δ 8.39 (d, *J* = 8.6 Hz, 1H), 7.92 (dd, *J* = 8.1, 1.5 Hz, 1H), 7.86 (d, *J* = 8.6 Hz, 1H), 7.76 (dd, *J* = 7.2, 1.5 Hz, 1H), 7.63 – 7.60 (m, 1H), 7.37 (d, *J* = 1.7 Hz, 1H), 7.21 (dd, *J* = 8.1, 1.7 Hz, 1H), 7.02 (d, *J* = 8.1 Hz, 1H), 6.08 (s, 2H), 5.42 (s, 1H), 1.48 (s, 6H). ^{13}C NMR (101 MHz, DMSO) δ 167.42, 146.68, 146.44, 143.16, 138.51, 137.08, 132.87, 129.76, 127.17, 127.08, 126.01, 124.01, 117.64, 111.25, 107.75, 100.95, 72.89, 30.37. ESI–MS calcd for $C_{19}H_{18}NO_3$ $[M + H]^+$ 308.1281; found 308.1286

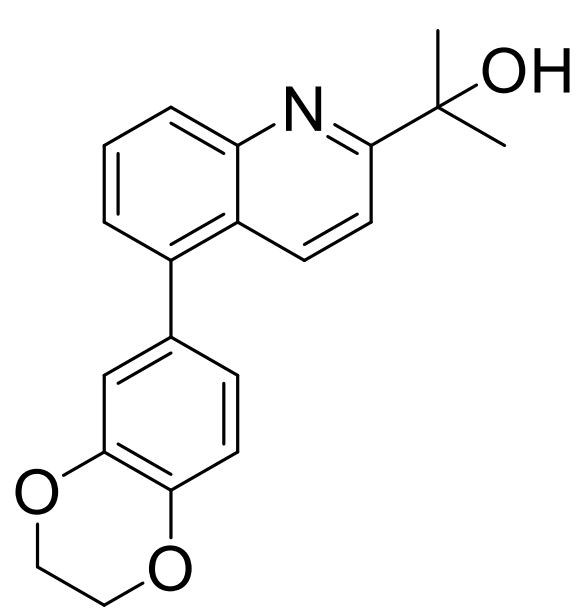

YKJ-190

2-(5-(2,3-Dihydrobenzo[*b*][1,4]dioxin-6-yl)quinolin-2-yl)propan-2-ol

Yield: 96.9% ^{1}H NMR (400 MHz, DMSO) δ 8.38 (d, *J* = 8.6 Hz, 1H), 7.91 (dd, *J* = 8.2, 1.5 Hz, 1H), 7.86 (d, *J* = 8.6 Hz, 1H), 7.74 (dd, *J* = 7.3, 1.5 Hz, 1H), 7.60 (dd, *J* = 8.1, 7.2 Hz, 1H), 7.33 (d, *J* = 2.1 Hz, 1H), 7.24 (dd, *J* = 8.4, 2.1 Hz, 1H), 6.94 (d, *J* = 8.4 Hz, 1H), 5.42 (s, 1H), 4.30 (s, 4H), 1.48 (s, 6H). ^{13}C NMR (101 MHz, DMSO) δ 167.36, 143.13, 142.75, 142.70, 138.21, 137.08, 132.00, 129.64, 127.09, 127.04, 126.01, 123.60, 119.35, 117.57, 116.19, 72.88, 64.21, 64.07, 30.40. ESI–MS calcd for $C_{20}H_{20}NO_3$ $[M + H]^+$ 322.1438; found 322.1442

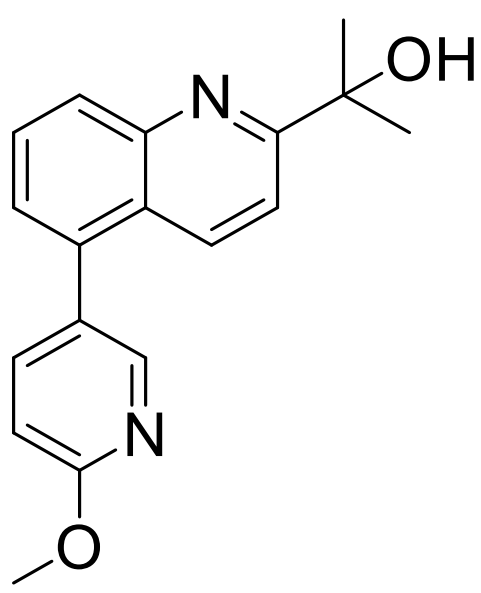

YKJ-191

2-(5-(6-Methoxypyridin-3-yl)quinolin-2-yl)propan-2-ol

Yield: quantitative ^{1}H NMR (400 MHz, DMSO) δ 8.52 (d, *J* = 2.4 Hz, 1H), 8.41 (d, *J* = 8.6 Hz, 1H), 8.14 (dd, *J* = 8.6, 2.5 Hz, 1H), 7.96 (dd, *J* = 8.2, 1.5 Hz, 1H), 7.90 (d, *J* = 8.6 Hz, 1H), 7.81 (dd, *J* = 7.2, 1.4 Hz, 1H), 7.64 (t, *J* = 7.6 Hz, 1H), 6.94 (d, *J* = 8.6 Hz, 1H), 5.39 (s, 1H), 3.93 (s, 3H), 1.47 (s, 6H). ^{13}C NMR (101 MHz, DMSO) δ 167.82, 162.67, 147.48, 143.27, 141.47, 137.00, 135.58, 129.48, 128.07, 127.58, 127.04, 126.05, 117.86, 109.13, 72.93, 53.20, 30.36. ESI–MS calcd for $C_{18}H_{19}N_2O_2$ $[M + H]^+$ 295.1441; found 295.1448

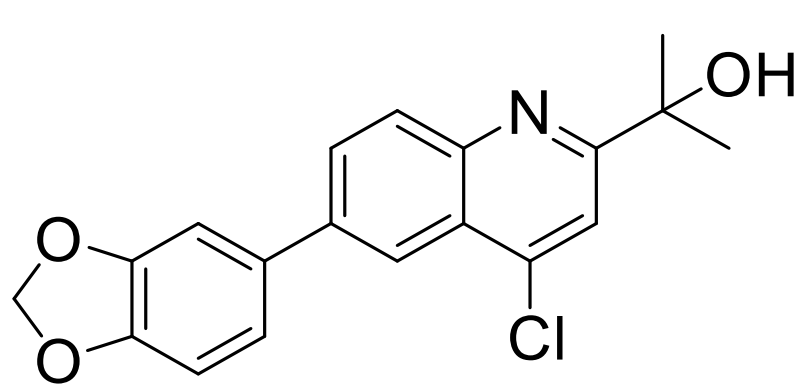

YKJ-192

2-(6-(Benzo[*d*][1,3]dioxol-5-yl)-4-chloroquinolin-2-yl)propan-2-ol

Yield: 45.9% ^{1}H NMR (400 MHz, DMSO) δ 8.25 (d, *J* = 2.0 Hz, 1H), 8.14 – 8.04 (m, 2H), 8.01 (s, 1H), 7.43 (d, *J* = 1.9 Hz, 1H), 7.32 (dd, *J* = 8.1, 1.9 Hz, 1H), 7.07 (d, *J* = 8.1 Hz, 1H), 6.11 (s, 2H), 5.56 (s, 1H), 1.55 (s, 6H). ^{13}C NMR (101 MHz, DMSO) δ 168.71, 148.24, 147.50, 146.57,

141.55, 138.82, 133.21, 129.86, 129.82, 124.42, 121.12, 119.97, 118.31, 108.90, 107.51, 101.38, 72.71, 30.26. ESI–MS calcd for $C_{19}H_{17}ClNO_3$ $[M + H]^+$ 342.0891; found 342.0890

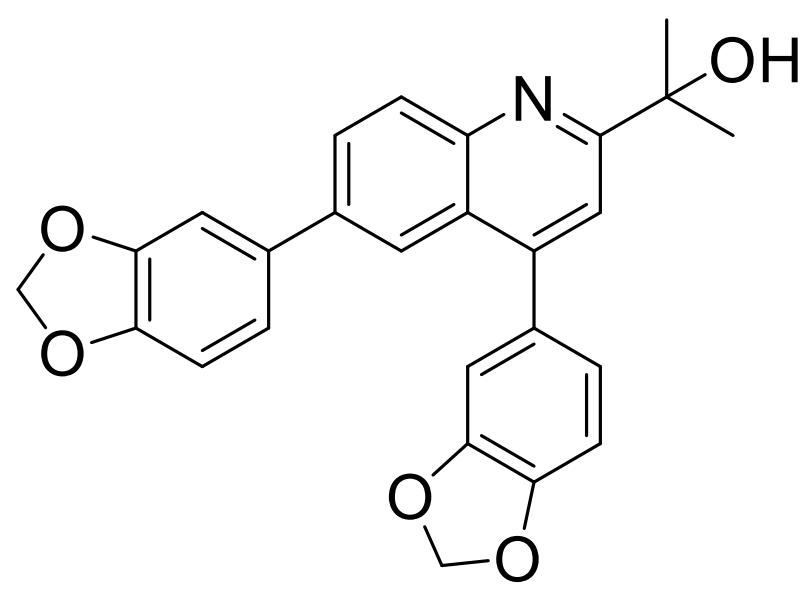

YKJ-193

2-(4,6-Bis(benzo[*d*][1,3]dioxol-5-yl)quinolin-2-yl)propan-2-ol

Yield: 53.1% ^{1}H NMR (400 MHz, DMSO) δ 8.10 – 8.03 (m, 1H), 8.03 – 7.95 (m, 2H), 7.75 (s, 1H), 7.23 (dd, *J* = 11.4, 1.8 Hz, 2H), 7.17 – 7.11 (m, 2H), 7.08 (dd, *J* = 8.0, 1.7 Hz, 1H), 7.02 (d, *J* = 8.1 Hz, 1H), 6.15 (s, 2H), 6.07 (s, 2H), 5.47 (s, 1H), 1.57 (s, 6H). ^{13}C NMR (101 MHz, DMSO) δ 167.76, 148.10, 147.72, 147.61, 147.14, 146.18, 137.70, 134.07, 131.57, 129.78, 128.58, 124.95, 123.36, 122.10, 120.79, 118.24, 109.76, 108.87, 108.68, 107.39, 101.45, 101.27, 72.74, 30.48. ESI–MS calcd for $C_{26}H_{22}NO_5$ $[M + H]^+$ 428.1492; found 428.1498

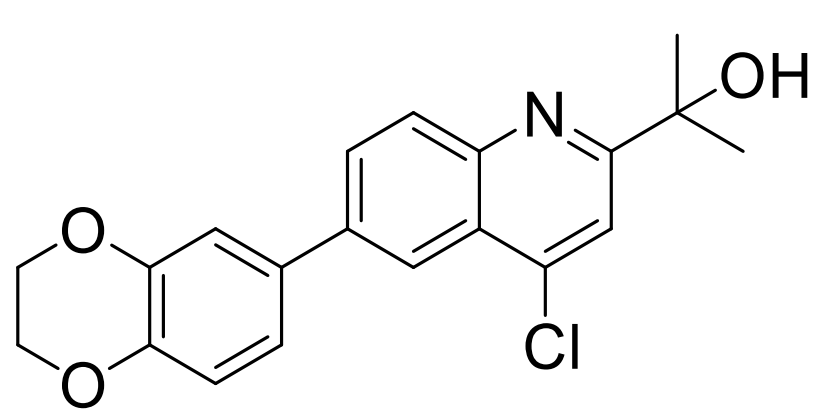

YKJ-194

2-(4-Chloro-6-(2,3-dihydrobenzo[*b*][1,4]dioxin-6-yl)quinolin-2-yl)propan-2-ol

Yield: 75.2% ^{1}H NMR (400 MHz, DMSO) δ 8.24 (d, *J* = 2.0 Hz, 1H), 8.12 – 8.04 (m, 2H), 8.01 (s, 1H), 7.34 – 7.29 (m, 2H), 7.01 (d, *J* = 8.1 Hz, 1H), 5.55 (s, 1H), 4.31 (s, 4H), 1.55 (s, 6H). ^{13}C NMR (101 MHz, DMSO) δ 168.68, 146.56, 143.90, 143.79, 141.53, 138.52, 132.20, 129.90, 129.63, 124.44, 120.11, 119.70, 118.29, 117.81, 115.64, 72.71, 64.25, 64.15, 30.26. ESI–MS calcd for $C_{20}H_{19}ClNO_3$ $[M + H]^+$ 356.1048; found 356.1047

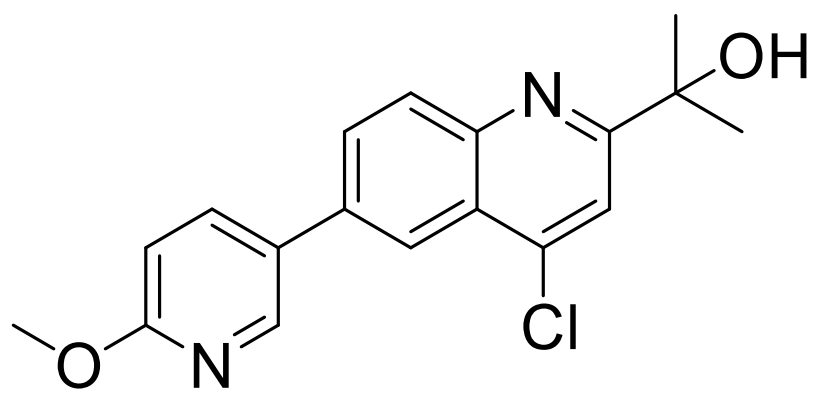

YKJ-195

2-(4-Chloro-6-(6-methoxypyridin-3-yl)quinolin-2-yl)propan-2-ol

Yield: 69.2% ^{1}H NMR (400 MHz, DMSO) δ 8.66 (dd, *J* = 2.6, 0.8 Hz, 1H), 8.31 (d, *J* = 2.0 Hz, 1H), 8.21 (dd, *J* = 8.7, 2.6 Hz, 1H), 8.17 (dd, *J* = 8.8, 2.1 Hz, 1H), 8.11 (d, *J* = 8.8 Hz, 1H), 8.03 (s, 1H), 6.98 (dd, *J* = 8.6, 0.7 Hz, 1H), 5.57 (s, 1H), 3.93 (s, 3H), 1.55 (s, 6H). ^{13}C NMR (101 MHz, DMSO) δ 168.96, 163.50, 146.70, 145.37, 141.59, 138.03, 136.01, 130.12, 129.47, 128.24, 124.52, 120.06, 118.42, 110.88, 72.73, 53.41, 30.25. ESI–MS calcd for $C_{18}H_{18}ClN_2O_2$ $[M + H]^+$ 329.1051; found 329.1052

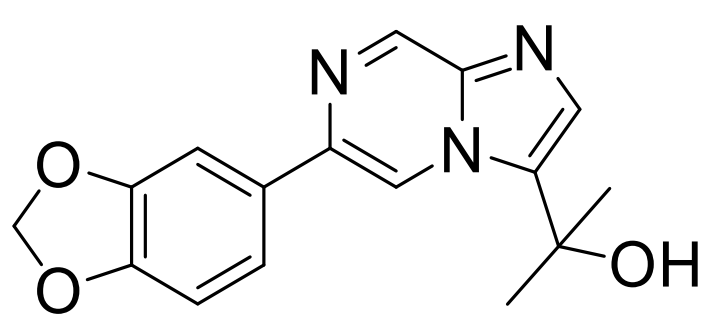

YKJ-196

2-(6-(Benzo[*d*][1,3]dioxol-5-yl)imidazo[1,2-*a*]pyrazin-3-yl)propan-2-ol

Yield: 87.4% ^{1}H NMR (400 MHz, DMSO) δ 9.11 (d, *J* = 1.5 Hz, 1H), 8.97 (d, *J* = 1.5 Hz, 1H), 7.69 (s, 1H), 7.56 – 7.48 (m, 2H), 7.06 (d, *J* = 8.1 Hz, 1H), 6.10 (s, 2H), 5.68 (s, 1H), 1.67 (s, 6H). ^{13}C NMR (101 MHz, DMSO) δ 148.03, 147.65, 142.55, 139.95, 137.23, 133.12, 132.14, 130.77, 119.94, 114.99, 108.70, 106.33, 101.38, 67.19, 29.04. ESI–MS calcd for $C_{16}H_{16}N_3O_3$ $[M + H]^+$ 298.1186; found 298.1184

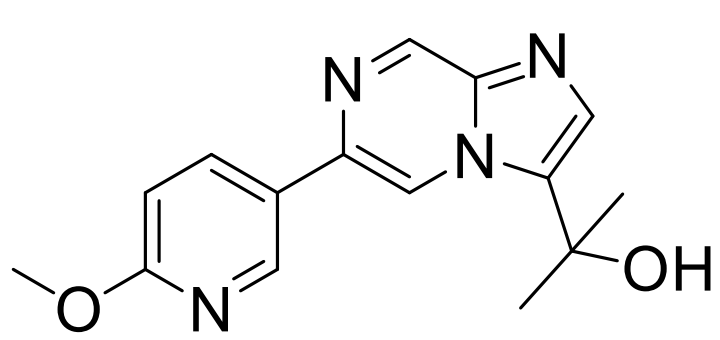

YKJ-197

2-(6-(6-Methoxypyridin-3-yl)imidazo[1,2-*a*]pyrazin-3-yl)propan-2-ol

Yield: quantitative ^{1}H NMR (400 MHz, DMSO) δ 9.15 (d, *J* = 1.5 Hz, 1H), 9.04 (d, *J* = 1.5 Hz, 1H), 8.79 (d, *J* = 2.4 Hz, 1H), 8.27 (dd, *J* = 8.7, 2.6 Hz, 1H), 7.71 (s, 1H), 6.97 (d, *J* = 8.6 Hz, 1H), 5.68 (s, 1H), 3.92 (s, 3H), 1.68 (s, 6H). ^{13}C NMR (101 MHz, DMSO) δ 163.71, 144.70, 142.99, 139.97, 137.00, 135.30, 133.31, 132.24, 126.07, 115.19, 110.70, 67.21, 53.45, 29.03. ESI–MS calcd for $C_{15}H_{17}N_4O_2$ $[M + H]^+$ 285.1346; found 285.1345

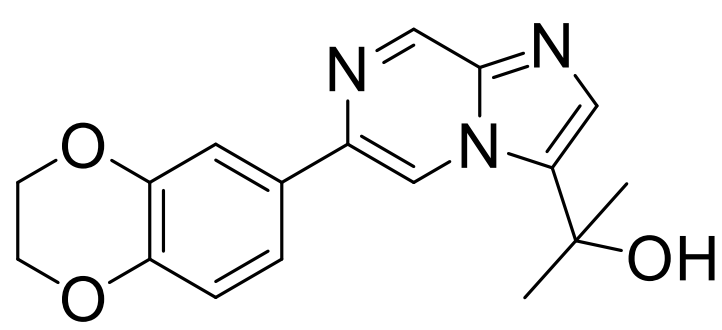

YKJ-198

2-(6-(2,3-Dihydrobenzo[*b*][1,4]dioxin-6-yl)imidazo[1,2-*a*]pyrazin-3-yl)propan-2-ol

Yield: 84.7% ^{1}H NMR (400 MHz, DMSO) δ 9.10 (d, *J* = 1.4 Hz, 1H), 8.97 (d, *J* = 1.5 Hz, 1H), 7.67 (s, 1H), 7.51 – 7.42 (m, 2H), 6.99 (d, *J* = 8.4 Hz, 1H), 5.70 (s, 1H), 4.30 (s, 4H), 1.67 (s, 6H). ^{13}C NMR (101 MHz, DMSO) δ 143.93, 143.73, 142.61, 139.98, 137.04, 133.07, 132.11, 129.83, 118.94, 117.59, 114.85, 114.66, 67.23, 64.29, 64.19, 29.06. ESI–MS calcd for $C_{17}H_{18}N_3O_3$ $[M + H]^+$ 312.1343; found 312.1345

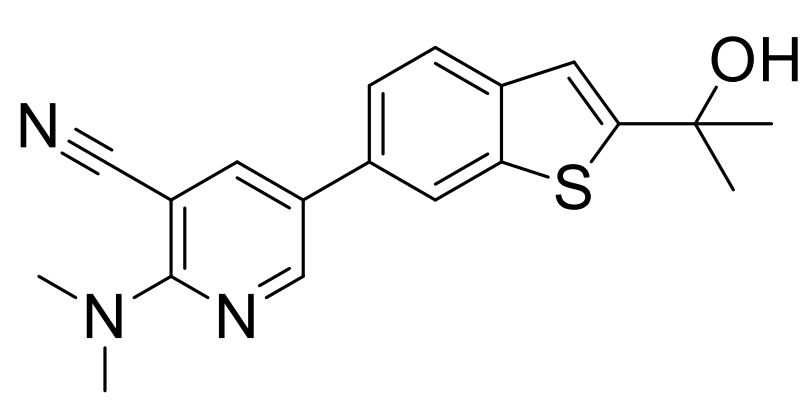

YKJ-199

2-(Dimethylamino)-5-(2-(2-hydroxypropan-2-yl)benzo[*b*]thiophen-6-yl)nicotinonitrile

Yield: 90.6% ^{1}H NMR (400 MHz, DMSO) δ 8.77 (d, *J* = 2.5 Hz, 1H), 8.37 (d, *J* = 2.6 Hz, 1H), 8.24 (d, *J* = 1.7 Hz, 1H), 7.78 (d, *J* = 8.3 Hz, 1H), 7.65 (dd, *J* = 8.3, 1.7 Hz, 1H), 7.21 (s, 1H), 5.65 (s, 1H), 3.26 (s, 6H), 1.57 (s, 6H). ^{13}C NMR (101 MHz, DMSO) δ 157.94, 157.91, 150.04, 142.06, 139.45, 139.04, 131.02, 124.45, 123.61, 122.21, 119.32, 118.86, 117.41, 90.38, 69.98, 40.05, 32.04. ESI–MS calcd for $C_{19}H_{20}N_3OS$ $[M + H]^+$ 338.1322; found 338.1323

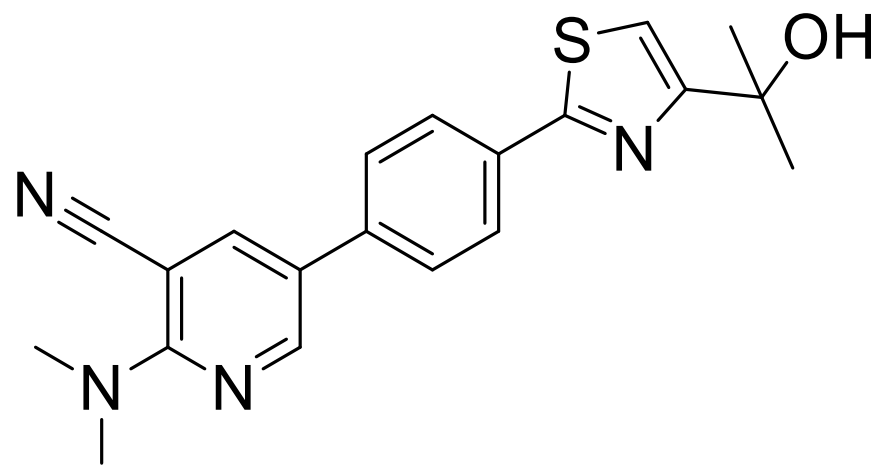

YKJ-200

2-(Dimethylamino)-5-(4-(4-(2-hydroxypropan-2-yl)thiazol-2-yl)phenyl)nicotinonitrile

Yield: quantitative ^{1}H NMR (400 MHz, DMSO) δ 8.77 (d, *J* = 2.5 Hz, 1H), 8.38 (d, *J* = 2.5 Hz, 1H), 7.99 – 7.93 (m, 2H), 7.85 – 7.78 (m, 2H), 7.42 (s, 1H), 5.24 (s, 1H), 3.27 (s, 6H), 1.51 (s, 6H). ^{13}C NMR (101 MHz, DMSO) δ 166.43, 165.81, 158.06, 150.06, 142.14, 136.92, 132.21, 126.65, 126.32, 123.26, 118.85, 112.81, 90.22, 70.50, 40.05, 30.61. ESI–MS calcd for $C_{20}H_{21}N_4OS$ [M + H]$^+$ 365.1431; found 365.1431

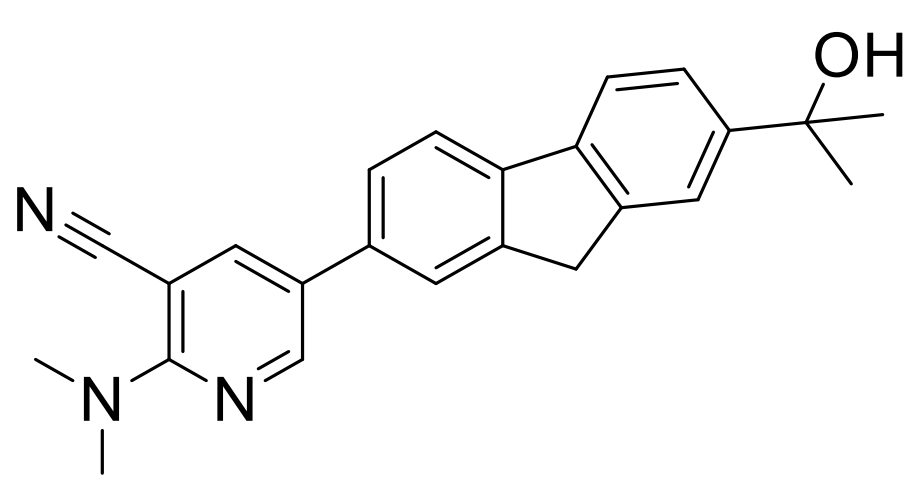

YKJ-201

2-(Dimethylamino)-5-(7-(2-hydroxypropan-2-yl)-9*H*-fluoren-2-yl)nicotinonitrile

Yield: 57.8% ^{1}H NMR (400 MHz, DMSO) δ 8.76 (d, *J* = 2.5 Hz, 1H), 8.35 (d, *J* = 2.6 Hz, 1H), 7.94 – 7.87 (m, 2H), 7.83 (d, *J* = 8.0 Hz, 1H), 7.73 – 7.66 (m, 2H), 7.50 (dd, *J* = 8.0, 1.7 Hz, 1H), 5.06 (s, 1H), 3.94 (s, 2H), 3.26 (s, 6H), 1.48 (s, 6H). ^{13}C NMR (101 MHz, DMSO) δ 157.93, 149.96, 149.69, 144.07, 142.88, 141.99, 140.45, 138.51, 133.65, 124.79, 124.60, 123.42, 122.50, 121.40, 120.30, 119.41, 118.89, 90.37, 70.89, 40.07, 36.59, 32.15. ESI–MS calcd for $C_{24}H_{24}N_3O$ [M + H]$^+$ 370.1914; found 370.1916

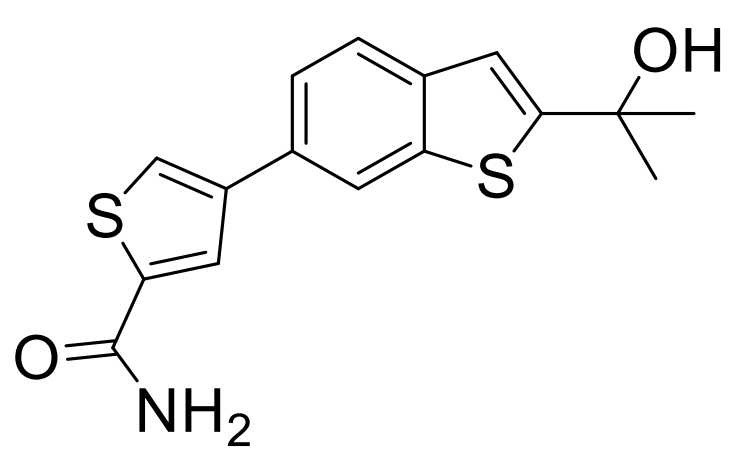

YKJ-202

4-(2-(2-Hydroxypropan-2-yl)benzo[*b*]thiophen-6-yl)thiophene-2-carboxamide

Yield: 74.5% ^{1}H NMR (400 MHz, DMSO) δ 8.30 (d, *J* = 1.5 Hz, 1H), 8.19 (d, *J* = 1.8 Hz, 1H), 8.11 (d, *J* = 1.5 Hz, 1H), 7.99 (s, 1H), 7.79 (d, *J* = 8.3 Hz, 1H), 7.66 (dd, *J* = 8.3, 1.7 Hz, 1H), 7.48 (s, 1H), 7.21 (s, 1H), 5.66 (s, 1H), 1.58 (s, 6H). ^{13}C NMR (101 MHz, DMSO) δ 162.82, 157.95, 141.73, 140.87, 139.29, 139.11, 130.39, 127.54, 125.32, 123.59, 122.46, 119.22, 117.55, 69.99, 32.03. ESI–MS calcd for $C_{16}H_{16}NO_2S_2$ $[M + H]^+$ 318.0617; found 318.0619

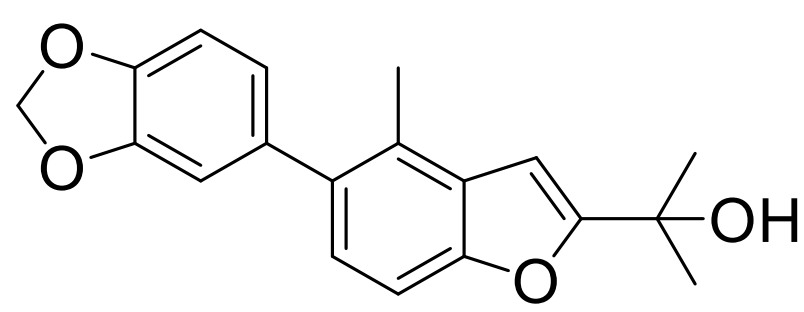

YKJ-203

2-(5-(Benzo[*d*][1,3]dioxol-5-yl)-4-methylbenzofuran-2-yl)propan-2-ol

Yield: quantitative ^{1}H NMR (400 MHz, DMSO) δ 7.36 (d, *J* = 8.4 Hz, 1H), 7.06 (d, *J* = 8.4 Hz, 1H), 6.97 (d, *J* = 7.9 Hz, 1H), 6.89 (d, *J* = 1.7 Hz, 1H), 6.77 (dd, *J* = 7.9, 1.7 Hz, 1H), 6.74 (d, *J* = 1.0 Hz, 1H), 6.06 (s, 2H), 5.41 (s, 1H), 2.35 (s, 3H), 1.53 (s, 6H). ^{13}C NMR (101 MHz, DMSO) δ 164.82, 152.72, 147.02, 146.01, 135.23, 135.17, 128.63, 127.39, 125.61, 122.77, 109.97, 108.25, 108.02, 100.97, 99.28, 67.63, 29.05, 16.56. ESI–MS calcd for $C_{19}H_{17}O_3$ $[M + H]^+$ 293.1172; found 293.1175

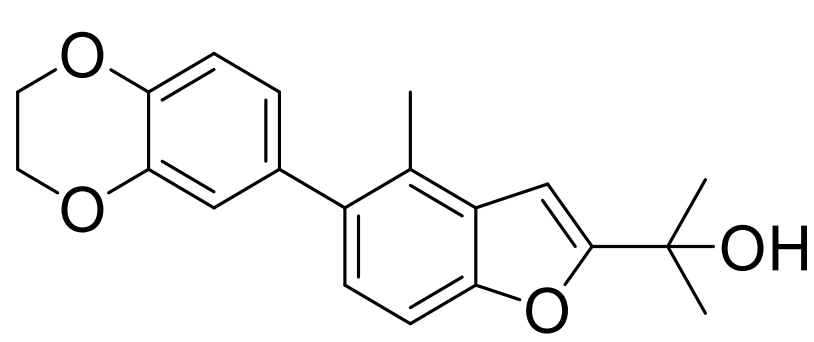

YKJ-204

2-(5-(2,3-Dihydrobenzo[*b*][1,4]dioxin-6-yl)-4-methylbenzofuran-2-yl)propan-2-ol

Yield: 83.4% ^{1}H NMR (500 MHz, DMSO) δ 7.35 (d, *J* = 8.4 Hz, 1H), 7.05 (d, *J* = 8.4 Hz, 1H), 6.91 (d, *J* = 8.1 Hz, 1H), 6.81 – 6.75 (m, 2H), 6.73 (d, *J* = 1.0 Hz, 1H), 5.41 (s, 1H), 4.28 (s, 4H), 2.35 (s, 3H), 1.53 (s, 6H). ^{13}C NMR (126 MHz, DMSO) δ 164.79, 152.69, 142.91, 142.29, 134.95, 134.52, 128.64, 127.29, 125.57, 122.42, 117.99, 116.70, 108.28, 99.30, 67.64, 64.13, 64.11, 29.06, 16.60. ESI–MS calcd for $C_{20}H_{19}O_3$ $[M - OH]^+$ 307.1329; found 307.1330

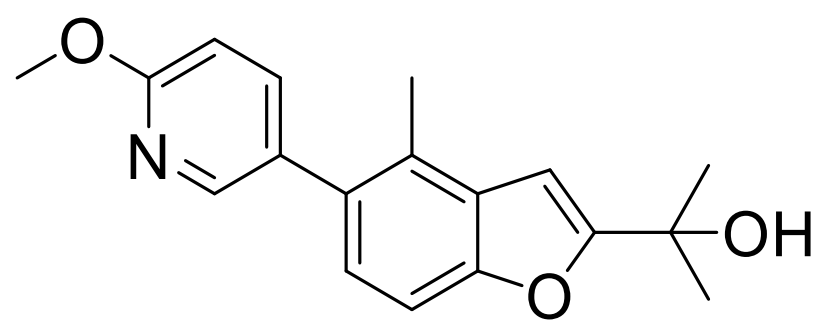

YKJ-205

2-(5-(6-Methoxypyridin-3-yl)-4-methylbenzofuran-2-yl)propan-2-ol

Yield: quantitative ^{1}H NMR (500 MHz, DMSO) δ 8.14 (d, *J* = 2.5 Hz, 1H), 7.71 (dd, *J* = 8.4, 2.6 Hz, 1H), 7.42 (d, *J* = 8.4 Hz, 1H), 7.10 (d, *J* = 8.4 Hz, 1H), 6.89 (d, *J* = 8.5 Hz, 1H), 6.77 (s, 1H), 5.42 (s, 1H), 3.90 (s, 3H), 2.36 (s, 3H), 1.54 (s, 6H). ^{13}C NMR (126 MHz, DMSO) δ 164.97, 162.39, 153.03, 146.67, 140.24, 131.70, 130.25, 128.83, 127.88, 125.72, 109.83, 108.56, 99.27, 67.63, 53.18, 29.05, 16.51. ESI–MS calcd for $C_{18}H_{20}NO_3$ $[M + H]^+$ 298.1438; found 298.1438

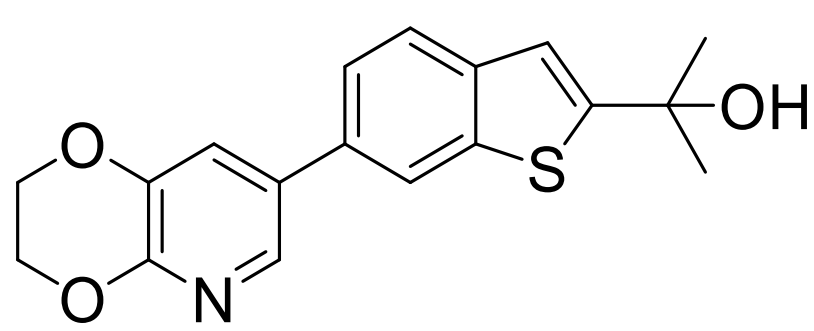

YKJ-206

2-(6-(2,3-Dihydro-[1,4]dioxino[2,3-*b*]pyridin-7-yl)benzo[*b*]thiophen-2-yl)propan-2-ol

Yield: quantitative ^{1}H NMR (500 MHz, DMSO) δ 8.20 (s, 1H), 8.13 (d, *J* = 2.1 Hz, 1H), 7.79 (d, *J* = 8.3 Hz, 1H), 7.70 – 7.59 (m, 2H), 7.22 (s, 1H), 5.66 (s, 1H), 4.49 – 4.41 (m, 2H), 4.34 – 4.26 (m, 2H), 1.57 (s, 6H). ^{13}C NMR (126 MHz, DMSO) δ 158.09, 150.26, 139.42, 139.24, 139.22, 137.01, 132.11, 131.22, 123.61, 122.92, 122.61, 120.03, 117.42, 70.03, 64.81, 63.84, 32.08. ESI–MS calcd for $C_{18}H_{18}NO_3S$ $[M + H]^+$ 328.1002; found 328.1004

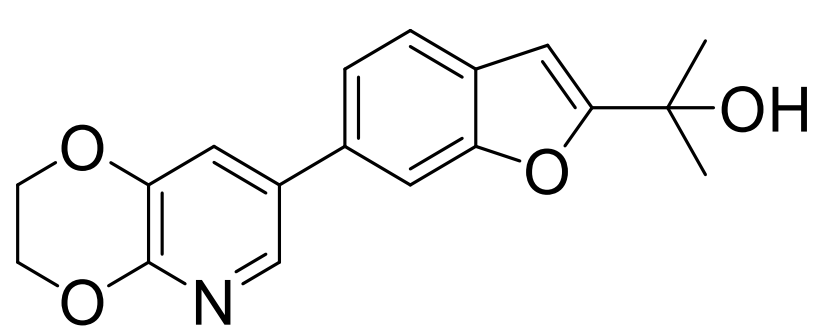

YKJ-207

2-(6-(2,3-Dihydro-[1,4]dioxino[2,3-*b*]pyridin-7-yl)benzofuran-2-yl)propan-2-ol

Yield: 64.7% ^{1}H NMR (500 MHz, DMSO) δ 8.12 (d, *J* = 2.3 Hz, 1H), 7.83 (s, 1H), 7.69 – 7.60 (m, 2H), 7.51 (dd, *J* = 8.1, 1.6 Hz, 1H), 6.69 (s, 1H), 5.44 (s, 1H), 4.47 – 4.40 (m, 2H), 4.33 – 4.25

(m, 2H), 1.53 (s, 6H). ^{13}C NMR (126 MHz, DMSO) δ 165.59, 154.68, 150.20, 139.14, 137.03, 132.55, 131.40, 127.71, 122.69, 121.50, 121.23, 108.91, 100.00, 67.60, 64.76, 63.79, 29.01. ESI–MS calcd for $C_{18}H_{18}NO_4$ [M + H]$^+$ 312.1230; found 312.1236

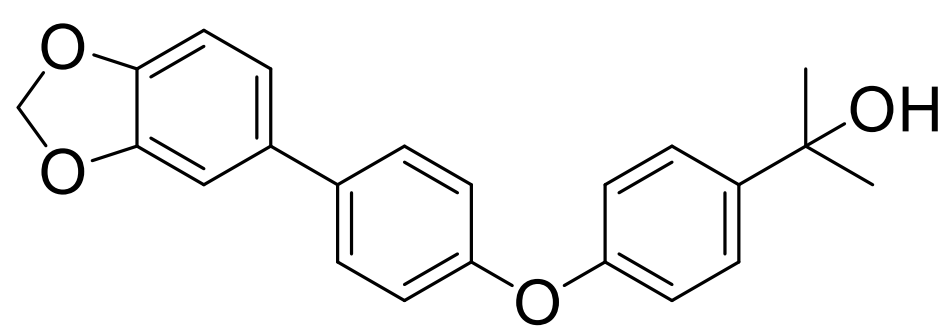

YKJ-208

2-(4-(4-(Benzo[*d*][1,3]dioxol-5-yl)phenoxy)phenyl)propan-2-ol

Yield: 83.6% ^{1}H NMR (400 MHz, DMSO) δ 7.62 – 7.57 (m, 2H), 7.51 – 7.46 (m, 2H), 7.21 (d, *J* = 1.8 Hz, 1H), 7.10 (dd, *J* = 8.1, 1.9 Hz, 1H), 7.03 – 6.95 (m, 5H), 6.05 (s, 2H), 5.01 (s, 1H), 1.43 (s, 6H). ^{13}C NMR (101 MHz, DMSO) δ 156.37, 154.51, 147.95, 146.59, 145.98, 134.89, 133.86, 127.97, 126.25, 119.93, 118.49, 118.19, 108.64, 106.93, 101.09, 70.42, 31.99. ESI–MS calcd for $C_{22}H_{19}O_3$ [M - OH]$^+$ 331.1329; found 331.1329

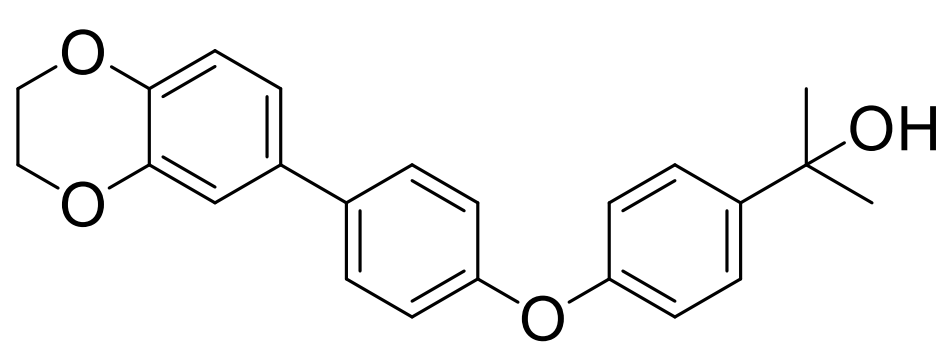

YKJ-209

2-(4-(4-(2,3-Dihydrobenzo[*b*][1,4]dioxin-6-yl)phenoxy)phenyl)propan-2-ol

Yield: 80.1% ^{1}H NMR (400 MHz, DMSO) δ 7.61 – 7.55 (m, 2H), 7.51 – 7.46 (m, 2H), 7.13 – 7.07 (m, 2H), 7.02 – 6.96 (m, 4H), 6.91 (d, *J* = 8.3 Hz, 1H), 5.00 (s, 1H), 4.27 (s, 4H), 1.43 (s, 6H). ^{13}C NMR (101 MHz, DMSO) δ 156.32, 154.49, 145.96, 143.63, 142.85, 134.57, 132.87, 127.75, 126.23, 119.25, 118.47, 118.19, 117.44, 114.84, 70.40, 64.10 (2 C's), 31.98. ESI–MS calcd for $C_{23}H_{21}O_3$ [M - OH]$^+$ 345.1485; found 345.1481

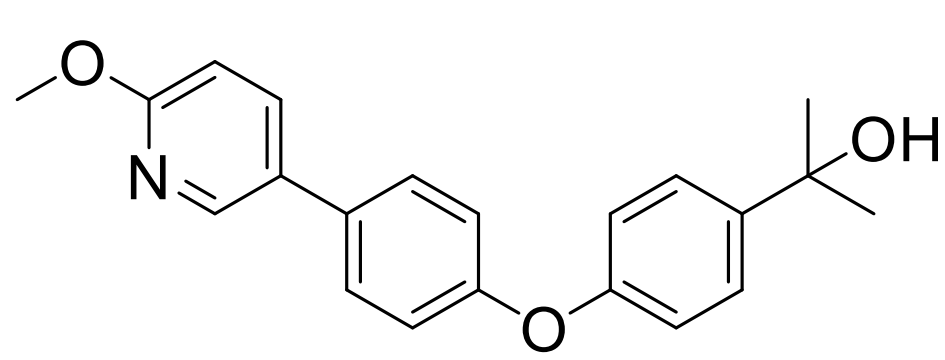

YKJ-210

2-(4-(4-(6-Methoxypyridin-3-yl)phenoxy)phenyl)propan-2-ol

Yield: 97.2% ^{1}H NMR (400 MHz, DMSO) δ 8.45 (d, *J* = 2.6 Hz, 1H), 7.98 (dd, *J* = 8.6, 2.6 Hz, 1H), 7.67 – 7.64 (m, 2H), 7.51 – 7.47 (m, 2H), 7.08 – 7.04 (m, 2H), 7.01 – 6.97 (m, 2H), 6.90 (d, *J* = 8.7 Hz, 1H), 5.01 (s, 1H), 3.89 (s, 3H), 1.43 (s, 6H). ^{13}C NMR (101 MHz, DMSO) δ 162.83, 156.69, 154.42, 146.04, 144.37, 137.34, 131.90, 128.74, 127.92, 126.25, 118.68, 118.20, 110.53, 70.40, 53.22, 31.98. ESI–MS calcd for $C_{21}H_{22}NO_3$ $[M + H]^+$ 336.1594; found 336.1592

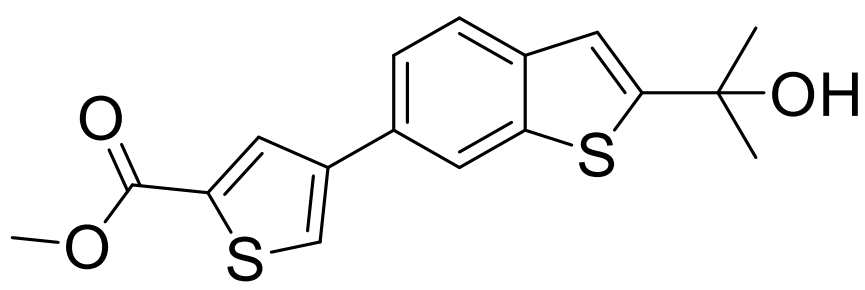

YKJ-211

Methyl 4-(2-(2-hydroxypropan-2-yl)benzo[*b*]thiophen-6-yl)thiophene-2-carboxylate

Yield: 83.5% ^{1}H NMR (400 MHz, DMSO) δ 8.35 (s, 1H), 8.32 – 8.29 (m, 2H), 7.80 – 7.72 (m, 2H), 7.21 (s, 1H), 5.65 (s, 1H), 3.86 (s, 3H), 1.58 (s, 6H). ^{13}C NMR (101 MHz, DMSO) δ 161.84, 158.19, 142.19, 139.30, 133.43, 132.07, 129.57, 127.92, 123.51, 122.59, 119.76, 117.47, 69.97, 52.30, 32.01 (one low field carbon not observed). ESI–MS calcd for $C_{17}H_{15}O_2S_2$ $[M + H]^+$ 315.0508; found 315.0510

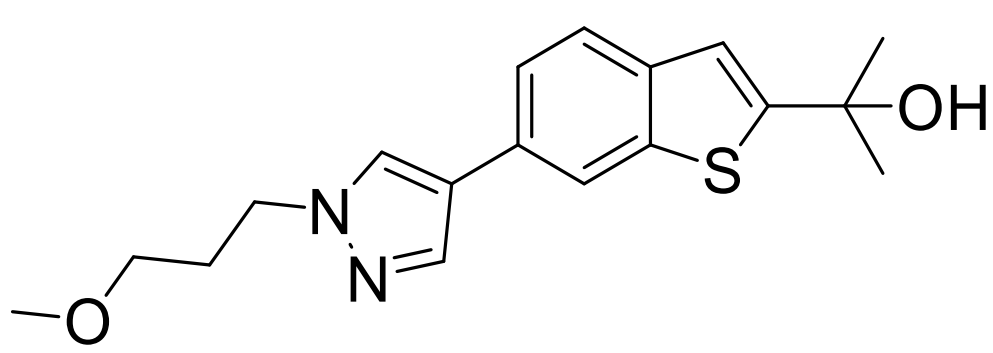

YKJ-212

2-(6-(1-(3-Methoxypropyl)-1*H*-pyrazol-4-yl)benzo[*b*]thiophen-2-yl)propan-2-ol

Yield: 79.4% 1H NMR (400 MHz, DMSO) δ 8.21 (s, 1H), 8.09 (s, 1H), 7.94 (s, 1H), 7.69 (d, *J* = 8.3 Hz, 1H), 7.54 (dd, *J* = 8.2, 1.6 Hz, 1H), 7.15 (s, 1H), 5.60 (s, 1H), 4.16 (t, *J* = 7.0 Hz, 2H), 3.32 (t, *J* = 7.0 Hz, 2H), 3.24 (s, 3H), 2.04 (pentet, *J* = 6.6 Hz, 2H), 1.56 (s, 6H). ^{13}C NMR (101 MHz, DMSO) δ 156.47, 139.31, 137.97, 136.13, 128.36, 127.05, 123.40, 121.83, 121.75, 117.92, 117.53, 69.88, 68.80, 57.96, 48.58, 32.02, 29.90. ESI–MS calcd for $C_{18}H_{23}N_2O_2S$ $[M + H]^+$ 331.1475; found 331.1475

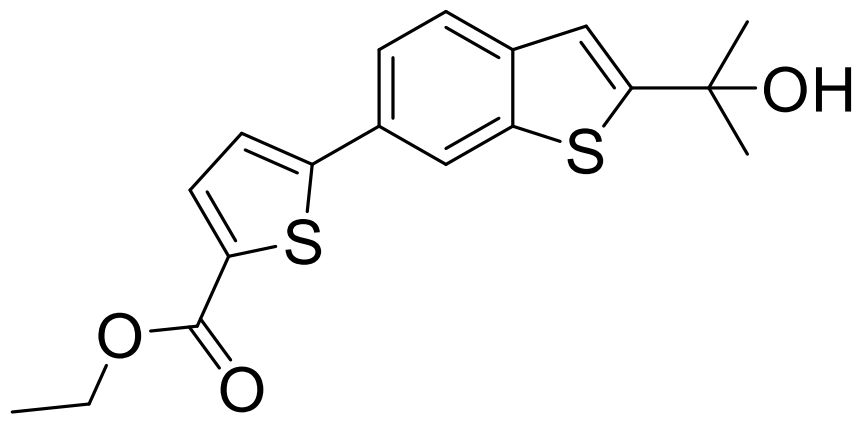

YKJ-213

Ethyl 5-(2-(2-hydroxypropan-2-yl)benzo[*b*]thiophen-6-yl)thiophene-2-carboxylate

Yield: 86.2% ^{1}H NMR (500 MHz, DMSO) δ 8.37 – 8.33 (m, 1H), 8.32 – 8.27 (m, 2H), 7.80 – 7.71 (m, 2H), 7.21 (s, 1H), 5.65 (s, 1H), 4.33 (q, *J* = 7.1 Hz, 2H), 1.57 (s, 6H), 1.33 (t, *J* = 7.1 Hz, 3H). ^{13}C NMR (126 MHz, DMSO) δ 161.43, 158.21, 142.18, 139.32, 139.31, 133.88, 131.94, 129.61, 127.85, 123.54, 122.61, 119.77, 117.50, 70.00, 61.10, 32.04, 14.23. ESI–MS calcd for $C_{18}H_{17}O_2S_2$ $[M - OH]^+$ 329.0664; found 329.0670

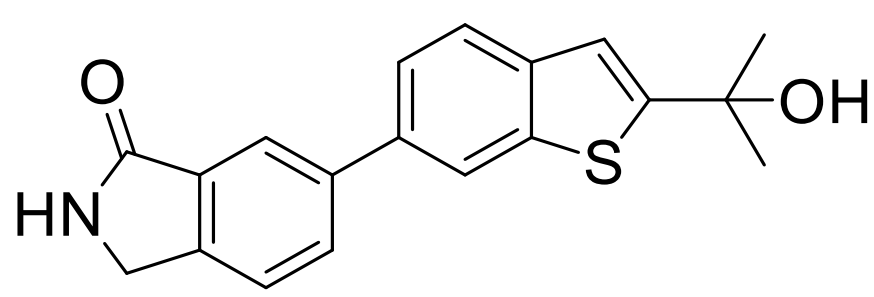

YKJ-214

6-(2-(2-Hydroxypropan-2-yl)benzo[*b*]thiophen-6-yl)isoindolin-1-one

Yield: 92.3% ^{1}H NMR (400 MHz, DMSO) δ 8.62 (s, 1H), 8.28 (s, 1H), 8.00 – 7.94 (m, 2H), 7.83 (d, *J* = 8.3 Hz, 1H), 7.72 – 7.62 (m, 2H), 7.24 (s, 1H), 5.66 (s, 1H), 4.43 (s, 2H), 1.59 (s, 6H). ^{13}C NMR (101 MHz, DMSO) δ 169.81, 158.24, 142.96, 140.05, 139.44, 139.33, 135.11, 133.46, 130.12, 124.27, 123.60, 123.33, 120.69, 120.53, 117.35, 70.00, 44.77, 32.06. ESI–MS calcd for $C_{19}H_{18}NO_2S$ $[M + H]^+$ 324.1053; found 324.1052

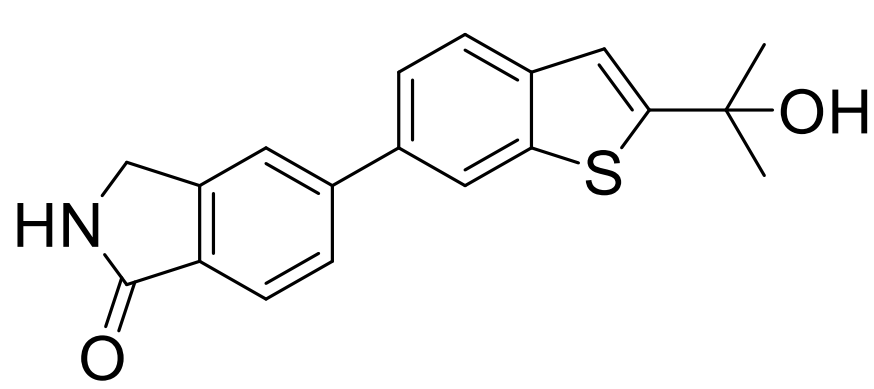

YKJ-215

5-(2-(2-Hydroxypropan-2-yl)benzo[*b*]thiophen-6-yl)isoindolin-1-one

Yield: 43.6% ^{1}H NMR (400 MHz, DMSO) δ 8.56 (s, 1H), 8.30 – 8.25 (m, 1H), 7.93 (s, 1H), 7.85 (s, 1H), 7.83 (s, 1H), 7.77 – 7.66 (m, 2H), 7.25 (s, 1H), 5.67 (s, 1H), 4.44 (s, 2H), 1.59 (s, 6H). ^{13}C NMR (101 MHz, DMSO) δ 169.77, 158.54, 145.02, 143.33, 139.58, 139.40, 135.12, 131.43, 126.63, 123.62, 123.47, 123.23, 122.05, 120.76, 117.41, 70.02, 45.00, 32.05. ESI–MS calcd for $C_{19}H_{18}NO_2S$ $[M + H]^+$ 324.1053; found 324.1051

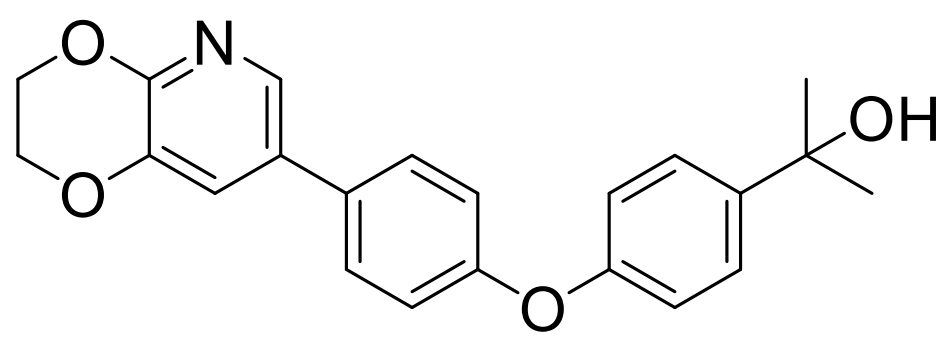

YKJ-216

2-(4-(4-(2,3-Dihydro-[1,4]dioxino[2,3-*b*]pyridin-7-yl)phenoxy)phenyl)propan-2-ol

Yield: 54.2% ^{1}H NMR (400 MHz, DMSO) δ 8.03 (d, *J* = 2.2 Hz, 1H), 7.69 – 7.61 (m, 2H), 7.56 (d, *J* = 2.3 Hz, 1H), 7.53 – 7.45 (m, 2H), 7.07 – 6.95 (m, 4H), 5.01 (s, 1H), 4.46 – 4.40 (m, 2H), 4.32 – 4.25 (m, 2H), 1.43 (s, 6H). ^{13}C NMR (101 MHz, DMSO) δ 156.90, 154.32, 150.15, 146.12, 139.12, 136.63, 131.46, 130.55, 128.07, 126.27, 122.26, 118.54, 118.31, 70.41, 64.73, 63.78, 31.98. ESI–MS calcd for $C_{22}H_{22}NO_4$ $[M + H]^+$ 364.1543; found 364.1550

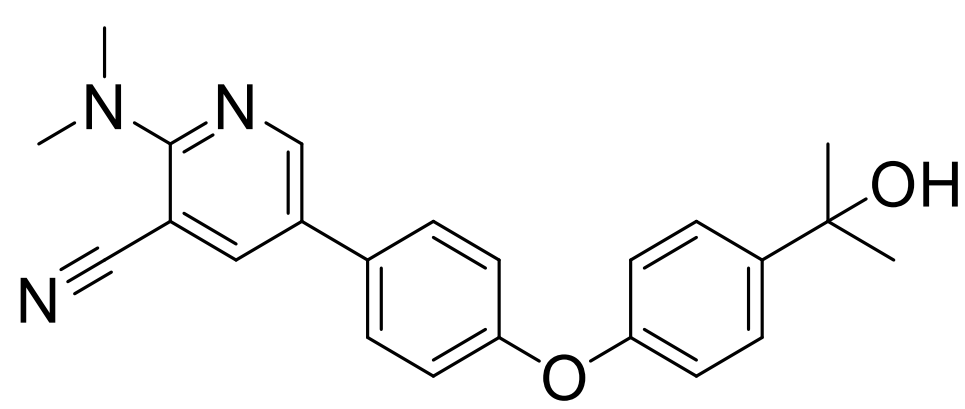

YKJ-217

2-(Dimethylamino)-5-(4-(4-(2-hydroxypropan-2-yl)phenoxy)phenyl)nicotinonitrile

Yield: 83.0% ^{1}H NMR (400 MHz, DMSO) δ 8.67 (d, *J* = 2.6 Hz, 1H), 8.25 (d, *J* = 2.5 Hz, 1H), 7.72 – 7.65 (m, 2H), 7.52 – 7.46 (m, 2H), 7.05 – 7.01 (m, 2H), 7.00 – 6.96 (m, 2H), 5.01 (s, 1H), 3.33 (s, 6H), 1.43 (s, 6H). ^{13}C NMR (101 MHz, DMSO) δ 157.89, 156.72, 154.37, 149.75, 146.07, 141.73, 130.45, 127.46, 126.26, 123.90, 118.81, 118.64, 118.23, 90.30, 70.40, 40.02, 31.97. ESI–MS calcd for $C_{23}H_{24}N_3O_2$ $[M + H]^+$ 374.1863; found 374.1863

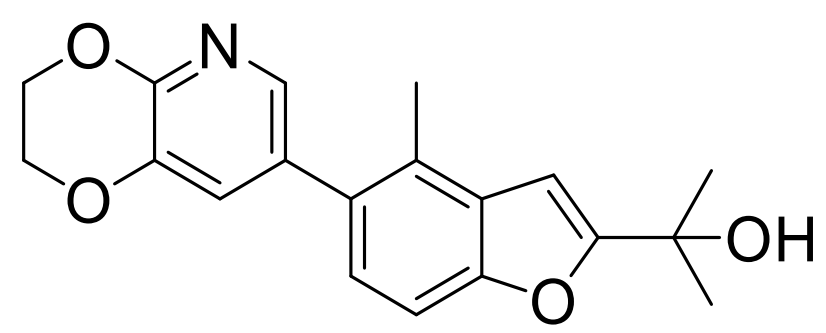

YKJ-218

2-(5-(2,3-Dihydro-[1,4]dioxino[2,3-*b*]pyridin-7-yl)-4-methylbenzofuran-2-yl)propan-2-ol

Yield: 63.5% ^{1}H NMR (400 MHz, DMSO) δ 7.71 (d, *J* = 2.1 Hz, 1H), 7.40 (d, *J* = 8.4 Hz, 1H), 7.29 (d, *J* = 2.1 Hz, 1H), 7.09 (d, *J* = 8.4 Hz, 1H), 6.77 (d, *J* = 0.9 Hz, 1H), 5.41 (s, 1H), 4.48 – 4.42 (m, 2H), 4.33 – 4.27 (m, 2H), 2.36 (s, 3H), 1.53 (s, 6H). ^{13}C NMR (101 MHz, DMSO) δ 164.97, 153.02, 149.70, 139.08, 138.44, 132.00, 131.27, 128.76, 127.89, 125.68, 125.35, 108.49, 99.24, 67.60, 64.67, 63.75, 29.02, 16.46. ESI–MS calcd for $C_{19}H_{20}NO_4$ $[M + H]^+$ 326.1387 found 326.1388

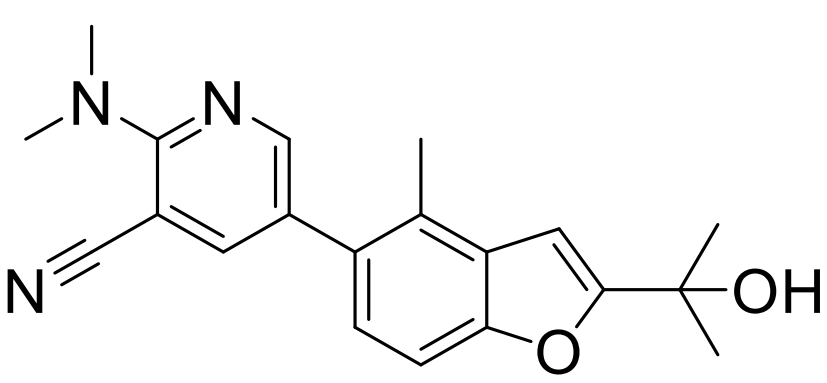

YKJ-219

2-(Dimethylamino)-5-(2-(2-hydroxypropan-2-yl)-4-methylbenzofuran-5-yl)nicotinonitrile

Yield: 89.7% ^{1}H NMR (400 MHz, DMSO) δ 8.35 (d, *J* = 2.4 Hz, 1H), 7.98 (d, *J* = 2.4 Hz, 1H), 7.42 (d, *J* = 8.4 Hz, 1H), 7.12 (d, *J* = 8.4 Hz, 1H), 6.77 (d, *J* = 0.9 Hz, 1H), 5.41 (s, 1H), 3.32 (s, 6H), 2.37 (s, 3H), 1.54 (s, 6H). ^{13}C NMR (101 MHz, DMSO) δ 164.99, 157.80, 153.07, 152.20, 144.53, 130.26, 128.84, 128.05, 125.58, 125.50, 118.82, 108.65, 99.26, 89.83, 67.59, 40.05, 29.01, 16.42. ESI–MS calcd for $C_{20}H_{22}N_3O_2$ $[M + H]^+$ 336.1707; found 336.1708

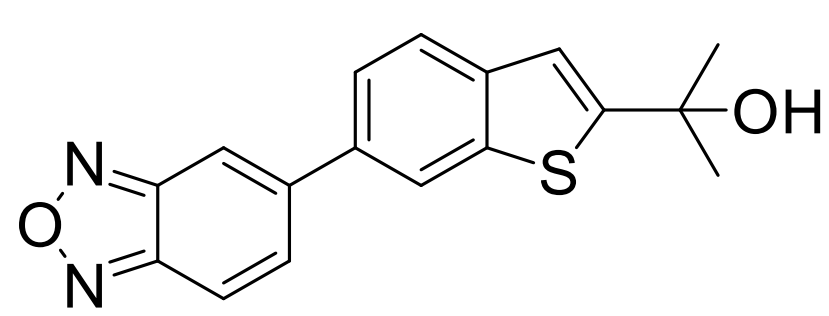

YKJ-220

2-(6-(Benzo[*c*][1,2,5]oxadiazol-5-yl)benzo[*b*]thiophen-2-yl)propan-2-ol

Yield: 70.9% ^{1}H NMR (400 MHz, DMSO) δ 8.47 (s, 1H), 8.36 (t, *J* = 1.3 Hz, 1H), 8.21 – 8.07 (m, 2H), 7.92 – 7.81 (m, 2H), 7.29 (s, 1H), 5.71 (s, 1H), 1.59 (s, 6H). ^{13}C NMR (101 MHz, DMSO) δ 159.65, 149.64, 148.34, 143.70, 140.48, 139.33, 133.62, 133.04, 123.72, 123.50, 121.39, 117.50, 116.48, 111.57, 70.06, 32.02. ESI–MS calcd for $C_{17}H_{15}N_2O_2S$ $[M + H]^+$ 311.0849; found 311.0852

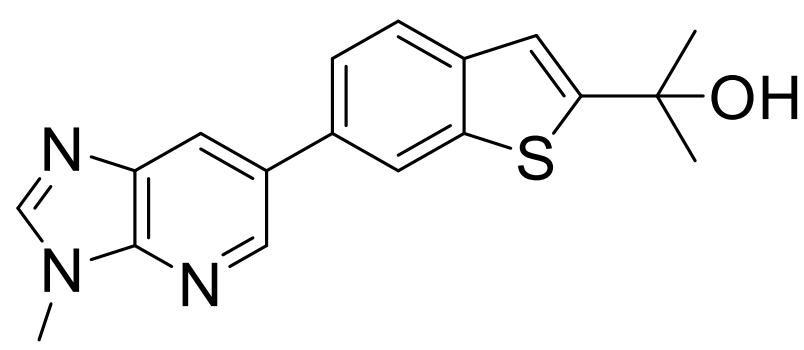

YKJ-221

2-(6-(3-Methyl-3*H*-imidazo[4,5-*b*]pyridin-6-yl)benzo[*b*]thiophen-2-yl)propan-2-ol

Yield: 59.7% ^{1}H NMR (500 MHz, DMSO) δ 8.76 (d, *J* = 2.0 Hz, 1H), 8.47 (s, 1H), 8.39 (d, *J* = 2.0 Hz, 1H), 8.30 (s, 1H), 7.84 (d, *J* = 8.2 Hz, 1H), 7.72 (dd, *J* = 8.3, 1.7 Hz, 1H), 7.25 (s, 1H), 5.67 (s, 1H), 3.88 (s, 3H), 1.59 (s, 6H). ^{13}C NMR (126 MHz, DMSO) δ 157.97, 146.77, 142.85, 139.49, 139.06, 135.06, 133.71, 130.90, 125.17, 123.70, 123.64, 120.72, 117.39, 70.01, 32.08, 29.53 (one low field carbon not observed). ESI–MS calcd for $C_{18}H_{18}N_3OS$ $[M + H]^+$ 324.1165; found 324.1168

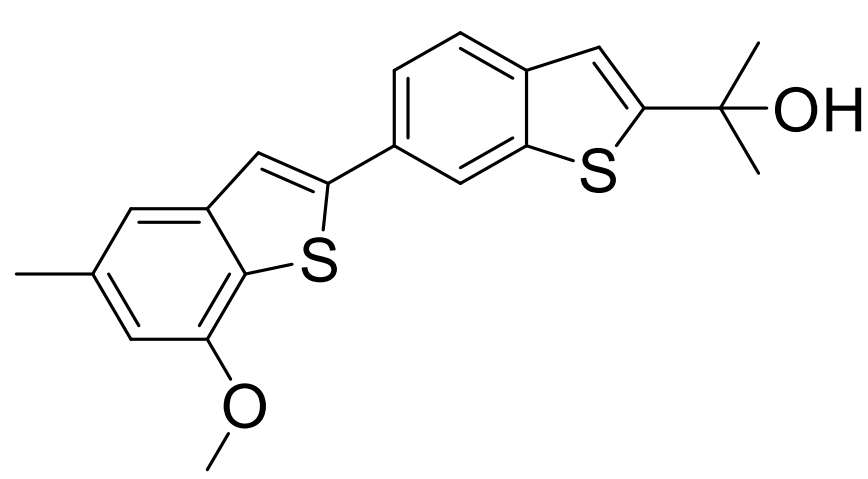

YKJ-222

2-(7-Methoxy-5-methyl-[2,6'-bibenzo[*b*]thiophen]-2'-yl)propan-2-ol

Yield: 49.1% ^{1}H NMR (500 MHz, DMSO) δ 8.32 (s, 1H), 7.82 – 7.78 (m, 2H), 7.75 – 7.71 (m, 1H), 7.24 (s, 2H), 6.79 (s, 1H), 5.69 (s, 1H), 3.96 (s, 3H), 2.43 (s, 3H), 1.58 (s, 6H). ^{13}C NMR (126 MHz, DMSO) δ 158.87, 153.47, 143.76, 142.18, 139.92, 139.35, 135.94, 129.17, 123.97, 123.70, 122.50, 119.88, 119.57, 117.62, 116.09, 106.71, 70.03, 55.64, 32.01, 21.52. ESI–MS calcd for $C_{21}H_{19}OS_2$ $[M - OH]^+$ 351.0872; found 351.0873

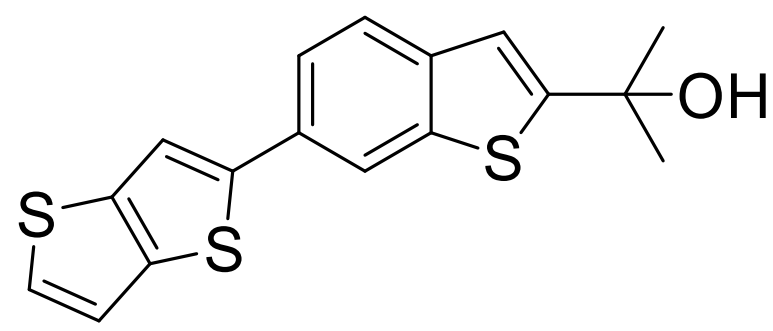

YKJ-223

2-(6-(Thieno[3,2-*b*]thiophen-2-yl)benzo[*b*]thiophen-2-yl)propan-2-ol

Yield: 50.7% ^{1}H NMR (500 MHz, DMSO) δ 8.23 (s, 1H), 7.93 (s, 1H), 7.79 (d, *J* = 8.3 Hz, 1H), 7.69 – 7.64 (m, 2H), 7.47 (d, *J* = 5.1 Hz, 1H), 7.23 (s, 1H), 5.68 (s, 1H), 1.58 (s, 6H). ^{13}C NMR (126 MHz, DMSO) δ 158.47, 145.49, 139.83, 139.54, 139.42, 137.74, 129.78, 128.13, 123.74, 122.00, 120.15, 118.75, 117.60, 116.02, 70.01, 32.02. ESI–MS calcd for $C_{17}H_{13}S_3$ [M + H]$^+$ 313.0174; found 313.0177

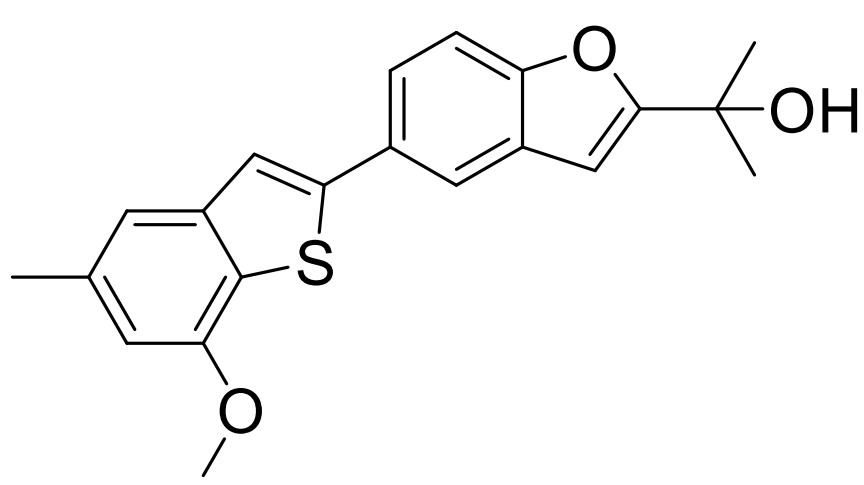

YKJ-224

2-(5-(7-Methoxy-5-methylbenzo[*b*]thiophen-2-yl)benzofuran-2-yl)propan-2-ol

Yield: 43.9% ^{1}H NMR (500 MHz, DMSO) δ 7.96 (d, *J* = 1.9 Hz, 1H), 7.71 (s, 1H), 7.66 (dd, *J* = 8.6, 2.0 Hz, 1H), 7.62 (d, *J* = 8.6 Hz, 1H), 7.24 (s, 1H), 6.78 (s, 1H), 6.74 (d, *J* = 0.9 Hz, 1H), 5.47 (s, 1H), 3.95 (s, 3H), 2.43 (s, 3H), 1.53 (s, 6H). ^{13}C NMR (126 MHz, DMSO) δ 166.04, 154.02, 153.46, 144.06, 142.22, 135.87, 129.08, 128.64, 123.88, 122.22, 119.49, 118.43, 116.06, 111.63, 106.57, 100.32, 67.59, 55.62, 28.96, 21.53. ESI–MS calcd for $C_{21}H_{19}O_2S$ [M - OH]$^+$ 335.1100; found 335.1101

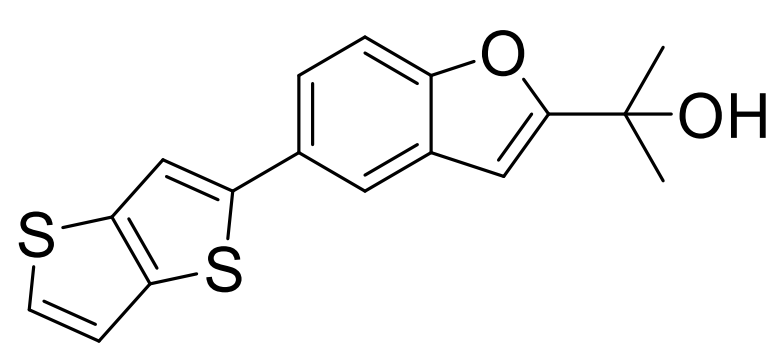

YKJ-225

2-(5-(Thieno[3,2-*b*]thiophen-2-yl)benzofuran-2-yl)propan-2-ol

Yield: 50.1% ^{1}H NMR (500 MHz, DMSO) δ 7.88 (s, 1H), 7.84 (s, 1H), 7.65 (d, *J* = 5.2 Hz, 1H), 7.61 – 7.59 (m, 2H), 7.45 (d, *J* = 5.3 Hz, 1H), 6.73 (s, 1H), 5.46 (s, 1H), 1.53 (s, 6H). ^{13}C NMR (126 MHz, DMSO) δ 165.99, 153.76, 145.79, 139.77, 137.50, 129.22, 129.11, 127.81, 121.72, 120.10, 117.69, 115.70, 111.66, 100.28, 67.59, 28.96. ESI–MS calcd for $C_{17}H_{13}OS_2$ $[M - OH]^+$ 297.0402; found 297.0405

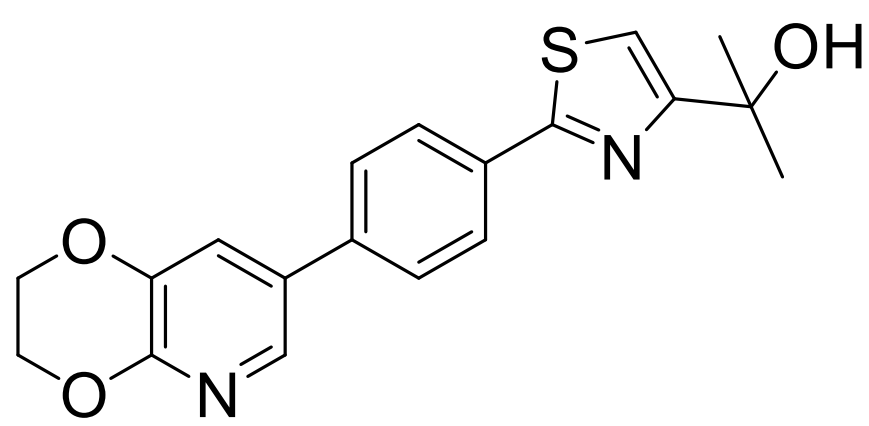

YKJ-226

2-(2-(4-(2,3-Dihydro-[1,4]dioxino[2,3-*b*]pyridin-7-yl)phenyl)thiazol-4-yl)propan-2-ol

Yield: 64.8% ^{1}H NMR (500 MHz, DMSO) δ 8.15 (d, *J* = 2.2 Hz, 1H), 7.98 (d, *J* = 8.5 Hz, 2H), 7.80 (d, *J* = 8.5 Hz, 2H), 7.68 (d, *J* = 2.2 Hz, 1H), 7.43 (s, 1H), 5.22 (s, 1H), 4.48 – 4.43 (m, 2H), 4.34 – 4.28 (m, 2H), 1.52 (s, 6H). ^{13}C NMR (126 MHz, DMSO) δ 166.43, 165.74, 150.77, 139.27, 137.94, 137.01, 132.45, 130.14, 127.09, 126.60, 122.42, 112.86, 70.47, 64.85, 63.81, 30.59. ESI–MS calcd for $C_{19}H_{19}N_2O_3S$ $[M + H]^+$ 355.1111; found 355.1111

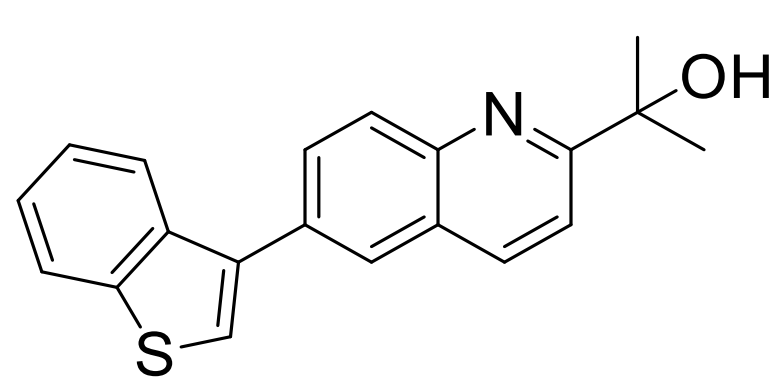

YKJ-227

2-(6-(Benzo[*b*]thiophen-3-yl)quinolin-2-yl)propan-2-ol

Yield: 98.8% ^{1}H NMR (500 MHz, DMSO) δ 8.45 (d, *J* = 8.6 Hz, 1H), 8.21 (d, *J* = 2.0 Hz, 1H), 8.14 – 8.08 (m, 2H), 8.03 – 7.96 (m, 3H), 7.92 (d, *J* = 8.6 Hz, 1H), 7.51 – 7.45 (m, 2H), 5.48 (s, 1H), 1.57 (s, 6H). ^{13}C NMR (126 MHz, DMSO) δ 168.56, 145.63, 140.21, 137.20, 136.74, 136.38, 132.77, 130.16, 129.21, 126.84, 126.71, 125.55, 124.87, 124.73, 123.36, 122.51, 118.29, 72.74, 30.52. ESI–MS calcd for $C_{20}H_{18}NOS$ $[M + H]^+$ 320.1104; found 320.1106

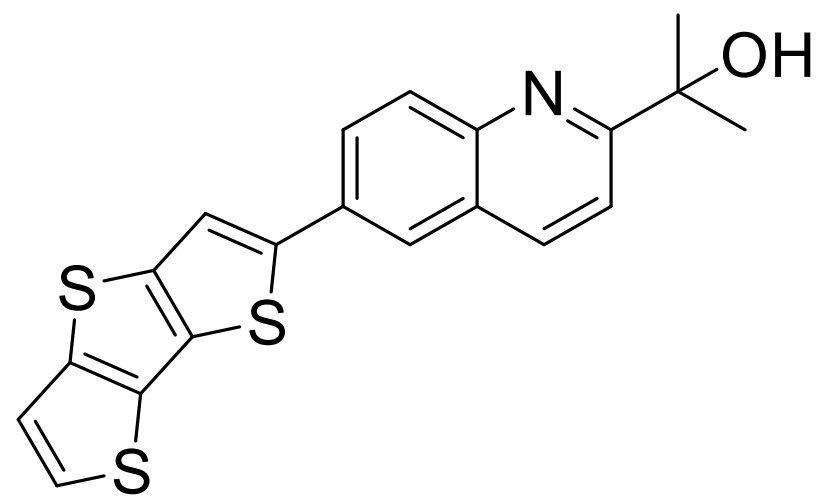

YKJ-228

2-(6-(Dithieno[3,2-*b*:2',3'-*d*]thiophen-2-yl)quinolin-2-yl)propan-2-ol

Yield: 85.8% ^{1}H NMR (500 MHz, DMSO) δ 8.40 (d, *J* = 8.6 Hz, 1H), 8.24 (d, *J* = 2.2 Hz, 1H), 8.15 (s, 1H), 8.12 (dd, *J* = 8.8, 2.2 Hz, 1H), 8.02 (d, *J* = 8.8 Hz, 1H), 7.90 (d, *J* = 8.6 Hz, 1H), 7.76 (d, *J* = 5.1 Hz, 1H), 7.57 (d, *J* = 5.2 Hz, 1H), 5.46 (s, 1H), 1.55 (s, 6H). ^{13}C NMR (126 MHz, DMSO) δ 168.62, 145.85, 143.27, 142.26, 141.63, 136.57, 131.33, 130.23, 129.65, 129.46, 128.05, 126.98, 123.17, 121.58, 118.78, 118.69, 72.72, 30.45 (one low field carbon not observed). ESI–MS calcd for $C_{20}H_{16}NOS_3$ $[M + H]^+$ 382.0389; found 382.0389

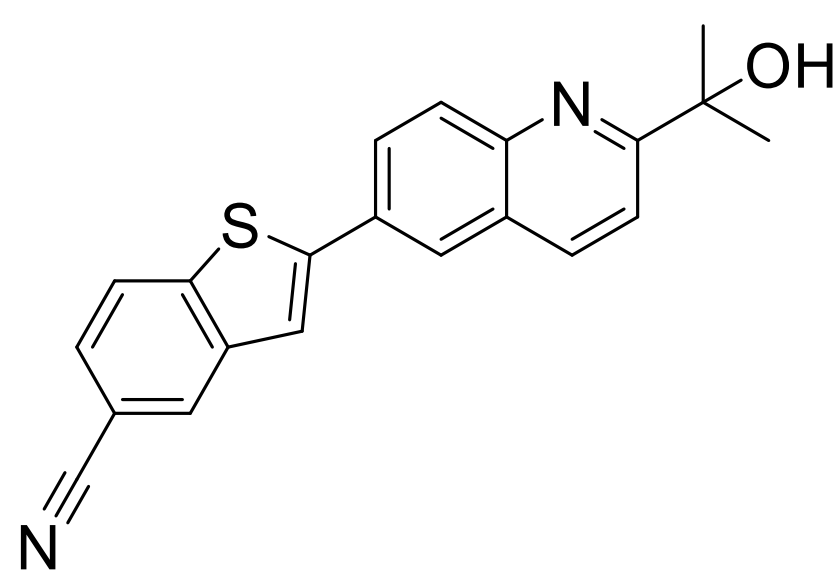

YKJ-229

2-(2-(2-Hydroxypropan-2-yl)quinolin-6-yl)benzo[*b*]thiophene-5-carbonitrile

Yield: quantitative ^{1}H NMR (500 MHz, DMSO) δ 8.46 (d, *J* = 8.6 Hz, 1H), 8.41 (d, *J* = 1.6 Hz, 1H), 8.38 (d, *J* = 2.3 Hz, 1H), 8.28 (d, *J* = 8.3 Hz, 1H), 8.20 (dd, *J* = 8.8, 2.2 Hz, 1H), 8.13 (s, 1H), 8.07 (d, *J* = 8.8 Hz, 1H), 7.93 (d, *J* = 8.6 Hz, 1H), 7.75 (dd, *J* = 8.4, 1.6 Hz, 1H), 5.48 (s, 1H), 1.56 (s, 6H). ^{13}C NMR (126 MHz, DMSO) δ 169.28, 146.34, 145.44, 143.15, 140.39, 136.90, 130.13, 129.78, 128.34, 127.42, 126.86, 126.76, 125.23, 124.08, 120.66, 119.26, 118.80, 107.65, 72.77, 30.44. ESI–MS calcd for $C_{21}H_{17}N_2OS$ $[M + H]^+$ 345.1056; found 345.1056

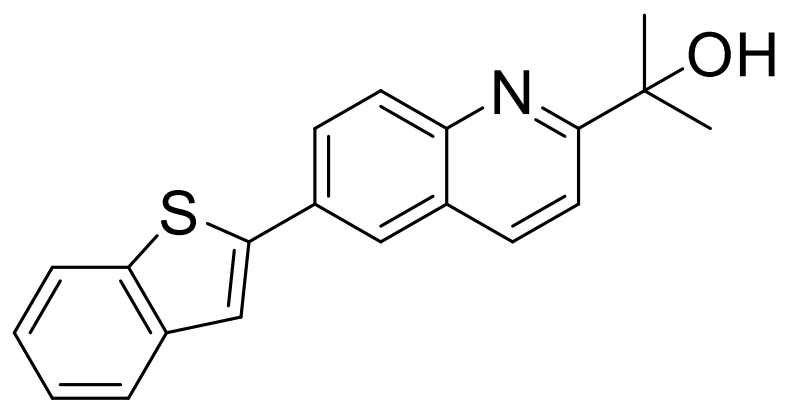

YKJ-230

2-(6-(Benzo[*b*]thiophen-2-yl)quinolin-2-yl)propan-2-ol

Yield: 84.7% ^{1}H NMR (500 MHz, DMSO) δ 8.44 (d, *J* = 8.6 Hz, 1H), 8.32 (d, *J* = 2.1 Hz, 1H), 8.20 (dd, *J* = 8.8, 2.2 Hz, 1H), 8.07 – 8.00 (m, 3H), 7.93 – 7.88 (m, 2H), 7.44 – 7.37 (m, 2H), 5.47 (s, 1H), 1.56 (s, 6H). ^{13}C NMR (126 MHz, DMSO) δ 168.87, 146.09, 142.58, 140.51, 138.81, 136.75, 130.99, 129.57, 127.42, 126.90, 124.97, 124.53, 123.95, 122.55, 121.11, 118.67, 72.74, 30.45 (one low field carbon not observed). ESI–MS calcd for $C_{20}H_{18}NOS$ $[M + H]^+$ 320.1104; found 320.1104

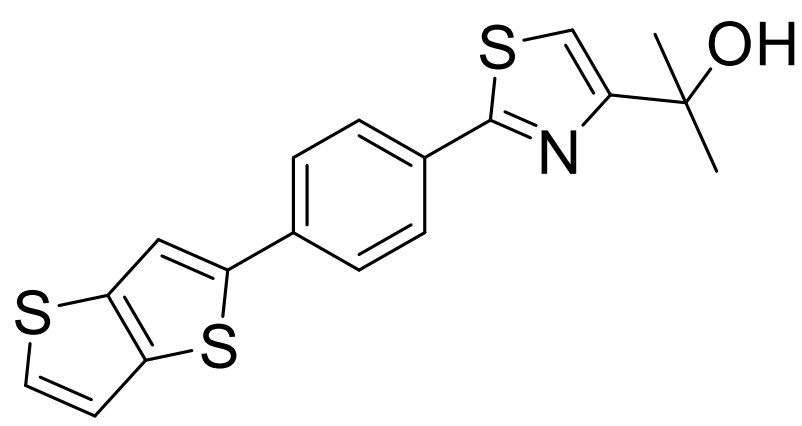

YKJ-231

2-(2-(4-(Thieno[3,2-*b*]thiophen-2-yl)phenyl)thiazol-4-yl)propan-2-ol

Yield: 78.4% ^{1}H NMR (500 MHz, DMSO) δ 8.01 (s, 1H), 7.98 (d, *J* = 8.5 Hz, 2H), 7.81 (d, *J* = 8.5 Hz, 2H), 7.72 (d, *J* = 5.2 Hz, 1H), 7.48 (d, *J* = 5.2 Hz, 1H), 7.44 (s, 1H), 5.23 (s, 1H), 1.52 (s, 6H). ^{13}C NMR (126 MHz, DMSO) δ 166.47, 165.60, 144.13, 139.94, 138.47, 135.50, 132.53, 128.99, 126.79, 125.82, 120.21, 117.09, 112.91, 70.46, 30.60. ESI–MS calcd for $C_{18}H_{14}NS_3$ $[M - OH]^+$ 340.0283; found 340.0282

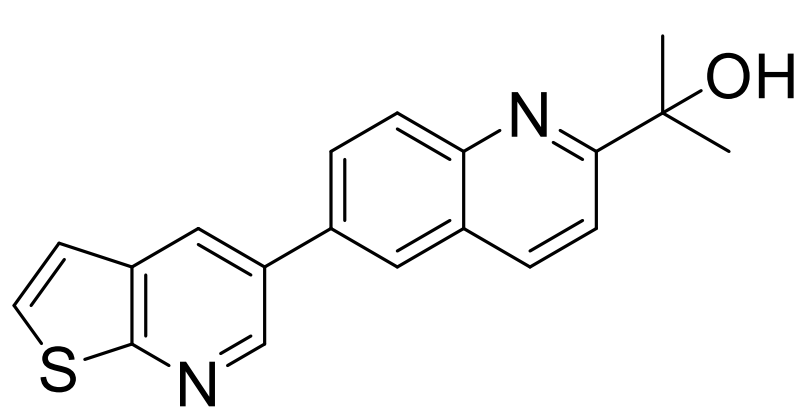

YKJ-232

2-(6-(Thieno[2,3-*b*]pyridin-5-yl)quinolin-2-yl)propan-2-ol

Yield: 83.4% ^{1}H NMR (500 MHz, DMSO) δ 9.05 (d, *J* = 2.3 Hz, 1H), 8.72 (d, *J* = 2.3 Hz, 1H), 8.45 – 8.39 (m, 2H), 8.18 (dd, *J* = 8.8, 2.2 Hz, 1H), 8.10 (d, *J* = 8.7 Hz, 1H), 7.97 (d, *J* = 6.0 Hz, 1H), 7.92 (d, *J* = 8.6 Hz, 1H), 7.54 (d, *J* = 6.0 Hz, 1H), 5.48 (s, 1H), 1.57 (s, 6H). ^{13}C NMR (126 MHz, DMSO) δ 168.76, 160.21, 145.80, 145.52, 136.81, 134.68, 132.63, 131.44, 129.59, 129.53, 128.80, 128.63, 126.93, 125.83, 122.33, 118.45, 72.75, 30.49. ESI–MS calcd for $C_{19}H_{17}N_2OS$ [M + H]$^+$ 321.1056; found 321.1059

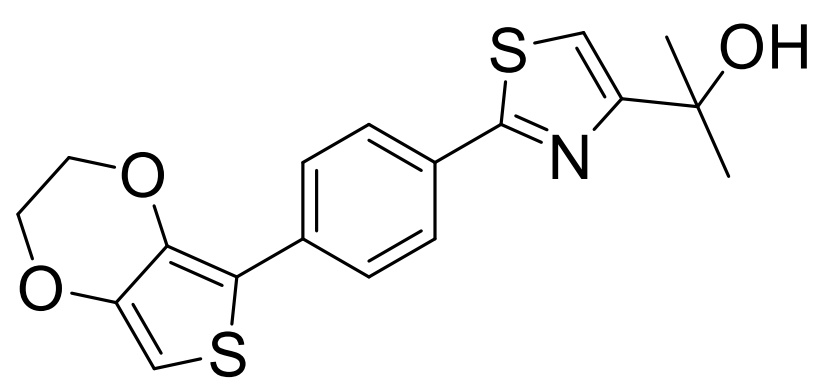

YKJ-233

2-(2-(4-(2,3-Dihydrothieno[3,4-*b*][1,4]dioxin-5-yl)phenyl)thiazol-4-yl)propan-2-ol

Yield: 69.3% ^{1}H NMR (500 MHz, DMSO) δ 7.92 (d, *J* = 8.5 Hz, 2H), 7.76 (d, *J* = 8.7 Hz, 2H), 7.40 (s, 1H), 6.70 (s, 1H), 5.21 (s, 1H), 4.38 – 4.34 (m, 2H), 4.29 – 4.24 (m, 2H), 1.51 (s, 6H). ^{13}C NMR (126 MHz, DMSO) δ 166.38, 165.87, 142.32, 139.31, 134.30, 131.22, 126.47, 125.67, 115.10, 112.60, 99.07, 70.45, 64.90, 64.11, 30.60. ESI–MS calcd for $C_{18}H_{18}NO_3S_2$ [M + H]$^+$ 360.0723; found 360.0724

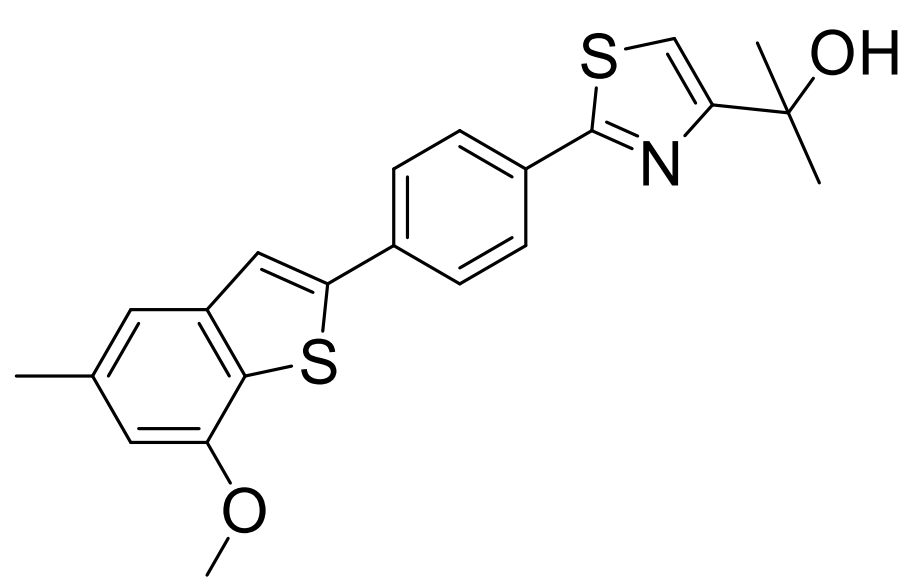

YKJ-234

2-(2-(4-(7-Methoxy-5-methylbenzo[*b*]thiophen-2-yl)phenyl)thiazol-4-yl)propan-2-ol

Yield: 76.9% ^{1}H NMR (500 MHz, DMSO) δ 8.00 (d, *J* = 8.4 Hz, 2H), 7.90 – 7.86 (m, 3H), 7.45 (s, 1H), 7.27 (s, 1H), 6.82 (s, 1H), 5.24 (s, 1H), 3.96 (s, 3H), 2.44 (s, 3H), 1.52 (s, 6H). ^{13}C NMR (126 MHz, DMSO) δ 166.53, 165.53, 153.49, 142.48, 142.00, 136.15, 134.91, 133.03, 126.73,

126.59, 124.32, 120.98, 116.34, 113.08, 107.13, 70.48, 55.70, 30.61, 21.53. ESI–MS calcd for $C_{22}H_{22}NO_2S_2$ $[M + H]^+$ 396.1086; found 396.1086

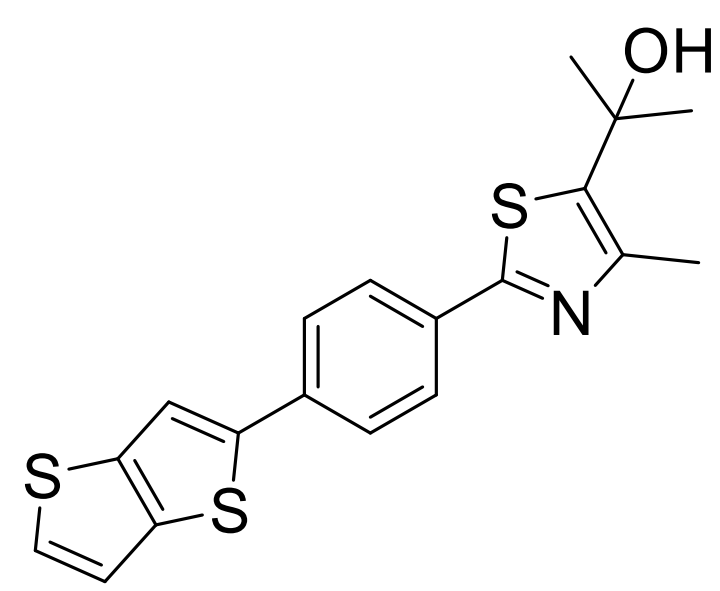

YKJ-235

2-(4-Methyl-2-(4-(thieno[3,2-*b*]thiophen-2-yl)phenyl)thiazol-5-yl)propan-2-ol

Yield: 82.8% ^{1}H NMR (500 MHz, DMSO) δ 7.98 (s, 1H), 7.90 (d, *J* = 8.4 Hz, 2H), 7.77 (d, *J* = 8.4 Hz, 2H), 7.71 (d, *J* = 5.2 Hz, 1H), 7.48 (d, *J* = 5.2 Hz, 1H), 5.72 (s, 1H), 2.46 (s, 3H), 1.56 (s, 6H). ^{13}C NMR (126 MHz, DMSO) δ 160.35, 146.17, 144.25, 143.38, 139.94, 138.36, 135.04, 132.64, 128.88, 126.25, 125.78, 120.20, 116.88, 69.27, 31.00, 16.59. ESI–MS calcd for $C_{19}H_{18}NOS_3$ $[M + H]^+$ 372.0545; found 372.0551

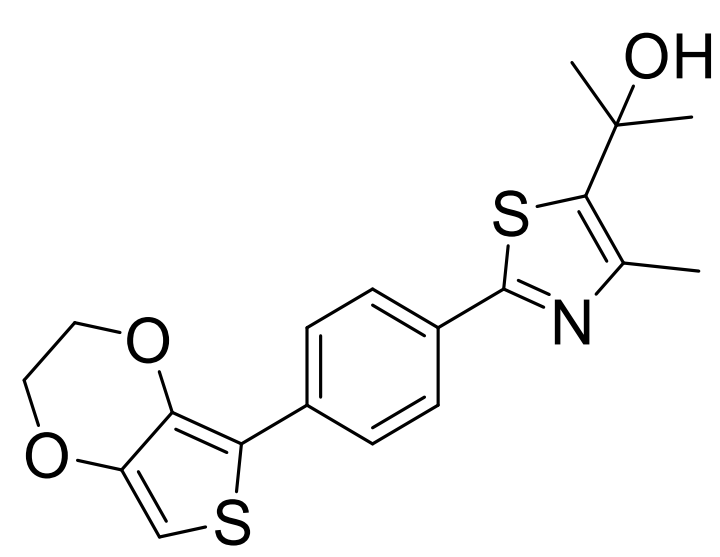

YKJ-236

2-(2-(4-(2,3-Dihydrothieno[3,4-*b*][1,4]dioxin-5-yl)phenyl)-4-methylthiazol-5-yl)propan-2-ol

Yield: 73.9% ^{1}H NMR (500 MHz, DMSO) δ 7.85 (d, *J* = 8.5 Hz, 2H), 7.72 (d, *J* = 8.6 Hz, 2H), 6.69 (s, 1H), 5.69 (s, 1H), 4.37 – 4.34 (m, 2H), 4.30 – 4.24 (m, 2H), 2.45 (s, 3H), 1.55 (s, 6H). ^{13}C NMR (126 MHz, DMSO) δ 160.61, 146.05, 143.00, 142.30, 139.17, 133.82, 131.32, 125.93, 125.63, 115.18, 98.90, 69.25, 64.87, 64.10, 31.03, 16.58. ESI–MS calcd for $C_{19}H_{20}NO_3S_2$ $[M + H]^+$ 374.0879; found 378.0884

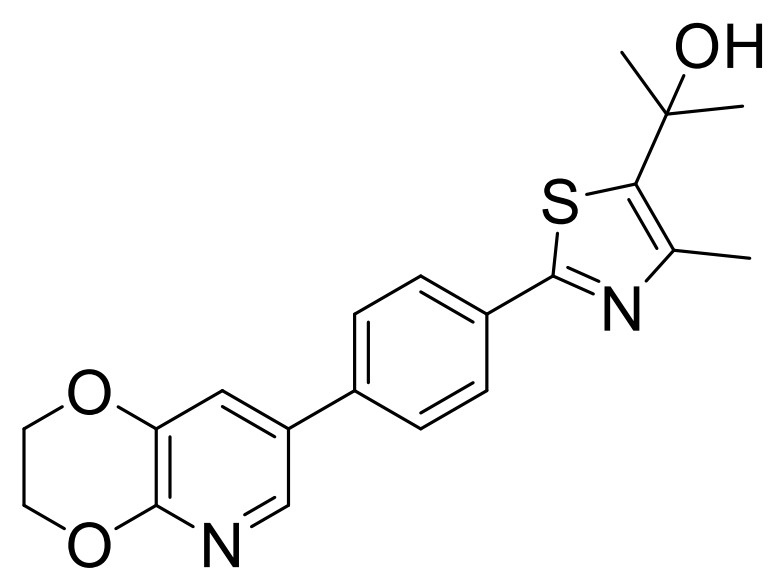

YKJ-237

2-(2-(4-(2,3-Dihydro-[1,4]dioxino[2,3-*b*]pyridin-7-yl)phenyl)-4-methylthiazol-5-yl)propan-2-ol

Yield: 48.5% ^{1}H NMR (500 MHz, DMSO) δ 8.13 (d, *J* = 2.3 Hz, 1H), 7.90 (d, *J* = 8.5 Hz, 2H), 7.76 (d, *J* = 8.5 Hz, 2H), 7.66 (d, *J* = 2.3 Hz, 1H), 5.71 (s, 1H), 4.48 – 4.43 (m, 2H), 4.33 – 4.28 (m, 2H), 2.46 (s, 3H), 1.56 (s, 6H). ^{13}C NMR (126 MHz, DMSO) δ 160.51, 150.69, 146.12, 143.30, 139.25, 137.48, 136.94, 132.53, 130.21, 127.04, 126.06, 122.36, 69.26, 64.82, 63.79, 31.01, 16.61. ESI–MS calcd for $C_{20}H_{21}N_2O_3S$ [M + H]$^+$ 369.1267; found 369.1269

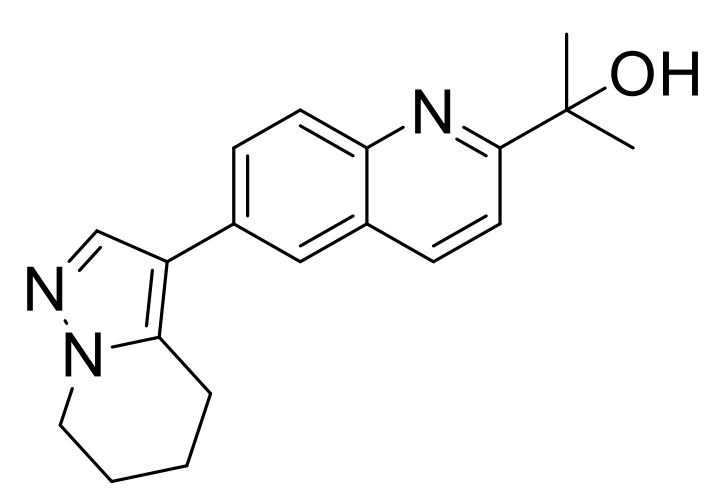

YKJ-238

2-(6-(4,5,6,7-Tetrahydropyrazolo[1,5-*a*]pyridin-3-yl)quinolin-2-yl)propan-2-ol

Yield: 80.1% ^{1}H NMR (500 MHz, DMSO) δ 8.31 (d, *J* = 8.6 Hz, 1H), 7.97 – 7.92 (m, 2H), 7.90 – 7.85 (m, 2H), 7.83 (d, *J* = 8.6 Hz, 1H), 5.42 (s, 1H), 4.13 (t, *J* = 6.1 Hz, 2H), 3.03 (t, *J* = 6.3 Hz, 2H), 2.05 – 1.99 (m, 2H), 1.90 – 1.84 (m, 2H), 1.54 (s, 6H). ^{13}C NMR (126 MHz, DMSO) δ 167.43, 144.57, 136.89, 136.20, 136.04, 131.11, 128.92, 128.69, 127.03, 123.29, 118.04, 116.84, 72.62, 47.85, 30.52, 22.91, 22.51, 19.96. ESI–MS calcd for $C_{19}H_{22}N_3O$ [M + H]$^+$ 308.1757; found 308.1759

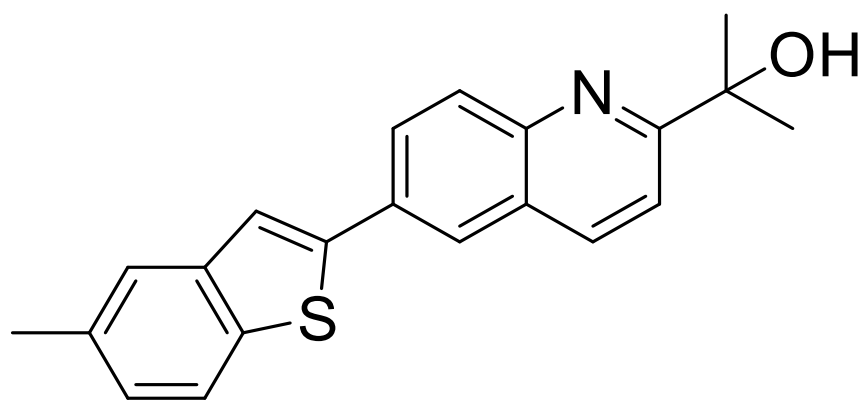

YKJ-239

2-(6-(5-Methylbenzo[*b*]thiophen-2-yl)quinolin-2-yl)propan-2-ol

Yield: quantitative ^{1}H NMR (500 MHz, DMSO) δ 8.43 (d, *J* = 8.6 Hz, 1H), 8.30 (d, *J* = 2.2 Hz, 1H), 8.18 (dd, *J* = 8.8, 2.2 Hz, 1H), 8.03 (d, *J* = 8.7 Hz, 1H), 7.97 (s, 1H), 7.89 (t, *J* = 8.3 Hz, 2H), 7.68 (s, 1H), 7.22 (dd, *J* = 8.1, 1.7 Hz, 1H), 5.46 (s, 1H), 2.44 (s, 3H), 1.55 (s, 6H). ^{13}C NMR (126 MHz, DMSO) δ 168.80, 146.06, 142.68, 140.84, 136.74, 136.05, 134.23, 131.12, 129.53, 127.42, 126.91, 126.62, 124.39, 123.75, 122.22, 120.82, 118.65, 72.73, 30.45, 21.01. ESI–MS calcd for $C_{21}H_{20}NOS$ $[M + H]^+$ 334.1260; found 334.1269

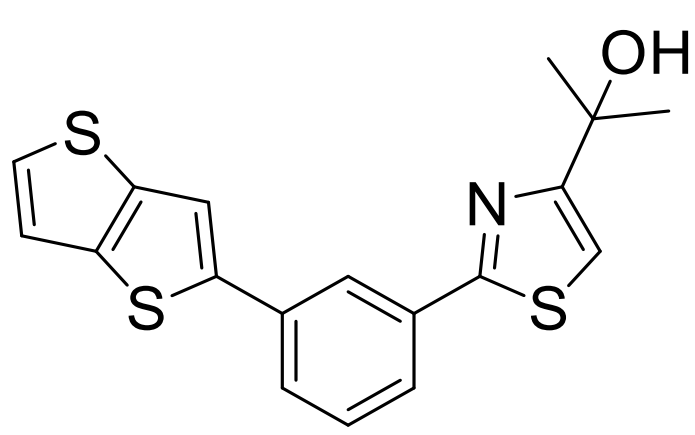

YKJ-240

2-(2-(3-(Thieno[3,2-*b*]thiophen-2-yl)phenyl)thiazol-4-yl)propan-2-ol

Yield: 64.6% ^{1}H NMR (500 MHz, DMSO) δ 8.18 (t, *J* = 1.8 Hz, 1H), 8.03 (s, 1H), 7.86 (d, *J* = 7.8 Hz, 1H), 7.79 (d, *J* = 7.9 Hz, 1H), 7.72 (d, *J* = 5.2 Hz, 1H), 7.57 (t, *J* = 7.8 Hz, 1H), 7.48 (d, *J* = 4.3 Hz, 2H), 5.25 (s, 1H), 1.53 (s, 6H). ^{13}C NMR (126 MHz, DMSO) δ 166.42, 165.62, 144.00, 139.87, 138.29, 135.03, 134.22, 130.24, 128.87, 126.80, 125.58, 122.30, 120.19, 117.18, 113.29, 70.47, 30.61. ESI–MS calcd for $C_{18}H_{16}NOS_3$ $[M + H]^+$ 358.0389; found 358.0388

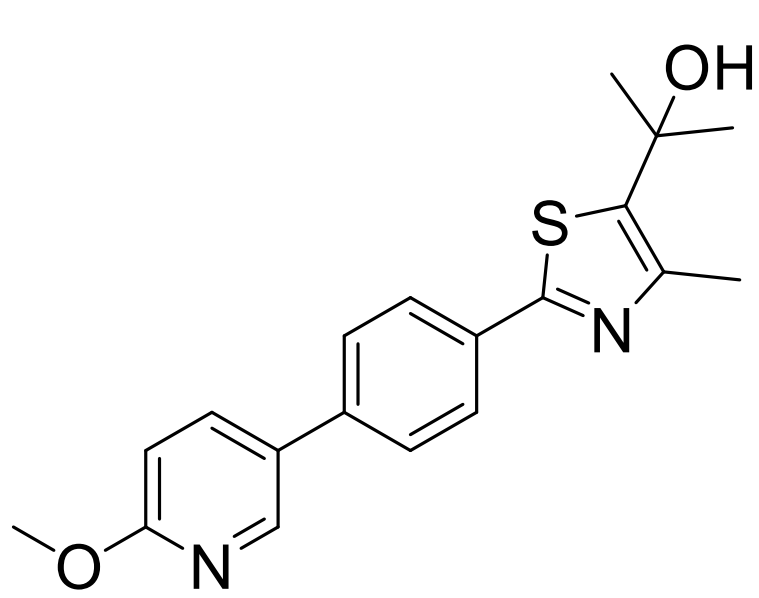

YKJ-241

2-(2-(4-(6-Methoxypyridin-3-yl)phenyl)-4-methylthiazol-5-yl)propan-2-ol

Yield: 80.5% ^{1}H NMR (500 MHz, DMSO) δ 8.55 (d, *J* = 2.6 Hz, 1H), 8.07 (dd, *J* = 8.7, 2.6 Hz, 1H), 7.92 (d, *J* = 8.5 Hz, 2H), 7.76 (d, *J* = 8.4 Hz, 2H), 6.93 (d, *J* = 8.7 Hz, 1H), 5.72 (s, 1H), 3.90 (s, 3H), 2.46 (s, 3H), 1.56 (s, 6H). ^{13}C NMR (126 MHz, DMSO) δ 163.31, 160.57, 146.11, 144.75, 143.26, 137.90, 137.46, 132.38, 128.44, 126.87, 126.12, 110.73, 69.27, 53.36, 31.02, 16.61. ESI–MS calcd for $C_{19}H_{21}N_2O_2S$ $[M + H]^+$ 341.1318; found 341.1312

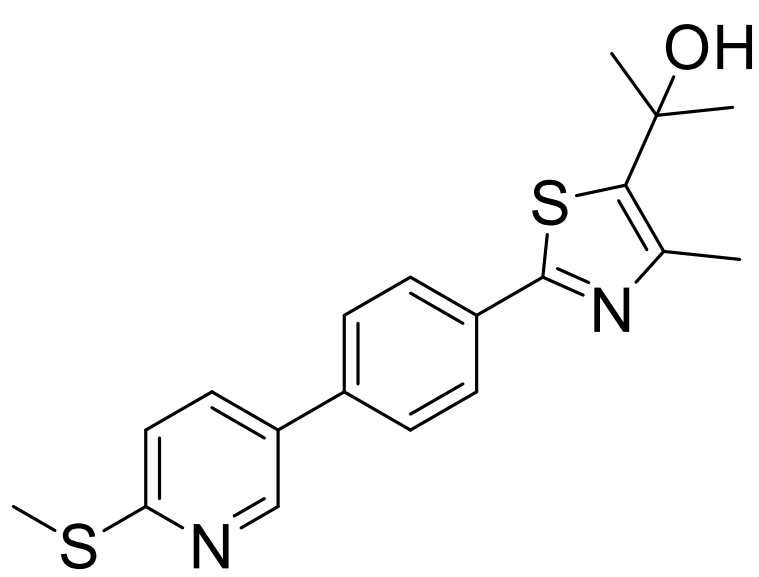

YKJ-242

2-(4-Methyl-2-(4-(6-(methylthio)pyridin-3-yl)phenyl)thiazol-5-yl)propan-2-ol

Yield: 60.3% ^{1}H NMR (500 MHz, DMSO) δ 8.82 (d, *J* = 1.6 Hz, 1H), 8.01 (dd, *J* = 8.4, 2.5 Hz, 1H), 7.94 (d, *J* = 8.5 Hz, 2H), 7.80 (d, *J* = 8.4 Hz, 2H), 7.40 (d, *J* = 8.3 Hz, 1H), 5.73 (s, 1H), 2.56 (s, 3H), 2.46 (s, 3H), 1.56 (s, 6H). ^{13}C NMR (126 MHz, DMSO) δ 160.47, 158.80, 147.11, 146.17, 143.43, 137.59, 134.41, 132.80, 130.48, 127.06, 126.17, 121.06, 69.28, 31.01, 16.61, 12.85. ESI–MS calcd for $C_{19}H_{21}N_2OS_2$ $[M + H]^+$ 357.1090; found 357.1089

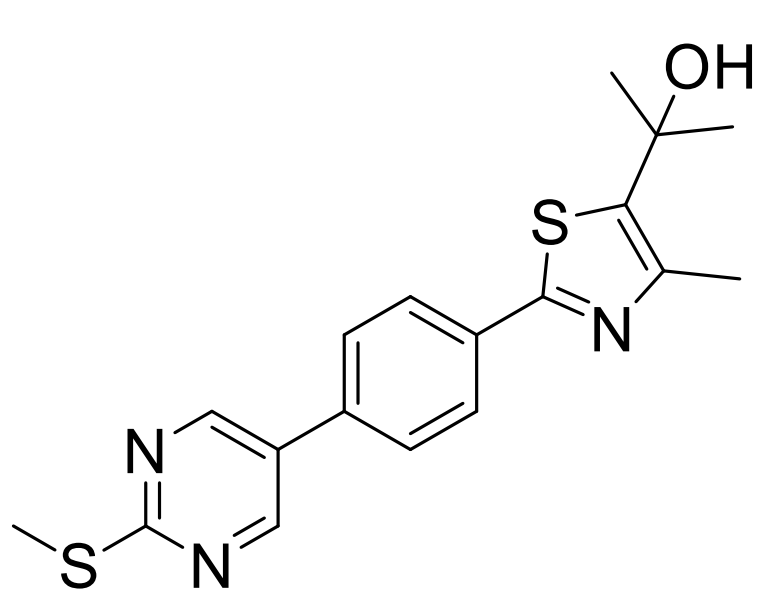

YKJ-243

2-(4-Methyl-2-(4-(2-(methylthio)pyrimidin-5-yl)phenyl)thiazol-5-yl)propan-2-ol

Yield: 82.5% ^{1}H NMR (500 MHz, DMSO) δ 9.03 (s, 2H), 7.96 (d, *J* = 8.5 Hz, 2H), 7.86 (d, *J* = 8.4 Hz, 2H), 5.74 (s, 1H), 2.57 (s, 3H), 2.47 (s, 3H), 1.56 (s, 6H). ^{13}C NMR (126 MHz, DMSO) δ 170.54, 160.30, 155.22, 146.24, 143.71, 134.60, 133.36, 127.79, 127.12, 126.22, 69.30, 30.98, 16.61, 13.69. ESI–MS calcd for $C_{18}H_{20}N_3OS_2$ $[M + H]^+$ 358.1042; found 358.1043

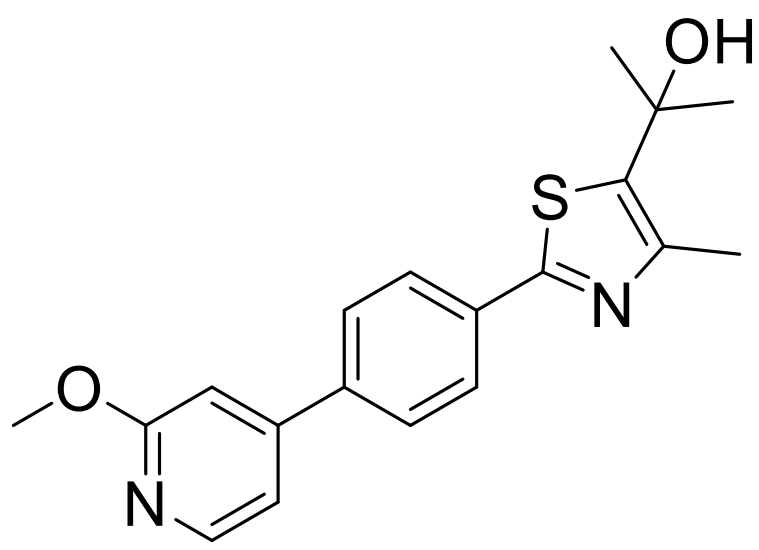

YKJ-244

2-(2-(4-(2-Methoxypyridin-4-yl)phenyl)-4-methylthiazol-5-yl)propan-2-ol

Yield: 93.3% ^{1}H NMR (500 MHz, DMSO) δ 8.25 (d, *J* = 5.4 Hz, 1H), 7.96 (d, *J* = 8.5 Hz, 2H), 7.88 (d, *J* = 8.5 Hz, 2H), 7.36 (dd, *J* = 5.4, 1.6 Hz, 1H), 7.16 (d, *J* = 0.9 Hz, 1H), 5.74 (s, 1H), 3.90 (s, 3H), 2.47 (s, 3H), 1.56 (s, 6H). ^{13}C NMR (126 MHz, DMSO) δ 164.54, 160.24, 149.31, 147.63, 146.29, 143.84, 137.82, 134.08, 127.64, 126.11, 114.91, 107.35, 69.30, 53.32, 30.98, 16.61. ESI–MS calcd for $C_{19}H_{21}N_2O_2S$ $[M + H]^+$ 341.1318; found 341.1315

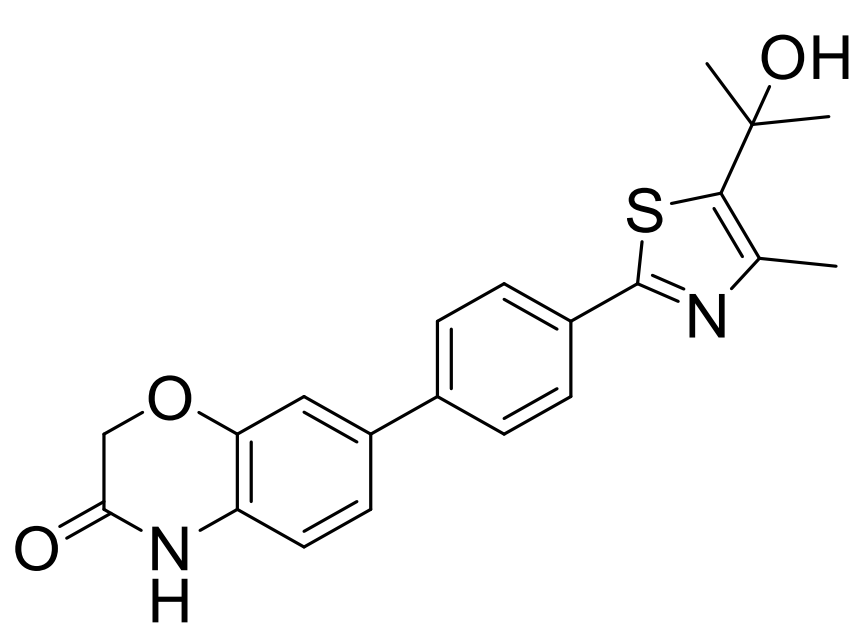

YKJ-245

7-(4-(5-(2-Hydroxypropan-2-yl)-4-methylthiazol-2-yl)phenyl)-2H-benzo[*b*][1,4]oxazin-3(4*H*)-one

Yield: 72.5% ^{1}H NMR (500 MHz, DMSO) δ 10.83 (s, 1H), 7.88 (d, *J* = 8.4 Hz, 2H), 7.72 (d, *J* = 8.5 Hz, 2H), 7.36 – 7.30 (m, 2H), 6.99 (d, *J* = 8.0 Hz, 1H), 5.71 (s, 1H), 4.62 (s, 2H), 2.46 (s, 3H), 1.56 (s, 6H). ^{13}C NMR (126 MHz, DMSO) δ 164.79, 160.65, 146.09, 143.74, 143.14, 140.15,

134.22, 132.20, 127.06, 126.85, 126.02, 120.66, 116.32, 114.11, 69.27, 66.80, 31.04, 16.62. ESI–MS calcd for $C_{21}H_{21}N_2O_3S$ $[M + H]^+$ 381.1267; found 381.1268

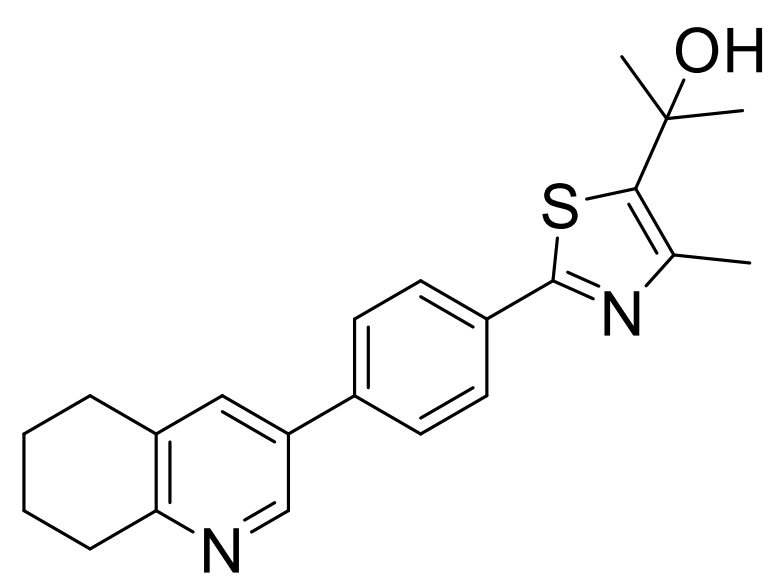

YKJ-246

2-(4-Methyl-2-(4-(5,6,7,8-tetrahydroquinolin-3-yl)phenyl)thiazol-5-yl)propan-2-ol

Yield: 77.1% ^{1}H NMR (500 MHz, DMSO) δ 8.65 (s, 1H), 7.93 (d, *J* = 8.4 Hz, 2H), 7.82 – 7.76 (m, 3H), 5.72 (s, 1H), 2.88 – 2.79 (m, 4H), 2.46 (s, 3H), 1.90 – 1.82 (m, 2H), 1.82 – 1.73 (m, 2H), 1.56 (s, 6H). ^{13}C NMR (126 MHz, DMSO) δ 160.52, 156.25, 146.15, 144.35, 143.34, 138.17, 134.40, 132.71, 132.01, 131.94, 127.17, 126.12, 69.26, 31.77, 31.02, 28.07, 22.64, 22.22, 16.60. ESI–MS calcd for $C_{22}H_{25}N_2OS$ $[M + H]^+$ 365.1682; found 365.1684

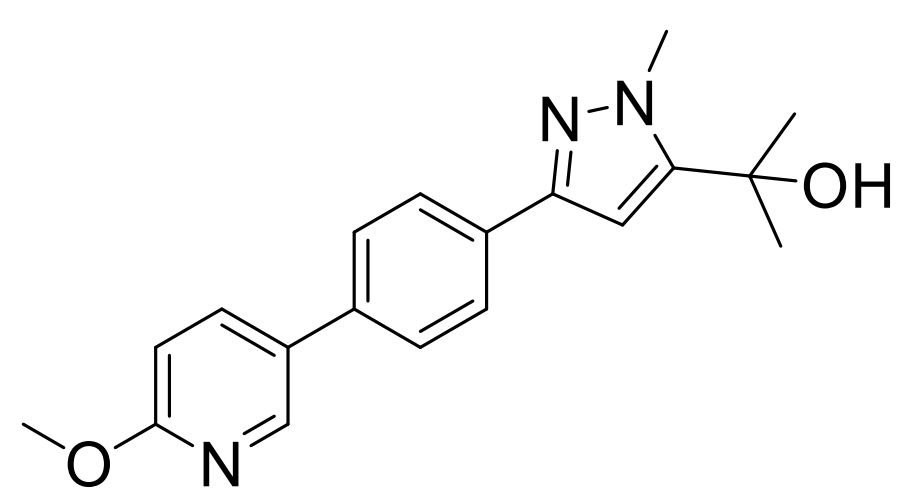

YKJ-247

2-(3-(4-(6-Methoxypyridin-3-yl)phenyl)-1-methyl-1*H*-pyrazol-5-yl)propan-2-ol

Yield: 82.7% ^{1}H NMR (500 MHz, DMSO) δ 8.52 (d, *J* = 2.6 Hz, 1H), 8.05 (dd, *J* = 8.6, 2.6 Hz, 1H), 7.84 (d, *J* = 8.4 Hz, 2H), 7.67 (d, *J* = 8.4 Hz, 2H), 6.91 (d, *J* = 8.6 Hz, 1H), 6.60 (s, 1H), 5.34 (s, 1H), 4.02 (s, 3H), 3.90 (s, 3H), 1.55 (s, 6H). ^{13}C NMR (126 MHz, DMSO) δ 163.01, 150.50, 147.08, 144.46, 137.35, 135.57, 132.62, 128.98, 126.43, 125.40, 110.60, 100.48, 66.91, 53.29, 38.77, 30.23. ESI–MS calcd for $C_{19}H_{22}N_3O_2$ $[M + H]^+$ 324.1707; found 324.1704

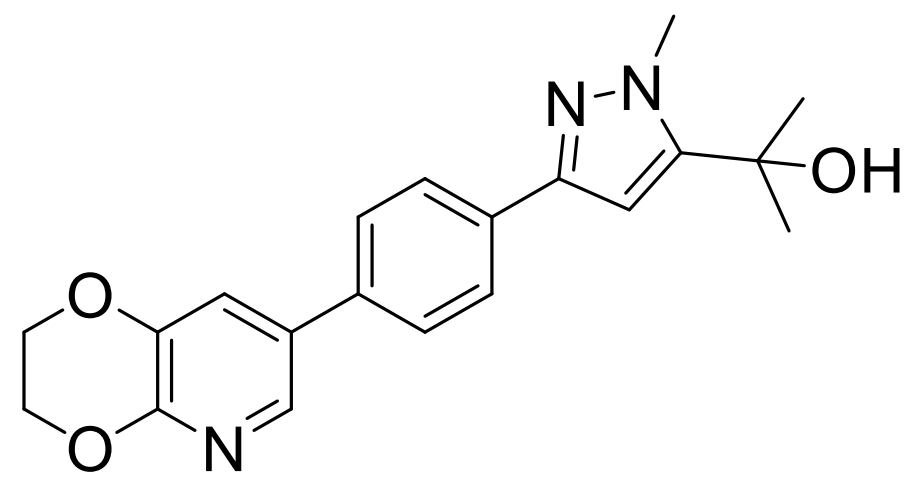

YKJ-248

2-(3-(4-(2,3-Dihydro-[1,4]dioxino[2,3-*b*]pyridin-7-yl)phenyl)-1-methyl-1*H*-pyrazol-5-yl)propan-2-ol

Yield: 77.5% ^{1}H NMR (500 MHz, DMSO) δ 8.10 (d, *J* = 2.1 Hz, 1H), 7.82 (d, *J* = 8.2 Hz, 2H), 7.67 (d, *J* = 8.3 Hz, 2H), 7.62 (d, *J* = 2.2 Hz, 1H), 6.60 (s, 1H), 5.34 (s, 1H), 4.45 – 4.42 (m, 2H), 4.31 – 4.28 (m, 2H), 4.01 (s, 3H), 1.54 (s, 6H). ^{13}C NMR (126 MHz, DMSO) δ 150.50, 150.33, 147.04, 139.18, 136.72, 135.16, 132.79, 130.80, 126.60, 125.35, 122.24, 100.50, 66.91, 64.77, 63.79, 38.77, 30.23. ESI–MS calcd for $C_{20}H_{22}N_3O_3$ $[M + H]^+$ 352.1656; found 352.1656

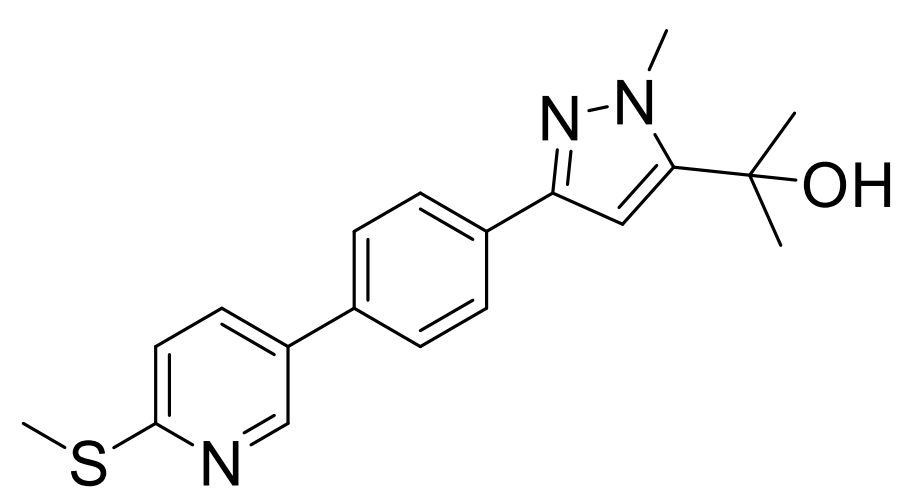

YKJ-249

2-(1-Methyl-3-(4-(6-(methylthio)pyridin-3-yl)phenyl)-1*H*-pyrazol-5-yl)propan-2-ol

Yield: 52.5% ^{1}H NMR (500 MHz, DMSO) δ 8.80 (dd, *J* = 2.5, 0.8 Hz, 1H), 7.98 (dd, *J* = 8.4, 2.5 Hz, 1H), 7.86 (d, *J* = 8.4 Hz, 2H), 7.72 (d, *J* = 8.5 Hz, 2H), 7.38 (dd, *J* = 8.4, 0.8 Hz, 1H), 6.61 (s, 1H), 5.34 (s, 1H), 4.02 (s, 3H), 2.55 (s, 3H), 1.55 (s, 6H). ^{13}C NMR (126 MHz, DMSO) δ 158.14, 150.55, 147.00, 146.96, 135.24, 134.29, 133.09, 131.02, 126.60, 125.46, 121.00, 100.57, 66.92, 38.80, 30.23, 12.84. ESI–MS calcd for $C_{19}H_{22}N_3OS$ $[M + H]^+$ 340.1478; found 340.1480

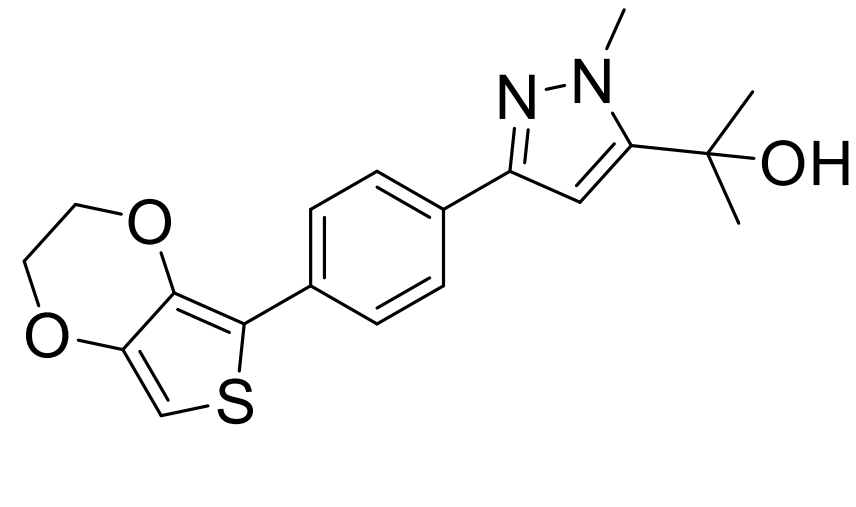

YKJ-250

2-(3-(4-(2,3-Dihydrothieno[3,4-*b*][1,4]dioxin-5-yl)phenyl)-1-methyl-1*H*-pyrazol-5-yl)propan-2-ol

Yield: 68.8% ^{1}H NMR (500 MHz, DMSO) δ 7.76 (d, *J* = 8.5 Hz, 2H), 7.65 (d, *J* = 8.5 Hz, 2H), 6.61 (s, 1H), 6.54 (s, 1H), 5.33 (s, 1H), 4.36 – 4.31 (m, 2H), 4.28 – 4.23 (m, 2H), 4.00 (s, 3H), 1.54 (s, 6H). ^{13}C NMR (126 MHz, DMSO) δ 150.48, 147.13, 142.25, 138.46, 131.72, 131.55, 125.45, 125.14, 115.78, 100.35, 97.90, 66.91, 64.78, 64.12, 38.74, 30.22. ESI–MS calcd for $C_{19}H_{21}N_2O_3S$ $[M + H]^+$ 357.1267; found 357.1276

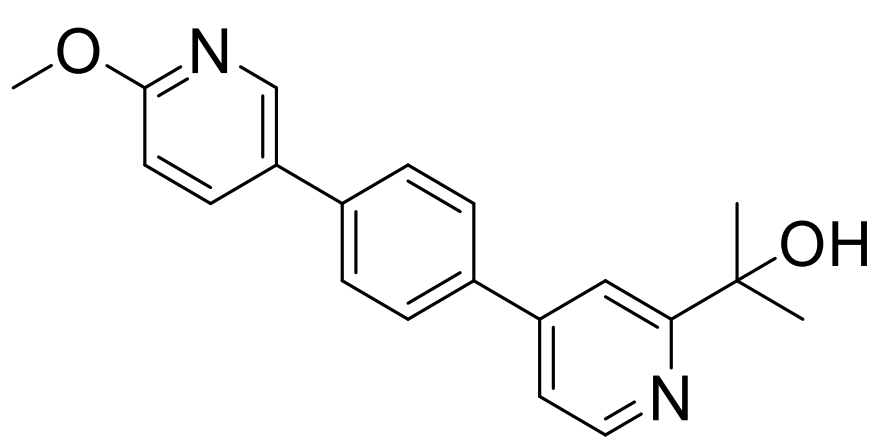

YKJ-251

2-(4-(4-(6-methoxypyridin-3-yl)phenyl)pyridin-2-yl)propan-2-ol

Yield: 65.3% ^{1}H NMR (500 MHz, DMSO) δ 8.58 (d, *J* = 2.0 Hz, 1H), 8.56 (d, *J* = 5.0 Hz, 1H), 8.10 (dd, *J* = 8.6, 2.6 Hz, 1H), 7.98 (d, *J* = 1.0 Hz, 1H), 7.89 – 7.82 (m, 4H), 7.59 (dd, *J* = 5.2, 1.8 Hz, 1H), 6.95 (d, *J* = 8.6 Hz, 1H), 5.32 (s, 1H), 3.91 (s, 3H), 1.49 (s, 6H). ^{13}C NMR (126 MHz, DMSO) δ 168.95, 163.32, 148.77, 146.89, 144.84, 137.63, 137.57, 136.74, 128.49, 127.46 (two peaks overlap), 127.03 (two peaks overlap), 118.83, 115.71, 110.74, 72.59, 53.36, 30.73. ESI–MS calcd for $C_{20}H_{21}N_2O_2$ $[M + H]^+$ 321.1598; found 321.1598

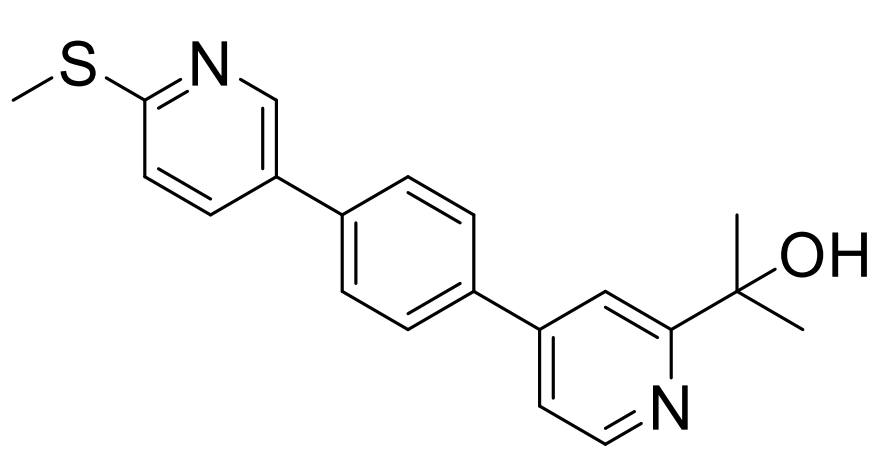

YKJ-252

2-(4-(4-(6-(methylthio)pyridin-3-yl)phenyl)pyridin-2-yl)propan-2-ol

Yield: 49.5% ^{1}H NMR (500 MHz, DMSO) δ 8.87 – 8.83 (m, 1H), 8.57 (d, *J* = 5.0 Hz, 1H), 8.04 (dd, *J* = 8.4, 2.5 Hz, 1H), 7.99 (d, *J* = 1.0 Hz, 1H), 7.91 – 7.87 (m, 4H), 7.59 (dd, *J* = 5.1, 1.8 Hz, 1H), 7.42 (d, *J* = 8.4 Hz, 1H), 5.32 (s, 1H), 2.57 (s, 3H), 1.49 (s, 6H). ^{13}C NMR (126 MHz, DMSO) δ 168.97, 158.78, 148.79, 147.22, 146.81, 137.33, 137.21, 134.52, 130.53, 127.53 (two peaks overlap), 127.19 (two peaks overlap), 121.09, 118.86, 115.73, 72.59, 30.73, 12.85. ESI–MS calcd for $C_{20}H_{21}N_2OS$ $[M + H]^+$ 337.1369; found 337.1372

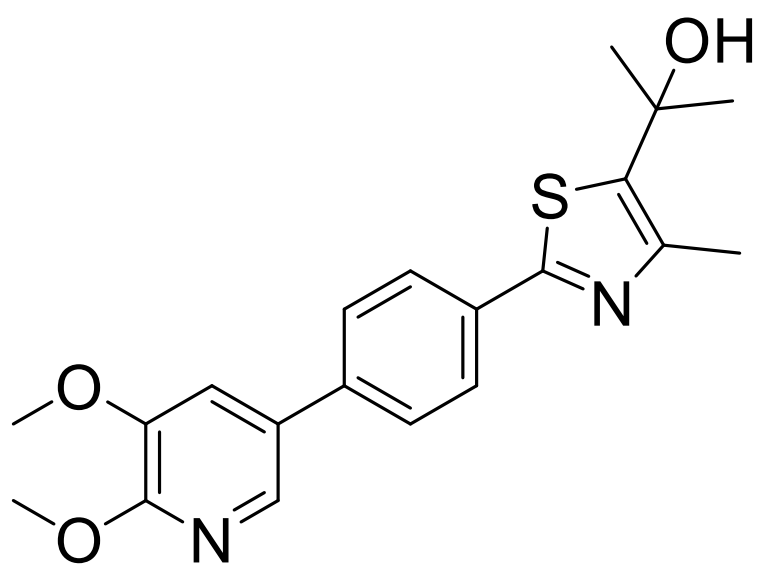

YKJ-253

2-(2-(4-(5,6-dimethoxypyridin-3-yl)phenyl)-4-methylthiazol-5-yl)propan-2-ol

Yield: quantitative ^{1}H NMR (500 MHz, DMSO) δ 8.07 (d, *J* = 2.0 Hz, 1H), 7.91 (d, *J* = 8.4 Hz, 2H), 7.79 (d, *J* = 8.4 Hz, 2H), 7.59 (d, *J* = 2.1 Hz, 1H), 5.72 (s, 1H), 3.91 (s, 3H), 3.90 (s, 3H), 2.46 (s, 3H), 1.56 (s, 6H). ^{13}C NMR (126 MHz, DMSO) δ 160.61, 153.63, 146.10, 143.86, 143.24, 138.21, 134.23, 132.39, 129.04, 127.13 (two peaks overlap), 126.01 (two peaks overlap), 116.50, 69.27, 55.63, 53.23, 31.02, 16.61. ESI–MS calcd for $C_{20}H_{23}N_2O_3S$ $[M + H]^+$ 371.1424; found 371.1430

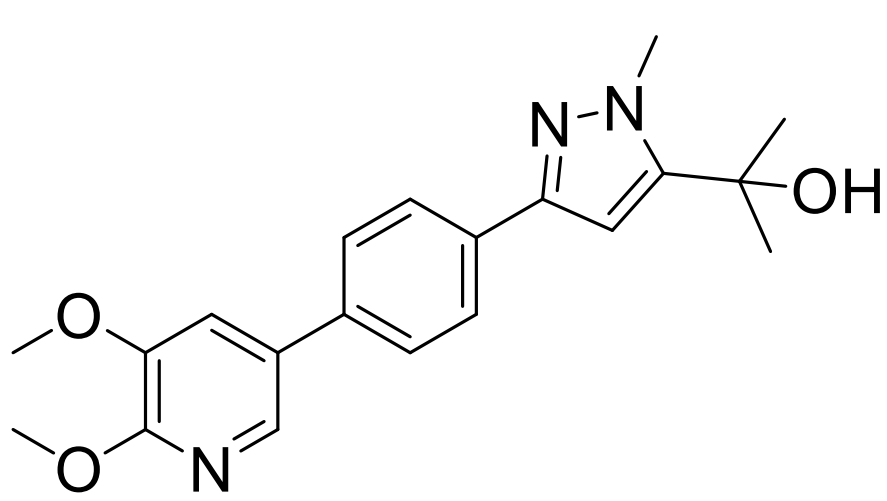

YKJ-254

2-(3-(4-(5,6-dimethoxypyridin-3-yl)phenyl)-1-methyl-1H-pyrazol-5-yl)propan-2-ol

Yield: 90.9% ^{1}H NMR (500 MHz, DMSO) δ 8.03 (d, *J* = 2.0 Hz, 1H), 7.84 (d, *J* = 8.5 Hz, 2H), 7.71 (d, *J* = 8.4 Hz, 2H), 7.56 (d, *J* = 2.0 Hz, 1H), 6.60 (s, 1H), 5.34 (s, 1H), 4.02 (s, 3H), 3.90 (s,

3H), 3.89 (s, 3H), 1.55 (s, 6H). ^{13}C NMR (126 MHz, DMSO) δ 153.32, 150.51, 147.11, 143.80, 135.88, 133.94, 132.64, 129.61, 126.70 (two peaks overlap), 125.30 (two peaks overlap), 116.50, 100.48, 66.93, 55.59, 53.15, 38.77, 30.24. ESI–MS calcd for $C_{20}H_{24}N_3O_3$ $[M + H]^+$ 354.1812; found 354.1816

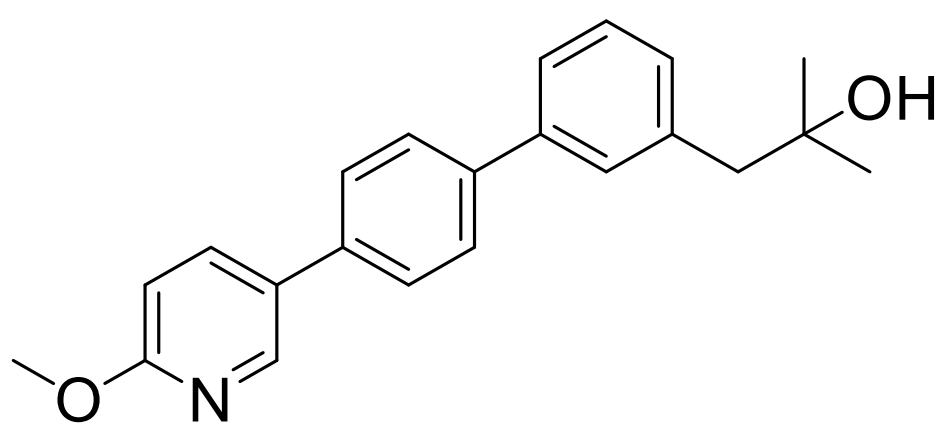

YKJ-255

1-(4'-(6-methoxypyridin-3-yl)-[1,1'-biphenyl]-3-yl)-2-methylpropan-2-ol

Yield: 85.5% ^{1}H NMR (500 MHz, DMSO) δ 8.54 (dd, *J* = 2.6, 0.8 Hz, 1H), 8.07 (dd, *J* = 8.6, 2.6 Hz, 1H), 7.78 – 7.70 (m, 4H), 7.56 – 7.51 (m, 2H), 7.37 (t, *J* = 7.9 Hz, 1H), 7.24 – 7.19 (m, 1H), 6.93 (dd, *J* = 8.6, 0.7 Hz, 1H), 4.36 (s, 1H), 3.91 (s, 3H), 2.74 (s, 2H), 1.10 (s, 6H). ^{13}C NMR (126 MHz, DMSO) δ 163.12, 144.62, 139.63, 139.45, 138.84, 137.46, 135.84, 129.71, 128.83, 128.78, 128.22, 127.28 (two peaks overlap), 126.78 (two peaks overlap), 124.09, 110.67, 69.34, 53.32, 49.41, 29.27. ESI–MS calcd for $C_{22}H_{24}NO_2$ $[M + H]^+$ 334.1802; found 334.1807

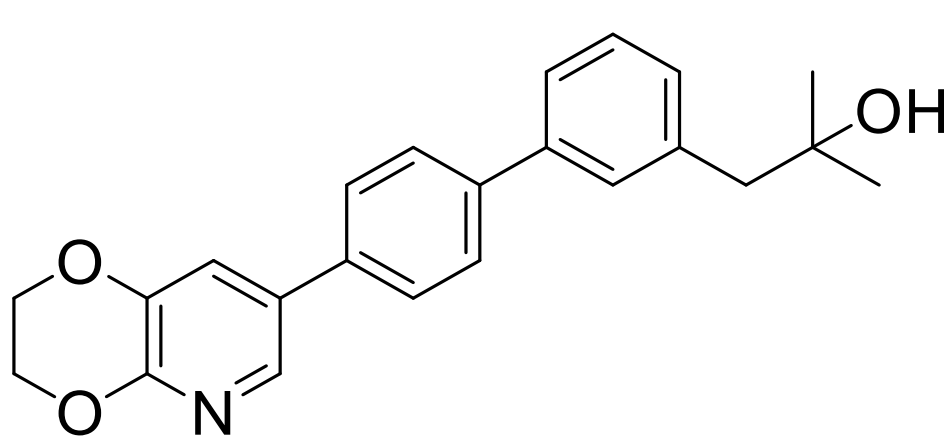

YKJ-256

1-(4'-(2,3-dihydro-[1,4]dioxino[2,3-b]pyridin-7-yl)-[1,1'-biphenyl]-3-yl)-2-methylpropan-2-ol

Yield: 47.3% ^{1}H NMR (500 MHz, DMSO) δ 8.13 (d, *J* = 2.2 Hz, 1H), 7.78 – 7.70 (m, 4H), 7.65 (d, *J* = 2.2 Hz, 1H), 7.55 – 7.51 (m, 2H), 7.36 (t, *J* = 7.9 Hz, 1H), 7.23 – 7.19 (m, 1H), 4.47 – 4.43 (m, 2H), 4.36 (s, 1H), 4.32 – 4.29 (m, 2H), 2.73 (s, 2H), 1.10 (s, 6H). ^{13}C NMR (126 MHz, DMSO) δ 150.47, 139.62, 139.22, 138.80, 136.85, 135.44, 130.64, 129.73, 128.79, 128.21, 127.24 (two

peaks overlap), 126.94 (two peaks overlap), 124.09, 122.37, 69.33, 64.80, 63.81, 49.40, 29.27 (one low field carbon not observed). ESI–MS calcd for $C_{23}H_{24}NO_3$ $[M + H]^+$ 362.1751; found 362.1753

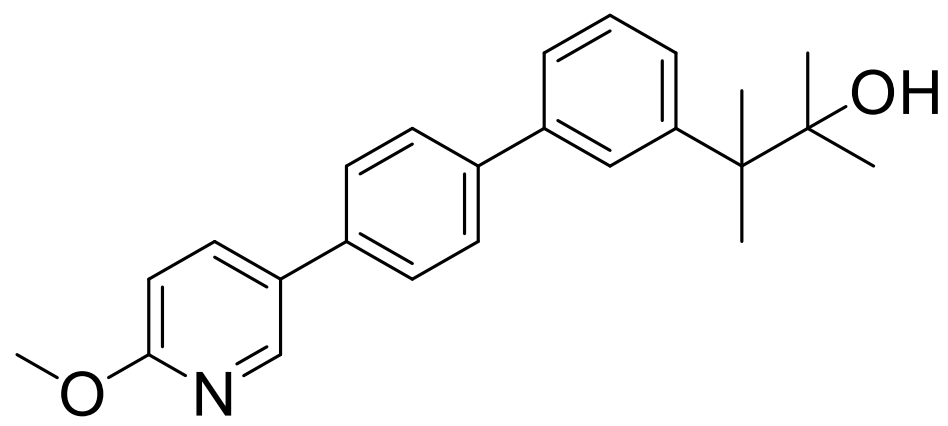

YKJ-257

3-(4'-(6-methoxypyridin-3-yl)-[1,1'-biphenyl]-3-yl)-2,3-dimethylbutan-2-ol

Yield: 80.6% ^{1}H NMR (500 MHz, DMSO) δ 8.54 (dd, *J* = 2.6, 0.8 Hz, 1H), 8.06 (dd, *J* = 8.7, 2.6 Hz, 1H), 7.78 – 7.68 (m, 5H), 7.52 – 7.42 (m, 2H), 7.36 (t, *J* = 7.7 Hz, 1H), 6.93 (dd, *J* = 8.7, 0.8 Hz, 1H), 4.19 (s, 1H), 3.91 (s, 3H), 1.38 (s, 6H), 1.03 (s, 6H). ^{13}C NMR (126 MHz, DMSO) δ 163.11, 148.09, 144.61, 140.04, 138.21, 137.45, 135.73, 128.88, 127.54, 127.46, 127.40 (two peaks overlap), 126.79 (two peaks overlap), 123.68, 110.67, 72.84, 53.31, 44.75, 26.21, 24.45 (one low field carbon not observed). ESI–MS calcd for $C_{24}H_{28}NO_2$ $[M + H]^+$ 362.2115; found 362.2125

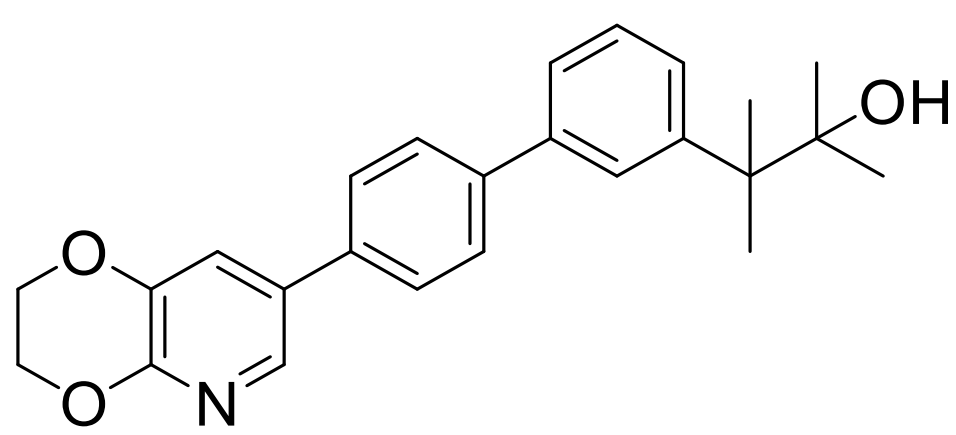

YKJ-258

3-(4'-(2,3-dihydro-[1,4]dioxino[2,3-b]pyridin-7-yl)-[1,1'-biphenyl]-3-yl)-2,3-dimethylbutan-2-ol

Yield: 46.1% ^{1}H NMR (500 MHz, DMSO) δ 8.12 (d, *J* = 2.2 Hz, 1H), 7.78 – 7.67 (m, 5H), 7.64 (d, *J* = 2.2 Hz, 1H), 7.51 – 7.46 (m, 1H), 7.46 – 7.42 (m, 1H), 7.35 (t, *J* = 7.7 Hz, 1H), 4.47 – 4.42 (m, 2H), 4.33 – 4.29 (m, 2H), 4.19 (s, 1H), 1.38 (s, 6H), 1.03 (s, 6H). ^{13}C NMR (126 MHz, DMSO) δ 150.46, 148.09, 140.22, 139.22, 138.18, 136.85, 135.34, 130.70, 127.54, 127.36 (two peaks overlap), 126.95(two peaks overlap), 126.80, 123.69, 122.38, 72.84, 64.80, 63.81, 44.75, 26.21, 24.45 (one low field carbon not observed). ESI–MS calcd for $C_{25}H_{28}NO_3$ $[M + H]^+$ 390.2064; found 390.2066

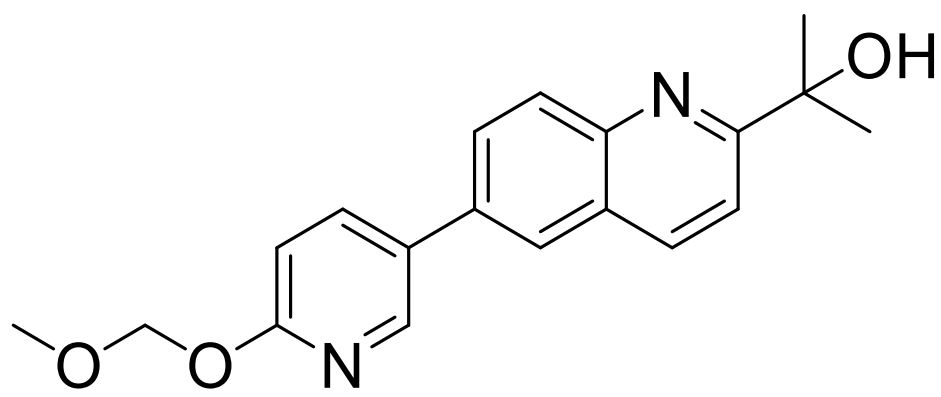

YKJ-259

2-(6-(6-(methoxymethoxy)pyridin-3-yl)quinolin-2-yl)propan-2-ol

Yield: 46.9% ^{1}H NMR (500 MHz, DMSO) δ 8.33 (d, *J* = 8.7 Hz, 1H), 8.28 (d, *J* = 2.9 Hz, 1H), 8.16 (t, *J* = 1.4 Hz, 1H), 8.05 (dd, *J* = 9.6, 2.8 Hz, 1H), 8.00 (d, *J* = 1.4 Hz, 2H), 7.87 (d, *J* = 8.6 Hz, 1H), 6.60 (d, *J* = 9.5 Hz, 1H), 5.44 (s, 1H), 5.35 (s, 2H), 3.34 (s, 27H), 1.54 (s, 6H). ^{13}C NMR (126 MHz, DMSO) δ 168.20, 161.07, 145.35, 139.85, 136.47, 136.24, 133.10, 129.29, 127.16, 126.88, 123.27, 120.57, 118.37, 117.24, 78.40, 72.69, 56.43, 30.48. ESI–MS calcd for $C_{19}H_{21}N_2O_3$ $[M + H]^+$ 325.1547; found 325.1547

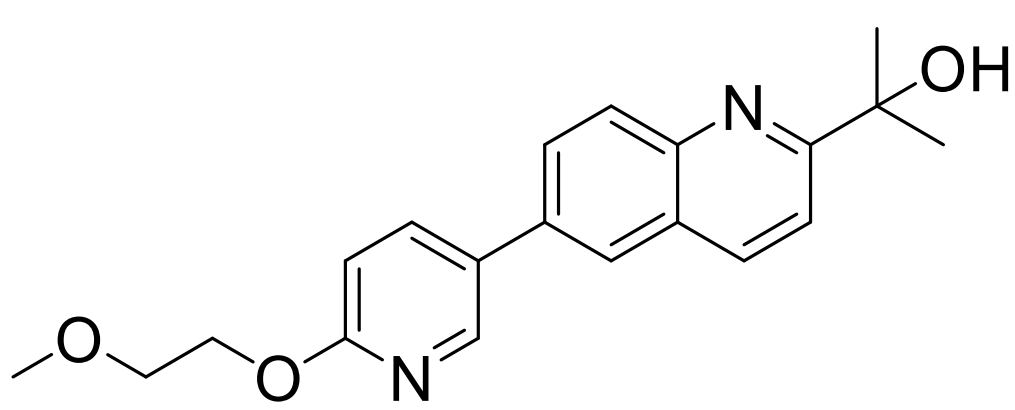

YKJ-260

2-(6-(6-(2-methoxyethoxy)pyridin-3-yl)quinolin-2-yl)propan-2-ol

Yield: 36.2% ^{1}H NMR (500 MHz, DMSO) δ 8.63 (d, *J* = 2.6 Hz, 1H), 8.37 (d, *J* = 8.6 Hz, 1H), 8.25 (d, *J* = 2.0 Hz, 1H), 8.18 (dd, *J* = 8.7, 2.6 Hz, 1H), 8.09 – 8.00 (m, 2H), 7.89 (d, *J* = 8.7 Hz, 1H), 6.98 (d, *J* = 8.5 Hz, 1H), 5.46 (s, 1H), 4.47 – 4.42 (m, 2H), 3.72 – 3.67 (m, 2H), 3.32 (s, 3H), 1.55 (s, 6H). ^{13}C NMR (126 MHz, DMSO) δ 168.40, 162.82, 145.56, 144.99, 137.90, 136.66, 134.31, 129.34, 128.62, 128.04, 126.90, 124.58, 118.32, 110.96, 72.70, 70.26, 64.83, 58.13, 30.49. ESI–MS calcd for $C_{20}H_{23}N_2O_3$ $[M + H]^+$ 339.1703; found 339.1704

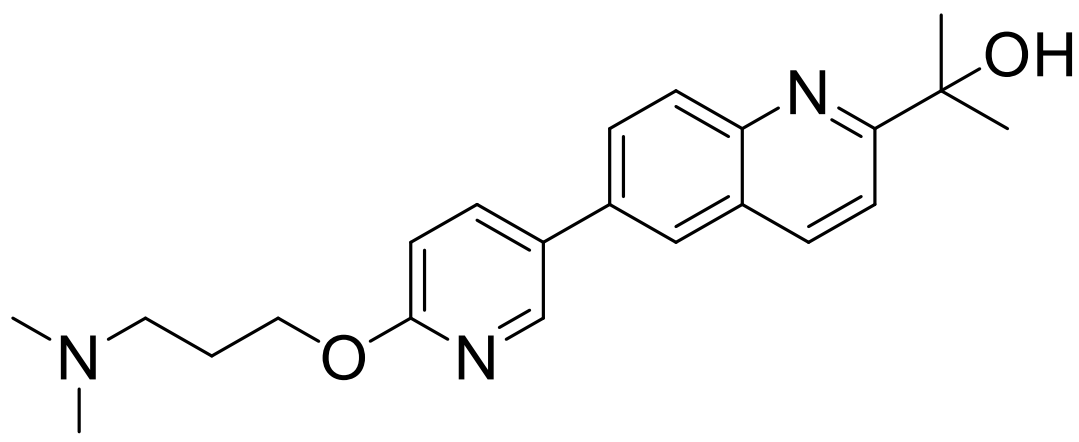

YKJ-261

2-(6-(6-(3-(dimethylamino)propoxy)pyridin-3-yl)quinolin-2-yl)propan-2-ol

Yield: 39.1% ^{1}H NMR (500 MHz, DMSO) δ 8.63 (d, *J* = 2.5 Hz, 1H), 8.37 (d, *J* = 8.7 Hz, 1H), 8.24 (d, *J* = 2.0 Hz, 1H), 8.18 (dd, *J* = 8.6, 2.6 Hz, 1H), 8.09 – 8.00 (m, 2H), 7.89 (d, *J* = 8.7 Hz, 1H), 6.95 (d, *J* = 8.6 Hz, 1H), 5.47 (s, 1H), 4.36 (t, *J* = 6.5 Hz, 2H), 2.63 – 2.55 (m, 2H), 2.33 (s, 6H), 1.99 – 1.91 (m, 2H), 1.55 (s, 6H). ^{13}C NMR (126 MHz, DMSO) δ 168.42, 162.98, 145.58, 145.09, 137.86, 136.70, 134.36, 129.38, 128.58, 128.07, 126.94, 124.56, 118.35, 110.94, 72.74, 63.86, 55.40, 44.48, 30.51, 25.99. ESI–MS calcd for $C_{22}H_{28}N_3O_2$ $[M + H]^+$ 366.2176; found 366.2176

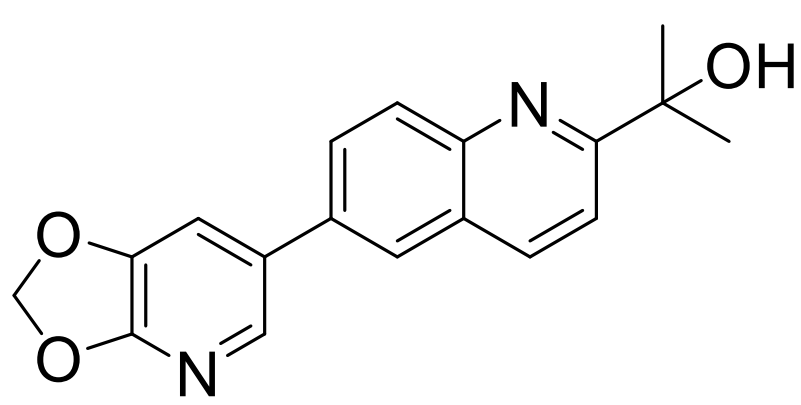

YKJ-262

2-(6-([1,3]dioxolo[4,5-b]pyridin-6-yl)quinolin-2-yl)propan-2-ol

Yield: 52.5% ^{1}H NMR (500 MHz, DMSO) δ 8.36 (d, *J* = 8.5 Hz, 1H), 8.24 (d, *J* = 2.0 Hz, 1H), 8.08 (d, *J* = 1.9 Hz, 1H), 8.05 – 8.00 (m, 2H), 7.89 (d, *J* = 8.6 Hz, 1H), 7.75 (d, *J* = 2.0 Hz, 1H), 6.23 (s, 2H), 5.46 (s, 1H), 1.55 (s, 6H). ^{13}C NMR (126 MHz, DMSO) δ 168.51, 157.84, 145.65, 140.54, 136.98, 136.68, 134.38, 130.15, 129.26, 128.28, 126.82, 125.03, 118.35, 113.41, 100.69, 72.71, 30.48. ESI–MS calcd for $C_{18}H_{17}N_2O_3$ $[M + H]^+$ 309.1234; found 309.1238

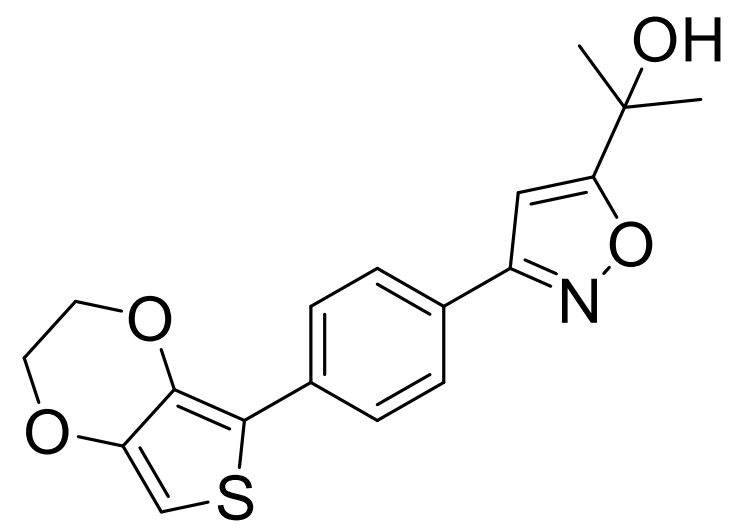

YKJ-263

2-(3-(4-(2,3-dihydrothieno[3,4-b][1,4]dioxin-5-yl)phenyl)isoxazol-5-yl)propan-2-ol

Yield: 55.7% ^{1}H NMR (500 MHz, DMSO) δ 7.88 (d, *J* = 8.3 Hz, 2H), 7.76 (d, *J* = 8.3 Hz, 2H), 6.85 (s, 1H), 6.70 (s, 1H), 5.68 (s, 1H), 4.39 – 4.34 (m, 2H), 4.29 – 4.24 (m, 2H), 1.52 (s, 6H). ^{13}C NMR (126 MHz, DMSO) δ 179.80, 161.07, 142.31, 139.30, 134.38, 127.02 (two peaks overlap), 126.63, 125.62 (two peaks overlap), 115.06, 99.02, 97.62, 67.44, 64.89, 64.11, 29.03. ESI–MS calcd for $C_{18}H_{18}NO_4S$ $[M + H]^+$ 344.0951; found 344.0950

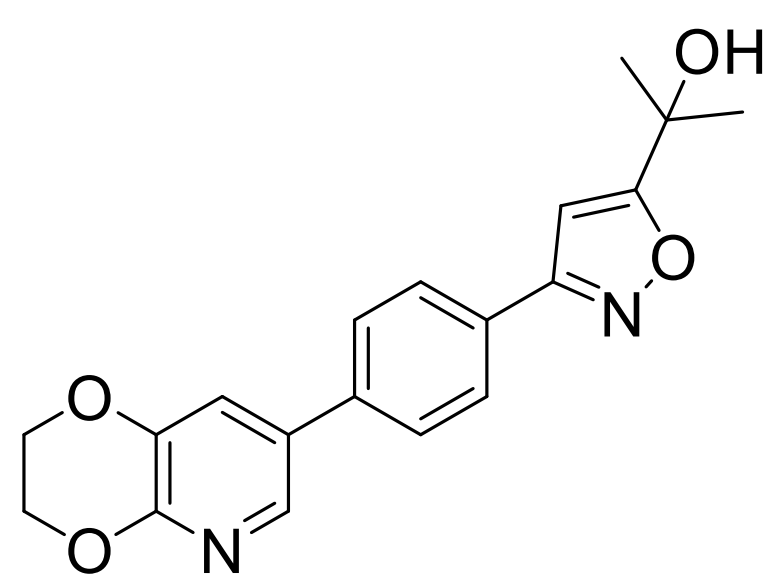

YKJ-264

2-(3-(4-(2,3-dihydro-[1,4]dioxino[2,3-b]pyridin-7-yl)phenyl)isoxazol-5-yl)propan-2-ol

Yield: 54.7% ^{1}H NMR (500 MHz, DMSO) δ 8.15 (d, *J* = 2.2 Hz, 1H), 7.94 (d, *J* = 8.4 Hz, 2H), 7.81 (d, *J* = 8.5 Hz, 2H), 7.69 (d, *J* = 2.3 Hz, 1H), 6.92 (s, 1H), 5.69 (s, 1H), 4.48 – 4.43 (m, 2H), 4.33 – 4.28 (m, 2H), 1.53 (s, 6H). ^{13}C NMR (126 MHz, DMSO) δ 179.83, 161.09, 150.76, 139.25, 138.10, 137.06, 130.22, 127.92, 127.15 (two peaks overlap), 127.04 (two peaks overlap), 122.51, 97.74, 67.41, 64.84, 63.80, 29.03. ESI–MS calcd for $C_{19}H_{19}N_2O_4$ $[M + H]^+$ 339.1339; found 339.1336

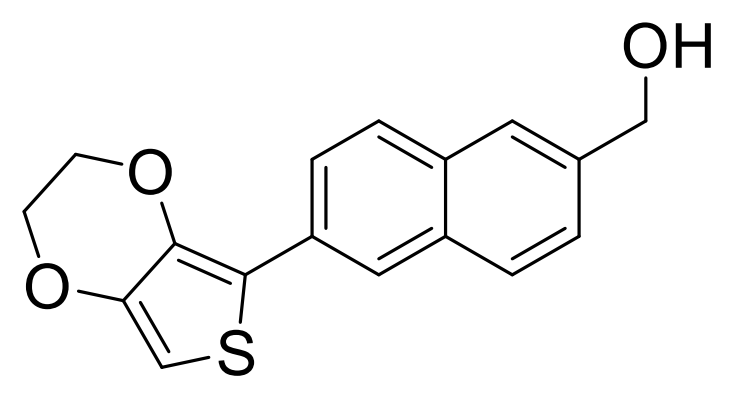

YKJ-265

(6-(2,3-dihydrothieno[3,4-b][1,4]dioxin-5-yl)naphthalen-2-yl)methanol

Yield: 62.7% ^{1}H NMR (500 MHz, DMSO) δ 8.14 (d, *J* = 1.9 Hz, 1H), 7.92 – 7.84 (m, 2H), 7.82 – 7.76 (m, 2H), 7.46 (dd, *J* = 8.4, 1.7 Hz, 1H), 6.67 (s, 1H), 5.33 (t, *J* = 5.7 Hz, 1H), 4.65 (d, *J* = 5.7 Hz, 2H), 4.41 – 4.35 (m, 2H), 4.31 – 4.25 (m, 2H). ^{13}C NMR (126 MHz, DMSO) δ 142.34, 140.25, 138.77, 132.28, 131.55, 129.93, 128.16, 127.68, 125.97, 124.19, 124.08, 123.20, 115.94, 98.34, 64.85, 64.16, 62.97. ESI–MS calcd for $C_{17}H_{15}O_3S$ $[M + H]^+$ 299.0736; found 299.0741

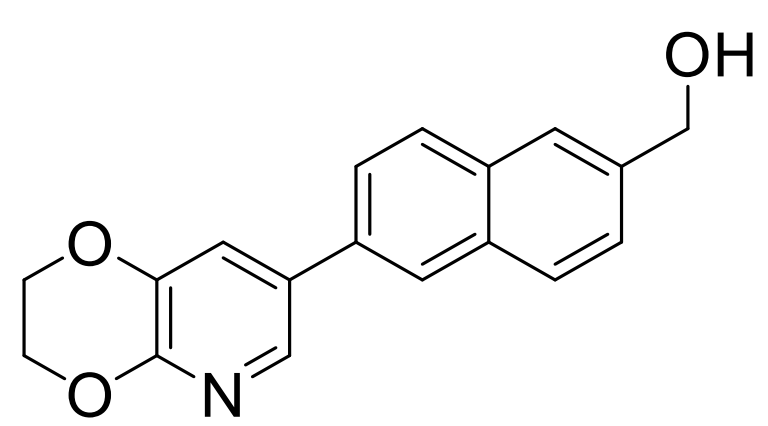

YKJ-266

(6-(2,3-dihydro-[1,4]dioxino[2,3-b]pyridin-7-yl)naphthalen-2-yl)methanol

Yield: 62.4% ^{1}H NMR (500 MHz, DMSO) δ 8.24 – 8.17 (m, 2H), 7.96 (d, *J* = 8.6 Hz, 1H), 7.92 (d, *J* = 8.5 Hz, 1H), 7.86 – 7.79 (m, 2H), 7.74 (d, *J* = 2.2 Hz, 1H), 7.49 (d, *J* = 8.5 Hz, 1H), 5.35 (t, *J* = 5.7 Hz, 1H), 4.67 (d, *J* = 5.1 Hz, 2H), 4.49 – 4.43 (m, 2H), 4.35 – 4.29 (m, 2H). ^{13}C NMR (126 MHz, DMSO) δ 150.45, 140.55, 139.27, 137.16, 133.46, 132.44, 132.17, 131.03, 128.42, 127.96, 125.78, 124.84, 124.81, 124.05, 122.65, 64.82, 63.83, 62.98. ESI–MS calcd for $C_{18}H_{16}NO_3$ $[M + H]^+$ 294.1125; found 294.1132

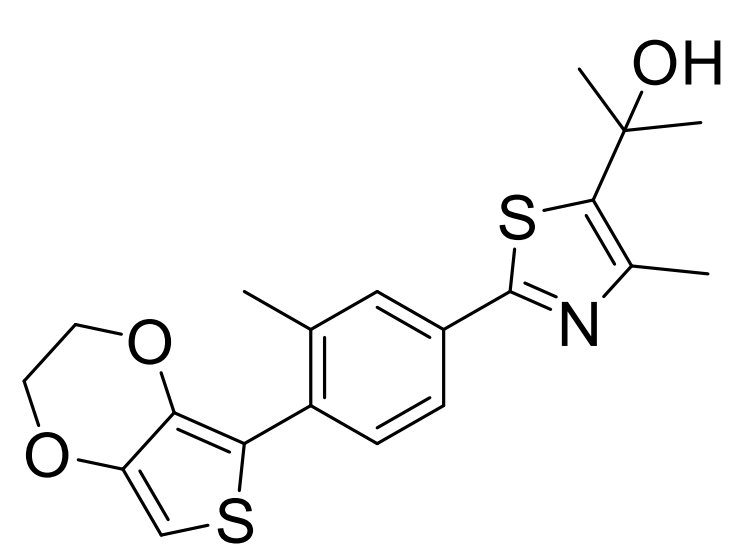

YKJ-267

2-(2-(4-(2,3-dihydrothieno[3,4-b][1,4]dioxin-5-yl)-3-methylphenyl)-4-methylthiazol-5-yl)propan-2-ol

Yield: 53.9% ^{1}H NMR (500 MHz, DMSO-d_6) δ 7.77 (d, *J* = 1.9 Hz, 1H), 7.67 (dd, *J* = 8.0, 1.9 Hz, 1H), 7.34 (d, *J* = 8.0 Hz, 1H), 6.71 (s, 1H), 5.69 (s, 1H), 4.23 (s, 4H), 2.46 (s, 3H), 2.35 (s, 3H), 1.56 (s, 6H). ^{13}C NMR (126 MHz, DMSO-d_6) δ 160.58, 146.14, 143.19, 141.38, 138.03, 137.75, 132.78, 132.76, 131.30, 127.12, 122.91, 114.41, 99.20, 69.23, 64.43, 64.17, 31.04, 20.14, 16.59. ESI–MS calcd for $C_{20}H_{22}NO_3S_2$ $[M + H]^+$ 388.1036 found 388.1044

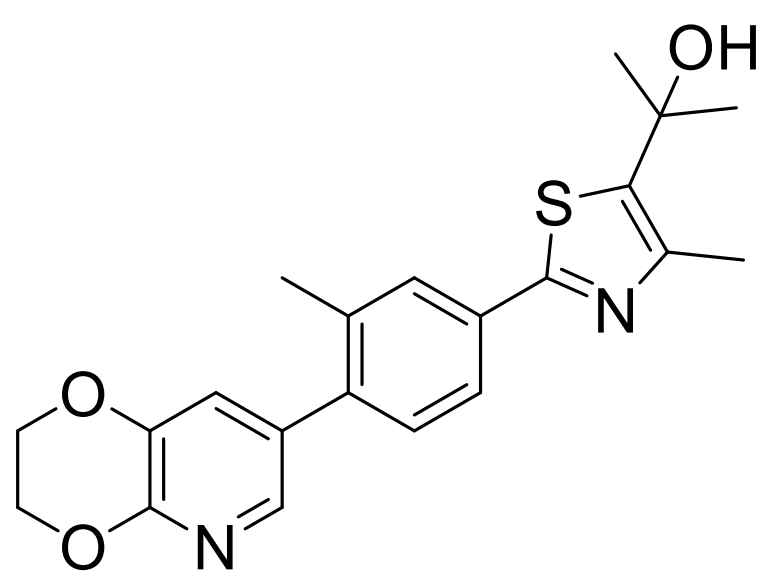

YKJ-268

2-(2-(4-(2,3-dihydro-[1,4]dioxino[2,3-b]pyridin-7-yl)-3-methylphenyl)-4-methylthiazol-5-yl)propan-2-ol

Yield: 30.5% ^{1}H NMR (500 MHz, DMSO-d_6) δ 7.79 (d, *J* = 1.8 Hz, 1H), 7.75 (d, *J* = 2.1 Hz, 1H), 7.72 – 7.69 (m, 1H), 7.37 (d, *J* = 2.2 Hz, 1H), 7.29 (d, *J* = 8.0 Hz, 1H), 5.70 (s, 1H), 4.48 – 4.42 (m, 2H), 4.33 – 4.28 (m, 2H), 2.46 (s, 3H), 2.31 (s, 3H), 1.56 (s, 6H). ^{13}C NMR (126 MHz, DMSO-d_6) δ 160.68, 150.12, 146.11, 143.12, 138.70, 138.59, 138.34, 136.25, 132.73, 131.24, 130.56, 127.35, 124.90, 123.25, 69.24, 64.75, 63.77, 31.06, 20.16, 16.61. ESI–MS calcd for $C_{21}H_{23}N_2O_3S$ $[M + H]^+$ 383.1424 found 383.1430

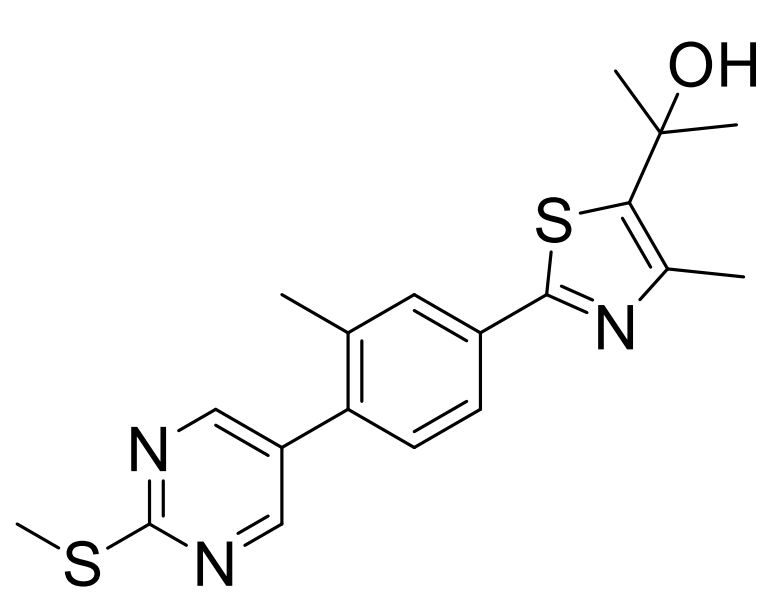

YKJ-269

2-(4-methyl-2-(3-methyl-4-(2-(methylthio)pyrimidin-5-yl)phenyl)thiazol-5-yl)propan-2-ol

Yield: 86.6% ^{1}H NMR (500 MHz, DMSO-d_6) δ 8.74 (s, 2H), 7.84 (d, J = 1.9 Hz, 1H), 7.76 (dd, J = 7.9, 2.0 Hz, 1H), 7.39 (d, J = 8.0 Hz, 1H), 5.71 (s, 1H), 2.57 (s, 3H), 2.47 (s, 3H), 2.34 (s, 3H), 1.56 (s, 6H). ^{13}C NMR (126 MHz, DMSO-d_6) δ 170.15, 160.45, 157.00, 146.21, 143.44, 136.63, 135.19, 133.45, 130.67, 129.16, 127.46, 123.46, 69.25, 31.03, 20.00, 16.60, 13.65. ESI–MS calcd for $C_{19}H_{22}N_3OS_2$ $[M + H]^+$ 372.1199 found 372.1200

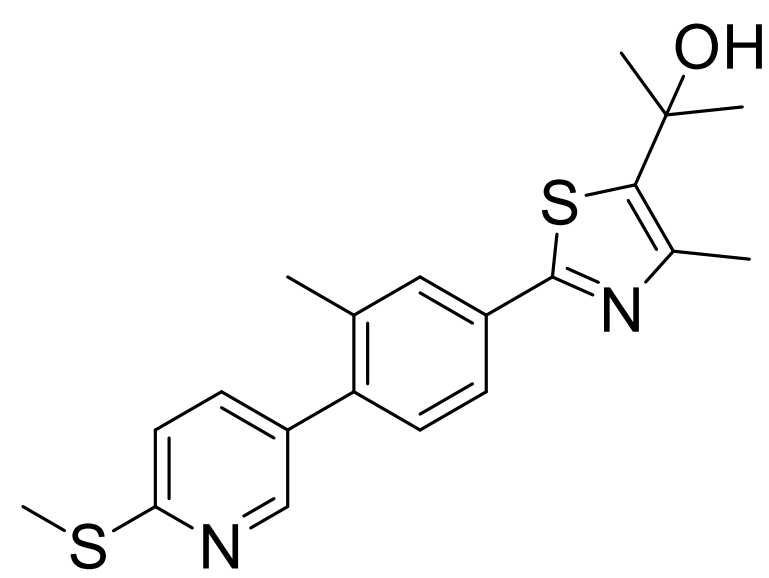

YKJ-270

2-(4-methyl-2-(3-methyl-4-(6-(methylthio)pyridin-3-yl)phenyl)thiazol-5-yl)propan-2-ol

Yield: quantitative ^{1}H NMR (500 MHz, DMSO-d_6) δ 8.47 (d, J = 2.3 Hz, 1H), 7.81 (s, 1H), 7.77 – 7.69 (m, 2H), 7.39 (d, J = 8.2 Hz, 1H), 7.32 (d, J = 7.9 Hz, 1H), 5.70 (s, 1H), 2.56 (s, 3H), 2.46 (s, 3H), 2.32 (s, 3H), 1.56 (s, 6H). ^{13}C NMR (126 MHz, DMSO-d_6) δ 160.62, 158.16, 148.85, 146.14, 143.19, 138.51, 136.86, 136.24, 132.89, 131.75, 130.51, 127.42, 123.37, 120.45, 69.24, 31.05, 20.13, 16.60, 12.78. ESI–MS calcd for $C_{20}H_{23}N_2OS_2$ $[M + H]^+$ 371.1246 found 371.1255

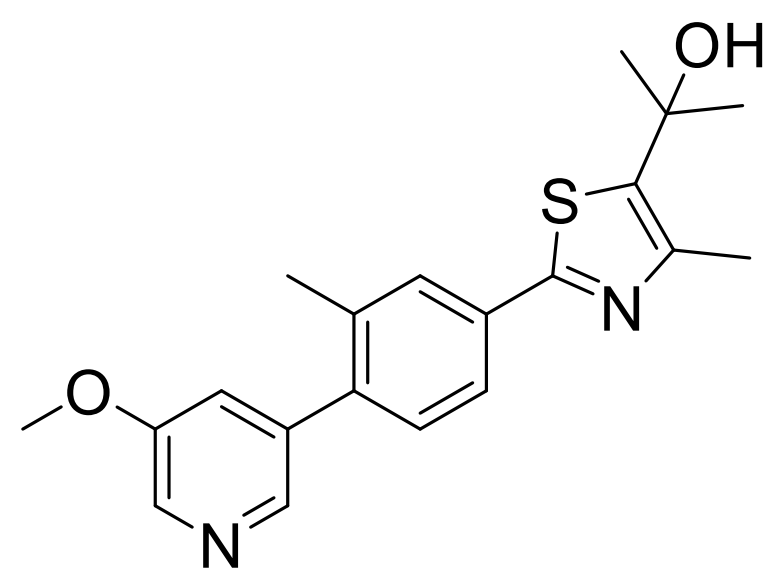

YKJ-271

2-(2-(4-(5-methoxypyridin-3-yl)-3-methylphenyl)-4-methylthiazol-5-yl)propan-2-ol

Yield: quantitative ^{1}H NMR (500 MHz, DMSO-d_6) δ 8.23 (dd, J = 5.3, 0.7 Hz, 1H), 7.81 (d, J = 1.8 Hz, 1H), 7.74 (dd, J = 8.0, 1.9 Hz, 1H), 7.31 (d, J = 7.9 Hz, 1H), 7.02 (dd, J = 5.2, 1.5 Hz, 1H), 6.84 – 6.80 (m, 1H), 5.71 (s, 1H), 3.90 (s, 3H), 2.46 (s, 3H), 2.31 (s, 3H), 1.56 (s, 6H). ^{13}C NMR (126 MHz, DMSO-d_6) δ 163.80, 160.49, 151.25, 146.81, 146.18, 143.37, 139.64, 135.76, 133.30, 129.95, 127.43, 123.30, 117.73, 110.25, 69.25, 53.22, 31.04, 19.97, 16.60. ESI–MS calcd for $C_{20}H_{23}N_2O_2S$ $[M + H]^+$ 355.1475 found 355.1480

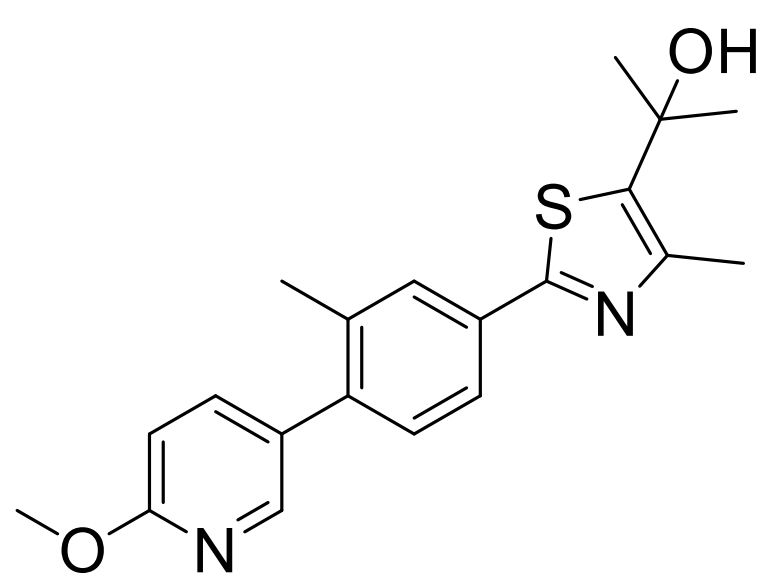

YKJ-272

2-(2-(4-(6-methoxypyridin-3-yl)-3-methylphenyl)-4-methylthiazol-5-yl)propan-2-ol

Yield: 98.6% ^{1}H NMR (500 MHz, DMSO-d_6) δ 8.19 (dd, J = 2.5, 0.8 Hz, 1H), 7.80 (d, J = 1.9 Hz, 1H), 7.77 (dd, J = 8.5, 2.5 Hz, 1H), 7.72 (dd, J = 8.0, 2.0 Hz, 1H), 7.30 (d, J = 7.9 Hz, 1H), 6.91 (dd, J = 8.5, 0.8 Hz, 1H), 5.70 (s, 1H), 3.90 (s, 3H), 2.46 (s, 3H), 2.30 (s, 3H), 1.56 (s, 6H). ^{13}C NMR (126 MHz, DMSO-d_6) δ 162.73, 160.71, 146.37, 146.10, 143.08, 139.76, 138.79, 136.23, 132.66, 130.52, 129.51, 127.36, 123.30, 110.01, 69.23, 53.25, 31.06, 20.17, 16.61. ESI–MS calcd for $C_{20}H_{23}N_2O_2S$ $[M + H]^+$ 355.1475 found 355.1476

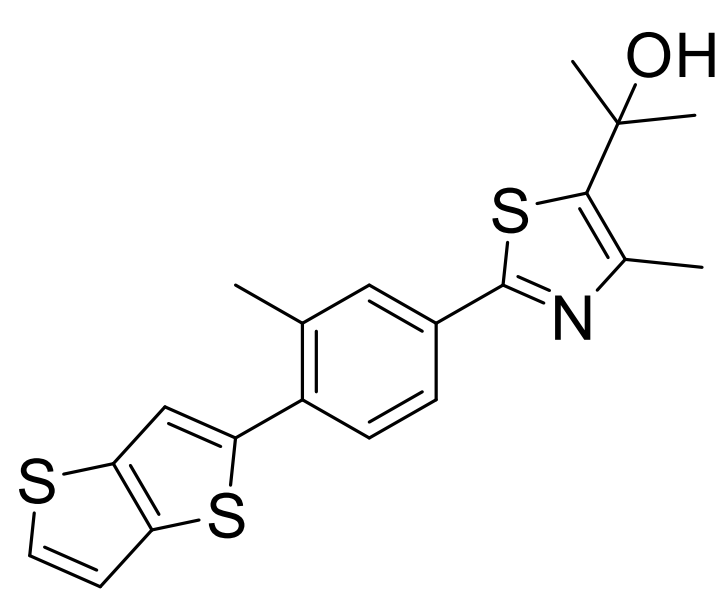

YKJ-273

2-(4-methyl-2-(3-methyl-4-(thieno[3,2-b]thiophen-2-yl)phenyl)thiazol-5-yl)propan-2-ol

Yield: 65.3% ^{1}H NMR (500 MHz, DMSO-d_6) δ 7.84 (d, J = 1.8 Hz, 1H), 7.74 (dd, J = 8.0, 2.0 Hz, 1H), 7.70 (d, J = 5.3 Hz, 1H), 7.64 (s, 1H), 7.53 (d, J = 8.1 Hz, 1H), 7.48 (d, J = 5.3 Hz, 1H), 5.71 (s, 1H), 2.52 (s, 3H), 2.47 (s, 3H), 1.57 (s, 6H). ^{13}C NMR (126 MHz, DMSO-d_6) δ 160.40, 146.22, 143.41, 143.21, 139.28, 138.72, 136.28, 134.82, 132.84, 130.59, 128.32, 127.88, 123.43, 119.95, 119.85, 69.26, 31.04, 21.05, 16.60. ESI–MS calcd for $C_{20}H_{20}NOS_3$ [M + H]$^+$ 386.0702 found 386.0706

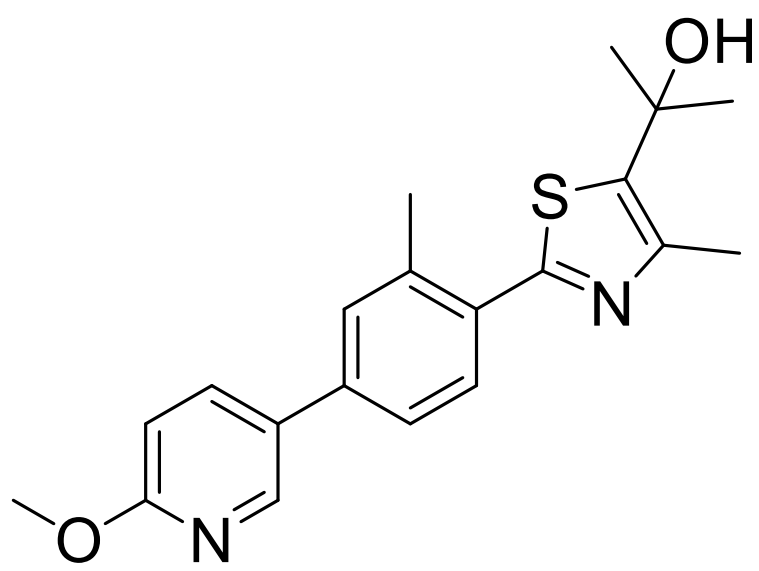

YKJ-274

2-(2-(4-(6-methoxypyridin-3-yl)-2-methylphenyl)-4-methylthiazol-5-yl)propan-2-ol

Yield: 84.1% ^{1}H NMR (500 MHz, DMSO-d_6) δ 8.55 (d, J = 2.2 Hz, 1H), 8.07 (dd, J = 8.6, 2.6 Hz, 1H), 7.80 (d, J = 8.1 Hz, 1H), 7.65 (d, J = 2.1 Hz, 1H), 7.58 (dd, J = 8.1, 2.1 Hz, 1H), 6.93 (d, J = 8.6 Hz, 1H), 5.69 (s, 1H), 3.90 (s, 3H), 2.62 (s, 3H), 2.48 (s, 3H), 1.57 (s, 6H). ^{13}C NMR (126 MHz, DMSO-d_6) δ 163.27, 160.46, 145.44, 144.79, 143.25, 137.50, 137.25, 136.06, 131.61, 129.64, 129.28, 128.41, 124.02, 110.66, 69.27, 53.33, 31.12, 21.60, 16.65. ESI–MS calcd for $C_{20}H_{23}N_2O_2S$ [M + H]$^+$ 355.1475 found 355.1482

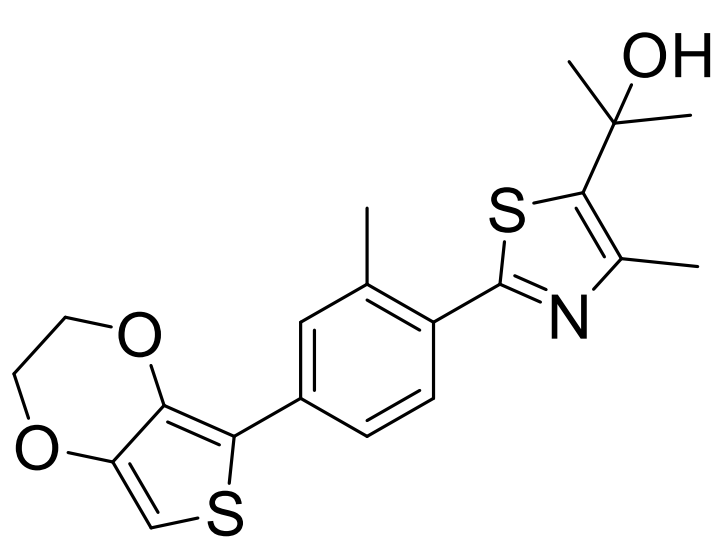

YKJ-275

2-(2-(4-(2,3-dihydrothieno[3,4-b][1,4]dioxin-5-yl)-2-methylphenyl)-4-methylthiazol-5-yl)propan-2-ol

Yield: 80.0% ^{1}H NMR (500 MHz, DMSO-d_6) δ 7.74 (d, J = 8.9 Hz, 1H), 7.60 – 7.56 (m, 2H), 6.68 (s, 1H), 5.67 (s, 1H), 4.38 – 4.33 (m, 2H), 4.29 – 4.23 (m, 2H), 2.57 (s, 3H), 2.47 (s, 3H), 1.57 (s, 6H). ^{13}C NMR (126 MHz, DMSO-d_6) δ 160.49, 145.41, 143.08, 142.29, 139.17, 135.73, 133.21, 130.65, 129.46, 127.86, 123.07, 115.08, 98.82, 69.26, 64.85, 64.10, 31.13, 21.68, 16.63. ESI–MS calcd for $C_{20}H_{22}NO_3S_2$ $[M + H]^+$ 388.1036 found 388.1043

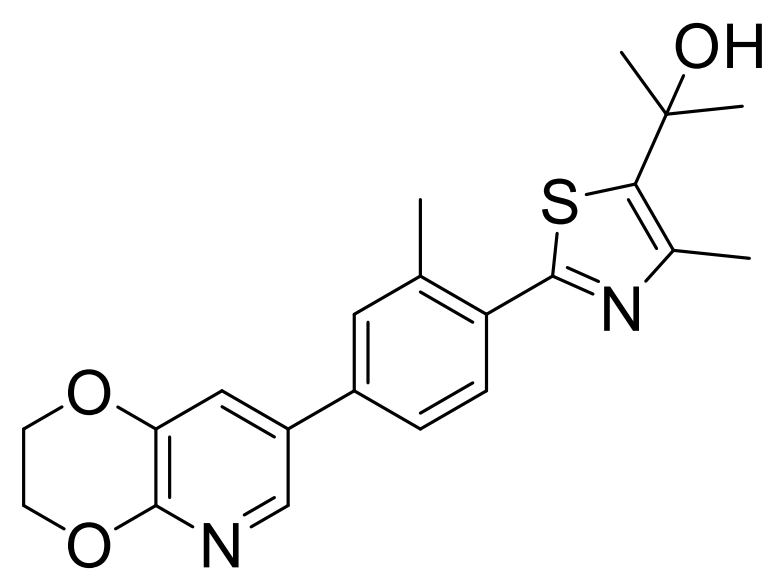

YKJ-276

2-(2-(4-(2,3-dihydro-[1,4]dioxino[2,3-b]pyridin-7-yl)-2-methylphenyl)-4-methylthiazol-5-yl)propan-2-ol

Yield: 61.6% ^{1}H NMR (500 MHz, DMSO-d_6) δ 8.14 (d, J = 2.1 Hz, 1H), 7.78 (d, J = 8.1 Hz, 1H), 7.68 – 7.64 (m, 2H), 7.58 (dd, J = 8.2, 2.0 Hz, 1H), 5.69 (s, 1H), 4.48 – 4.42 (m, 2H), 4.33 – 4.28 (m, 2H), 2.61 (s, 3H), 2.48 (s, 3H), 1.57 (s, 6H). ^{13}C NMR (126 MHz, DMSO-d_6) δ 160.42, 150.66, 145.46, 143.29, 139.22, 136.99, 136.82, 136.04, 131.78, 130.19, 129.58, 129.44, 124.15, 122.41, 69.27, 64.82, 63.80, 31.12, 21.56, 16.65. ESI–MS calcd for $C_{21}H_{23}N_2O_3S$ $[M + H]^+$ 383.1424 found 383.1430

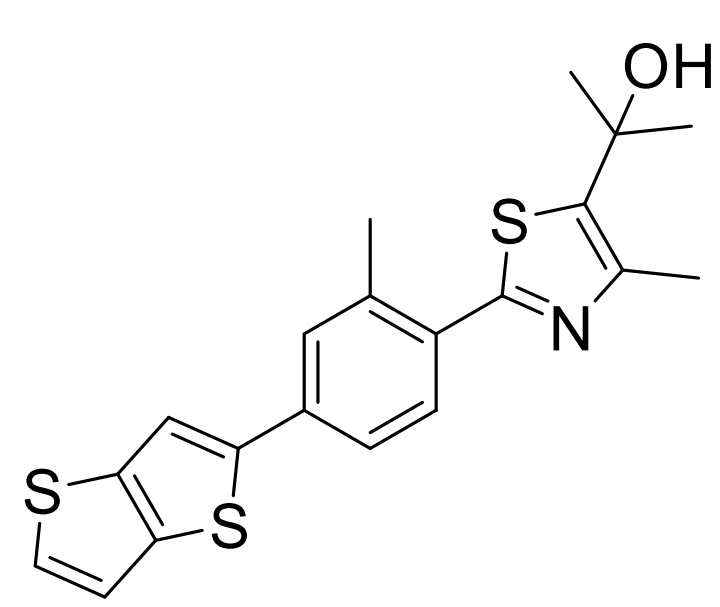

YKJ-277

2-(4-methyl-2-(2-methyl-4-(thieno[3,2-b]thiophen-2-yl)phenyl)thiazol-5-yl)propan-2-ol

Yield: 59.2% ^{1}H NMR (500 MHz, DMSO-d_6) δ 7.98 (s, 1H), 7.80 (d, *J* = 8.2 Hz, 1H), 7.71 (d, *J* = 5.3 Hz, 1H), 7.67 (d, *J* = 2.1 Hz, 1H), 7.61 (dd, *J* = 8.1, 2.1 Hz, 1H), 7.48 (d, *J* = 5.2 Hz, 1H), 5.70 (s, 1H), 2.62 (s, 3H), 2.48 (s, 3H), 1.57 (s, 6H). ^{13}C NMR (126 MHz, DMSO-d_6) δ 160.23, 145.50, 144.26, 143.39, 139.88, 138.35, 136.22, 134.42, 131.94, 129.78, 128.79, 128.08, 123.07, 120.21, 116.85, 69.29, 31.11, 21.59, 16.63. ESI–MS calcd for $C_{20}H_{20}NOS_3$ $[M + H]^+$ 386.0702 found 386.0712

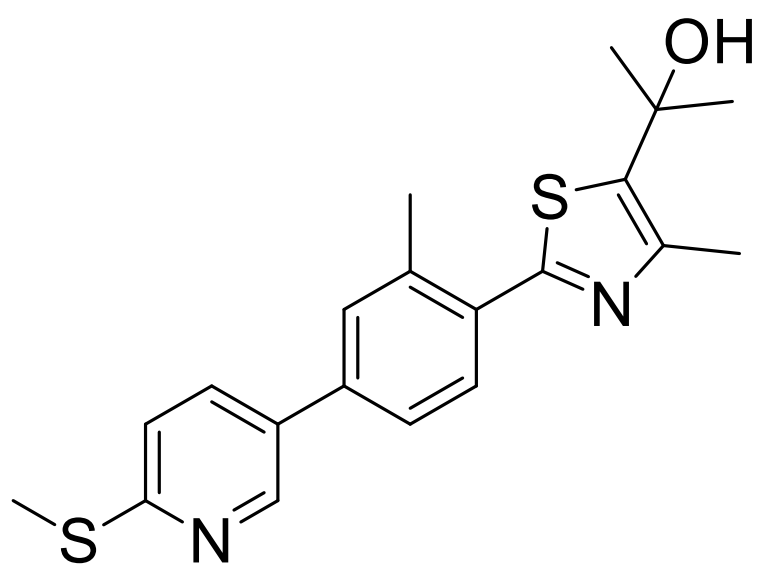

YKJ-278

2-(4-methyl-2-(2-methyl-4-(6-(methylthio)pyridin-3-yl)phenyl)thiazol-5-yl)propan-2-ol

Yield: 80.9% ^{1}H NMR (500 MHz, DMSO-d_6) δ 8.83 (dd, *J* = 2.5, 0.9 Hz, 1H), 8.02 (dd, *J* = 8.4, 2.5 Hz, 1H), 7.82 (d, *J* = 8.1 Hz, 1H), 7.71 (d, *J* = 2.0 Hz, 1H), 7.63 (dd, *J* = 8.2, 2.1 Hz, 1H), 7.40 (dd, *J* = 8.4, 0.9 Hz, 1H), 5.69 (s, 1H), 2.62 (s, 3H), 2.56 (s, 3H), 2.48 (s, 3H), 1.58 (s, 6H). ^{13}C NMR (126 MHz, DMSO-d_6) δ 160.36, 158.70, 147.17, 145.49, 143.40, 136.93, 136.15, 134.45, 132.04, 130.45, 129.69, 129.44, 124.17, 121.02, 69.28, 31.11, 21.60, 16.64, 12.83. ESI–MS calcd for $C_{20}H_{23}N_2OS_2$ $[M + H]^+$ 371.1246 found 371.1255

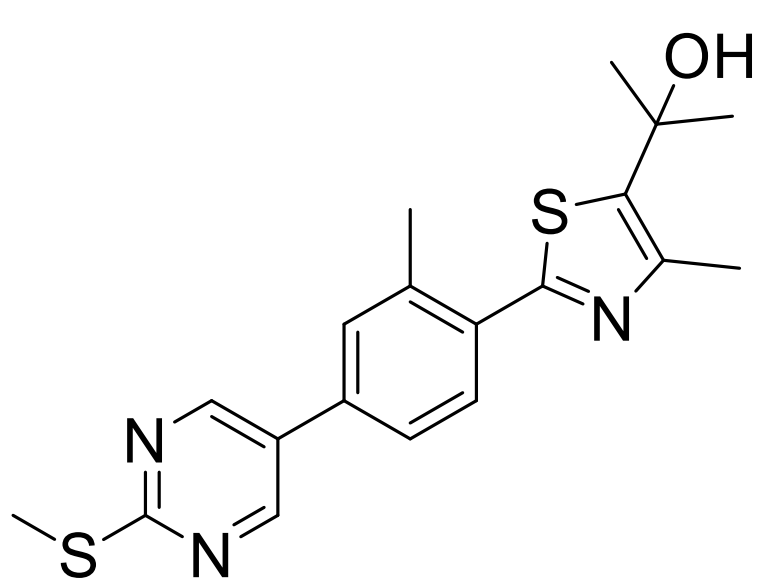

YKJ-279

2-(4-methyl-2-(2-methyl-4-(2-(methylthio)pyrimidin-5-yl)phenyl)thiazol-5-yl)propan-2-ol

Yield: 93.9% ^{1}H NMR (500 MHz, DMSO-d_6) δ 9.04 (s, 2H), 7.84 (d, *J* = 8.2 Hz, 1H), 7.78 (d, *J* = 2.0 Hz, 1H), 7.70 (dd, *J* = 8.1, 2.0 Hz, 1H), 5.71 (s, 1H), 2.63 (s, 3H), 2.57 (s, 3H), 2.48 (s, 3H), 1.58 (s, 6H). ^{13}C NMR (126 MHz, DMSO-d_6) δ 170.48, 160.18, 155.26, 145.55, 143.63, 136.31, 133.93, 132.60, 129.74, 129.43, 127.74, 124.16, 69.30, 31.08, 21.59, 16.64, 13.67. ESI–MS calcd for $C_{19}H_{22}N_3OS_2$ $[M + H]^+$ 372.1199 found 372.1214

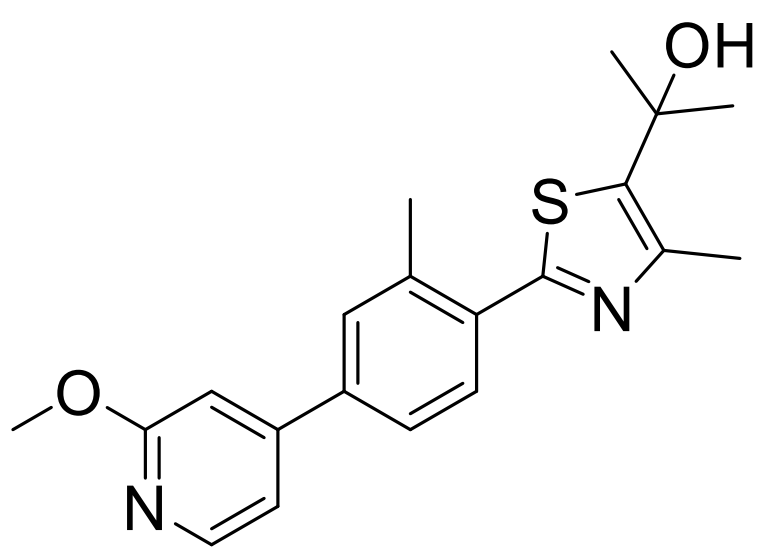

YKJ-280

2-(2-(4-(2-methoxypyridin-4-yl)-2-methylphenyl)-4-methylthiazol-5-yl)propan-2-ol

Yield: 89.5% ^{1}H NMR (500 MHz, DMSO-d_6) δ 8.24 (d, *J* = 5.6 Hz, 1H), 7.84 (d, *J* = 8.2 Hz, 1H), 7.78 (d, *J* = 2.0 Hz, 1H), 7.70 (dd, *J* = 8.1, 2.0 Hz, 1H), 7.36 (dd, *J* = 5.4, 1.6 Hz, 1H), 7.17 (d, *J* = 0.9 Hz, 1H), 5.71 (s, 1H), 3.90 (s, 3H), 2.63 (s, 3H), 2.48 (s, 3H), 1.58 (s, 6H). ^{13}C NMR (126 MHz, DMSO-d_6) δ 164.51, 160.12, 149.35, 147.55, 145.57, 143.72, 137.19, 136.15, 133.32, 129.98, 129.63, 124.65, 114.98, 107.41, 69.30, 53.29, 31.08, 21.54, 16.63. ESI–MS calcd for $C_{20}H_{23}N_2O_2S$ $[M + H]^+$ 355.1475 found 355.1488

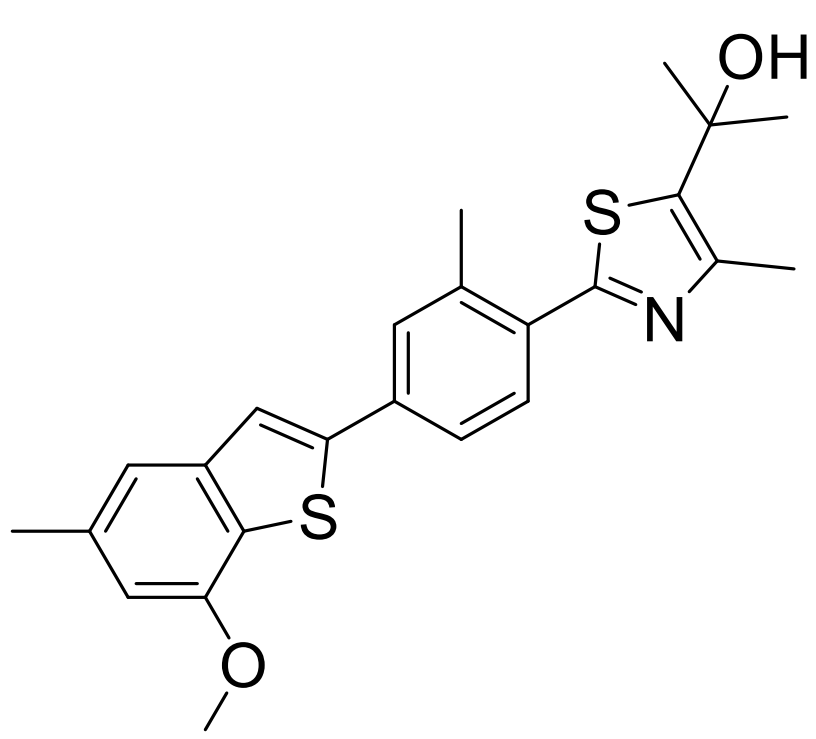

YKJ-281

2-(2-(4-(7-methoxy-5-methylbenzo[b]thiophen-2-yl)-2-methylphenyl)-4-methylthiazol-5-yl)propan-2-ol

Yield: 84.6% ^{1}H NMR (500 MHz, DMSO-d_6) δ 7.86 – 7.81 (m, 2H), 7.75 (d, *J* = 2.1 Hz, 1H), 7.68 (dd, *J* = 8.1, 2.0 Hz, 1H), 7.26 (s, 1H), 6.81 (s, 1H), 5.71 (s, 1H), 3.96 (s, 3H), 2.63 (s, 3H), 2.48 (s, 3H), 2.44 (s, 3H), 1.58 (s, 6H). ^{13}C NMR (126 MHz, DMSO-d_6) δ 160.16, 153.51, 145.55, 143.56, 142.60, 142.03, 136.20, 136.10, 133.84, 132.45, 129.73, 128.88, 124.30, 123.77, 120.78, 116.29, 107.02, 69.31, 55.69, 31.10, 21.58, 21.53, 16.64. ESI–MS calcd for $C_{24}H_{26}NO_2S_2$ $[M + H]^+$ 424.1399 found 424.1424

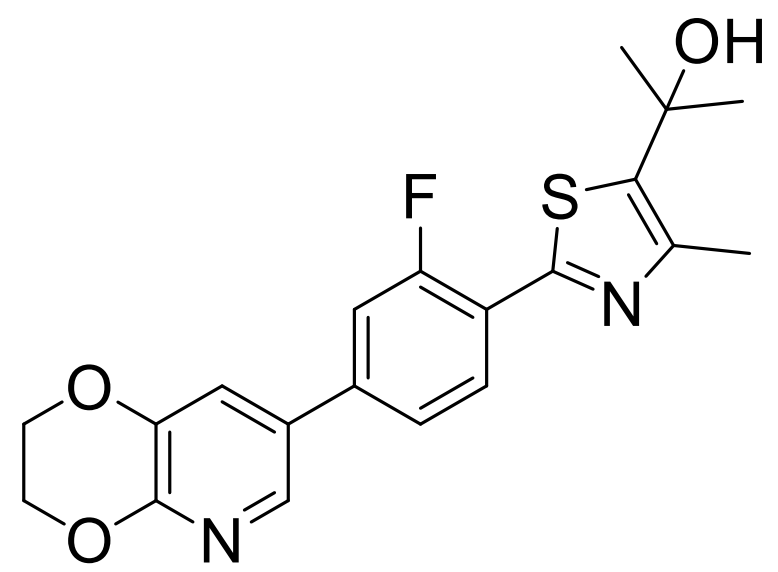

YKJ-282

2-(2-(4-(2,3-dihydro-[1,4]dioxino[2,3-b]pyridin-7-yl)-2-fluorophenyl)-4-methylthiazol-5-yl)propan-2-ol

Yield: 81.6% ^{1}H NMR (500 MHz, DMSO-d_6) δ 8.20 (d, *J* = 2.3 Hz, 1H), 8.18 (t, *J* = 8.2 Hz, 1H), 7.77 (dd, *J* = 13.1, 1.8 Hz, 1H), 7.75 (d, *J* = 2.2 Hz, 1H), 7.67 (dd, *J* = 8.3, 1.8 Hz, 1H), 4.49 – 4.42 (m, 2H), 4.33 – 4.29 (m, 2H), 2.49 (s, 3H), 1.57 (s, 6H). ^{13}C NMR (126 MHz, DMSO-d_6) δ 159.34 (d, *J* = 249.0 Hz), 153.12 (d, *J* = 5.3 Hz), 151.09, 145.30, 144.54 (d, *J* = 7.3 Hz), 139.27, 139.19 (d, *J* = 8.8 Hz), 137.24, 128.88 (d, *J* = 2.1 Hz), 128.11 (d, *J* = 3.6 Hz), 122.76 (d, *J* = 2.8 Hz), 122.51, 119.80 (d, *J* = 12.0 Hz), 113.95 (d, *J* = 23.1 Hz), 69.34, 64.88, 63.78, 30.89, 16.50. ESI–MS calcd for $C_{20}H_{20}FN_2O_3S$ $[M + H]^+$ 387.1173 found 387.1184

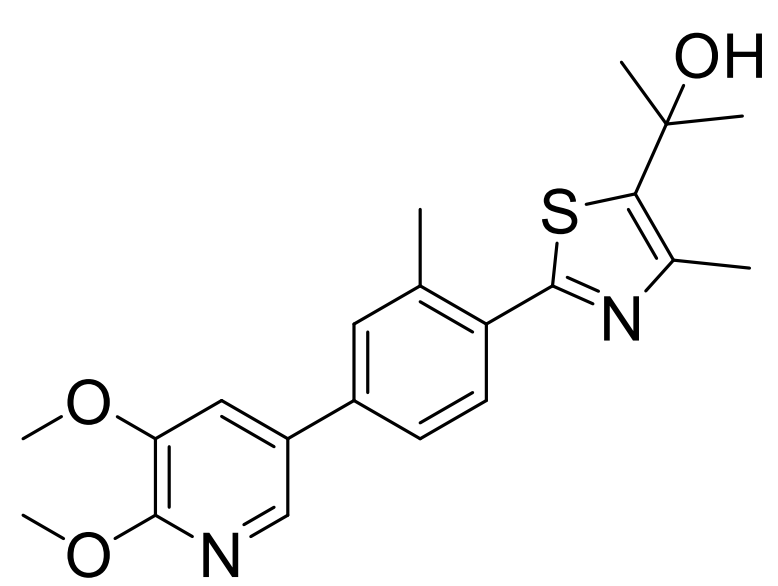

YKJ-283

2-(2-(4-(5,6-dimethoxypyridin-3-yl)-2-methylphenyl)-4-methylthiazol-5-yl)propan-2-ol

Yield: 52.6% ^{1}H NMR (500 MHz, DMSO-d_6) δ 8.07 (d, J = 2.1 Hz, 1H), 7.79 (d, J = 8.2 Hz, 1H), 7.69 (d, J = 1.9 Hz, 1H), 7.62 (dd, J = 8.1, 2.0 Hz, 1H), 7.59 (d, J = 2.0 Hz, 1H), 5.69 (s, 1H), 3.91 (s, 3H), 3.90 (s, 3H), 2.63 (s, 3H), 2.48 (s, 3H), 1.58 (s, 6H). ^{13}C NMR (126 MHz, DMSO-d_6) δ 160.51, 153.59, 145.44, 143.83, 143.23, 137.54, 135.98, 134.27, 131.60, 129.52 (two peaks overlap), 129.01, 124.26, 116.56, 69.28, 55.64, 53.20, 31.12, 21.60, 16.65. ESI–MS calcd for $C_{21}H_{25}N_2O_3S$ $[M + H]^+$ 385.1580 found 385.1587

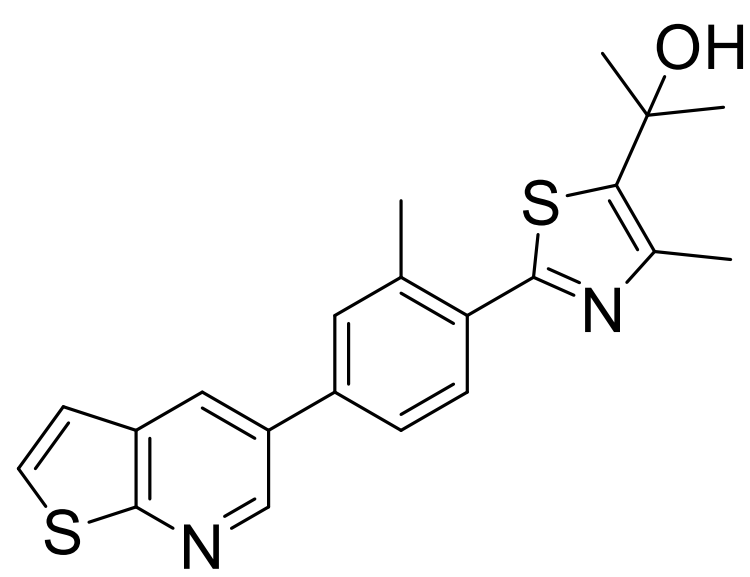

YKJ-284

2-(4-methyl-2-(2-methyl-4-(thieno[2,3-b]pyridin-5-yl)phenyl)thiazol-5-yl)propan-2-ol

Yield: 57.1% ^{1}H NMR (500 MHz, DMSO-d_6) δ 8.95 (d, J = 2.2 Hz, 1H), 8.62 (d, J = 2.2 Hz, 1H), 7.95 (d, J = 5.9 Hz, 1H), 7.87 (d, J = 8.1 Hz, 1H), 7.81 (d, J = 2.0 Hz, 1H), 7.73 (dd, J = 8.1, 2.0 Hz, 1H), 7.52 (d, J = 5.9 Hz, 1H), 5.71 (s, 1H), 2.66 (s, 3H), 2.49 (s, 3H), 1.58 (s, 6H). ^{13}C NMR (126 MHz, DMSO-d_6) δ 160.33, 160.24, 145.52, 145.25, 143.46, 137.55, 136.21, 132.57, 132.16, 131.21, 130.18, 129.76, 129.21, 128.73, 124.87, 122.31, 69.29, 31.12, 21.59, 16.65. ESI–MS calcd for $C_{21}H_{21}N_2OS_2$ $[M + H]^+$ 381.1090 found 381.1095

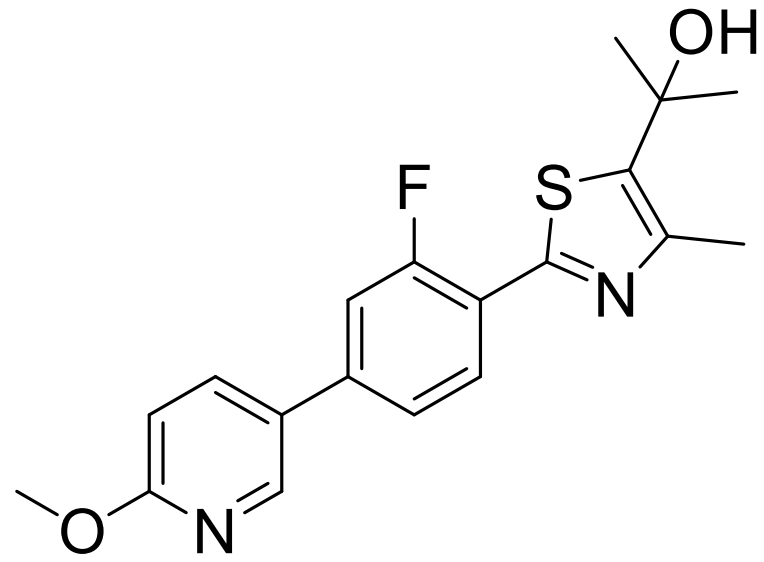

YKJ-285

2-(2-(2-fluoro-4-(6-methoxypyridin-3-yl)phenyl)-4-methylthiazol-5-yl)propan-2-ol

Yield: 89.9% ^{1}H NMR (500 MHz, DMSO-d_6) δ 8.62 (dd, *J* = 2.6, 0.8 Hz, 1H), 8.20 (t, *J* = 8.2 Hz, 1H), 8.14 (dd, *J* = 8.7, 2.6 Hz, 1H), 7.77 (dd, *J* = 13.0, 1.8 Hz, 1H), 7.67 (dd, *J* = 8.3, 1.8 Hz, 1H), 6.94 (dd, *J* = 8.7, 0.7 Hz, 1H), 5.75 (s, 1H), 3.91 (s, 3H), 2.49 (s, 3H), 1.57 (s, 6H). ^{13}C NMR (126 MHz, DMSO-d_6) δ 163.63, 159.39 (d, *J* = 249.0 Hz), 153.16 (d, *J* = 5.3 Hz), 145.28, 145.13, 144.50 (d, *J* = 7.3 Hz), 139.61 (d, *J* = 8.6 Hz), 137.55, 128.19 (d, *J* = 3.6 Hz), 127.16 (d, *J* = 2.2 Hz), 122.60 (d, *J* = 2.9 Hz), 119.64 (d, *J* = 12.1 Hz), 113.72 (d, *J* = 23.1 Hz), 110.76, 69.35, 53.42, 30.90, 16.50. ESI–MS calcd for $C_{19}H_{20}FN_2O_2S$ $[M + H]^+$ 359.1224 found 359.1234

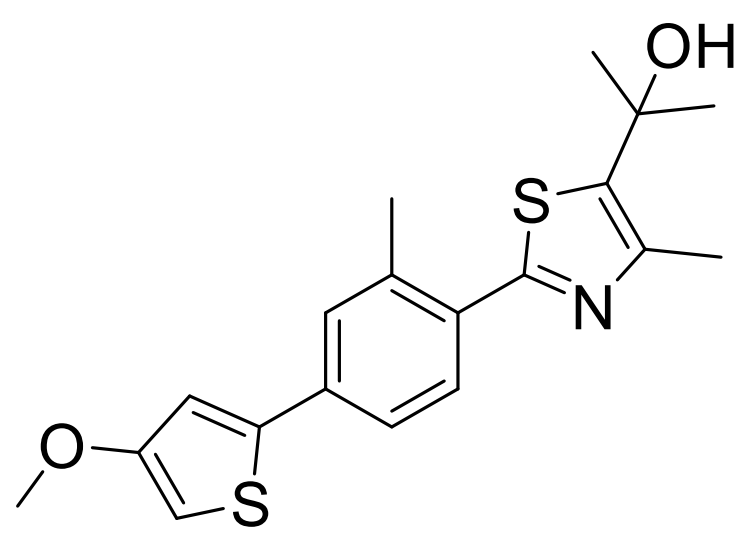

YKJ-286

2-(2-(4-(4-methoxythiophen-2-yl)-2-methylphenyl)-4-methylthiazol-5-yl)propan-2-ol

Yield: 81.6% ^{1}H NMR (500 MHz, DMSO-d_6) δ 7.75 (d, *J* = 8.2 Hz, 1H), 7.60 (d, *J* = 2.0 Hz, 1H), 7.53 (dd, *J* = 8.1, 2.0 Hz, 1H), 7.30 (d, *J* = 1.7 Hz, 1H), 6.64 (d, *J* = 1.7 Hz, 1H), 5.69 (s, 1H), 3.78 (s, 3H), 2.59 (s, 3H), 2.47 (s, 3H), 1.57 (s, 6H). ^{13}C NMR (126 MHz, DMSO-d_6) δ 160.26, 158.53, 145.46, 143.33, 140.83, 136.08, 133.85, 131.88, 129.63, 127.66, 122.62, 116.17, 97.94, 69.27, 57.18, 31.10, 21.51, 16.62. ESI–MS calcd for $C_{19}H_{22}NO_2S_2$ $[M + H]^+$ 360.1086 found 360.1107

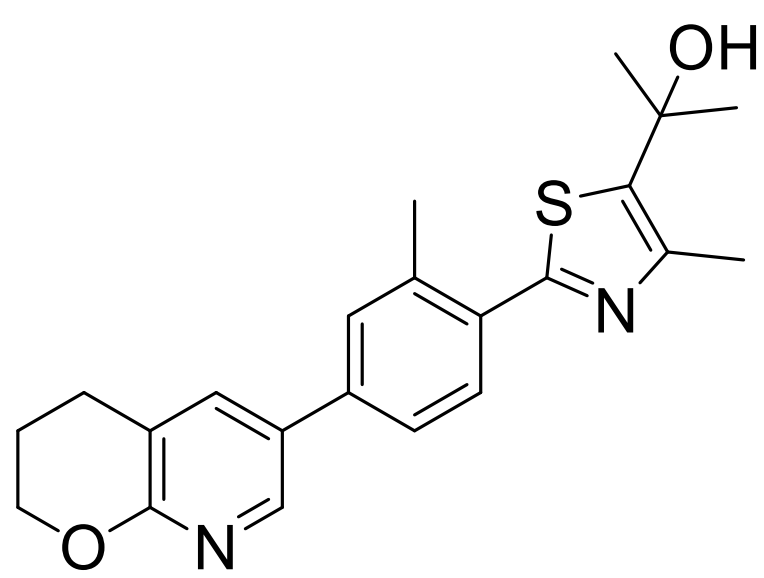

YKJ-287

2-(2-(4-(3,4-dihydro-2H-pyrano[2,3-b]pyridin-6-yl)-2-methylphenyl)-4-methylthiazol-5-yl)propan-2-ol

Yield: 84.6% ^{1}H NMR (500 MHz, DMSO-d_6) δ 8.36 (d, J = 2.6 Hz, 1H), 7.89 (d, J = 2.6 Hz, 1H), 7.79 (d, J = 8.2 Hz, 1H), 7.65 (d, J = 2.0 Hz, 1H), 7.57 (dd, J = 8.2, 2.0 Hz, 1H), 5.68 (s, 1H), 4.34 – 4.28 (m, 2H), 2.86 (t, J = 6.4 Hz, 2H), 2.61 (s, 3H), 2.48 (s, 3H), 1.99 – 1.91 (m, 2H), 1.57 (s, 6H). ^{13}C NMR (126 MHz, DMSO-d_6) δ 160.80, 160.49, 145.43, 143.66, 143.20, 137.30, 136.85, 136.01, 131.49, 129.62, 129.10, 128.35, 123.85, 117.65, 69.27, 66.92, 31.13, 24.25, 21.57, 21.29, 16.64. ESI–MS calcd for $C_{22}H_{25}N_2O_2S$ $[M + H]^+$ 381.1631 found 381.1638

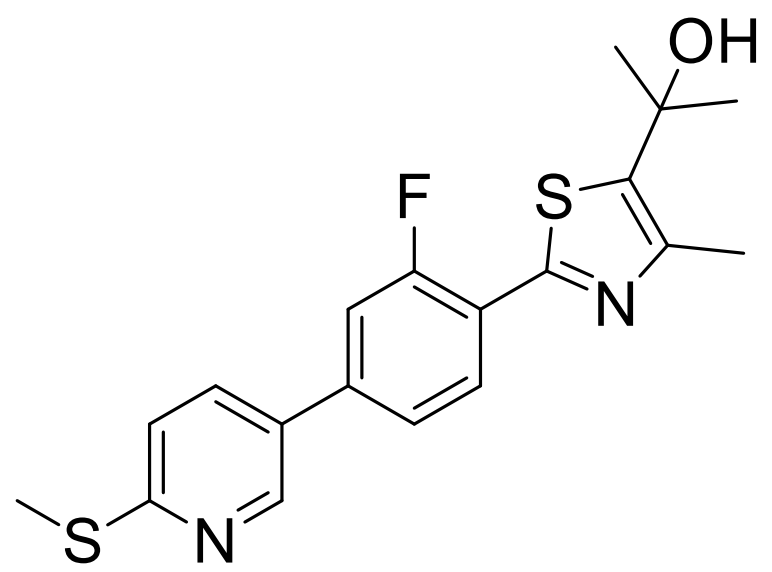

YKJ-288

2-(2-(2-fluoro-4-(6-(methylthio)pyridin-3-yl)phenyl)-4-methylthiazol-5-yl)propan-2-ol

Yield: 77.6% ^{1}H NMR (500 MHz, DMSO-d_6) δ 8.88 (dd, J = 2.5, 0.9 Hz, 1H), 8.22 (t, J = 8.2 Hz, 1H), 8.07 (dd, J = 8.5, 2.5 Hz, 1H), 7.82 (dd, J = 13.0, 1.8 Hz, 1H), 7.71 (dd, J = 8.3, 1.8 Hz, 1H), 7.42 (dd, J = 8.5, 0.8 Hz, 1H), 5.76 (s, 1H), 2.56 (s, 3H), 2.49(s, 3H), 1.57 (s, 6H). ^{13}C NMR (126 MHz, DMSO-d_6) δ 159.54, 159.39 (d, J = 249.5 Hz), 153.07 (d, J = 5.3 Hz), 147.28, 145.34, 144.67 (d, J = 7.4 Hz), 139.29 (d, J = 8.6 Hz), 134.47, 129.18 (d, J = 2.0 Hz), 128.24 (d, J = 3.6 Hz), 122.78 (d, J = 2.7 Hz), 121.03, 120.08 (d, J = 12.2 Hz), 113.95 (d, J = 23.0 Hz), 69.35, 30.88, 16.50, 12.82. ESI–MS calcd for $C_{19}H_{20}FN_2OS_2$ $[M + H]^+$ 375.0996 found 375.1000

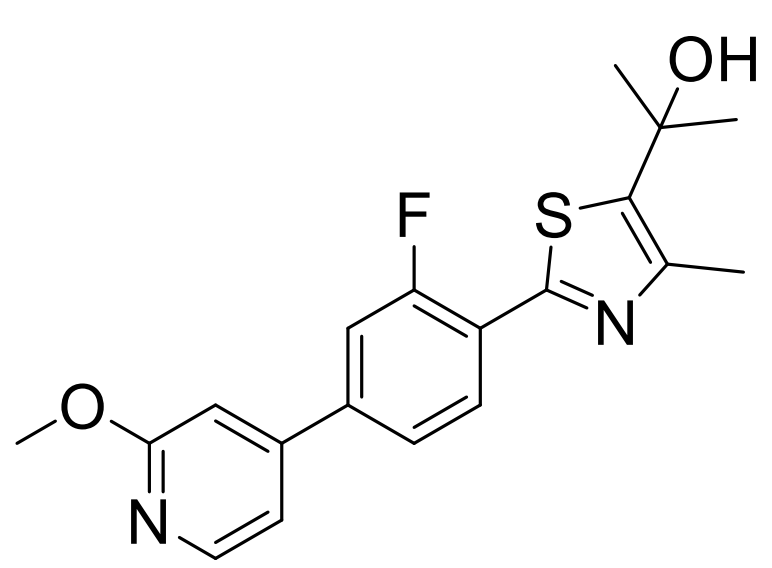

YKJ-289

2-(2-(2-fluoro-4-(2-methoxypyridin-4-yl)phenyl)-4-methylthiazol-5-yl)propan-2-ol

Yield: 82.6% ^{1}H NMR (500 MHz, DMSO-d_6) δ 8.28 – 8.20 (m, 2H), 7.89 (dd, *J* = 12.8, 1.8 Hz, 1H), 7.78 (dd, *J* = 8.4, 1.8 Hz, 1H), 7.41 (dd, *J* = 5.4, 1.6 Hz, 1H), 7.23 (d, *J* = 0.9 Hz, 1H), 5.77 (s, 1H), 3.90 (s, 3H), 2.49 (s, 3H), 1.57 (s, 6H). ^{13}C NMR (126 MHz, DMSO-d_6) δ 164.57, 159.26 (d, *J* = 249.6 Hz), 152.83 (d, *J* = 5.3 Hz), 147.95 (d, *J* = 2.0 Hz), 147.72, 145.47, 145.09 (d, *J* = 7.4 Hz), 139.43 (d, *J* = 8.5 Hz), 128.20 (d, *J* = 3.3 Hz), 123.38 (d, *J* = 2.7 Hz), 121.42 (d, *J* = 12.1 Hz), 114.87, 114.75 (d, *J* = 23.4 Hz), 107.61, 69.37, 53.36, 30.85, 16.49. ESI–MS calcd for $C_{19}H_{20}FN_2O_2S$ $[M + H]^+$ 359.1224 found 359.1228

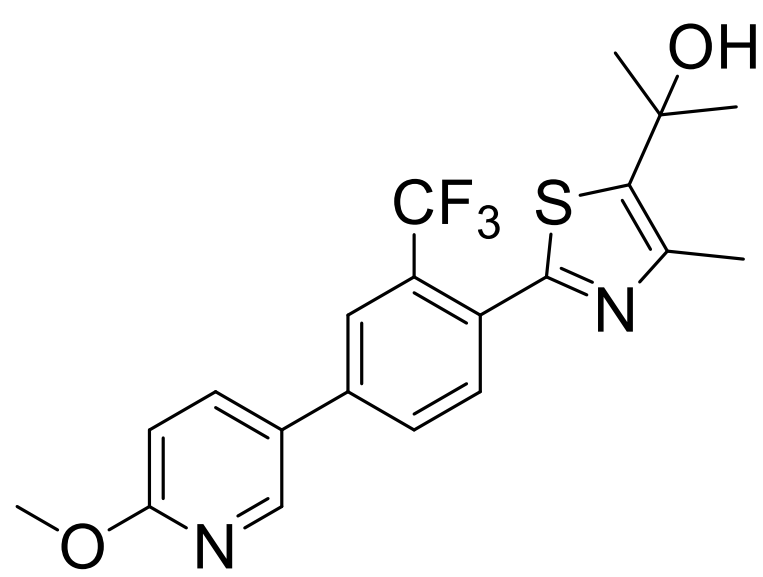

YKJ-290

2-(2-(4-(6-methoxypyridin-3-yl)-2-(trifluoromethyl)phenyl)-4-methylthiazol-5-yl)propan-2-ol

Yield: 78.1% ^{1}H NMR (500 MHz, DMSO-d_6) δ 8.64 (d, *J* = 2.6 Hz, 1H), 8.18 (dd, *J* = 8.7, 2.6 Hz, 1H), 8.10 (d, *J* = 1.8 Hz, 1H), 8.04 (dd, *J* = 8.1, 1.9 Hz, 1H), 7.78 (d, *J* = 8.1 Hz, 1H), 6.97 (d, *J* = 8.6 Hz, 1H), 5.78 (s, 1H), 3.92 (s, 3H), 2.46 (s, 3H), 1.58 (s, 6H). ^{13}C NMR (126 MHz, DMSO-d_6) δ 163.74, 157.63, 145.52, 145.40, 145.27, 138.38, 137.92, 132.96, 131.22 (d, *J* = 1.5 Hz), 130.06, 127.47 (q, *J* = 30.2 Hz), 127.19, 124.48 (q, *J* = 5.0 Hz), 123.66 (q, *J* = 274.7 Hz), 110.83, 69.33, 53.45, 30.97, 16.48. ESI–MS calcd for $C_{20}H_{20}F_3N_2O_2S$ $[M + H]^+$ 409.1192 found 409.1192

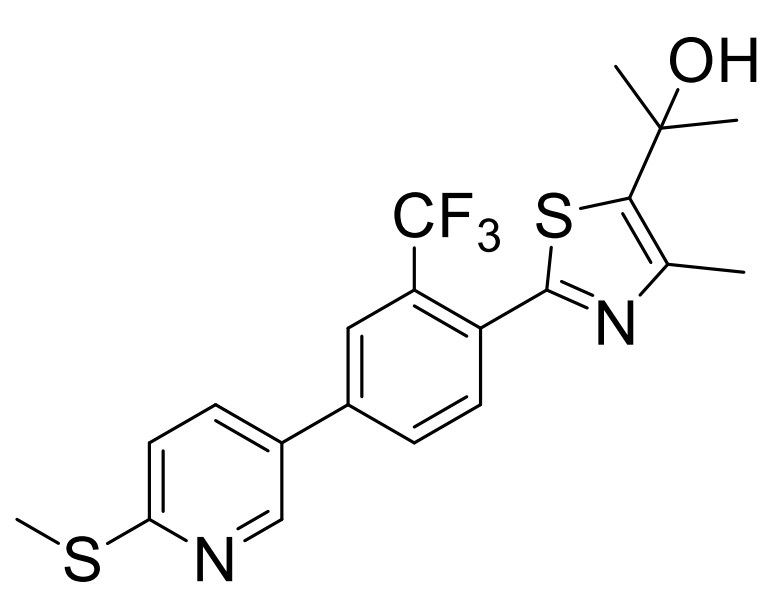

YKJ-291

2-(4-methyl-2-(4-(6-(methylthio)pyridin-3-yl)-2-(trifluoromethyl)phenyl)thiazol-5-yl)propan-2-ol

Yield: 68.2% ^{1}H NMR (500 MHz, DMSO-d_6) δ 8.90 (d, J = 2.1 Hz, 1H), 8.17 – 8.06 (m, 3H), 7.80 (d, J = 8.1 Hz, 1H), 7.44 (d, J = 8.4 Hz, 1H), 5.78 (s, 1H), 2.57 (s, 3H), 2.46 (s, 3H), 1.58 (s, 6H). ^{13}C NMR (126 MHz, DMSO-d_6) δ 159.77, 157.55, 147.53, 145.56, 145.38, 138.12, 134.88, 133.02, 131.64 (d, J = 1.4 Hz), 130.26, 129.22, 127.53 (q, J = 30.8 Hz), 124.71 (q, J = 6.3 Hz), 123.63 (q, J = 274.7 Hz), 121.09, 69.34, 30.97, 16.48, 12.83. ESI–MS calcd for $C_{20}H_{20}F_3N_2OS_2$ [M + H]$^+$ 425.0964 found 425.0969

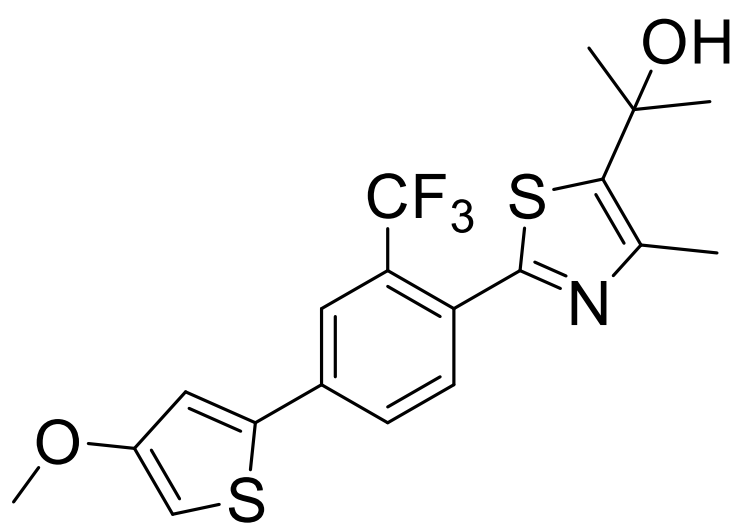

YKJ-292

2-(2-(4-(4-methoxythiophen-2-yl)-2-(trifluoromethyl)phenyl)-4-methylthiazol-5-yl)propan-2-ol

Yield: 92.5% ^{1}H NMR (500 MHz, DMSO-d_6) δ 8.03 (d, J = 2.1 Hz, 1H), 7.96 (dd, J = 8.1, 2.0 Hz, 1H), 7.73 (d, J = 8.1 Hz, 1H), 7.52 (d, J = 1.7 Hz, 1H), 6.75 (d, J = 1.7 Hz, 1H), 5.77 (s, 1H), 3.80 (s, 3H), 2.45 (s, 3H), 1.57 (s, 6H). ^{13}C NMR (126 MHz, DMSO-d_6) δ 158.73, 157.46, 145.58, 145.34, 138.98, 134.87, 133.13, 131.27 (d, J = 1.9 Hz), 128.34, 127.46 (q, J = 30.2 Hz), 123.49 (q, J = 274.7 Hz), 122.70 (q, J = 5.6 Hz), 117.74, 99.37, 69.32, 57.33, 30.96, 16.46. ESI–MS calcd for $C_{19}H_{19}F_3NO_2S_2$ [M + H]$^+$ 414.0804 found 414.0819

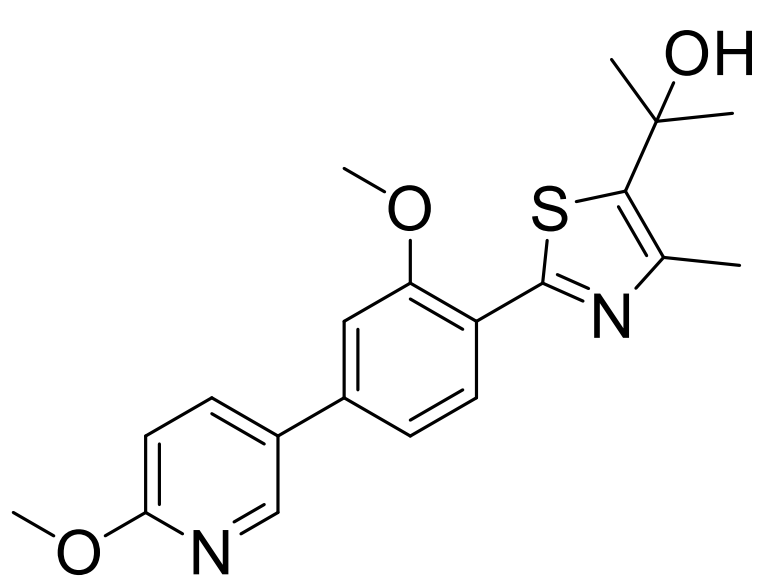

YKJ-293

2-(2-(2-methoxy-4-(6-methoxypyridin-3-yl)phenyl)-4-methylthiazol-5-yl)propan-2-ol

Yield: 82.6% ^{1}H NMR (500 MHz, DMSO-d_6) δ 8.61 (d, *J* = 2.1 Hz, 1H), 8.25 (d, *J* = 8.2 Hz, 1H), 8.13 (dd, *J* = 8.7, 2.6 Hz, 1H), 7.45 (d, *J* = 1.7 Hz, 1H), 7.36 (dd, *J* = 8.2, 1.8 Hz, 1H), 6.94 (d, *J* = 8.6 Hz, 1H), 5.59 (s, 1H), 4.08 (s, 3H), 3.91 (s, 3H), 2.47 (s, 3H), 1.56 (s, 6H). ^{13}C NMR (126 MHz, DMSO-d_6) δ 163.35, 156.13, 155.01, 145.00, 144.34, 143.07, 138.81, 137.67, 128.64, 127.43, 120.84, 118.68, 110.58, 109.93, 69.31, 56.00, 53.34, 31.16, 16.66. ESI–MS calcd for $C_{20}H_{23}N_2O_3S$ [M + H]$^+$ 371.1424 found 371.1443

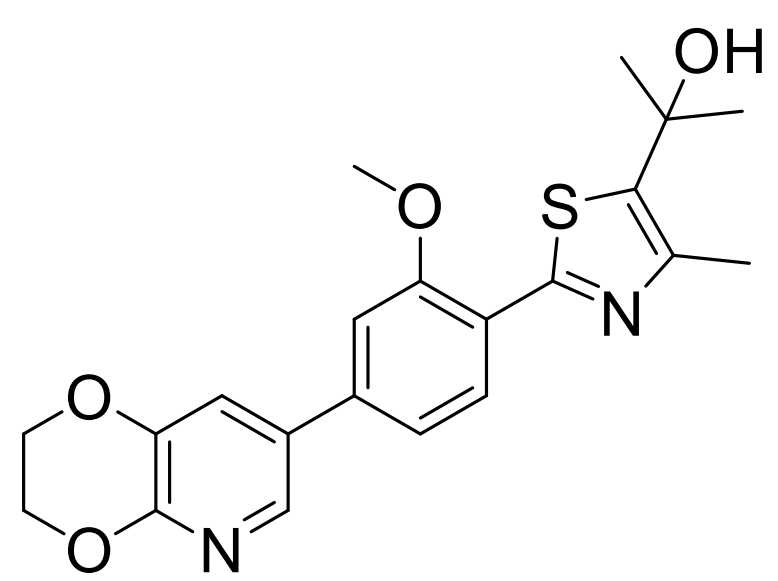

YKJ-294

2-(2-(4-(2,3-dihydro-[1,4]dioxino[2,3-b]pyridin-7-yl)-2-methoxyphenyl)-4-methylthiazol-5-yl)propan-2-ol

Yield: 70.6% ^{1}H NMR (500 MHz, DMSO-d_6) δ 8.23 (d, *J* = 8.1 Hz, 1H), 8.19 (d, *J* = 2.3 Hz, 1H), 7.74 (d, *J* = 2.2 Hz, 1H), 7.43 (d, *J* = 1.7 Hz, 1H), 7.36 (dd, *J* = 8.2, 1.7 Hz, 1H), 5.59 (s, 1H), 4.48 – 4.43 (m, 2H), 4.34 – 4.28 (m, 2H), 4.07 (s, 3H), 2.47 (s, 3H), 1.56 (s, 6H). ^{13}C NMR (126 MHz, DMSO-d_6) δ 156.09, 154.98, 150.73, 144.36, 143.12, 139.19, 138.39, 137.21, 130.43, 127.38, 122.64, 121.00, 118.82, 110.12, 69.31, 64.85, 63.81, 56.02, 31.16, 16.66. ESI–MS calcd for $C_{21}H_{23}N_2O_4S$ [M + H]$^+$ 399.1373 found 399.1375

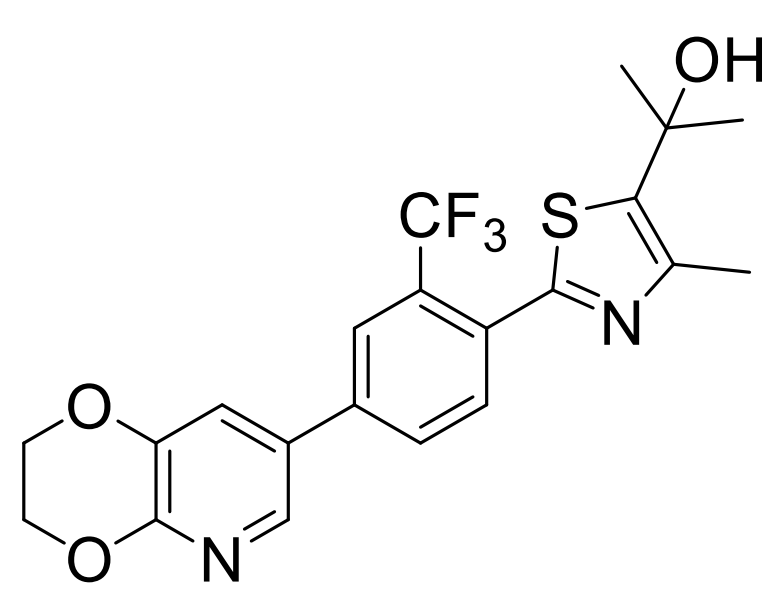

YKJ-295

2-(2-(4-(2,3-dihydro-[1,4]dioxino[2,3-b]pyridin-7-yl)-2-(trifluoromethyl)phenyl)-4-methylthiazol-5-yl)propan-2-ol

Yield: 70.7% ^{1}H NMR (500 MHz, DMSO-d_6) δ 8.21 (d, *J* = 2.3 Hz, 1H), 8.09 (d, *J* = 1.9 Hz, 1H), 8.04 (dd, *J* = 8.0, 1.9 Hz, 1H), 7.80 (d, *J* = 2.2 Hz, 1H), 7.76 (d, *J* = 8.1 Hz, 1H), 5.78 (s, 1H), 4.50 – 4.44 (m, 2H), 4.35 – 4.29 (m, 2H), 2.46 (s, 3H), 1.57 (s, 6H). ^{13}C NMR (126 MHz, DMSO-d_6) δ 157.60, 151.22, 145.53, 145.30, 139.32, 137.97, 137.48, 132.90, 131.39 (d, *J* = 1.3 Hz), 130.25, 128.91, 127.44 (q, *J* = 30.4 Hz), 124.69 (q, *J* = 5.0 Hz), 123.63 (q, *J* = 273.7 Hz), 122.89, 69.33, 64.90, 63.80, 30.97, 16.48. ESI–MS calcd for $C_{21}H_{20}F_3N_2O_3S$ [M + H]$^+$ 437.1141 found 437.1148

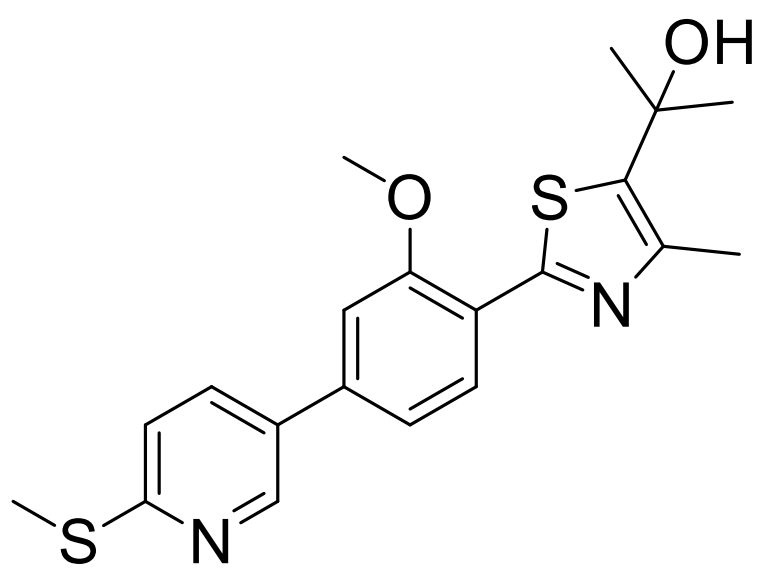

YKJ-296

2-(2-(2-methoxy-4-(6-(methylthio)pyridin-3-yl)phenyl)-4-methylthiazol-5-yl)propan-2-ol

Yield: 80.2% ^{1}H NMR (500 MHz, DMSO-d_6) δ 8.89 (dd, *J* = 2.5, 0.9 Hz, 1H), 8.27 (d, *J* = 8.2 Hz, 1H), 8.06 (dd, *J* = 8.4, 2.5 Hz, 1H), 7.50 (d, *J* = 1.7 Hz, 1H), 7.44 – 7.38 (m, 2H), 5.60 (s, 1H), 4.08 (s, 3H), 2.57 (s, 3H), 2.47 (s, 3H), 1.56 (s, 6H). ^{13}C NMR (126 MHz, DMSO-d_6) δ 158.78, 156.16, 154.91, 147.35, 144.40, 143.25, 138.46, 134.58, 130.66, 127.49, 121.26, 120.98, 118.83, 110.06, 69.32, 56.04, 31.15, 16.66, 12.82. ESI–MS calcd for $C_{20}H_{23}N_2O_2S_2$ [M + H]$^+$ 387.1195 found 387.1198

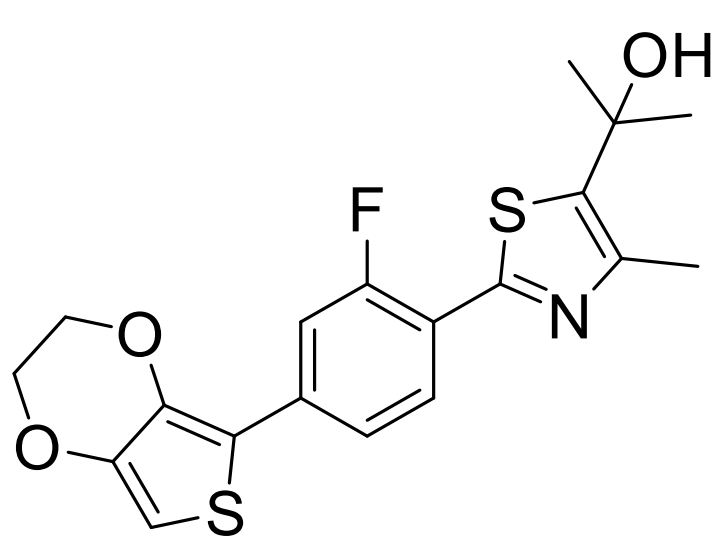

YKJ-297

2-(2-(4-(2,3-Dihydrothieno[3,4-*b*][1,4]dioxin-5-yl)-2-fluorophenyl)-4-methylthiazol-5-yl)propan-2-ol

Yield: 84.4% ^{1}H NMR (500 MHz, DMSO-d_6) δ 8.14 (t, *J* = 8.4 Hz, 1H), 7.64 (dd, *J* = 13.3, 1.8 Hz, 1H), 7.53 (dd, *J* = 8.3, 1.8 Hz, 1H), 6.76 (s, 1H), 5.73 (s, 1H), 4.41 – 4.36 (m, 2H), 4.30 – 4.25 (m, 2H), 2.48 (s, 3H), 1.56 (s, 6H). ^{13}C NMR (126 MHz, DMSO-d_6) δ 159.03 (d, *J* = 248.9 Hz), 153.20 (d, *J* = 5.5 Hz), 145.24, 144.24 (d, *J* = 7.4 Hz), 142.30, 140.02, 135.30 (d, *J* = 9.4 Hz), 128.09 (d, *J* = 3.6 Hz), 121.33 (d, *J* = 2.6 Hz), 118.61 (d, *J* = 12.2 Hz), 113.87 (d, *J* = 2.6 Hz), 112.16 (d, *J* = 24.2 Hz), 99.98, 69.33, 65.03, 64.10, 30.92, 16.48. ESI–MS calcd for $C_{19}H_{19}FNO_3S_2$ $[M + H]^+$ 392.0785 found 392.0799

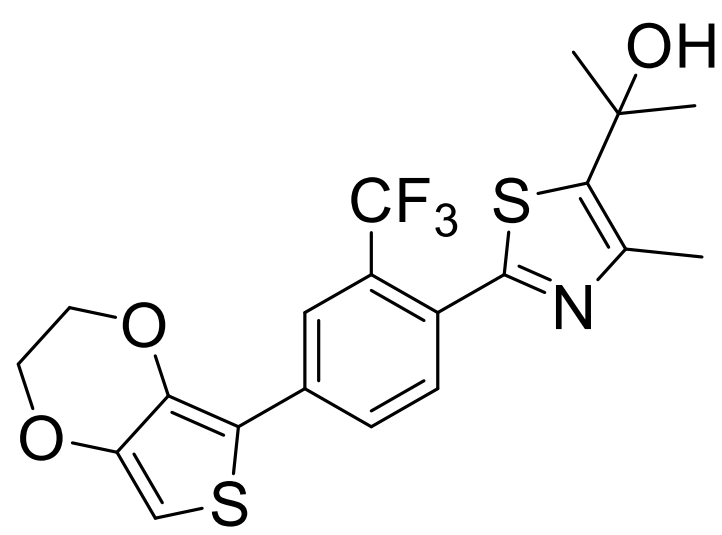

YKJ-298

2-(2-(4-(2,3-Dihydrothieno[3,4-*b*][1,4]dioxin-5-yl)-2-(trifluoromethyl)phenyl)-4-methylthiazol-5-yl)propan-2-ol

Yield: 80.3% ^{1}H NMR (500 MHz, DMSO-d_6) δ 8.11 (d, *J* = 2.0 Hz, 1H), 7.91 (dd, *J* = 8.2, 2.0 Hz, 1H), 7.73 (d, *J* = 8.2 Hz, 1H), 6.80 (s, 1H), 5.76 (s, 1H), 4.43 – 4.38 (m, 2H), 4.32 – 4.26 (m, 2H), 2.45 (s, 3H), 1.57 (s, 6H). ^{13}C NMR (126 MHz, DMSO-d_6) δ 157.62, 145.56, 145.16, 142.38, 140.24, 134.09, 132.97, 129.86, 128.24, 127.12 (q, *J* = 31.5 Hz), 123.58 (q, *J* = 274.7 Hz), 122.69 (q, *J* = 6.3 Hz), 113.39, 100.27, 69.31, 65.12, 64.09, 30.98, 16.46. ESI–MS calcd for $C_{20}H_{19}F_3NO_3S_2$ $[M + H]^+$ 442.0753 found 442.0761

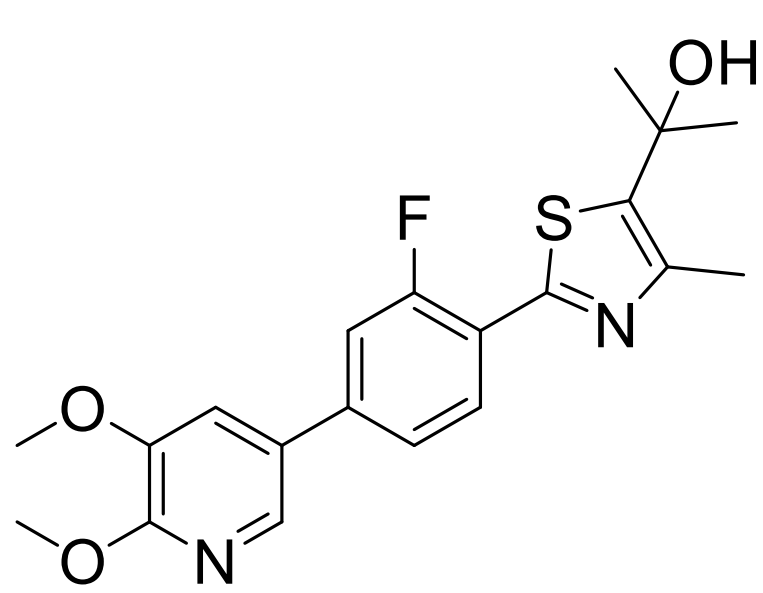

YKJ-299

2-(2-(4-(5,6-Dimethoxypyridin-3-yl)-2-fluorophenyl)-4-methylthiazol-5-yl)propan-2-ol

Yield: 91.1% ^{1}H NMR (500 MHz, DMSO-d_6) δ 8.19 (t, *J* = 8.2 Hz, 1H), 8.14 (d, *J* = 2.0 Hz, 1H), 7.82 (dd, *J* = 13.1, 1.8 Hz, 1H), 7.70 (dd, *J* = 8.3, 1.8 Hz, 1H), 7.65 (d, *J* = 2.1 Hz, 1H), 5.75 (s, 1H), 3.92 (s, 3H), 3.90 (s, 3H), 2.49 (s, 3H), 1.57 (s, 6H). ^{13}C NMR (126 MHz, DMSO-d_6) δ 159.39 (d, *J* = 249.0 Hz), 153.99, 153.22 (d, *J* = 5.2 Hz), 145.29, 144.47 (d, *J* = 7.2 Hz), 143.89, 139.94 (d, *J* = 8.6 Hz), 134.60, 128.04 (d, *J* = 3.5 Hz), 127.74 (d, *J* = 2.0 Hz), 122.81 (d, *J* = 2.6 Hz), 119.63 (d, *J* = 12.2 Hz), 116.49, 114.01 (d, *J* = 23.2 Hz), 69.36, 55.72, 53.30, 30.91, 16.51. ESI–MS calcd for $C_{20}H_{22}FN_2O_3S$ $[M + H]^+$ 389.1330 found 389.1336

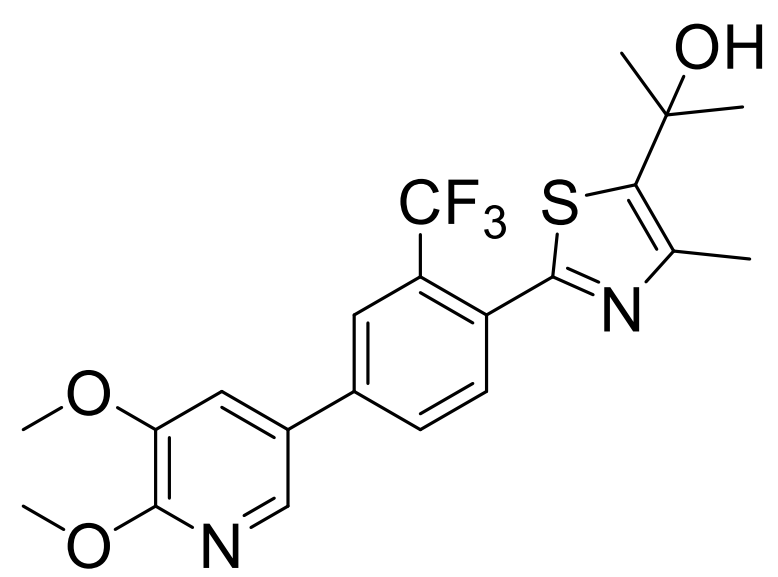

YKJ-300

2-(2-(4-(5,6-Dimethoxypyridin-3-yl)-2-(trifluoromethyl)phenyl)-4-methylthiazol-5-yl)propan-2-ol

Yield: 83.8% ^{1}H NMR (500 MHz, DMSO-d_6) δ 8.16 – 8.11 (m, 2H), 8.07 (dd, *J* = 8.1, 1.9 Hz, 1H), 7.77 (d, *J* = 8.1 Hz, 1H), 7.69 (d, *J* = 2.1 Hz, 1H), 5.78 (s, 1H), 3.93 (s, 3H), 3.91 (s, 3H), 2.46 (s, 3H), 1.58 (s, 6H). ^{13}C NMR (126 MHz, DMSO-d_6) δ 157.69, 154.10, 145.51, 145.26, 143.95, 138.69, 134.86, 132.84, 131.22 (d, *J* = 1.6 Hz), 130.35, 127.81, 127.42 (q, *J* = 31.5 Hz), 124.76 (q, *J* = 5.2 Hz), 123.69 (q, *J* = 273.4 Hz), 116.83, 69.34, 55.77, 53.32, 30.98, 16.49. ESI–MS calcd for $C_{21}H_{22}F_3N_2O_3S$ $[M + H]^+$ 439.1298 found 439.1304

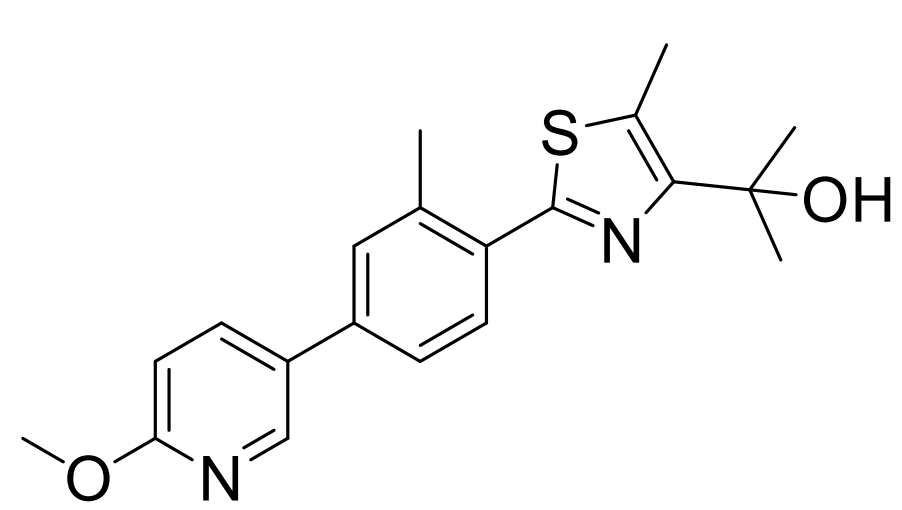

YKJ-301

2-(2-(4-(6-Methoxypyridin-3-yl)-2-methylphenyl)-5-methylthiazol-4-yl)propan-2-ol

Yield: 93.4% ^{1}H NMR (500 MHz, DMSO-d_6) δ 8.55 (d, J = 2.5 Hz, 1H), 8.07 (dd, J = 8.7, 2.6 Hz, 1H), 7.75 (d, J = 8.1 Hz, 1H), 7.65 (d, J = 2.0 Hz, 1H), 7.59 (dd, J = 8.1, 2.0 Hz, 1H), 6.93 (d, J = 8.7 Hz, 1H), 5.07 (s, 1H), 3.90 (s, 3H), 2.63 (s, 3H), 2.61 (s, 3H), 1.55 (s, 6H). ^{13}C NMR (126 MHz, DMSO-d_6) δ 163.28, 159.91, 158.04, 144.80, 137.49, 137.25, 136.17, 131.45, 129.65, 129.39, 128.41, 127.99, 124.06, 110.67, 72.12, 53.34, 31.25, 21.57, 12.03. ESI–MS calcd for $C_{20}H_{23}N_2O_2S$ $[M + H]^+$ 355.1475 found 355.1482

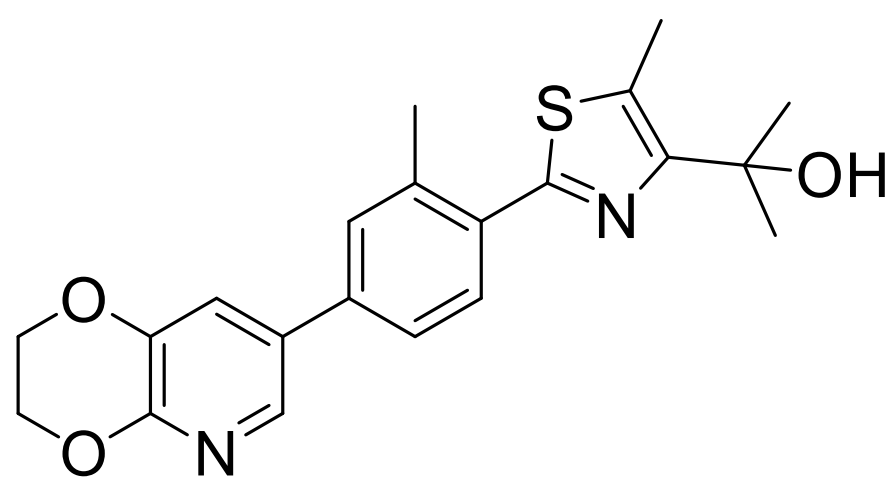

YKJ-302

2-(2-(4-(2,3-Dihydro-[1,4]dioxino[2,3-*b*]pyridin-7-yl)-2-methylphenyl)-5-methylthiazol-4-yl)propan-2-ol

Yield: 63.1% ^{1}H NMR (500 MHz, DMSO-d_6) δ 8.13 (d, J = 2.3 Hz, 1H), 7.73 (d, J = 8.1 Hz, 1H), 7.68 – 7.64 (m, 2H), 7.59 (dd, J = 8.0, 2.1 Hz, 1H), 5.07 (s, 1H), 4.47 – 4.42 (m, 2H), 4.33 – 4.28 (m, 2H), 2.63 (s, 3H), 2.60 (s, 3H), 1.55 (s, 6H). ^{13}C NMR (126 MHz, DMSO-d_6) δ 159.85, 158.05, 150.67, 139.23, 136.99, 136.82, 136.15, 131.61, 130.18, 129.59, 129.53, 128.03, 124.20, 122.41, 72.11, 64.82, 63.80, 31.24, 21.53, 12.03. ESI–MS calcd for $C_{21}H_{23}N_2O_3S$ $[M + H]^+$ 383.1424 found 383.1424

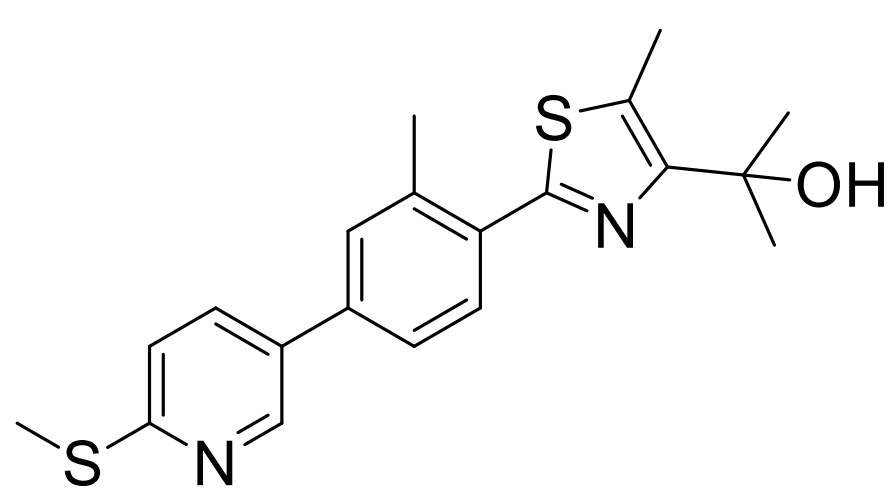

YKJ-303

2-(5-Methyl-2-(2-methyl-4-(6-(methylthio)pyridin-3-yl)phenyl)thiazol-4-yl)propan-2-ol

Yield: 71.6% ^{1}H NMR (500 MHz, DMSO-d_6) δ 8.82 (d, J = 2.4 Hz, 1H), 8.01 (dd, J = 8.5, 2.5 Hz, 1H), 7.77 (d, J = 8.1 Hz, 1H), 7.71 (d, J = 1.9 Hz, 1H), 7.64 (dd, J = 8.1, 2.0 Hz, 1H), 7.40 (d, J = 8.4 Hz, 1H), 5.08 (s, 1H), 2.63 (s, 3H), 2.62 (s, 3H), 2.56 (s, 3H), 1.55 (s, 6H). ^{13}C NMR (126 MHz, DMSO-d_6) δ 159.80, 158.72, 158.09, 147.17, 136.92, 136.26, 134.43, 131.86, 130.45, 129.70, 129.54, 128.13, 124.21, 121.03, 72.11, 31.24, 21.57, 12.83, 12.04. ESI–MS calcd for $C_{20}H_{23}N_2OS_2$ $[M + H]^+$ 371.1246 found 371.1260

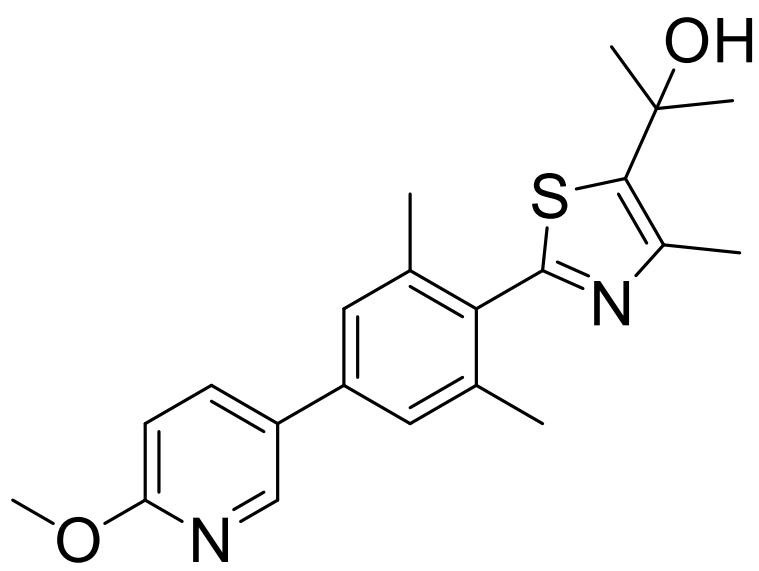

YKJ-304

2-(2-(4-(6-Methoxypyridin-3-yl)-2,6-dimethylphenyl)-4-methylthiazol-5-yl)propan-2-ol

Yield: 78.5% ^{1}H NMR (500 MHz, DMSO-d_6) δ 8.52 (dd, J = 2.6, 0.8 Hz, 1H), 8.05 (dd, J = 8.7, 2.6 Hz, 1H), 7.44 (s, 2H), 6.92 (dd, J = 8.6, 0.8 Hz, 1H), 5.67 (s, 1H), 3.90 (s, 3H), 2.47 (s, 3H), 2.17 (s, 6H), 1.58 (s, 6H). ^{13}C NMR (126 MHz, DMSO-d_6) δ 163.22, 159.62, 144.91, 144.79, 143.53, 137.73, 137.60, 137.32, 132.83, 128.72, 125.37, 110.59, 69.29, 53.31, 31.20, 20.18, 16.50. ESI–MS calcd for $C_{21}H_{25}N_2O_2S$ $[M + H]^+$ 369.1631 found 369.1639

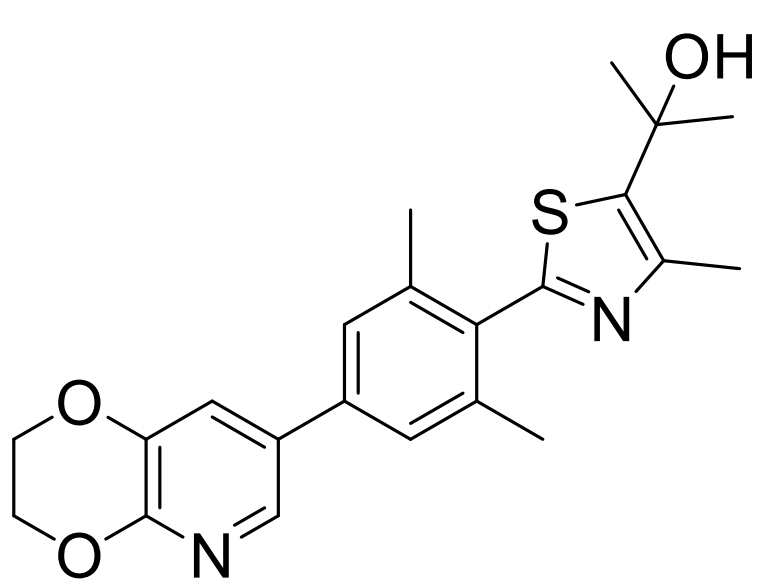

YKJ-305

2-(2-(4-(2,3-Dihydro-[1,4]dioxino[2,3-*b*]pyridin-7-yl)-2,6-dimethylphenyl)-4-methylthiazol-5-yl)propan-2-ol

Yield: 61.5% ^{1}H NMR (500 MHz, DMSO-d_6) δ 8.11 (d, *J* = 2.2 Hz, 1H), 7.63 (d, *J* = 2.2 Hz, 1H), 7.45 (s, 2H), 5.67 (s, 1H), 4.47 – 4.42 (m, 2H), 4.33 – 4.27 (m, 2H), 2.46 (s, 3H), 2.15 (s, 6H), 1.57 (s, 6H). ^{13}C NMR (126 MHz, DMSO-d_6) δ 159.59, 150.58, 144.91, 143.53, 139.17, 137.71, 137.00, 136.88, 133.01, 130.49, 125.49, 122.51, 69.28, 64.80, 63.80, 31.20, 20.14, 16.49. ESI–MS calcd for $C_{22}H_{25}N_2O_3S$ $[M + H]^+$ 397.1580 found 397.1589

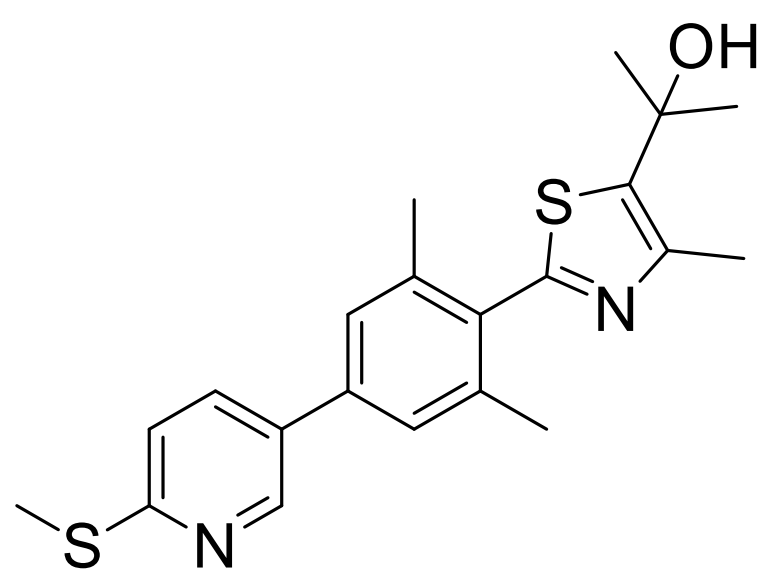

YKJ-306

2-(2-(2,6-Dimethyl-4-(6-(methylthio)pyridin-3-yl)phenyl)-4-methylthiazol-5-yl)propan-2-ol

Yield: 60.5% ^{1}H NMR (500 MHz, DMSO-d_6) δ 8.80 (d, *J* = 2.0 Hz, 1H), 7.99 (dd, *J* = 8.4, 2.5 Hz, 1H), 7.49 (s, 2H), 7.40 (d, *J* = 8.3 Hz, 1H), 5.68 (s, 1H), 2.56 (s, 3H), 2.47 (s, 3H), 2.17 (s, 6H), 1.58 (s, 6H). ^{13}C NMR (126 MHz, DMSO-d_6) δ 159.54, 158.59, 147.23, 144.94, 143.59, 137.86, 137.02, 134.56, 133.30, 130.77, 125.50, 120.98, 69.30, 31.20, 20.18, 16.50, 12.84. ESI–MS calcd for $C_{21}H_{25}N_2OS_2$ $[M + H]^+$ 385.1403 found 385.1403

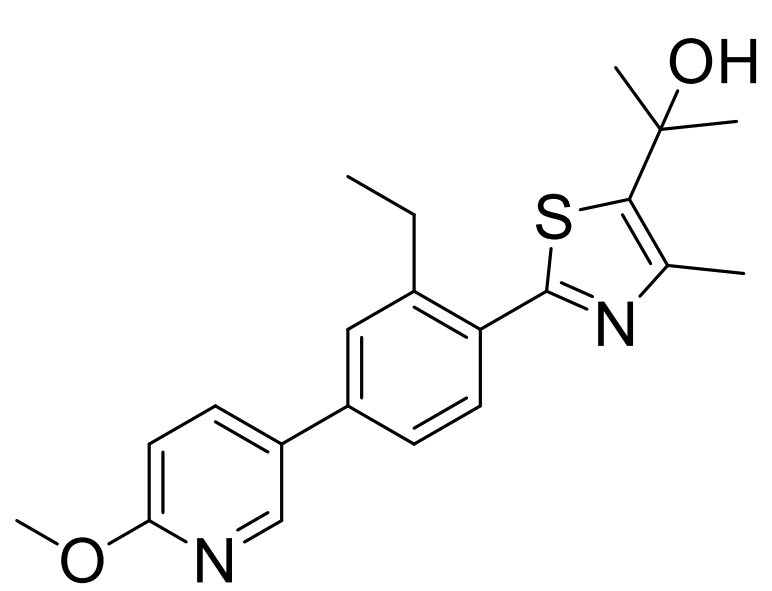

YKJ-307

2-(2-(2-Ethyl-4-(6-methoxypyridin-3-yl)phenyl)-4-methylthiazol-5-yl)propan-2-ol

Yield: 83.4% ^{1}H NMR (500 MHz, DMSO-d_6) δ 8.55 (d, *J* = 2.4 Hz, 1H), 8.08 (dd, *J* = 8.6, 2.6 Hz, 1H), 7.69 – 7.62 (m, 2H), 7.57 (dd, *J* = 8.1, 2.0 Hz, 1H), 6.93 (d, *J* = 8.6 Hz, 1H), 5.69 (s, 1H), 3.91 (s, 3H), 3.00 (q, *J* = 7.5 Hz, 2H), 2.47 (s, 3H), 1.57 (s, 6H), 1.20 (t, *J* = 7.5 Hz, 3H). ^{13}C NMR

(126 MHz, DMSO-d_6) δ 163.28, 160.53, 145.45, 144.83, 143.31, 142.60, 137.60, 137.58, 131.22, 130.32, 128.56, 127.80, 123.98, 110.65, 69.27, 53.33, 31.13, 26.43, 16.67, 15.72. ESI–MS calcd for $C_{21}H_{25}N_2O_2S$ $[M + H]^+$ 369.1631 found 369.1632

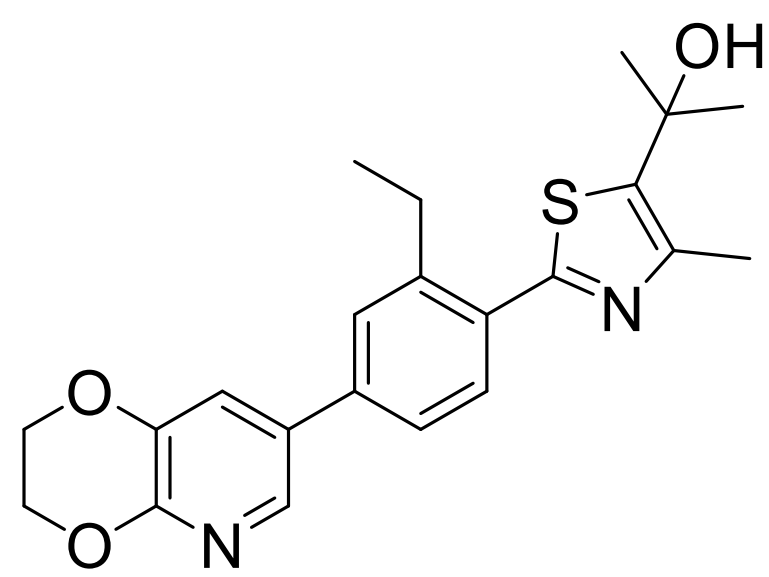

YKJ-308

2-(2-(4-(2,3-Dihydro-[1,4]dioxino[2,3-*b*]pyridin-7-yl)-2-ethylphenyl)-4-methylthiazol-5-yl)propan-2-ol

Yield: 67.9% ^{1}H NMR (500 MHz, DMSO-d_6) δ 8.14 (d, *J* = 2.2 Hz, 1H), 7.71 – 7.62 (m, 3H), 7.56 (dd, *J* = 8.2, 2.0 Hz, 1H), 5.69 (s, 1H), 4.48 – 4.42 (m, 2H), 4.34 – 4.27 (m, 2H), 2.99 (q, *J* = 7.5 Hz, 2H), 2.47 (s, 3H), 1.57 (s, 6H), 1.19 (t, *J* = 7.5 Hz, 3H). ^{13}C NMR (126 MHz, DMSO-d_6) δ 160.49, 150.65, 145.46, 143.34, 142.59, 139.23, 137.19, 137.05, 131.38, 130.36, 130.27, 128.00, 124.13, 122.50, 69.27, 64.82, 63.80, 31.13, 26.40, 16.67, 15.72. ESI–MS calcd for $C_{22}H_{25}N_2O_3S$ $[M + H]^+$ 397.1580 found 397.1580

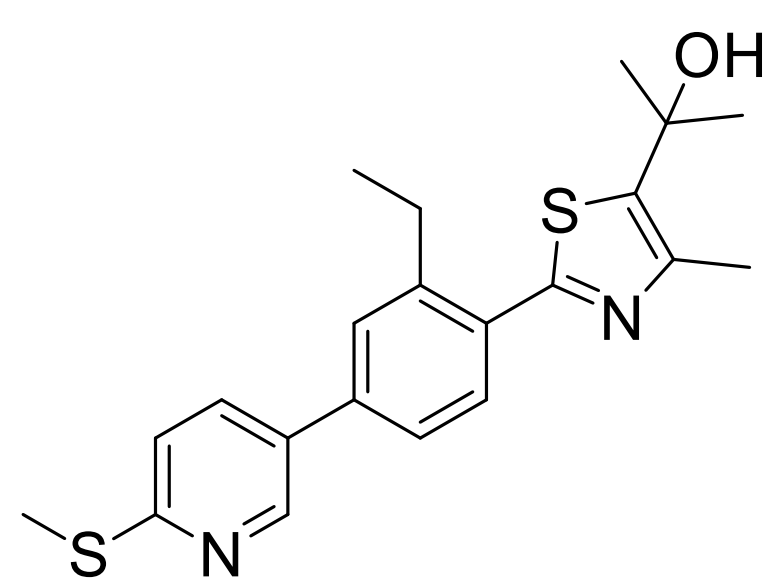

YKJ-309

2-(2-(2-Ethyl-4-(6-(methylthio)pyridin-3-yl)phenyl)-4-methylthiazol-5-yl)propan-2-ol

Yield: 67.1% ^{1}H NMR (500 MHz, DMSO-d_6) δ 8.83 (dd, *J* = 2.5, 0.9 Hz, 1H), 8.02 (dd, *J* = 8.4, 2.4 Hz, 1H), 7.72 – 7.65 (m, 2H), 7.62 (dd, *J* = 8.2, 2.0 Hz, 1H), 7.40 (dd, *J* = 8.4, 0.9 Hz, 1H), 5.69 (s, 1H), 3.01 (q, *J* = 7.4 Hz, 2H), 2.56 (s, 3H), 2.47 (s, 3H), 1.57 (s, 6H), 1.20 (t, *J* = 7.4 Hz,

3H). ^{13}C NMR (126 MHz, DMSO-d_6) δ 160.43, 158.69, 147.22, 145.50, 143.44, 142.69, 137.29, 134.53, 131.64, 130.60, 130.38, 127.98, 124.13, 121.03, 69.28, 31.12, 26.43, 16.67, 15.71, 12.83. ESI–MS calcd for $C_{21}H_{25}N_2OS_2$ $[M + H]^+$ 38.1403 found 385.1403

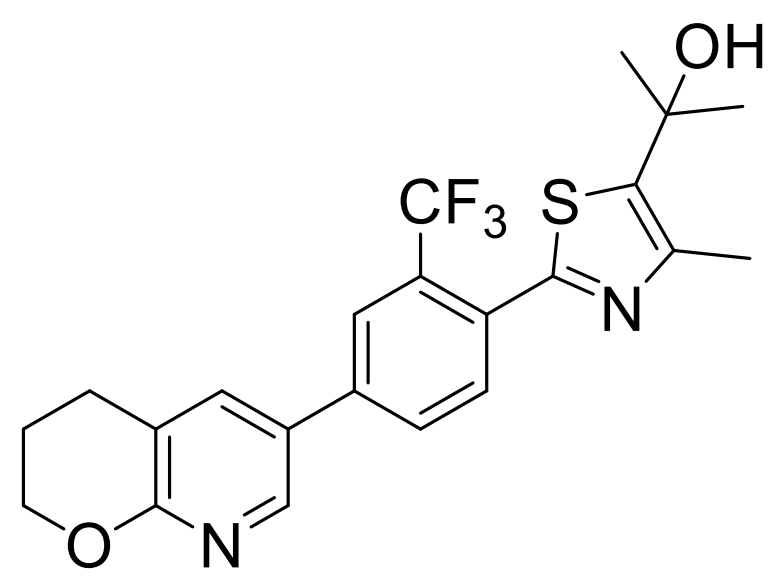

YKJ-310

2-(2-(4-(3,4-dihydro-2H-pyrano[2,3-b]pyridin-6-yl)-2-(trifluoromethyl)phenyl)-4-methylthiazol-5-yl)propan-2-ol

Yield: 90.8% ^1H NMR (500 MHz, DMSO-d_6) δ 8.45 (d, J = 2.6 Hz, 1H), 8.11 – 8.00 (m, 3H), 7.76 (d, J = 8.1 Hz, 1H), 5.77 (s, 1H), 4.37 – 4.30 (m, 2H), 2.88 (t, J = 6.4 Hz, 2H), 2.46 (s, 3H), 2.00 – 1.92 (m, 2H), 1.57 (s, 6H). ^{13}C NMR (126 MHz, DMSO-d_6) δ 161.33, 157.67, 145.51, 145.23, 144.13, 138.44, 137.30, 132.95, 131.06 (d, J = 1.3 Hz), 129.84, 127.45 (q, J = 30.2 Hz), 127.06, 124.28 (q, J = 5.0 Hz), 123.68 (q, J = 274.7 Hz), 117.91, 69.32, 67.05, 30.98, 24.20, 21.21, 16.48. ESI–MS calcd for $C_{22}H_{22}F_3N_2O_2S$ $[M + H]^+$ 435.1349 found 435.1350.

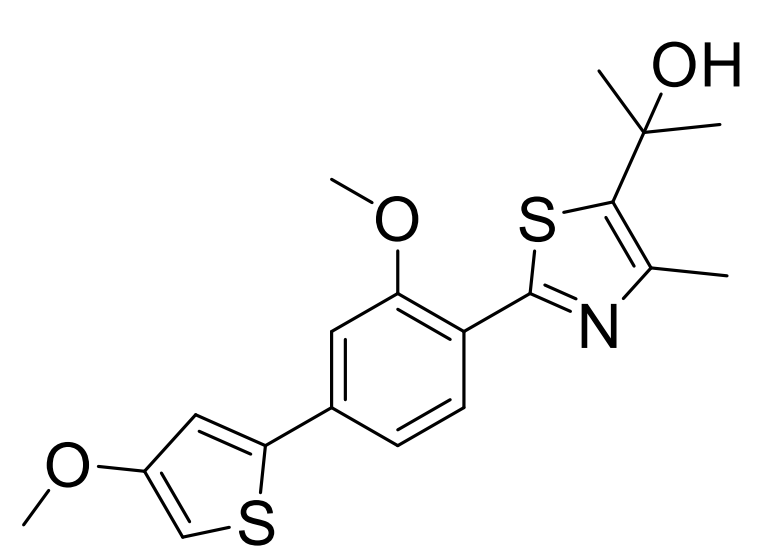

YKJ-311

2-(2-(2-methoxy-4-(4-methoxythiophen-2-yl)phenyl)-4-methylthiazol-5-yl)propan-2-ol

Yield: 85.4% ^1H NMR (500 MHz, DMSO-d_6) δ 8.19 (d, J = 8.2 Hz, 1H), 7.40 (s, 1H), 7.40 (s, 1H), 7.29 (dd, J = 8.2, 1.7 Hz, 1H), 6.66 (d, J = 1.7 Hz, 1H), 5.59 (s, 1H), 4.05 (s, 3H), 3.79 (s, 3H), 2.46 (s, 3H), 1.55 (s, 6H). ^{13}C NMR (126 MHz, DMSO-d_6) δ 158.52, 156.00, 154.86, 144.38,

143.19, 141.00, 135.33, 127.46, 121.24, 117.43, 116.59, 108.30, 98.10, 69.31, 57.24, 56.03, 31.15, 16.64. ESI–MS calcd for $C_{19}H_{22}NO_3S_2$ $[M + H]^+$ 376.1036 found 376.1040.

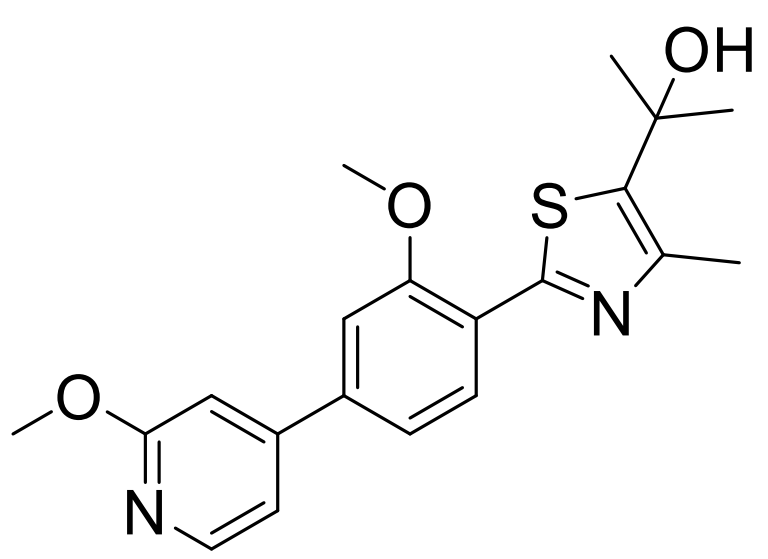

YKJ-312

2-(2-(2-methoxy-4-(2-methoxypyridin-4-yl)phenyl)-4-methylthiazol-5-yl)propan-2-ol

Yield: 88.8% ^{1}H NMR (500 MHz, DMSO-d_6) δ 8.29 (d, J = 8.2 Hz, 1H), 8.25 (d, J = 5.3 Hz, 1H), 7.54 (d, J = 1.7 Hz, 1H), 7.48 (dd, J = 8.2, 1.7 Hz, 1H), 7.41 (dd, J = 5.4, 1.6 Hz, 1H), 7.26 – 7.22 (m, 1H), 5.61 (s, 1H), 4.10 (s, 3H), 3.91 (s, 3H), 2.48 (s, 3H), 1.56 (s, 6H). ^{13}C NMR (126 MHz, DMSO-d_6) δ 164.51, 156.03, 154.68, 149.53, 147.49, 144.53, 143.68, 138.72, 127.41, 122.50, 119.33, 115.15, 110.61, 107.67, 69.33, 56.10, 53.29, 31.12, 16.65. ESI–MS calcd for $C_{20}H_{23}N_2O_3S$ $[M + H]^+$ 371.1424 found 371.1425.

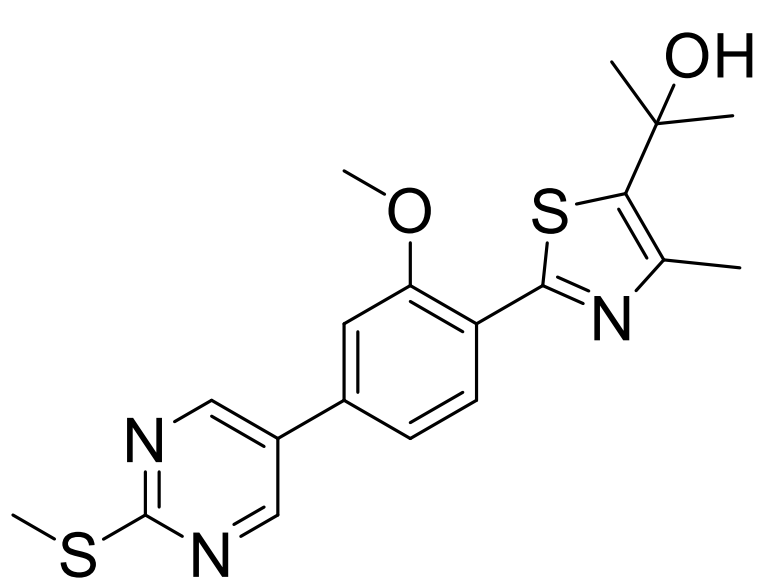

YKJ-313

2-(2-(2-methoxy-4-(2-(methylthio)pyrimidin-5-yl)phenyl)-4-methylthiazol-5-yl)propan-2-ol

Yield: 80.9% ^{1}H NMR (500 MHz, DMSO-d_6) δ 9.10 (s, 2H), 8.29 (d, J = 8.1 Hz, 1H), 7.58 (d, J = 1.8 Hz, 1H), 7.47 (dd, J = 8.2, 1.7 Hz, 1H), 5.61 (s, 1H), 4.09 (s, 3H), 2.58 (s, 3H), 2.48 (s, 3H), 1.56 (s, 6H). ^{13}C NMR (126 MHz, DMSO-d_6) δ 170.56, 156.20, 155.41, 154.72, 144.48, 143.54, 135.38, 127.92, 127.54, 121.79, 118.79, 110.18, 69.33, 56.17, 31.12, 16.66, 13.69. ESI–MS calcd for $C_{19}H_{22}N_3O_2S_2$ $[M + H]^+$ 388.1148 found 388.1149.

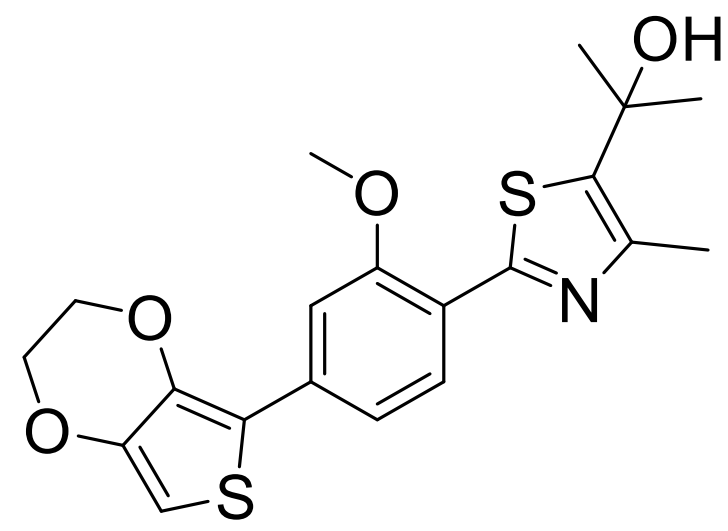

YKJ-314

2-(2-(4-(2,3-dihydrothieno[3,4-b][1,4]dioxin-5-yl)-2-methoxyphenyl)-4-methylthiazol-5-yl)propan-2-ol

Yield: 98.0% ^{1}H NMR (500 MHz, DMSO-d_6) δ 8.18 (d, J = 8.3 Hz, 1H), 7.42 (d, J = 1.7 Hz, 1H), 7.33 (dd, J = 8.2, 1.7 Hz, 1H), 6.70 (s, 1H), 5.57 (s, 1H), 4.39 – 4.34 (m, 2H), 4.30 – 4.24 (m, 2H), 4.01 (s, 3H), 2.46 (s, 3H), 1.55 (s, 6H). ^{13}C NMR (126 MHz, DMSO-d_6) δ 155.77, 155.00, 144.32, 142.89, 142.28, 139.26, 134.56, 127.25, 120.08, 117.85, 115.33, 108.43, 98.99, 69.30, 64.95, 64.12, 55.73, 31.18, 16.64. ESI–MS calcd for $C_{20}H_{22}NO_4S_2$ [M + H]$^+$ 404.0985 found 404.0987.

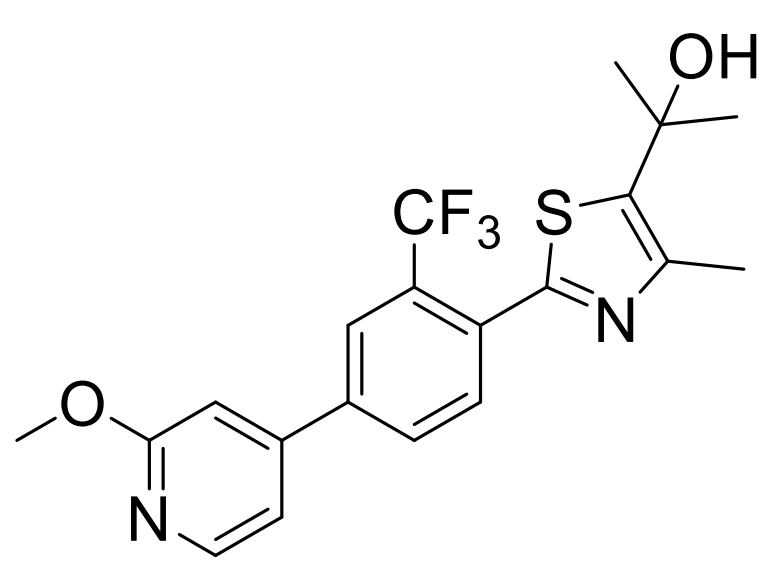

YKJ-315

2-(2-(4-(2-methoxypyridin-4-yl)-2-(trifluoromethyl)phenyl)-4-methylthiazol-5-yl)propan-2-ol

Yield: 94.2% ^{1}H NMR (500 MHz, DMSO-d_6) δ 8.30 (d, J = 5.4 Hz, 1H), 8.18 (d, J = 1.9 Hz, 1H), 8.15 (dd, J = 8.1, 1.9 Hz, 1H), 7.83 (d, J = 8.1 Hz, 1H), 7.45 (dd, J = 5.4, 1.6 Hz, 1H), 7.30 – 7.26 (m, 1H), 5.80 (s, 1H), 3.92 (s, 3H), 2.47 (s, 3H), 1.58 (s, 6H). ^{13}C NMR (126 MHz, DMSO-d_6) δ 164.54, 157.34, 148.04, 147.83, 145.64, 145.62, 138.41, 133.05, 133.01, 130.90, 127.5 (q, J = 30.7 Hz), 125.32 (q, J = 5.5 Hz), 123.55 (q, J = 275.1 Hz), 115.22, 108.10, 69.35, 53.41, 30.94, 16.47. ESI–MS calcd for $C_{20}H_{20}F_3N_2O_2S$ [M + H]$^+$ 409.1192 found 409.1193.

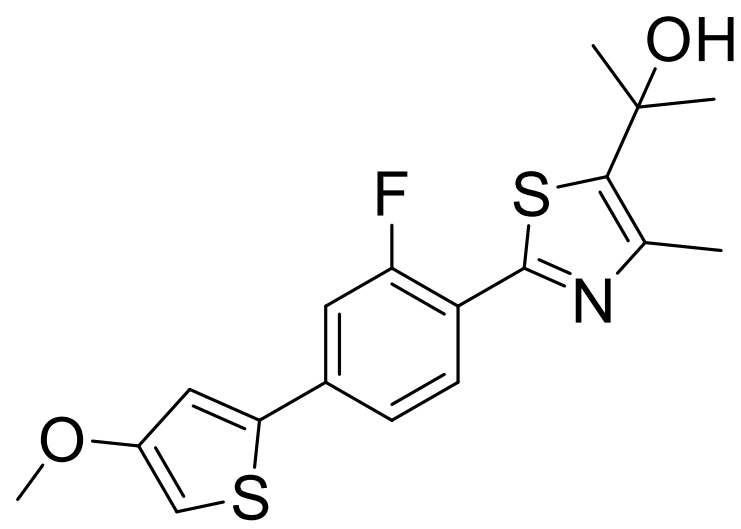

YKJ-316

2-(2-(2-fluoro-4-(4-methoxythiophen-2-yl)phenyl)-4-methylthiazol-5-yl)propan-2-ol

Yield: 90.0% ^{1}H NMR (500 MHz, DMSO-d_6) δ 8.14 (t, J = 8.2 Hz, 1H), 7.70 (dd, J = 12.8, 1.8 Hz, 1H), 7.55 (dd, J = 8.3, 1.8 Hz, 1H), 7.42 (d, J = 1.7 Hz, 1H), 6.71 (d, J = 1.7 Hz, 1H), 5.74 (s, 1H), 3.78 (s, 3H), 2.48 (s, 3H), 1.56 (s, 6H). ^{13}C NMR (126 MHz, DMSO-d_6) δ 159.24 (d, J = 249.4 Hz), 158.59, 153.04 (d, J = 5.4 Hz), 145.31, 144.54 (d, J = 7.4 Hz), 139.42 (d, J = 2.5 Hz), 136.13 (d, J = 9.0 Hz), 128.27 (d, J = 3.6 Hz), 121.24 (d, J = 2.7 Hz), 119.86 (d, J = 12.2 Hz), 117.29, 112.22 (d, J = 23.8 Hz), 98.97, 69.34, 57.26, 30.89, 16.47. ESI–MS calcd for $C_{18}H_{19}FNO_2S_2$ $[M + H]^+$ 364.0836 found 364.0837.

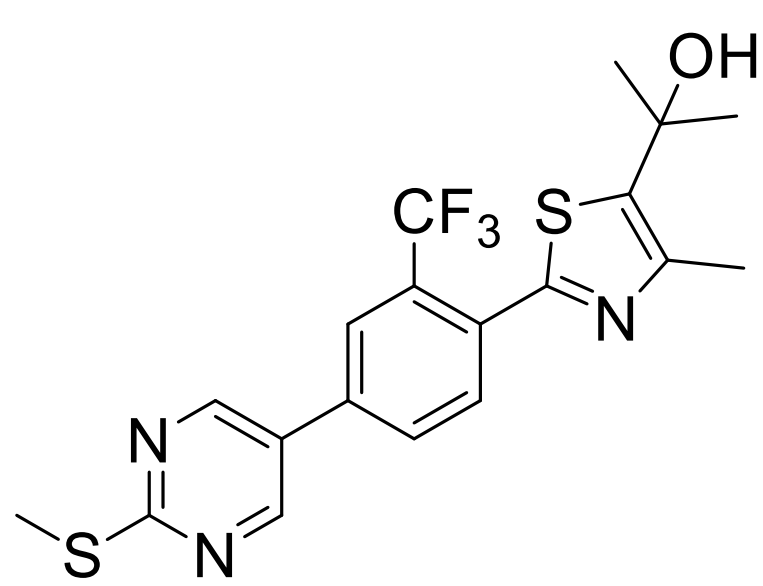

YKJ-317

2-(4-methyl-2-(4-(2-(methylthio)pyrimidin-5-yl)-2-(trifluoromethyl)phenyl)thiazol-5-yl)propan-2-ol

Yield: 84.1% ^{1}H NMR (500 MHz, DMSO-d_6) δ 9.12 (s, 2H), 8.24 (d, J = 1.5 Hz, 1H), 8.14 (dd, J = 8.1, 1.9 Hz, 1H), 7.83 (d, J = 8.1 Hz, 1H), 5.79 (s, 1H), 2.59 (s, 3H), 2.47 (s, 3H), 1.58 (s, 6H). 13C NMR (126 MHz, DMSO-d_6) δ 171.34, 157.43, 155.76, 145.60, 145.54, 135.32, 133.04, 132.24, 130.40, 127.64 (q, J = 31.9 Hz), 126.70, 124.95 (q, J = 6.3 Hz), 123.58 (q, J = 273.8 Hz), 69.35, 30.95, 16.48, 13.71. ESI–MS calcd for $C_{19}H_{19}F_3N_3OS_2$ $[M + H]^+$ 426.0916 found 426.0919.

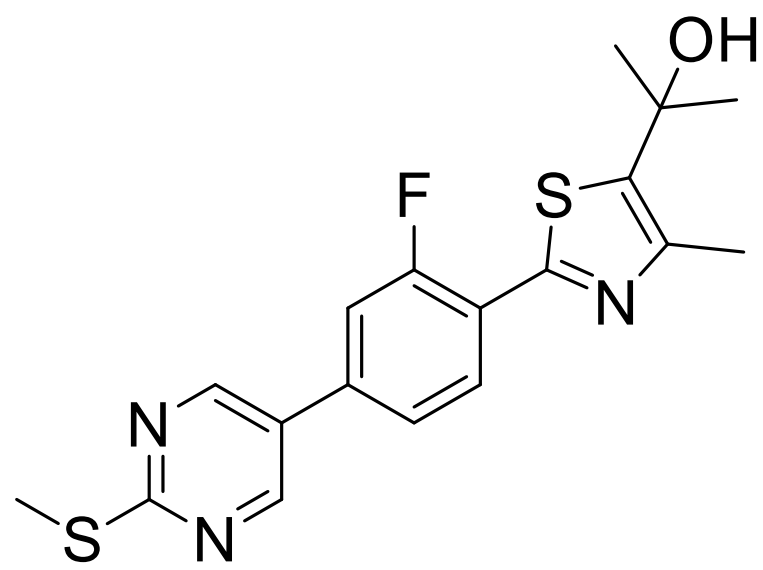

YKJ-318

2-(2-(2-fluoro-4-(2-(methylthio)pyrimidin-5-yl)phenyl)-4-methylthiazol-5-yl)propan-2-ol

Yield: 86.6% ^{1}H NMR (500 MHz, DMSO-d_6) δ 9.08 (s, 2H), 8.24 (t, *J* = 8.2 Hz, 1H), 7.91 (dd, *J* = 12.8, 1.8 Hz, 1H), 7.77 (dd, *J* = 8.3, 1.8 Hz, 1H), 5.77 (s, 1H), 2.58 (s, 3H), 2.50 (s, 3H), 1.57 (s, 6H). ^{13}C NMR (126 MHz, DMSO-d_6) δ 171.13, 159.34 (d, *J* = 249.5 Hz), 155.39, 152.88 (d, *J* = 5.3 Hz), 145.43, 144.94 (d, *J* = 7.4 Hz), 136.28 (d, *J* = 8.8 Hz), 128.31 (d, *J* = 3.4 Hz), 126.58 (d, *J* = 2.1 Hz), 122.82 (d, *J* = 2.8 Hz), 120.66 (d, *J* = 12.2 Hz), 114.12 (d, *J* = 23.5 Hz), 69.37, 30.86, 16.50, 13.70. ESI–MS calcd for $C_{18}H_{19}FN_3OS_2$ $[M + H]^+$ 376.0948 found 376.0950.

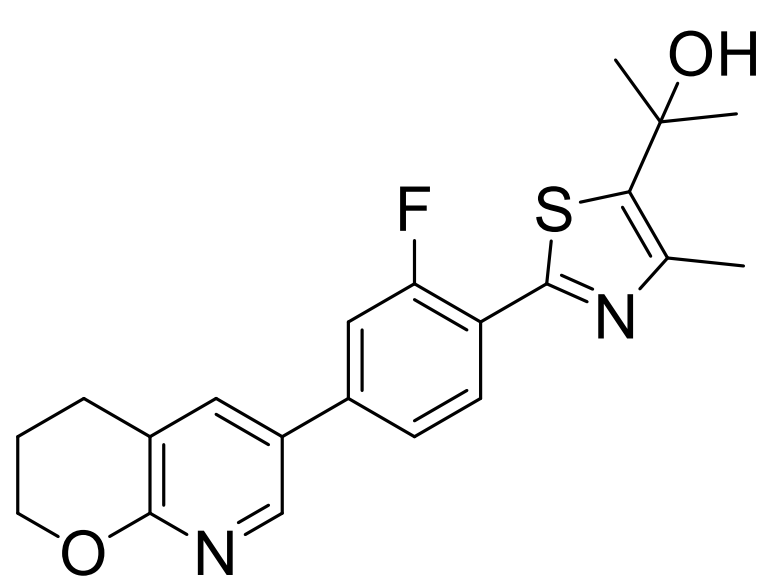

YKJ-319

2-(2-(4-(3,4-dihydro-2H-pyrano[2,3-b]pyridin-6-yl)-2-fluorophenyl)-4-methylthiazol-5-yl)propan-2-ol

Yield: 70.5% ^{1}H NMR (500 MHz, DMSO-d_6) δ 8.43 (d, *J* = 2.6 Hz, 1H), 8.19 (t, *J* = 8.3 Hz, 1H), 7.98 – 7.95 (m, 1H), 7.74 (dd, *J* = 13.0, 1.8 Hz, 1H), 7.65 (dd, *J* = 8.4, 1.8 Hz, 1H), 5.75 (s, 1H), 4.34 – 4.30 (m, 2H), 2.86 (t, *J* = 6.4 Hz, 2H), 2.49 (s, 3H), 1.98 – 1.91 (m, 2H), 1.57 (s, 6H). ^{13}C NMR (126 MHz, DMSO-d_6) δ 161.22, 159.41 (d, *J* = 249.0 Hz), 153.21 (d, *J* = 5.1 Hz), 145.28, 144.43 (d, *J* = 7.2 Hz), 143.92, 139.73 (d, *J* = 8.6 Hz), 136.98, 128.16 (d, *J* = 3.5 Hz), 127.09 (d, *J* = 1.5 Hz), 122.44 (d, *J* = 2.4 Hz), 119.51 (d, *J* = 12.2 Hz), 117.79, 113.53 (d, *J* = 22.9 Hz), 69.35,

67.03, 30.92, 24.23, 21.23, 16.50. ESI–MS calcd for $C_{21}H_{22}FN_2O_2S$ $[M + H]^+$ 385.1381 found 385.1381.

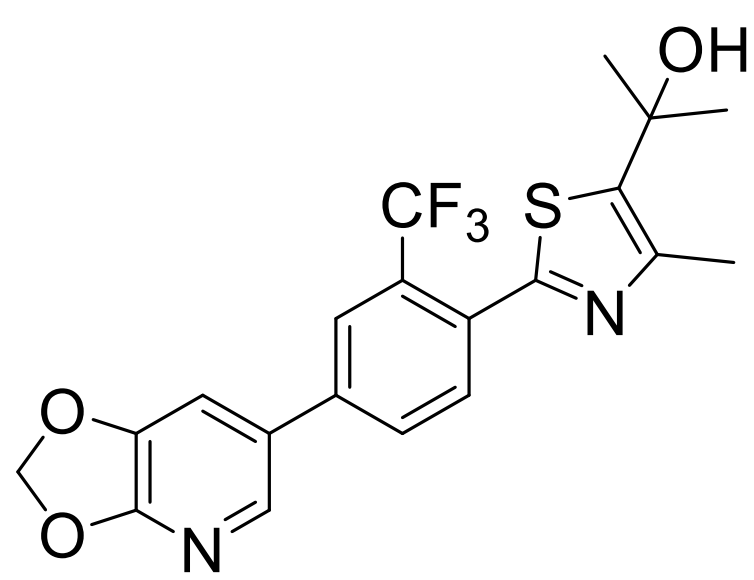

YKJ-320

2-(2-(4-([1,3]dioxolo[4,5-b]pyridin-6-yl)-2-(trifluoromethyl)phenyl)-4-methylthiazol-5-yl)propan-2-ol

Yield: 93.1% ^{1}H NMR (500 MHz, DMSO-d_6) δ 8.09 – 8.00 (m, 3H), 7.79 – 7.75 (m, 2H), 6.23 (s, 2H), 5.78 (s, 1H), 2.46 (s, 3H), 1.57 (s, 6H). ^{13}C NMR (126 MHz, DMSO-d_6) δ 158.39, 157.57, 145.53, 145.32, 140.59, 138.41, 137.39, 132.88, 131.44 (d, *J* = 1.3 Hz), 130.39, 128.72, 127.42 (q, *J* = 30.7 Hz), 124.86 (q, *J* = 5.5 Hz), 123.62 (q, *J* = 274.7 Hz), 113.44, 100.83, 69.33, 30.97, 16.48. ESI–MS calcd for $C_{20}H_{18}F_3N_2O_3S$ $[M + H]^+$ 423.0985 found 423.0985.

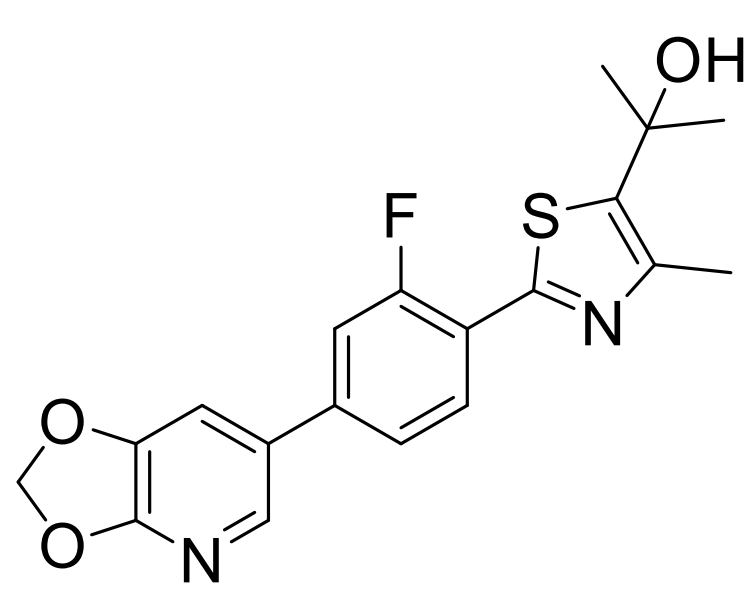

YKJ-321

2-(2-(4-([1,3]dioxolo[4,5-b]pyridin-6-yl)-2-fluorophenyl)-4-methylthiazol-5-yl)propan-2-ol

Yield: quantitative ^{1}H NMR (500 MHz, DMSO-d_6) δ 8.18 (t, *J* = 8.2 Hz, 1H), 8.06 (d, *J* = 2.0 Hz, 1H), 7.78 – 7.70 (m, 2H), 7.65 (dd, *J* = 8.3, 1.9 Hz, 1H), 6.22 (s, 2H), 5.75 (s, 1H), 2.49 (s, 3H), 1.57 (s, 6H). ^{13}C NMR (126 MHz, DMSO-d_6) δ 159.29 (d, *J* = 249.2 Hz), 158.27, 153.09 (d, *J* = 5.2 Hz), 145.31, 144.57 (d, *J* = 7.4 Hz), 140.56, 139.61 (d, *J* = 8.6 Hz), 137.10, 128.68 (d, *J* = 2.1 Hz), 128.08 (d, *J* = 3.5 Hz), 122.91 (d, *J* = 2.7 Hz), 119.85 (d, *J* = 12.1 Hz), 114.11 (d, *J* = 23.2

Hz), 113.07, 100.80, 69.35, 30.89, 16.50. ESI–MS calcd for $C_{19}H_{18}FN_2O_3S$ [M + H]$^+$ 373.1017 found 373.1016.

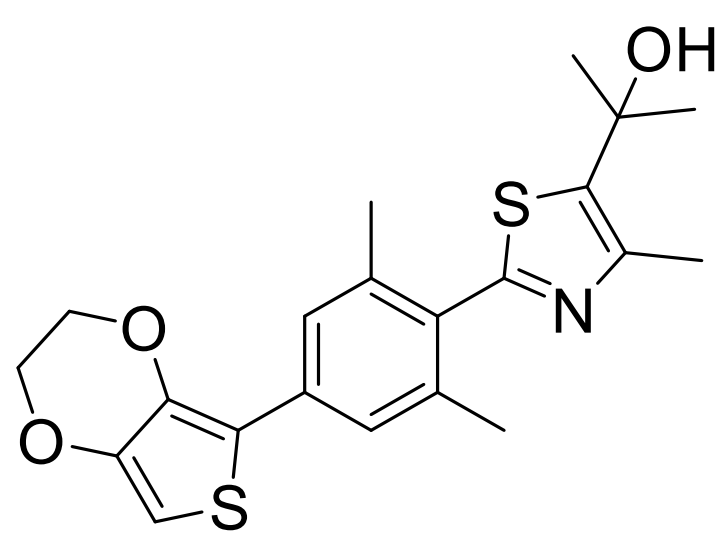

YKJ-322

2-(2-(4-(2,3-dihydrothieno[3,4-b][1,4]dioxin-5-yl)-2,6-dimethylphenyl)-4-methylthiazol-5-yl)propan-2-ol

Yield: 54.1% ^{1}H NMR (500 MHz, DMSO-d_6) δ 7.41 (s, 2H), 6.65 (s, 1H), 5.66 (s, 1H), 4.37 – 4.31 (m, 2H), 4.28 – 4.23 (m, 2H), 2.46 (s, 3H), 2.12 (s, 6H), 1.57 (s, 6H). ^{13}C NMR (126 MHz, DMSO-d_6) δ 159.57, 144.90, 143.48, 142.24, 138.99, 137.46, 133.21, 131.95, 124.19, 115.18, 98.55, 69.28, 64.82, 64.11, 31.22, 20.20, 16.48. ESI–MS calcd for $C_{21}H_{24}NO_3S_2$ [M + H]$^+$ 402.1192 found 402.1192.

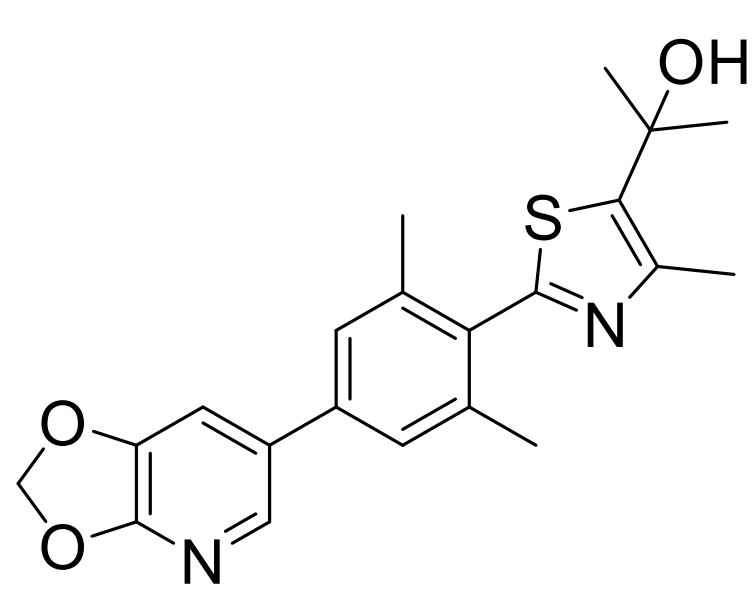

YKJ-323

2-(2-(4-([1,3]dioxolo[4,5-b]pyridin-6-yl)-2,6-dimethylphenyl)-4-methylthiazol-5-yl)propan-2-ol

Yield: 80.8% ^{1}H NMR (500 MHz, DMSO-d_6) δ 7.95 (d, *J* = 2.0 Hz, 1H), 7.62 (d, *J* = 2.0 Hz, 1H), 7.43 (s, 2H), 6.20 (s, 2H), 5.67 (s, 1H), 2.46 (s, 3H), 2.15 (s, 6H), 1.57 (s, 6H). ^{13}C NMR (126 MHz, DMSO-d_6) δ 159.57, 157.77, 144.92, 143.55, 140.38, 137.69, 137.33, 136.66, 133.05, 130.26, 125.67, 113.27, 100.60, 69.29, 31.20, 20.14, 16.50. ESI–MS calcd for $C_{21}H_{23}N_2O_3S$ [M + H]$^+$ 383.1424 found 383.1424.

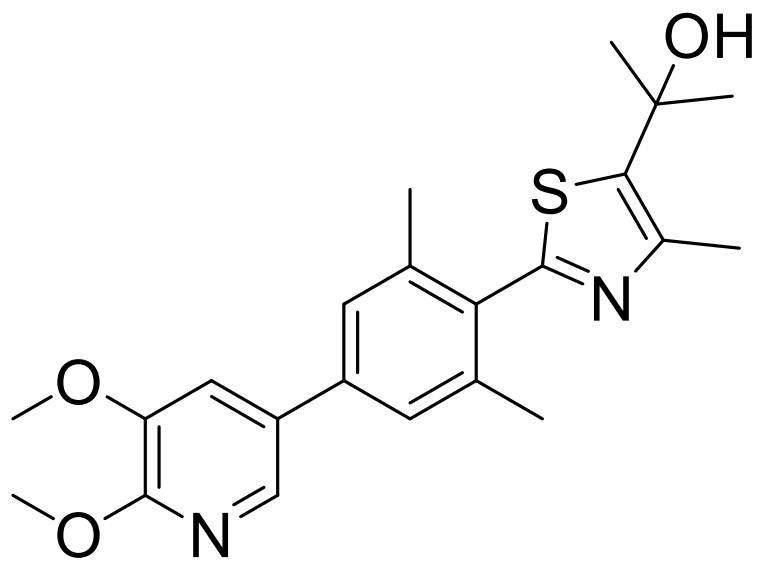

YKJ-324

2-(2-(4-(5,6-dimethoxypyridin-3-yl)-2,6-dimethylphenyl)-4-methylthiazol-5-yl)propan-2-ol

Yield: 40.9% ^{1}H NMR (500 MHz, DMSO-d_6) δ 8.04 (d, J = 2.0 Hz, 1H), 7.56 (d, J = 2.0 Hz, 1H), 7.48 (s, 2H), 5.67 (s, 1H), 3.91 (s, 3H), 3.89 (s, 3H), 2.47 (s, 3H), 2.17 (s, 6H), 1.58 (s, 6H). ^{13}C NMR (126 MHz, DMSO-d_6) δ 159.68, 153.53, 144.91, 143.79, 143.53, 137.63, 137.59, 134.26, 132.82, 129.32, 125.59, 116.69, 69.30, 55.65, 53.18, 31.21, 20.18, 16.50. ESI–MS calcd for $C_{22}H_{27}N_2O_3S$ $[M + H]^+$ 399.1737 found 399.1734.

NMR Spectra of Selected YKJ Compounds
(YKJ-86, YKJ-124, YKJ-262, YKJ-300, and YKJ-305)

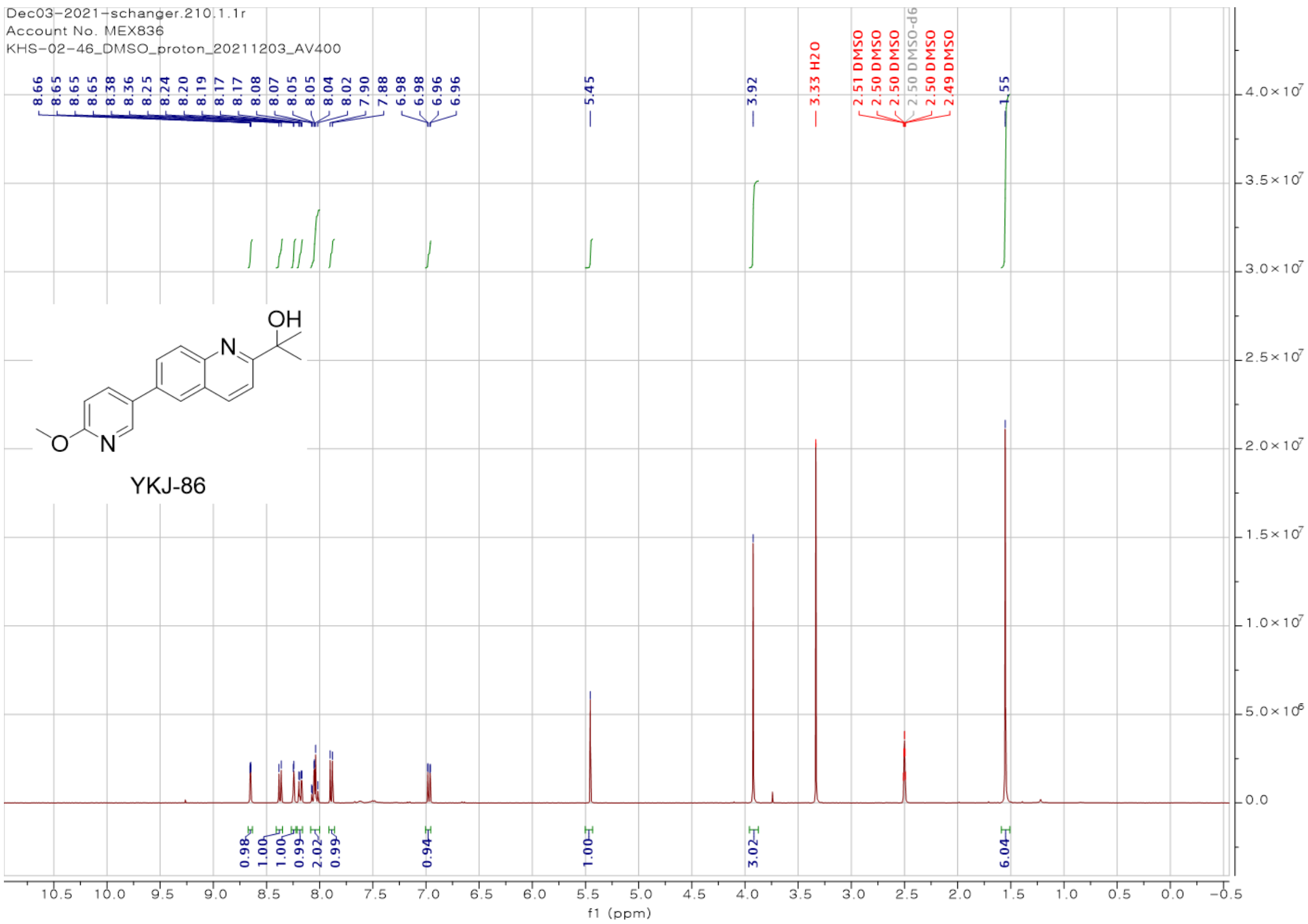

Dec03-2021-schanger.210.1.1r
Account No. MEX836
KHS-02-46_DMSO_proton_20211203_AV400
8.66 8.65 8.65 8.65 8.38 8.36 8.25 8.24 8.20 8.19 8.17 8.17 8.08 8.07 8.05 8.05 8.04 8.02 7.90 7.88 6.98 6.98 6.96 6.96
5.45
3.92
3.33 H2O
2.51 DMSO 2.50 DMSO 2.50 DMSO 2.50 DMSO-d6 2.50 DMSO 2.49 DMSO
1.55
OH
N
O
N
YKJ-86
0.98 1.00 1.00 0.99 2.02 0.99
0.94
1.00
3.02
6.04
f1 (ppm)

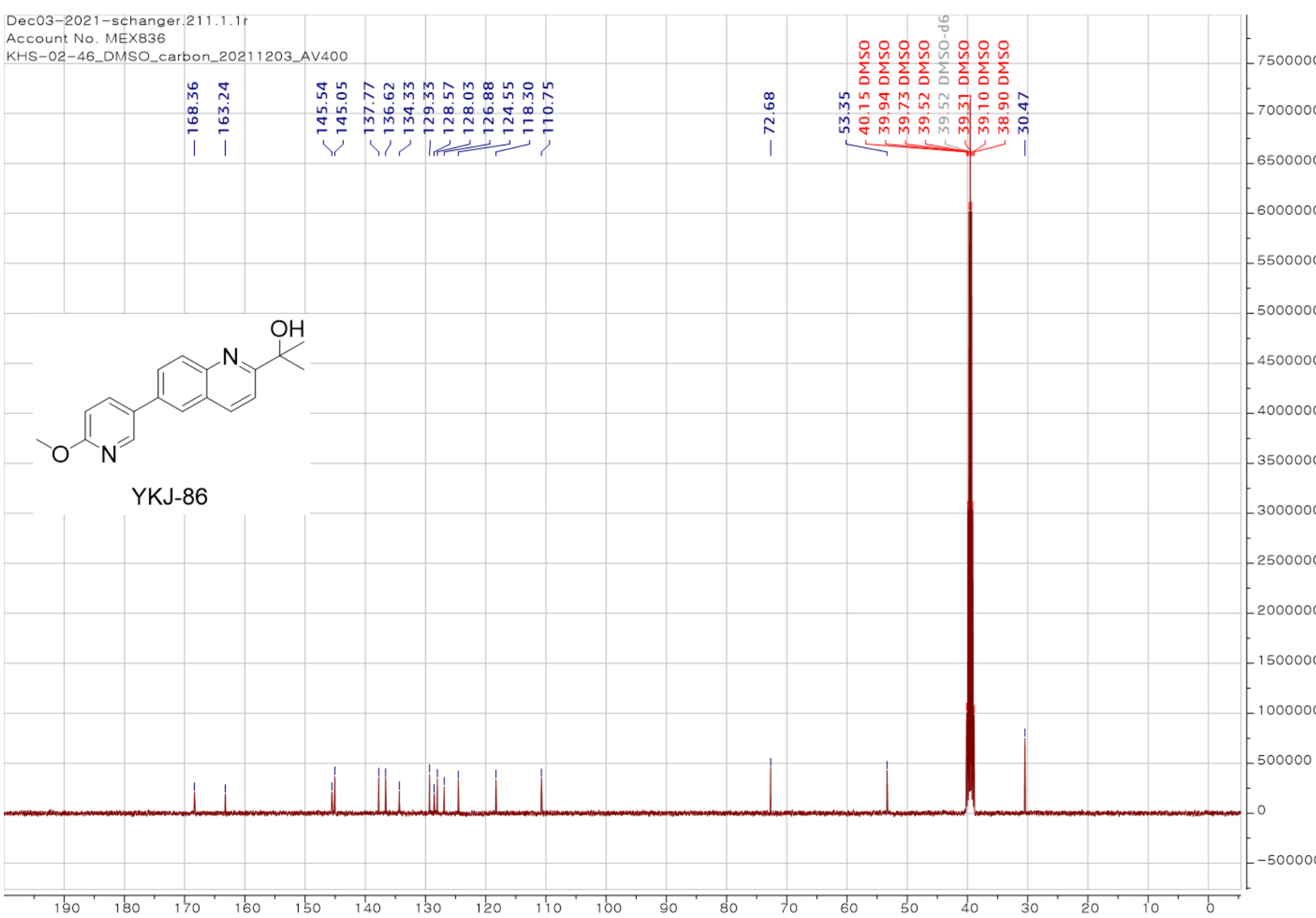

Dec03-2021-schanger.211.1.1r
Account No. MEX836
KHS-02-46_DMSO_carbon_20211203_AV400
168.36
163.24
145.54 145.05
137.77 136.62 134.33 129.33 128.57 128.03 126.88 124.55 118.30 110.75
72.68
53.35
40.15 DMSO 39.94 DMSO 39.73 DMSO 39.52 DMSO 39.52 DMSO-d6 39.31 DMSO 39.10 DMSO 38.90 DMSO
30.47
OH
N
O
N
YKJ-86
f1 (ppm)

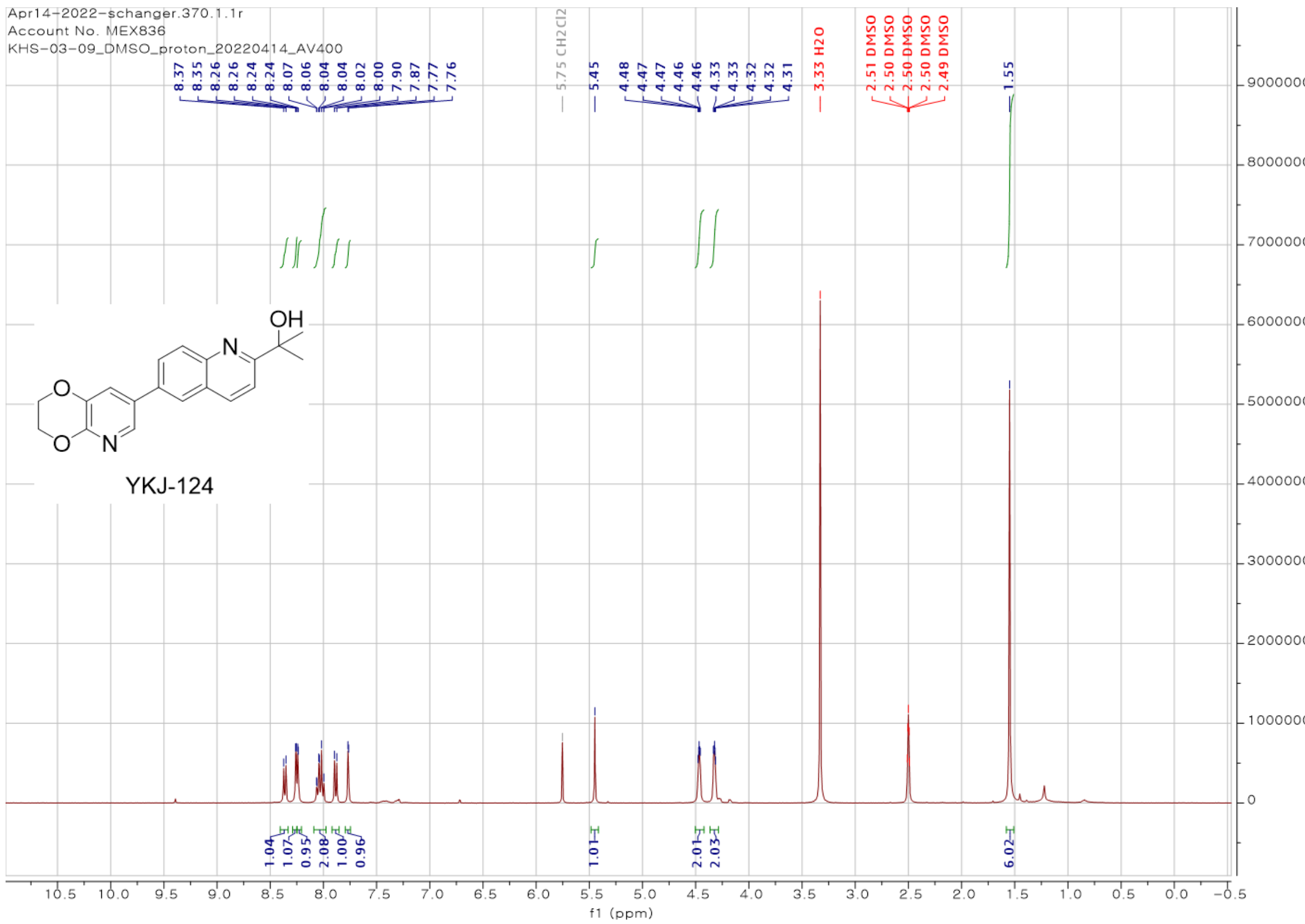
Apr14-2022-schanger.370.1.1r
Account No. MEX836
KHS-03-09_DMSO_proton_20220414_AV400
YKJ-124
f1 (ppm)

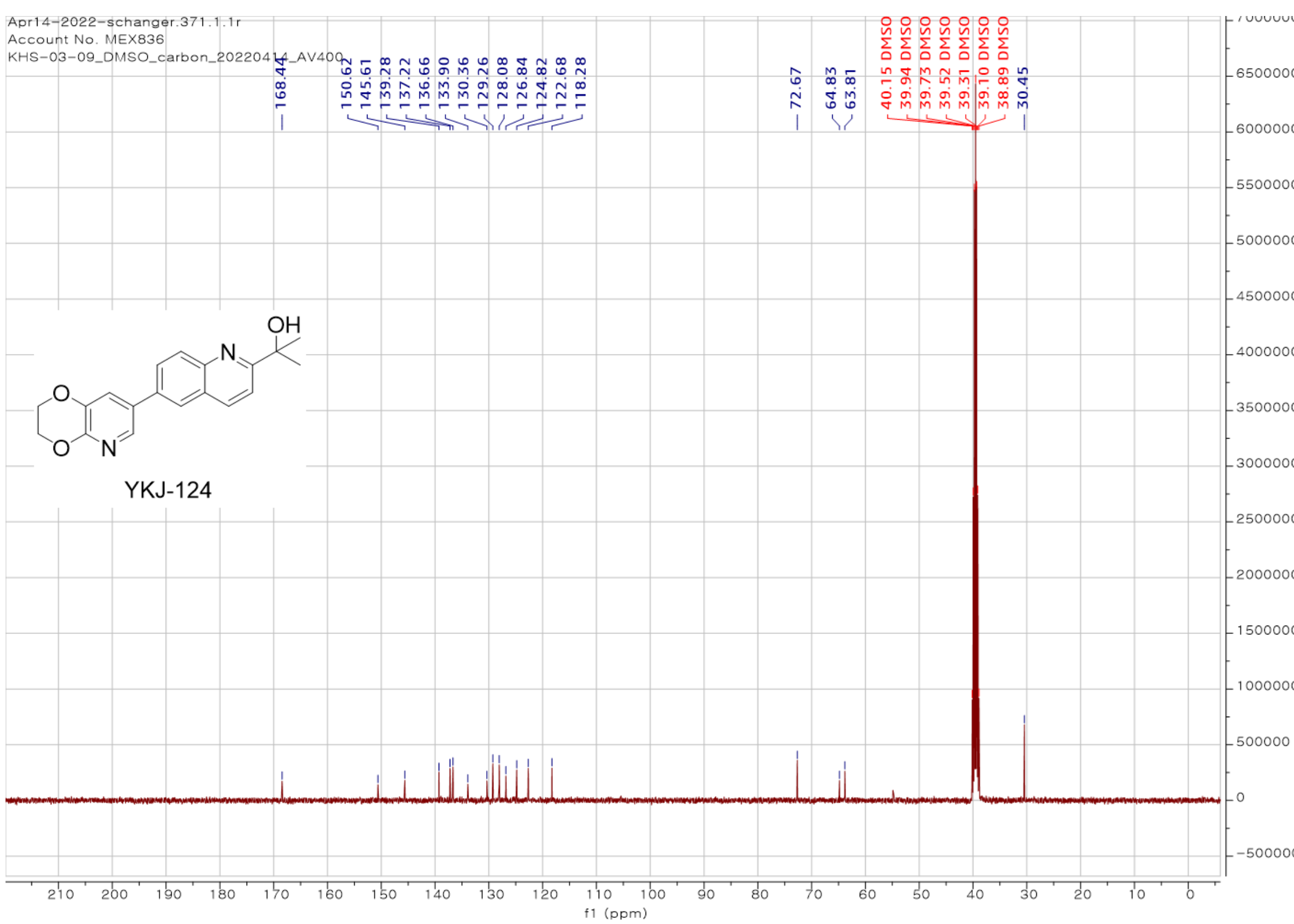
Apr14-2022-schanger.371.1.1r
Account No. MEX836
KHS-03-09_DMSO_carbon_20220414_AV400
YKJ-124
f1 (ppm)

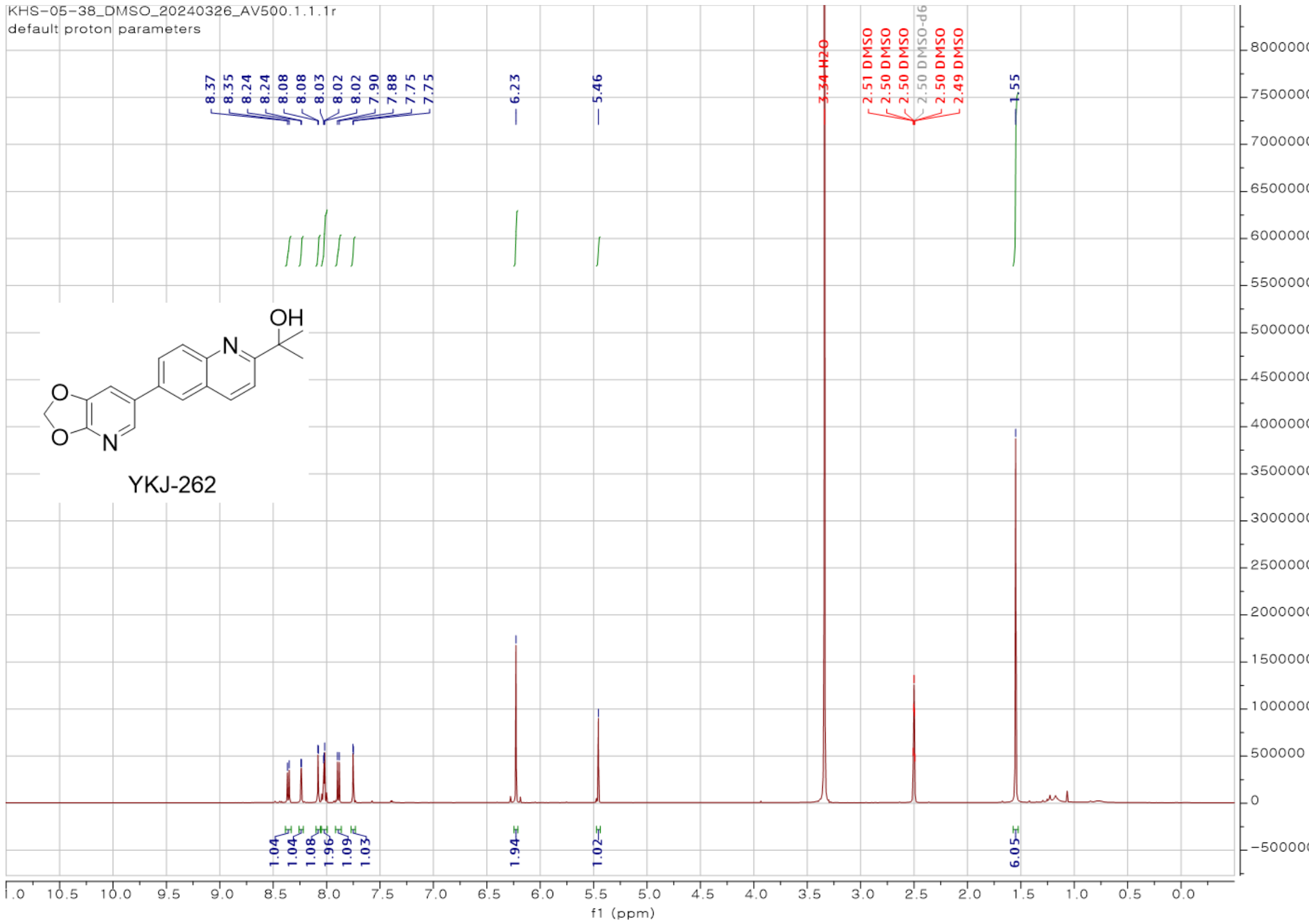
KHS-05-38_DMSO_20240326_AV500.1.1.1r
default proton parameters
8.37
8.35
8.24
8.24
8.08
8.08
8.03
8.02
8.02
7.90
7.88
7.75
7.75
6.23
5.46
3.34 H2O
2.51 DMSO
2.50 DMSO
2.50 DMSO
2.50 DMSO-d6
2.50 DMSO
2.49 DMSO
1.55
OH
N
O
O
N
YKJ-262
1.04
1.04
1.08
1.96
1.09
1.03
1.94
1.02
6.05
f1 (ppm)

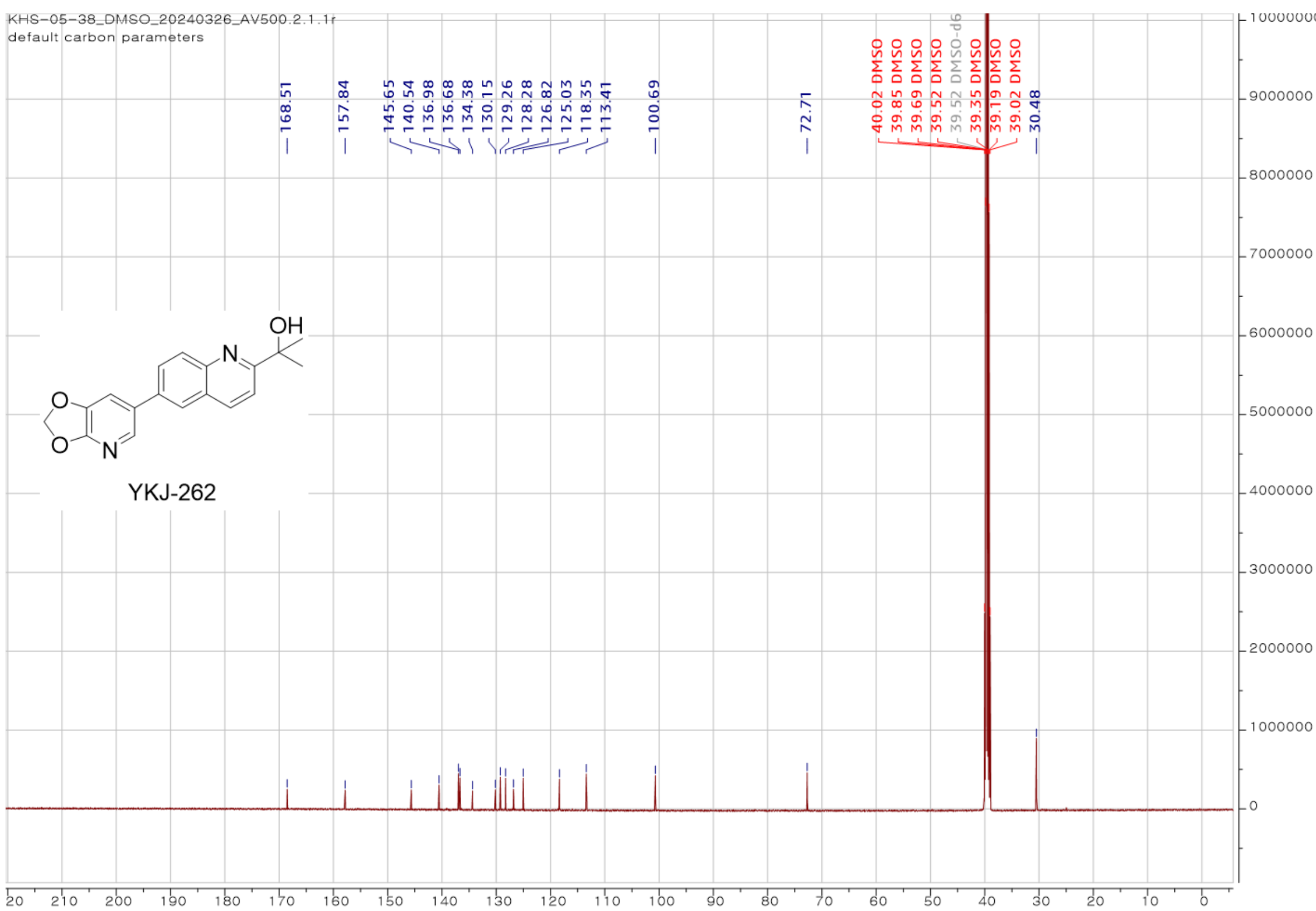
KHS-05-38_DMSO_20240326_AV500.2.1.1r
default carbon parameters
168.51
157.84
145.65
140.54
136.98
136.68
134.38
130.15
129.26
128.28
126.82
125.03
118.35
113.41
100.69
72.71
40.02 DMSO
39.85 DMSO
39.69 DMSO
39.52 DMSO-d6
39.35 DMSO
39.19 DMSO
39.02 DMSO
30.48
OH
N
O
O
N
YKJ-262
f1 (ppm)

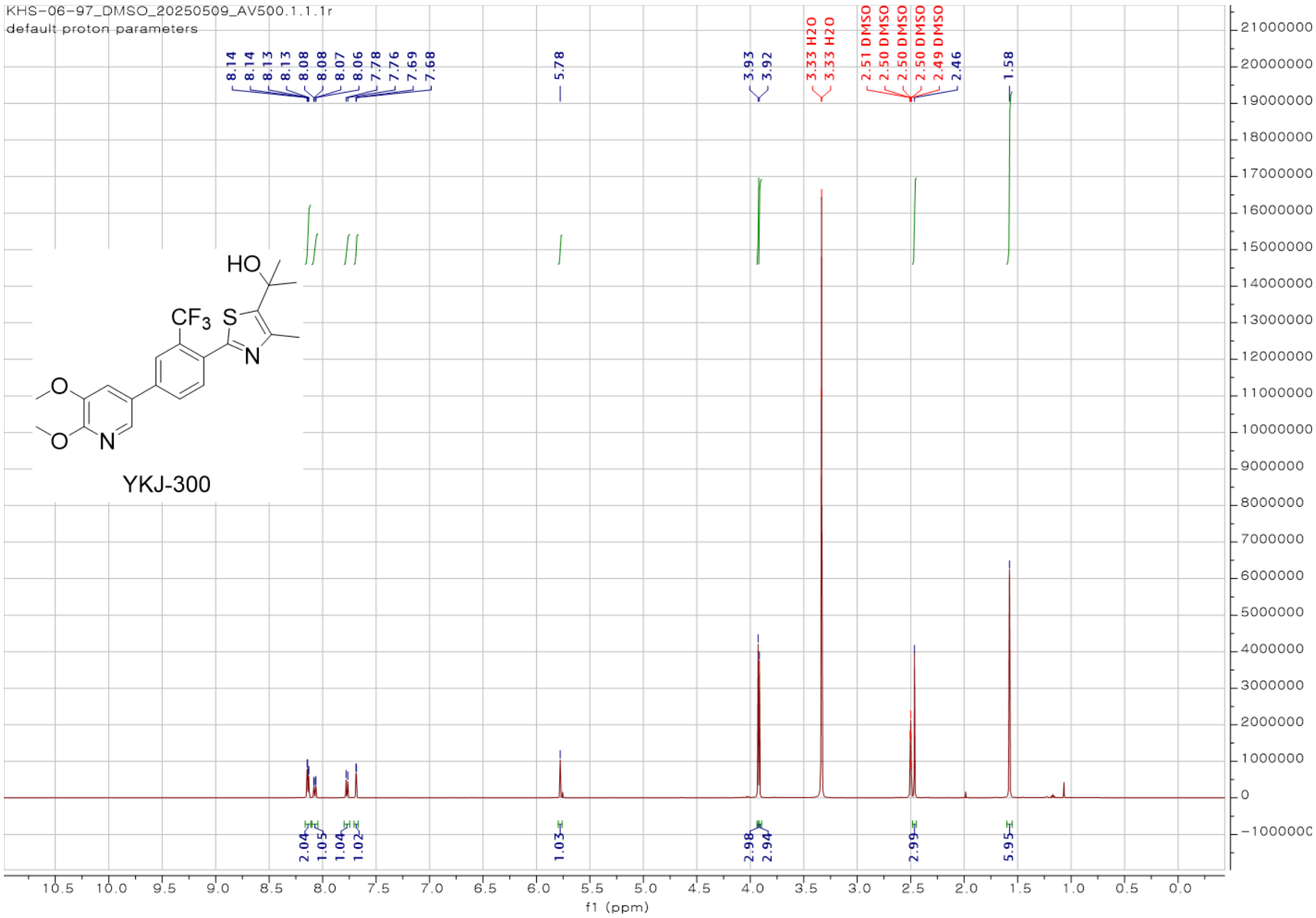
KHS-06-97_DMSO_20250509_AV500.1.1.1r
default proton parameters
8.14
8.14
8.13
8.13
8.08
8.08
8.07
8.06
7.78
7.76
7.69
7.68
5.78
3.93
3.92
3.33 H2O
3.33 H2O
2.51 DMSO
2.50 DMSO
2.50 DMSO
2.50 DMSO
2.49 DMSO
2.46
1.58
YKJ-300
2.04
1.05
1.04
1.02
1.03
2.98
2.94
2.99
5.95
f1 (ppm)

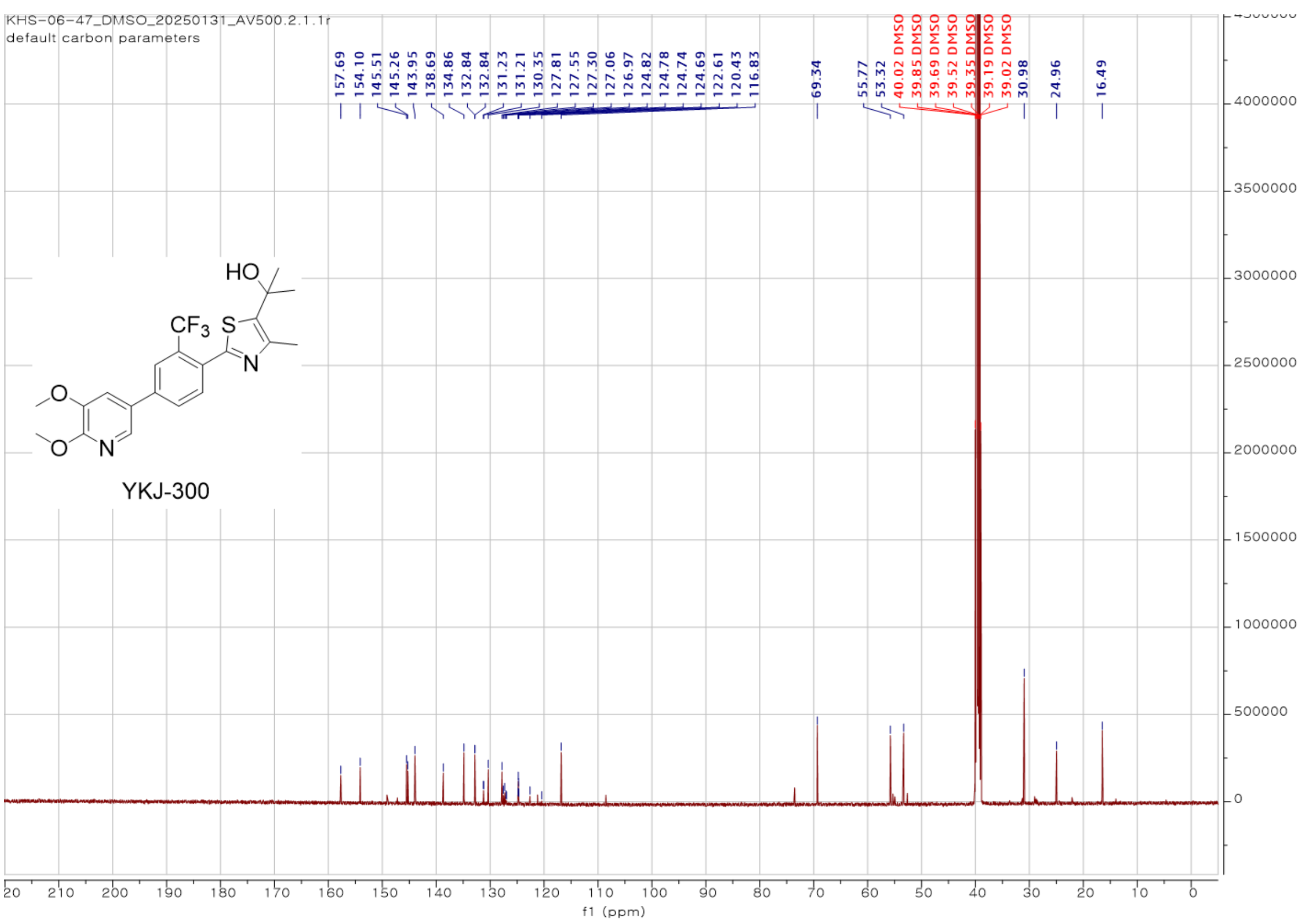
KHS-06-47_DMSO_20250131_AV500.2.1.1r
default carbon parameters
157.69
154.10
145.51
145.26
143.95
138.69
134.86
132.84
132.84
131.23
131.21
130.35
127.81
127.55
127.30
127.06
126.97
124.82
124.78
124.74
124.69
122.61
120.43
116.83
69.34
55.77
53.32
40.02 DMSO
39.85 DMSO
39.69 DMSO
39.52 DMSO
39.35 DMSO
39.19 DMSO
39.02 DMSO
30.98
24.96
16.49
YKJ-300
f1 (ppm)

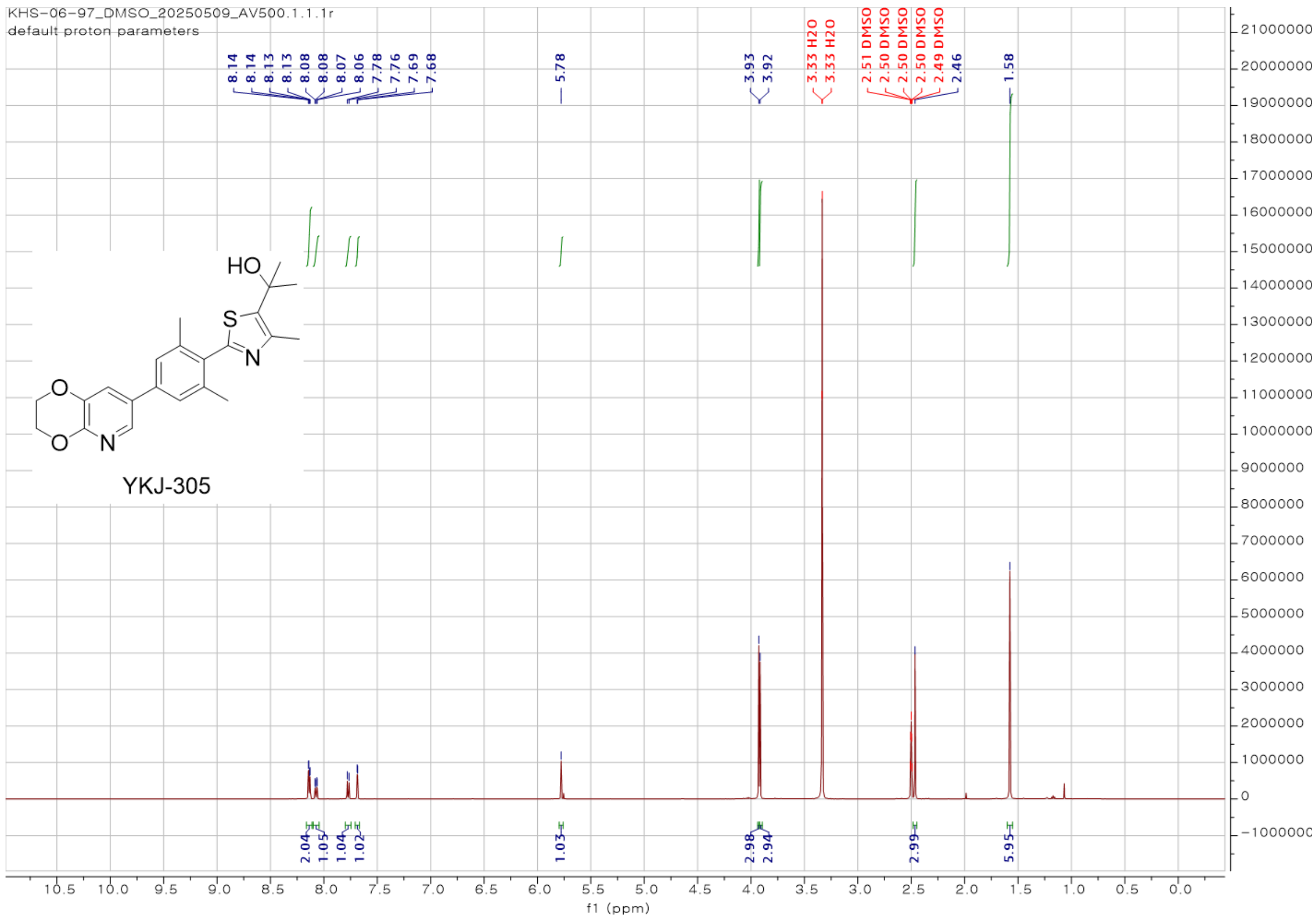
KHS-06-97_DMSO_20250509_AV500.1.1.1r
default proton parameters
8.14 8.14 8.13 8.13 8.08 8.08 8.07 8.06 7.78 7.76 7.69 7.68
5.78
3.93 3.92
3.33 H2O 3.33 H2O
2.51 DMSO 2.50 DMSO 2.50 DMSO 2.50 DMSO 2.49 DMSO 2.46
1.58
HO
S
N
O
O
N
YKJ-305
2.04 1.05 1.04 1.02
1.03
2.98 2.94
2.99
5.95
f1 (ppm)

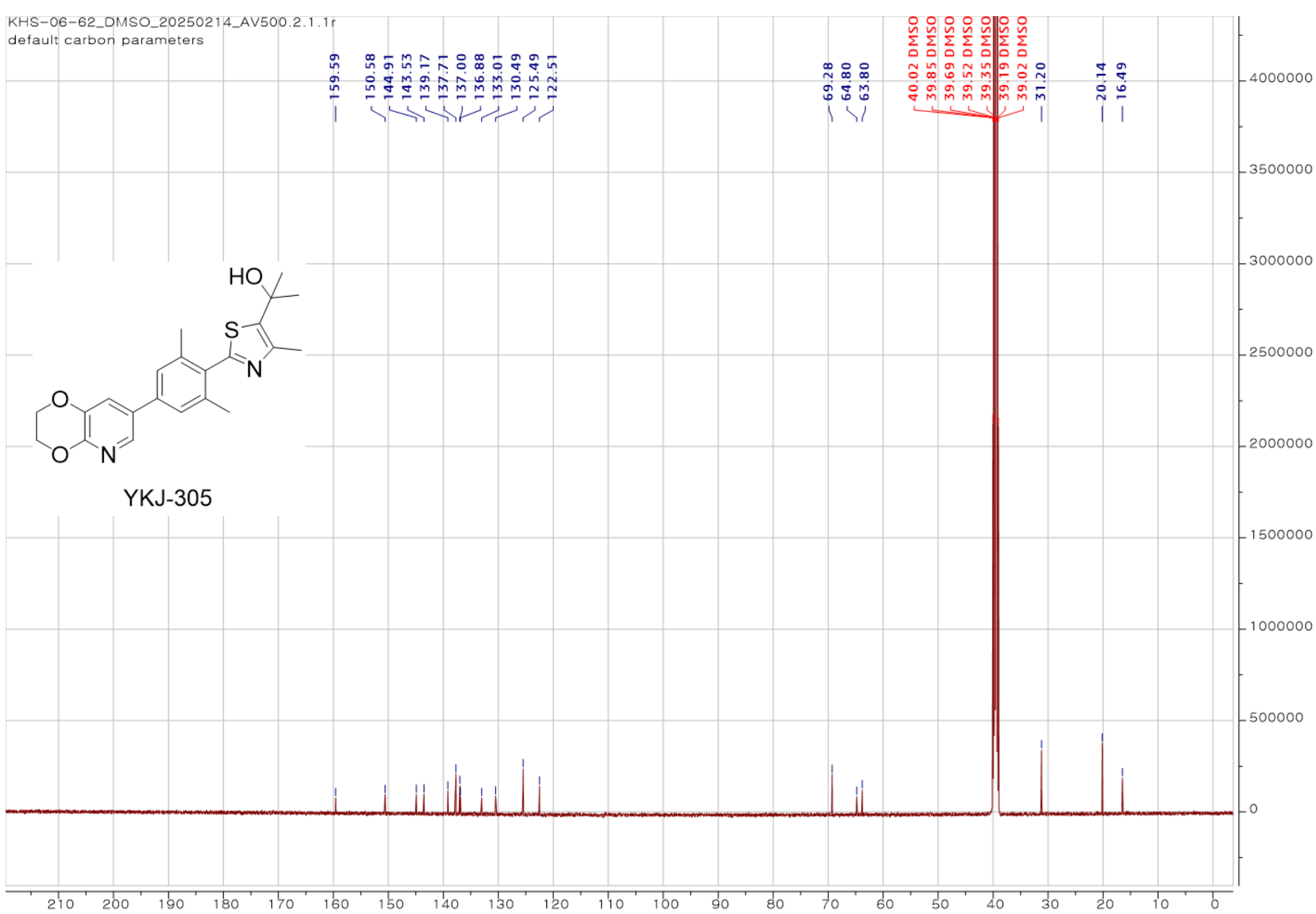
KHS-06-62_DMSO_20250214_AV500.2.1.1r
default carbon parameters
159.59
150.58 144.91 143.53 139.17 137.71 137.00 136.88 133.01 130.49 125.49 122.51
69.28 64.80 63.80
40.02 DMSO 39.85 DMSO 39.69 DMSO 39.52 DMSO 39.35 DMSO 39.19 DMSO 39.02 DMSO
31.20
20.14 16.49
HO
S
N
O
O
N
YKJ-305
f1 (ppm)
